# Novel Molecular Insights into Classical and Alternative Activation States of Microglia as Revealed by Stable Isotope Labeling by Amino Acids in Cell Culture (SILAC)-based Proteomics*[Fn FN2]

**DOI:** 10.1074/mcp.M115.053926

**Published:** 2015-09-30

**Authors:** Harris Bell-Temin, Ashley E. Culver-Cochran, Dale Chaput, Christina M. Carlson, Melanie Kuehl, Brant R. Burkhardt, Paula C. Bickford, Bin Liu, Stanley M. Stevens

**Affiliations:** From the ‡Department of Cell Biology, Microbiology, and Molecular Biology, University of South Florida, 4202 E. Fowler Ave, Tampa, Florida 33620;; §James A. Haley VA Hospital, Research Service and Department of Neurosurgery and Brain Repair, University of South Florida, 12901 Bruce B. Downs Blvd, Tampa, Florida 33612;; ¶Department of Pharmacodynamics, University of Florida, 1345 Center Drive, Gainesville, Florida 32610

## Abstract

Microglia, the resident immune cells of the brain, have been shown to display a complex spectrum of roles that span from neurotrophic to neurotoxic depending on their activation status. Microglia can be classified into four stages of activation, M1, which most closely matches the classical (pro-inflammatory) activation stage, and the alternative activation stages M2a, M2b, and M2c. The alternative activation stages have not yet been comprehensively analyzed through unbiased, global-scale protein expression profiling. In this study, BV2 mouse immortalized microglial cells were stimulated with agonists specific for each of the four stages and total protein expression for 4644 protein groups was quantified using SILAC-based proteomic analysis. After validating induction of the various stages through a targeted cytokine assay and Western blotting of activation states, the data revealed novel insights into the similarities and differences between the various states. The data identify several protein groups whose expression in the anti-inflammatory, pro-healing activation states are altered presumably to curtail inflammatory activation through differential protein expression, in the M2a state including CD74, LYN, SQST1, TLR2, and CD14. The differential expression of these proteins promotes healing, limits phagocytosis, and limits activation of reactive nitrogen species through toll-like receptor cascades. The M2c state appears to center around the down-regulation of a key member in the formation of actin-rich phagosomes, SLP-76. In addition, the proteomic data identified a novel activation marker, DAB2, which is involved in clathrin-mediated endocytosis and is significantly different between M2a and either M1 or M2b states. Western blot analysis of mouse primary microglia stimulated with the various agonists of the classical and alternative activation states revealed a similar trend of DAB2 expression compared with BV2 cells.

Microglia, along with astrocytes, form the backbone of the immune response in the brain. Microglia, in particular, comprise 10–15% of the brain, varying by region and predominating in areas of the midbrain such as the hippocampus and substantia nigra (1). Separated from the systemic immune system by the blood-brain barrier, the brain's immune response relies on the ability of microglia to act as a multifaceted immune cell; microglia are able to sense pathogens, toxins, injury, and cytokine levels, as well as respond in a neurotrophic or neurotoxic manner similar to the macrophage in the systemic immune system (2).

Microglia can respond to insult and injury in a neurotoxic manner (3, 4) where activated microglia are able to induce pro-inflammatory cytokines to recruit other microglia and astrocytes in response to bacterial infection and produce a wide and varied array of factors including reactive oxygen species (ROS)^1^, and reactive nitrogen species (RNS), cytokines and lipid mediators as well as remove cellular debris as a post-infection response through phagocytosis (5). As such, microglia protect themselves from their own toxic products through a series of antioxidant proteins regulated through the actions of nuclear factor, erythroid 2-like 2 protein (NFE2L2) (6). Microglia have been implicated in a growing number of CNS-associated diseases; classically activated microglia have been found in brain regions afflicted with Parkinson's disease, Alzheimer's disease, and AIDS-related dementia (7–9). Microglial activation has also been reported to play a role in brain injury because of chronic alcohol exposure (10–13).

Raivich *et al.* described microglia response and phases as a linear set of stages that microglia pass through in response to injury, pathogens, or antibodies from the systemic immune system that have crossed the blood-brain barrier (14). The first stage is a quiescent resting state, followed by an alert stage characterized by increased expression of integrin-binding proteins, or cell adhesion molecules, such as CD11b. The homing stage of activation that follows is characterized by increased cell mobility and adhesion as microglia target sites of injury or invasion. The fourth stage is a phagocytic stage that is often termed the classical microglia response, characterized by production of neurotoxic factors such as ROS through a cell membrane-bound NADPH oxidase complex and RNS through the action of inducible nitric oxide synthase, iNOS, as well as phagocytosis of cellular debris. The final stage, known as the bystander activation stage, potentiates the microglia response by activating additional microglia through the production and release of pro-inflammatory cytokines such as tumor necrosis factor alpha (TNFα), interferon gamma (IFNγ), and interleukin-6 (IL-6).

Our understanding of the role of microglia has broadened in recent years to include neurotrophic as well as neurotoxic features (15, 16). The presence of activated microglia does not always correlate to an inflammatory state in the local brain region, implying a noninflammatory or possibly neurotrophic role for these microglia. Microglia that display multiple activation states have been observed in the brains of Alzheimer's patients (17). It has been suggested that microglia that enter an inflammatory neurotoxic state first change into a neurotrophic healing response prior to returning to their quiescent resting phase (1). As such, a new schema to describe microglia phenotype was required. M1 phase, which can be triggered *in vivo* and *in vitro* by lipopolysaccharide (LPS) and inflammatory cytokines, has been established to describe classically activated microglial cells that are similar to those found in the fourth and fifth stages of Raivich's microglial hierarchy. Microglia do not return to a resting state without first receiving anti-inflammatory triggers that are released by other microglia. These additional stages have been classified as alternative activation and have multiple healing responses. Microglia can be induced into the first alternative activation stage, M2a, through treatment with interleukin-4 (IL-4), and/or interleukin-13 (IL-13). M2a is a healing phase typified by tissue repair and growth stimulation through the actions of various extracellular matrix factors. Most importantly, M2a microglia act as an anti-inflammatory counterpart to M1 phase microglia by competing for arginine, a nitrogen pool for the production of RNS during M1 phase; M2a phase microglia compete for this pool through the production of arginase-1 (ARG1) which converts arginine into ornithine (18). M2b phase is a mixed activation state that responds to viral infection and activated antibodies characterized by the production of the pro-inflammatory cytokines, TNFα and IL-6, in addition to reduction of IL-12 and increased production of IL-10 (19). M2b phase microglia can be reproduced, *in vitro*, by treating with IL-1β and LPS concurrently or activated IgA complexes, which bind to Fcγ receptors. M2c phase microglia can be induced through IL-10 exposure *in vivo* and *in vitro*, and the emergence of M2c microglia shuts down microglial immune response.

In order to study microglia in a laboratory setting, enriched *ex vivo* microglia, primary microglia, or immortalized cell lines are required. BV2 immortalized mouse microglia have been described as producing 41% of the cytokines and chemokines produced by *ex vivo* cells as compared with 96% coverage by primary microglia. However, Wilcock *et al.* showed that BV2 cells were successful at producing the classical activators for all four microglia activation stages as measured by real-time polymerase chain reaction (17). In addition, proteomic analysis of pathway level changes may be able to smooth over the lack of full expression through high levels of accurate protein quantification.

Because of their importance in immune response and possible role in multiple disease states, a thorough investigation of the differential proteomic expression in the various microglial activation states is required. Using SILAC-labeled immortalized BV2 microglial cells treated with activators of the various activation stages, a proteome profile that includes the major canonical microglial pathways across all four activation states, providing crucial information as to where in these pathways of various states diverge, was established. In addition, using the differential protein expression data, a novel marker of microglia activation, DAB2, was identified and confirmed in primary mouse microglia through Western blot analysis. The abundance of this protein, as well as other differentially expressed proteins identified in this study, may prove as novel indicators in differentiating and categorizing activated microglia in the brain.

## EXPERIMENTAL PROCEDURES

### 

#### 

##### Chemicals and Reagents

All chemicals and reagents were obtained from Fisher Scientific unless otherwise noted.

##### Cell Culture and Treatments

BV2 mouse microglial cells were grown in a base media of SILAC amino acid-denuded DMEM and supplemented with sodium pyruvate, glutamine, 5% dialyzed FBS, 100 μg/ml penicillin, and 100 U/ml streptomycin (20). Cells were grown in media supplemented with either 100 μg/ml each of heavy ^13^C_6_ l-lysine and ^13^C_6_ l-arginine or 100 μg/ml each of unlabeled l-arginine and l-lysine (Cambridge Isotope, Tewksbury, MA) depending on if they were to receive a treatment or serve as control media (21). Cells were allowed seven doublings in growth media before a 24 h period in serum free media, or 1% FBS in the case of the M1 phase LPS treatments, prior to 24 h treatment. This labeling procedure allowed for >99% incorporation of the amino acid labels into the microglial proteome.

All treatments were performed in triplicate. M1 phase was stimulated through the addition of 30 ng/ml LPS. M2a phase was triggered through simultaneous treatment with 30 ng/ml IL-4 and 10 ng/ml IL-13. For the M2b treated cells, Aβ1–42 (AnaSpec #24224, Fremont, CA) was resuspended in 35 μl of 1% NH_4_OH, and brought to a concentration of 1 mg/ml in PBS. Aβ1–42 (1 mg/ml) was aggregated for 48 h at 37 °C, ∼pH 7, followed by sonication (3 × 10 s at 20% amplitude) to form microaggregates. Aβ1–42-IgG complexes were generated by incubating 1.25 μg/ml Aβ1–42 microaggregates with 2.5 μg/ml anti-Aβ monoclonal antibody (Invitrogen 13–0100Z, Carlsbad, CA) in 8 ml of DMEM (2% FBS) for 3 h at 37 °C (22). Cells were then treated in the antibody complex-containing media. M2c activation was triggered through treatment with 10 ng/ml of IL-10. Control cells were treated in serum-free light SILAC media or 1% FBS for comparison with the LPS-treated cells.

Primary mouse microglia were obtained from ScienCell (Carlsbad, CA, #M1900) and were cultured using ScienCell Microglia Media (#M1901). Approximately 3.0E6 cells were distributed across five wells of a 6-well plate. The primary cells received the same treatments as BV2 cells for control, M2a, M2b, and M2c states. For the M1 stimulation, 5 ng/ml LPS was used instead of 30 ng/ml, which was used for the BV2 cells, because primary microglia are known to be markedly more responsive to LPS stimulation than immortalized microglial cells (23). Cells were treated for 24 h and collected using a rubber policeman. Cells were lysed as described below.

##### Cell Lysis, Digestion, Desalt, and Fractionation

Prior to lysis, cells were collected with a rubber policeman and washed three times in PBS prior to lysis. Cells were lysed in 4% SDS in 100 mm Tris-HCl, pH 7.6 and 100 mm dithiothreitol at 95 °C for 4 mins. Protein concentration was determined using the 660 nm Protein Assay supplemented with Ionic Detergent Compatibility Reagent (ThermoFisher Scientific, Waltham, MA). Samples were combined followed by buffer exchange and digestion utilizing the FASP method described by Wisniewski *et al.* (2009) and Manza *et al.* (2005) (24, 25). Briefly, cells were exchanged into 8 m urea and alkylated with 10 mm iodoacetamide in a 30 kDa Microcon Forensic Column (Millipore, Billerica, MA) across multiple 14,000 × *g* centrifugations prior to exchange into 25 mm ammonium bicarbonate. Proteins were digested overnight using a 1:100 ratio of Mass Spectrometry Grade, TPCK-treated trypsin (Promega, Madison, WI) prior to collection into a new tube. Samples were desalted on C18 SPE columns as described previously (26, 27). Samples were concentrated in a vacuum concentrator prior to resuspension for strong cation exchange fractionation.

Samples were fractionated on a U3000 offline HPLC fitted with a 200 mm x 1 mm I.D. polysulfoethyl strong cation exchange column employing a gradient of 10 mm ammonium formate to 200 mm ammonium formate in 25% ACN for 30 min as described previously (26, 27). Six fractions were pooled from previous optimization of unique peptides per fraction from fractionation data of 1 min fractions of unlabeled BV2 peptides.

Samples were concentrated again in a vacuum concentrator prior to resuspension in 0.1% formic acid prior to mass spectrometric analysis.

##### LC-MS/MS

Fractions were separated on an inline 10 cm × 75 μm I.D. reversed-phase column packed with 5 μm C18 material with 300 Å pore size using a 180 min gradient of 3–32% acetonitrile in 0.1% formic acid. Inline mass spectrometric analysis was performed on an Orbitrap XL (Thermo). Survey scans used a resolving power of 60,000, and the top ten abundant peaks were selected for MS/MS fragmentation and analysis. An exclusion list of 100 members, with early expiration after three readings, and monoisotopic precursor selection (MIPS) were employed.

##### Statistical, Pathway, and Upstream Regulator Analysis

High resolution mass spectrometric data were analyzed on the MaxQuant processing suite, version 1.4.1.2 (28–30). Spectra were identified using the MaxQuant built-in peptide identification algorithm, Andromeda, after recalibration to sub 1 ppm accuracy levels, and compared against the Uniprot reference data set for *Mus musculus* (45,182 proteins). Variable modifications included oxidation of methionine and N-terminal protein acetylation whereas fixed modifications included carbamidomethylation of cysteine. Trypsin was specified as the digestion protease with the possibility of two missed cleavages. Identifications were assigned using a target/decoy strategy employing reversed false positives and a threshold of false discovery rate of 1% for peptides and proteins (31). Intensities for all peptides were assigned by MaxQuant using full scan mass spectra, and ratios between heavy and light SILAC partners were calculated. Ratios were normalized assuming an average ratio of one across all peptides. After a treatment to control ratio was calculated, protein group ratios were assigned using the median peptide expression ratio of unique peptides only. Raw files of these data are publicly available from Chorus Project, project #888, https://chorusproject.org. Annotated MS/MS spectra for single peptide-based identifications are included as Supplemental MS/MS Spectra. The median value of all protein expression ratios were calculated and analyzed using the Perseus processing suite. Standard error for each individual protein ratio, as well as all peptide and protein data, are included as supplemental Tables S5–S7. Significance was established at a *p* value of less than 0.05 using the Significance A outlier test in Perseus. Proteins that were identified as significant were entered into the Ingenuity Pathway Analysis suite (IPA; Qiagen, Valencia, CA) to determine localization, molecular function, and protein interaction pathways (32). Upstream Regulator analysis was also performed to predict activity of upstream regulators based on the expression levels of downstream targets in relation to their known upstream regulator expression changes. Analysis determined the significance of overlap of detected targets through a Fisher's exact test (*p* < 0.05) in addition to a z-score algorithm to predict the direction of upstream regulator change (significant activation or inhibition established at a z-score of ≥ 2 or ≤ −2, respectively).

To compare the data from this study to prior studies of the BV2 proteome or transcriptome, KEGG pathway analysis and PANTHER analysis were performed using *Mus musculus* as the reference and default settings.

##### Western Blotting

The BV2 cell treatments described above were performed and the cells collected and lysed. Protein concentrations were determined using the 660 nm Protein Assay plus IDCR. For the BV2 cell lysates, 5 μg of protein was loaded onto a 4–20% Mini-PROTEAN TGX gel (BioRad, Hercules, CA, #456–1096) and run at 125 V for 75 min. Protein was transferred to a PVDF membrane using the high molecular weight predefined program on a Turbo Transfer semi-dry transfer apparatus (BioRad). The blot was probed with 1:500 DAB2, (rabbit, Santa Cruz, Dallas, TX), followed by 1:5,000 anti-rabbit HRP and, afterward, stripped using a stripping buffer (62.5 mm Tris-HCl, pH 6.8, 2% SDS, and 100 mm β-mercaptoethanol) at 65 °C for 45 min with gentle rocking. The same blot was then reprobed with 1:1,000 iNOS (rabbit, Cell Signaling, Danvers, MA), 1:500 ARG1 (rabbit, Santa Cruz), and 1:2,000 GAPDH (rabbit, Cell Signaling) with a stripping step between each antibody. Densitometry was performed using Image J to both normalize the loading between samples using GAPDH and quantify the abundance of DAB2 in each sample.

Primary microglia were treated as described above. The cells were collected, lysed, and 5 μg of protein were loaded onto a 4–20% Mini-PROTEAN TGX gel (Bio-Rad, #456–1096) and run at 125 V for 75 min. Protein was transferred to a PVDF membrane using the high molecular weight predefined program on a Turbo Transfer semi-dry transfer apparatus (BioRad). The blot was probed with 1:500 DAB2, (rabbit, Santa Cruz), followed by 1:5,000 anti-rabbit HRP and, afterward, stripped using a stripping buffer (62.5 mm Tris-HCl, pH 6.8, 2% SDS, and 100 mm β-mercaptoethanol) at 65 °C for 45 min with gentle rocking. The same blot was then reprobed with 1:1,000 iNOS (rabbit, Cell Signaling), 1:500 ARG1 (rabbit, Santa Cruz), and 1:2,000 GAPDH (rabbit, Cell Signaling) with a stripping step between each antibody. Densitometry was performed using Image J to both normalize the loading between samples using GAPDH and quantify the abundance of DAB2 in each sample.

##### Luminex Cytokine Assay

Cytokine levels were determined using a multiplexed bead immunoassay (R&D Systems Inc, Minneapolis, MN, catalog number Custom 03). Cytokines were measured using the high sensitivity mouse cytokine magnetic bead assay for TNFα, IL-6, IL-12, and IL-10. A volume of 50 μl of the microparticle mixture, containing magnetic beads and cytokine antibodies against the three target analytes, were added to every well of the microplate. The plate was prepared by adding 50 μl of each standard or control into appropriate wells. The 0 pg/ml standard consisted of 50 μl of calibrator diluent. A volume of 50 μl of the cell supernatant samples was added in triplicate to the plate. The plate was sealed, wrapped in foil, and incubated with agitation of 200–400 rpm on a plate shaker for 2 h at room temperature. The plate was placed on a hand-held magnetic plate to allow complete settling of magnetic beads. The well contents were removed by gently decanting the plate and tapping on an absorbent towel to remove residual liquid. The plate was washed with 100 μl of 1x wash buffer. The magnetic plate resting/wash process was repeated for a total of three times. A volume of 50 μl of the Biotin Antibody mixture was distributed to each well. The plate was sealed, protected from light, and incubated with agitation of 200–400 rpm on a plate shaker for 1 h at room temperature. A volume of 50 μl of Streptavidin conjugated to the fluorescent protein, R-Phycoerythrin, was added to each well and incubated at room temperature, protected from light, on a plate shaker set to 200–400 rpm for 30 min. The well contents were removed and the plate washed three times. Lastly, 100 μl of wash buffer was added to all wells and the beads will be resuspended on a plate shaker set at 200–400 rpm for 2 min. The plate was analyzed on the Luminex Magpix using a five-parameter logistic curve-fitting method for calculating cytokine concentrations in the media.

## RESULTS

### 

#### 

##### SILAC Protein Quantification and Significance across Treatment Groups

Following MaxQuant analysis, 3410 protein groups were quantified across the three biological replicates following M1 stimulation. There were 2418, 2510, and 2482 protein groups quantified in the first, second, and third biological replicates, respectively. At the peptide level, 17,433 unique peptides were quantified across the three biological replicates. Of these, 9,067 unique peptides were quantified in the three biological replicates, and 12,788 were found in at least two of the biological replicates. Four hundred and nine of the protein groups were found to be significantly differentially expressed compared with control (*p* < 0.05, SigA test using median value of three replicates), as shown in supplemental Table S1.

M2a stimulation resulted in the quantification of 4224 protein groups across the three biological replicates: 2959 of these protein groups were quantified in all three biological replicates, and 3684 were found in at least two biological replicates. At the peptide level, 25,260 unique peptides were quantified across the three biological replicates. Of these, 8863 peptides were quantified in three biological replicates, and 15,372 were quantified in at least two of the biological replicates. Four hundred and sixty seven protein groups were significantly differentially expressed at a *p* value of <0.05 (SigA test using median value of three replicates), as shown in supplemental Table S2.

Following M2b stimulation, 4062 protein groups were quantified across the three biological replicates. Of these, 2770 were found in all three biological replicates and 3393 were quantified in at least two of the biological samples. At the peptide level, 23,455 unique peptides were found across the three biological replicates, and 8379 were found in all three biological replicates. There were 13,555 unique peptides found in at least two of the biological replicates. Following Perseus analysis, 467 protein groups were found to be significantly up- or down-regulated because of treatment (SigA test using median value of three replicates), as shown in supplemental Table S3.

M2c stimulation resulted in the quantification of 4037 protein groups across the three biological replicates. Of these, 3028 protein groups were quantified in all three biological replicates and 3567 were found in at least two biological replicates. At the peptide level, 21,525 unique peptides were quantified across the three biological replicates, 8379 of which were found in all three biological replicates, and 13,555 were found in at least two biological replicates. Four hundred and sixty one protein groups were found to be significantly up- or down-regulated, as shown in supplemental Table S4. Log_2_ ratio distributions for all four activation stages can be found in supplemental Figs. S1*A*–S1*D*, and mass spectrometric raw data from this study can be found at the Chorus Project, project #888, https://chorusproject.org. Additionally, the standard error of the individual protein ratios can be found in supplemental Table S5 and all peptide and protein level data can be found in supplemental Tables S6 and S7.

##### Ingenuity Pathway Analysis of Significant Proteins

**Fig. 1.**
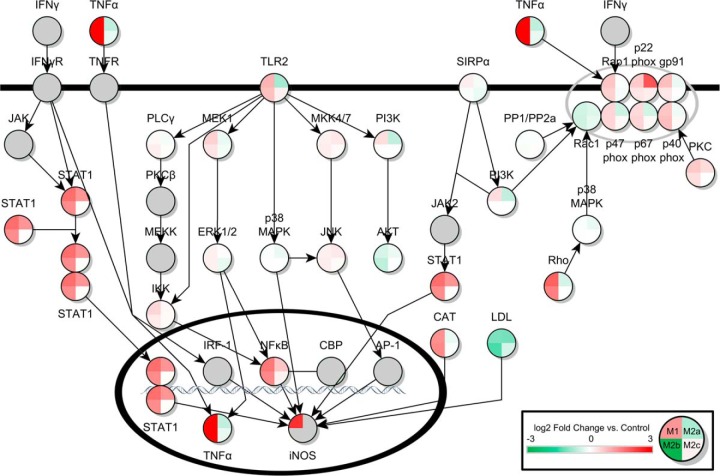
**Production of ROS and RNS in Microglia.** Canonical pathway for the production of ROS and RNS in microglia is shown above. The log_2_ protein expression ratio for all four activation states compared with control are shown in a three color heatmap scale of green-white-red, where green is underexpression, white is unchanged expression, and red is overexpression. Gray quadrants or circles represent protein groups not quantified for that activation state compared with control. M1, M2a, M2c, and M2b are represented from the top left quadrant moving clockwise. Data presented is the median value from across the three biological replicates for each activation state.

**Fig. 2.**
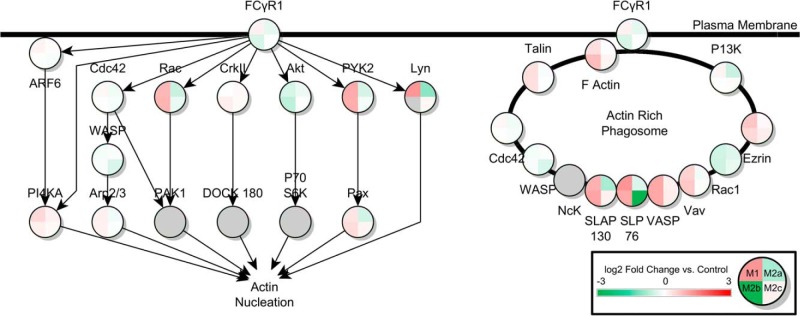
**Fcγ-Receptor Mediated Phagocytosis in Activated Microglia.** Canonical pathway for Fcγ-receptor mediated phagocytosis from Ingenuity Pathway Analysis. Design and statistical analysis is the same as in Fig. 1.

**Fig. 3.**
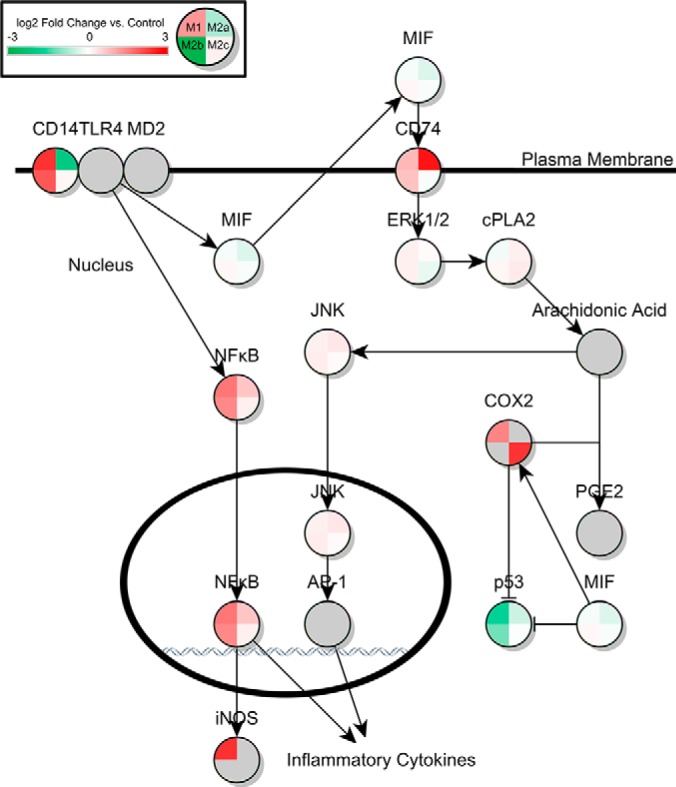
**MIF Regulation of Innate Immunity in Activated Microglia.** Canonical pathway for MIF regulation of innate immunity from Ingenuity Pathway Analysis. Design and statistical analysis is the same as in Fig. 1.

**Fig. 4.**
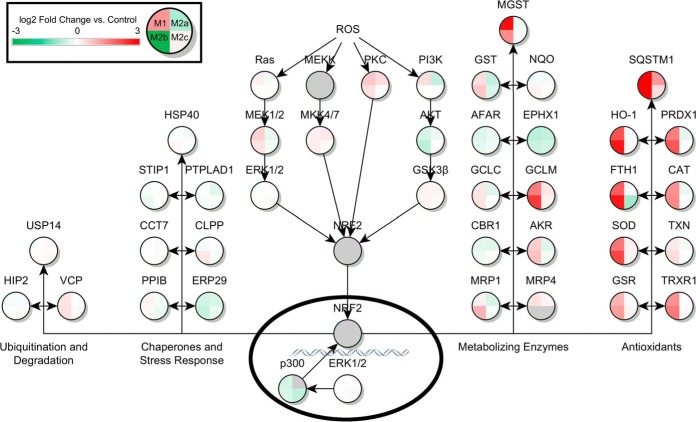
**NFE2L2-Regulated Antioxidant Response in Activated Microglia.** Canonical pathway for NFE2L2-regulated (shown as NRF2) antioxidant response from Ingenuity Pathway Analysis. Design and statistical analysis is the same as in Fig. 1.

Ingenuity Pathway Analysis (IPA) of the significant proteins across the four activation states led to the identification of multiple pathways known to be important to microglial function. In the ROS/RNS pathway controlling ROS release in response to bacterial infection or pro-inflammatory cytokines, 13 proteins were significantly up- or down-regulated in the M1 state in response to LPS stimulation. Thirteen proteins were also found significantly differentially expressed in the M2a state in response to IL-4/IL-13 treatment. In M2b phase, 9 proteins were found to be significantly differentially expressed, and no differentially expressed proteins were found in the M2c activation state. Also significantly perturbed compared with control was the Fcγ Receptor Mediated Phagocytosis Response. Ten, 7, 13, and 4 proteins from the pathway were differentially expressed in M1, M2a, M2b, and M2c phases respectively, as shown in Table II. Additionally, the pathway describing macrophage inhibitory factor regulation of innate immunity was predicted to be altered. Seven, 5, and 6 proteins were altered compared with control in the M1, M2a, and M2b states, respectively. No alternatively expressed proteins were seen in the M2c state. Finally, several proteins were predicted to be significantly altered in the NFE2L2 response to ROS pathway. Thirteen, 7, 17, and 7 proteins were significantly differentially expressed in the M1, M2a, M2b, and M2c states, respectively. Tables of all differentially expressed proteins, and their nondifferentially expressed counterpart ratios in other states, are shown in Table I, Table II, Table III, and Table IV. A heat map of pathways showing the log_2_ protein expression ratios of all proteins found in these pathways across all four activation states is shown in Figs. 1, 2, 3, 4. Additionally, bar charts of select canonical pathways can be found in supplemental Fig. S2.

**Table I:** Significantly altered proteins involved in ROS/RNS production in microglia. Pathway analysis identified the below proteins as significantly differentially expressed compared to control in one or more of the activation stages. All levels are shown across the treatment groups, proteins that were not observed are blank. Significantly differentially expressed (p < *0.05)* proteins shown with asterisk

Gene	Protein name	M1	M2a	M2b	M2c
PPP1R11	Protein phosphatase 1, regulatory (inhibitor) subunit 11	1.24		1.19	1.25*
TLR2	Toll-like receptor 2	1.87*	−1.76*	1.71*	1.14
PIK3R2	Phosphoinositide-3-kinase, regulatory subunit 2 (Beta)	1.10	−1.43*	1.01	1.04
APOE	Apolipoprotein E	−2.16*	−2.23*	−2.64*	−1.31*
CAT	Catalase	2.53*	−1.17	2.65*	−1.03
NFKB1	Nuclear factor of kappa light polypeptide gene enhancer in B-cells 1	1.77*	1.42*	1.84*	−1.03
PRKCD	Protein kinase C, Δ	1.60*	1.35*	1.32	1.01
NOS2	Nitric oxide synthase 2, inducible	5.29*			1.17
NCF4	Neutrophil cytosolic factor 4, 40Kda	1.61*	−1.12	1.73*	1.05
CYBB	Cytochrome B-245, β polypeptide	1.71*	−1.15	1.22	1.01
CYBA	Cytochrome B-245, α polypeptide	1.57*	4.06*	1.25	1.18*
RHOC	Ras homolog family member C	3.67*	−1.32	2.85*	1.01
ATM	Ataxia telangiectasia mutated	1.34	−1.50*		1.02
PPP2R2A	Protein phosphatase 2, regulatory subunit b, α	1.38	1.29*	1.39	1.21*
STAT1	Signal transducer and activator of transcription 1, 91Kda	3.32*	2.44*	2.61*	1.04
RAP1B	Rap1B, member of Ras oncogene family	1.64*	−1.03	1.48	−1.06
NFKB2	Nuclear factor of kappa light polypeptide gene enhancer in B-cells 2 (P49/P100)	3.10*	1.63*	2.66*	1.15

**Table II:** Significantly altered proteins involved in Fcγ-Receptor regulated phagocytosis. Pathway analysis identified the below proteins as significantly differentially expressed compared to control in one or more of the activation stages. All levels are shown across the treatment groups, proteins that were not observed are blank. Significantly differentially expressed (p < *0.05)* proteins shown with asterisk

Gene	Protein name	M1	M2a	M2b	M2c
ACTG1	Actin, γ1	1.33	-1.02	1.75*	1.10
ARPC1A	Actin related protein 2/3 complex, subunit 1A, 41Kda	1.11	−1.38*	−1.01	−1.49*
CBL	Cbl proto-oncogene, e3 ubiquitin protein ligase	1.46	1.44*	1.39*	−1.08
EZR	Ezrin	1.62*	1.07	1.40*	1.17
FYB	Fyn binding protein	1.91*	−1.70*	2.15*	1.16
HMOX1	Heme oxygenase (decycling) 1	4.16*	−1.07	6.41*	1.06
LCP2	Lymphocyte cytosolic protein 2 (Sh2 domain containing leukocyte protein of 76Kda)	2.41*	−1.26	2.06*	−7.47*
LYN	V-Yes-1 Yamaguchi sarcoma viral related oncogene homolog	2.38*	−1.99*		1.08
PIK3R2	Phosphoinositide-3-kinase, regulatory subunit 2 (β)	1.10	−1.43*	1.01	1.04
PLD4	Phospholipase D family, member 4	−1.54	−2.03*	−1.86*	−1.57*
PRKCD	Protein kinase C, Δ	1.60*	1.35	1.32	1.01
PTK2B	Protein tyrosine kinase 2 β	2.05*	−1.31	2.00*	−1.07
RAC2	Ras-related C3 botulinum toxin substrate 2 (Rho family, small Gtp binding protein Rac2)	1.83*	−1.43*	1.86*	−1.14
SYK	Spleen tyrosine kinase	2.65*	−1.42	2.44*	−1.12
VASP	Vasodilator-stimulated phosphoprotein	2.10*	1.14	2.08*	1.17
VAV1	Vav 1 guanine nucleotide exchange factor	1.47	−1.09	1.59*	1.03

**Table III:** Significantly altered proteins involved in MIF regulation of innate immunity. Pathway analysis identified the below proteins as significantly differentially expressed compared to control in one or more of the activation stages. All levels are shown across the treatment groups, proteins that were not observed are blank. Significantly differentially expressed (p < *0.05)* proteins shown with asterisk

Gene	Protein name	M1	M2a	M2b	M2c
CD14	Cd14 molecule	5.27*	-4.21*	3.93*	1.08
CD74	Cd74 molecule, major histocompatibility complex, class Ii invariant chain	1.81*	6.52*	1.67*	−1.04
NFKB1	Nuclear factor of κ light polypeptide gene enhancer in B-cells 1	1.77*	1.42*	1.84*	−1.03
NFKB2	Nuclear factor of κ light polypeptide gene enhancer in B-cells 2 (P49/P100)	3.10*	1.63*	2.66*	1.15
NOS2	Nitric oxide synthase 2, inducible	5.29*			1.17
PTGS2	Prostaglandin-endoperoxide synthase 2 (prostaglandin G/H synthase and cyclooxygenase)	2.75*		5.19*	
TP53	Tumor protein P53	−3.34*	−1.39	−2.26*	−1.02

**Table IV:** Significantly altered proteins involved in NFE2L2 regulated oxidative species response. Pathway analysis identified the below proteins as significantly differentially expressed compared to control in one or more of the activation stages. All levels are shown across the treatment groups, proteins that were not observed are blank. Significantly differentially expressed (p < *0.05)* proteins shown with asterisk

Gene	Protein Name	M1	M2a	M2b	M2c
CAT	Catalase	2.53*	-1.17	2.65*	-1.03
DNAJC5	Dnaj (Hsp40) homolog, subfamily C, member 5	1.70*	−1.19	1.20	1.24*
DNAJC11	Dnaj (Hsp40) homolog, subfamily C, member 11	1.66*	1.07	1.02	1.07
FTH1	Ferritin, heavy polypeptide 1	3.78*	−1.04	6.43*	−1.79*
GCLM	Glutamate-cysteine ligase, modifier subunit	2.98*	1.24	4.93*	1.15
GSR	Glutathione reductase	1.88*	−1.04	2.28*	1.12
HMOX1	Heme oxygenase (decycling) 1	4.16*	−1.07	6.41*	1.06
MGST1	Microsomal glutathione S-transferase 1	6.74*	−1.14	2.57*	1.07
PRDX1	Peroxiredoxin 1	2.94*	−1.20	4.04*	1.00
PRKCD	Protein kinase C, Δ	1.60*	1.35*	1.32	1.01
SOD2	Superoxide dismutase 2, mitochondrial	3.14*	1.16	4.59*	1.10
SQSTM1	Sequestosome 1	9.44*	2.35*	11.40*	1.34*
TXNRD1	Thioredoxin reductase 1	2.53*	−1.01	3.10*	1.05

The various treatments also significantly changed the biological function assignments by IPA. M1 and M2b microglia demonstrated increases in overall protein expression levels for proteins related to cell movement of phagocytes, phagocytosis by macrophages, immune response of cells, cell viability, and accumulation of lipids. The M1 and M2b states also led to decreases in proteins related to organismal death. In comparison, M2a state microglia demonstrated decreased levels of proteins relating to cell viability compared with quiescent microglia. Decreases in proteins related to inflammation of tissue were also observed in the M2a state microglia. M2c microglia demonstrate lower levels of immune response of cells compared with the resting state, as shown in Table V.

**Table V:** Significantly altered biological functions across all activation states. Ingenuity assigned z-scores for approximation of increased biological function as a log_2_ expression for all activation states. Only biological functions that Ingenuity has found to be statistically significant (p < *0.05)* have been included

Biological Function	M1	M2a	M2b	M2c
Accumulation of lipid	1.83		1.55	2.47
Cell movement of phagocytes	3.98	0.66	3.04	
Cell viability	2.26	−0.93	4.20	
Immune response of cells	2.00	−0.28	2.05	−1.45
Immune response of T lymphocytes	2.43	1.98	2.41	0.48
Organismal death	−2.71	−1.36	−2.57	0.82
Phagocytosis by macrophages	2.44	−0.51	1.40	
Toxicity of cells	2.99	−0.12	2.20	

##### Validation of Novel Activation Markers Determined by Proteomics

**Fig. 5.**
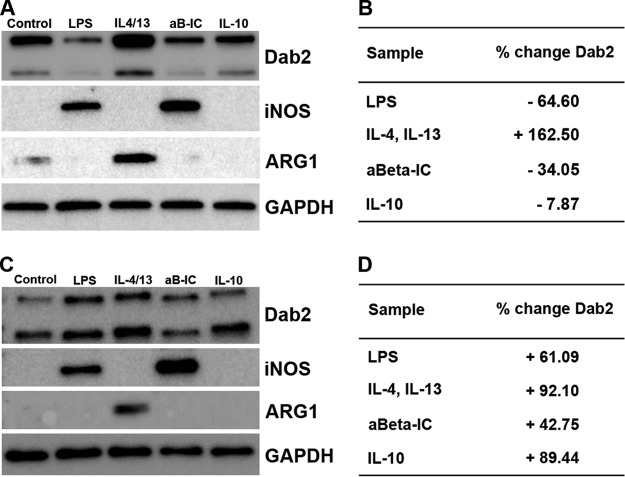
**Western analysis of Novel Activation Marker, DAB2, in Mouse Microglia.** Western blot shows DAB2 levels in *A*. BV2 mouse microglia and *C,* primary mouse microglia after treatment with control, LPS, IL-4/IL-13, aBeta-immune complex (aB-IC), or IL-10 (top row). Second and third rows show iNOS and ARG1, respectively, to verify M1, M2a, and M2b simulation. GAPDH was used to normalize the loading across samples (bottom row). Table shows the normalized relative abundance of DAB2 in *B*. BV2 mouse microglia and *D,* primary mouse microglia after various treatments as compared with the control sample. GAPDH levels were used to normalize protein abundance in each sample.

Western blotting analysis for classical markers of M1 phase and M2a phase activation was used to confirm successful stimulation of the respective microglial activation states. Across three biological replicates for each activation state and control, production of iNOS protein was observed for M1 and M2b activation states, as shown in Fig. 5. No iNOS signal was detected in the control, M2a, or M2c states of activation. ARG1 production was only present in M2a phase stimulation, as shown in Fig. 5.

A possible candidate as a novel activation marker was identified using the differential protein expression data and confirmed via Western blotting. Disabled homolog 2, DAB2, is a regulator of clathrin-mediated endocytosis. Western blot confirmed differences in protein abundance levels between resting microglia and activation states in BV2 cells, as shown in Fig. 5*A*. The relative DAB2 protein levels were increased by 162.5% in M2a phase, and decreased by 64.60%, 34.05%, and 7.87% in M1, M2b, and M2c phases, respectively (Fig. 5*B*).

Extending the analysis of DAB2 to primary mouse microglia revealed that DAB2 levels were altered between quiescent and stimulated microglia. Specifically, 61.1%, 92.1%, 42.8%, and 89.4% more DAB2 protein was found in M1, M2a, M2b, and M2c phases, respectively, compared with the control treatment (Fig. 5*C*, 5*D*). Re-probing the Western blot with iNOS, which produced a band of the expected size in the LPS and Aβ-IgG samples, bolstered confidence that the M1 and M2b phases were successfully stimulated. Likewise, the presence of an ARG1 band in only the IL-4- and IL-13-stimulated sample suggested successful simulation of M2a phase.

##### Measurement of microglial secreted cytokines

**Fig. 6.**
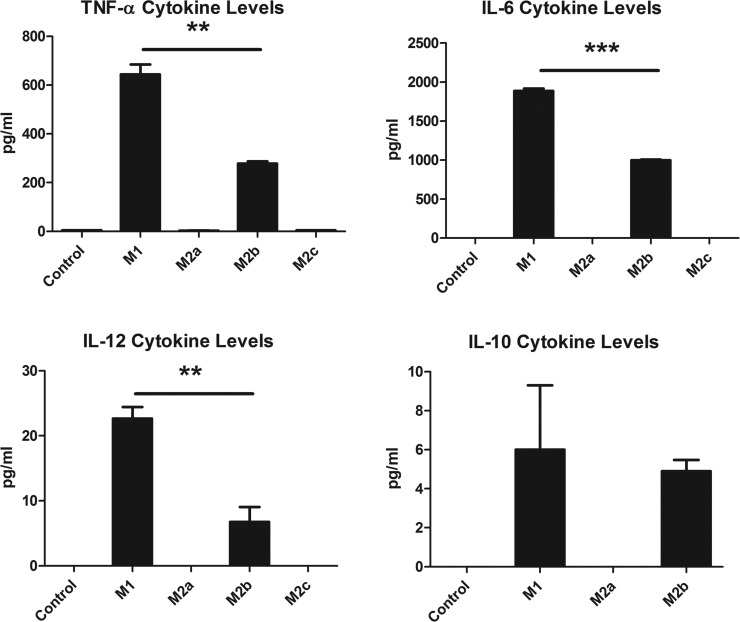
**Cytokine Secretion from Select Microglia.** Median fluorescent intensity readings were normalized to pg/ml from a standard curve for each cytokine each measured in triplicate. Error bars represent S.E. **, *p* < 0.01; ***, *p* < 0.0001. Difference between M1 and M2b production of IL-10 was not statistically significant.

Multiplexing analysis (Luminex) was used to measure cytokine secretion from both the activated and resting state. Secreted levels of the inflammatory cytokines TNFα, IL-6, and IL-12, as well as the M2c phase trigger IL-10 were examined and revealed significantly increased levels of all four cytokines in the M1 and M2b stages. No significant cytokine expression differences compared with resting state were observed for M2a or M2c states (Fig. 6).

## DISCUSSION

Previous studies have examined the proteome and transcriptome of the BV2 microglia cell line (32, 2, 33, 34). The untreated BV2 cells in this study showed continuity with these studies, identifying several key microglia markers, such as Macrosialin, CD18, CD11b, and F4/80 antigen [36], as well as proteins that mediate microglia function, such as MIF (36), CTSB (37), ILF2, NFκB1, and NFκB2 (35, 38, 39), in a similar abundance. As seen in other studies of BV2 cells, KEGG pathway analysis identified several proteins that are linked to neurological disorders, such as Alzheimer's disease (102 proteins), Huntington's disease (109 proteins), and Parkinson's disease (93 proteins). Several previously reported proteins involved in microglial function were also identified in this study, including ubiquitin-mediated proteolysis (59 proteins) (40), FcγR mediated phagocytosis (46 proteins) (41), neurotrophin signaling (47 proteins) (35), and SNARE interactions in vesicular transport (17 proteins) (35). Additionally, the PANTHER pathway database was used to identify signaling pathways that have been previously linked to microglial function (34), including PDGF signaling (36 proteins), Toll receptor signaling proteins (46 proteins), EGF receptor signaling pathway (28 proteins), FGF receptor signaling (26 proteins), VEGF receptor signaling (25 proteins), integrin signaling (46 proteins), and chemokine- and cytokine-mediated inflammation (58 proteins). These pathways have been linked to several neurological disorders (35, 42, 38), thereby underscoring the clinical relevance of the BV2 cell line, and also providing a basis for future studies that probe the downstream consequences of insult, injury, and also treatment using this model.

The Luminex data confirmed the successful activation of the inflammatory states M1 and M2b, which include increases in the inflammatory cytokines TNFα, IL-6, and IL-12. Interestingly, IL-10 was only produced by the microglia in the M1 and M2b inflammatory stages, but not in the M2a microglia as expected, despite being assigned originally to the Th2 family of neurotrophic microglia. These data suggest that 24 h after initial exposure to the activator, the cells are activating IL-10 in order to “switch off” the inflammatory process. Additional confirmation of successful activation of the various activation states is found in the Western blot data for the classical activation markers iNOS and ARG1. As expected, robust iNOS expression is observed in M1 and M2b state; it was not detected in the resting, M2a, or M2c states of activation, as expected. The enzyme that competes with iNOS for the use of the arginine pool, ARG1, was significantly up-regulated in the M2a state, as expected.

The biological function prediction data produced through IPA aligned with expected pro- or anti-inflammatory characteristics of the activation state. Bioinformatic analysis predicted M1 and M2b states to exhibit high levels of immune response functions relating to toxicity, phagocytosis, motility and overall viability. M2a was predicted to display levels unchanged from resting, or decreased in the case of cell viability. M2c state was predicted to show a significant decrease in the immune response of cells, as expected for a state that acts as a clamp on pro-inflammatory immune response.

The canonical pathway data provided valuable insights into the possible regulation and action of various canonical pathways of microglial activity. In the ROS/RNS pathway, all members of the NADPH oxidase complex were quantified in all of the activation states. As expected, the constituents of the NADPH oxidase complex were up-regulated in M1 and M2b phases, which both have pro-inflammatory characteristics. In addition, iNOS was only up-regulated in M1 phase, despite being detected in M2b phase by Western blot analysis. However, using a lower stringency applied to the mass spectrometric data, a SILAC pair was identified for iNOS in the M2b data set that indicated up-regulation (data not shown). In the M2a state, the toll-like receptor 2 protein, which is crucial for the NFκB-regulated inflammatory response to lipid-containing species is predicted to be significantly down-regulated when compared with resting state. In addition, CD14, the co-activator of the toll like receptor 4/MD2 complex, which responds to lipopolysaccharide, was predicted to be highly down-regulated in M2a, suggesting control of entry into the NFκB pathway of iNOS production is important in the M2a response.

Interestingly, several regulators of inflammatory response were found to be up-regulated in the mixed inflammatory state, M2b. Signal Transducer and Activator of Transcription 1, STAT1, NFκB1, and NFκB2 were up-regulated in M1 and M2b, but also less significantly up-regulated in M2a. Also, the negative regulator low-density lipoprotein (LDL) was predicted to be down-regulated in the inflammatory states M1 and M2b, but also down-regulated in M2a. The data suggest that overall levels of these important universal regulatory factors may be important to all activation states, and perhaps additional post-translational modifications are required to differentiate between a pro-inflammatory and anti-inflammatory response. For example, NFκB2 acetylation by the histone acetyltransferase p300 has been implicated in its pro-inflammatory regulatory activity, and STAT1 requires phosphorylation before dimerization and relocalization to the nucleus to transcribe inflammatory cytokines and agents as part of the JAK/STAT pathway (33, 34).

In the phagocytic pathway, M1 and M2b states are predicted to display strong up-regulation of regulators of the actin nucleation process, as well as bound proteins found in the actin-rich phagosome that is formed at the Fcγ-receptor. Of particular interest is the M2b state down-regulation of LYN, an SRC kinase involved not only in the process of actin nucleation as part of phagocytosis, but also in the release of pro-inflammatory cytokines by macrophages, suggesting a similar role in microglia (35). Also of note is the strong down-regulation in the M2c state of Src homology 2 domain-containing leukocyte-specific protein of 76 kDa, SLP-76. In T-cells, deletion of SLP-76 has been shown to abrogate antigen sensing and phagocytic activity of immature immune cells that have not yet been exposed to antigens (36, 37). It is possible that a similar response is possible in M2c phase microglia, disabling their ability to respond to antigens, and possibly the production of inflammatory cytokines as well.

IPA predicts that the MIF-related regulation of immunity pathway would show several points of regulation of the inflammatory processes. As expected, cyclooxygenase-2, COX-2, an enzyme associated with the inflammatory response (38), is increased in the M1 and M2b phases. Intriguingly, p53, which has been shown to be negatively regulated by COX-2 (39), is also significantly down-regulated in both M1 and M2b phases. Because p53 regulates apoptosis, lower levels of p53 could allow microglia to thrive in the inflammatory environment that the microglia are creating in an effort to fight infection (40). A small but significant down-regulation of p53 in M2a phase was observed; perhaps also in order to allow the microglia to perform their neurotrophic duties in a highly inflammatory and damaging environment. Major histocompatibility complex II, CD74, is up-regulated in both M1 and M2b states, suggesting a possible link to COX-2 and related p53 functional regulation through MIF (41). Of interest is that CD74 showed an even greater up-regulation in the M2a state, despite no downstream up-regulation of COX-2. Future studies could explore how the activity of CD74 is modulated in microglia depending on whether it has bound MIF and the related effects on microglial activation phenotype.

The NFE2L2 pathway includes extensive production of a variety of antioxidant proteins that are predicted to be up-regulated in the M1 and M2b inflammatory states. In addition, several metabolizing enzymes are predicted to be up-regulated in these states, such as microsomal glutathione-S-transferase and glutamine-cysteine ligase, the rate-limiting enzyme of the glutathione synthesis cascade. The M2a state shows little up-regulation of NFE2L2 -related ROS response genes, which is not surprising because of the possible lack of an ROS trigger for the multiple cascades that lead to NFE2L2 nuclear translocation. Sequestosome, an antioxidant protein, is up-regulated in M1 and M2b, as expected, but also up-regulated in M2a. Sequestosome has been shown in macrophages to inhibit the action of MyD88, a member of the cascade downstream of the TLR4/CD14/MD2 complex that is bound by LPS during M1 activation (42). As mentioned earlier, many of the unexpectedly up-regulated M2a proteins in these M1/M2b related pathways have dual functions; to carry out the inflammatory response in M1 and M2b phases, and to help block that same response in M2a (43).

The utility of mass spectrometry-based proteomics was demonstrated through the identification of a novel activation marker for microglia that is able to differentiate between the M2a and either the M1 or M2b activation states. Additionally, this study shows that the increased abundance of DAB2 in the M2a phase, for example, is not unique to the BV2 cells line, but is also seen in primary mouse microglia. DAB2 is a key regulator of the clathrin-mediated endocytosis pathway, which is predicted to be down-regulated during M1 and M2a states because of phagocytic action as a means of engulfment. Another protein involved in endocytosis, mannose receptor-1, MRC1, is often used as a marker of M2a state activation along with ARG1 and the cytokine YM1. MRC1 was also found to be up-regulated in the M2a data set. Other work, however, has suggested that DAB2 has a far greater role in the inflammatory response. For example, DAB2 has been shown to stabilize the anti-inflammatory tumor growth factor-β receptor (44). Future studies are needed to determine the mechanistic detail regarding the possible role of DAB2 in microglial function.

Proteomic analysis coupled with validation via cytokine assay and Western blotting of the various states of microglial activation yielded novel insights into the differences and similarities of these states. The level of coverage for the canonical pathways offers further evidence that BV2 cells can act as an appropriate stand-in for primary microglia, with none of the difficulties of limited quantity or difficult manipulation and isolation. The proteomic analysis implied that M2a anti- inflammatory action may act not only through the action of the classical ARG1, but also by blocking the inflammatory activities of the M1 and M2b states, particularly through differential protein expression of CD74, LYN, SQST1, TLR2, and CD14. The differential expression of these proteins is able to promote healing, limit phagocytosis, and limit activation of RNS through toll like receptor cascades. M2c anti-inflammatory action appears to center around the down-regulation of a key member in the formation of actin-rich phagosomes, SLP-76. Finally, the proteomic analysis was able to identify DAB2 as a potential candidate that can differentiate microglia activation states. Further work needs to explore microglial activation dynamics by measuring differential protein expression at multiple time points in order to better understand the phenotypic changes induced over time by various pro- and anti-inflammatory drivers. Knowledge of these differences would prove insightful to microglia switching in times of injury and insult.

## Supplementary Material

Supplemental Data

## Footnotes

^1^ The abbreviations used are:

ROS: reactive oxygen species
DAB2: disabled homolog 2
DMEM: Dulbecco's modification of Eagles medium
FASP: filter-aided sample preparation
IL-6: interleukin-6
IFNγ: interferon γ
iNOS: inducible nitric oxide synthase
SILAC: stable isotope labeling with amino acids in cell culture
STAT1: signal transducer and activator of transcription 1
TLR2: toll-like receptor 2
TNF-α: tumor necrosis factor α.

## References

[1] Block M. L., Zecca L., and Hong J. S. (2007) Microglia-mediated neurotoxicity: uncovering the molecular mechanisms. Nat. Rev. Neurosci. 8, 57–69 17180163 10.1038/nrn2038

[2] Colton C. A. (2009) Heterogeneity of microglial activation in the innate immune response in the brain. J. Neuroimmune Pharmacol. 4, 399–418 19655259 10.1007/s11481-009-9164-4 PMC2773116

[3] Block M. L., and Hong J. S. (2005) Microglia and inflammation-mediated neurodegeneration: multiple triggers with a common mechanism. Prog. Neurobiol. 76, 77–98 16081203 10.1016/j.pneurobio.2005.06.004

[4] Banati R. B., Gehrmann J., Schubert P., and Kreutzberg G. W. (1993) Cytotoxicity of microglia. Glia 7, 111–118 8423058 10.1002/glia.440070117

[5] Colton C., and Wilcock D. M. (2010) Assessing activation states in microglia. CNS Neurol. Disord. Drug Targets 9, 174–191 20205642 10.2174/187152710791012053

[6] de Vries H. E., Witte M., Hondius D., Rozemuller A. J., Drukarch B., Hoozemans J., and van Horssen J. (2008) Nrf2-induced antioxidant protection: a promising target to counteract ROS-mediated damage in neurodegenerative disease? Free Radical Biol. Med. 45, 1375–1383 18824091 10.1016/j.freeradbiomed.2008.09.001

[7] Colton C. A., Mott R. T., Sharpe H., Xu Q., Van Nostrand W. E., and Vitek M. P. (2006) Expression profiles for macrophage alternative activation genes in AD and in mouse models of AD. J. Neuroinflammation 3, 27 17005052 10.1186/1742-2094-3-27 PMC1609108

[8] Liu B. (2006) Modulation of microglial pro-inflammatory and neurotoxic activity for the treatment of Parkinson's disease. AAPS J. 8, E606–621 17025278 10.1208/aapsj080369 PMC2668934

[9] Liu B., Gao H. M., and Hong J. S. (2003) Parkinson's disease and exposure to infectious agents and pesticides and the occurrence of brain injuries: role of neuroinflammation. Environ. Health Perspect. 111, 1065–1073 12826478 10.1289/ehp.6361 PMC1241555

[10] Crews F. T., Bechara R., Brown L. A., Guidot D. M., Mandrekar P., Oak S., Qin L., Szabo G., Wheeler M., and Zou J. (2006) Cytokines and alcohol. Alcohol Clin. Exp. Res. 30, 720–730 16573591 10.1111/j.1530-0277.2006.00084.x

[11] He J., and Crews F. T. (2008) Increased MCP-1 and microglia in various regions of the human alcoholic brain. Exp. Neurol. 210, 349–358 18190912 10.1016/j.expneurol.2007.11.017 PMC2346541

[12] Qin L., and Crews F. T. (2012) NADPH oxidase and reactive oxygen species contribute to alcohol-induced microglial activation and neurodegeneration. J. Neuroinflammation 9, 5 22240163 10.1186/1742-2094-9-5 PMC3271961

[13] Crews F., Nixon K., Kim D., Joseph J., Shukitt-Hale B., Qin L., and Zou J. (2006) BHT blocks NF-kappaB activation and ethanol-induced brain damage. Alcohol Clin. Exp. Res. 30, 1938–1949 17067360 10.1111/j.1530-0277.2006.00239.x

[14] Raivich G., Bohatschek M., Kloss C. U., Werner A., Jones L. L., and Kreutzberg G. W. (1999) Neuroglial activation repertoire in the injured brain: graded response, molecular mechanisms and cues to physiological function. Brain Res. Brain Res. Rev. 30, 77–105 10407127 10.1016/s0165-0173(99)00007-7

[15] Graeber M. B., and Streit W. J. (2010) Microglia: biology and pathology. Acta Neuropathol. 119, 89–105 20012873 10.1007/s00401-009-0622-0

[16] Kreutzberg G. W. (1996) Microglia: a sensor for pathological events in the CNS. Trends Neurosci. 19, 312–318 8843599 10.1016/0166-2236(96)10049-7

[17] Wilcock D. M. (2012) A changing perspective on the role of neuroinflammation in Alzheimer's disease. Int. J. Alzheimers Dis. 2012, 495243 22844636 10.1155/2012/495243 PMC3403314

[18] Xu L., Hilliard B., Carmody R. J., Tsabary G., Shin H., Christianson D. W., and Chen Y. H. (2003) Arginase and autoimmune inflammation in the central nervous system. Immunology 110, 141–148 12941151 10.1046/j.1365-2567.2003.01713.x PMC1783013

[19] Mosser D. M. (2003) The many faces of macrophage activation. J. Leukoc Biol. 73, 209–212 12554797 10.1189/jlb.0602325

[20] Mao H., Fang X., Floyd K. M., Polcz J. E., Zhang P., and Liu B. (2007) Induction of microglial reactive oxygen species production by the organochlorinated pesticide dieldrin. Brain Res. 1186, 267–274 17999924 10.1016/j.brainres.2007.10.020

[21] Liu B., Barber D. S., and Stevens S. M. Jr. (2012) Stable isotope labeling with amino acids in cell culture-based proteomic analysis of ethanol-induced protein expression profiles in microglia. Methods Mol. Biol. 829, 551–565 22231838 10.1007/978-1-61779-458-2_35

[22] Das P., Howard V., Loosbrock N., Dickson D., Murphy M. P., and Golde T. E. (2003) Amyloid-beta immunization effectively reduces amyloid deposition in FcRgamma-/- knock-out mice. J. Neurosci. 23, 8532–8538 13679422 10.1523/JNEUROSCI.23-24-08532.2003 PMC6740360

[23] Liu B., Wang K., Gao H. M., Mandavilli B., Wang J. Y., and Hong J. S. (2001) Molecular consequences of activated microglia in the brain: overactivation induces apoptosis. Journal of neurochemistry 77, 182–189 11279274 10.1046/j.1471-4159.2001.t01-1-00216.x

[24] Wisniewski J. R., Zougman A., Nagaraj N., and Mann M. (2009) Universal sample preparation method for proteome analysis. Nat. Methods 6, 359–362 19377485 10.1038/nmeth.1322

[25] Manza L. L., Stamer S. L., Ham A. J., Codreanu S. G., and Liebler D. C. (2005) Sample preparation and digestion for proteomic analyses using spin filters. Proteomics 5, 1742–1745 15761957 10.1002/pmic.200401063

[26] Bell-Temin H., Zhang P., Chaput D., King M. A., You M., Liu B., and Stevens S. M. Jr. (2013) Quantitative proteomic characterization of ethanol-responsive pathways in rat microglial cells. J. Proteome Res. 12, 2067–2077 23495833 10.1021/pr301038f

[27] Bell-Temin H., Barber D. S., Zhang P., Liu B., and Stevens S. M. Jr. (2012) Proteomic analysis of rat microglia establishes a high-confidence reference data set of over 3000 proteins. Proteomics 12, 246–250 22121004 10.1002/pmic.201100398

[28] Cox J., Neuhauser N., Michalski A., Scheltema R. A., Olsen J. V., and Mann M. (2011) Andromeda: a peptide search engine integrated into the MaxQuant environment. J. Proteome Res. 10, 1794–1805 21254760 10.1021/pr101065j

[29] Cox J., Matic I., Hilger M., Nagaraj N., Selbach M., Olsen J. V., and Mann M. (2009) A practical guide to the MaxQuant computational platform for SILAC-based quantitative proteomics. Nat. Protocols 4, 698–705 19373234 10.1038/nprot.2009.36

[30] Cox J., and Mann M. (2008) MaxQuant enables high peptide identification rates, individualized p.p.b.-range mass accuracies and proteome-wide protein quantification. Nat. Biotechnol. 26, 1367–1372 19029910 10.1038/nbt.1511

[31] Elias J. E., and Gygi S. P. (2007) Target-decoy search strategy for increased confidence in large-scale protein identifications by mass spectrometry. Nat. Methods 4, 207–214 17327847 10.1038/nmeth1019

[32] Jimenez-Marin A., Collado-Romero M., Ramirez-Boo M., Arce C., and Garrido J. J. (2009) Biological pathway analysis by ArrayUnlock and Ingenuity Pathway Analysis. BMC Proc. 3, S6 19615119 10.1186/1753-6561-3-S4-S6 PMC2712749

[33] Greene W. C., and Chen L. F. (2004) Regulation of NF-kappaB action by reversible acetylation. Novartis Foundation Symp. 259, 208–217; discussion 218–225 15171256

[34] Kramer O. H., Knauer S. K., Greiner G., Jandt E., Reichardt S., Guhrs K. H., Stauber R. H., Bohmer F. D., and Heinzel T. (2009) A phosphorylation-acetylation switch regulates STAT1 signaling. Genes Develop. 23, 223–235 19171783 10.1101/gad.479209 PMC2648535

[35] Akimoto N., Ifuku M., Mori Y., and Noda M. (2013) Effects of chemokine (C-C motif) ligand 1 on microglial function. Biochem. Biophys. Res. Commun. 436, 455–461 23747724 10.1016/j.bbrc.2013.05.126

[36] Wu G. F., Corbo E., Schmidt M., Smith-Garvin J. E., Riese M. J., Jordan M. S., Laufer T. M., Brown E. J., and Maltzman J. S. (2011) Conditional deletion of SLP-76 in mature T cells abrogates peripheral immune responses. Eur. J. Immunol. 41, 2064–2073 21469089 10.1002/eji.201040809 PMC3124603

[37] Maltzman J. S., Kovoor L., Clements J. L., and Koretzky G. A. (2005) Conditional deletion reveals a cell-autonomous requirement of SLP-76 for thymocyte selection. J. Exp. Med. 202, 893–900 16186188 10.1084/jem.20051128 PMC2213170

[38] Minghetti L. (2004) Cyclooxygenase-2 (COX-2) in inflammatory and degenerative brain diseases. J. Neuropathol Exp. Neurol. 63, 901–910 15453089 10.1093/jnen/63.9.901

[39] Choi E. M., Heo J. I., Oh J. Y., Kim Y. M., Ha K. S., Kim J. I., and Han J. A. (2005) COX-2 regulates p53 activity and inhibits DNA damage-induced apoptosis. Biochem. Biophys. Res. Commun. 328, 1107–1112 15707991 10.1016/j.bbrc.2005.01.072

[40] Mitchell R. A., Liao H., Chesney J., Fingerle-Rowson G., Baugh J., David J., and Bucala R. (2002) Macrophage migration inhibitory factor (MIF) sustains macrophage proinflammatory function by inhibiting p53: regulatory role in the innate immune response. Proc. Natl. Acad. Sci. U.S.A. 99, 345–350 11756671 10.1073/pnas.012511599 PMC117563

[41] Conroy H., Mawhinney L., and Donnelly S. C. (2010) Inflammation and cancer: macrophage migration inhibitory factor (MIF)–the potential missing link. QJM 103, 831–836 20805118 10.1093/qjmed/hcq148 PMC2955282

[42] Into T., Inomata M., Niida S., Murakami Y., and Shibata K. (2010) Regulation of MyD88 aggregation and the MyD88-dependent signaling pathway by sequestosome 1 and histone deacetylase 6. J. Biol. Chem. 285, 35759–35769 20837465 10.1074/jbc.M110.126904 PMC2975200

[43] Lawrence T. (2009) The nuclear factor NF-kappaB pathway in inflammation. Cold Spring Harbor Perspectives Biol. 1, a001651 10.1101/cshperspect.a001651 PMC2882124 20457564

[44] Shapira K. E., Hirschhorn T., Barzilay L., Smorodinsky N. I., Henis Y. I., and Ehrlich M. (2014) Dab2 inhibits the cholesterol-dependent activation of JNK by TGF-beta. Mol. Biol. Cell 25, 1620–1628 24648493 10.1091/mbc.E13-09-0537 PMC4019493

