# Supplementary material for: Novel Molecular Insights into Classical and Alternative Activation States of Microglia as Revealed by Stable Isotope Labeling by Amino Acids in Cell Culture (SILAC)-based Proteomics
Source: Mol Cell Proteomics. 2015 Sep 30;14(12):3173–84. doi: 10.1074/mcp.M115.053926 (PMC4762627; doi:10.1074/mcp.M115.053926)
Supplement: Supplemental Data [file supp_14_12_3173__index.html]

Novel molecular insights into classical and alternative activation states of microglia as revealed by SILAC-based proteomics — Novel Molecular Insights into Classical and Alternative Activation States of Microglia as Revealed by Stable Isotope Labeling by Amino Acids in Cell Culture (SILAC)-based Proteomics — Proteomic Analysis of Microglial Activation — Supplemental Data 

# Novel Molecular Insights into Classical and Alternative Activation States of Microglia as Revealed by Stable Isotope Labeling by Amino Acids in Cell Culture (SILAC)-based Proteomics

## Supplemental Data

- Single peptide MS/MS spectra (.pdf, 4.5 MB) - Annotated MS/MS spectra for single peptide-based protein identifications
- Supplemental Figures 1 and 2, Tables 1-4 (.docx, 457 KB) - Protein ratio distributions, pathway analysis results, and list of differentially expressed proteins for M1, M2a, M2b and M2c SILAC datasets
- Supplemental Tables 5-7 (.xlsx, 33.1 MB) - Protein quantitation for individuals replicates with reported SEM, all peptide, and all protein (PEP values, % coverage) data
