# Supplementary material for: Novel Molecular Insights into Classical and Alternative Activation States of Microglia as Revealed by Stable Isotope Labeling by Amino Acids in Cell Culture (SILAC)-based Proteomics
Source: Mol Cell Proteomics. 2015 Sep 30;14(12):3173–84. doi: 10.1074/mcp.M115.053926 (PMC4762627; doi:10.1074/mcp.M115.053926)

Raw file	Scan	Method	Score	m/z	Gene names
HBT_20130916_BV2_IL43_06	5346	ITMS; CID	103.91	697.37	Pithd1

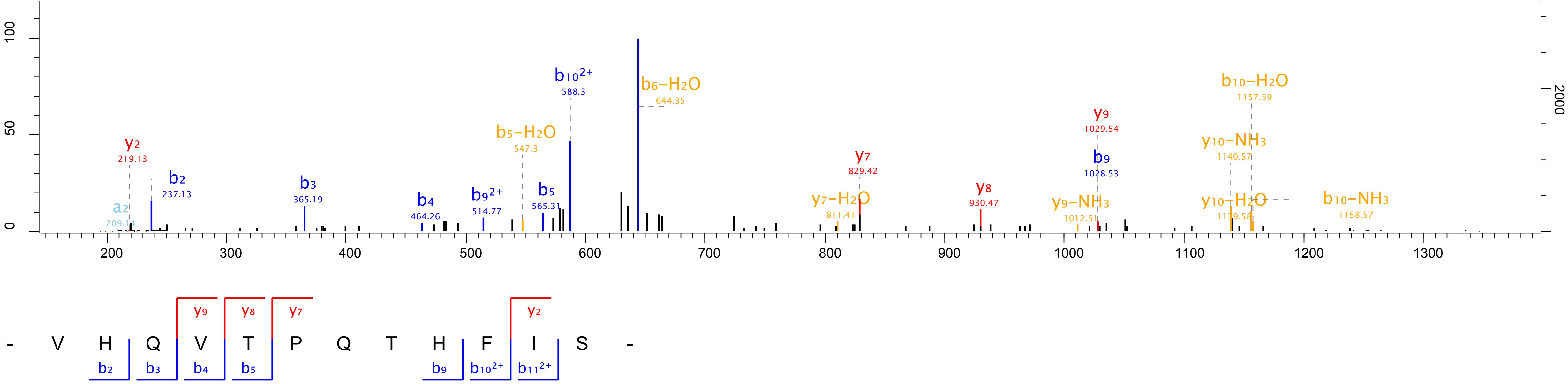

Raw file	Scan	Method	Score	m/z	Gene names
HBT_20130916_BV2_IL43_06	3367	ITMS; CID	112.83	633.34	Pop7

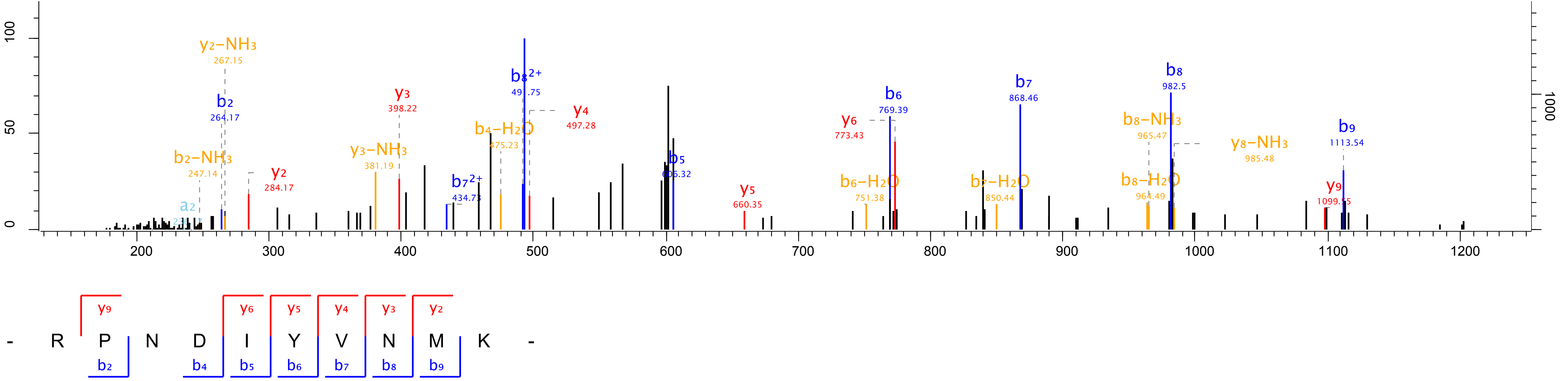

Raw file	Scan	Method	Score	m/z	Gene names
HBT_20130916_BV2_IL43_06	17851	ITMS; CID	73.93	570.35	Nfkbib

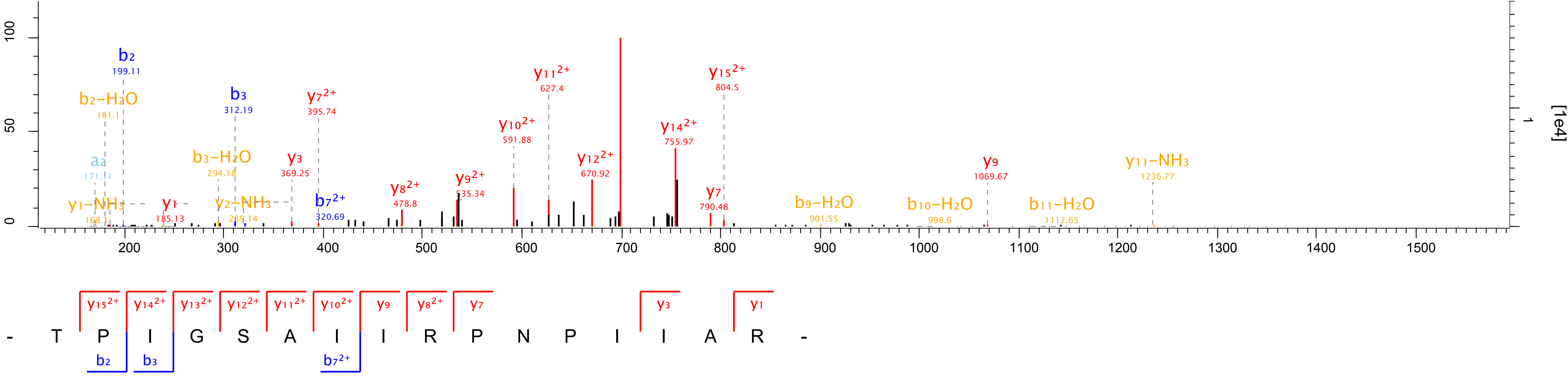

Raw file	Scan	Method	Score	m/z	Gene names
HBT_20130916_BV2_IL43_06	17477	ITMS; CID	73.9	460.6	Flot2

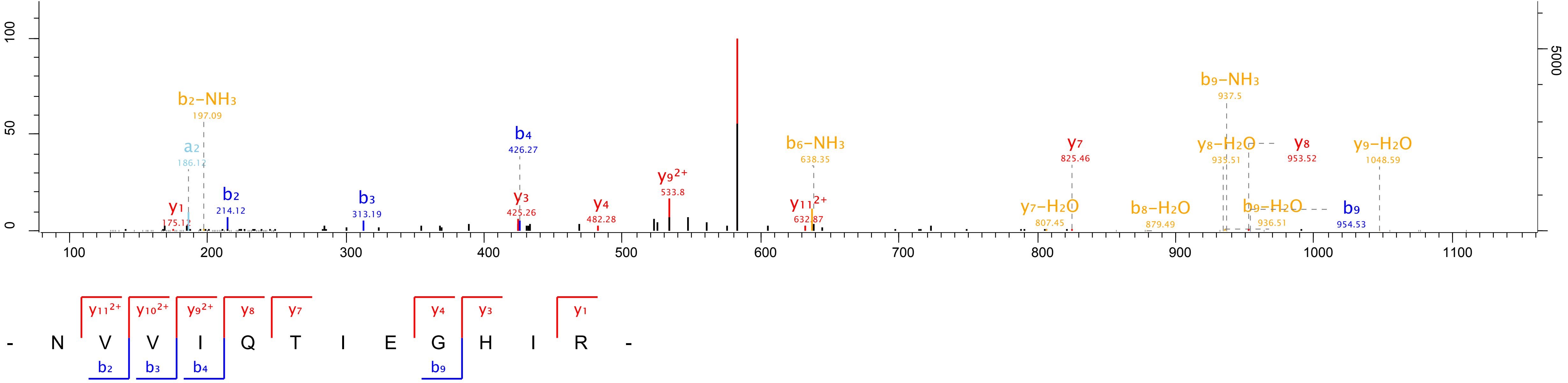

Raw file	Scan	Method	Score	m/z	Gene names
HBT_20130916_BV2_IL43_06	17336	ITMS; CID	73.5	433.26	Ccbl2

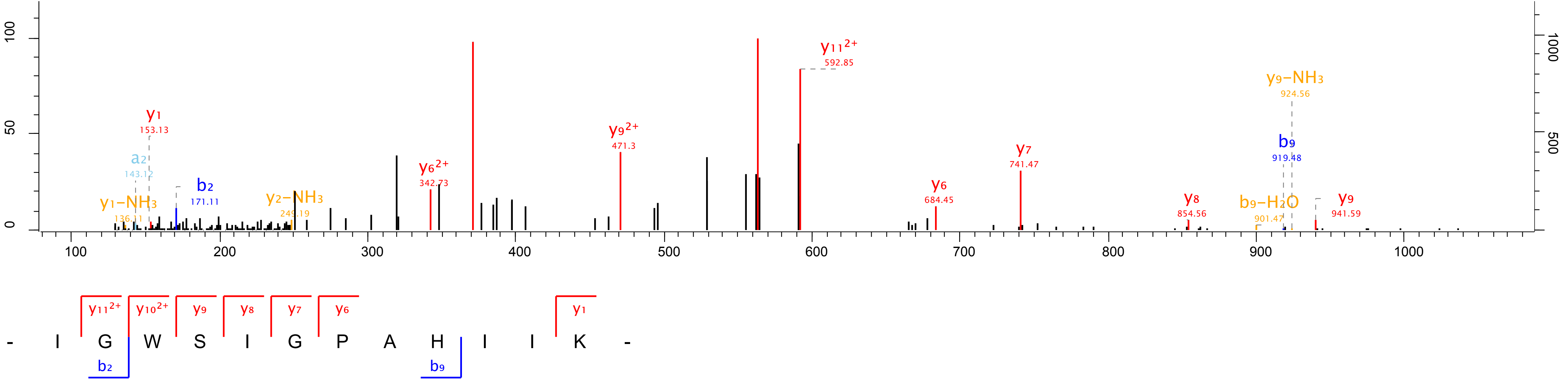

Raw file	Scan	Method	Score	m/z
HBT_20130916_BV2_IL43_06	16049	ITMS; CID	97.47	688.33

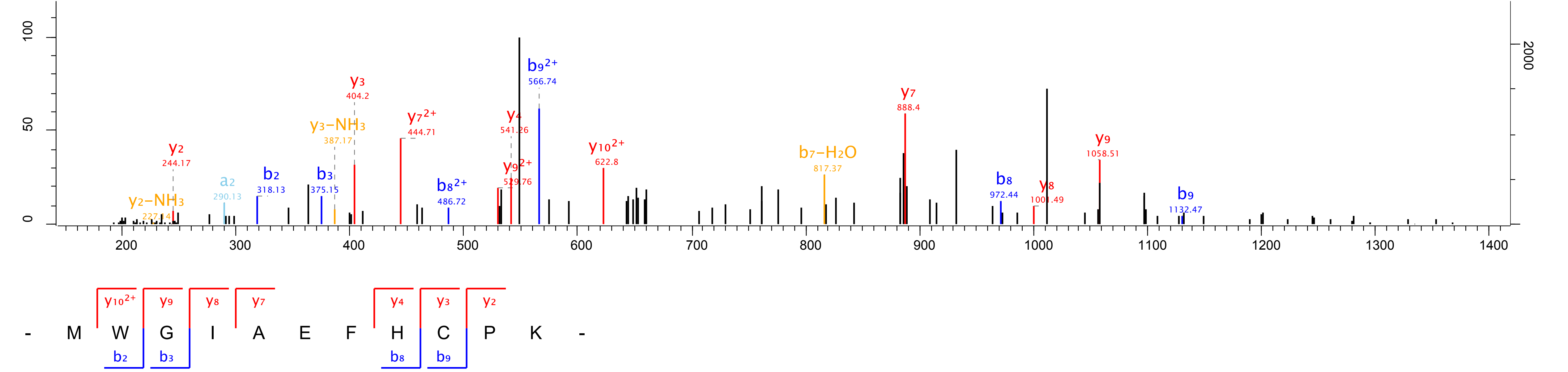

Raw file	Scan	Method	Score	m/z	Gene names
HBT_20130916_BV2_IL43_05	31725	ITMS; CID	104.38	933.81	Gins2

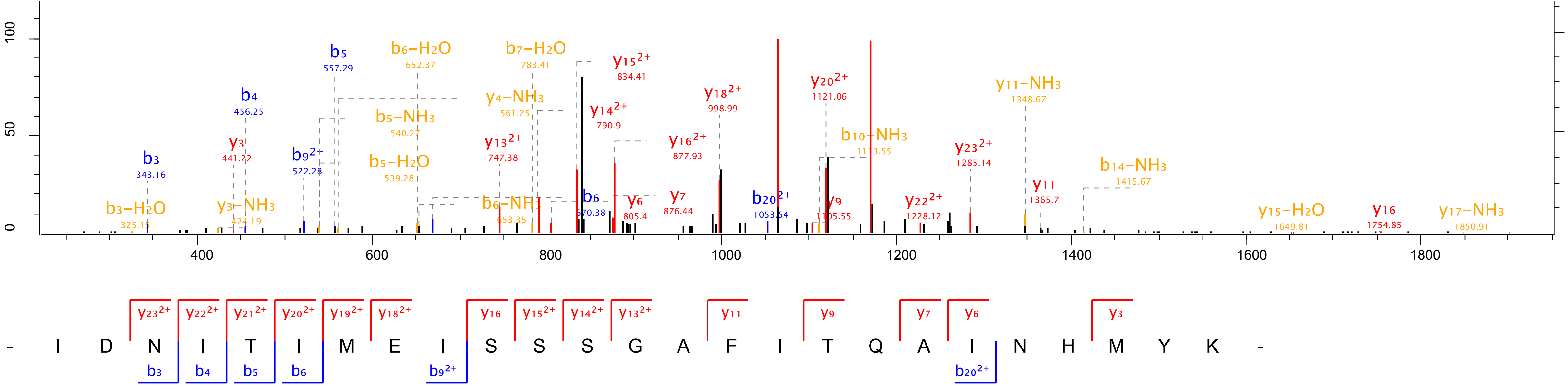

Raw file	Scan	Method	Score	m/z	Gene names
HBT_20130916_BV2_IL43_05	25017	ITMS; CID	80.32	507.64	Ccdc101

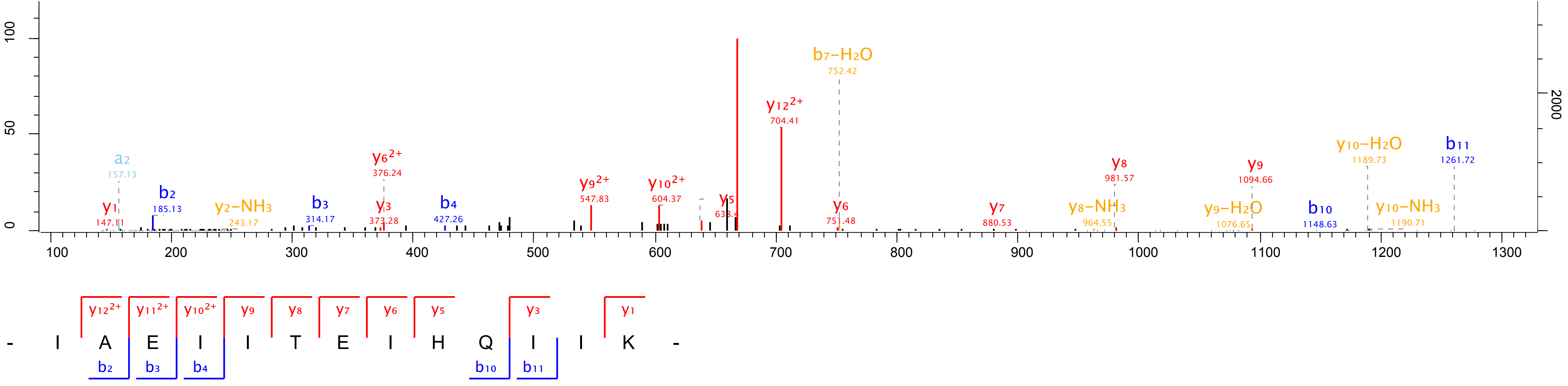

Raw file	Scan	Method	Score	m/z	Gene names
HBT_20130916_BV2_IL43_04	27266	ITMS; CID	100.11	778.93	Srd5a3

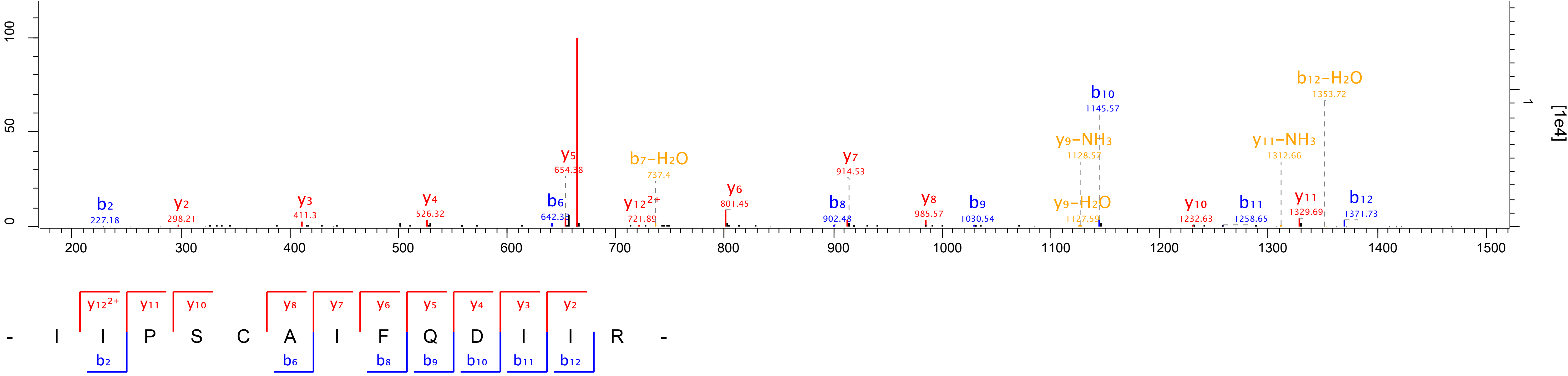

Raw file	Scan	Method	Score	m/z	Gene names
HBT_20130916_BV2_IL43_04	27168	ITMS; CID	102.72	905.01	Wdr37

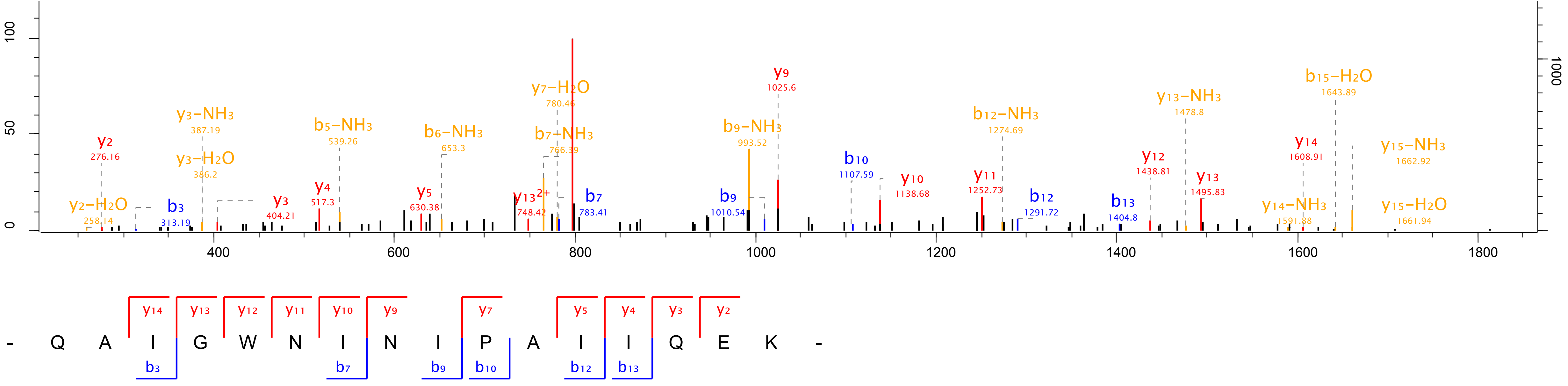

Raw file	Scan	Method	Score	m/z	Gene names
HBT_20130916_BV2_IL43_04	26740	ITMS; CID	145.81	684.92	Mgat5

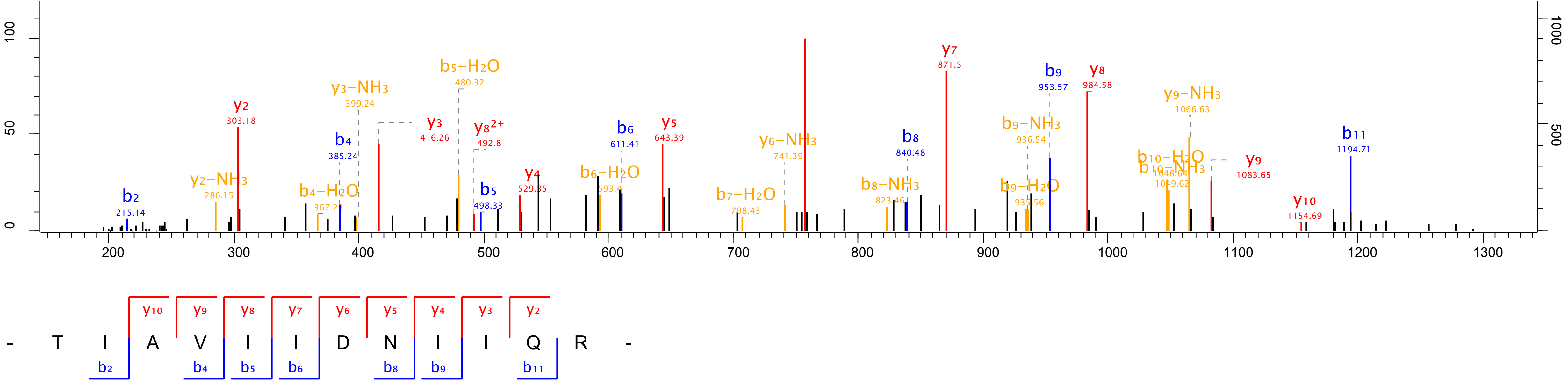

Raw file	Scan	Method	Score	m/z	Gene names
HBT_20130916_BV2_IL43_04	25043	ITMS; CID	118.61	656.38	Ptcd1

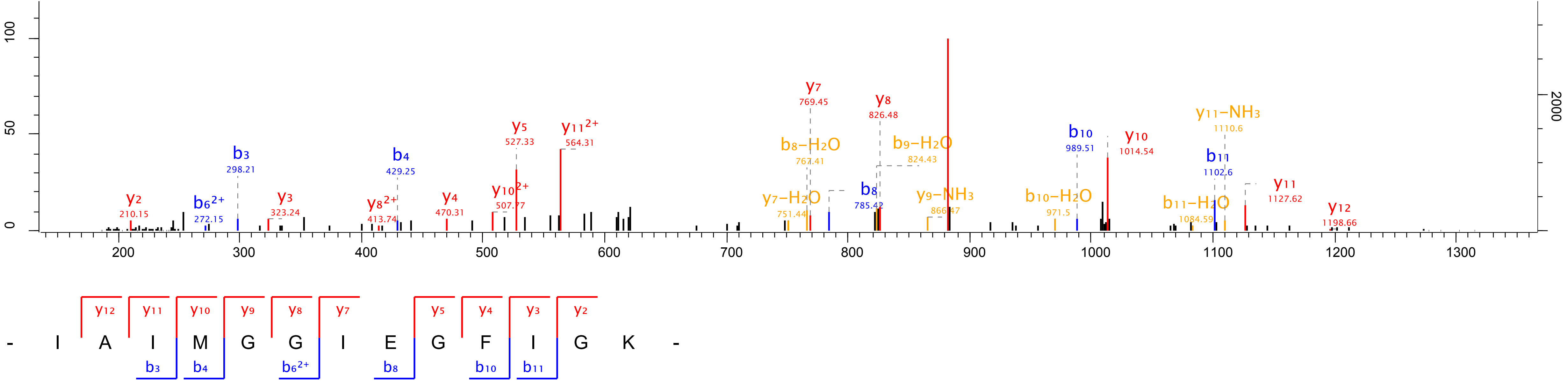

Raw file	Scan	Method	Score	m/z	Gene names
HBT_20130916_BV2_IL43_04	20674	ITMS; CID	128.88	629.87	Polr3f

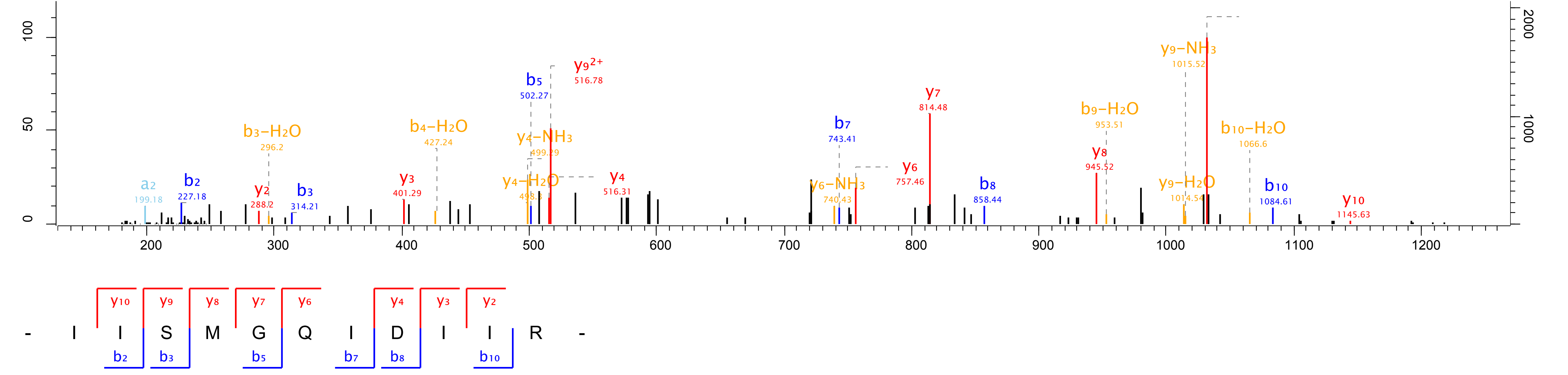

Raw file	Scan	Method	Score	m/z	Gene names
HBT_20130916_BV2_IL43_04	1236	ITMS; CID	104.82	539.28	Ikbip

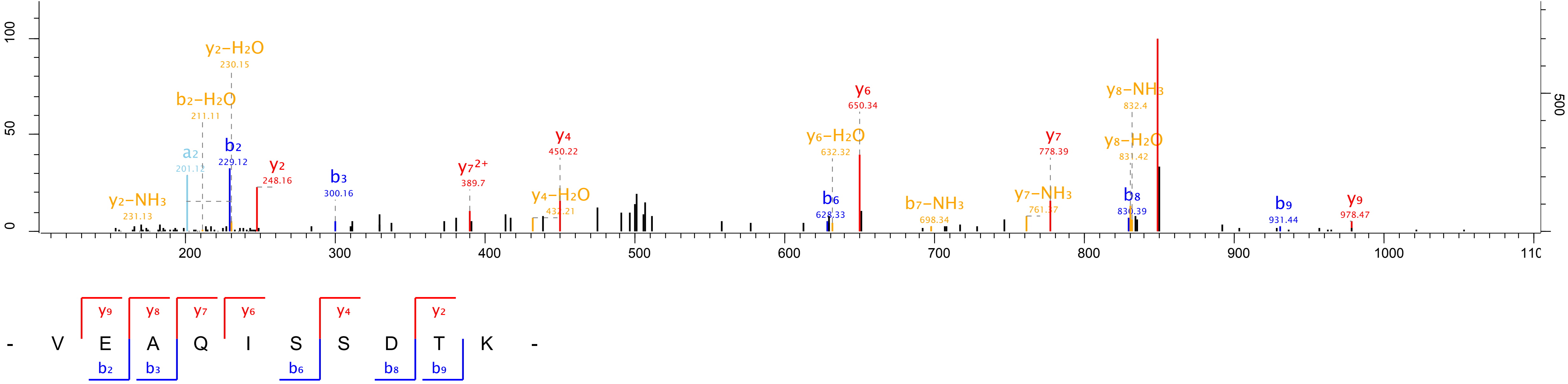

Raw file	Scan	Method	Score	m/z	Gene names
HBT_20130916_BV2_IL43_03	32042	ITMS; CID	123.95	690.38	Phkb

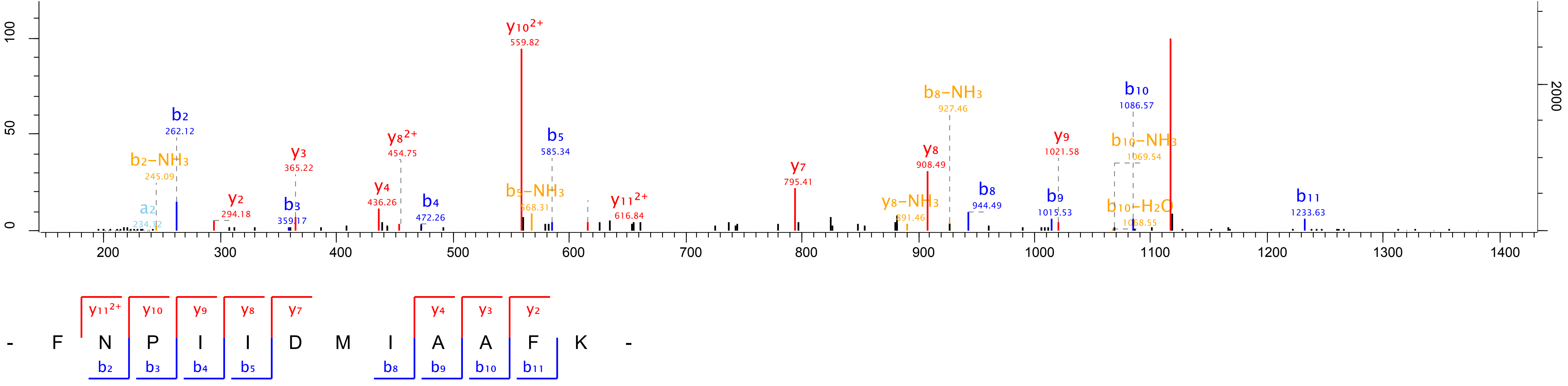

Raw file	Scan	Method	Score	m/z	Gene names
HBT_20130916_BV2_IL43_03	30467	ITMS; CID	92.65	606.32	Gtpbp10

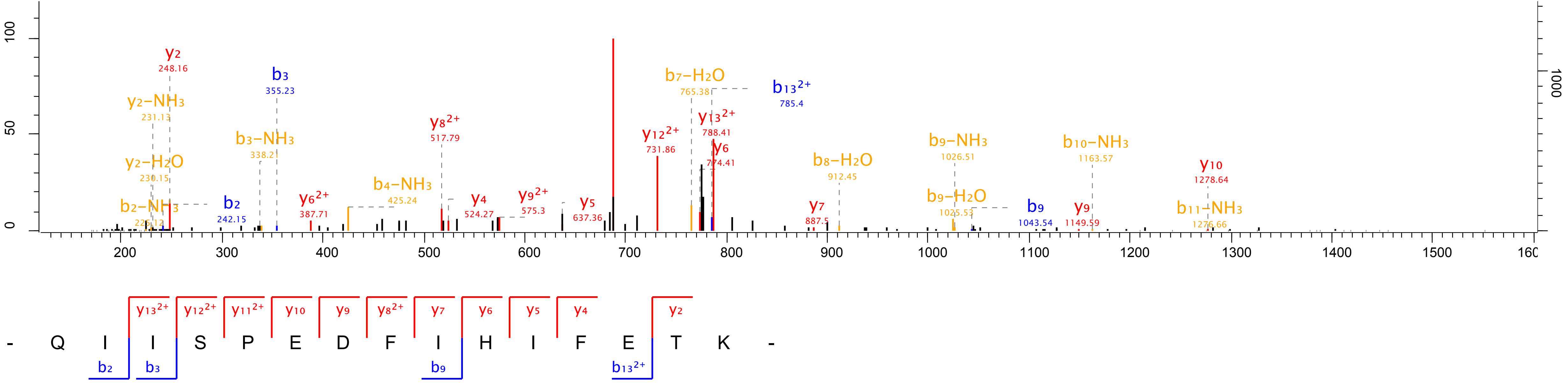

Raw file	Scan	Method	Score	m/z	Gene names
HBT_20130916_BV2_IL43_03	28333	ITMS; CID	144.1	645.35	5730455P16Rik

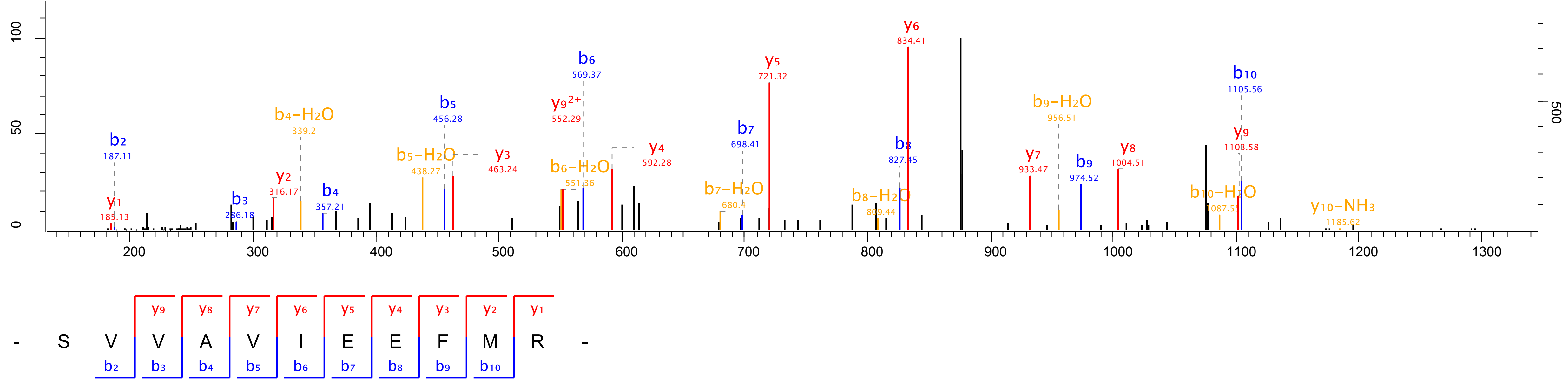

Raw file	Scan	Method	Score	m/z	Gene names
HBT_20130916_BV2_IL43_03	25365	ITMS; CID	123.86	573.83	Snx24

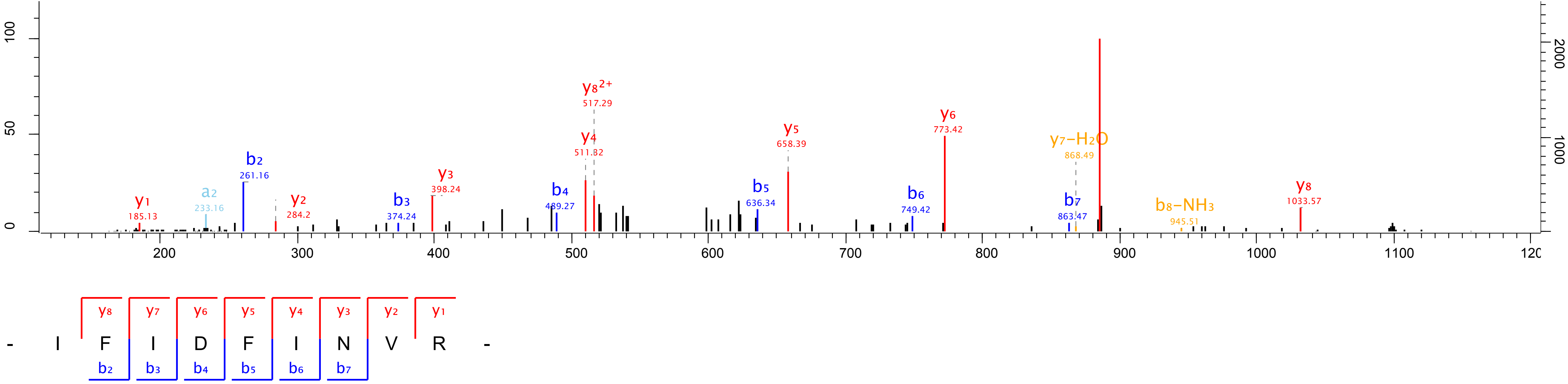

Raw file	Scan	Method	Score	m/z	Gene names
HBT_20130916_BV2_IL43_03	23280	ITMS; CID	87.67	621.34	Nckipsd

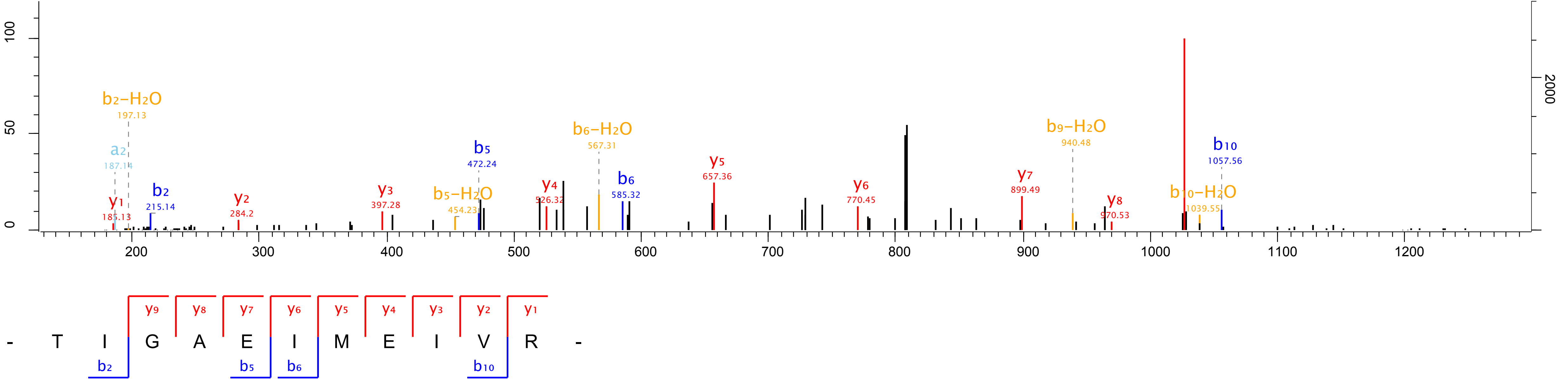

Raw file	Scan	Method	Score	m/z	Gene names
HBT_20130916_BV2_IL43_03	10971	ITMS; CID	144.8	902.95	Slc30a7

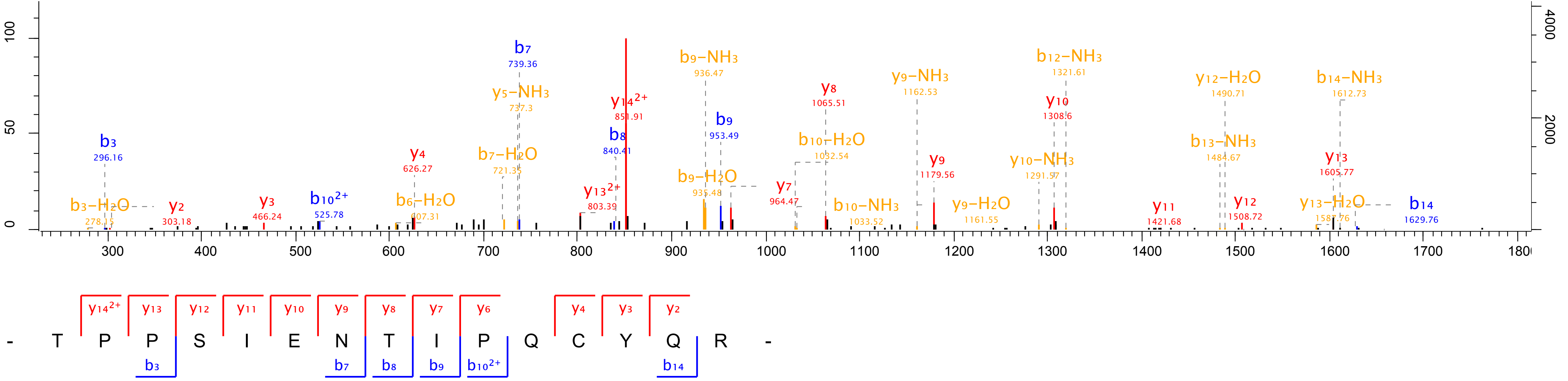

Raw file	Scan	Method	Score	m/z	Gene names
HBT_20130916_BV2_IL43_02	914	ITMS; CID	171.67	625.82	Mpg

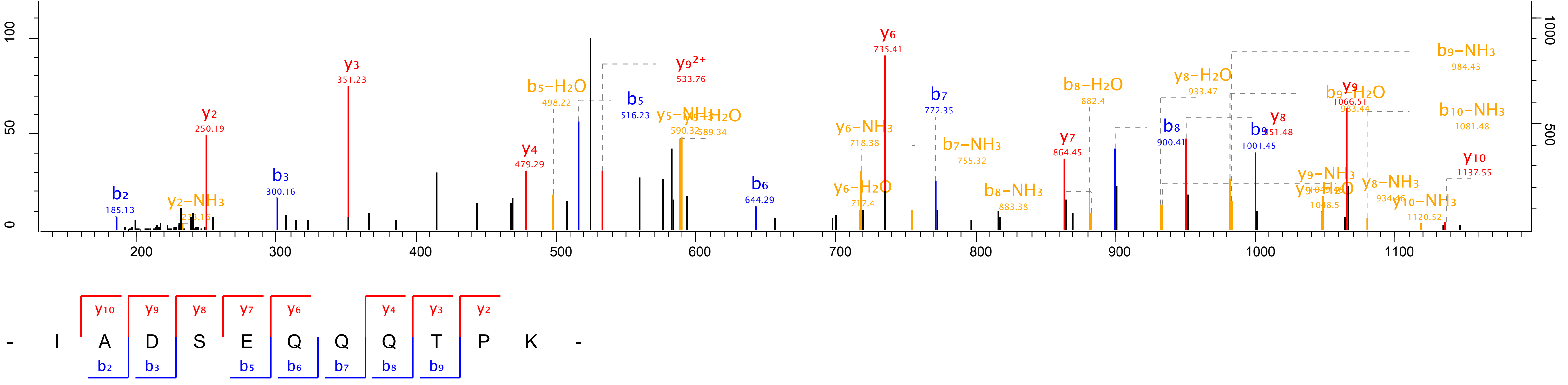

Raw file	Scan	Method	Score	m/z
HBT_20130916_BV2_IL43_02	9053	ITMS; CID	128.22	839.36

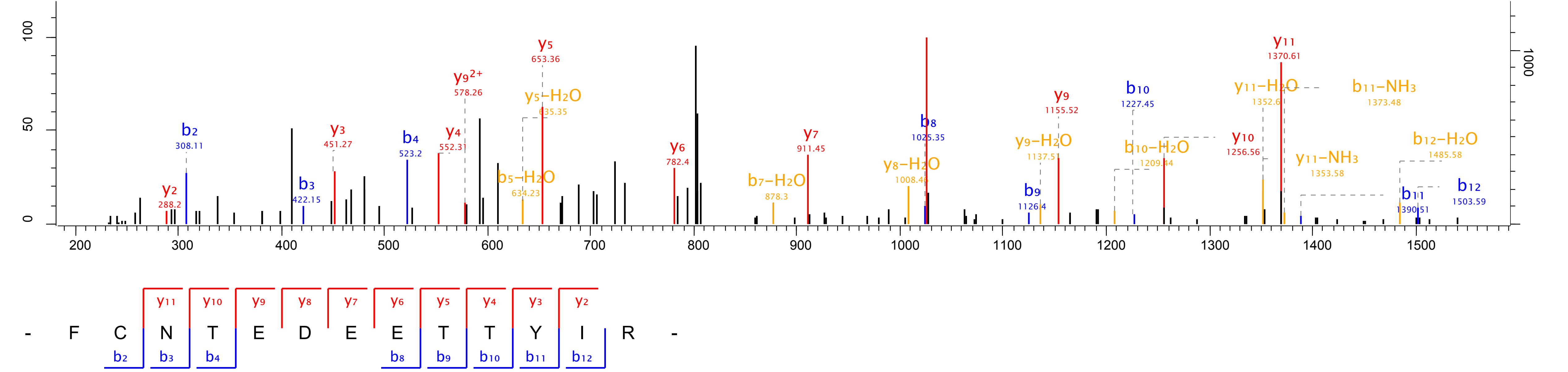

Raw file	Scan	Method	Score	m/z
HBT_20130916_BV2_IL43_02	8728	ITMS; CID	105.1	524.78

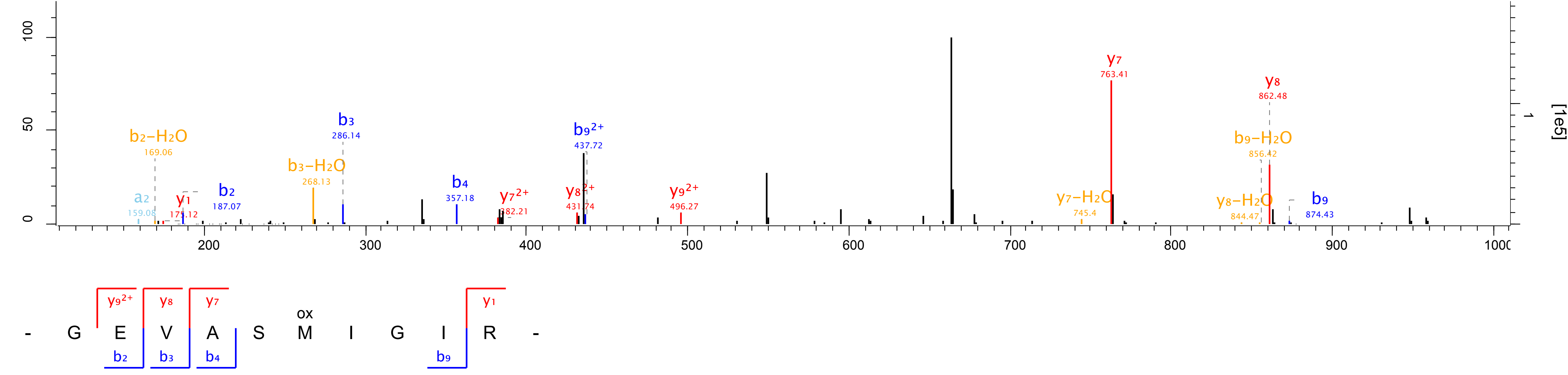

Raw file	Scan	Method	Score	m/z	Gene names
HBT_20130916_BV2_IL43_02	788	ITMS; CID	84.82	716.86	Tfcp2

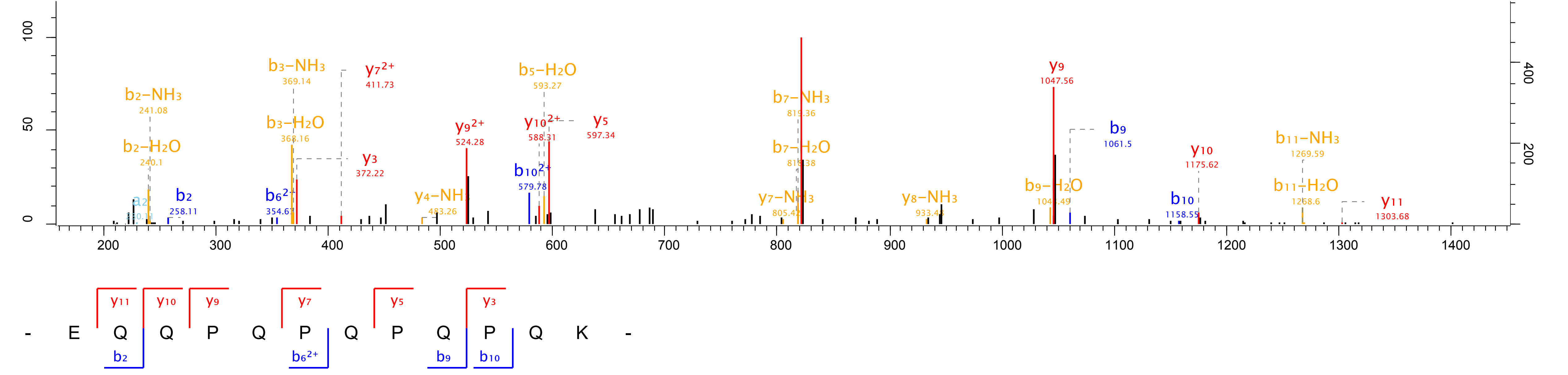

Raw file	Scan	Method	Score	m/z	Gene names
HBT_20130916_BV2_IL43_02	766	ITMS; CID	78.4	698.34	Ppp1r12c

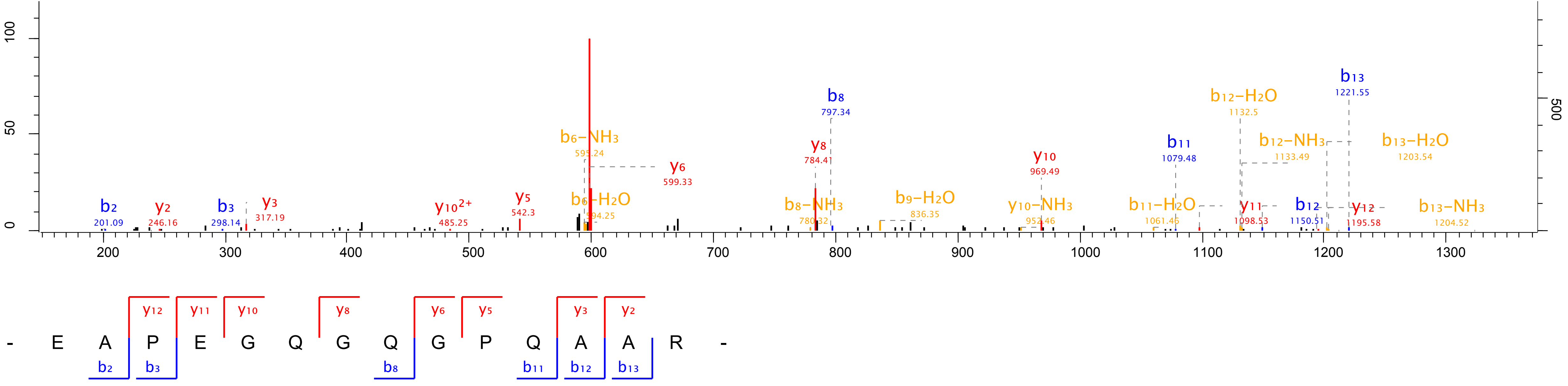

Raw file	Scan	Method	Score	m/z	Gene names
HBT_20130916_BV2_IL43_02	765	ITMS; CID	83.86	764.87	Gtse1

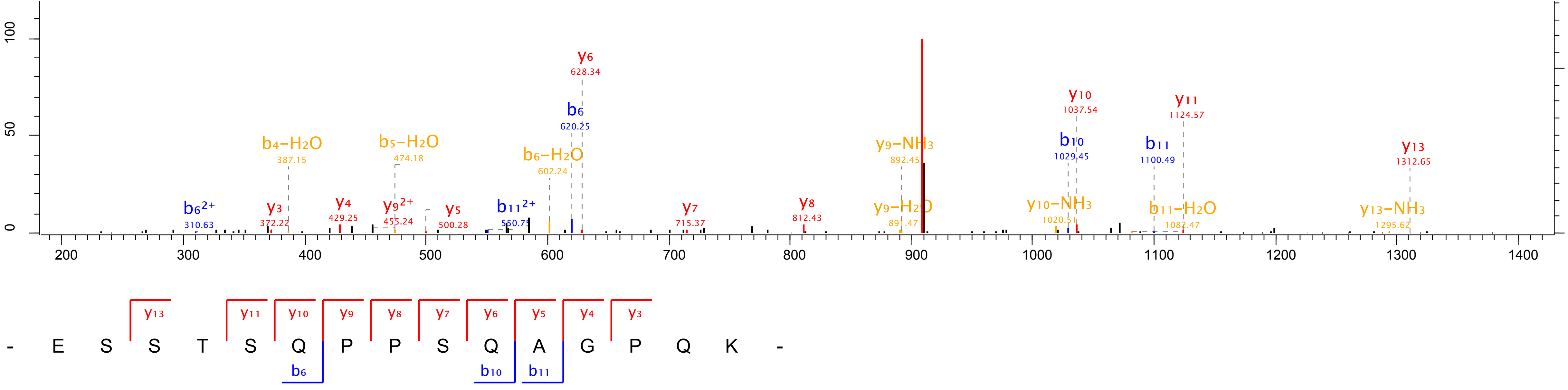

Raw file	Scan	Method	Score	m/z	Gene names
HBT_20130916_BV2_IL43_02	736	ITMS; CID	103.01	747.34	Trim41

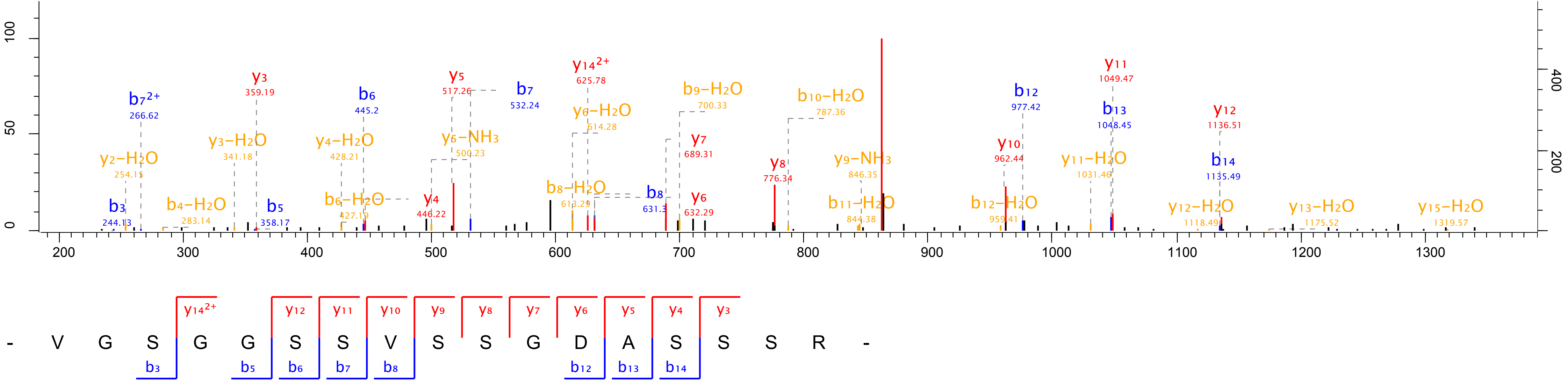

Raw file	Scan	Method	Score	m/z	Gene names
HBT_20130916_BV2_IL43_02	6983	ITMS; CID	125.97	746.87	Cnp

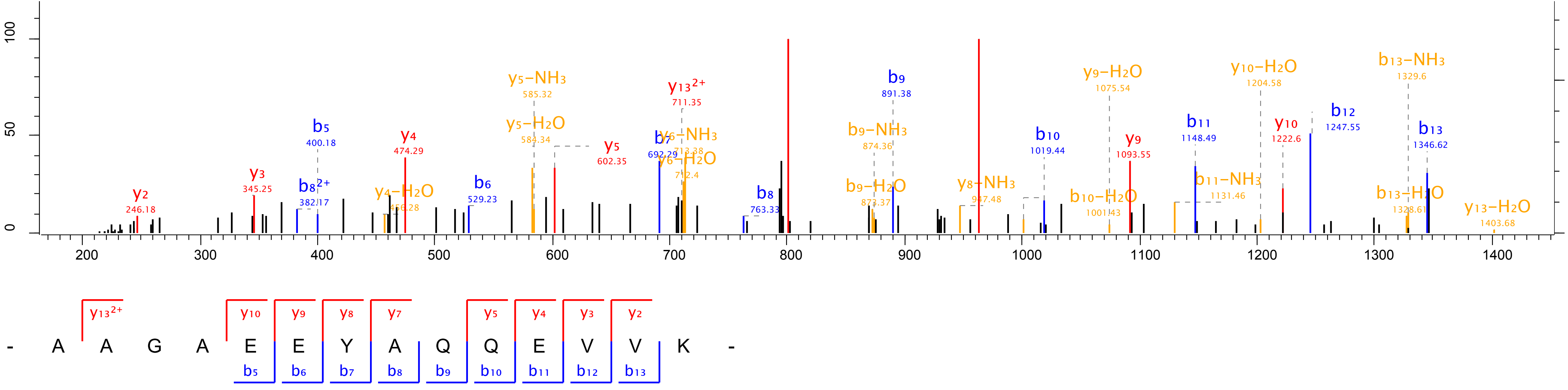

Raw file	Scan	Method	Score	m/z	Gene names
HBT_20130916_BV2_IL43_02	690	ITMS; CID	71.32	815.84	Tgs1

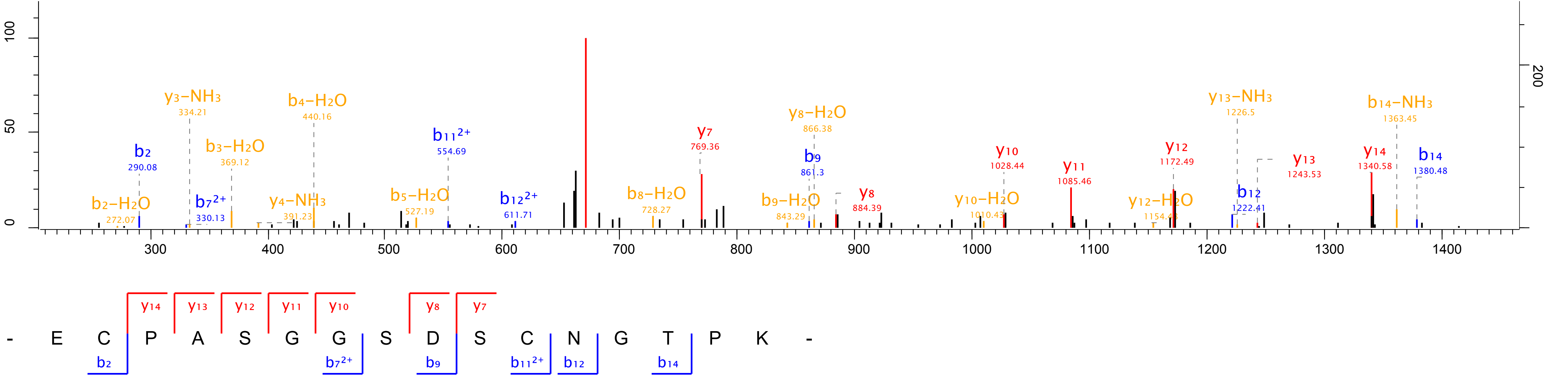

Raw file	Scan	Method	Score	m/z
HBT_20130916_BV2_IL43_02	6497	ITMS; CID	116.86	793.87

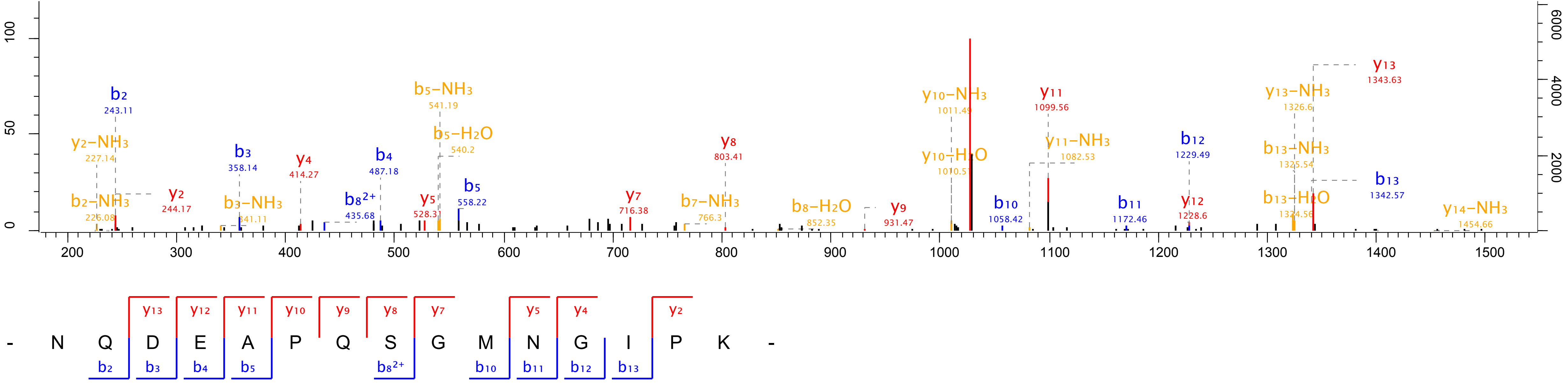

Raw file	Scan	Method	Score	m/z	Gene names
HBT_20130916_BV2_IL43_02	6356	ITMS; CID	123.62	610.85	Ndufb3

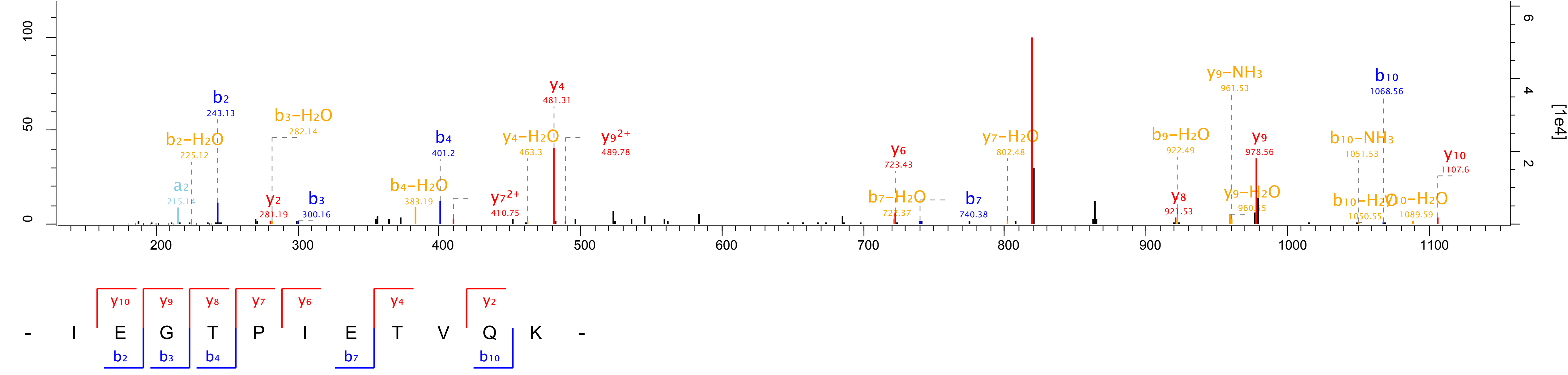

Raw file	Scan	Method	Score	m/z	Gene names
HBT_20130916_BV2_IL43_02	3832	ITMS; CID	117.02	544.28	Tldc1

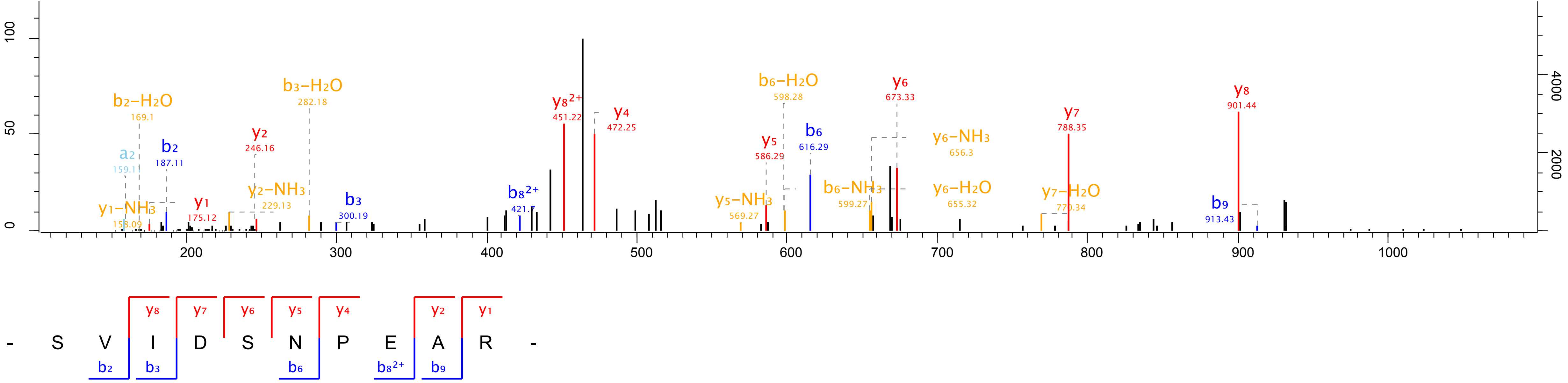

Raw file	Scan	Method	Score	m/z	Gene names
HBT_20130916_BV2_IL43_02	31931	ITMS; CID	124.25	931.98	Pign

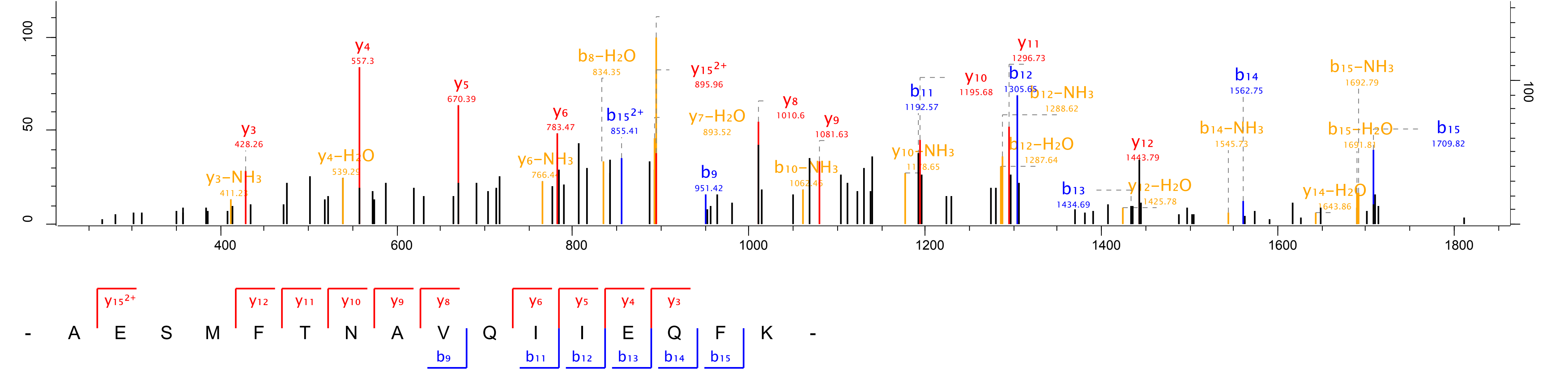

Raw file	Scan	Method	Score	m/z	Gene names
HBT_20130916_BV2_IL43_02	30836	ITMS; CID	123.58	1191.12	Pptc7

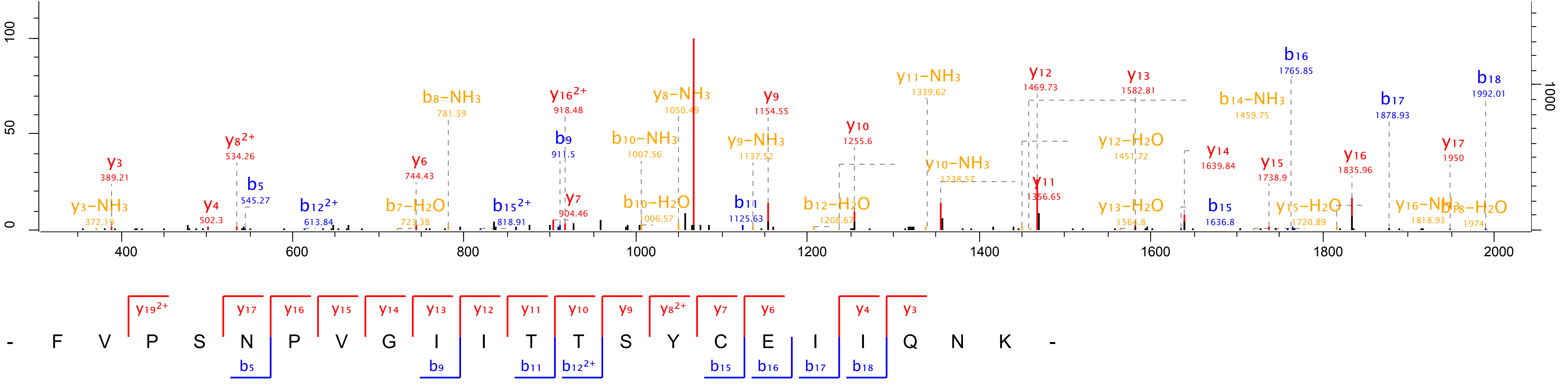

Raw file	Scan	Method	Score	m/z	Gene names
HBT_20130916_BV2_IL43_02	30659	ITMS; CID	90.71	1002.51	Kdm5c

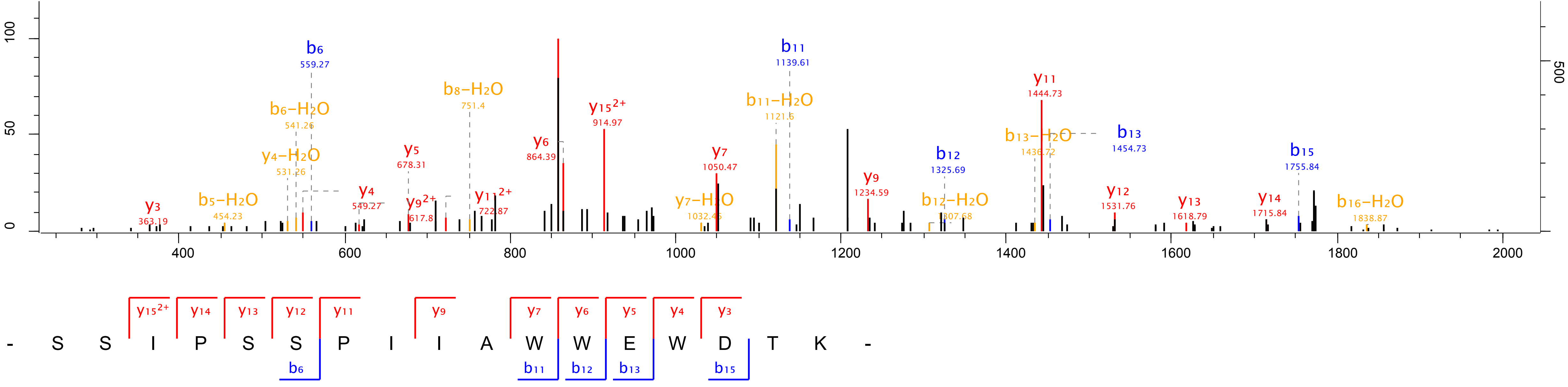

Raw file	Scan	Method	Score	m/z	Gene names
HBT_20130916_BV2_IL43_02	29649	ITMS; CID	191.01	718.43	Cd47

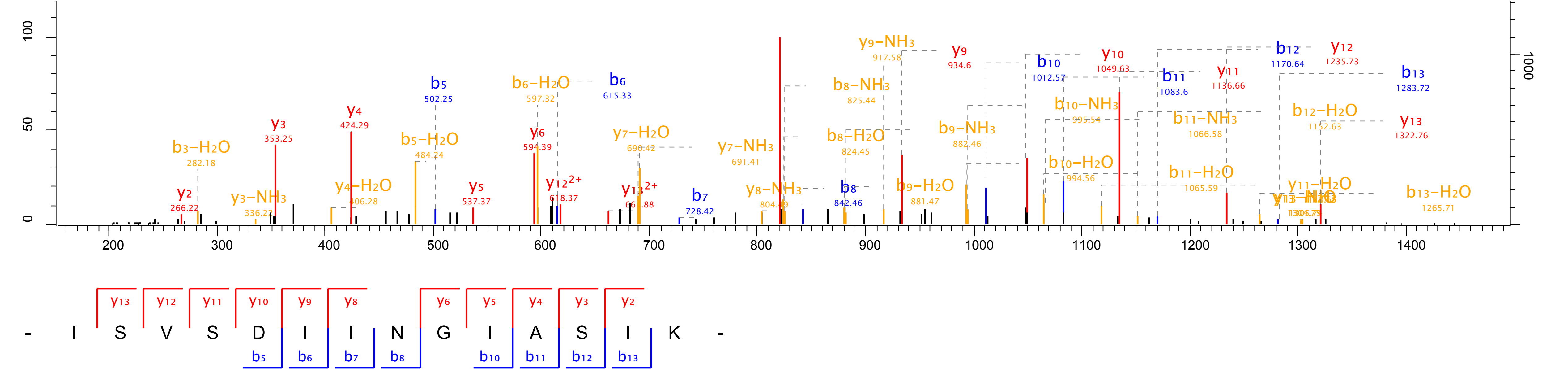

Raw file	Scan	Method	Score	m/z	Gene names
HBT_20130916_BV2_IL43_02	29626	ITMS; CID	245.78	1101.07	Mrps18c

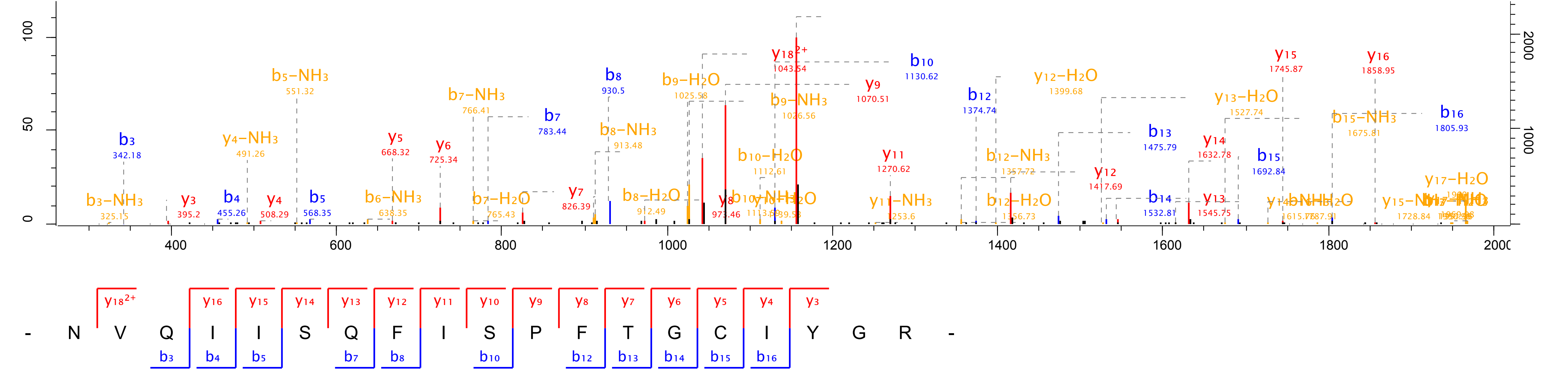

Raw file	Scan	Method	Score	m/z	Gene names
HBT_20130916_BV2_IL43_02	29265	ITMS; CID	69.93	942.55	Skp2

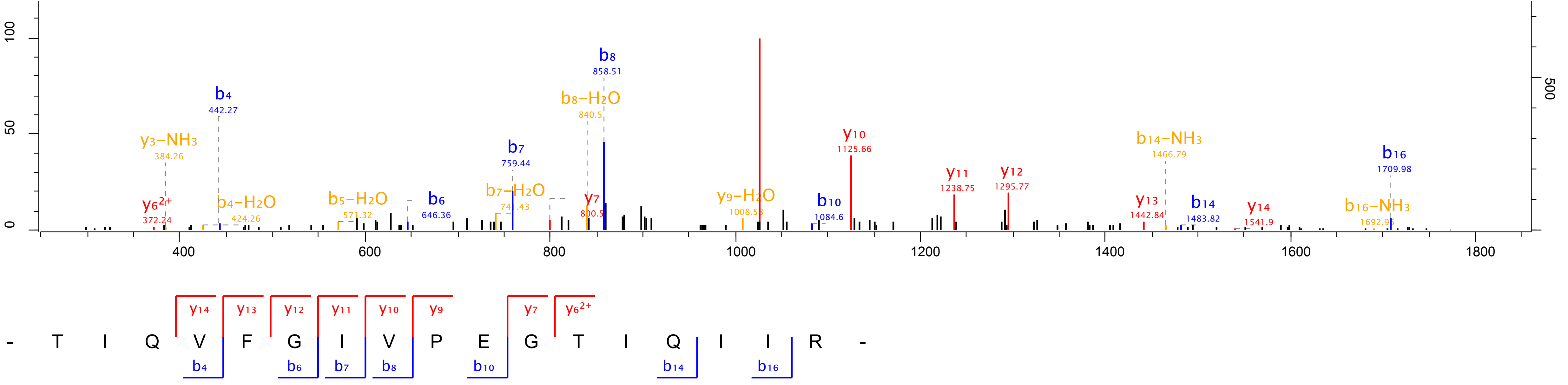

Raw file	Scan	Method	Score	m/z	Gene names
HBT_20130916_BV2_IL43_02	29246	ITMS; CID	82.45	912.52	Cyp4v2

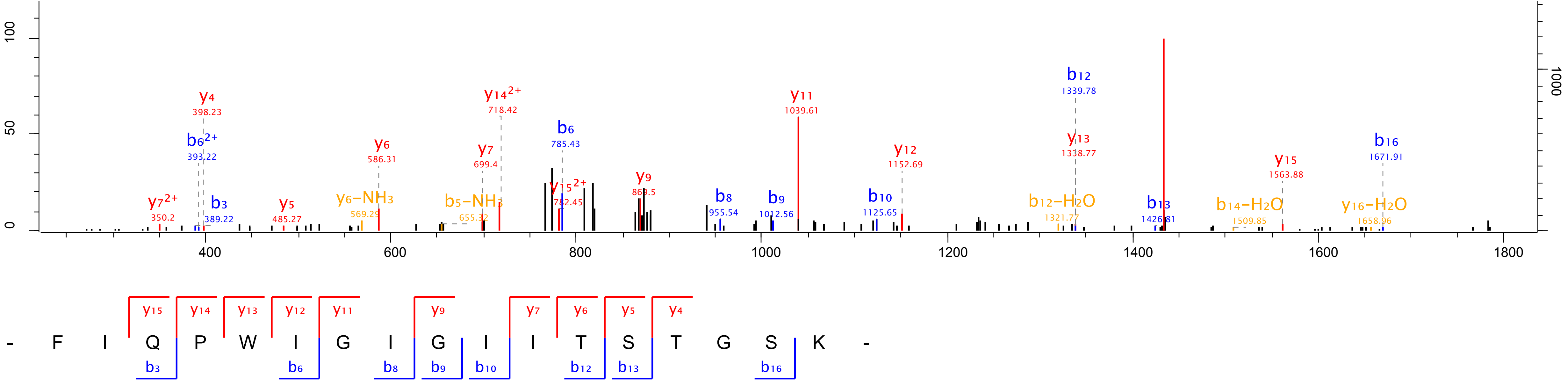

Raw file	Scan	Method	Score	m/z	Gene names
HBT_20130916_BV2_IL43_02	29178	ITMS; CID	87.45	835.45	Usp16

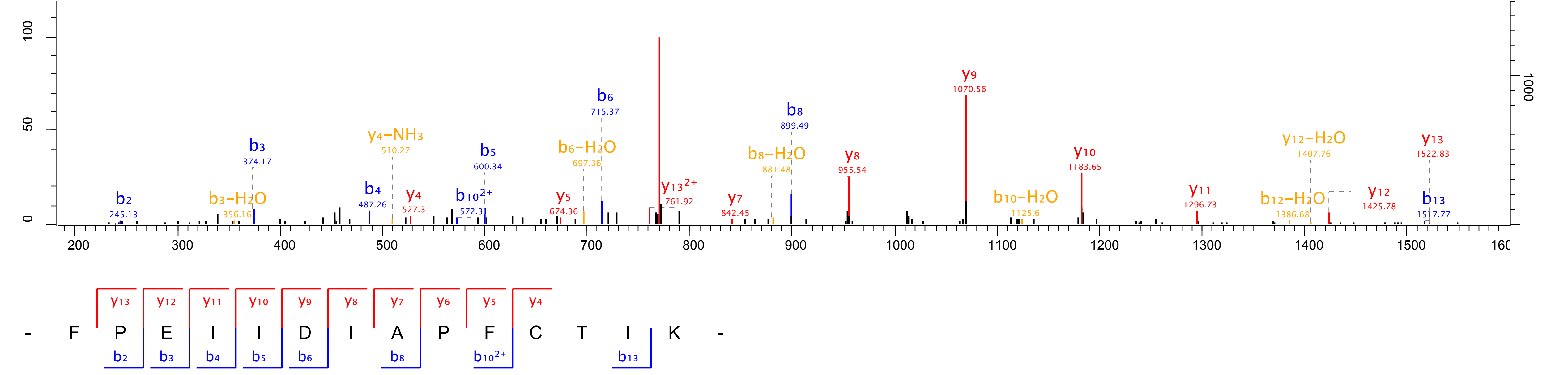

Raw file	Scan	Method	Score	m/z	Gene names
HBT_20130916_BV2_IL43_02	27984	ITMS; CID	115.56	917.47	Tmem168

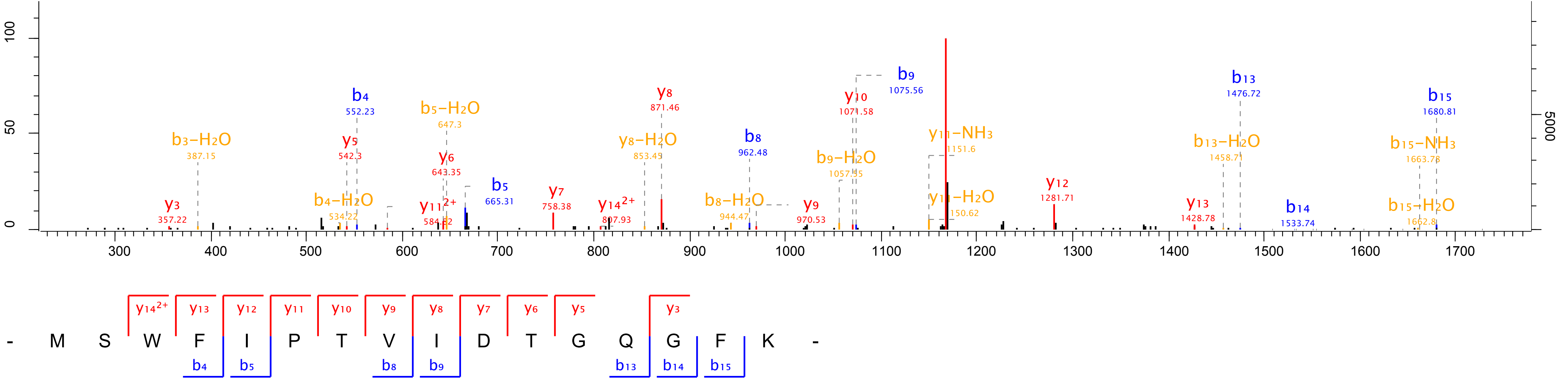

Raw file	Scan	Method	Score	m/z	Gene names
HBT_20130916_BV2_IL43_02	25072	ITMS; CID	103.13	849.91	Mbd1

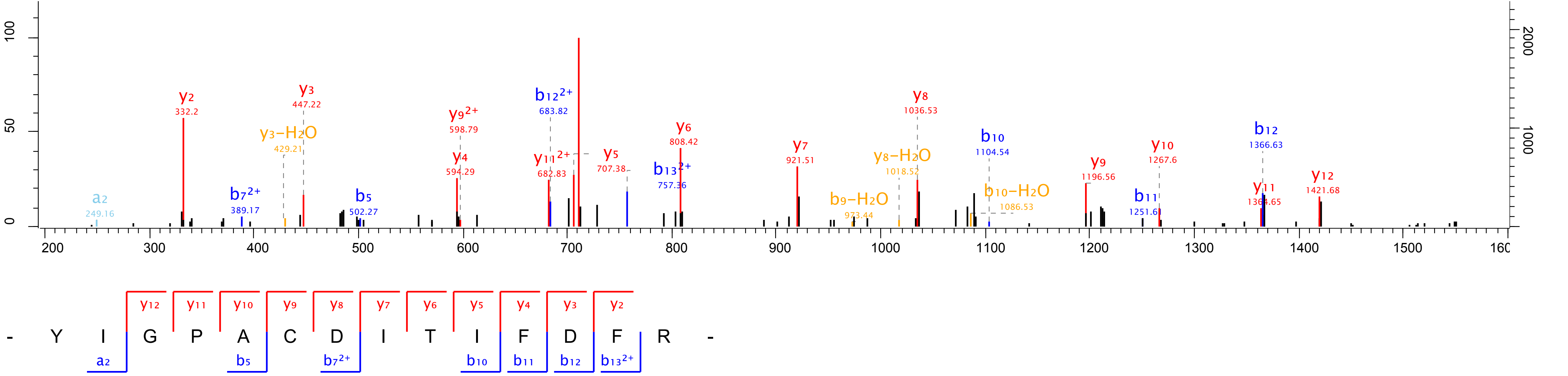

Raw file	Scan	Method	Score	m/z	Gene names
HBT_20130916_BV2_IL43_02	24811	ITMS; CID	110.08	636.37	Cradd

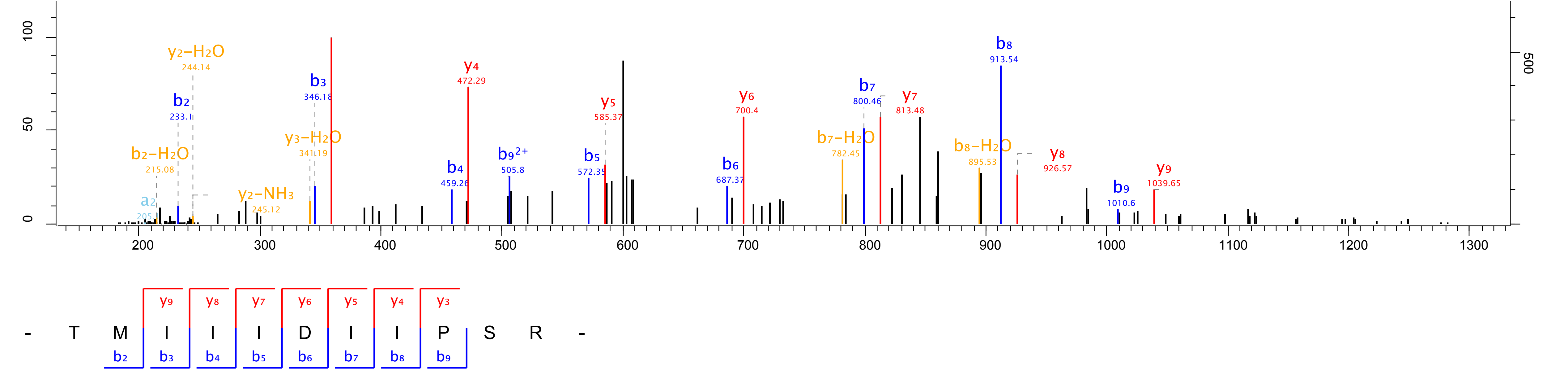

Raw file	Scan	Method	Score	m/z
HBT_20130916_BV2_IL43_02	24128	ITMS; CID	143.5	588.83

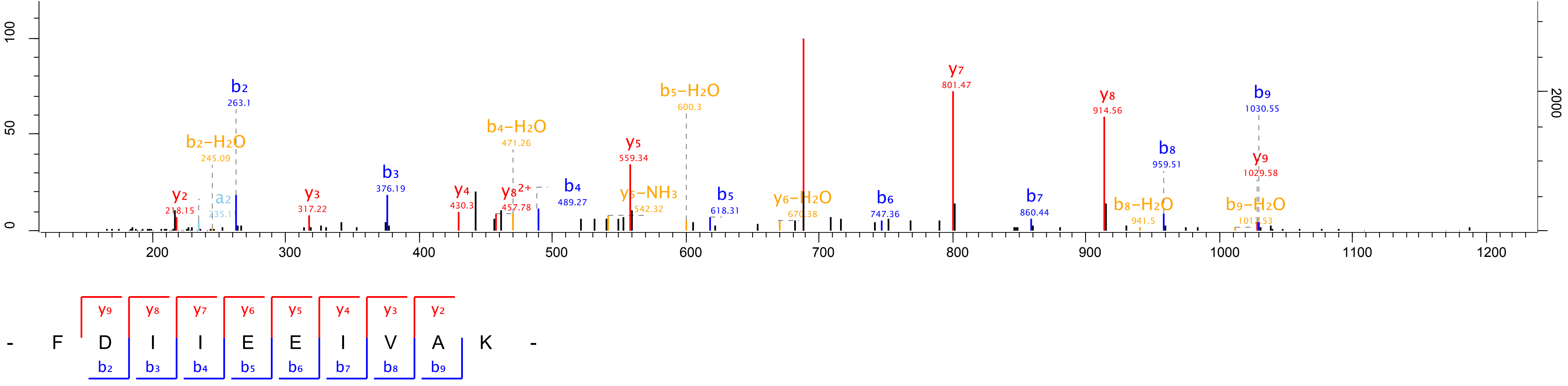

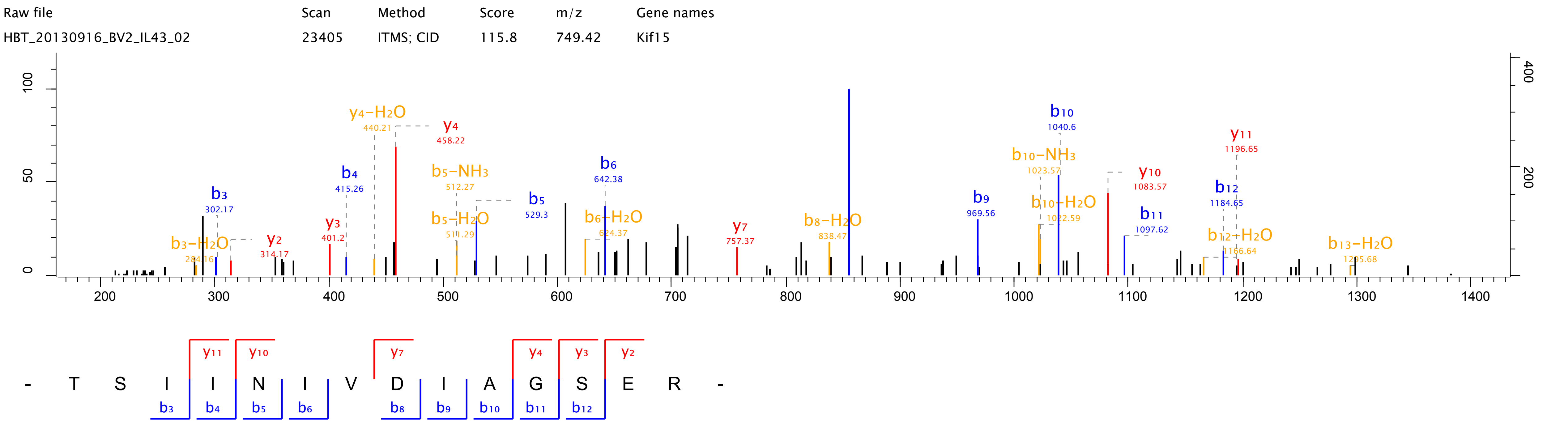
Raw file
HBT_20130916_BV2_IL43_02
Scan
23405
Method
ITMS; CID
Score
115.8
m/z
749.42
Gene names
Kif15
b3 302.17
b3−H2O 284.16
y2 314.17
y3 401.2
b4 415.26
y4−H2O 440.21
y4 458.22
b5−NH3 512.27
b5−H2O 511.29
b5 529.3
b6 642.38
b6−H2O 624.37
y7 757.37
b8−H2O 838.47
b9 969.56
b10−NH3 1023.57
b10−H2O 1022.59
b10 1040.6
y10 1083.57
b11 1097.62
b12 1184.65
b12−H2O 1166.64
y11 1196.65
b13−H2O 1295.68
- T S I I N I V D I A G S E R -

Raw file	Scan	Method	Score	m/z	Gene names
HBT_20130916_BV2_IL43_02	22769	ITMS; CID	84.4	914.97	Haus7

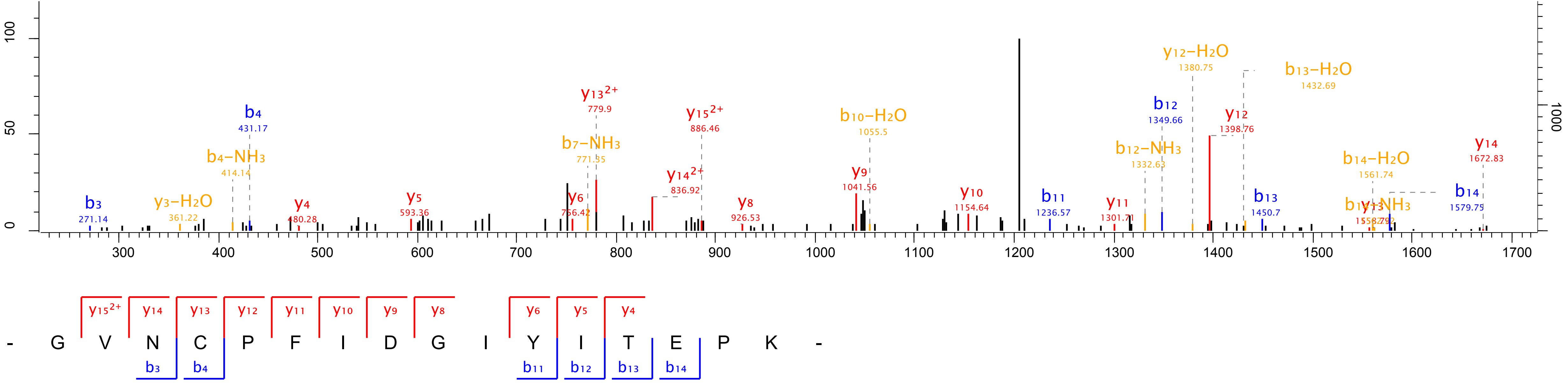

Raw file	Scan	Method	Score	m/z	Gene names
HBT_20130916_BV2_IL43_02	21734	ITMS; CID	115.33	622.35	Pcyox1l

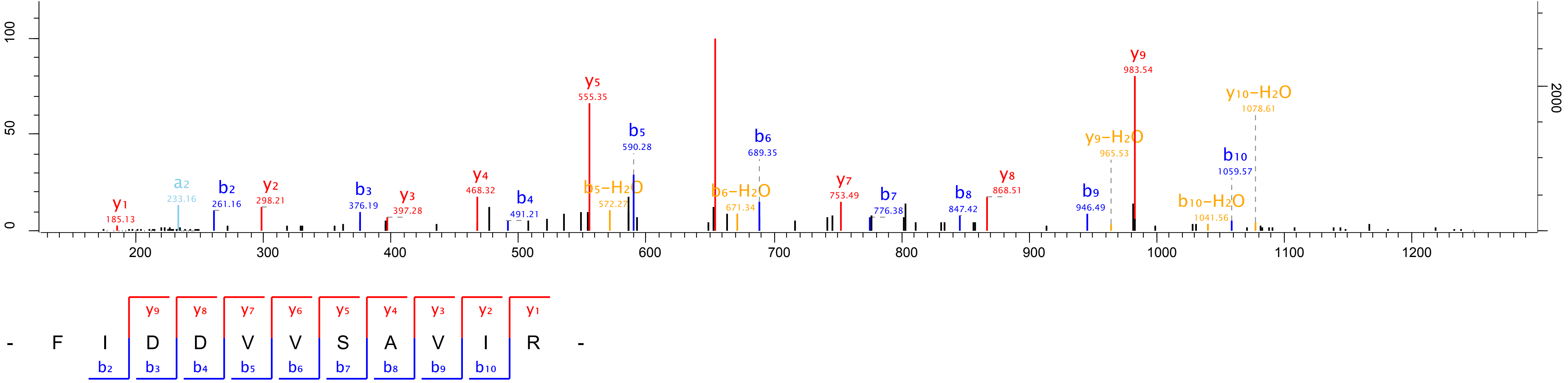

Raw file	Scan	Method	Score	m/z	Gene names
HBT_20130916_BV2_IL43_02	21685	ITMS; CID	68.58	910.48	Twistnb

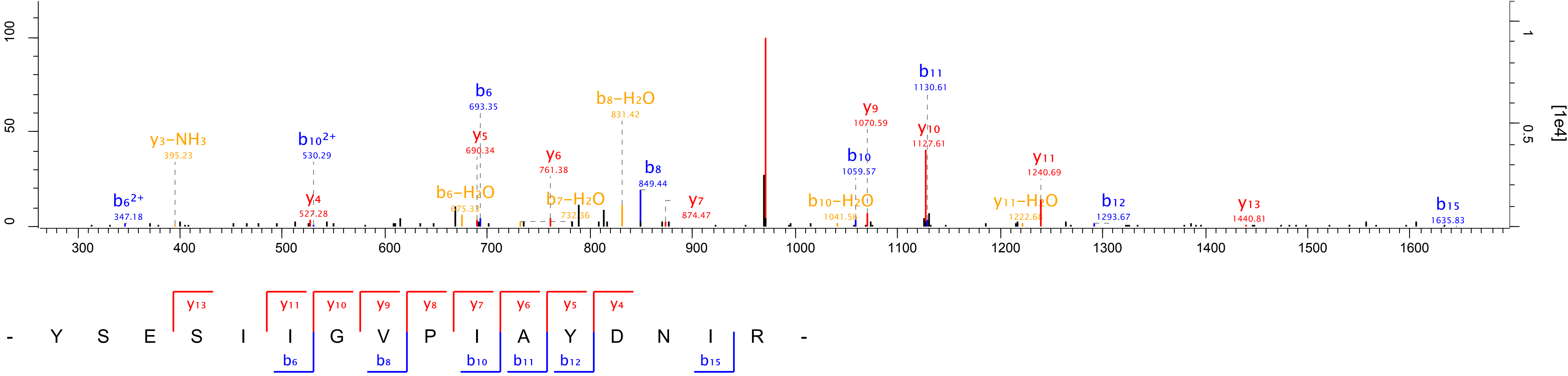

Raw file	Scan	Method	Score	m/z	Gene names
HBT_20130916_BV2_IL43_02	21364	ITMS; CID	169.65	564.85	Coil

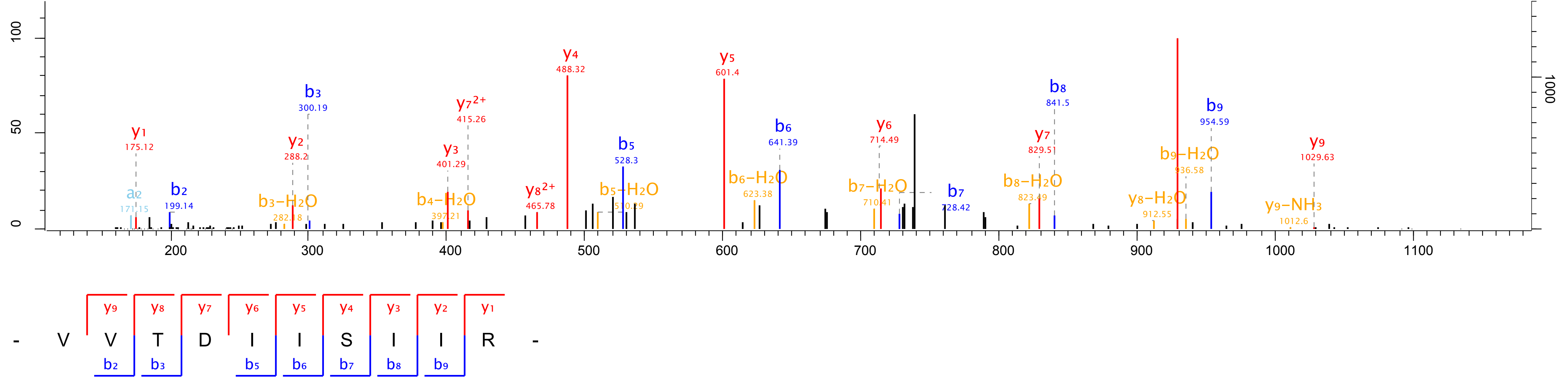

Raw file	Scan	Method	Score	m/z	Gene names
HBT_20130916_BV2_IL43_02	18179	ITMS; CID	130.75	572.3	Klhdc2

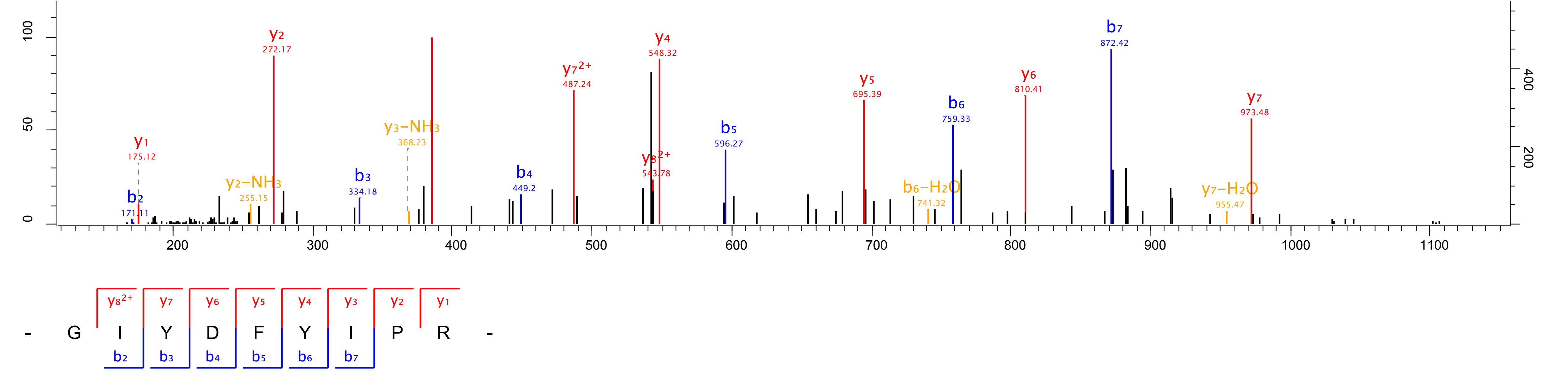

Raw file	Scan	Method	Score	m/z	Gene names
HBT_20130916_BV2_IL43_02	18019	ITMS; CID	122.66	707.36	Pfdn4

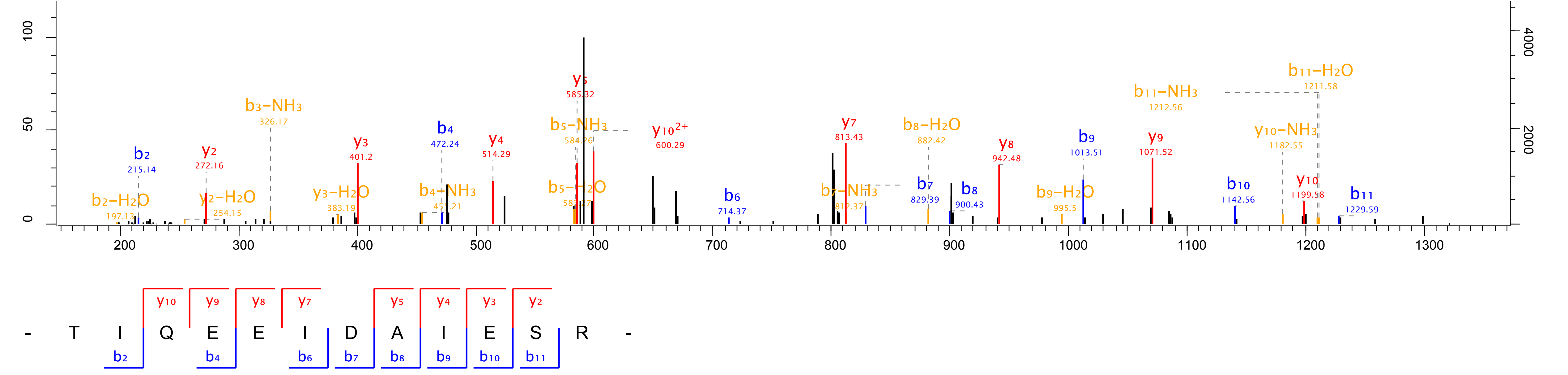

Raw file	Scan	Method	Score	m/z	Gene names
HBT_20130916_BV2_IL43_02	17094	ITMS; CID	97.27	826.9	Pex7

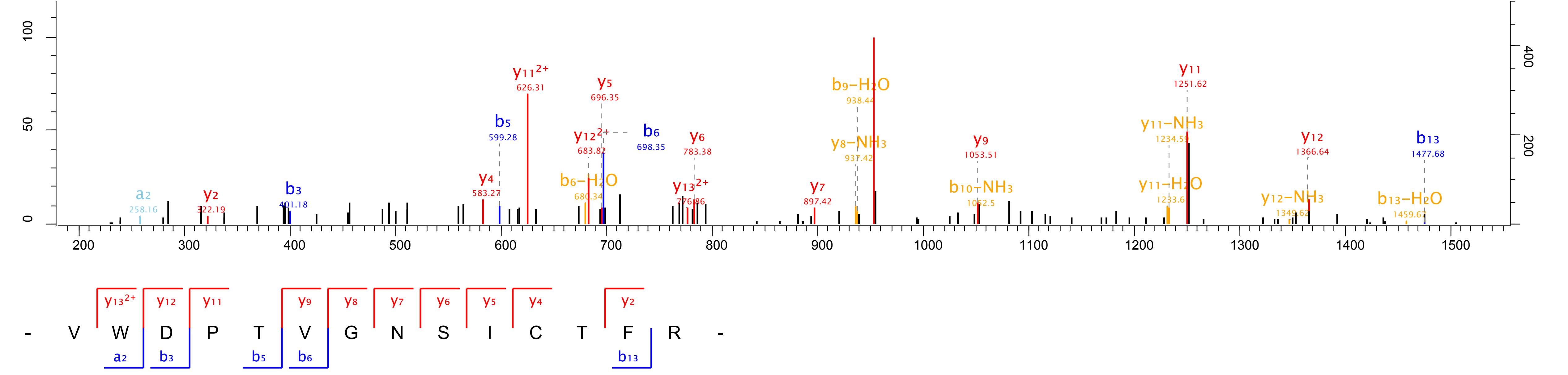

Raw file	Scan	Method	Score	m/z	Gene names
HBT_20130916_BV2_IL43_02	16592	ITMS; CID	109.16	730.88	Jagn1

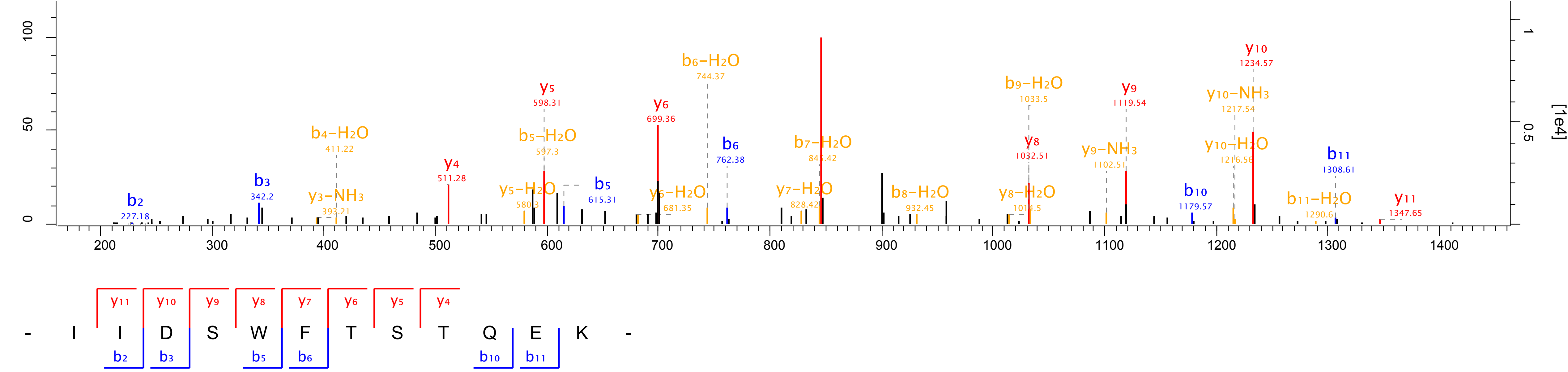

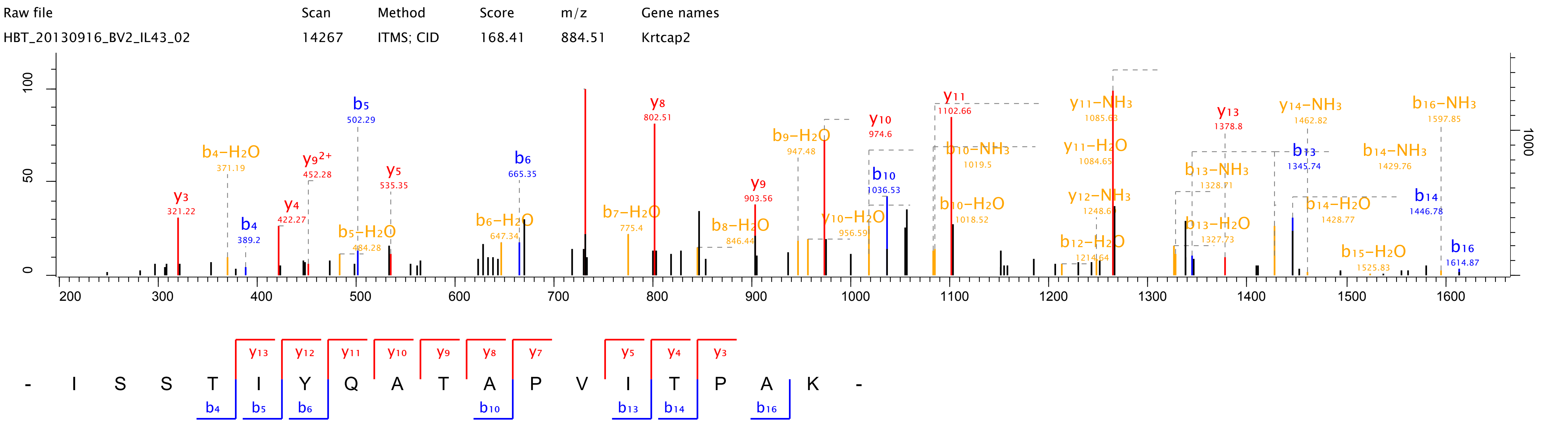

Raw file
HBT_20130916_BV2_IL43_02
Scan
14267
Method
ITMS; CID
Score
168.41
m/z
884.51
Gene names
Krtcap2
0
50
100
1000
200
300
400
500
600
700
800
900
1000
1100
1200
1300
1400
1500
1600
y3 321.22
b4-H2O 371.19
b4 389.2
y4 422.27
y9 2+ 452.28
b5-H2O 484.28
b5 502.29
y5 535.35
b6-H2O 647.34
b6 665.35
b7-H2O 775.4
y8 802.51
b8-H2O 846.44
y9 903.56
b9-H2O 947.48
y10-H2O 956.59
y10 974.6
b10-NH3 1019.5
b10-H2O 1018.52
b10 1036.53
y11 1102.66
y11-NH3 1085.63
y11-H2O 1084.65
y12-NH3 1248.6
b12-H2O 1214.64
b13-NH3 1328.71
b13-H2O 1327.73
y13 1378.8
b13 1345.74
y14-NH3 1462.82
b14-NH3 1429.76
b14-H2O 1428.77
b14 1446.78
b15-H2O 1525.83
b16-NH3 1597.85
b16 1614.87
y13 y12 y11 y10 y9 y8 y7 y5 y4 y3
- I S S T I Y Q A T A P V I T P A K -
b4 b5 b6 b10 b13 b14 b16

Raw file	Scan	Method	Score	m/z	Gene names
HBT_20130916_BV2_IL43_02	13834	ITMS; CID	99.5	567.37	Pet117

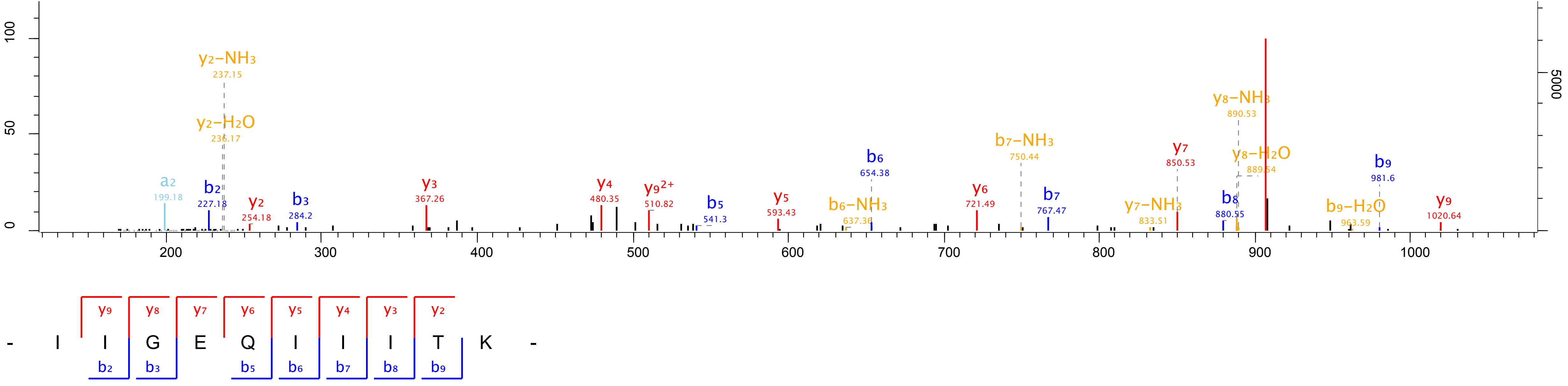

Raw file	Scan	Method	Score	m/z	Gene names
HBT_20130916_BV2_IL43_02	1261	ITMS; CID	151.49	700.83	Itpk1

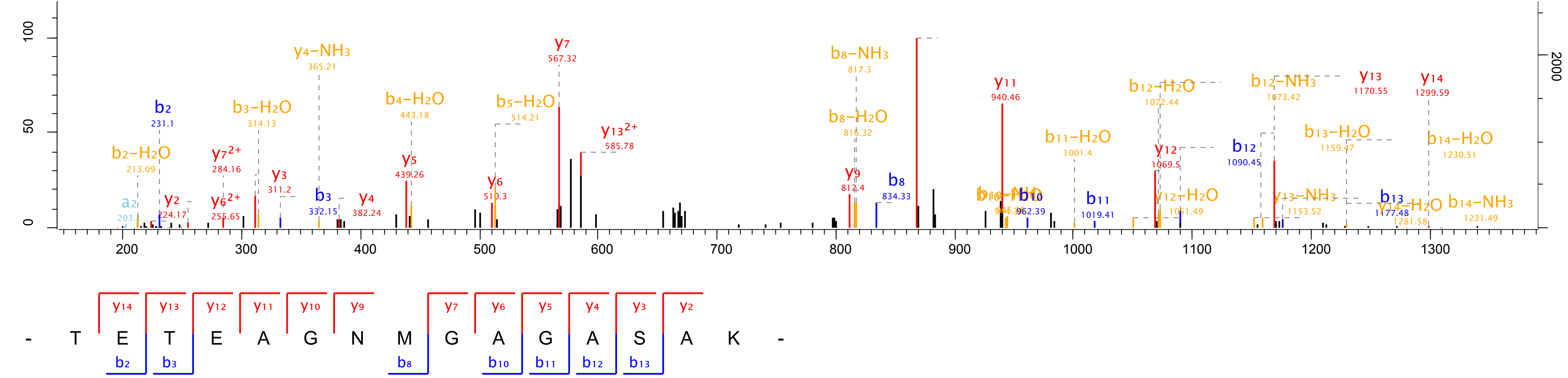

Raw file	Scan	Method	Score	m/z	Gene names
HBT_20130916_BV2_IL43_02	1034	ITMS; CID	131.79	601.29	Cenpt

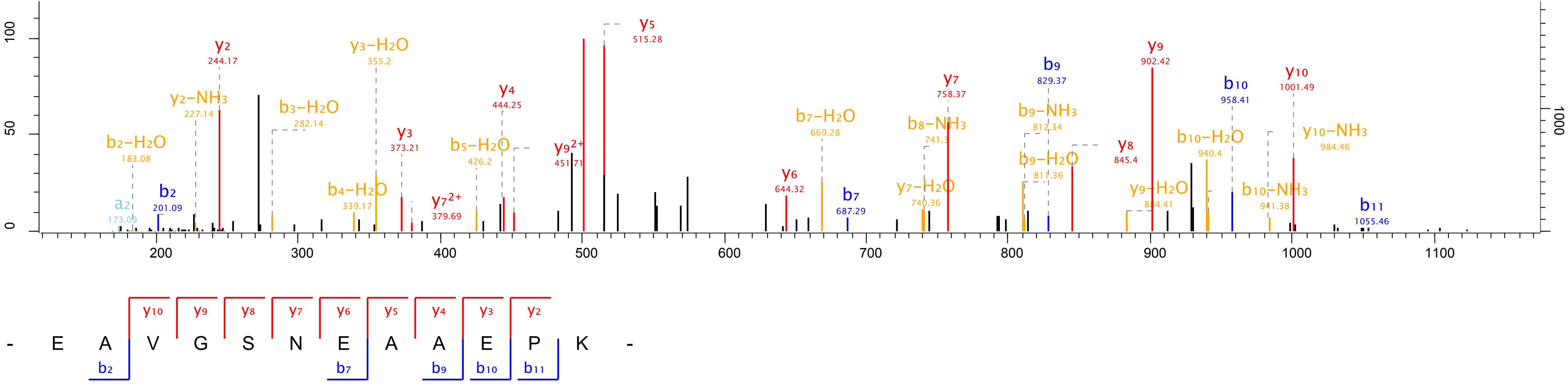

Raw file	Scan	Method	Score	m/z	Gene names
HBT_20130916_BV2_IL43_01	9521	ITMS; CID	114.9	941.97	Dctpp1

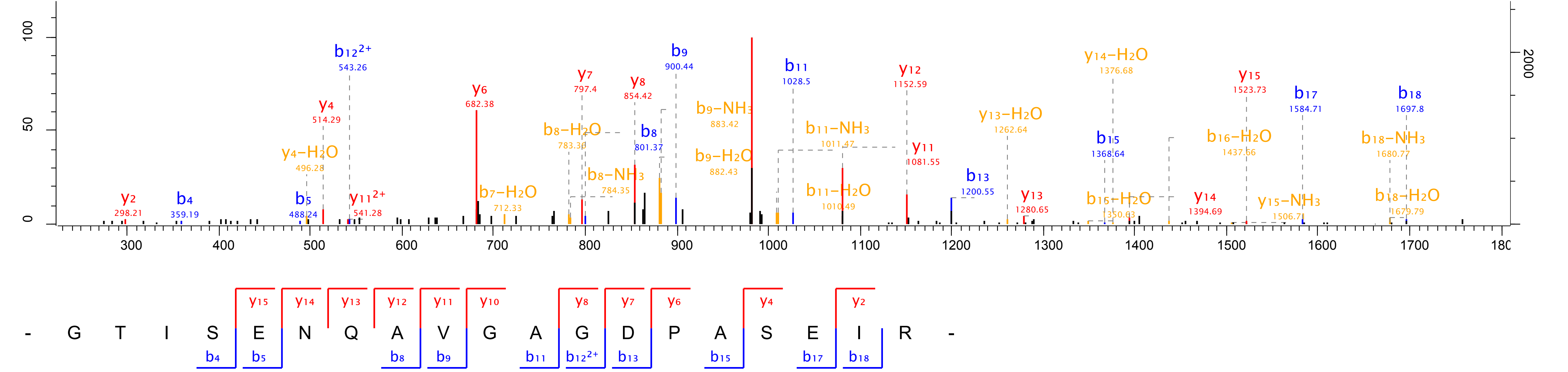

Raw file	Scan	Method	Score	m/z	Gene names
HBT_20130916_BV2_IL43_01	8914	ITMS; CID	131.11	524.81	Alg8

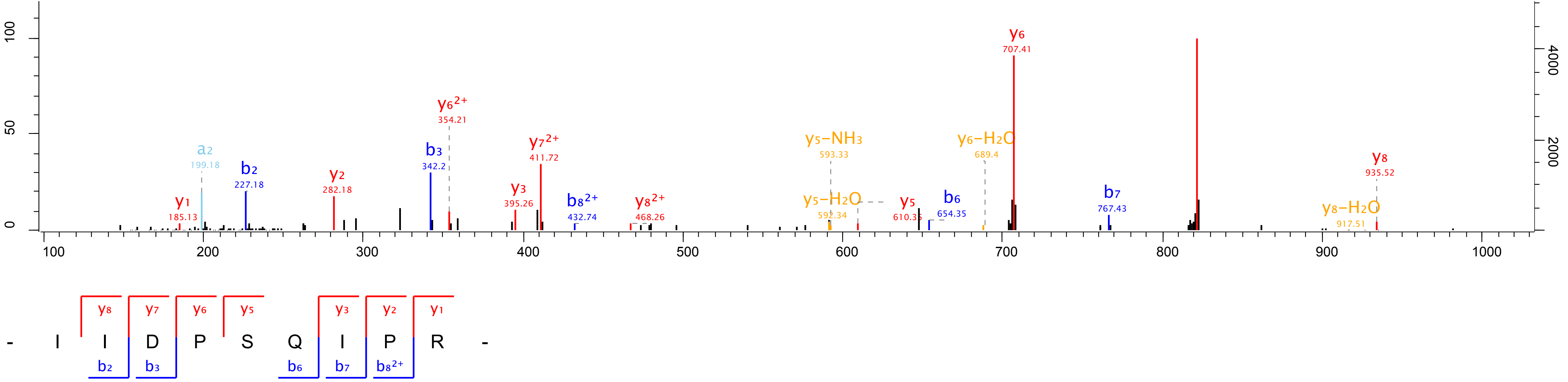

Raw file	Scan	Method	Score	m/z	Gene names
HBT_20130916_BV2_IL43_01	8434	ITMS; CID	157.02	943.47	Smg8

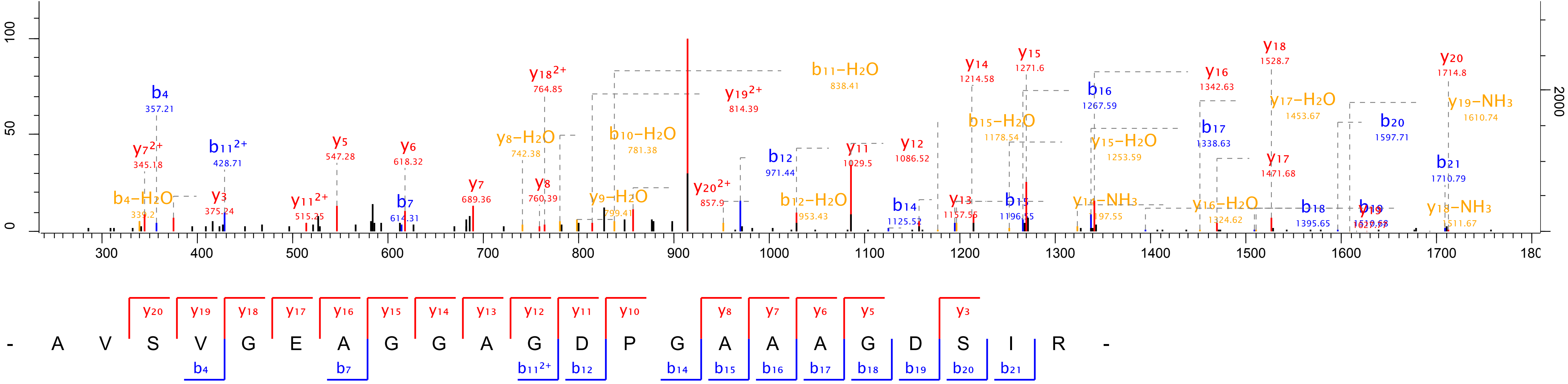

Raw file	Scan	Method	Score	m/z	Gene names
HBT_20130916_BV2_IL43_01	7184	ITMS; CID	101.5	817.89	Rnf115

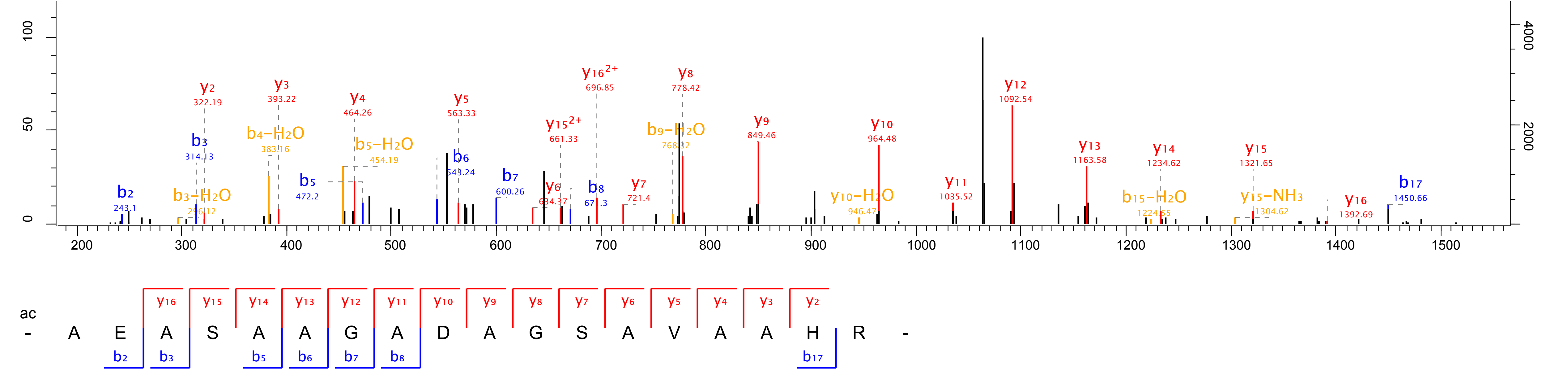

Raw file	Scan	Method	Score	m/z
HBT_20130916_BV2_IL43_01	6446	ITMS; CID	99.79	561.28

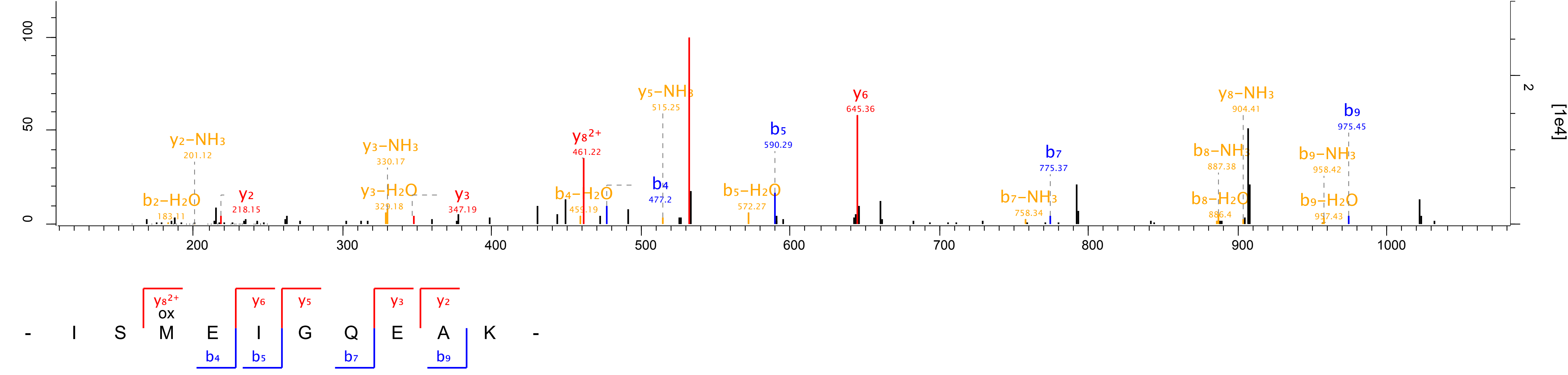

Raw file	Scan	Method	Score	m/z	Gene names
HBT_20130916_BV2_IL43_01	5714	ITMS; CID	86.29	622.81	Rcn1

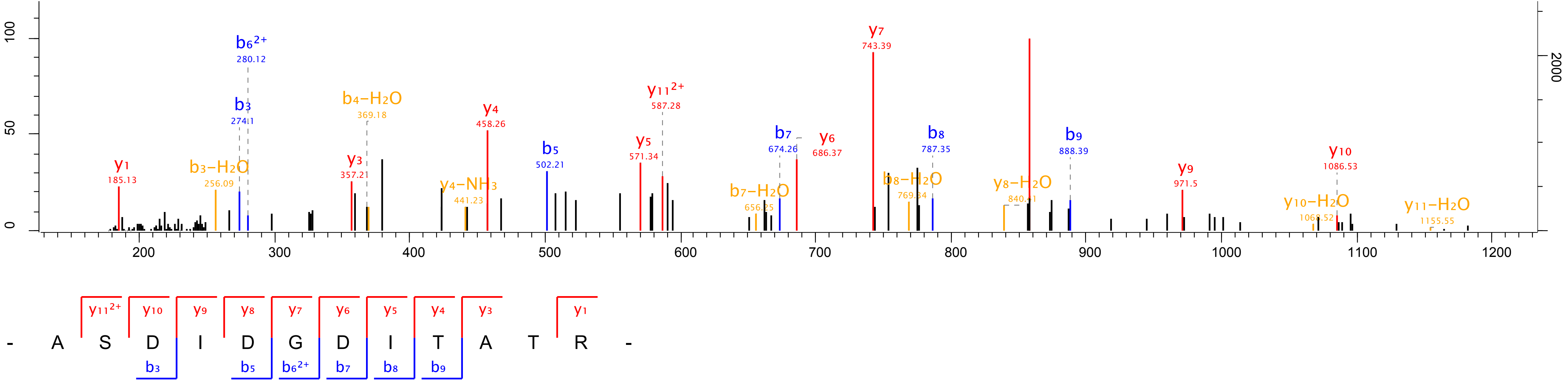

Raw file	Scan	Method	Score	m/z	Gene names
HBT_20130916_BV2_IL43_01	28831	ITMS; CID	94.45	950.01	Nedd1

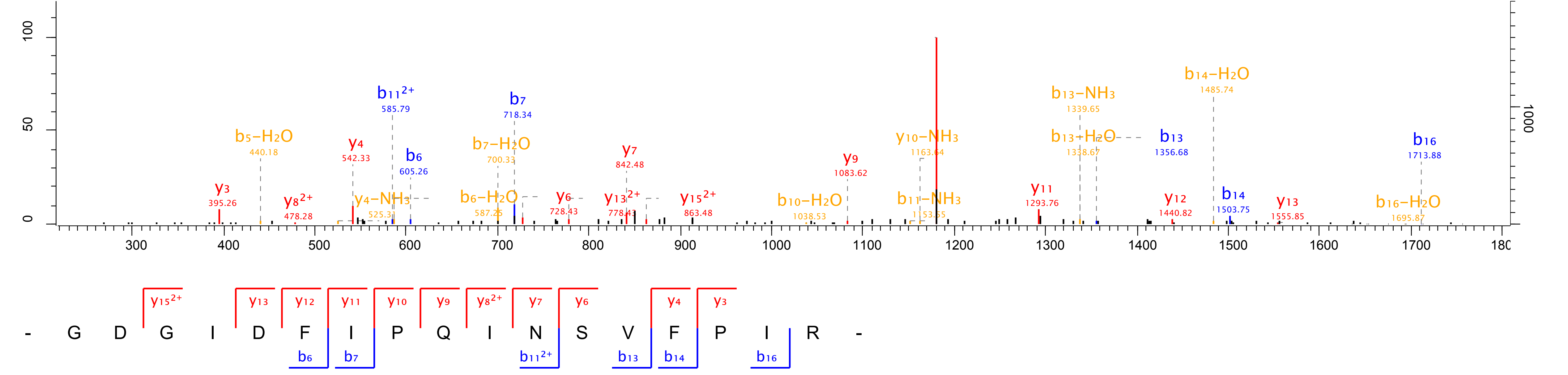

Raw file	Scan	Method	Score	m/z	Gene names
HBT_20130916_BV2_IL43_01	24617	ITMS; CID	176.32	691.41	Pmf1

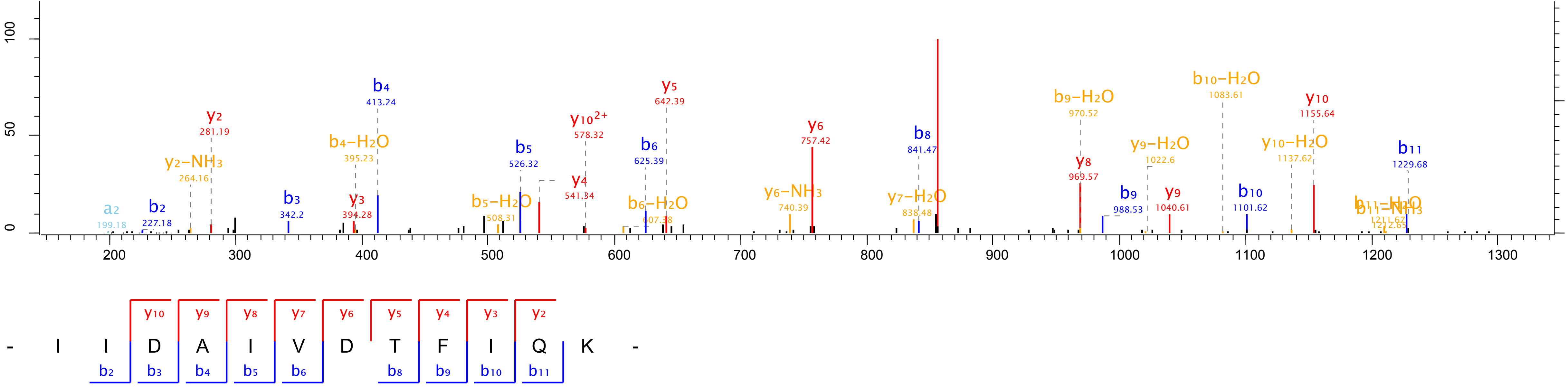

Raw file	Scan	Method	Score	m/z	Gene names
HBT_20130916_BV2_IL43_01	24359	ITMS; CID	119.92	1033.1	Agap3

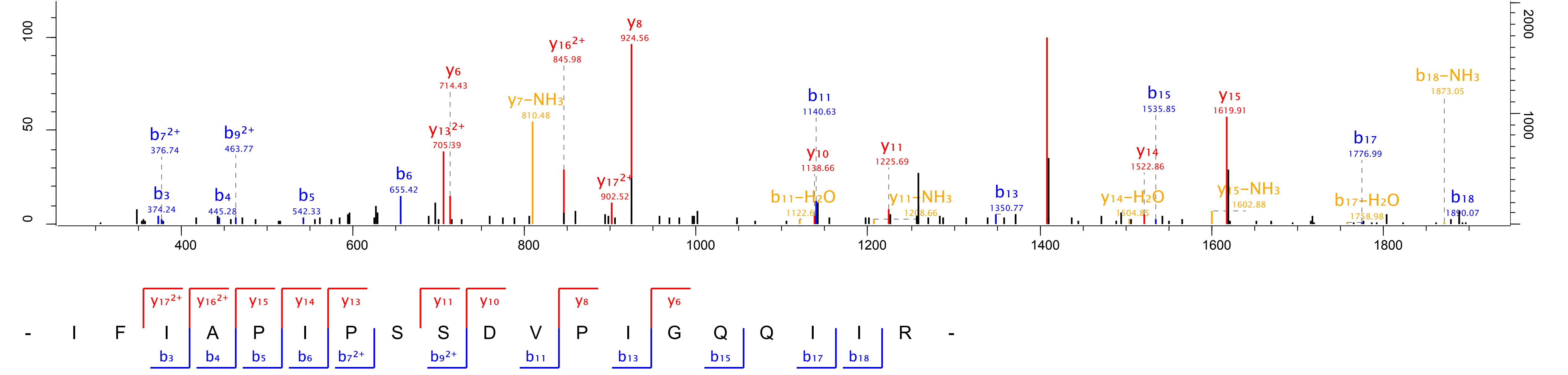

Raw file	Scan	Method	Score	m/z	Gene names
HBT_20130916_BV2_IL43_01	24266	ITMS; CID	89.01	1158.11	Pet112

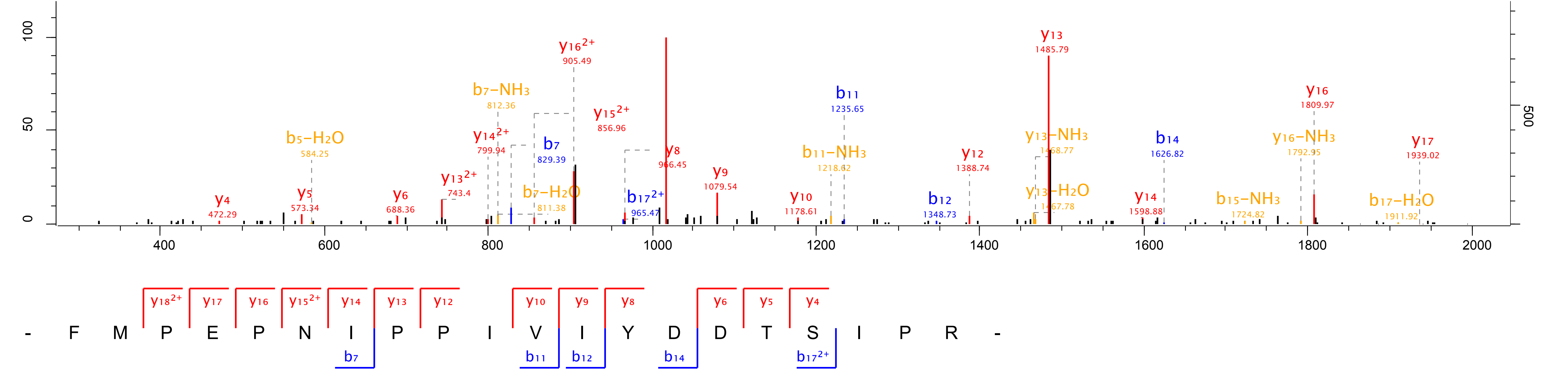

Raw file	Scan	Method	Score	m/z	Gene names
HBT_20130916_BV2_IL43_01	23824	ITMS; CID	139.64	716.4	Isg20l2

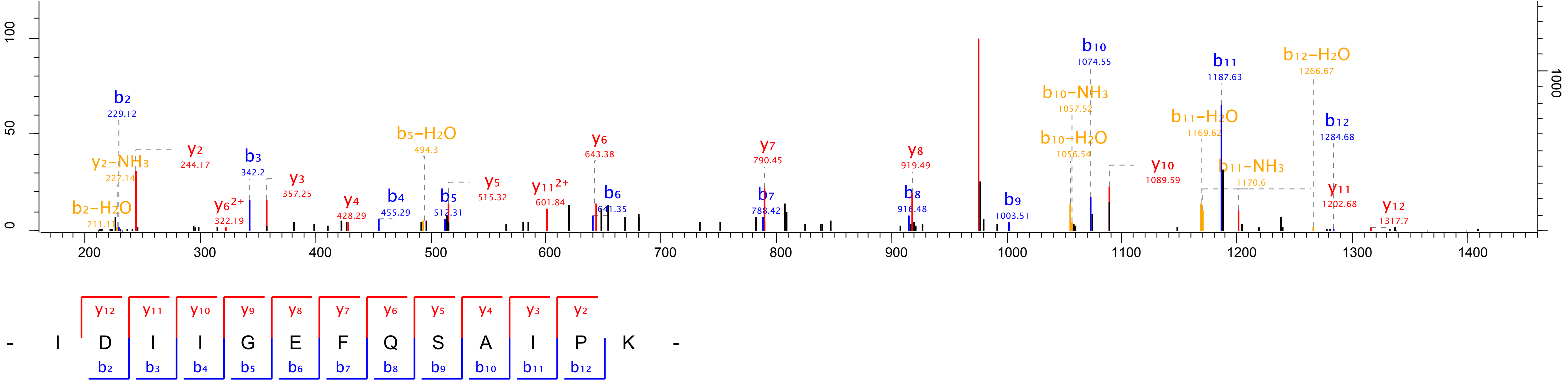

Raw file	Scan	Method	Score	m/z	Gene names
HBT_20130916_BV2_IL43_01	20888	ITMS; CID	104.21	916.04	Arfrp1

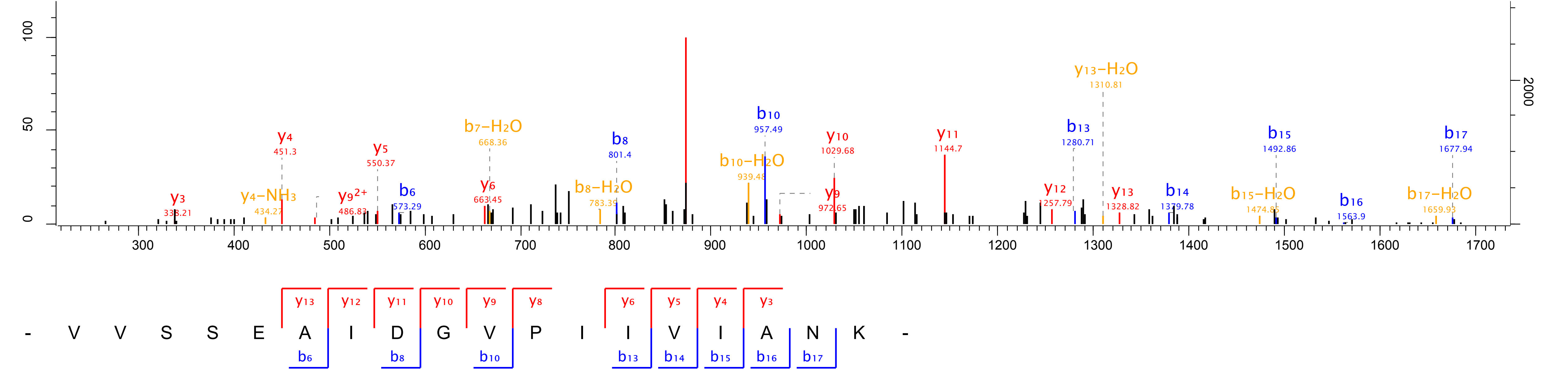

Raw file	Scan	Method	Score	m/z	Gene names
HBT_20130916_BV2_IL43_01	18910	ITMS; CID	200.93	647.35	Sfr1

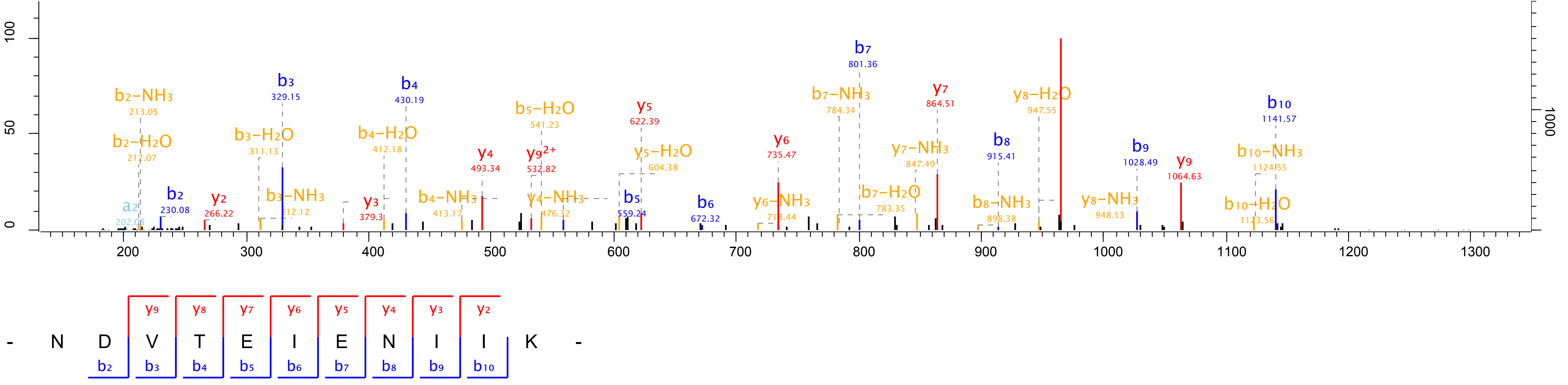

Raw file	Scan	Method	Score	m/z	Gene names
HBT_20130916_BV2_IL43_01	18875	ITMS; CID	58.33	1360.23	Syf2

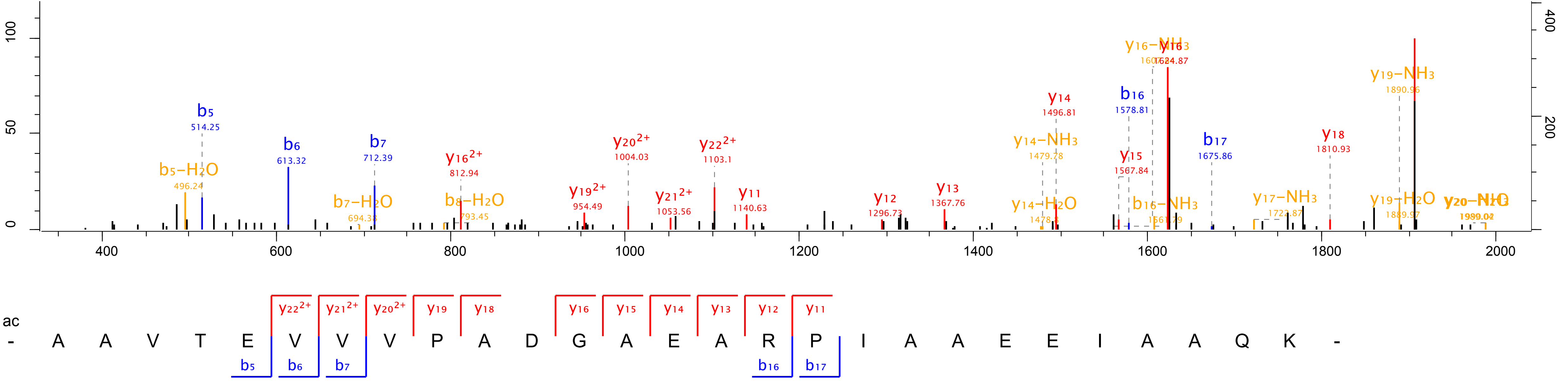

Raw file	Scan	Method	Score	m/z
HBT_20130916_BV2_IL43_01	17057	ITMS; CID	112.36	582.3

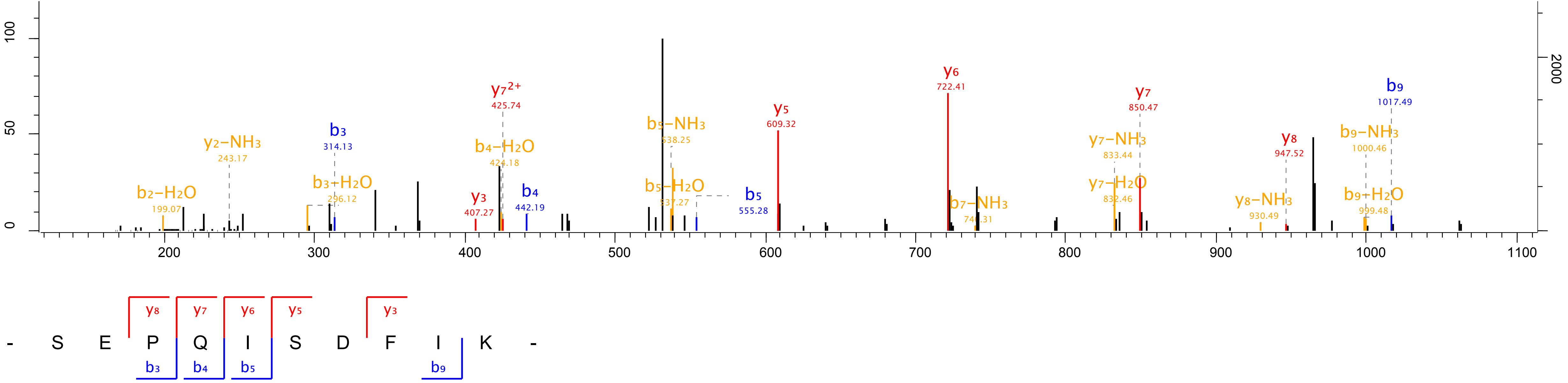

Raw file	Scan	Method	Score	m/z	Gene names
HBT_20130916_BV2_IL43_01	16145	ITMS; CID	162.88	568.29	mt-Cytb;Mt-Cyb

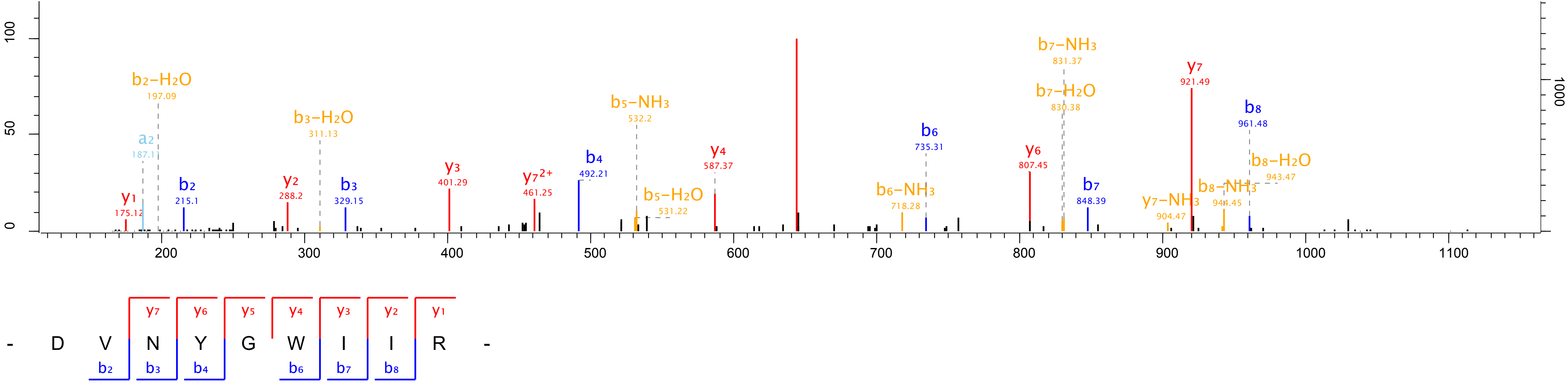

Raw file	Scan	Method	Score	m/z	Gene names
HBT_20130916_BV2_IL43_01	1493	ITMS; CID	99.5	642.82	Cnot11

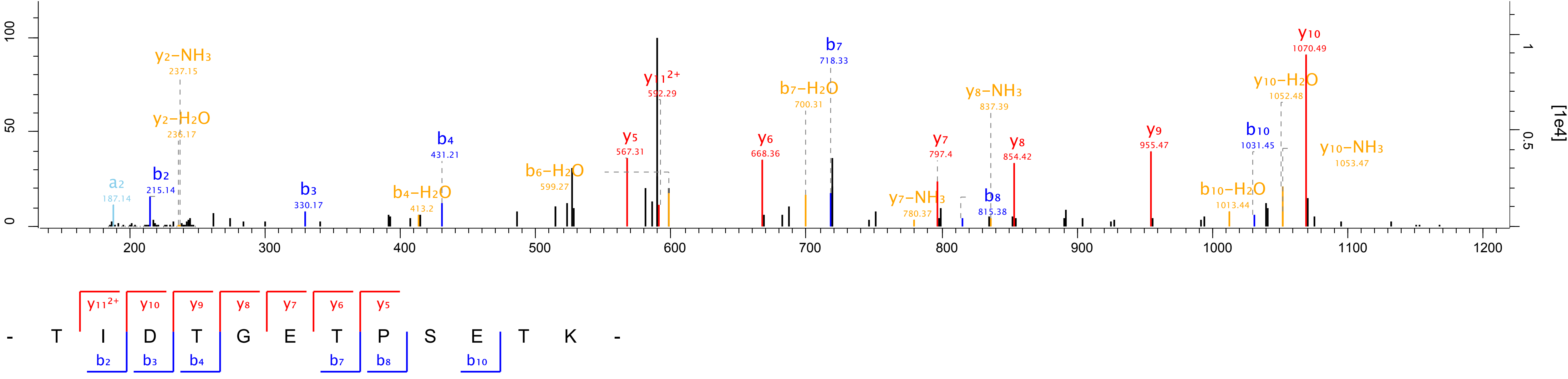

Raw file	Scan	Method	Score	m/z	Gene names
HBT_20130916_BV2_IL43_01	12675	ITMS; CID	108.25	824.44	Tax1bp3

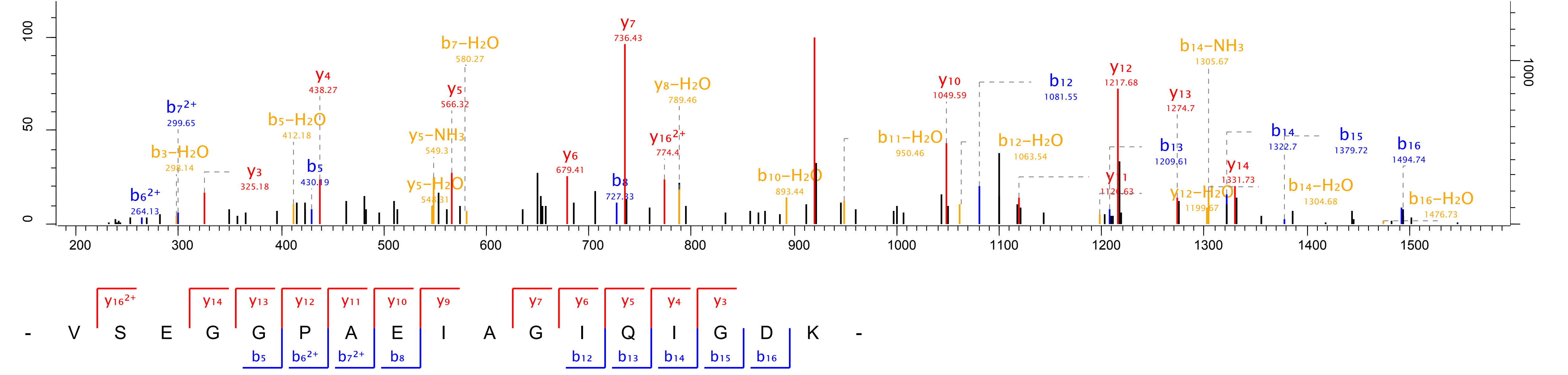

Raw file	Scan	Method	Score	m/z	Gene names
HBT_20130916_BV2_IL43_01	1228	ITMS; CID	124.21	630.77	Pja1

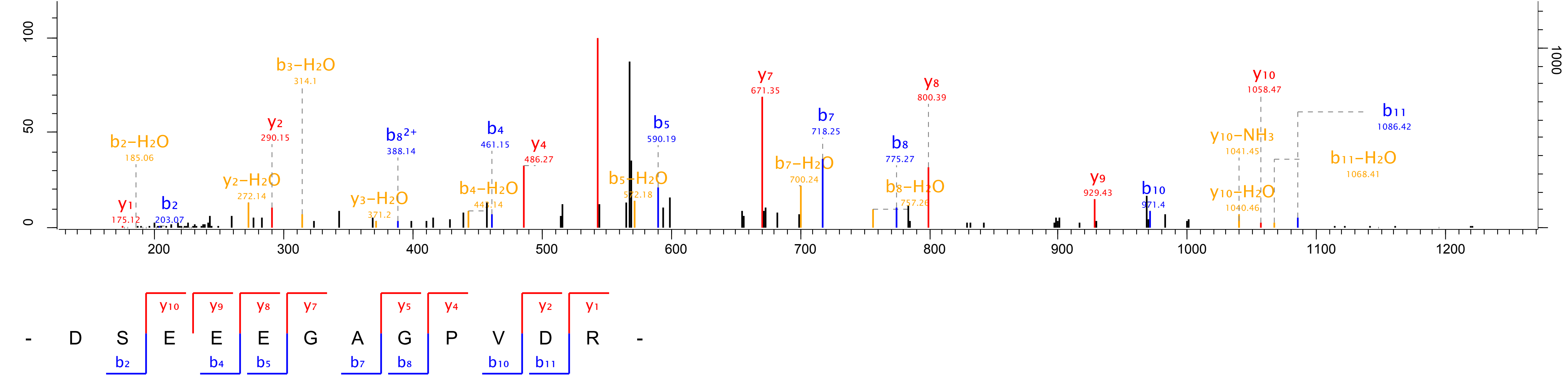

Raw file	Scan	Method	Score	m/z	Gene names
HBT_20130916_BV2_IL43_01	12148	ITMS; CID	127.49	1040.5	Zdhhc20

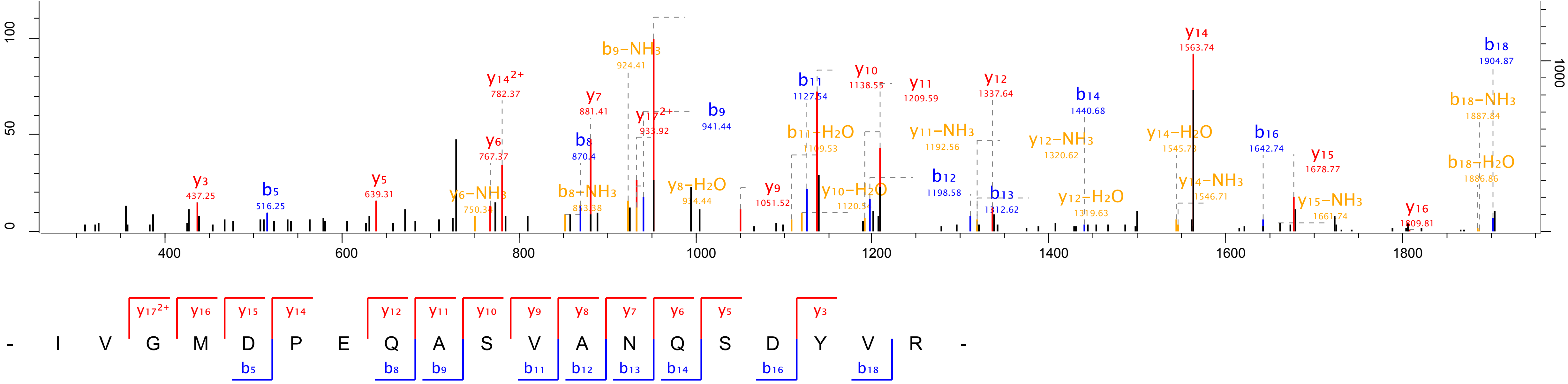

Raw file	Scan	Method	Score	m/z	Gene names
HBT_20130916_BV2_IL43_01	11534	ITMS; CID	152.97	553.82	Tmem14c

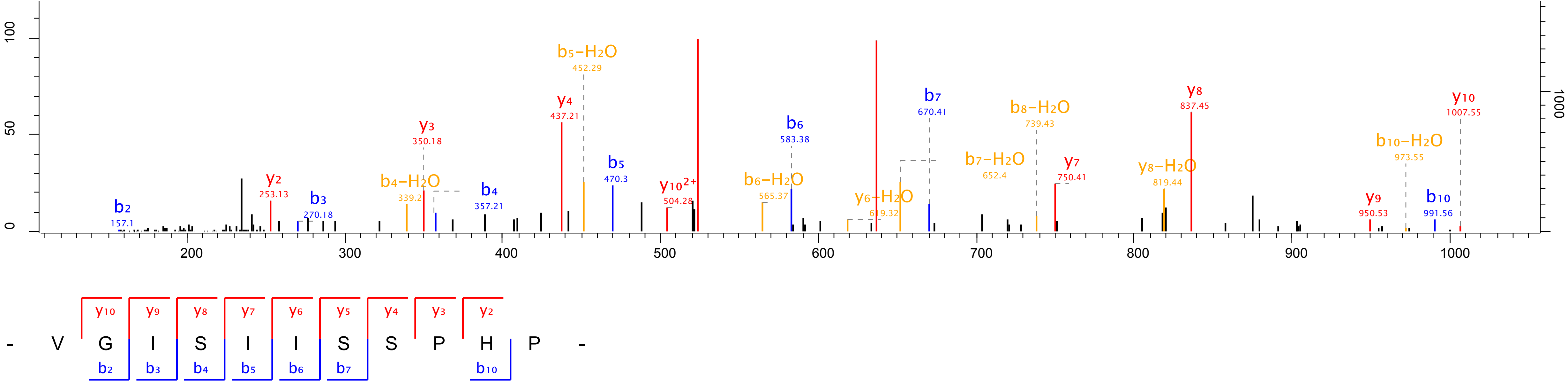

Raw file	Scan	Method	Score	m/z	Gene names
HBT_20130916_BV2_IL43_01	1118	ITMS; CID	170.26	729.32	Rnf214

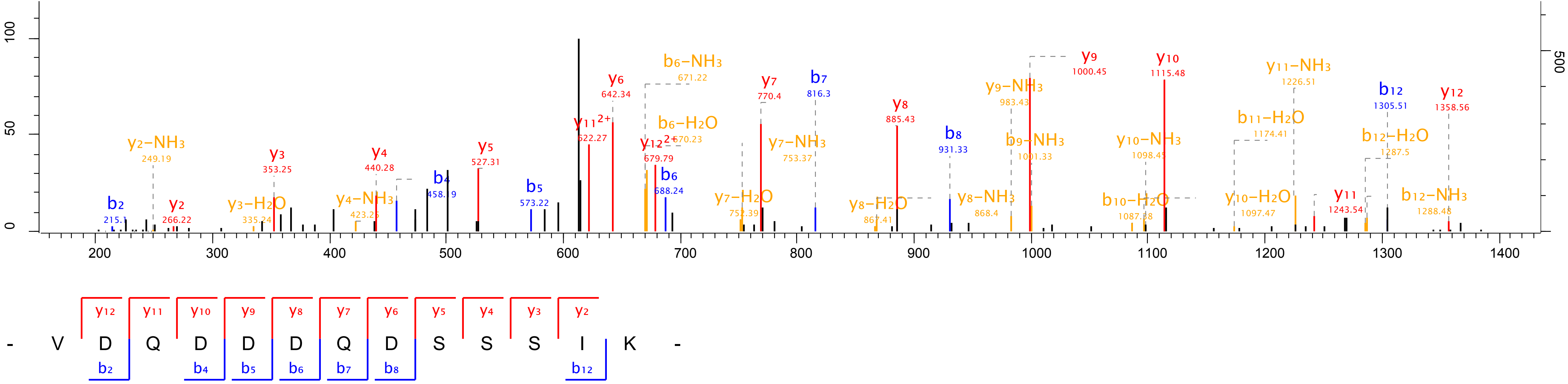

Raw file	Scan	Method	Score	m/z	Gene names
HBT_20130916_BV2_IL43_01	11128	ITMS; CID	124.5	852.38	Sp1

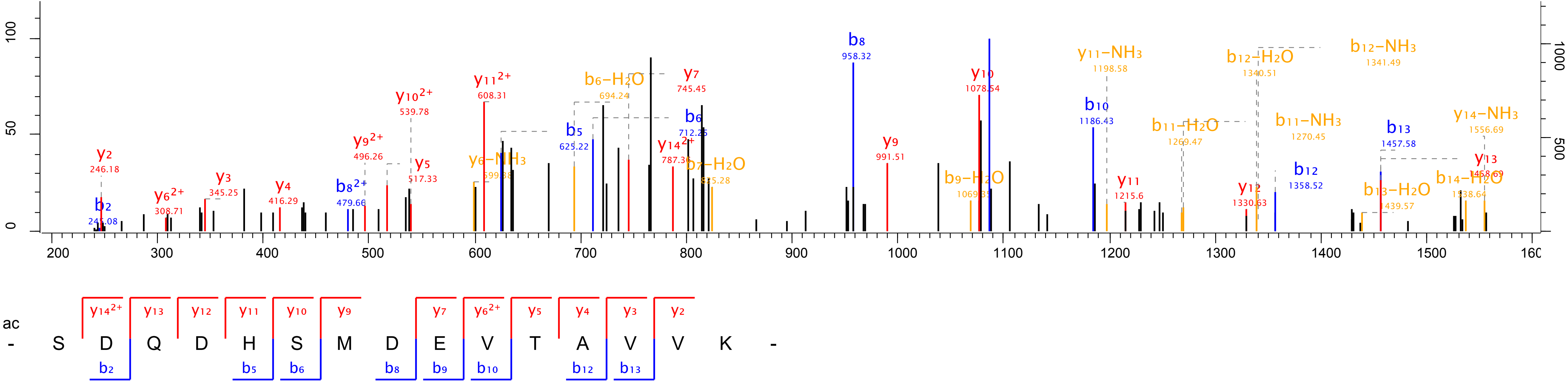

Raw file	Scan	Method	Score	m/z	Gene names
HBT_20130916_BV2_IL42_06	20576	ITMS; CID	92.94	727.32	Rsl24d1

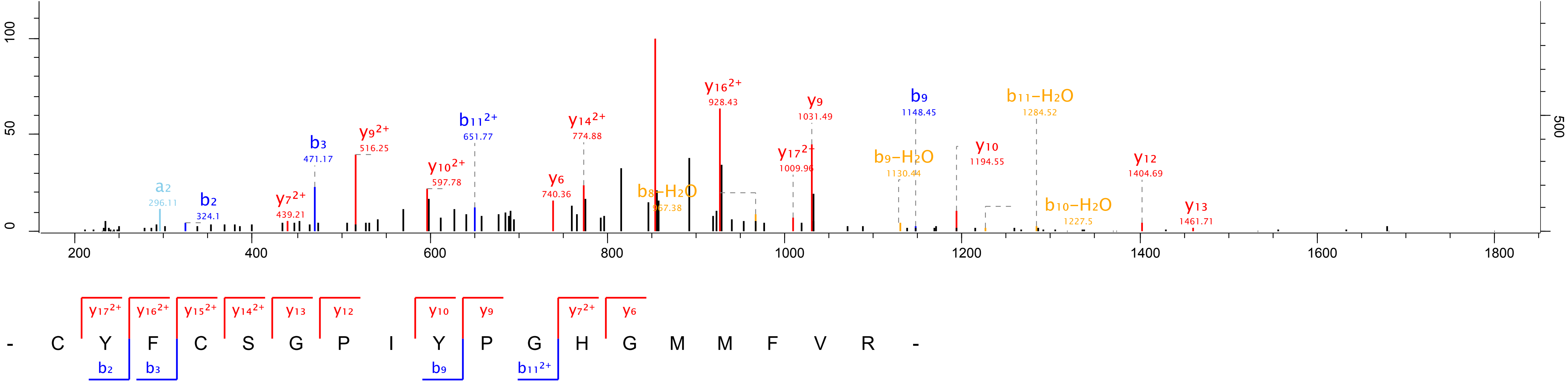

Raw file	Scan	Method	Score	m/z	Gene names
HBT_20130916_BV2_IL42_06	13037	ITMS; CID	107.79	404.93	Mettl2

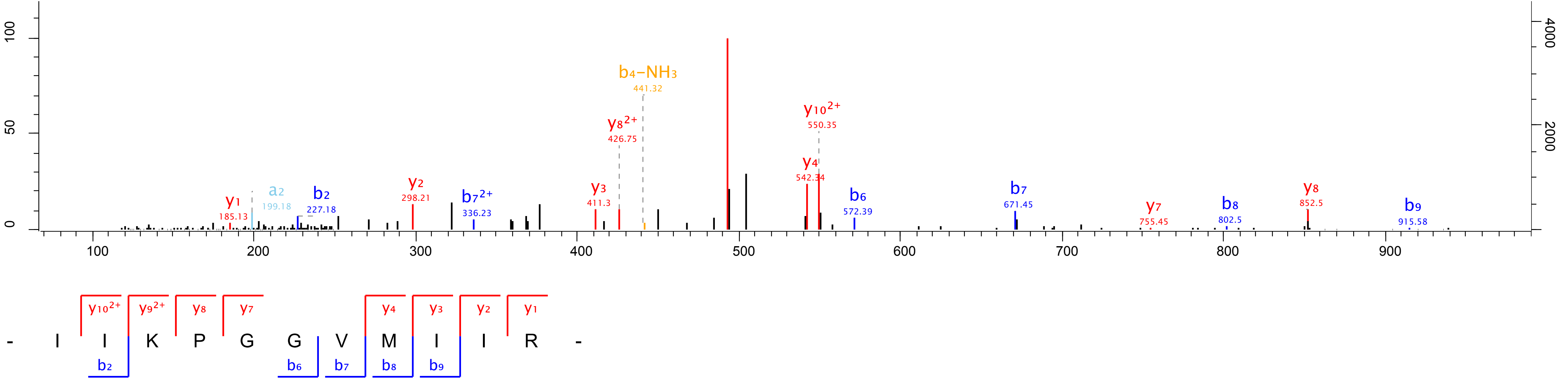

Raw file	Scan	Method	Score	m/z
HBT_20130916_BV2_IL42_05	8980	ITMS; CID	98.16	636.37

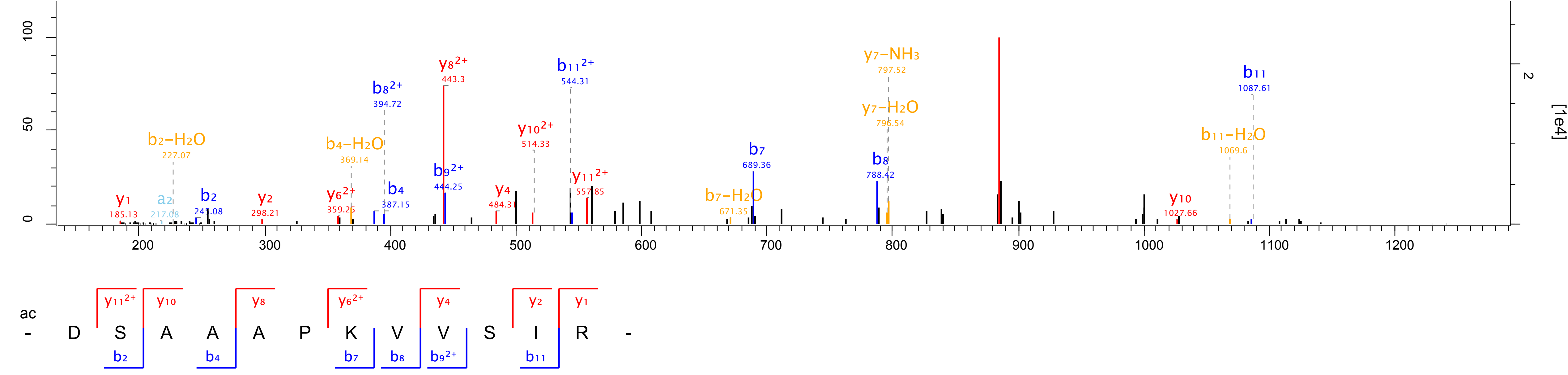

Raw file	Scan	Method	Score	m/z	Gene names
HBT_20130916_BV2_IL42_05	30275	ITMS; CID	130.64	742.74	Clk3

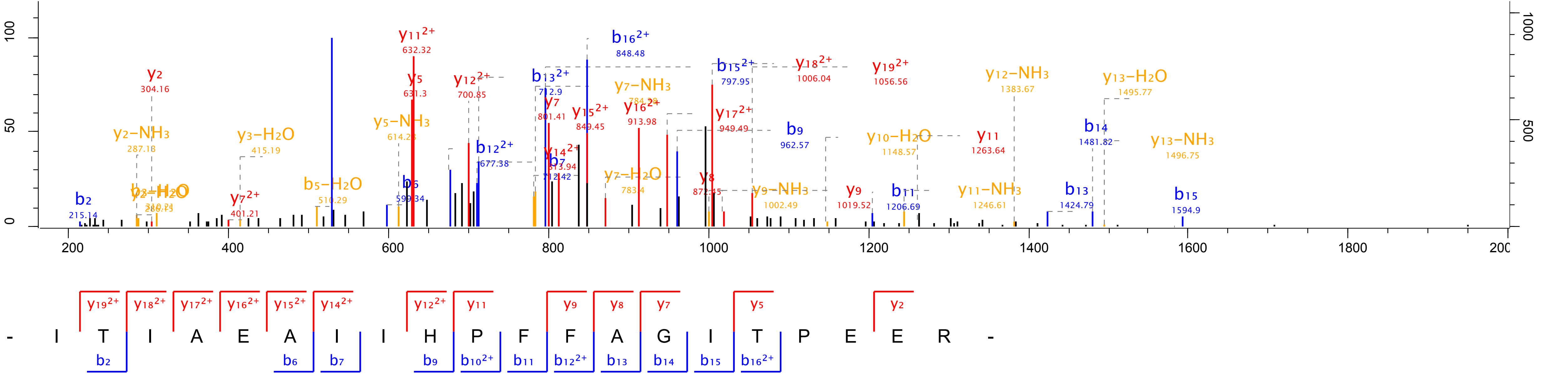

Raw file	Scan	Method	Score	m/z	Gene names
HBT_20130916_BV2_IL42_05	28071	ITMS; CID	60.76	773.79	Atp13a2

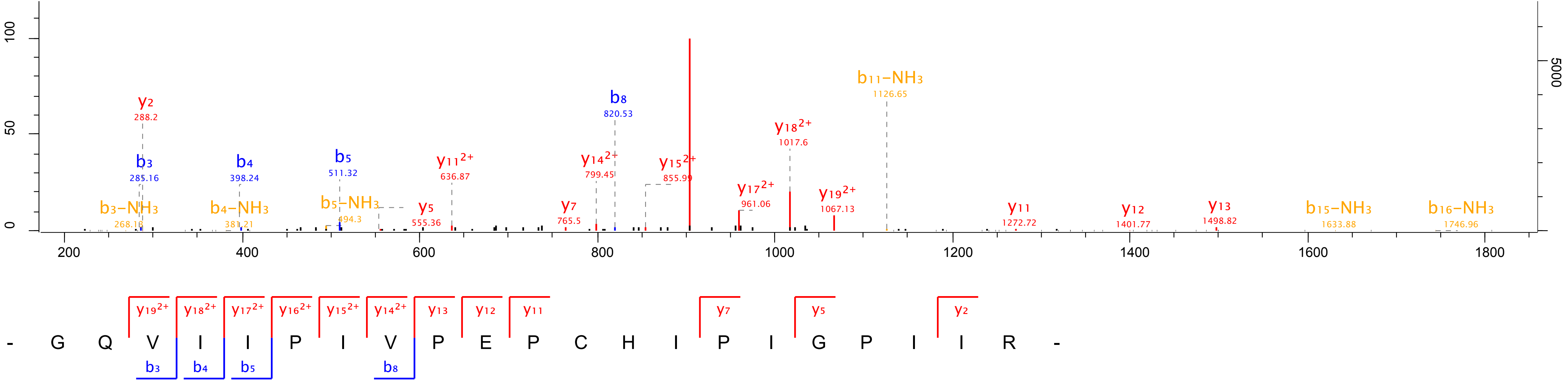

Raw file	Scan	Method	Score	m/z	Gene names
HBT_20130916_BV2_IL42_05	28015	ITMS; CID	97.38	800.47	Hps6

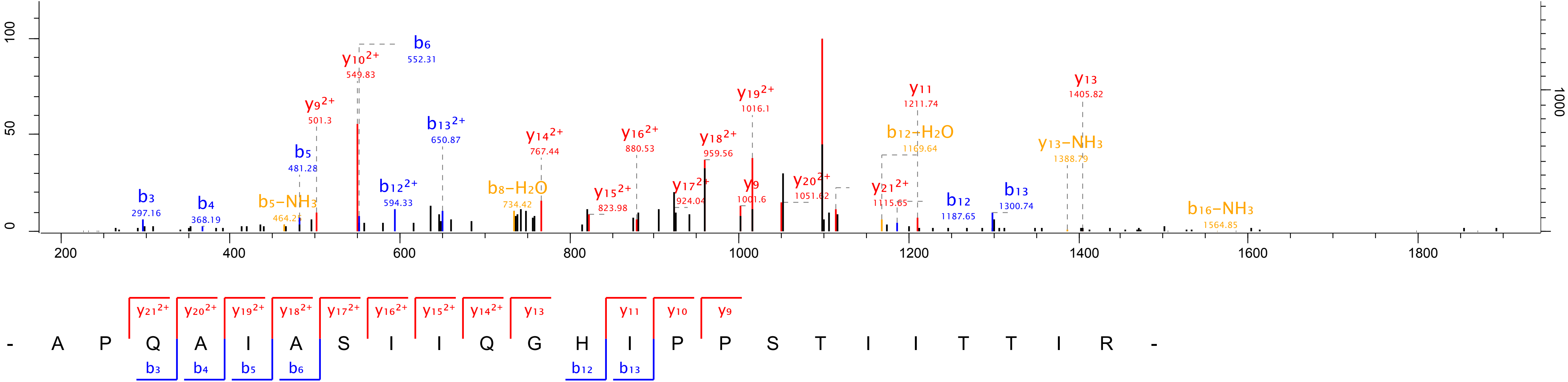

Raw file	Scan	Method	Score	m/z
HBT_20130916_BV2_IL42_05	24873	ITMS; CID	91.59	744.1

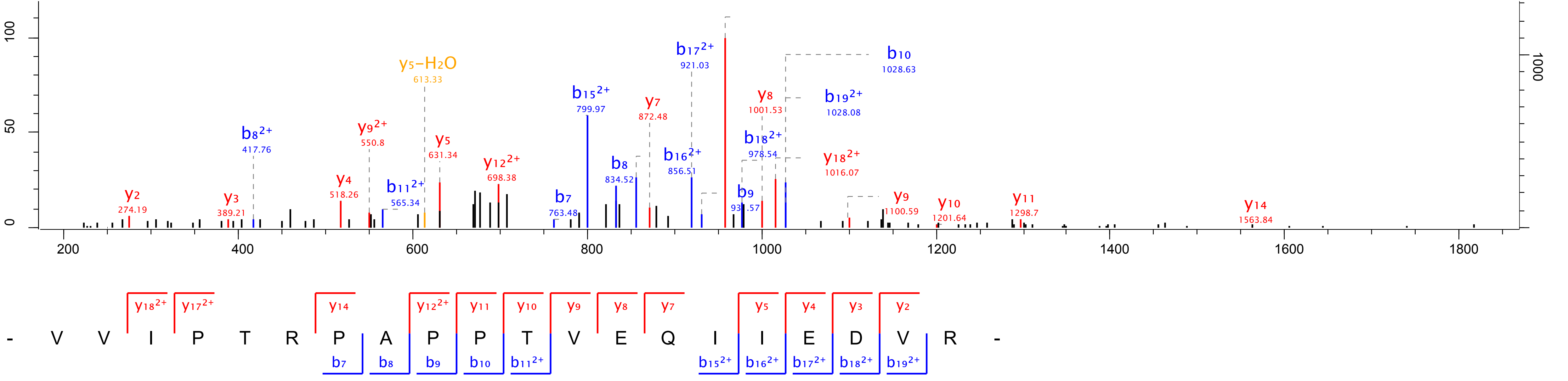

Raw file	Scan	Method	Score	m/z	Gene names
HBT_20130916_BV2_IL42_05	17506	ITMS; CID	62.06	1002.04	Crybb1

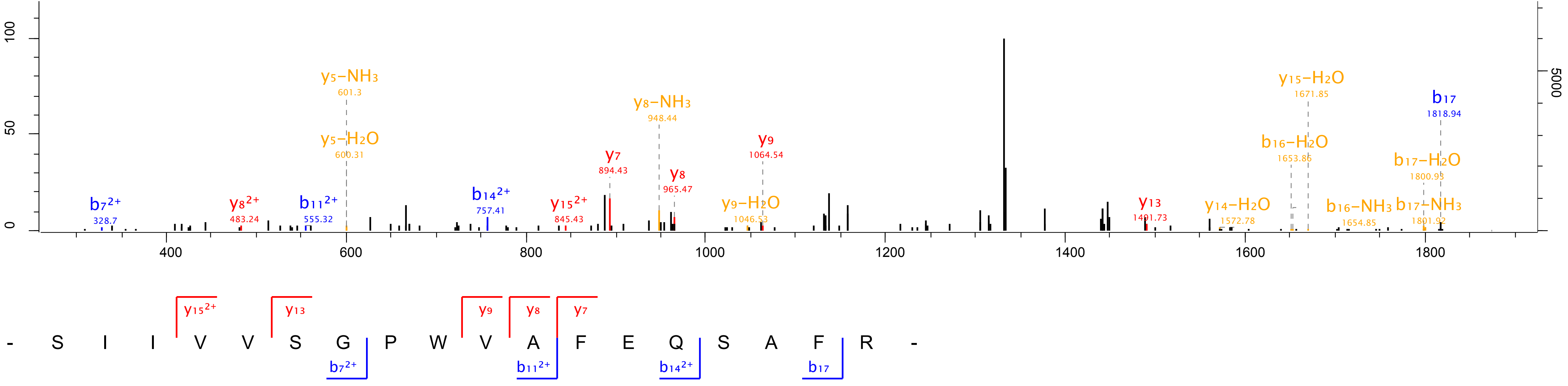

Raw file	Scan	Method	Score	m/z	Gene names
HBT_20130916_BV2_IL42_04	26253	ITMS; CID	68.42	938.83	Gpn1

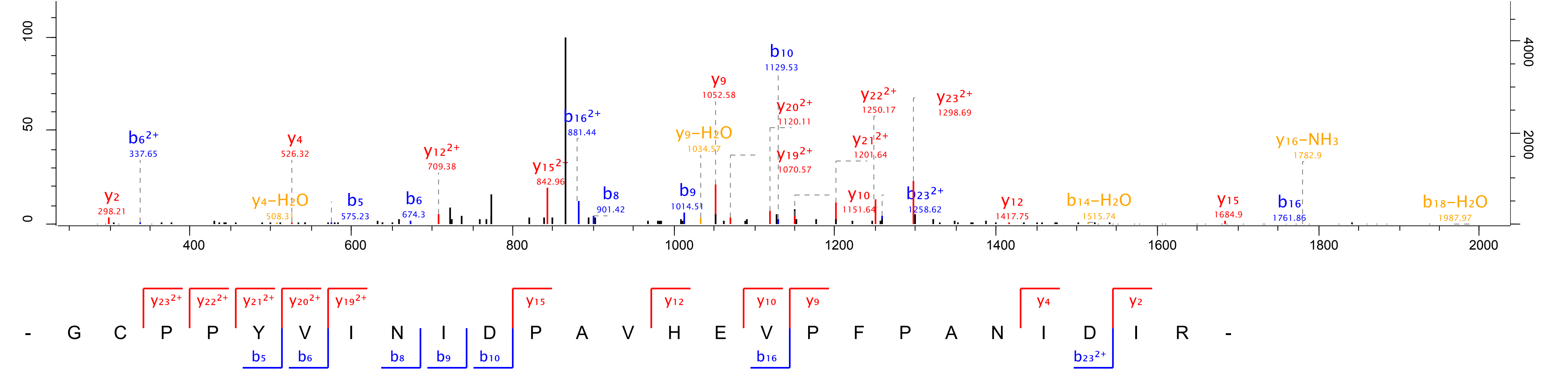

Raw file	Scan	Method	Score	m/z
HBT_20130916_BV2_IL42_04	22642	ITMS; CID	54.77	675.37

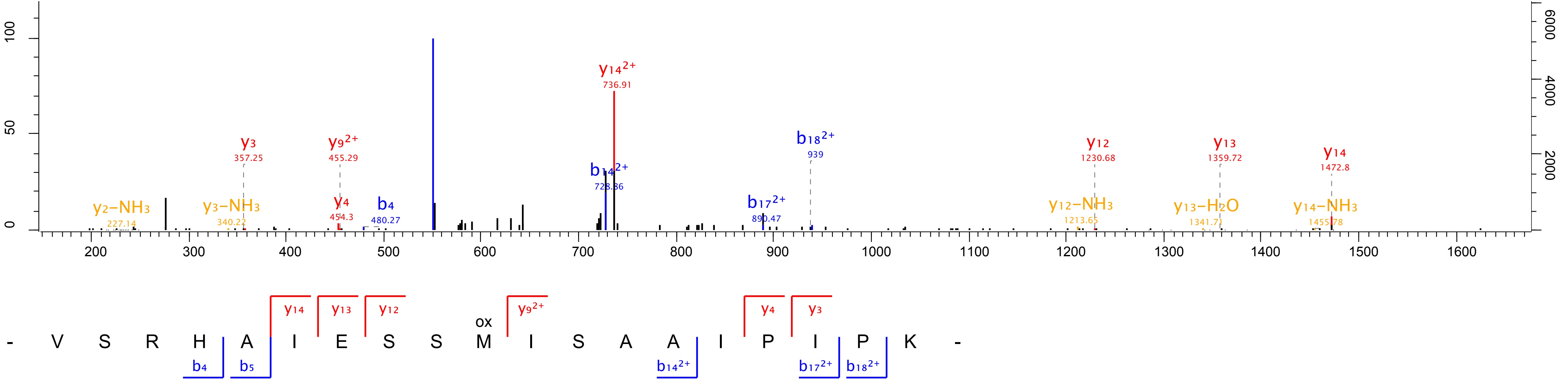

Raw file	Scan	Method	Score	m/z	Gene names
HBT_20130916_BV2_IL42_04	17597	ITMS; CID	165.35	731.33	Tmem147

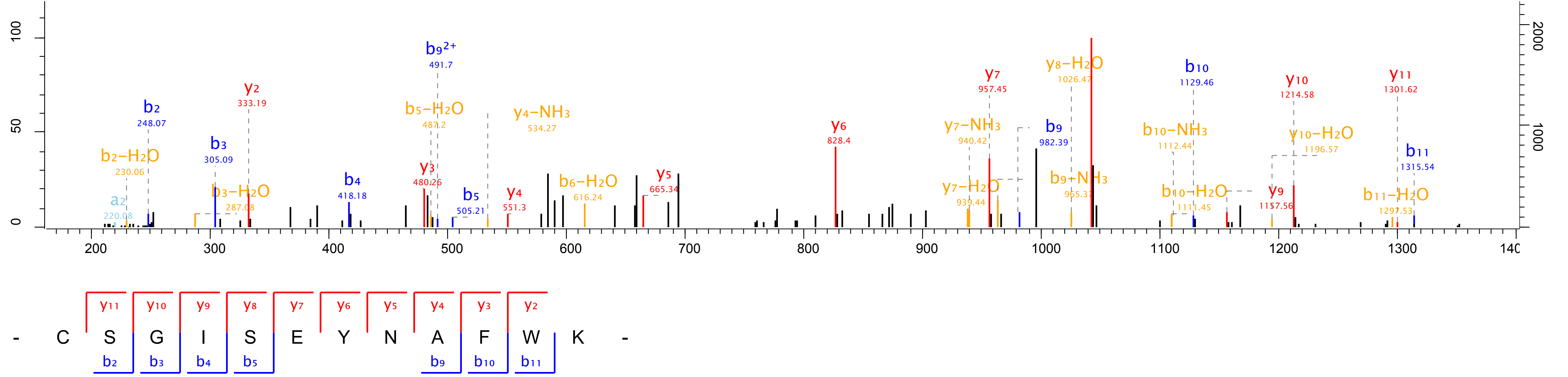

Raw file	Scan	Method	Score	m/z	Gene names
HBT_20130916_BV2_IL42_04	13425	ITMS; CID	96.11	590.79	Lrif1

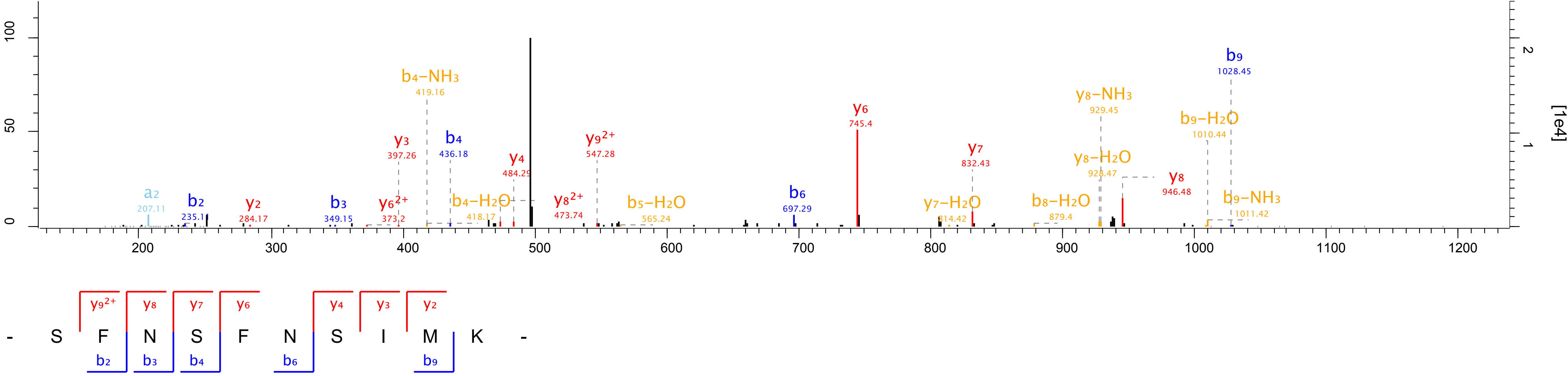

Raw file	Scan	Method	Score	m/z	Gene names
HBT_20130916_BV2_IL42_03	9021	ITMS; CID	80.87	724.38	Adat2

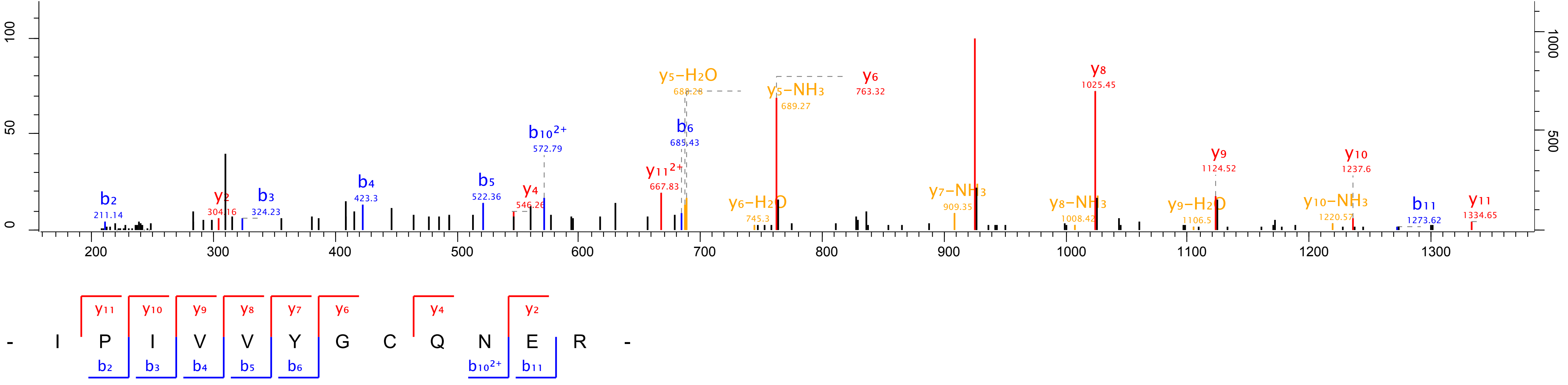

Raw file	Scan	Method	Score	m/z	Gene names
HBT_20130916_BV2_IL42_03	846	ITMS; CID	145.16	423.74	Tcam1

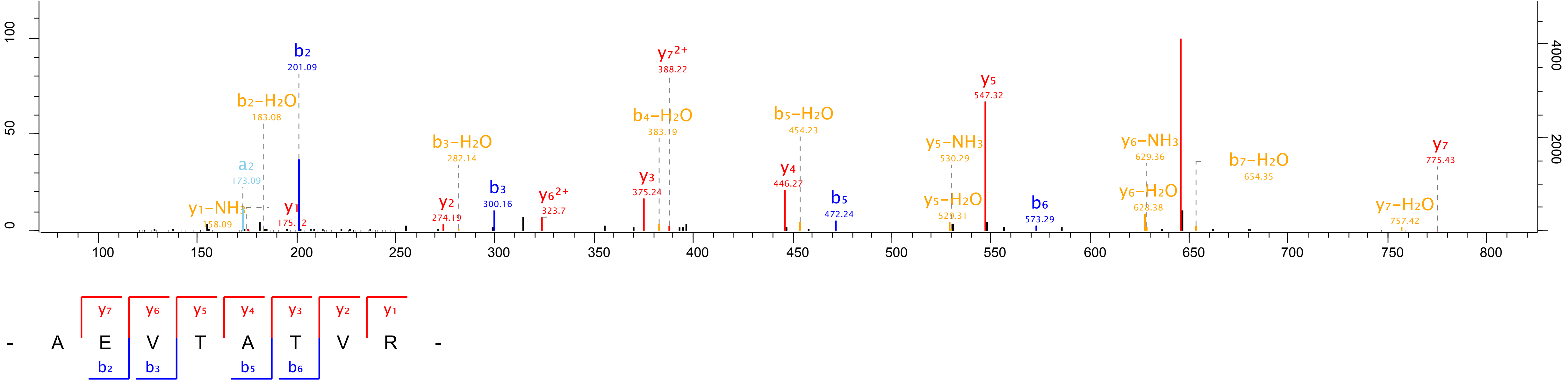

Raw file	Scan	Method	Score	m/z	Gene names
HBT_20130916_BV2_IL42_03	8138	ITMS; CID	119.87	698.38	Alg3

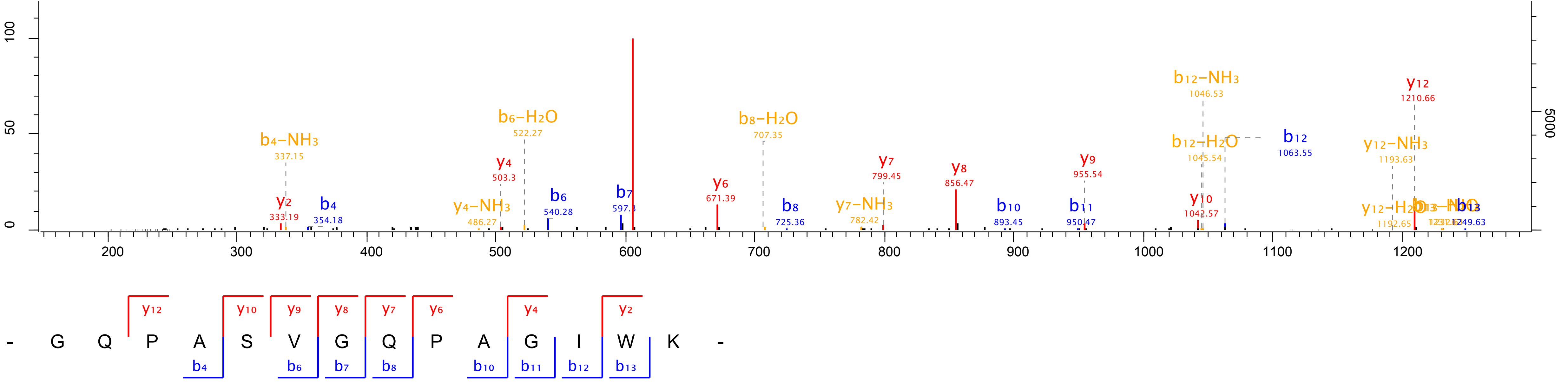

Raw file	Scan	Method	Score	m/z	Gene names
HBT_20130916_BV2_IL42_03	26084	ITMS; CID	76.01	693.4	Slc25a26

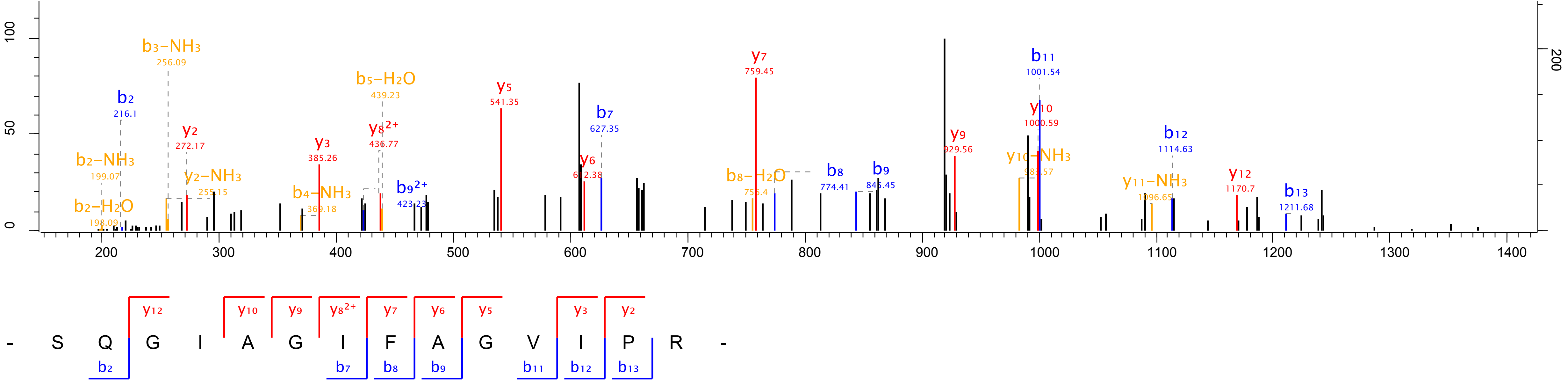

Raw file	Scan	Method	Score	m/z	Gene names
HBT_20130916_BV2_IL42_03	25602	ITMS; CID	107.45	623.33	Il17rb

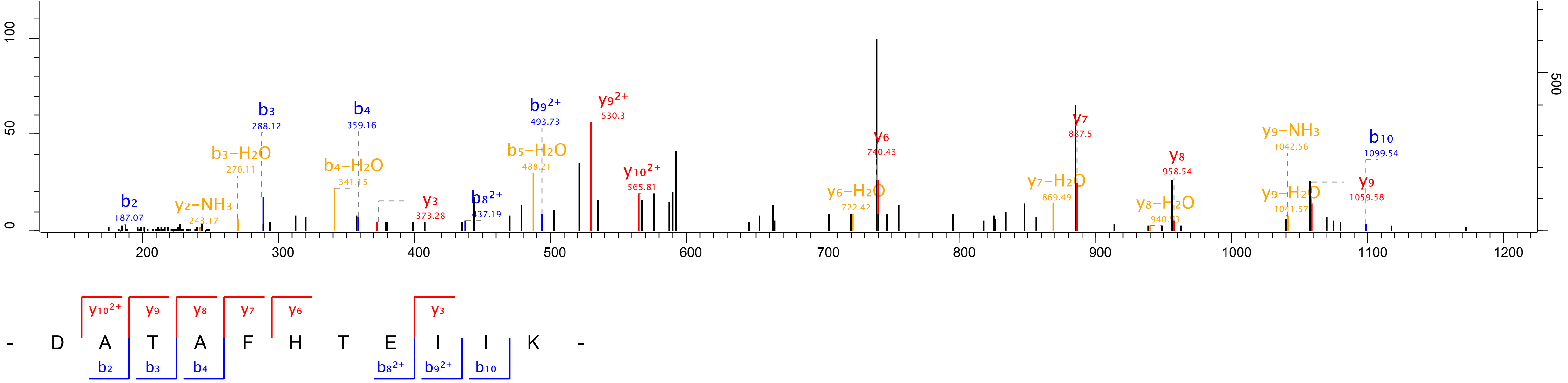

Raw file	Scan	Method	Score	m/z	Gene names
HBT_20130916_BV2_IL42_03	24723	ITMS; CID	143.7	590.89	Tbc1d1

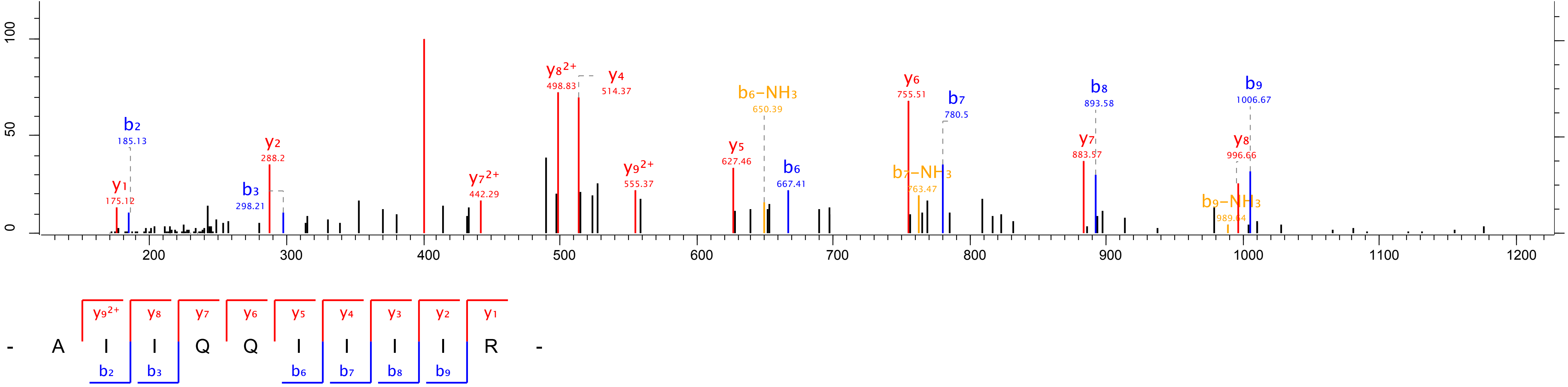

Raw file	Scan	Method	Score	m/z	Gene names
HBT_20130916_BV2_IL42_03	24393	ITMS; CID	189.38	580.82	Med30

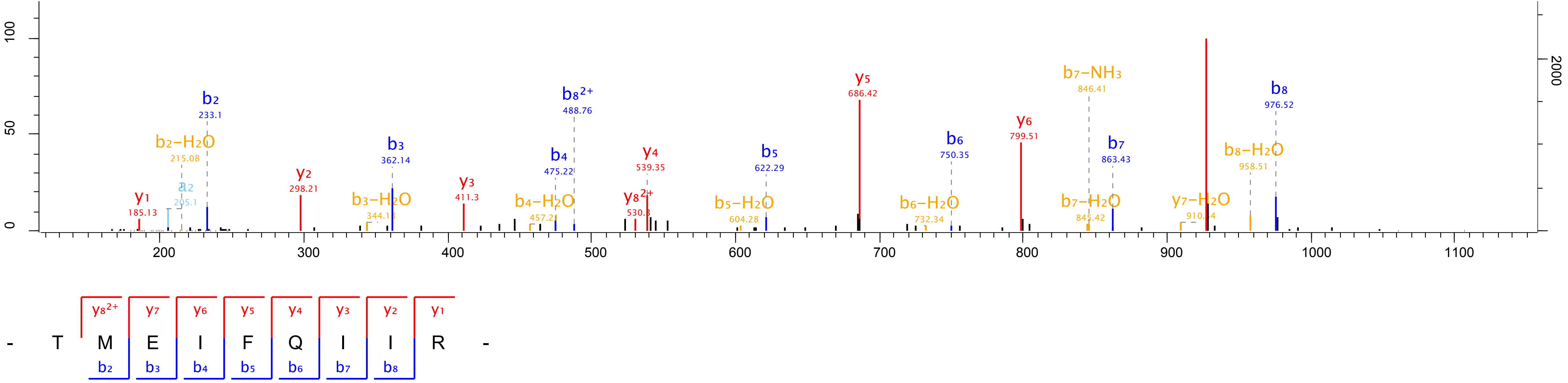

Raw file	Scan	Method	Score	m/z	Gene names
HBT_20130916_BV2_IL42_03	24212	ITMS; CID	83.88	872.96	Sorbs1

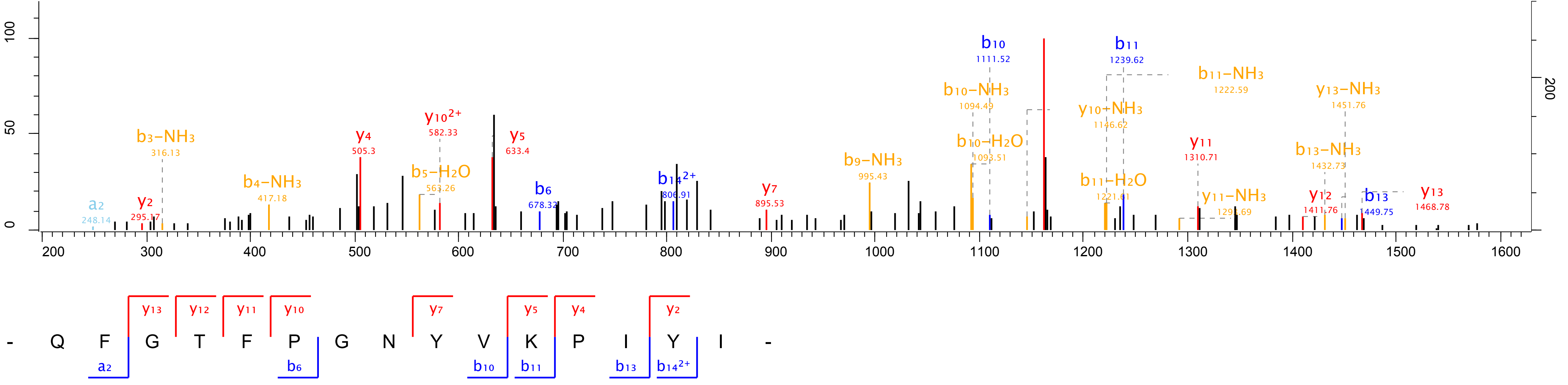

Raw file	Scan	Method	Score	m/z
HBT_20130916_BV2_IL42_03	23203	ITMS; CID	86.46	777.39

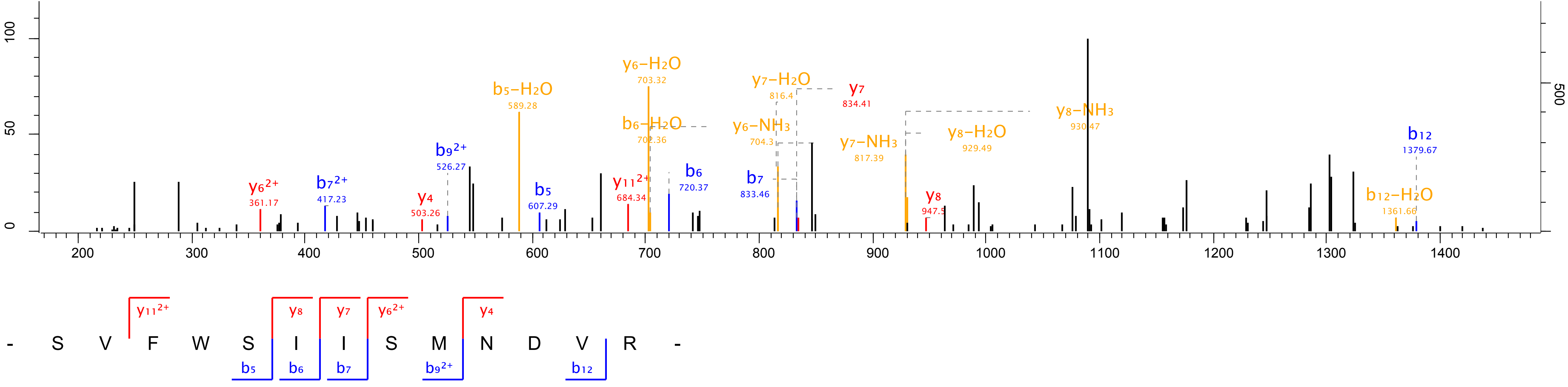

Raw file	Scan	Method	Score	m/z	Gene names
HBT_20130916_BV2_IL42_03	20463	ITMS; CID	122.18	531.82	Arl6

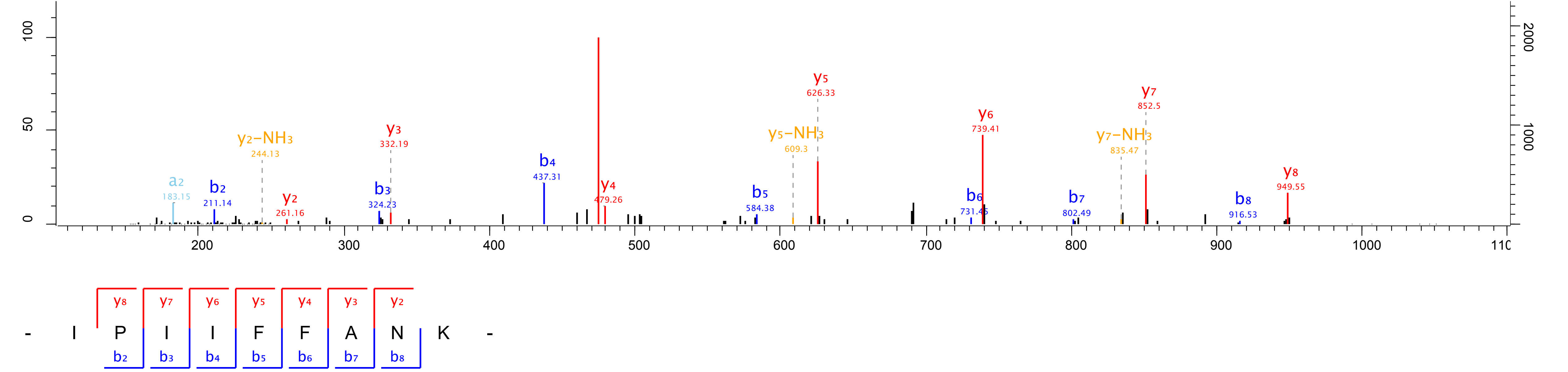

Raw file	Scan	Method	Score	m/z	Gene names
HBT_20130916_BV2_IL42_03	19704	ITMS; CID	80.96	884.91	Havcr2

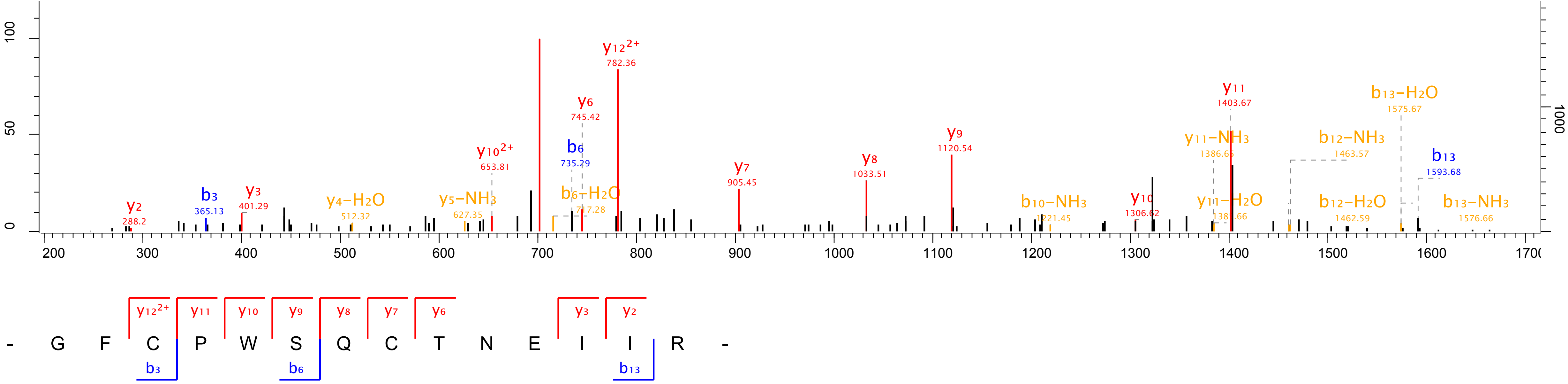

Raw file	Scan	Method	Score	m/z	Gene names
HBT_20130916_BV2_IL42_03	18359	ITMS; CID	141.73	685.89	Entpd6

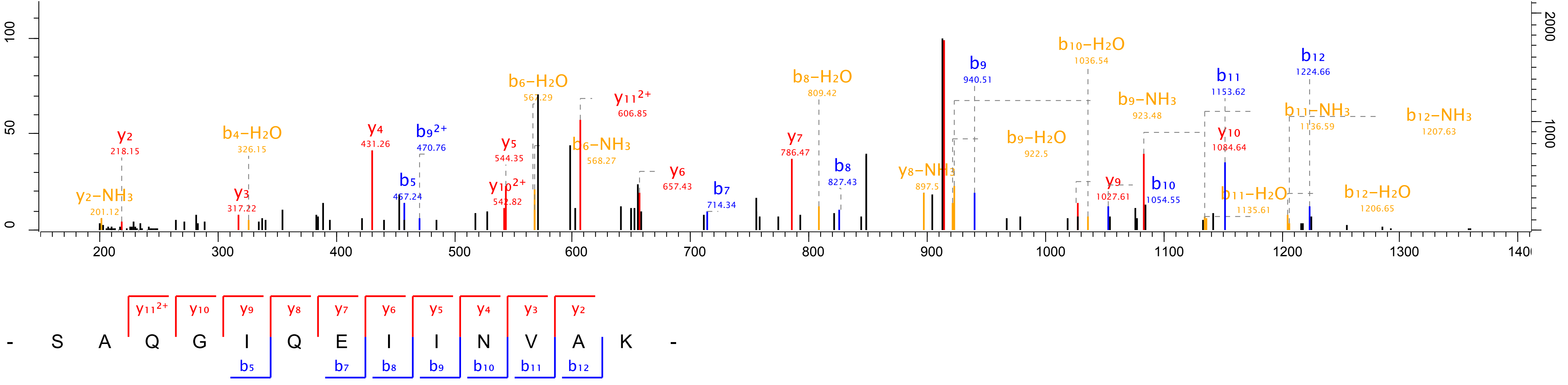

Raw file	Scan	Method	Score	m/z	Gene names
HBT_20130916_BV2_IL42_03	14937	ITMS; CID	136.27	759.88	Tgfb1

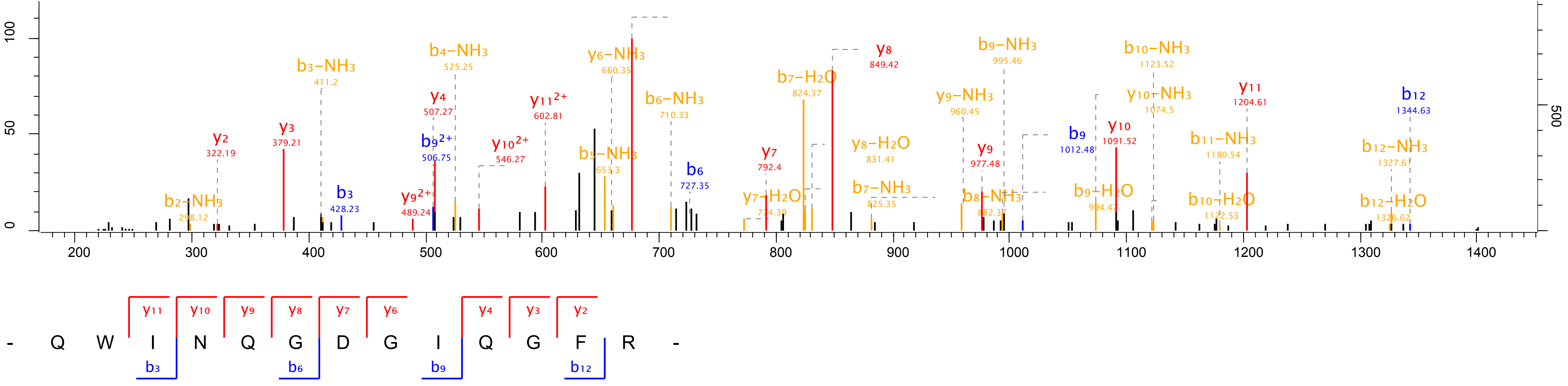

Raw file	Scan	Method	Score	m/z	Gene names
HBT_20130916_BV2_IL42_02	28306	ITMS; CID	109.07	857.95	Fbxo33

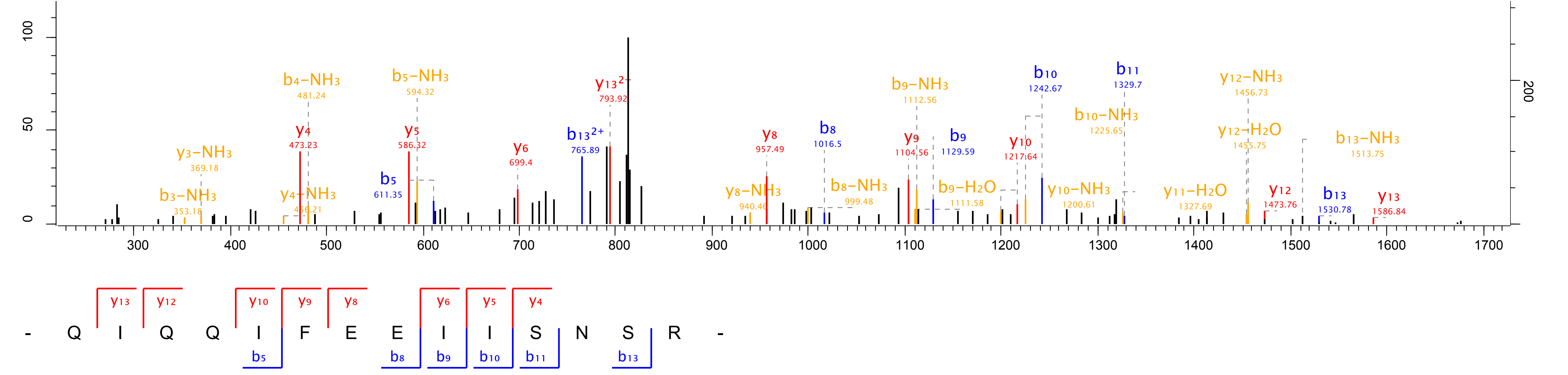

Raw file	Scan	Method	Score	m/z	Gene names
HBT_20130916_BV2_IL42_02	28129	ITMS; CID	118.61	648.4	Ect2

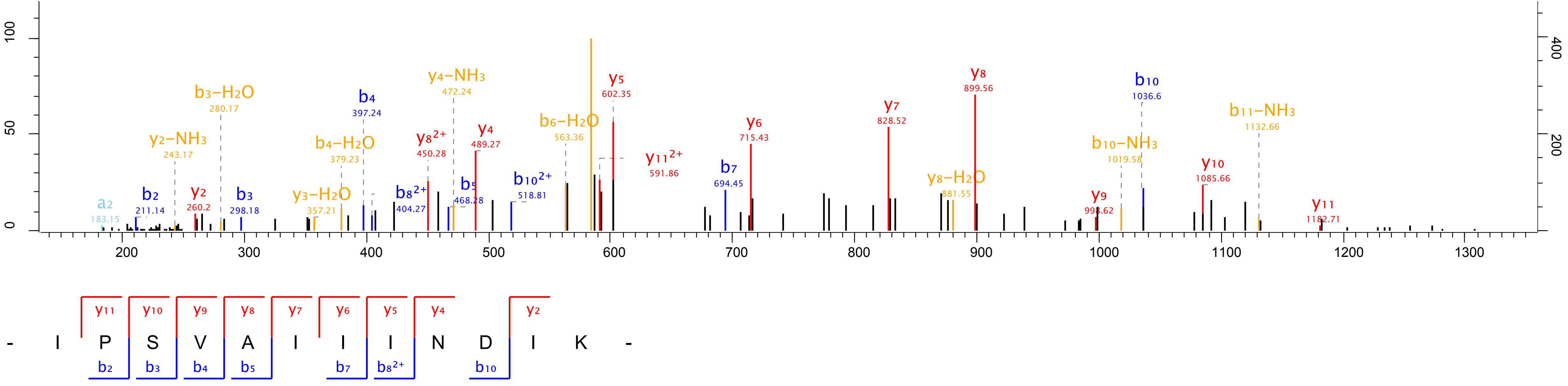

Raw file	Scan	Method	Score	m/z
HBT_20130916_BV2_IL42_02	27904	ITMS; CID	75.91	856.47

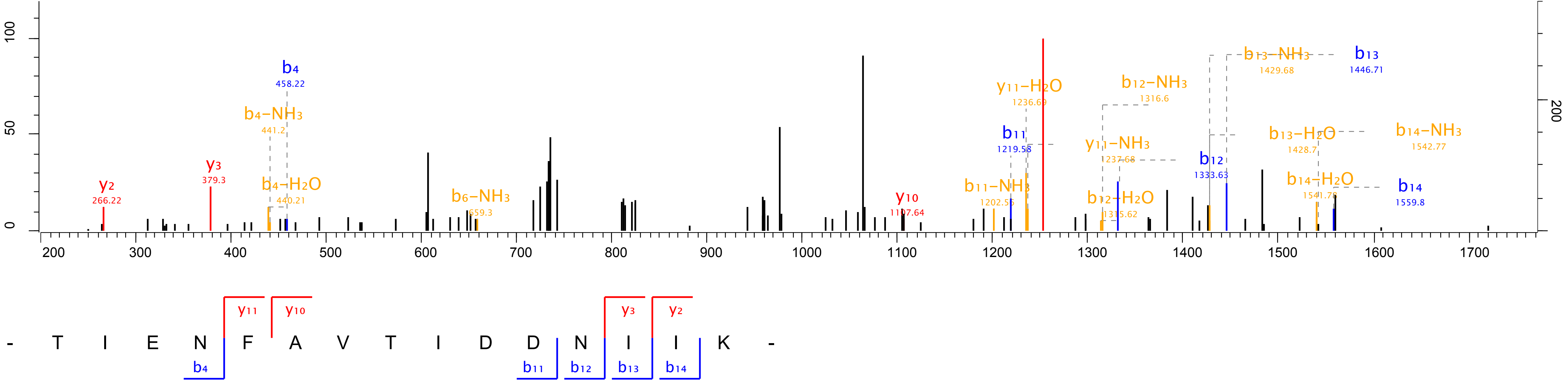

Raw file	Scan	Method	Score	m/z	Gene names
HBT_20130916_BV2_IL42_02	27618	ITMS; CID	125.47	790.42	Haus3

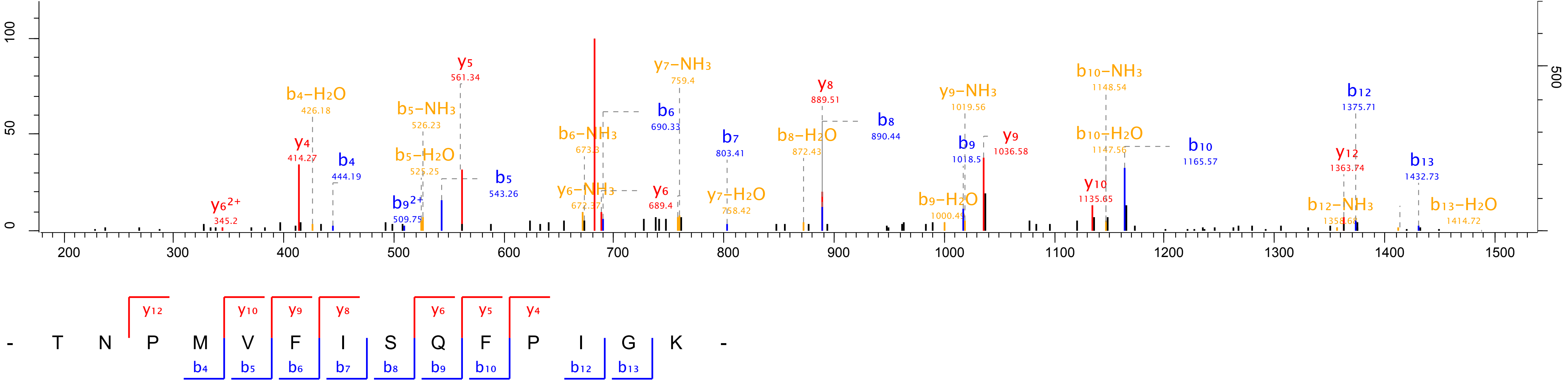

Raw file	Scan	Method	Score	m/z	Gene names
HBT_20130916_BV2_IL42_02	24119	ITMS; CID	90.05	744.42	Etv3

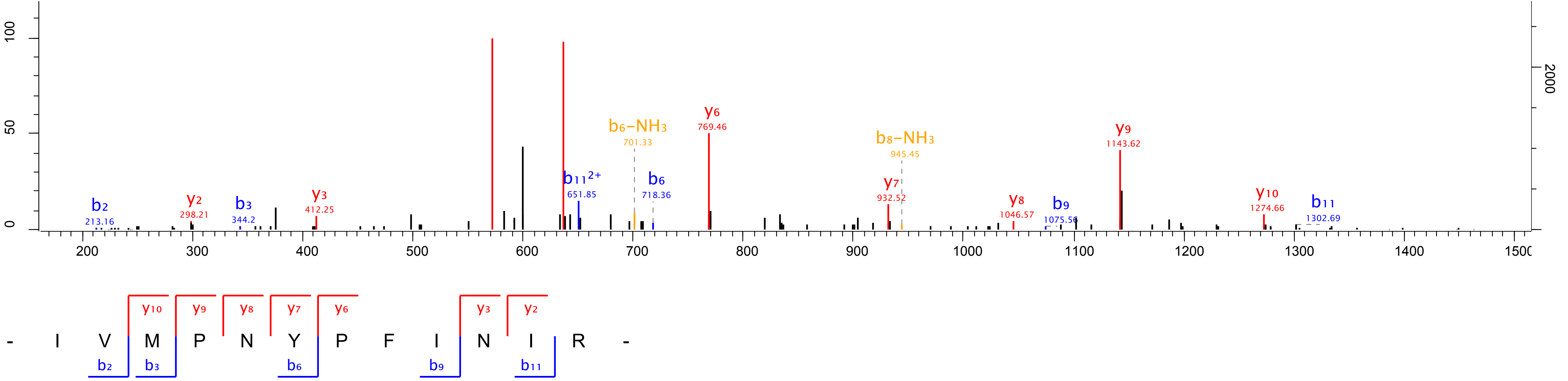

Raw file	Scan	Method	Score	m/z	Gene names
HBT_20130916_BV2_IL42_02	22912	ITMS; CID	186.56	819.94	Ptdss1

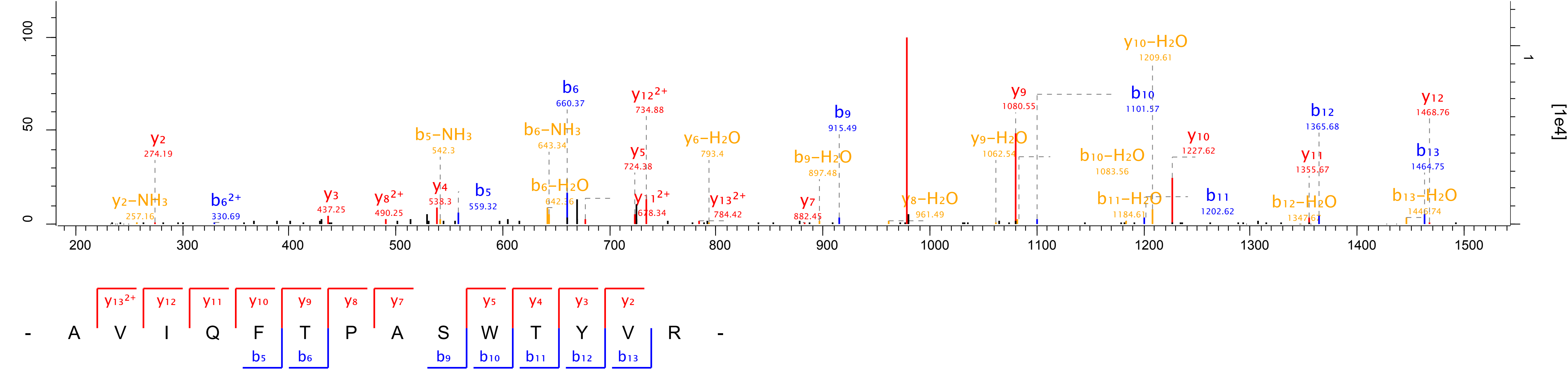

Raw file	Scan	Method	Score	m/z	Gene names
HBT_20130916_BV2_IL42_02	22815	ITMS; CID	122.44	998.49	Zc3h3;Gm7353

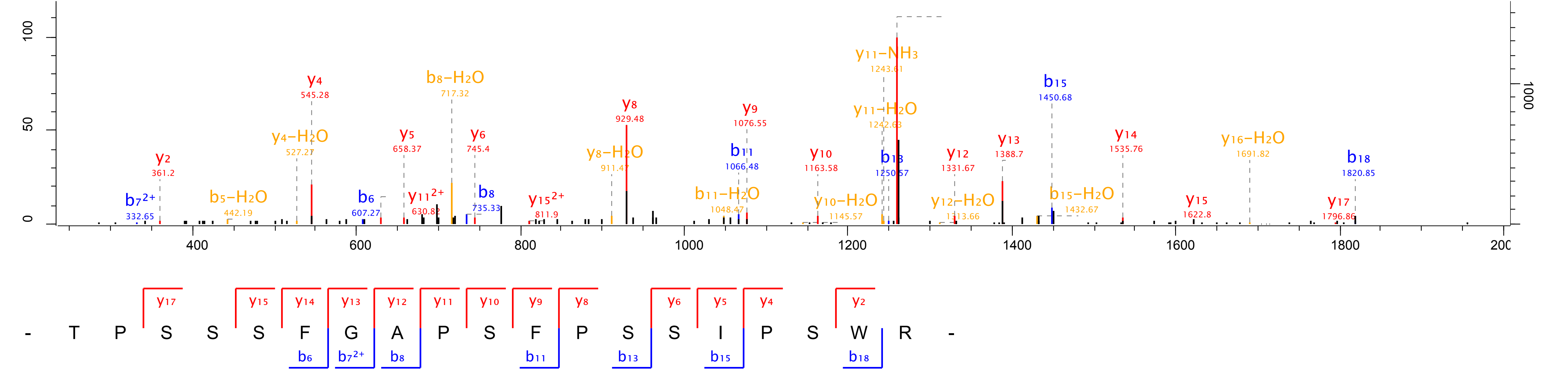

Raw file	Scan	Method	Score	m/z	Gene names
HBT_20130916_BV2_IL42_02	22342	ITMS; CID	151.22	697.82	Wdfy2

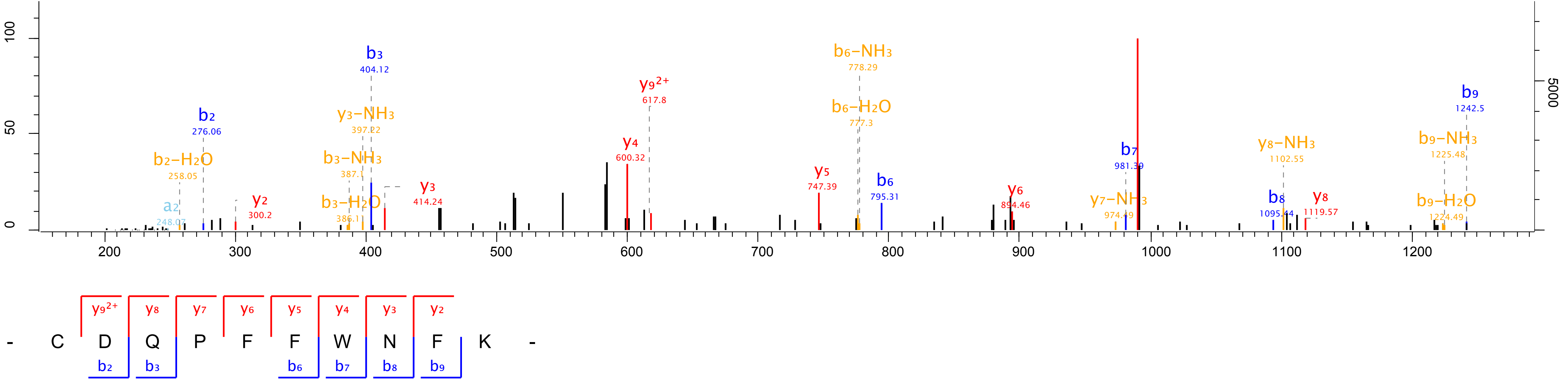

Raw file	Scan	Method	Score	m/z	Gene names
HBT_20130916_BV2_IL42_02	21967	ITMS; CID	82.48	657.89	Kansl3

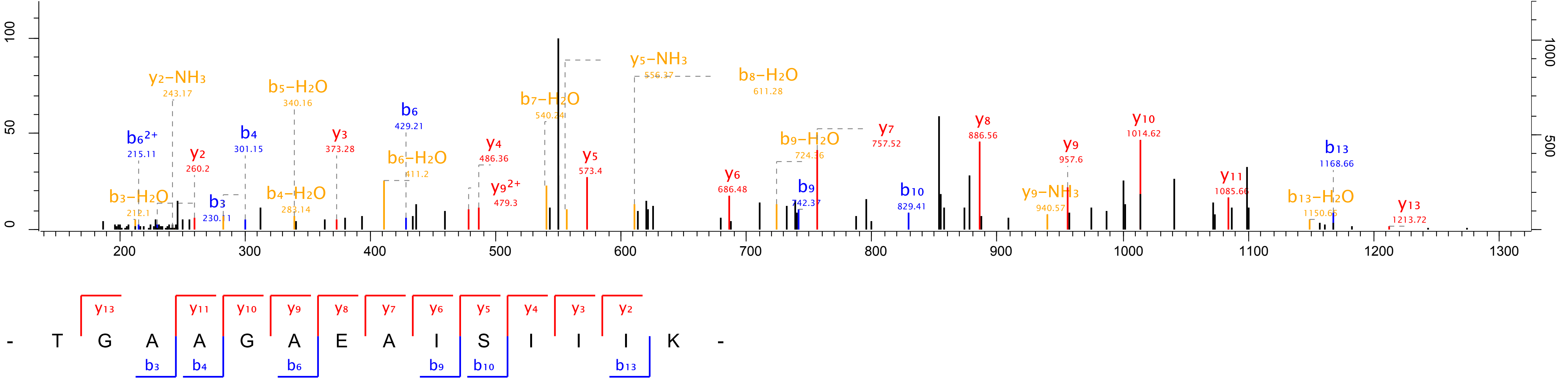

Raw file	Scan	Method	Score	m/z	Gene names
HBT_20130916_BV2_IL42_02	21378	ITMS; CID	79.35	845.96	Mea1

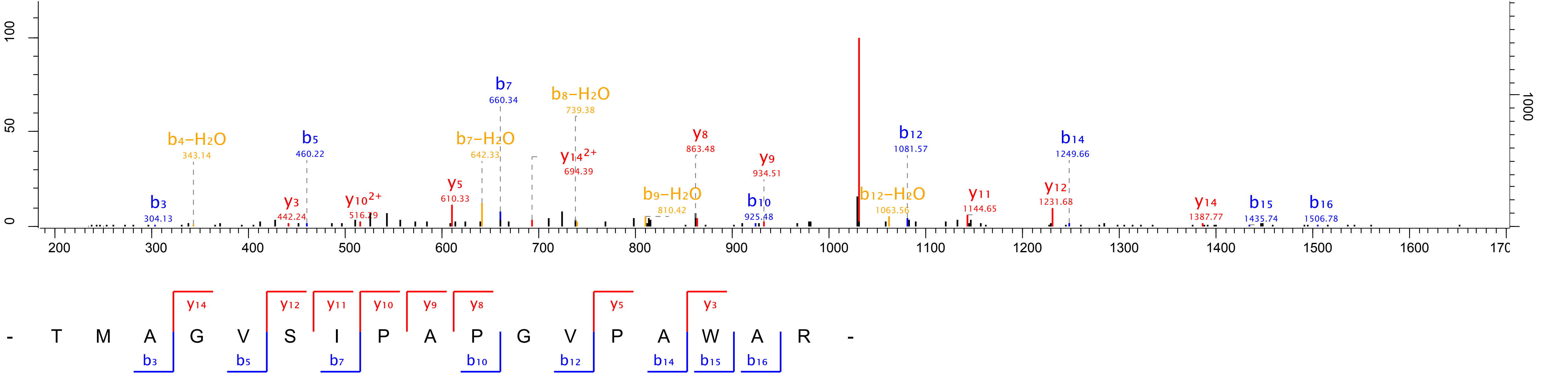

Raw file	Scan	Method	Score	m/z	Gene names
HBT_20130916_BV2_IL42_02	20489	ITMS; CID	153.72	874.98	Ech1

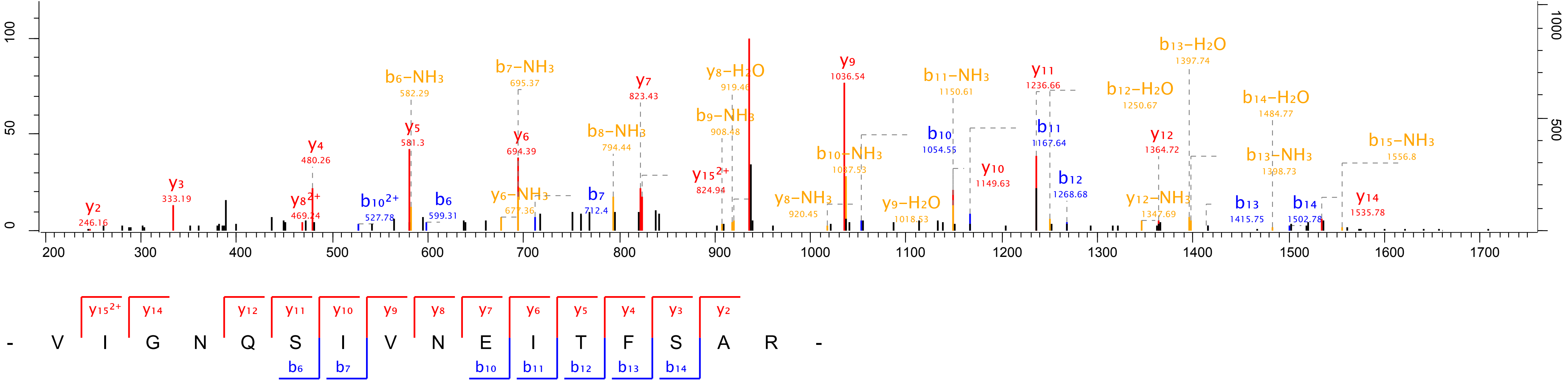

Raw file	Scan	Method	Score	m/z	Gene names
HBT_20130916_BV2_IL42_02	17730	ITMS; CID	108.32	799.89	Cln5

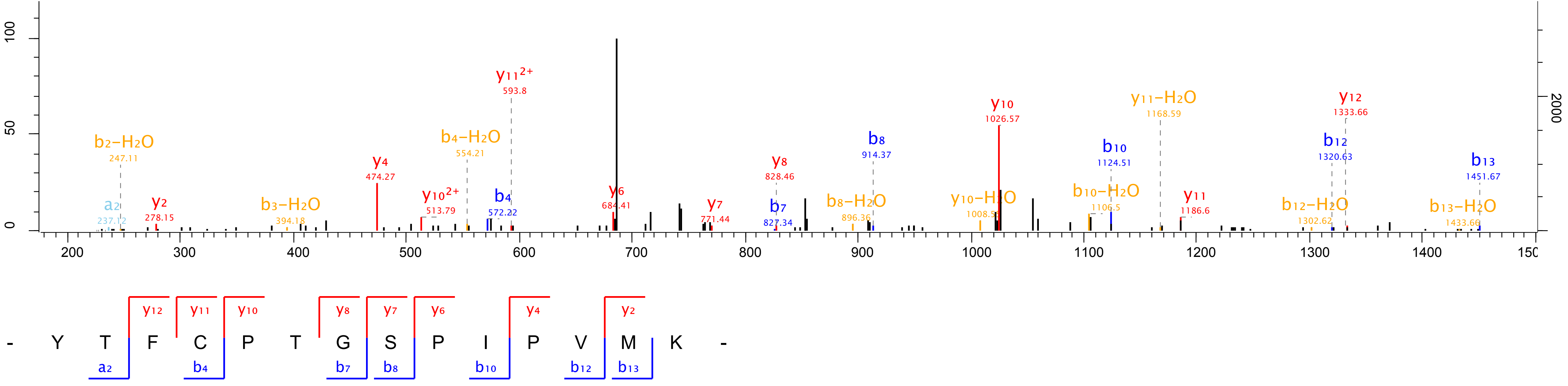

Raw file	Scan	Method	Score	m/z	Gene names
HBT_20130916_BV2_IL42_02	16228	ITMS; CID	112.11	525.82	Yipf3

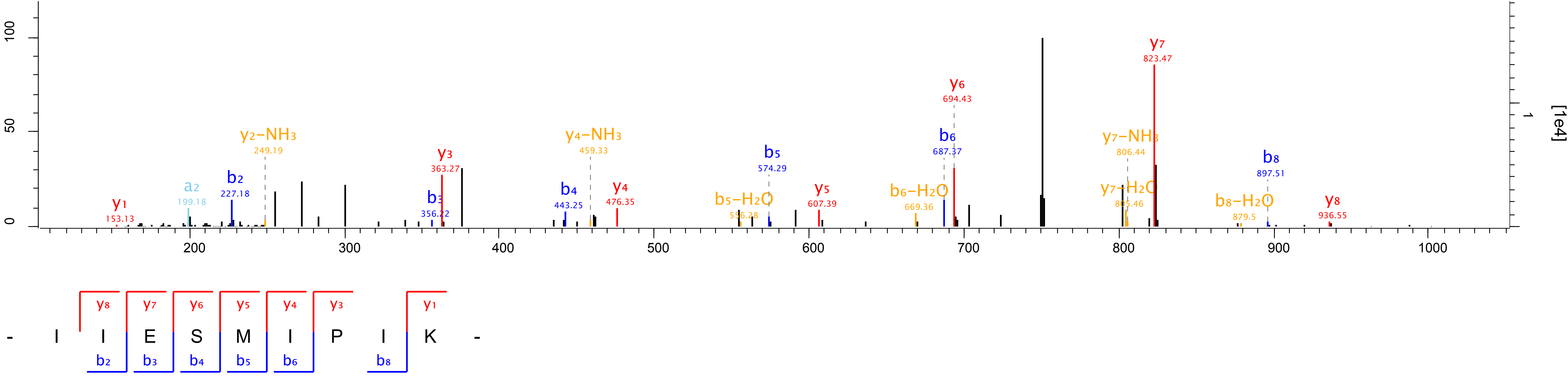

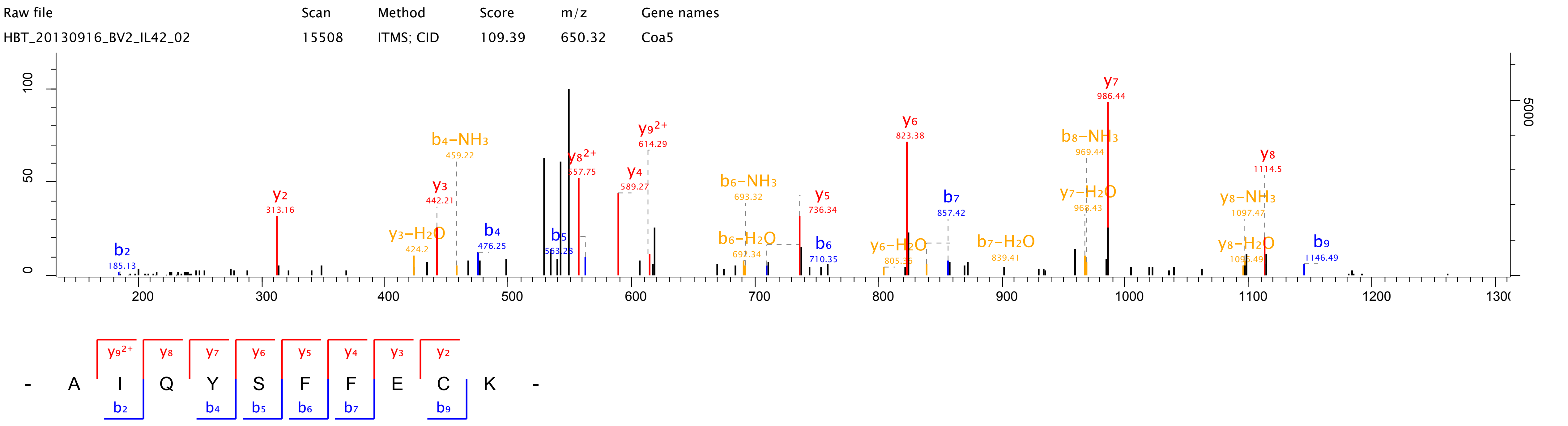
Raw file
HBT_20130916_BV2_IL42_02
Scan
15508
Method
ITMS; CID
Score
109.39
m/z
650.32
Gene names
Coa5
b2
185.13
y2
313.16
y3-H2O
424.2
y3
442.21
b4-NH3
459.22
b4
476.25
b5
563.28
y8 2+
557.75
y4
589.27
y9 2+
614.29
b6-NH3
693.32
b6-H2O
692.34
b6
710.35
y5
736.34
y6-H2O
805.35
y6
823.38
b7
857.42
b7-H2O
839.41
b8-NH3
969.44
y7-H2O
968.43
y7
986.44
y8-NH3
1097.47
y8-H2O
1096.49
y8
1114.5
b9
1146.49
- A I Q Y S F F E C K -
y9 2+ y8 y7 y6 y5 y4 y3 y2
b2 b4 b5 b6 b7 b9

Raw file	Scan	Method	Score	m/z	Gene names
HBT_20130916_BV2_IL42_02	14740	ITMS; CID	151.55	667.4	Mafb

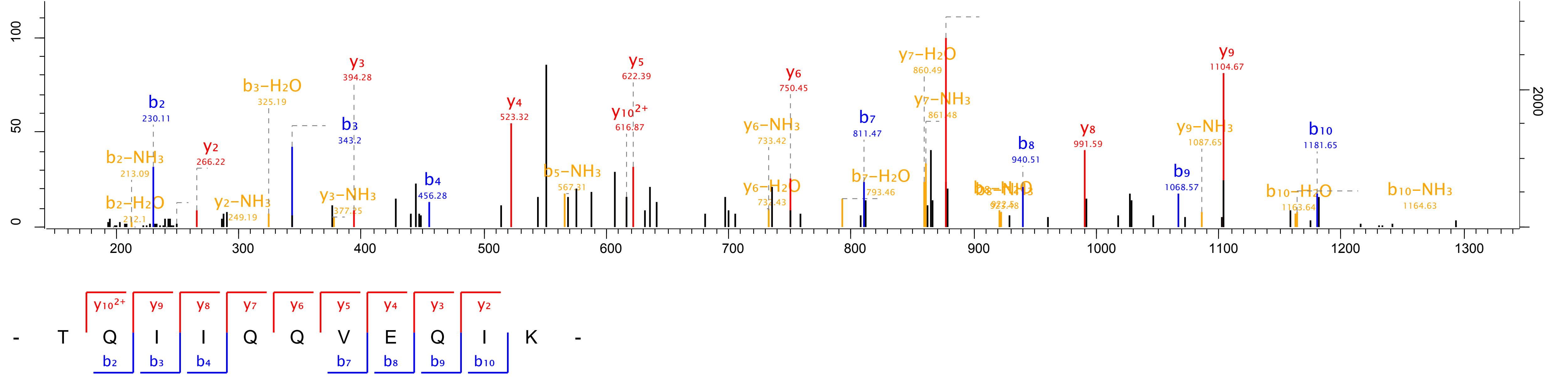

Raw file	Scan	Method	Score	m/z	Gene names
HBT_20130916_BV2_IL42_02	13028	ITMS; CID	125.84	702.91	Tpcn1

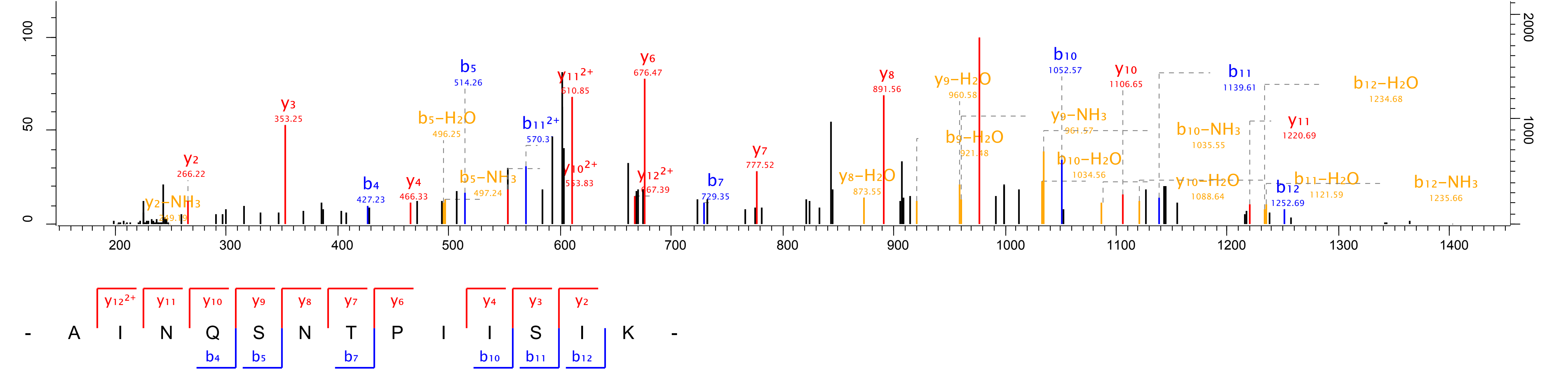

Raw file	Scan	Method	Score	m/z	Gene names
HBT_20130916_BV2_IL42_02	12367	ITMS; CID	115.04	874.45	Bri3

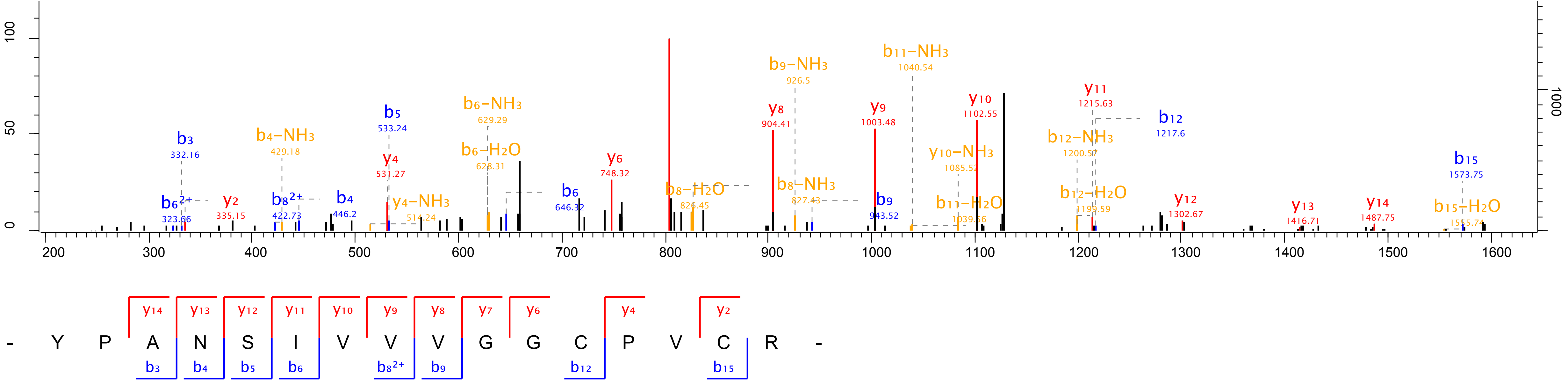

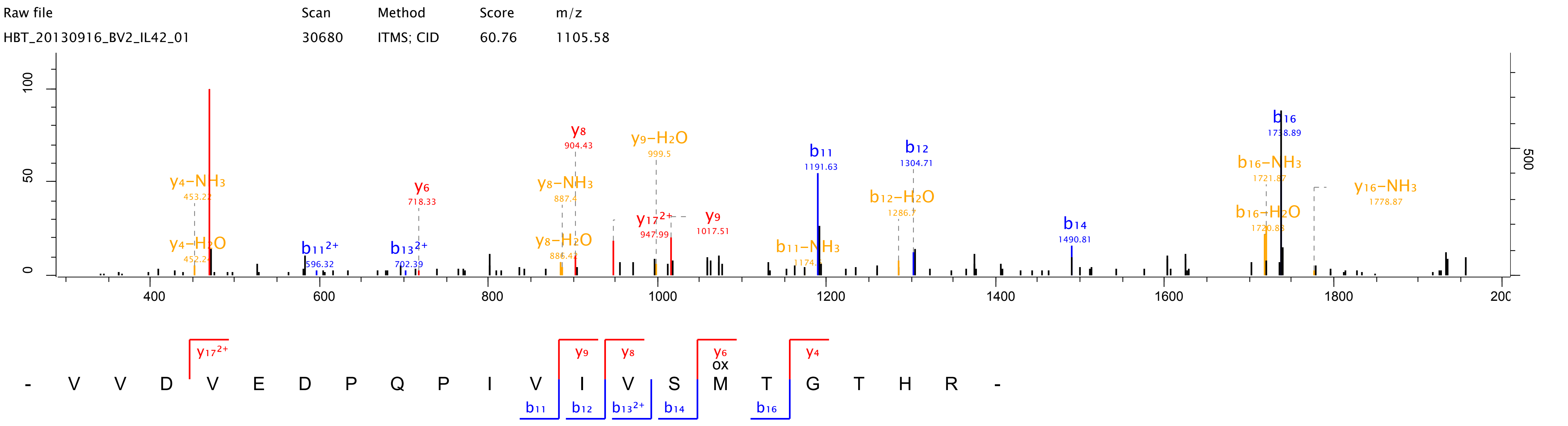

Raw file
HBT_20130916_BV2_IL42_01
Scan
30680
Method
ITMS; CID
Score
60.76
m/z
1105.58
y8
904.43
y9-H2O
999.5
b11
1191.63
b12
1304.71
b16
1738.89
y4-NH3
453.22
y6
718.33
y8-NH3
887.4
b16-NH3
1721.87
y16-NH3
1778.87
b12-H2O
1286.7
y17 2+
947.99
y9
1017.51
b16-H2O
1720.88
b14
1490.81
y4-H2O
452.24
b11 2+
596.32
b13 2+
702.39
y8-H2O
886.42
b11-NH3
- V V D V E D P Q P I V I V S M T G T H R -
ox

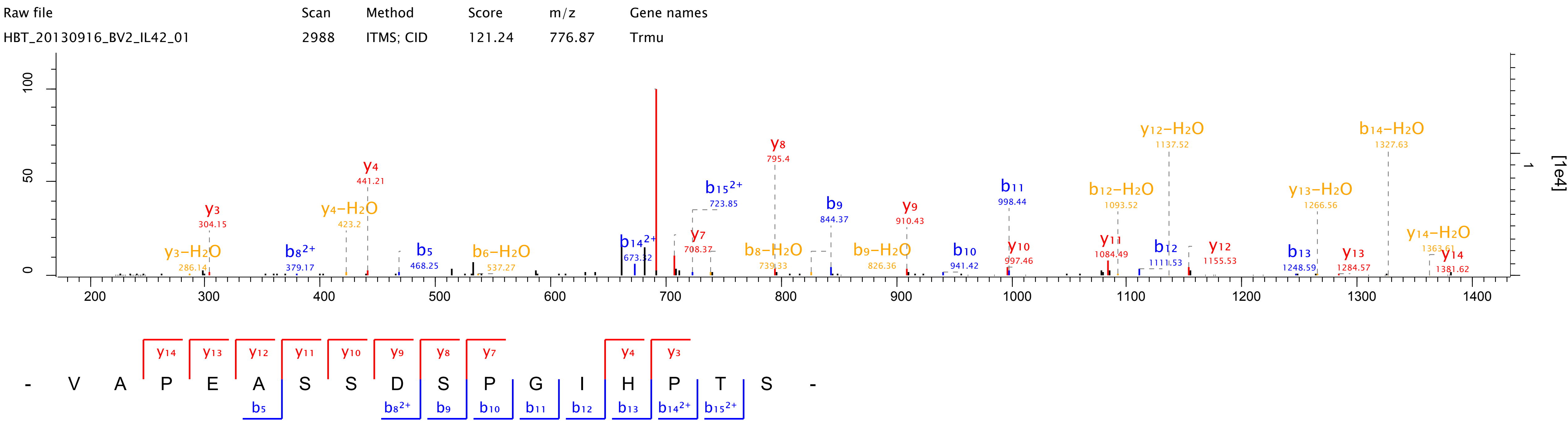
Raw file
HBT_20130916_BV2_IL42_01
Scan
2988
Method
ITMS; CID
Score
121.24
m/z
776.87
Gene names
Trmu
[1e4]
y3 304.15
y3−H2O 286.14
b8 2+ 379.17
y4−H2O 423.2
y4 441.21
b5 468.25
b6−H2O 537.27
b14 2+ 673.32
y7 708.37
b15 2+ 723.85
b8−H2O 739.33
y8 795.4
b9−H2O 826.36
b9 844.37
y9 910.43
b10 941.42
y10 997.46
b11 998.44
y11 1084.49
b12−H2O 1093.52
b12 1111.53
y12−H2O 1137.52
y12 1155.53
b13 1248.59
y13−H2O 1266.56
y13 1284.57
b14−H2O 1327.63
y14−H2O 1363.61
y14 1381.62
- V A P E A S S D S P G I H P T S -
y14 y13 y12 y11 y10 y9 y8 y7 y4 y3
b5 b8 2+ b9 b10 b11 b12 b13 b14 2+ b15 2+

Raw file	Scan	Method	Score	m/z	Gene names
HBT_20130916_BV2_IL42_01	25575	ITMS; CID	125.82	703.89	Supt3

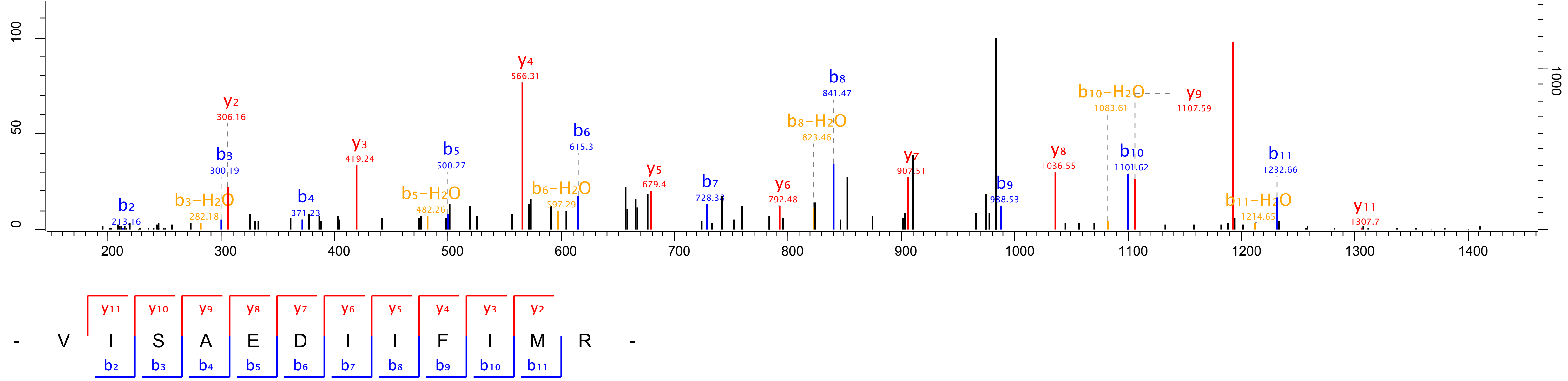

Raw file	Scan	Method	Score	m/z	Gene names
HBT_20130916_BV2_IL42_01	22482	ITMS; CID	110.31	717.44	Tsen34

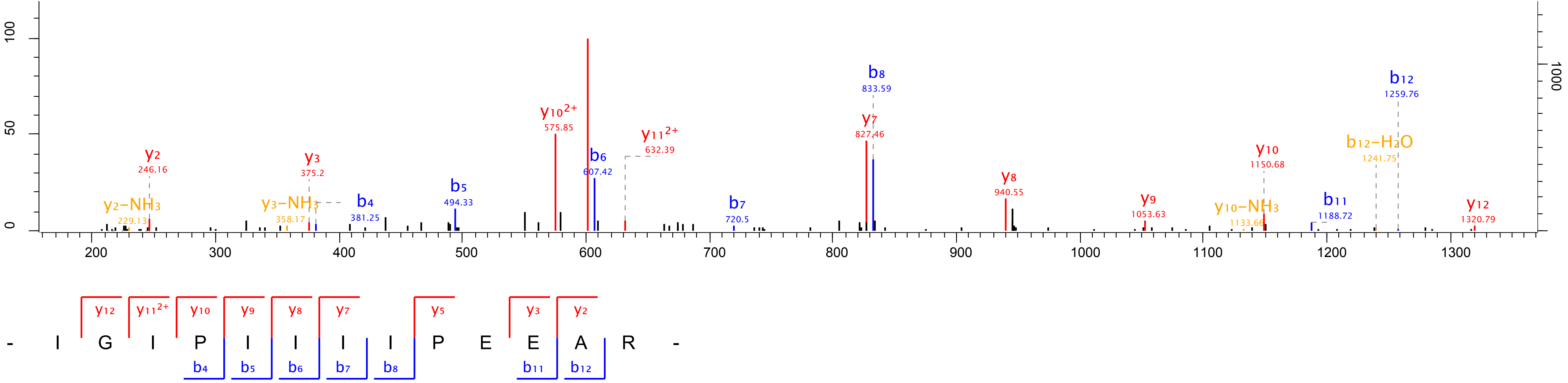

Raw file	Scan	Method	Score	m/z	Gene names
HBT_20130916_BV2_IL42_01	17840	ITMS; CID	93.26	971.48	Gnb2

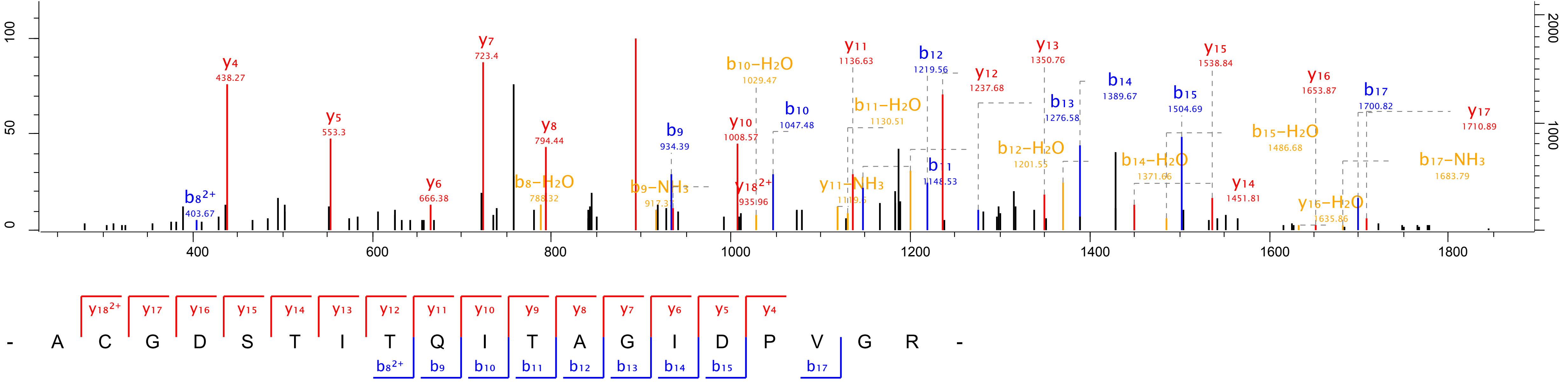

Raw file	Scan	Method	Score	m/z	Gene names
HBT_20130916_BV2_IL41_05	7138	ITMS; CID	184.44	636.84	Rtn3

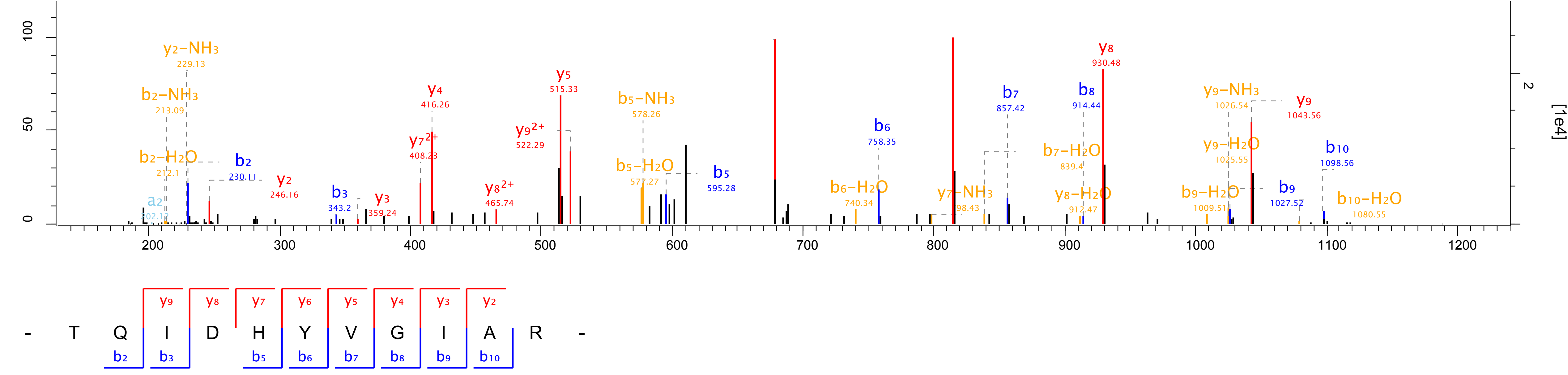

Raw file	Scan	Method	Score	m/z	Gene names
HBT_20130916_BV2_IL41_05	28004	ITMS; CID	86.22	801.42	Ap5s1

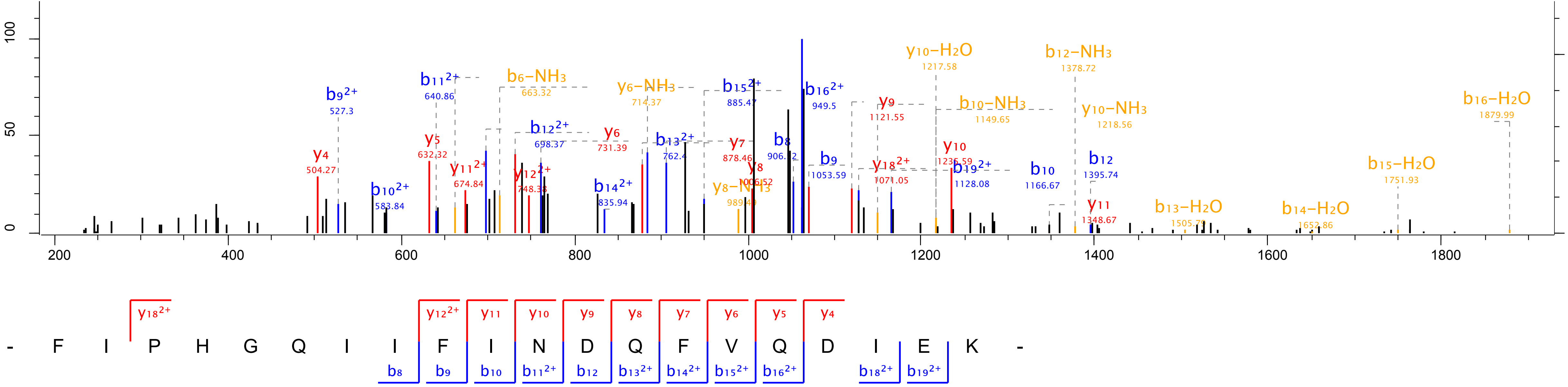

Raw file	Scan	Method	Score	m/z	Gene names
HBT_20130916_BV2_IL41_05	24863	ITMS; CID	94.69	537.31	Dhrsx

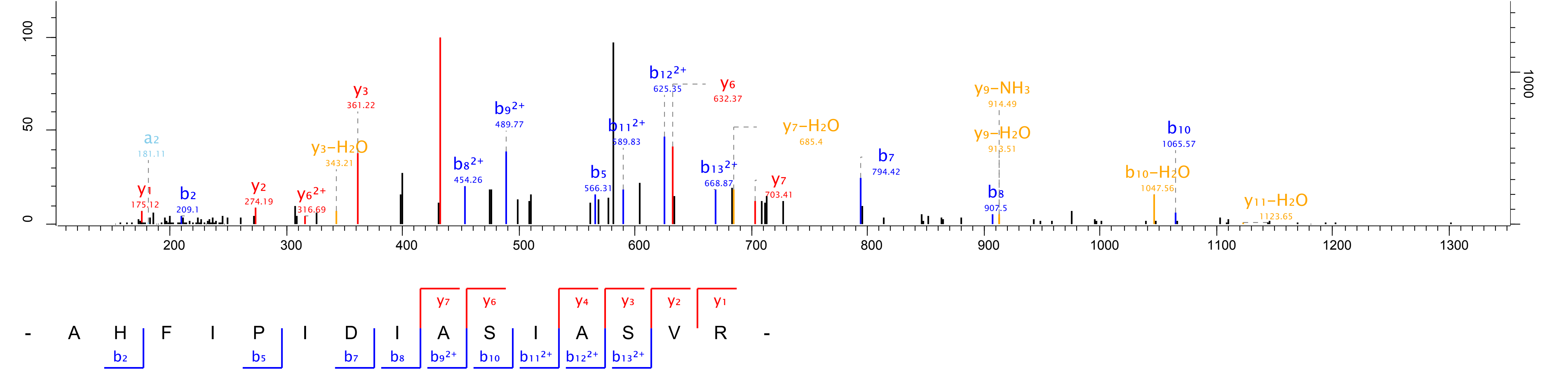

Raw file	Scan	Method	Score	m/z	Gene names
HBT_20130916_BV2_IL41_05	23385	ITMS; CID	71.88	554.34	Cln3

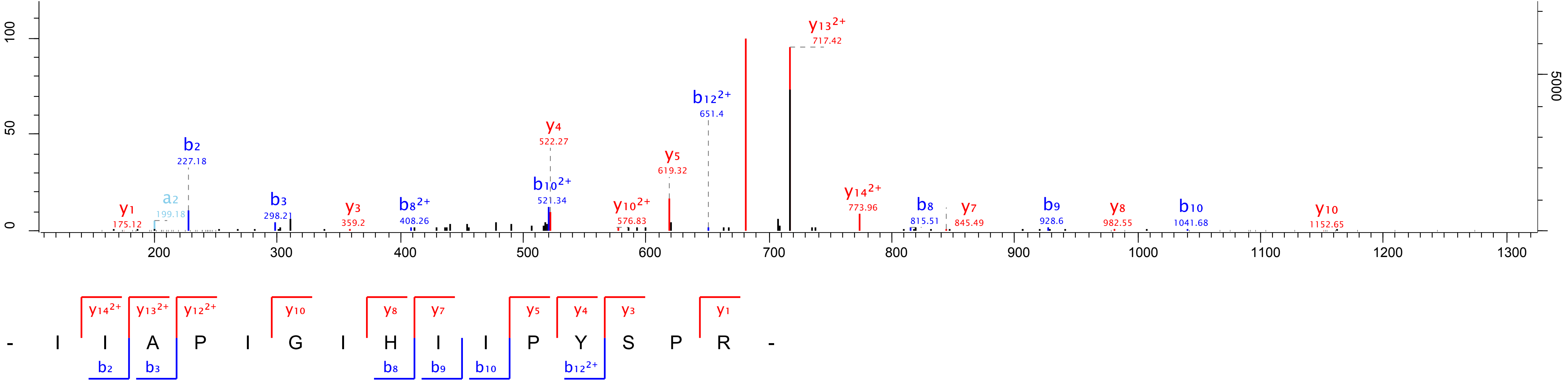

Raw file	Scan	Method	Score	m/z
HBT_20130916_BV2_IL41_05	21488	ITMS; CID	75.59	792.94

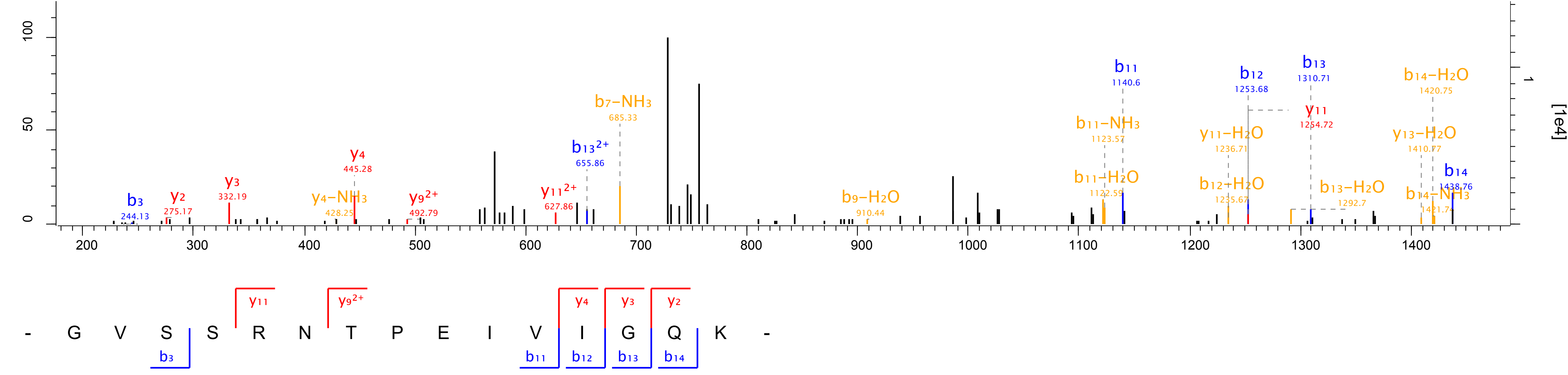

Raw file	Scan	Method	Score	m/z	Gene names
HBT_20130916_BV2_IL41_05	20206	ITMS; CID	66.39	557.65	Kiaa0907

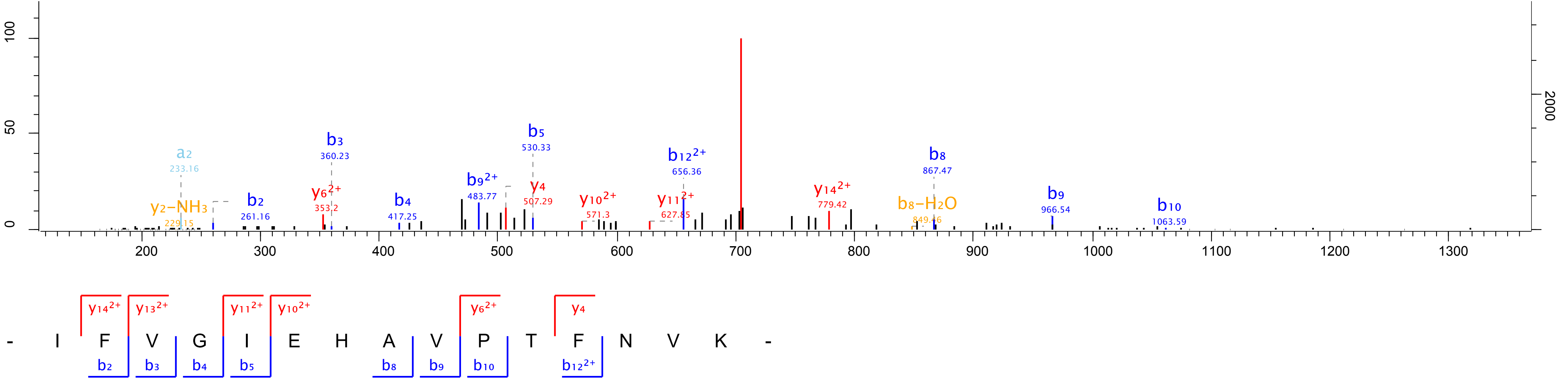

Raw file	Scan	Method	Score	m/z	Gene names
HBT_20130916_BV2_IL41_05	13092	ITMS; CID	171.92	687.35	Mrpl51

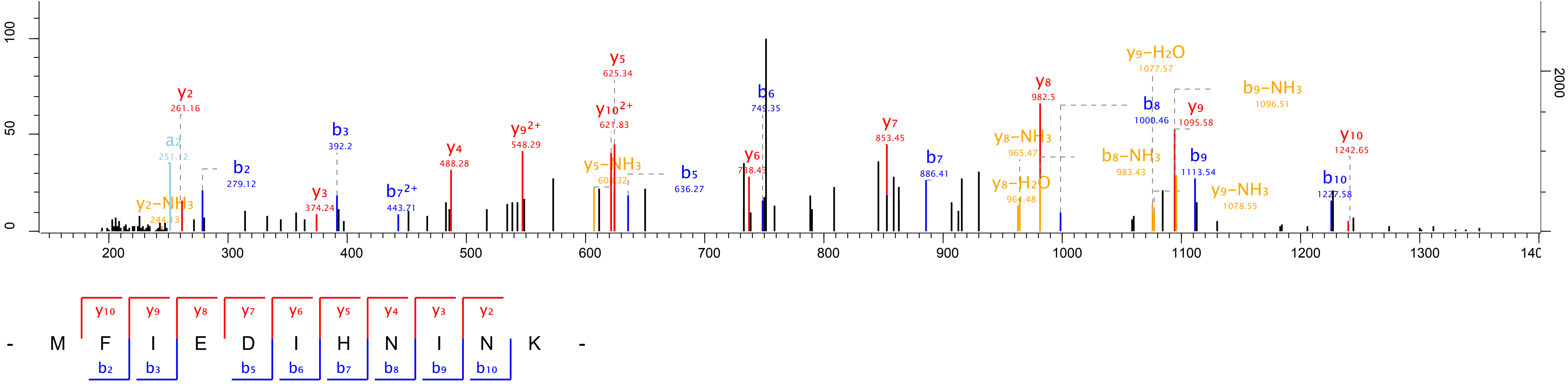

Raw file	Scan	Method	Score	m/z
HBT_20130916_BV2_IL41_05	11106	ITMS; CID	94.47	746.38

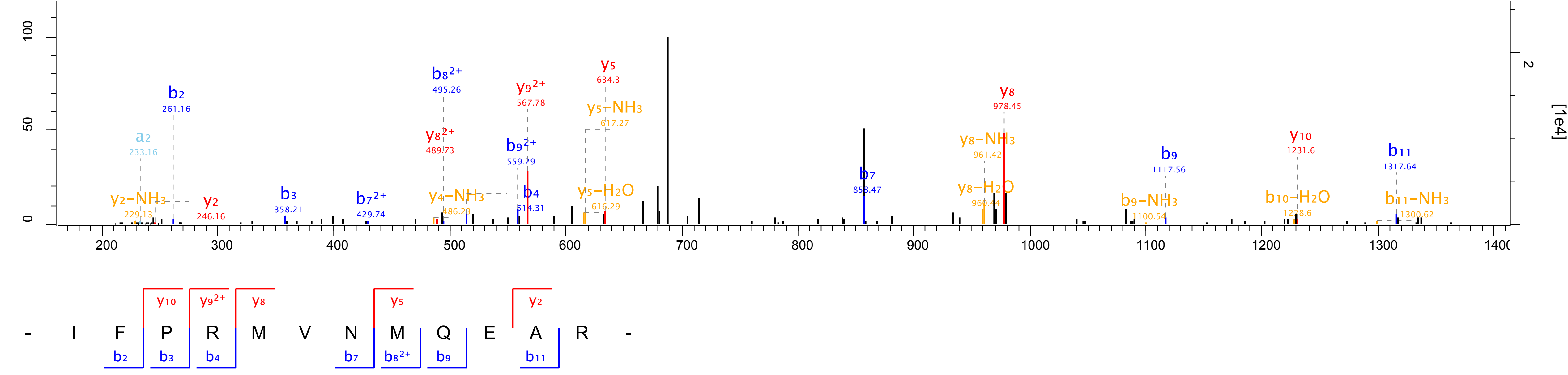

Raw file	Scan	Method	Score	m/z	Gene names
HBT_20130916_BV2_IL41_04	29023	ITMS; CID	74.15	696.37	Ndrg1

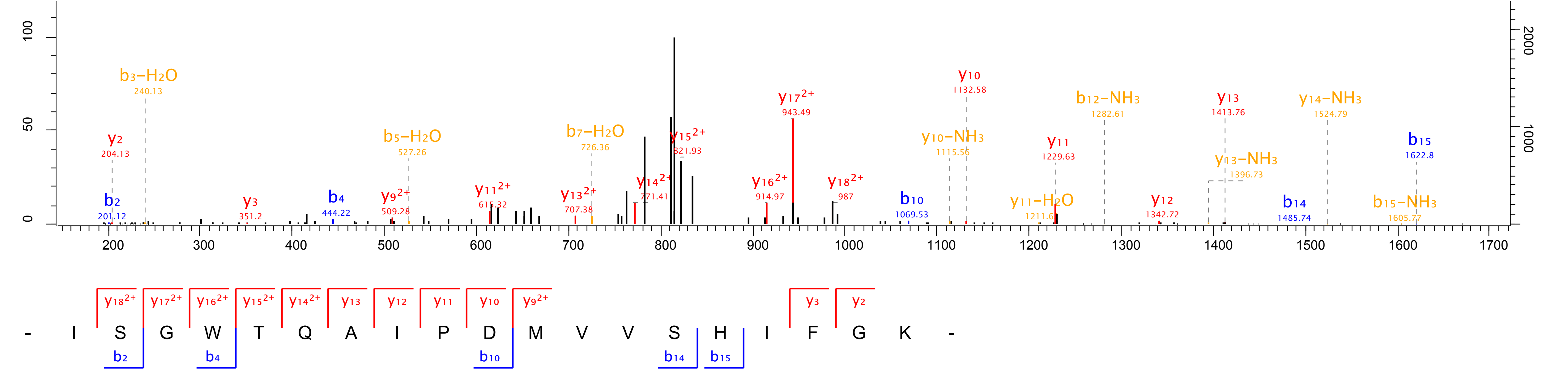

Raw file	Scan	Method	Score	m/z	Gene names
HBT_20130916_BV2_IL41_04	26092	ITMS; CID	109.54	657.72	Prkag2

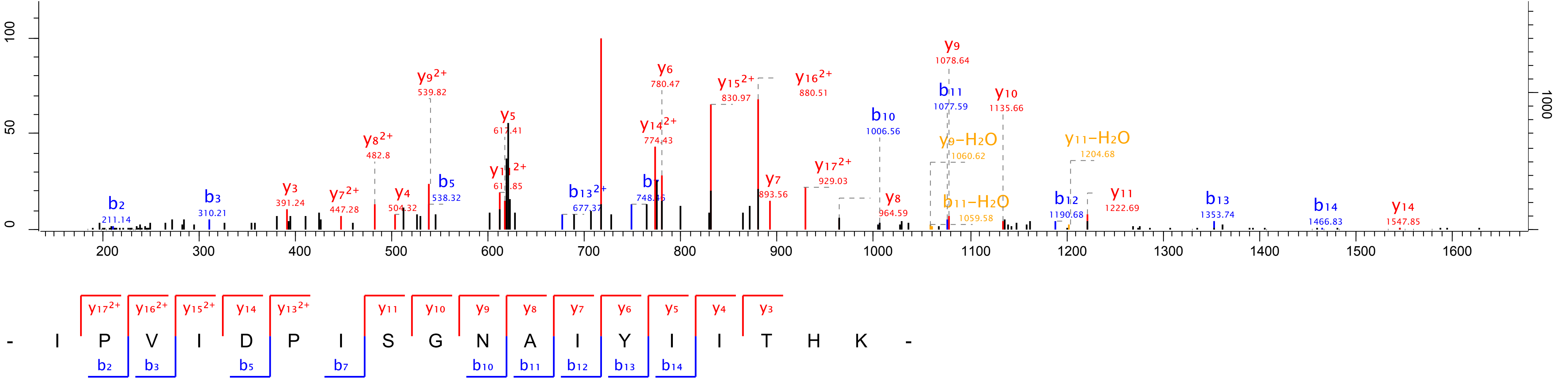

Raw file	Scan	Method	Score	m/z	Gene names
HBT_20130916_BV2_IL41_04	22934	ITMS; CID	64.84	649.36	Zbtb7a

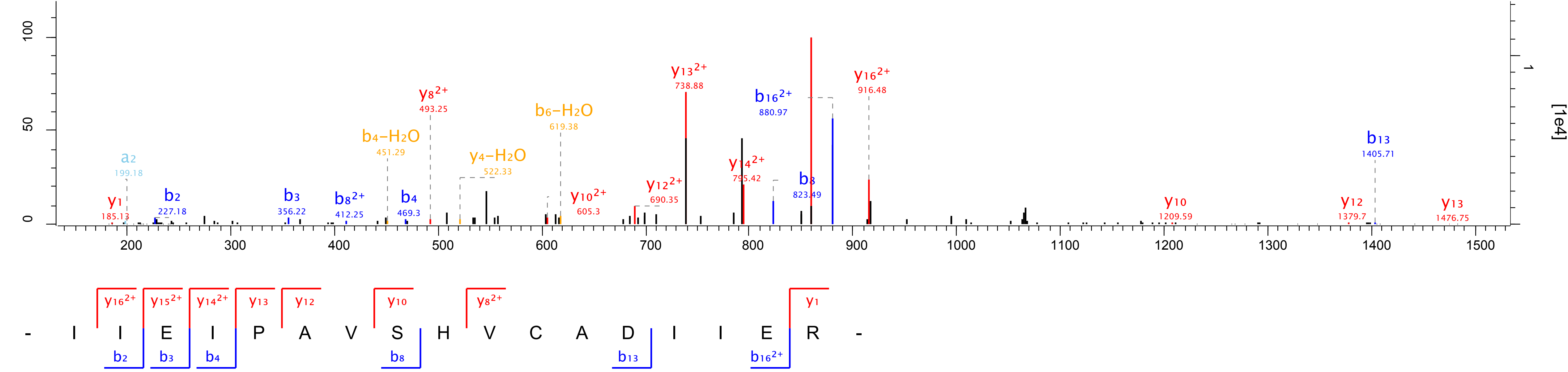

Raw file	Scan	Method	Score	m/z	Gene names
HBT_20130916_BV2_IL41_04	13982	ITMS; CID	122.1	768.42	Mrp63

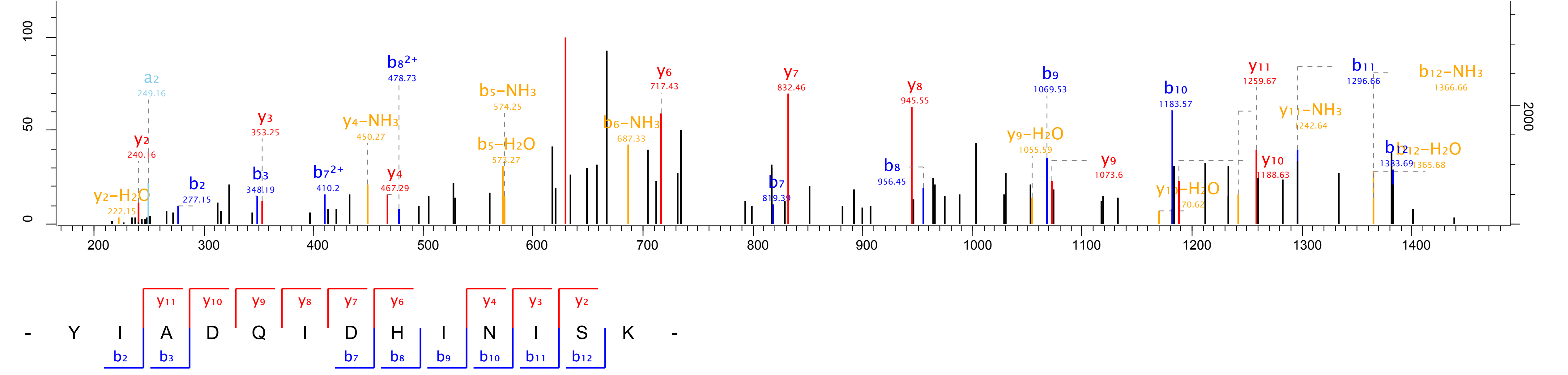

Raw file	Scan	Method	Score	m/z	Gene names
HBT_20130916_BV2_IL41_03	24548	ITMS; CID	159.83	619.35	Dis3l

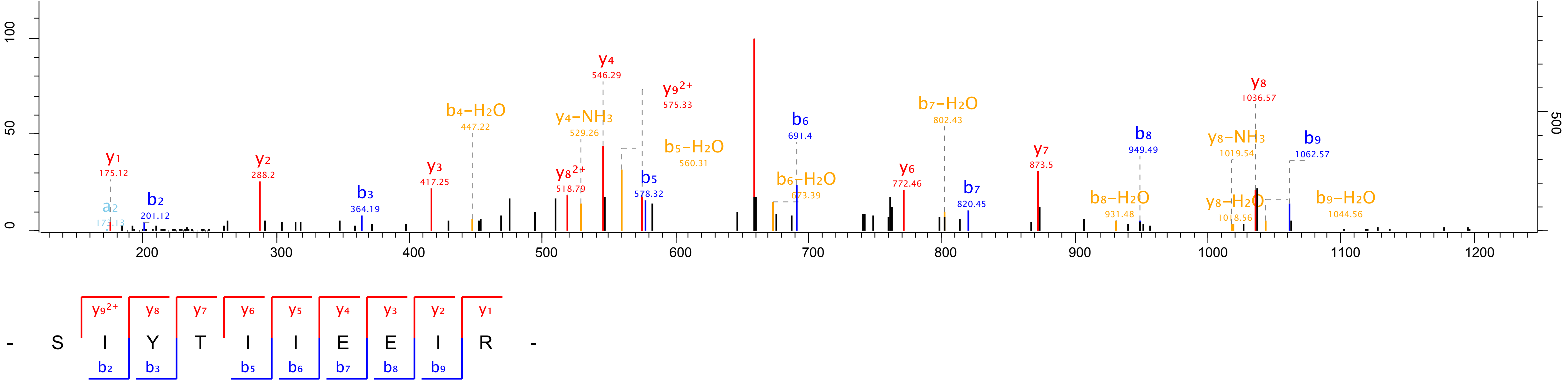

Raw file	Scan	Method	Score	m/z	Gene names
HBT_20130916_BV2_IL41_03	21284	ITMS; CID	129.42	534.78	Mau2

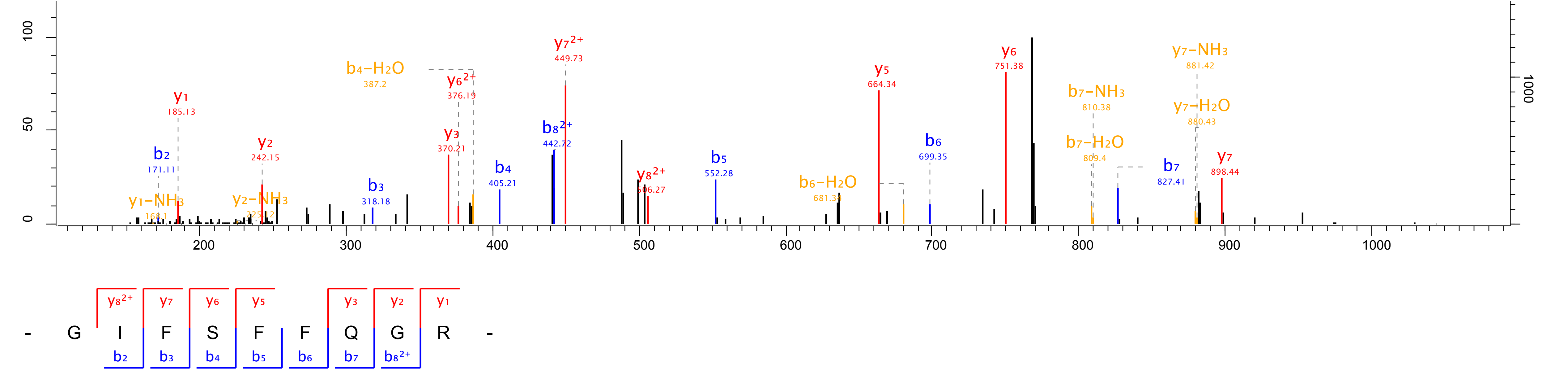

Raw file	Scan	Method	Score	m/z	Gene names
HBT_20130916_BV2_IL41_03	20567	ITMS; CID	130.66	576.35	Qrsl1

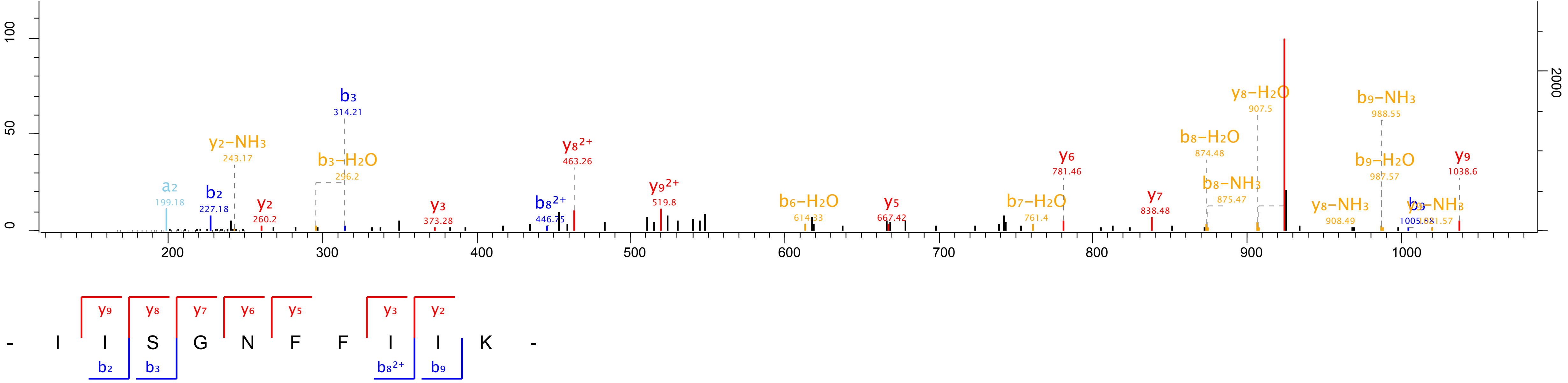

Raw file	Scan	Method	Score	m/z	Gene names
HBT_20130916_BV2_IL41_03	20145	ITMS; CID	156.16	632.34	Cdyl

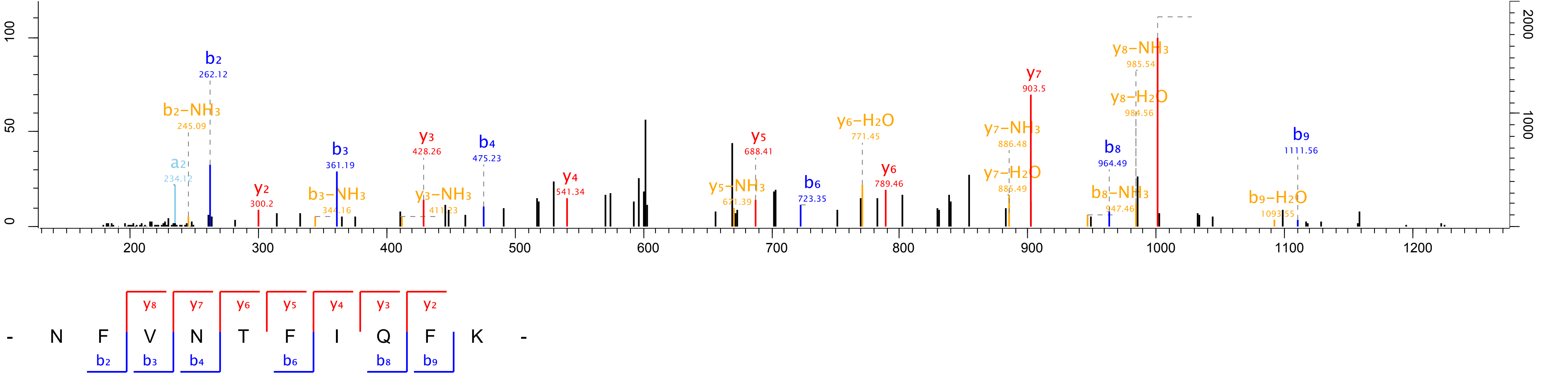

Raw file	Scan	Method	Score	m/z	Gene names
HBT_20130916_BV2_IL41_03	19462	ITMS; CID	190.26	494.3	Fbxl8

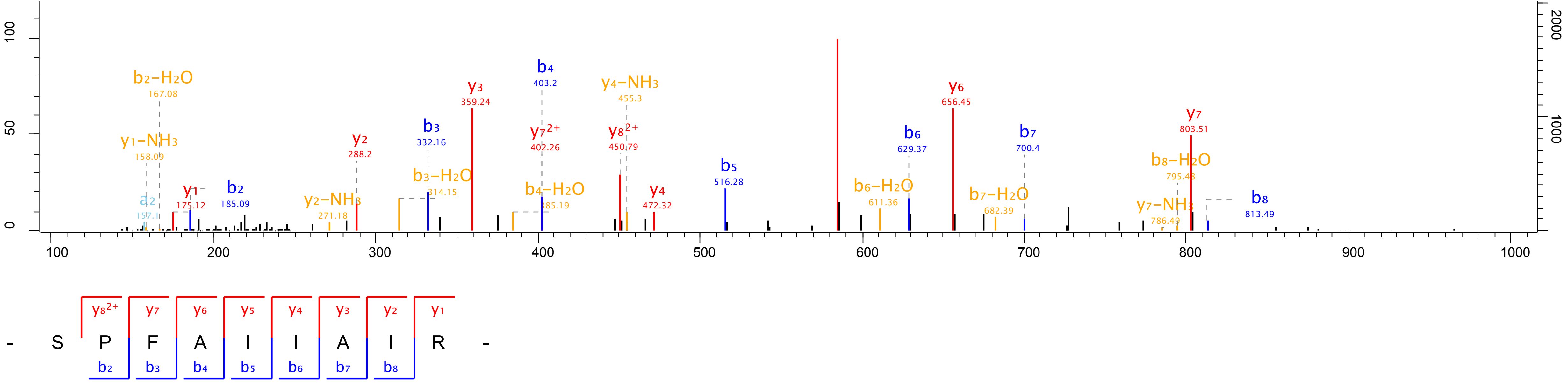

Raw file	Scan	Method	Score	m/z	Gene names
HBT_20130916_BV2_IL41_03	16011	ITMS; CID	178.03	907.94	Clcn5

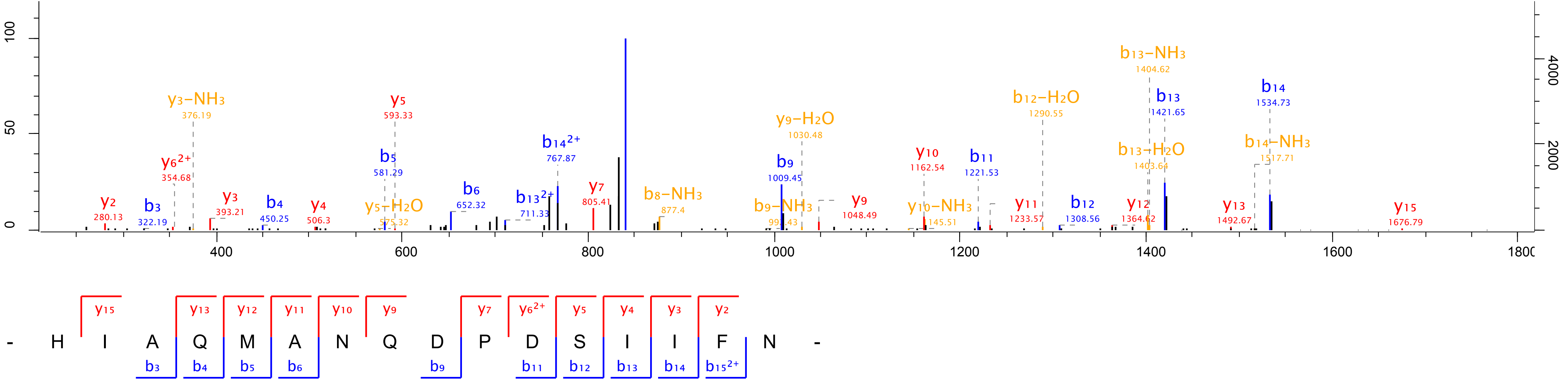

Raw file	Scan	Method	Score	m/z	Gene names
HBT_20130916_BV2_IL41_02	3461	ITMS; CID	144.77	587.33	Tktl1

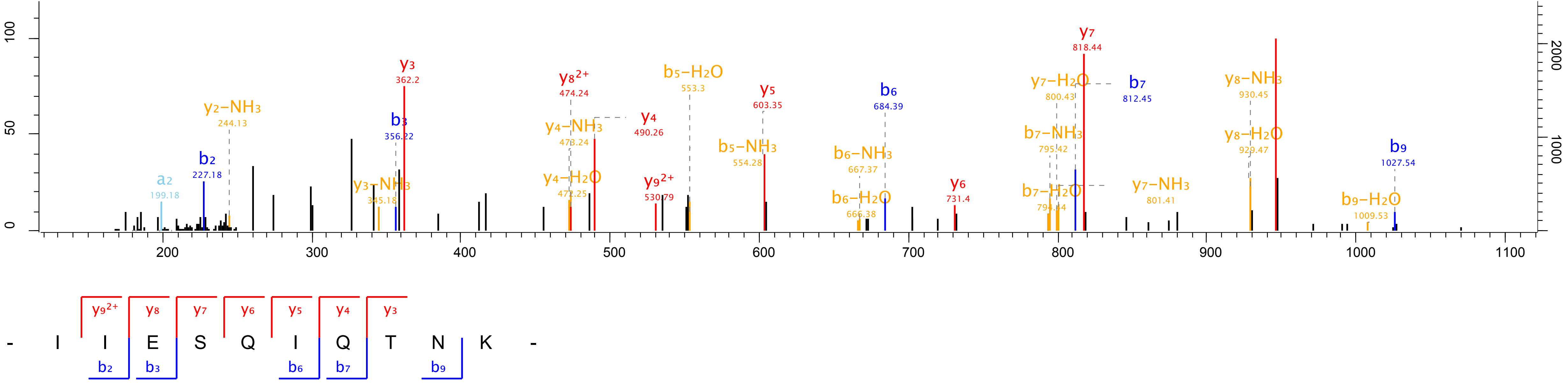

Raw file	Scan	Method	Score	m/z	Gene names
HBT_20130916_BV2_IL41_02	27758	ITMS; CID	140.31	782.97	Iigp1

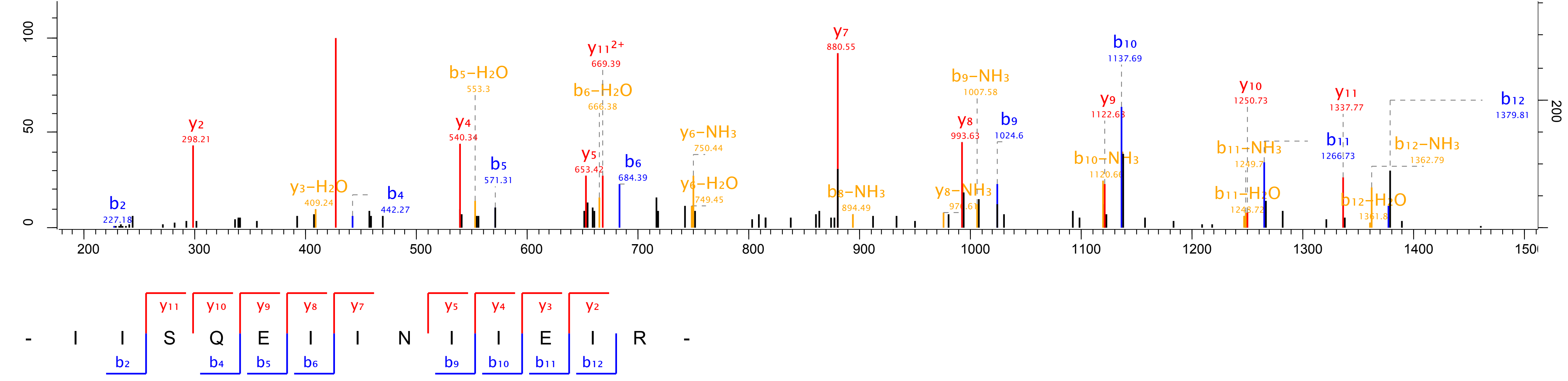

Raw file	Scan	Method	Score	m/z	Gene names
HBT_20130916_BV2_IL41_02	27365	ITMS; CID	124.47	724.46	Mto1

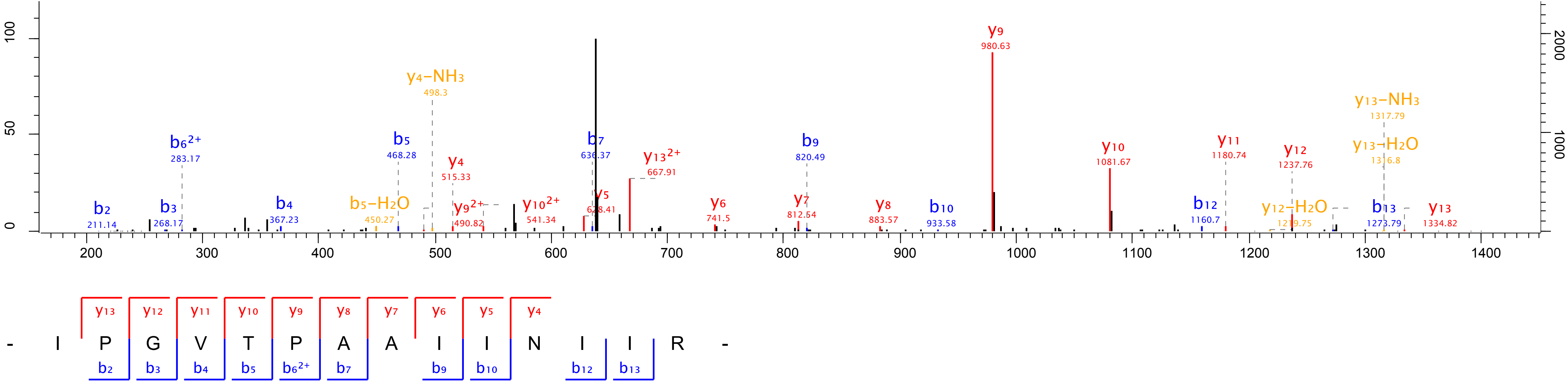

Raw file	Scan	Method	Score	m/z	Gene names
HBT_20130916_BV2_IL41_02	24412	ITMS; CID	97.73	630.36	Tlk1

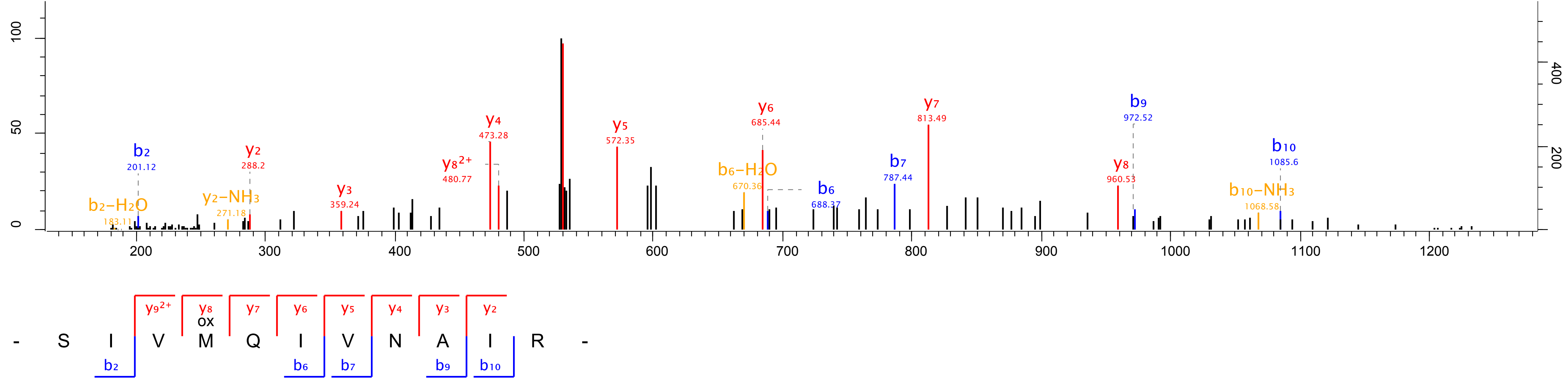

Raw file	Scan	Method	Score	m/z	Gene names
HBT_20130916_BV2_IL41_02	20854	ITMS; CID	112.85	591.37	Smcr8

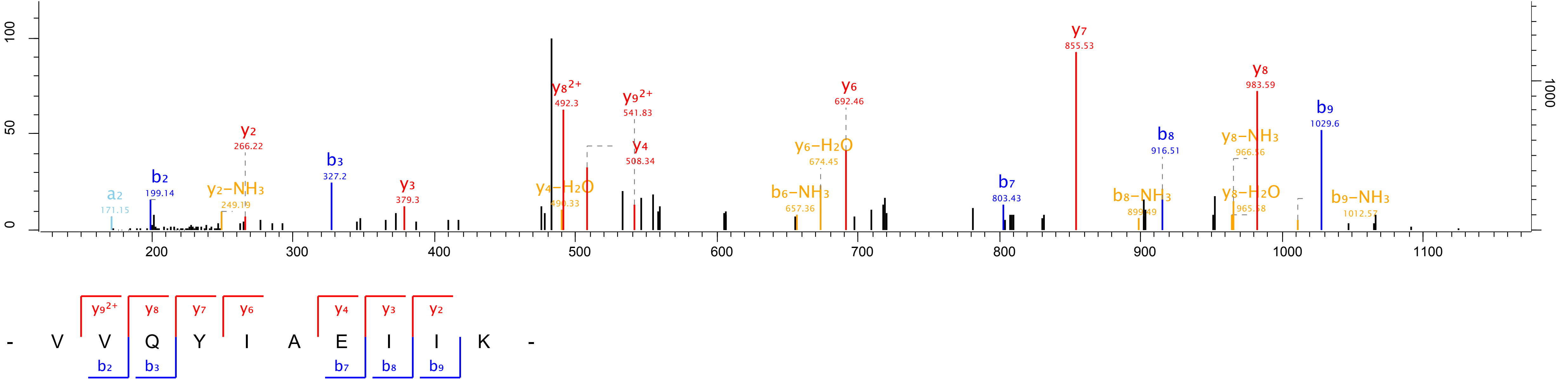

Raw file	Scan	Method	Score	m/z	Gene names
HBT_20130916_BV2_IL41_02	18743	ITMS; CID	76.07	527.29	Otof

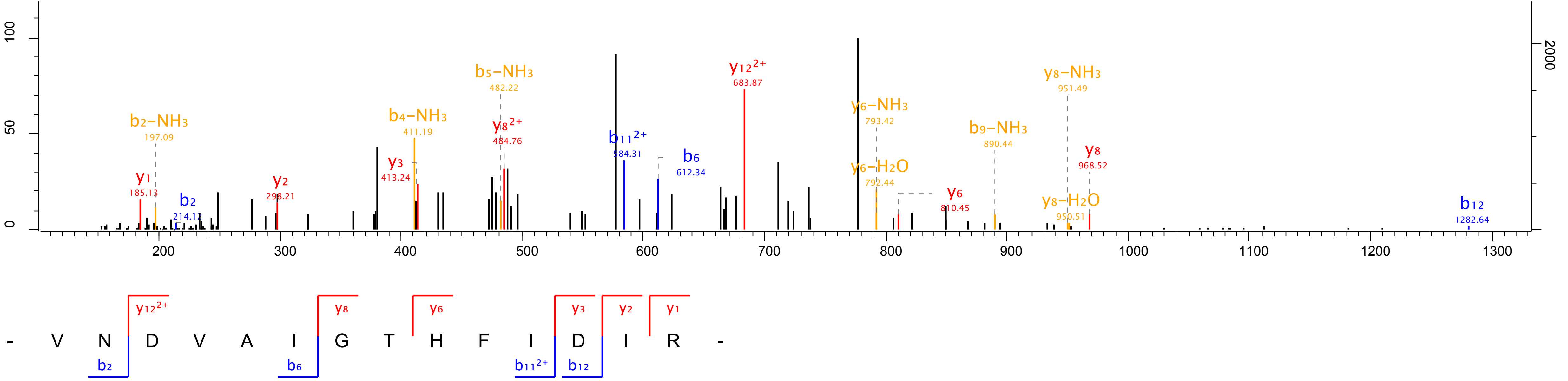

Raw file	Scan	Method	Score	m/z	Gene names
HBT_20130916_BV2_IL41_02	16228	ITMS; CID	107.34	588.87	Slc39a10

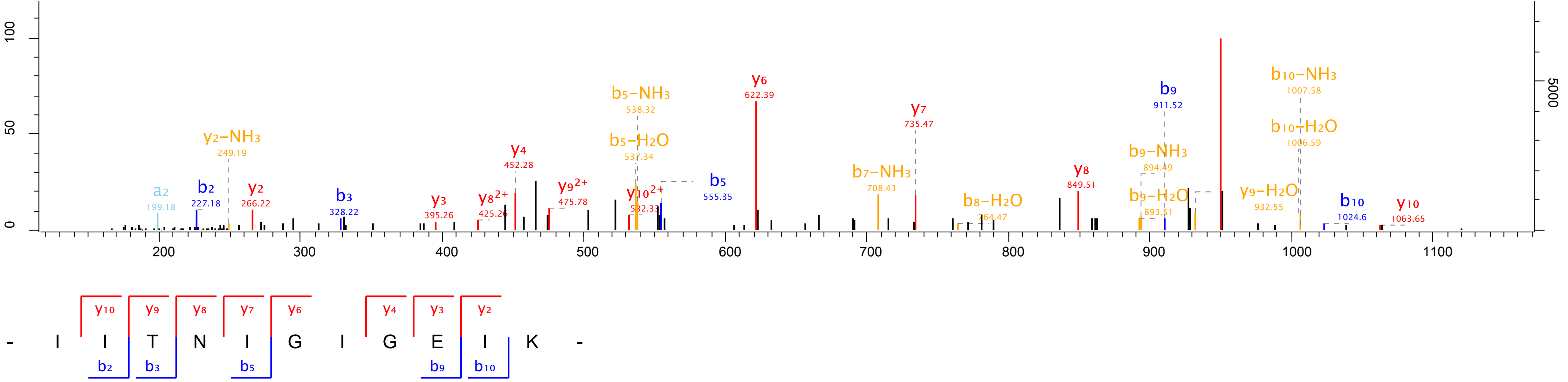

Raw file	Scan	Method	Score	m/z	Gene names
HBT_20130916_BV2_IL41_02	13446	ITMS; CID	209.21	632.38	Hic2

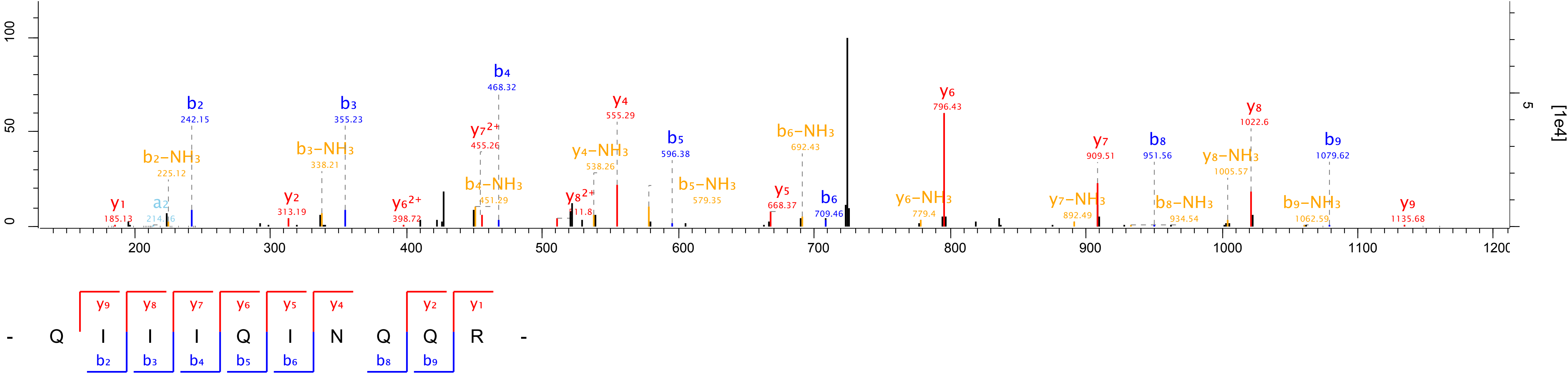

Raw file	Scan	Method	Score	m/z	Gene names
HBT_20130916_BV2_IL41_02	13220	ITMS; CID	116.55	623.35	Tango2

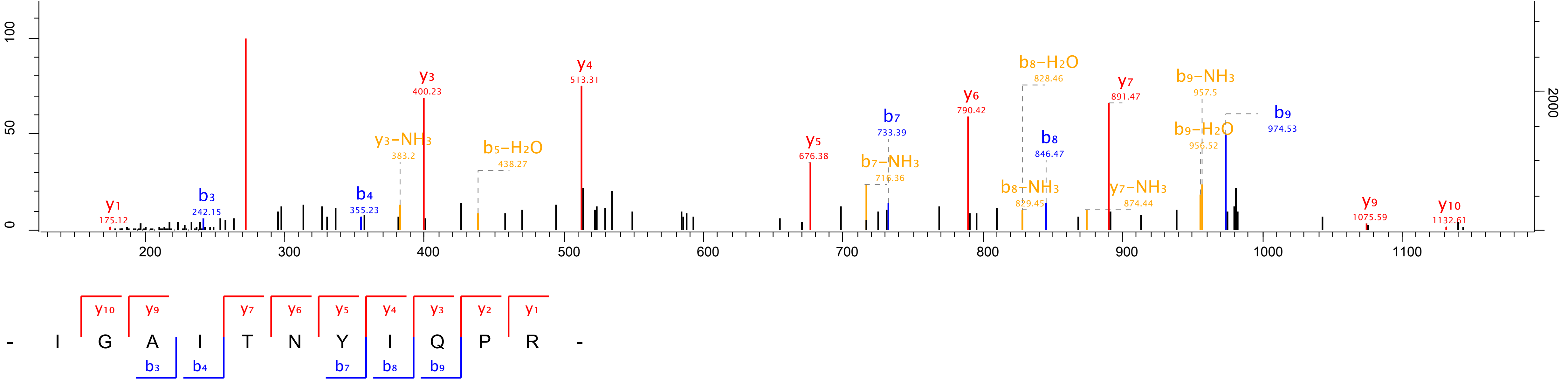

Raw file	Scan	Method	Score	m/z	Gene names
HBT_20130916_BV2_IL41_02	1216	ITMS; CID	131.62	503.78	Eng

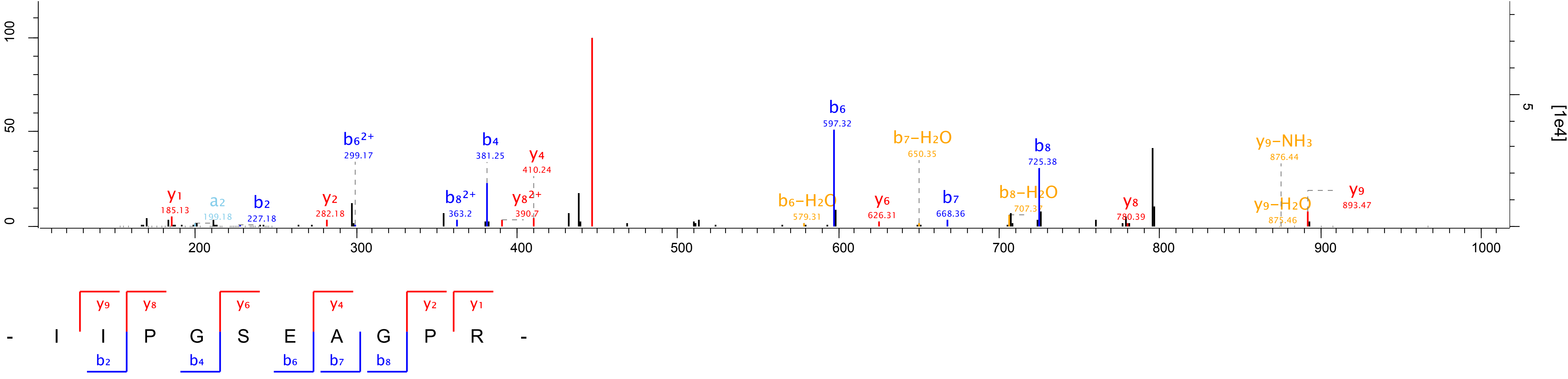

Raw file	Scan	Method	Score	m/z	Gene names
HBT_20130916_BV2_IL41_01	4359	ITMS; CID	193.15	621.84	Sumo3;Sumo2

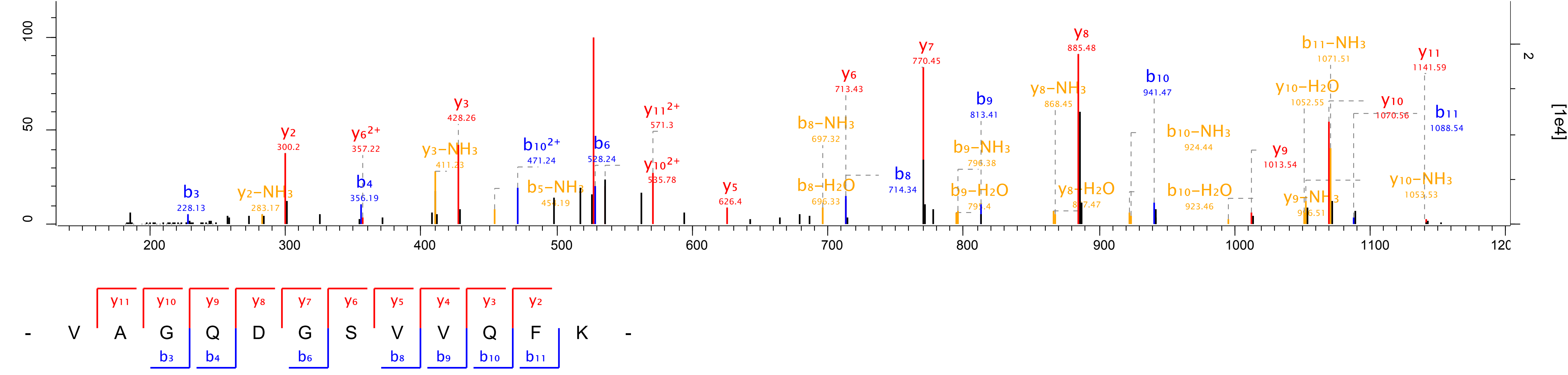

Raw file	Scan	Method	Score	m/z
HBT_20130916_BV2_IL41_01	2713	ITMS; CID	99.79	550.8

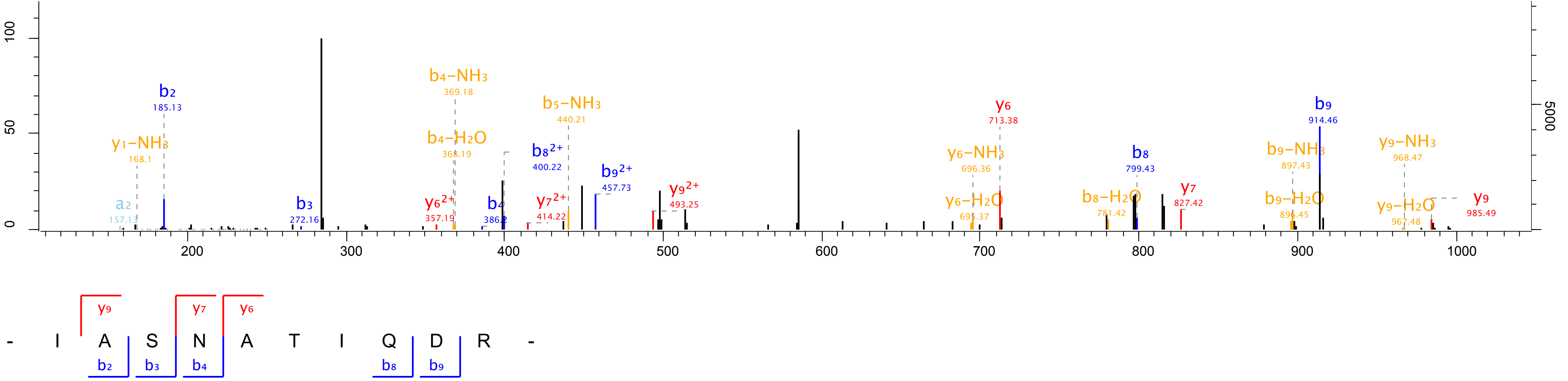

Raw file	Scan	Method	Score	m/z	Gene names
HBT_20130916_BV2_IL41_01	24958	ITMS; CID	76.76	1150.62	Parl

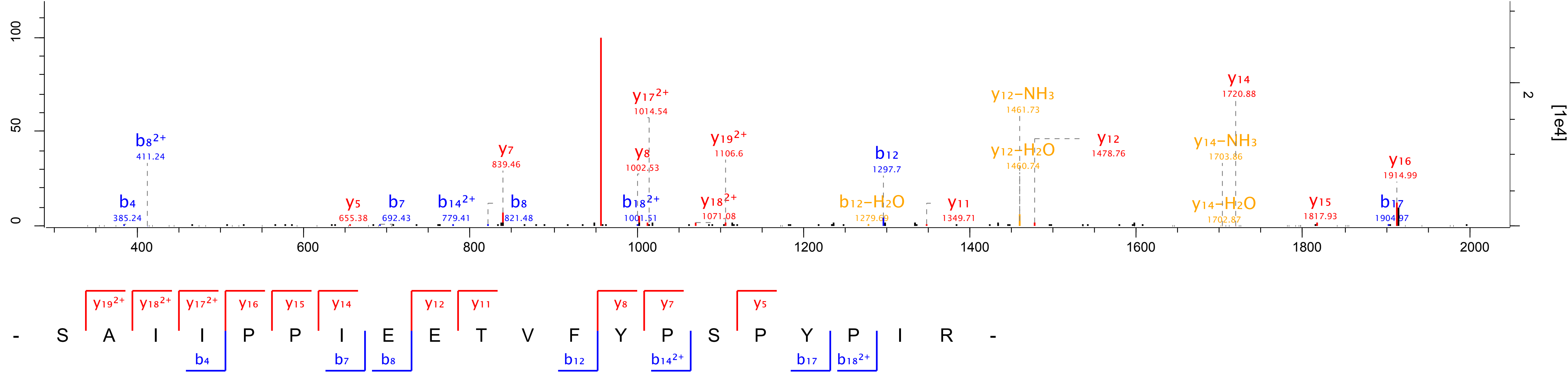

Raw file	Scan	Method	Score	m/z	Gene names
HBT_20130916_BV2_IL41_01	17805	ITMS; CID	193.98	859.92	Ssr3

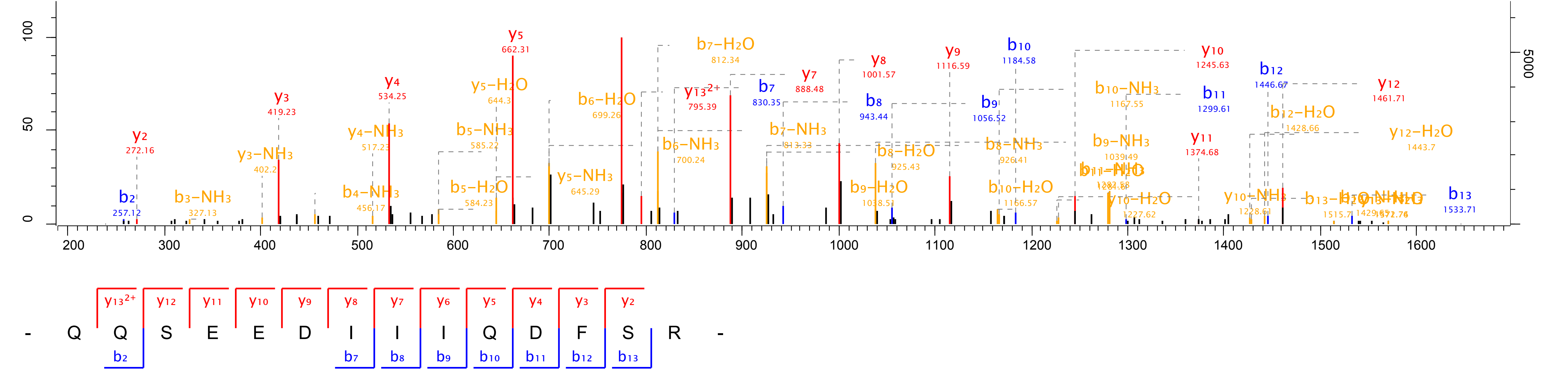

Raw file	Scan	Method	Score	m/z	Gene names
HBT_20130916_BV2_IL103_06	6383	ITMS; CID	97.6	623.82	Arl6ip1

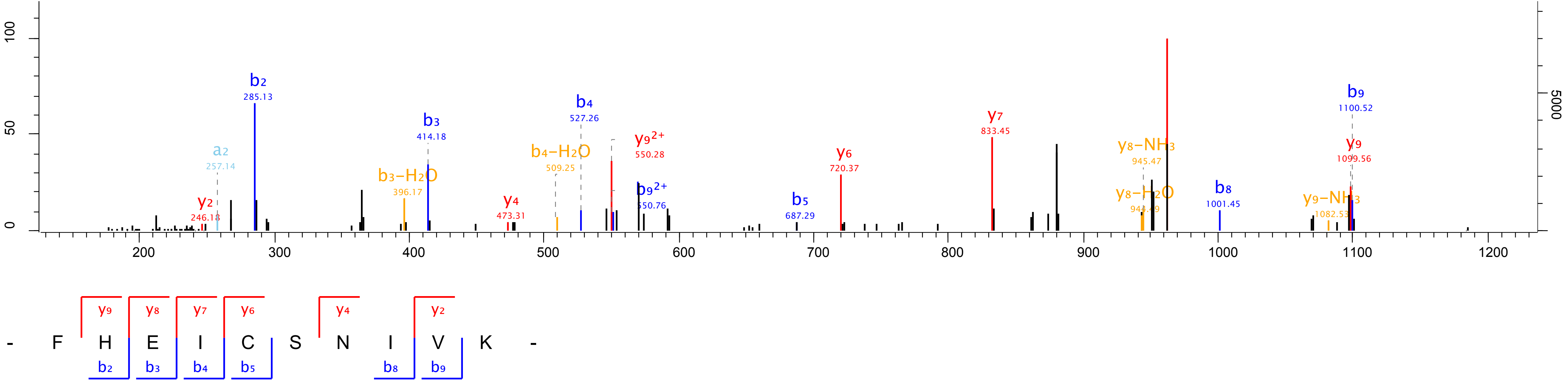

Raw file	Scan	Method	Score	m/z
HBT_20130916_BV2_IL103_05	9088	ITMS; CID	87.45	862.97

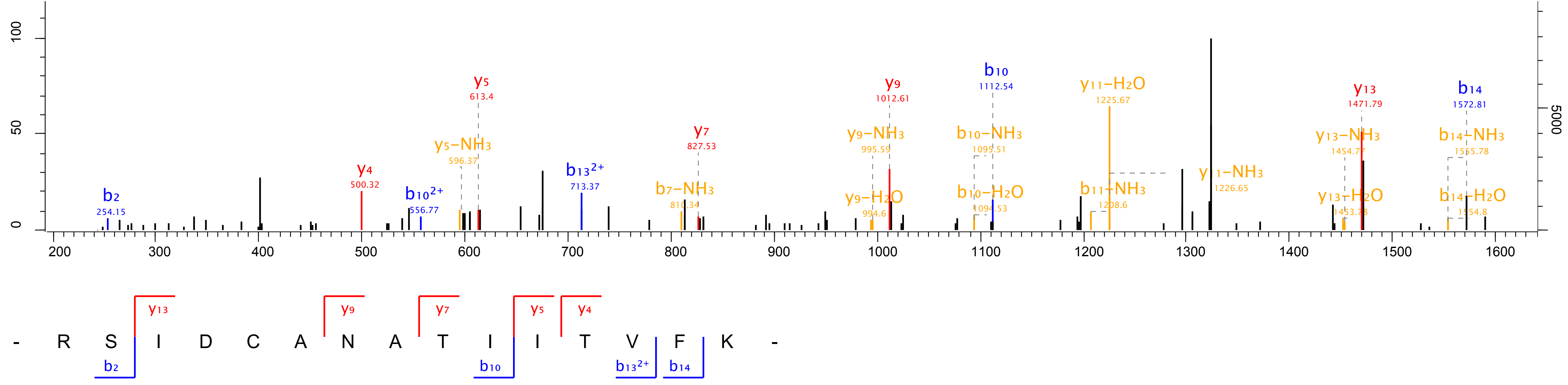

Raw file	Scan	Method	Score	m/z	Gene names
HBT_20130916_BV2_IL103_05	26976	ITMS; CID	65.23	643.38	Mtfr1l

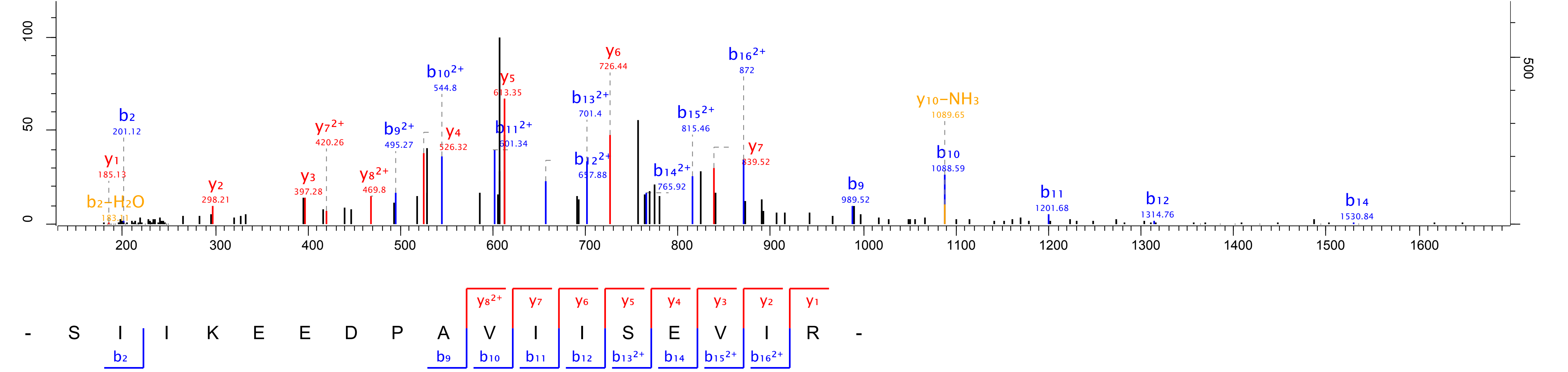

Raw file	Scan	Method	Score	m/z
HBT_20130916_BV2_IL103_05	13127	ITMS; CID	97.24	936

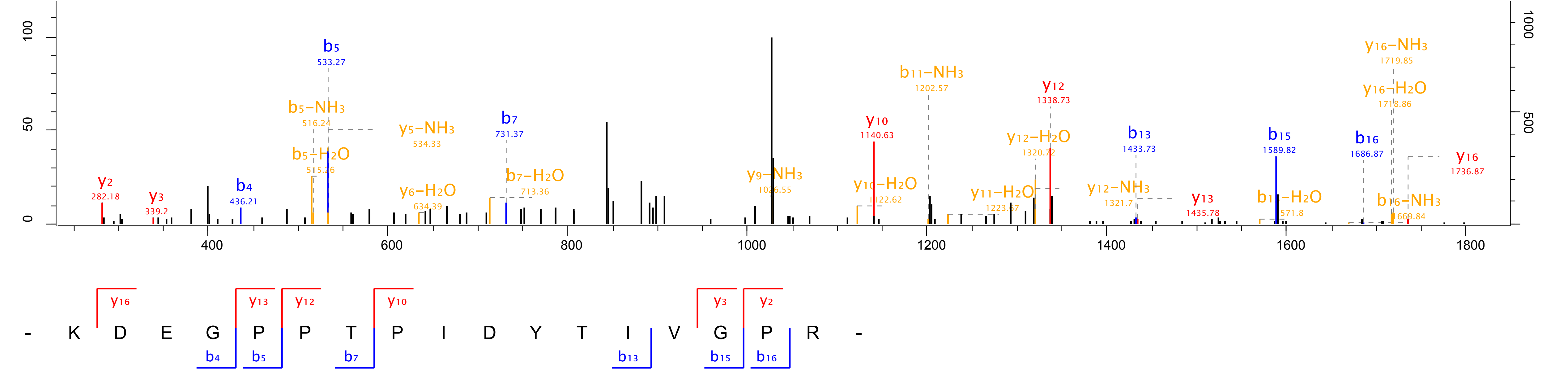

Raw file	Scan	Method	Score	m/z	Gene names
HBT_20130916_BV2_IL103_04	29602	ITMS; CID	109.04	767.38	S100a1

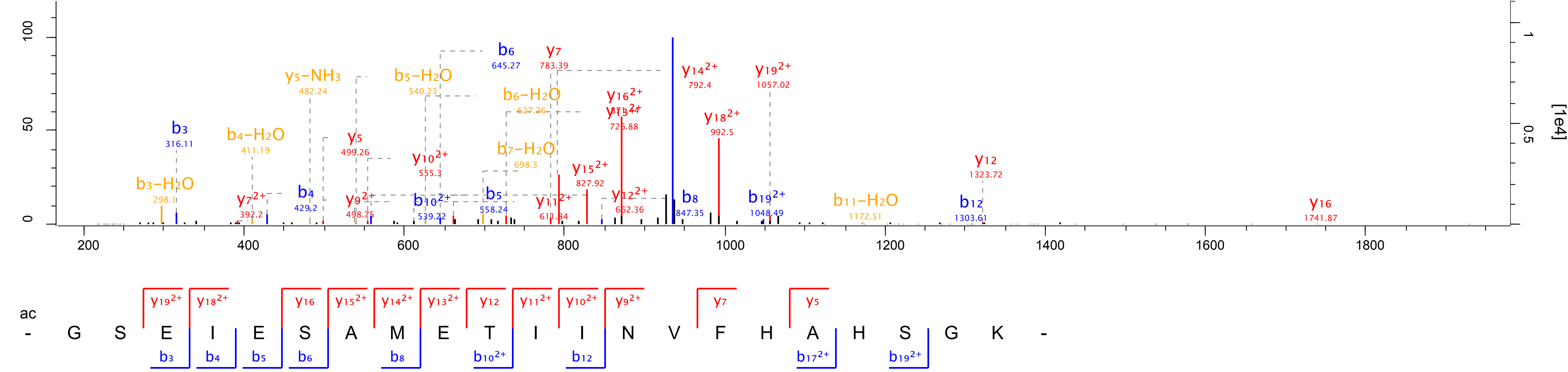

Raw file	Scan	Method	Score	m/z	Gene names
HBT_20130916_BV2_IL103_04	25868	ITMS; CID	64.57	648	Larp1b

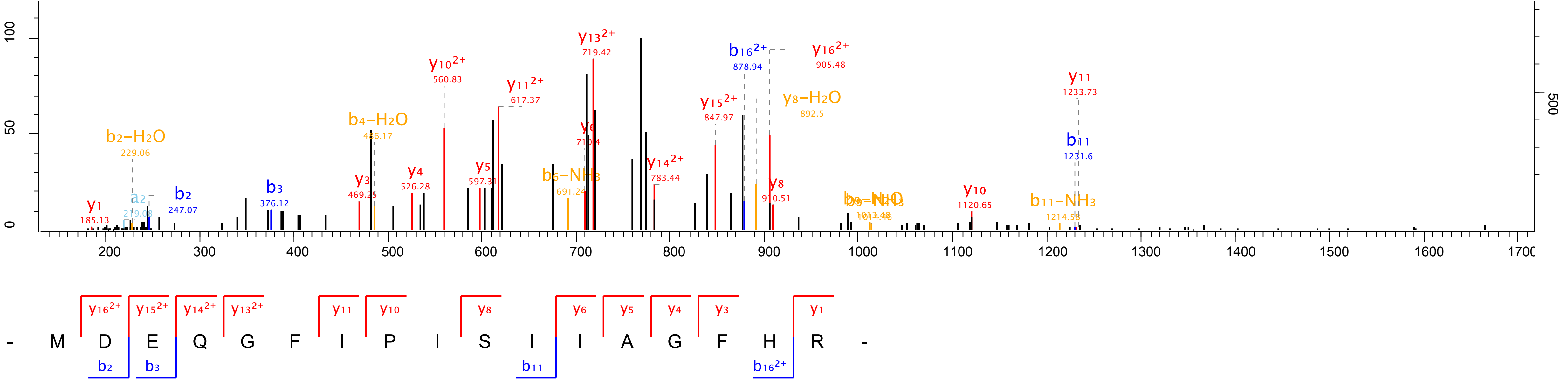

Raw file	Scan	Method	Score	m/z	Gene names
HBT_20130916_BV2_IL103_04	22905	ITMS; CID	75.38	703.35	Bbx

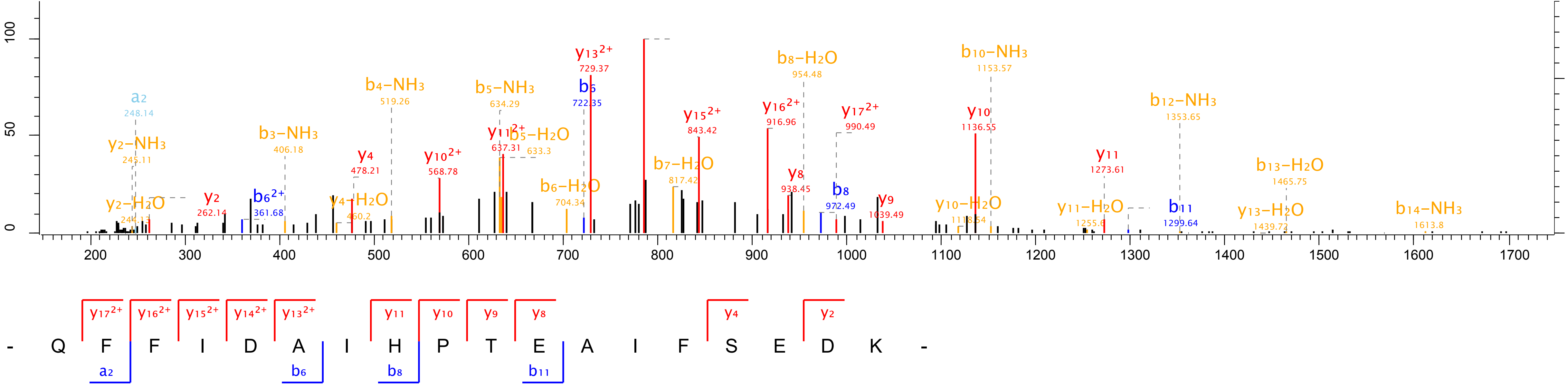

Raw file	Scan	Method	Score	m/z	Gene names
HBT_20130916_BV2_IL103_04	22461	ITMS; CID	63.68	708.4	Lrch3

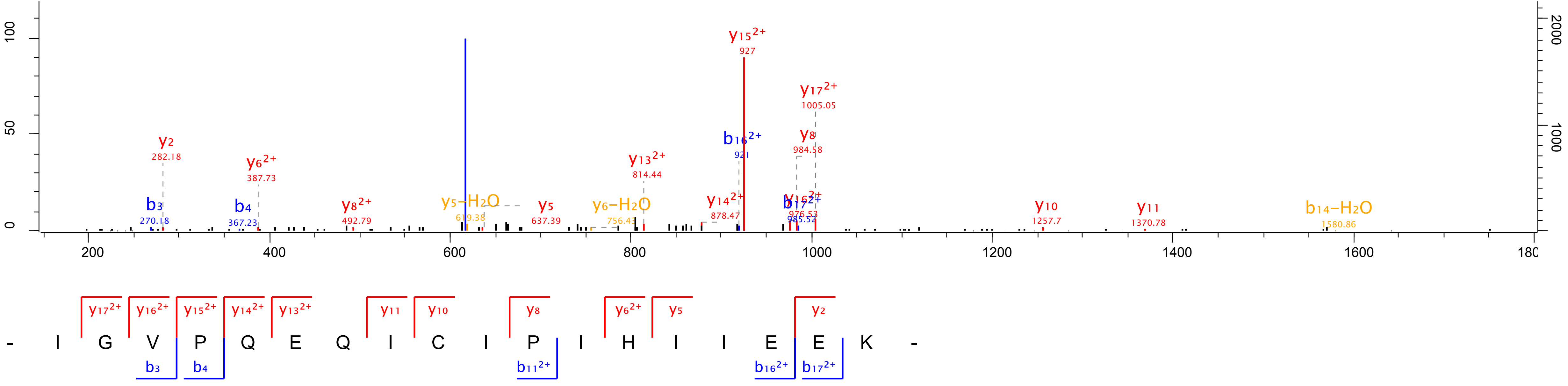

Raw file	Scan	Method	Score	m/z
HBT_20130916_BV2_IL103_04	21420	ITMS; CID	114.21	609.64

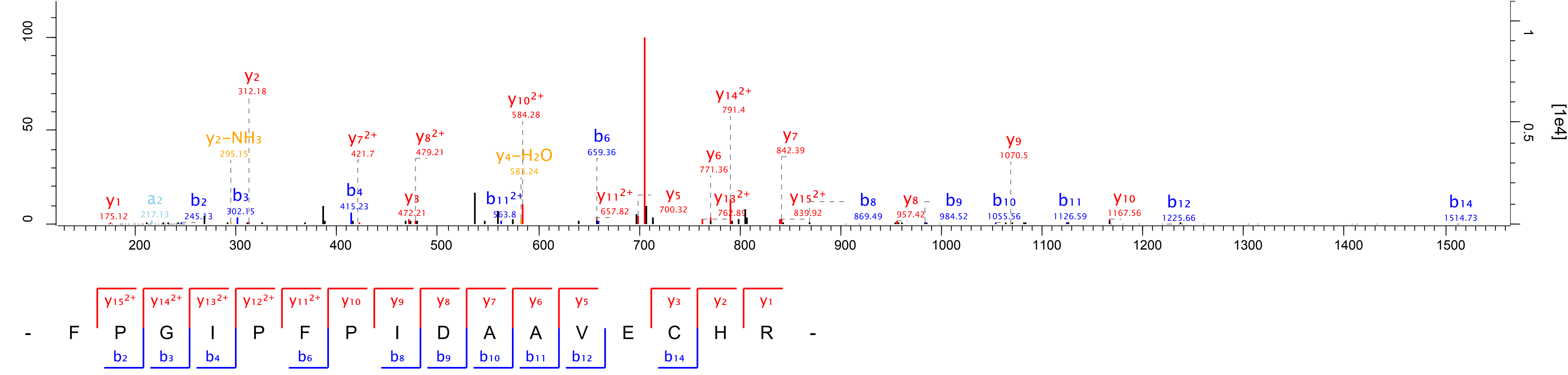

Raw file	Scan	Method	Score	m/z	Gene names
HBT_20130916_BV2_IL103_04	20408	ITMS; CID	97.46	566.29	Sbf1

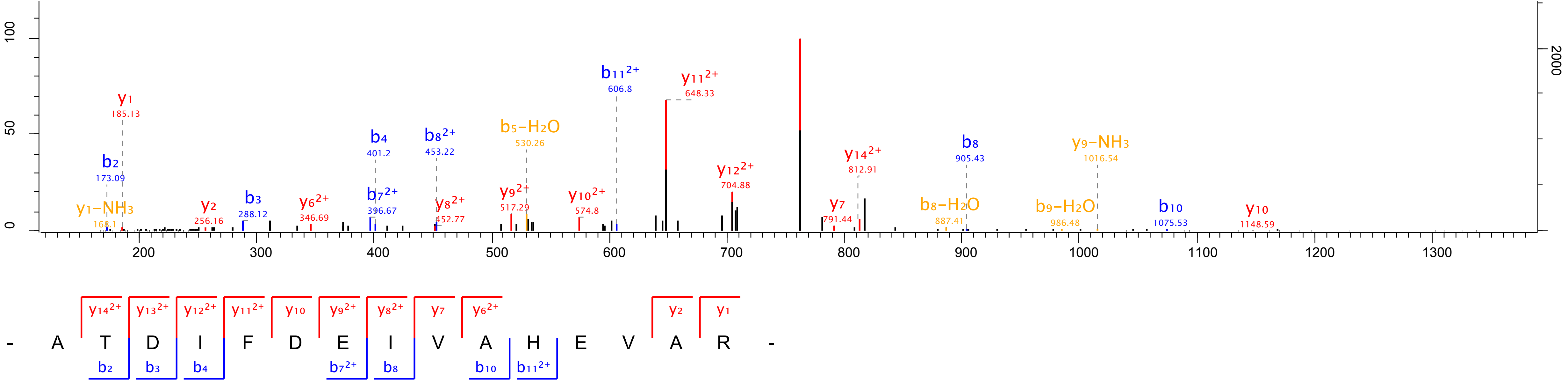

Raw file	Scan	Method	Score	m/z	Gene names
HBT_20130916_BV2_IL103_04	18131	ITMS; CID	59.59	818.46	Znrd1

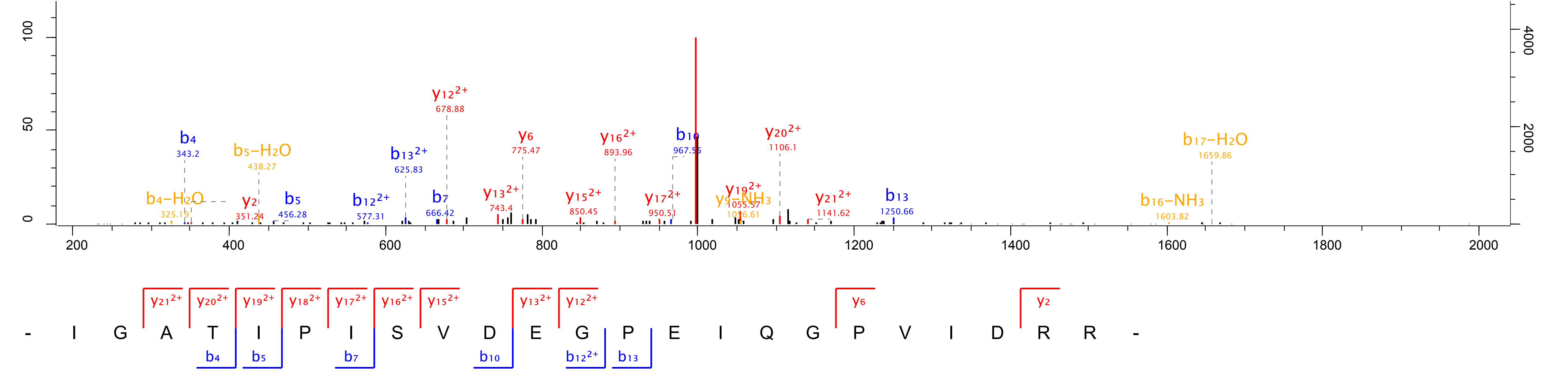

Raw file	Scan	Method	Score	m/z	Gene names
HBT_20130916_BV2_IL103_04	10913	ITMS; CID	238.51	598.33	mt-Atp8;Mtatp8

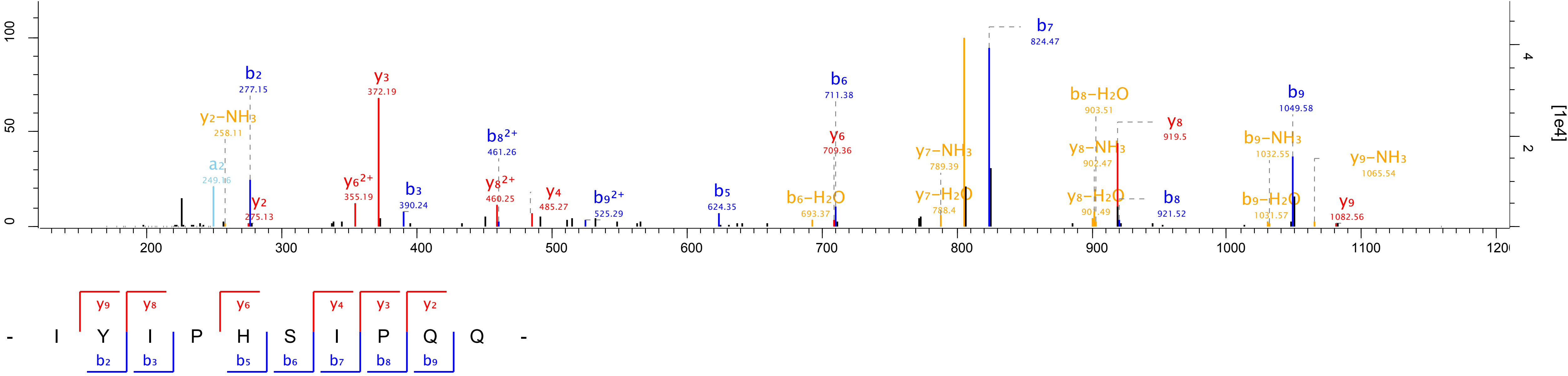

Raw file	Scan	Method	Score	m/z	Gene names
HBT_20130916_BV2_IL103_03	8242	ITMS; CID	86.91	574.32	Utp23

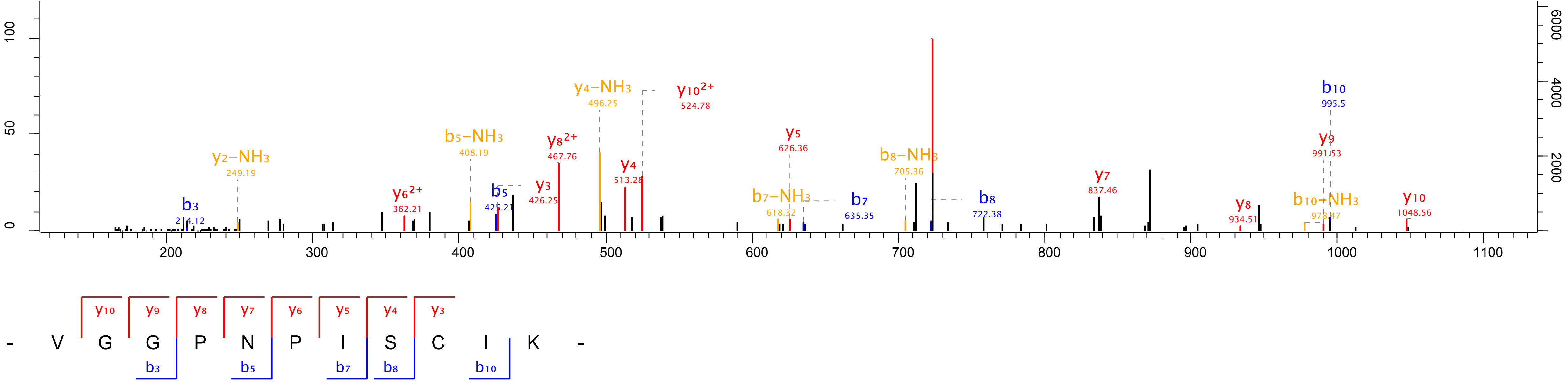

Raw file	Scan	Method	Score	m/z	Gene names
HBT_20130916_BV2_IL103_03	29176	ITMS; CID	102.69	1039.56	Zc3h7b

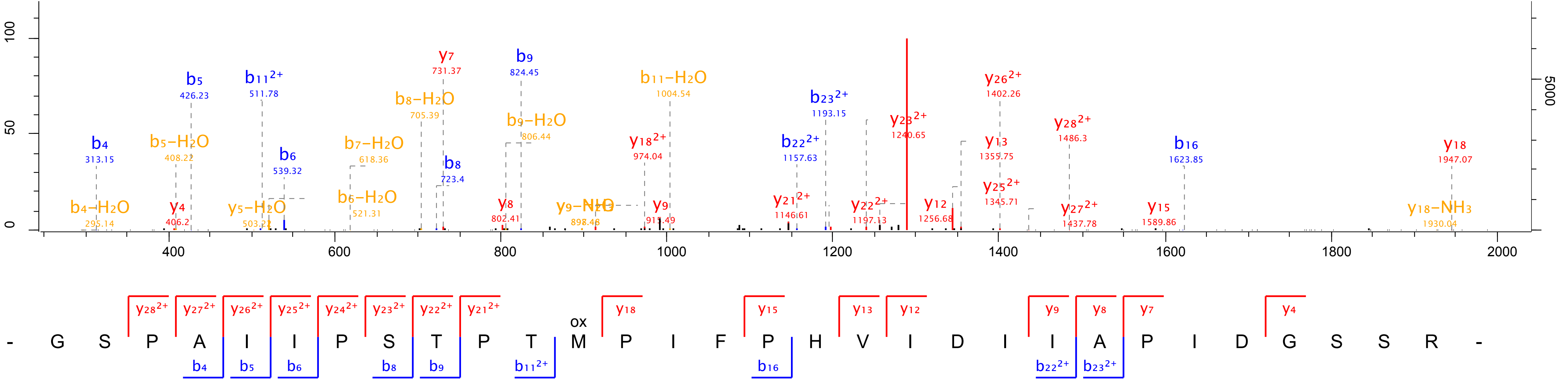

Raw file	Scan	Method	Score	m/z	Gene names
HBT_20130916_BV2_IL103_03	23944	ITMS; CID	87.15	623.35	Efr3b

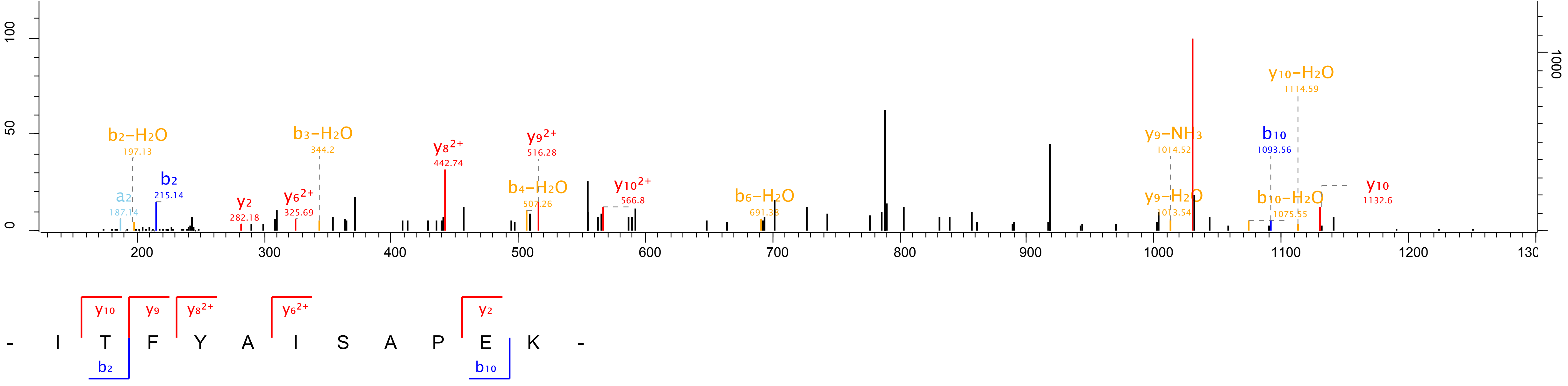

Raw file	Scan	Method	Score	m/z
HBT_20130916_BV2_IL103_03	20128	ITMS; CID	85.97	727.85

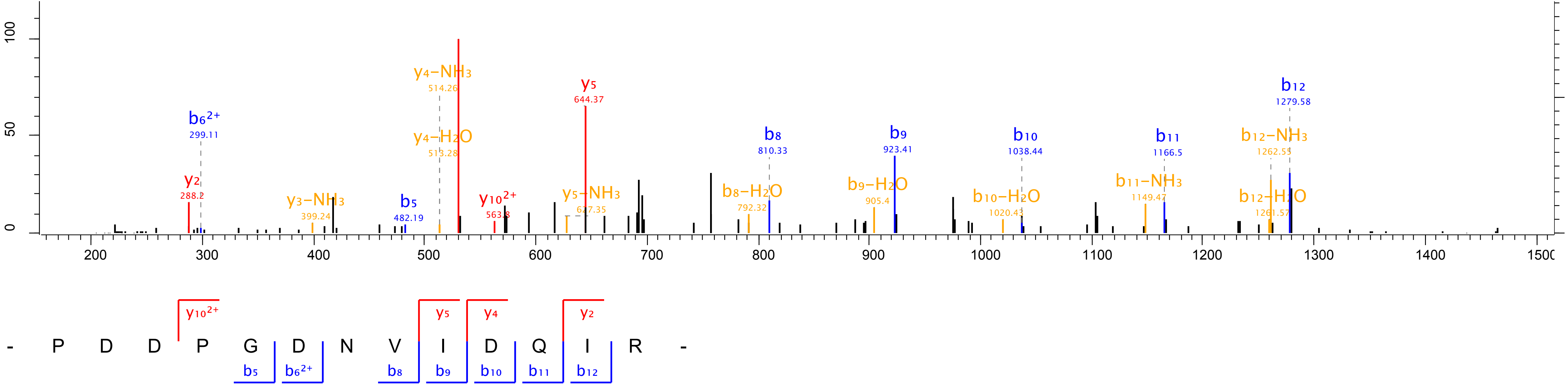

Raw file	Scan	Method	Score	m/z	Gene names
HBT_20130916_BV2_IL103_03	19118	ITMS; CID	135.8	594.33	Clec7a

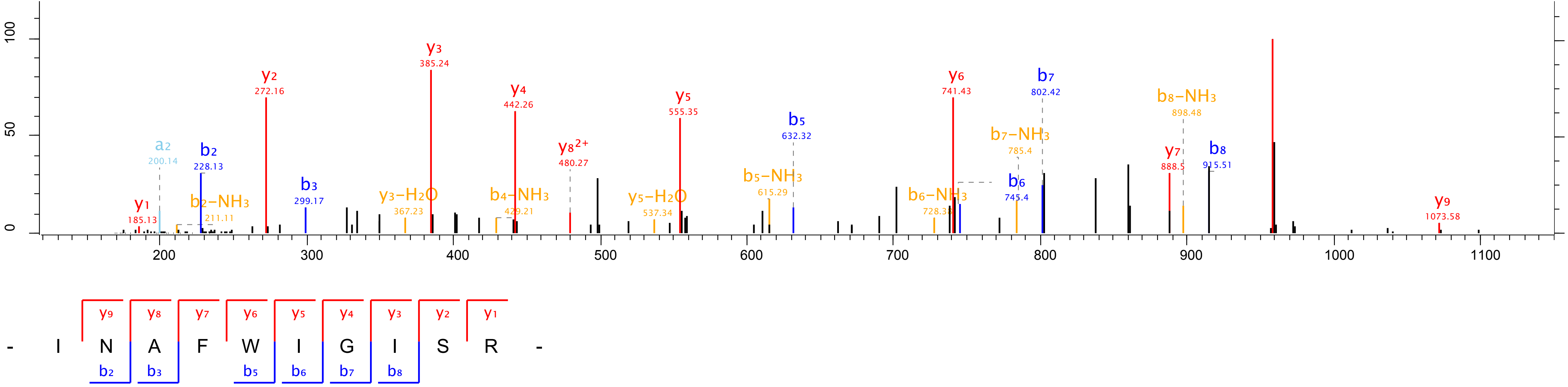

Raw file	Scan	Method	Score	m/z	Gene names
HBT_20130916_BV2_IL103_03	18681	ITMS; CID	152.96	712.89	Med27

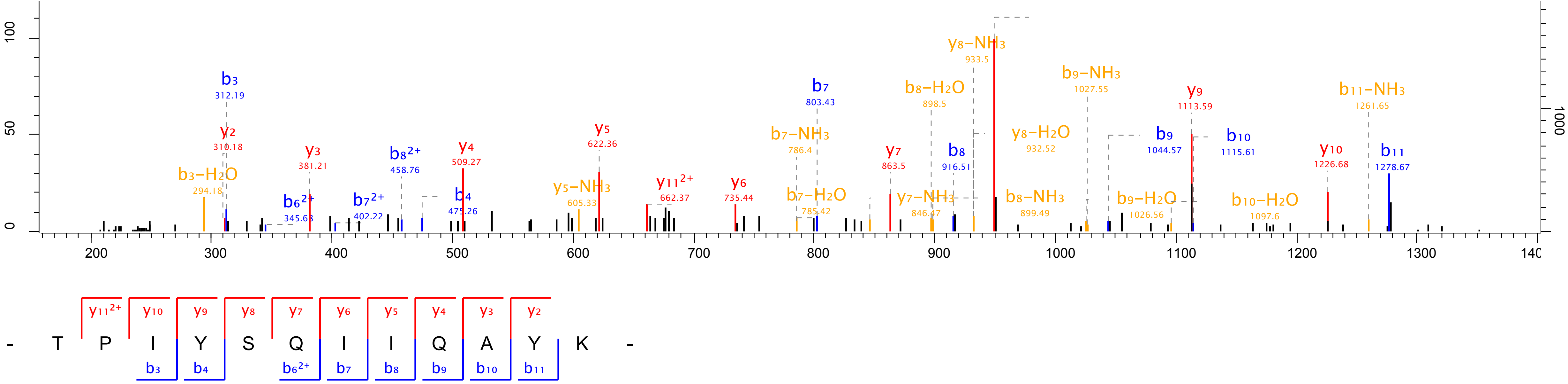

Raw file	Scan	Method	Score	m/z	Gene names
HBT_20130916_BV2_IL103_03	17247	ITMS; CID	137.95	590.37	Il2rg;Gm20489

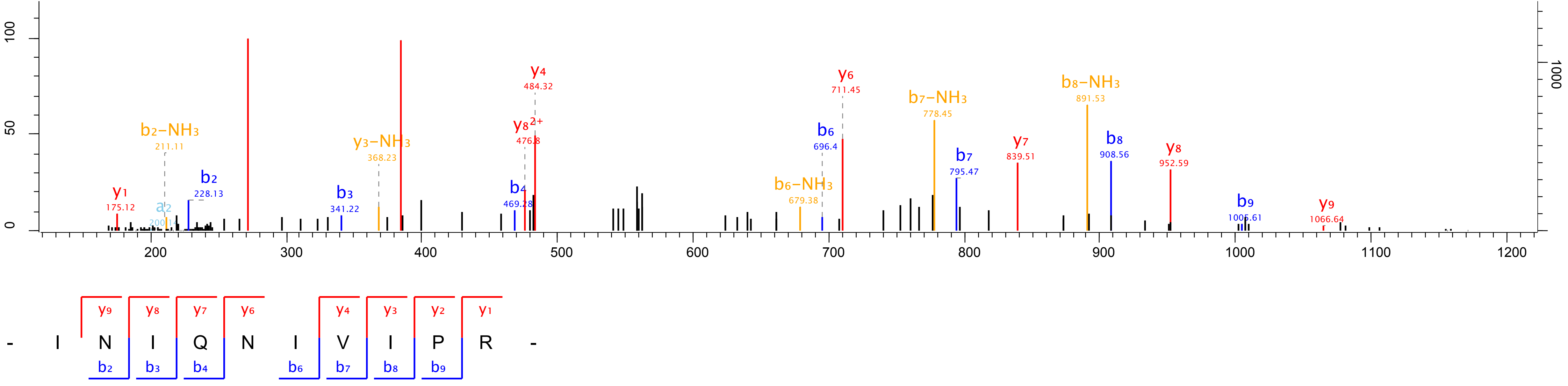

Raw file	Scan	Method	Score	m/z	Gene names
HBT_20130916_BV2_IL103_03	15891	ITMS; CID	117.14	713.35	Ndufa1

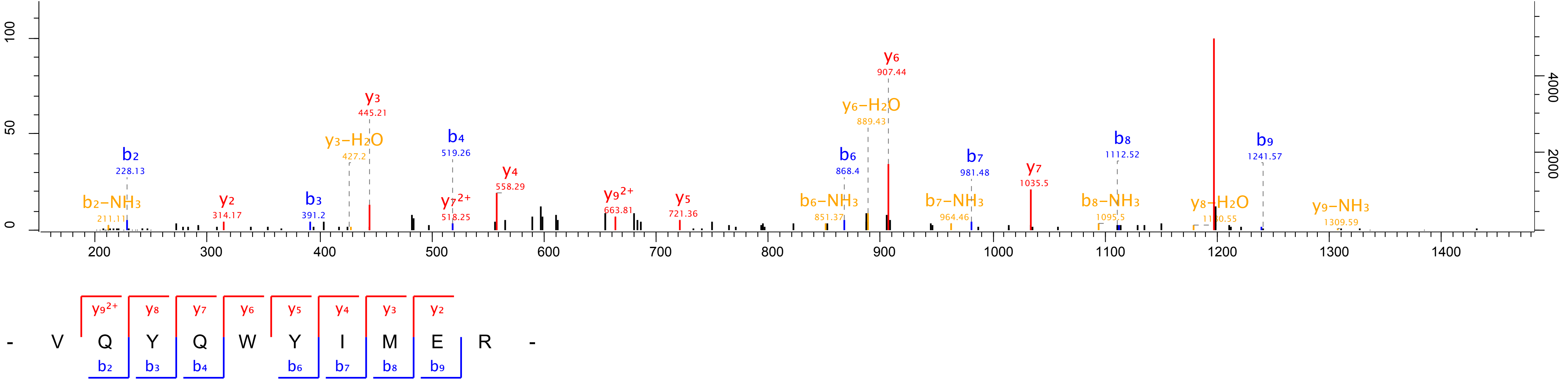

Raw file	Scan	Method	Score	m/z	Gene names
HBT_20130916_BV2_IL103_03	14924	ITMS; CID	138.76	607.85	Usf2;Usf1

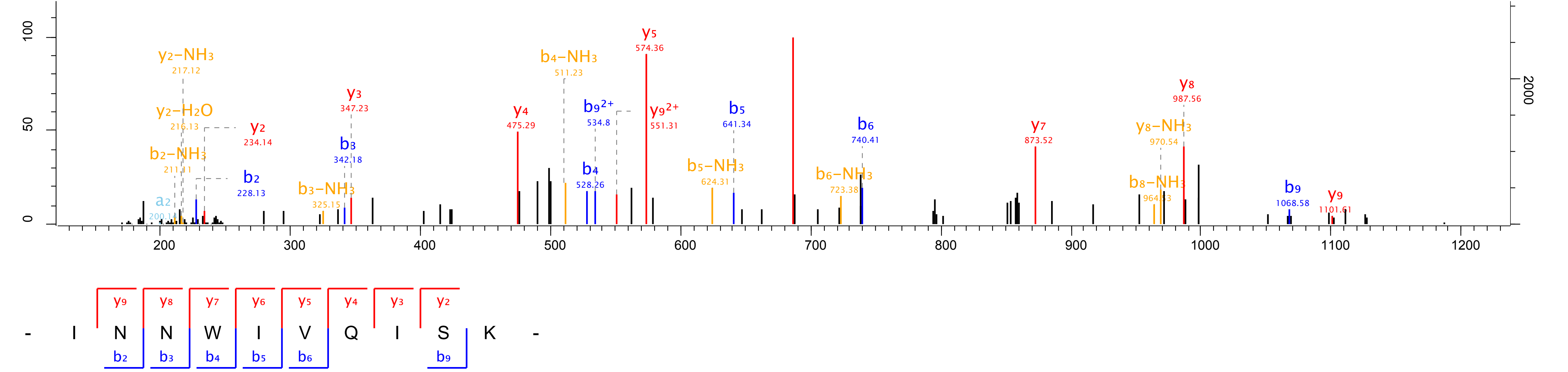

Raw file	Scan	Method	Score	m/z	Gene names
HBT_20130916_BV2_IL103_02	3833	ITMS; CID	103.97	677.84	Tox3;Tox;Tox4;Gm5828

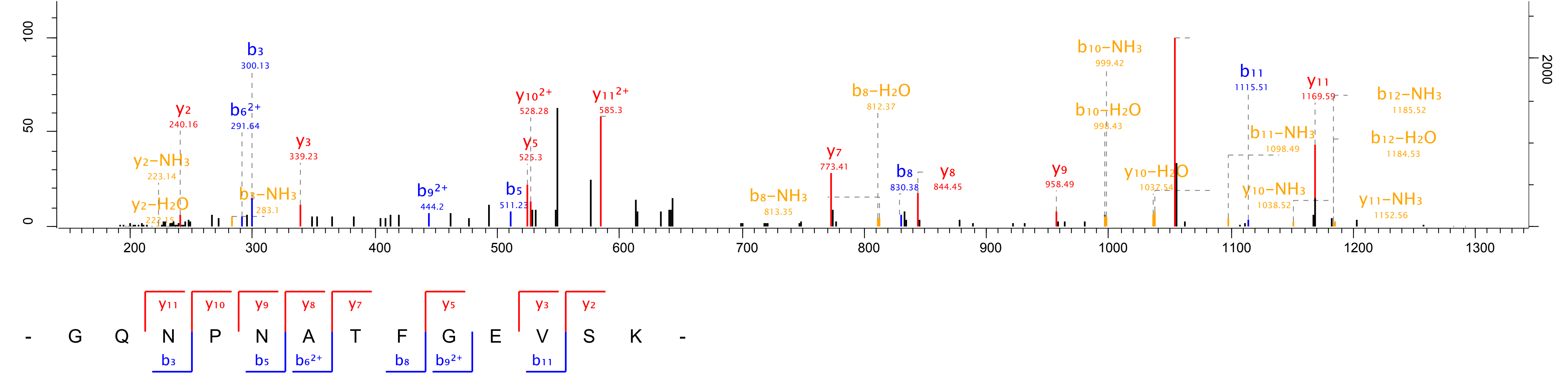

Raw file	Scan	Method	Score	m/z	Gene names
HBT_20130916_BV2_IL103_02	29827	ITMS; CID	145.73	1376.74	Znf668

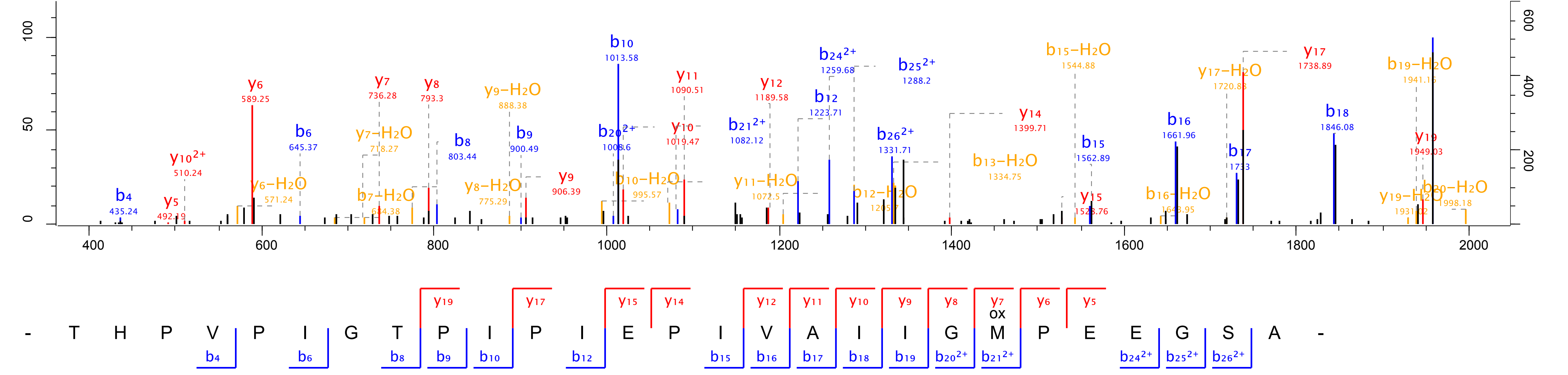

Raw file	Scan	Method	Score	m/z	Gene names
HBT_20130916_BV2_IL103_02	27438	ITMS; CID	97.73	722.91	Lpgat1

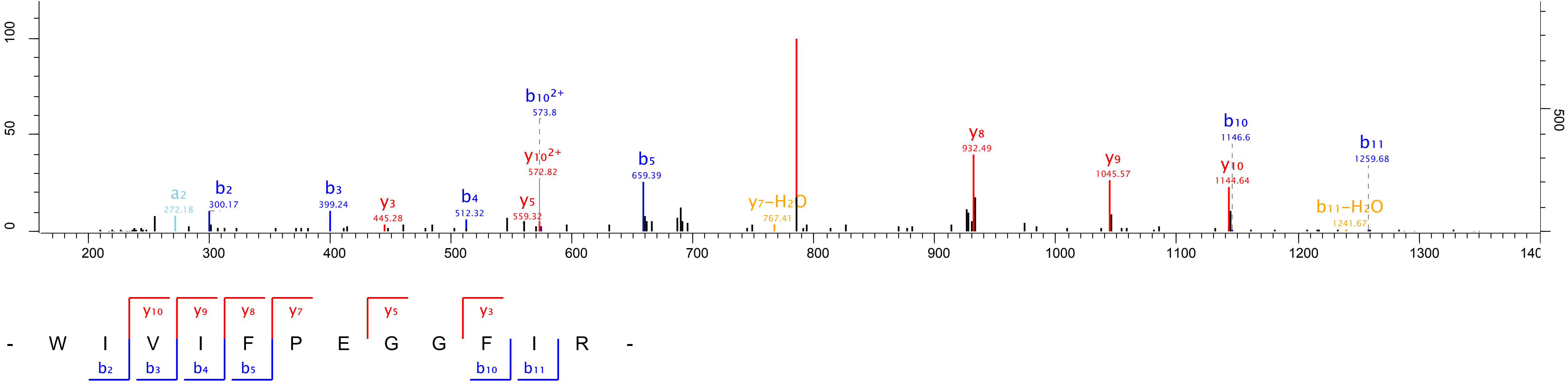

Raw file	Scan	Method	Score	m/z	Gene names
HBT_20130916_BV2_IL103_02	25246	ITMS; CID	145.7	933.44	G6pc3

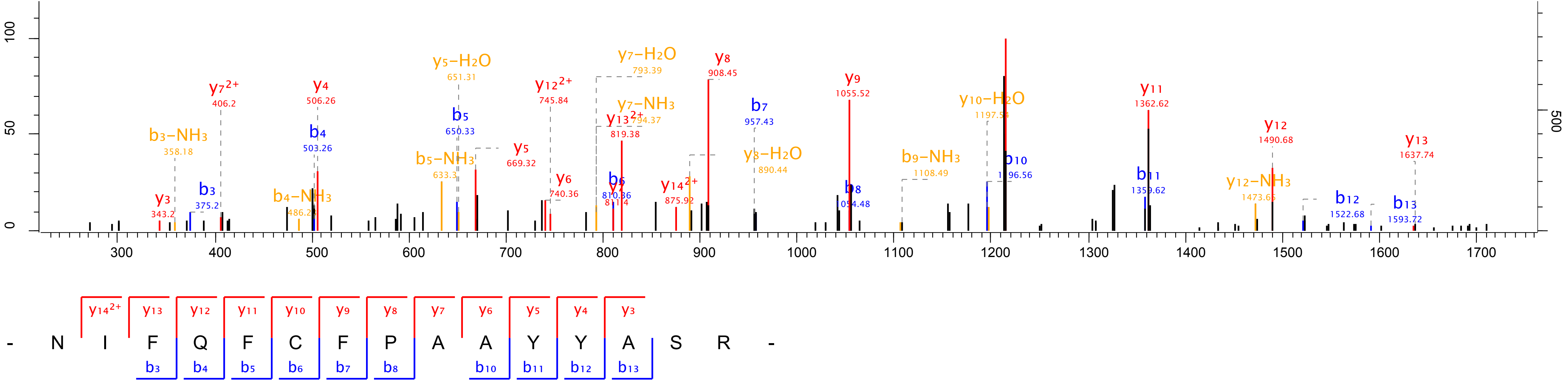

Raw file	Scan	Method	Score	m/z	Gene names
HBT_20130916_BV2_IL103_02	21441	ITMS; CID	104.07	594.34	Trappc6a

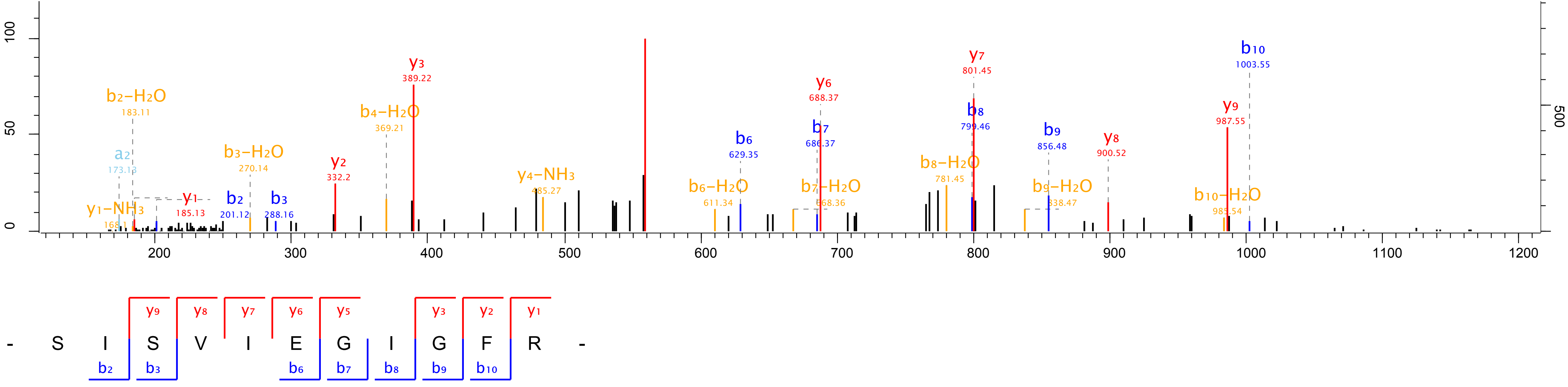

Raw file	Scan	Method	Score	m/z	Gene names
HBT_20130916_BV2_IL103_02	20782	ITMS; CID	124.2	897.98	Ccdc59

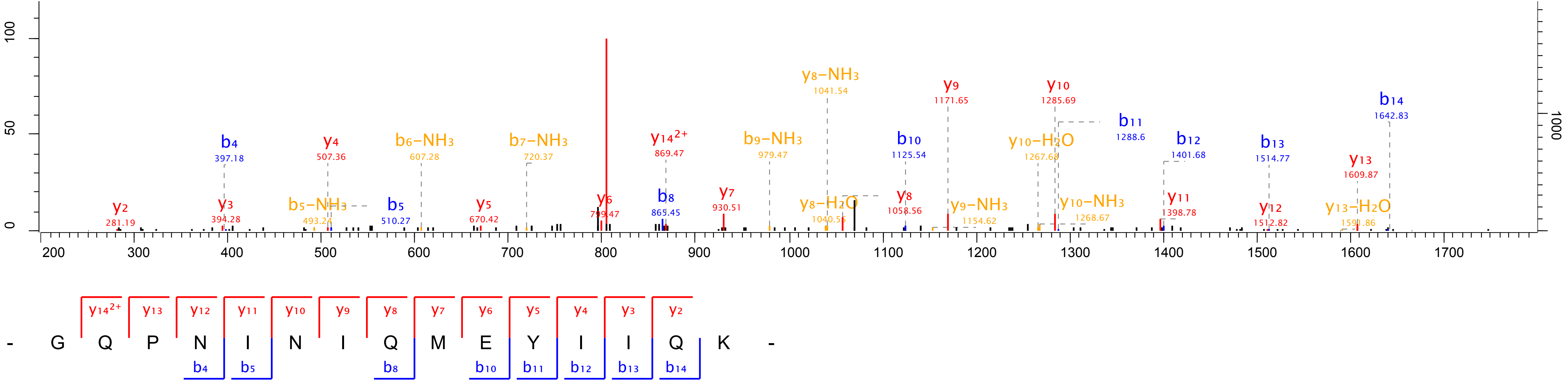

Raw file	Scan	Method	Score	m/z	Gene names
HBT_20130916_BV2_IL103_02	14622	ITMS; CID	76.73	757.9	Isca1;AK157302

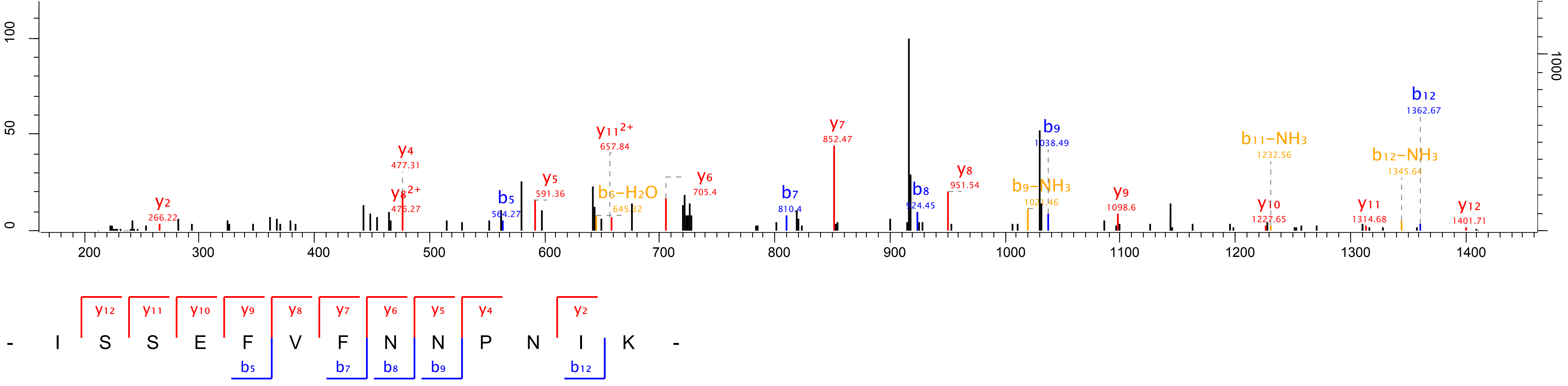

Raw file	Scan	Method	Score	m/z
HBT_20130916_BV2_IL103_01	2736	ITMS; CID	89.83	613.82

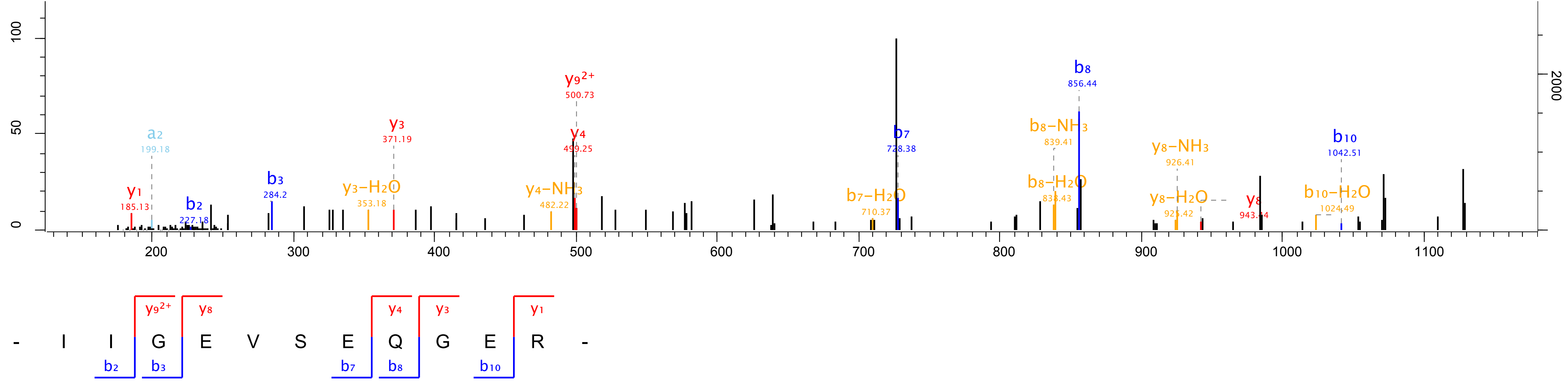

Raw file	Scan	Method	Score	m/z	Gene names
HBT_20130916_BV2_IL103_01	2005	ITMS; CID	103.43	634.78	Cr1l

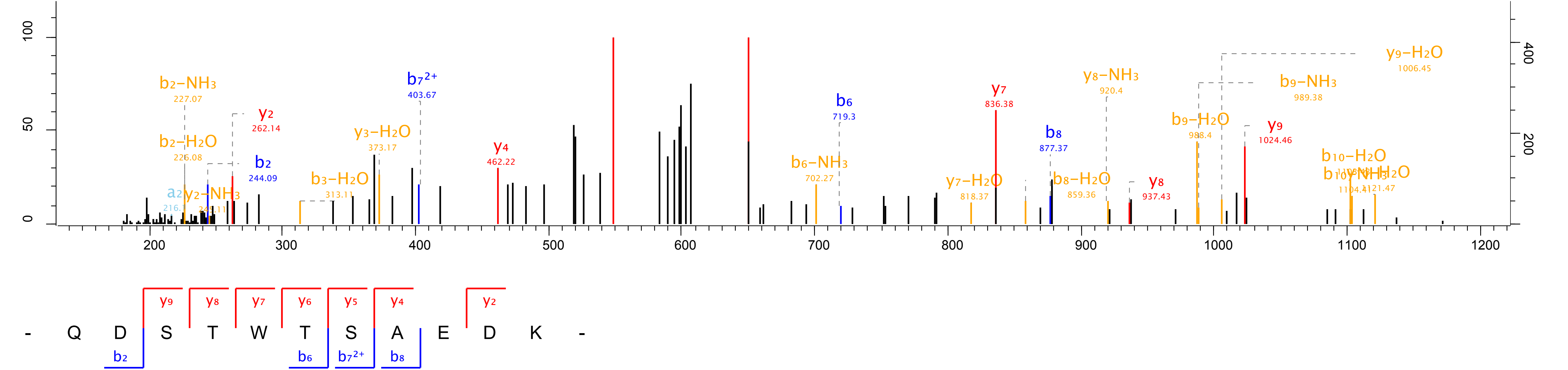

Raw file	Scan	Method	Score	m/z	Gene names
HBT_20130916_BV2_IL103_01	19828	ITMS; CID	62.58	1327.71	Atf1

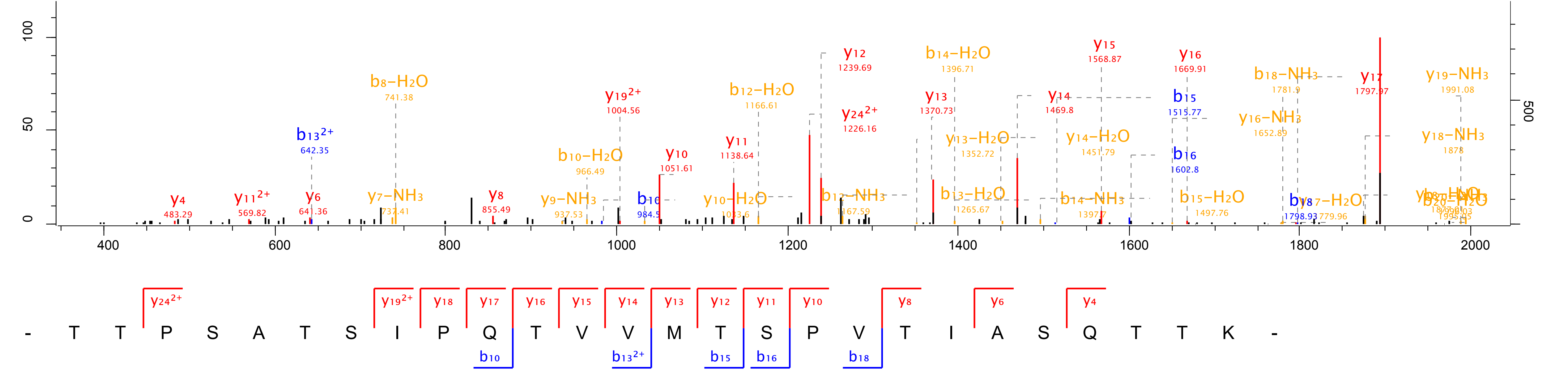

Raw file	Scan	Method	Score	m/z	Gene names
HBT_20130916_BV2_IL103_01	15406	ITMS; CID	145.88	1048.5	Trem2

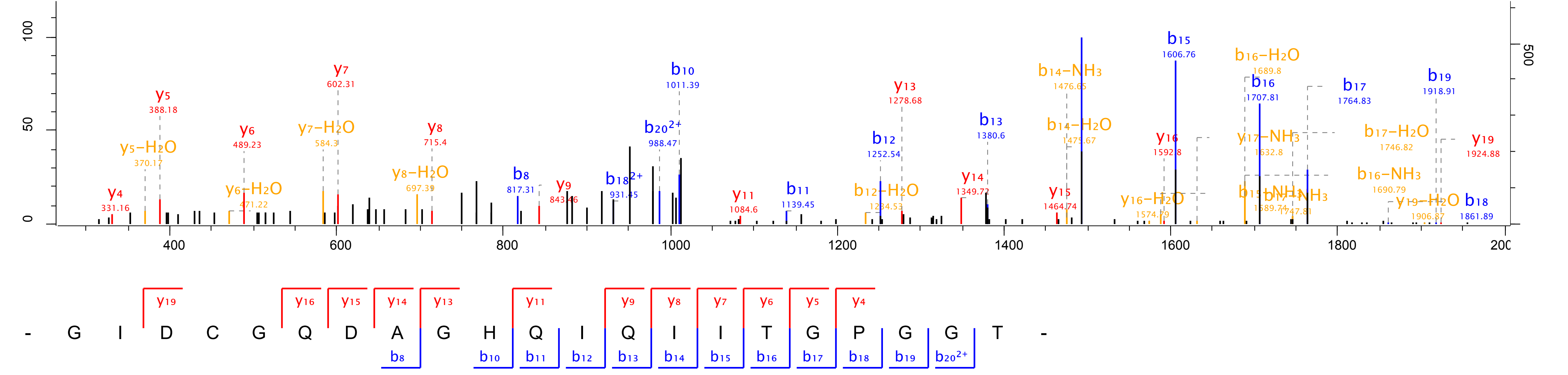

Raw file	Scan	Method	Score	m/z	Gene names
HBT_20130916_BV2_IL103_01	1373	ITMS; CID	76.73	656.8	Rnf149

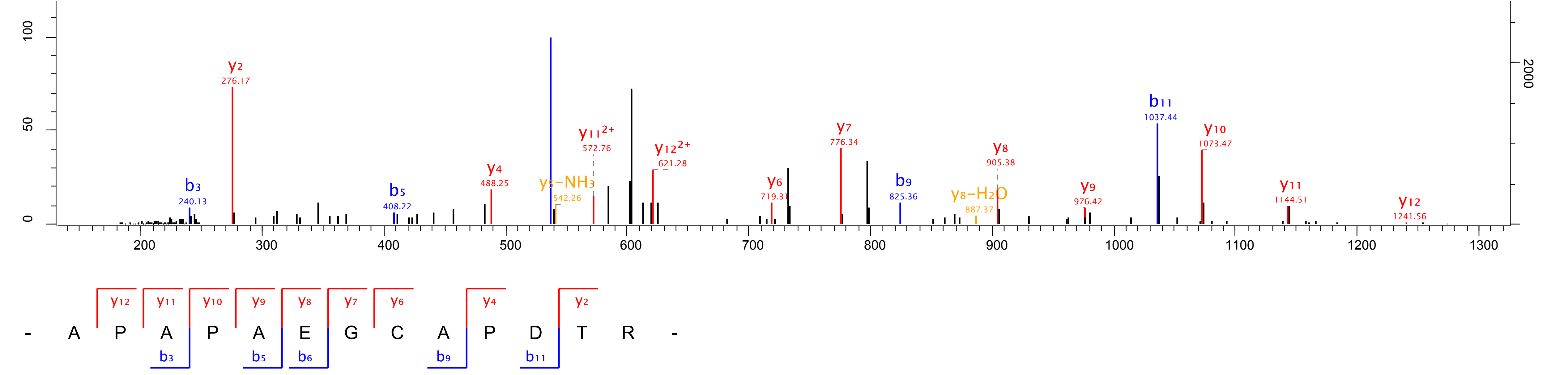

Raw file	Scan	Method	Score	m/z	Gene names
HBT_20130916_BV2_IL103_01	1243	ITMS; CID	108.9	614.3	Cox20

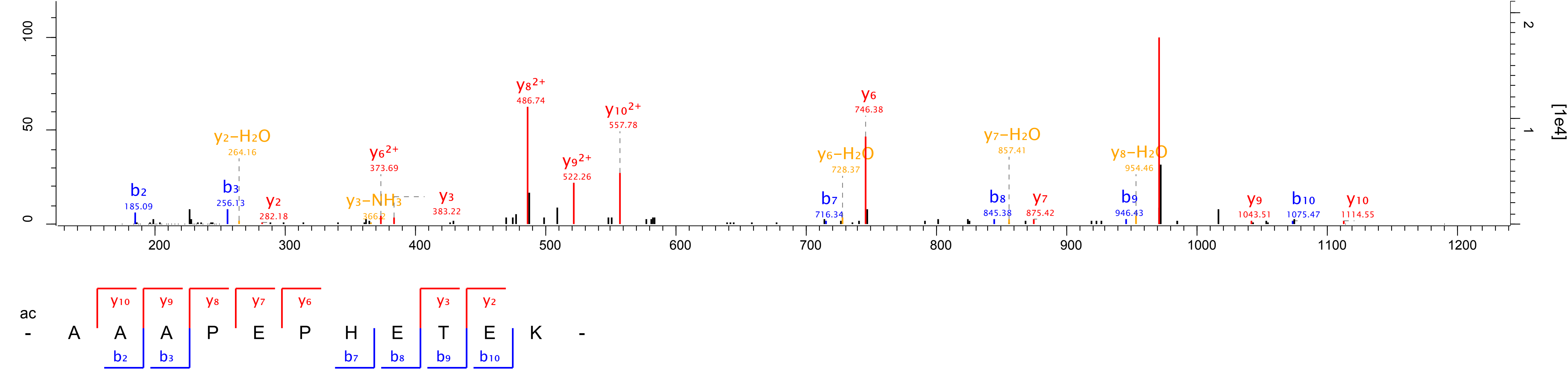

Raw file	Scan	Method	Score	m/z	Gene names
HBT_20130916_BV2_IL102_06	1040	ITMS; CID	155.66	800.92	mt-Co3;Mtco3

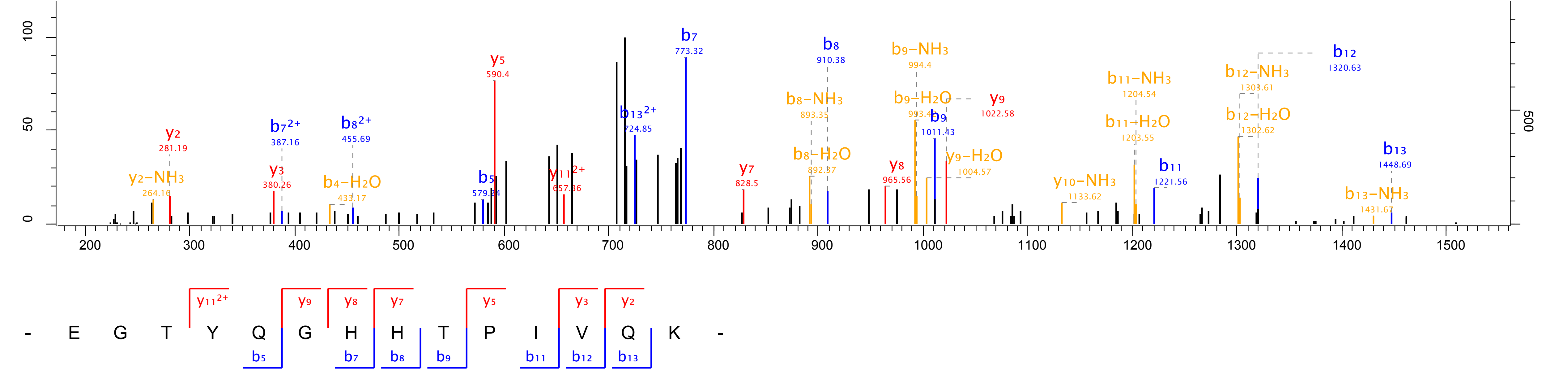

Raw file	Scan	Method	Score	m/z	Gene names
HBT_20130916_BV2_IL102_05	4457	ITMS; CID	119.88	754.86	Tmem41b

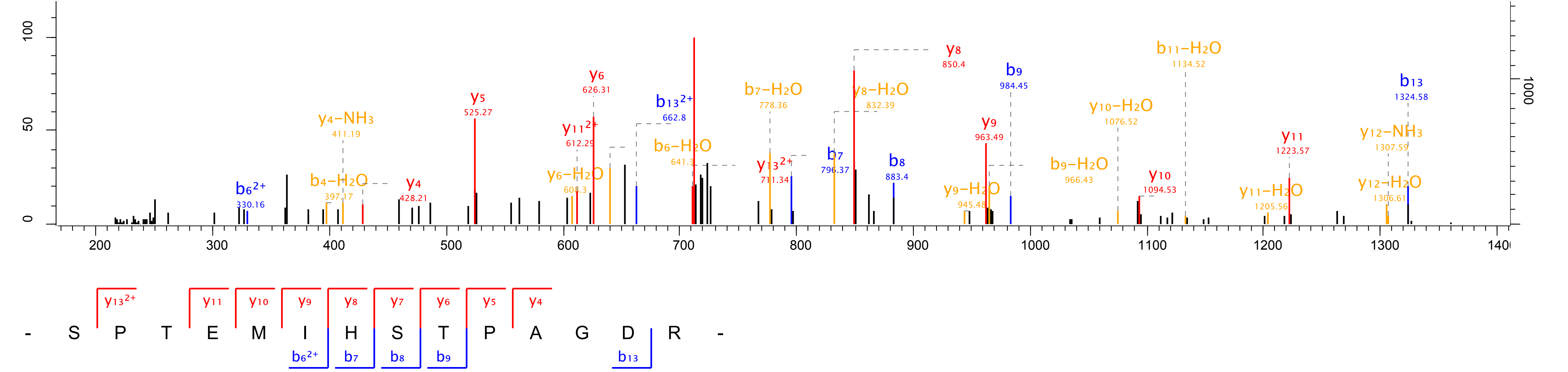

Raw file	Scan	Method	Score	m/z	Gene names
HBT_20130916_BV2_IL102_05	27106	ITMS; CID	76.16	677.72	Asnsd1

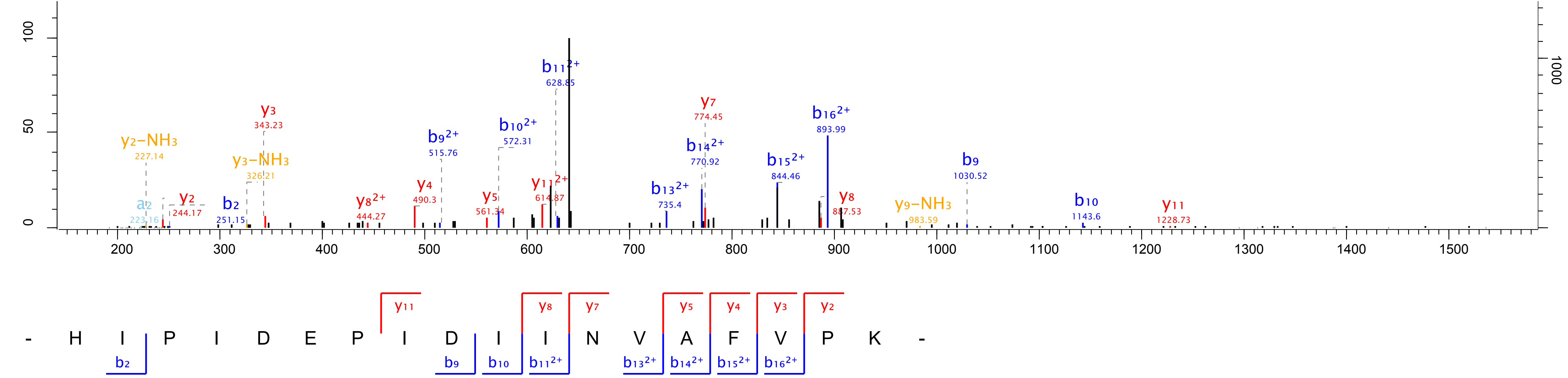

Raw file	Scan	Method	Score	m/z	Gene names
HBT_20130916_BV2_IL102_05	14106	ITMS; CID	177.66	1104.49	Elof1

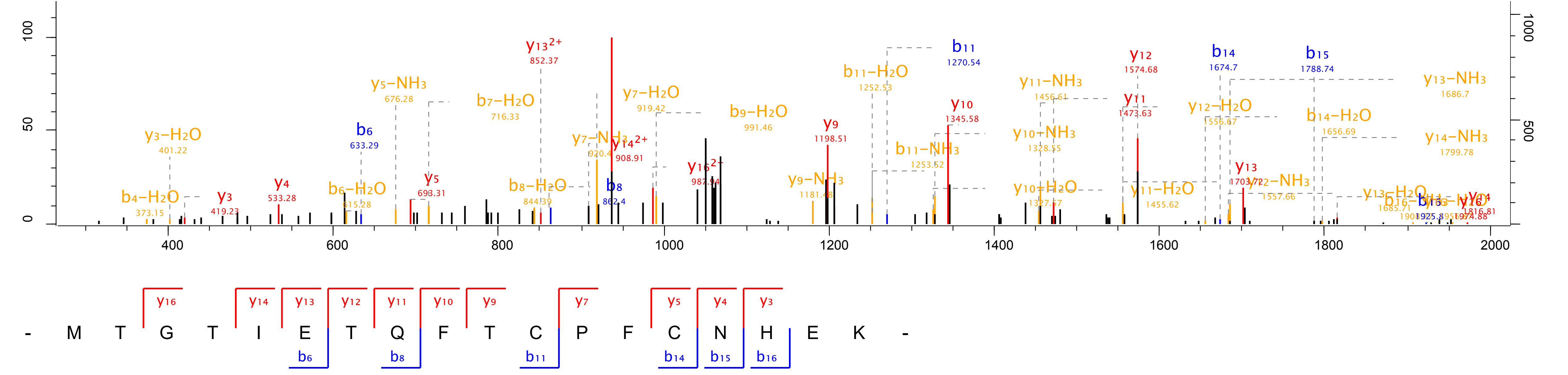

Raw file	Scan	Method	Score	m/z	Gene names
HBT_20130916_BV2_IL102_05	12570	ITMS; CID	89.14	913.89	Mrpl35

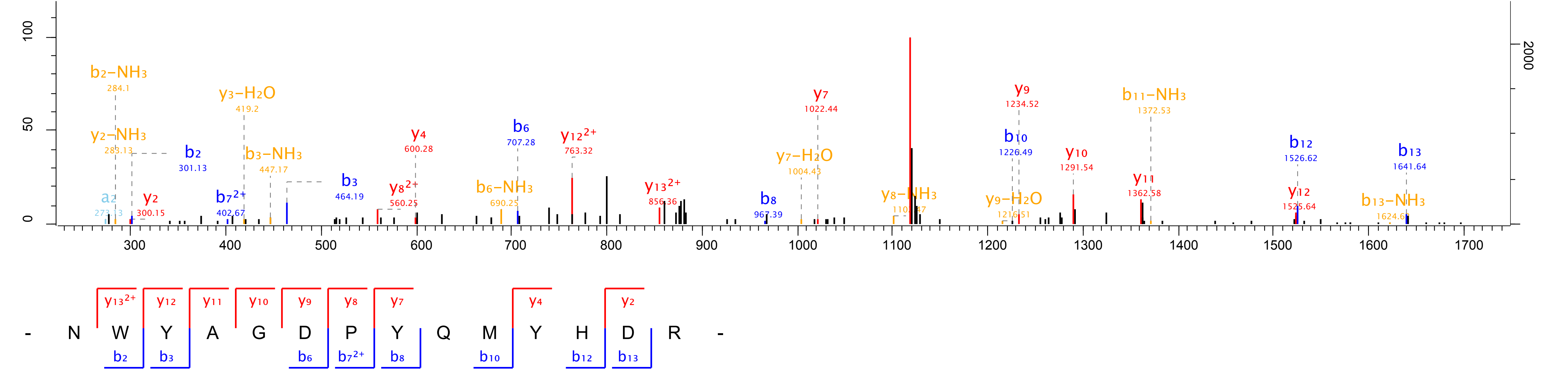

Raw file	Scan	Method	Score	m/z	Gene names
HBT_20130916_BV2_IL102_04	7893	ITMS; CID	154.13	777.88	Psmg4

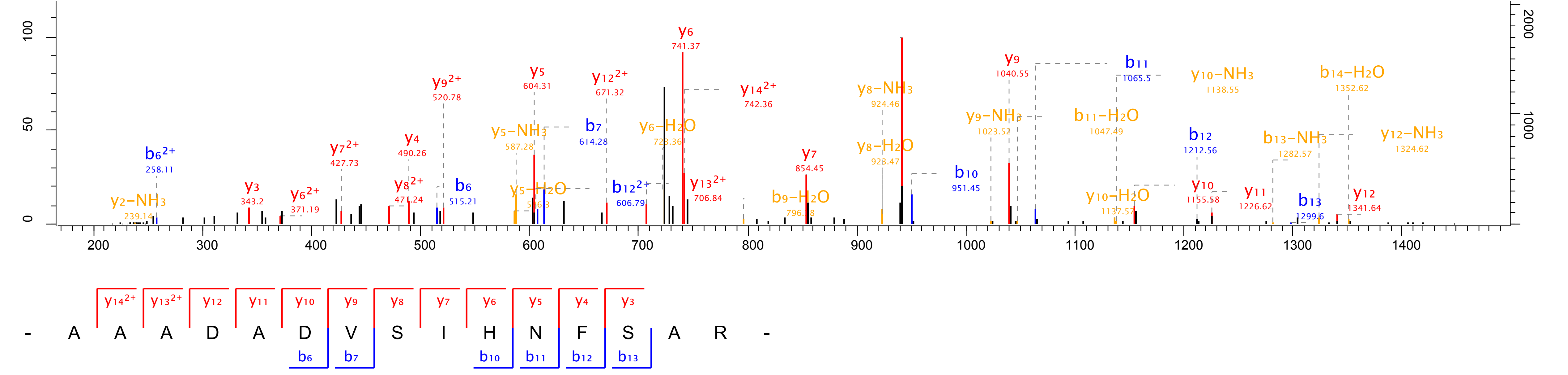

Raw file	Scan	Method	Score	m/z	Gene names
HBT_20130916_BV2_IL102_04	27406	ITMS; CID	55.16	1084.9	Nanp

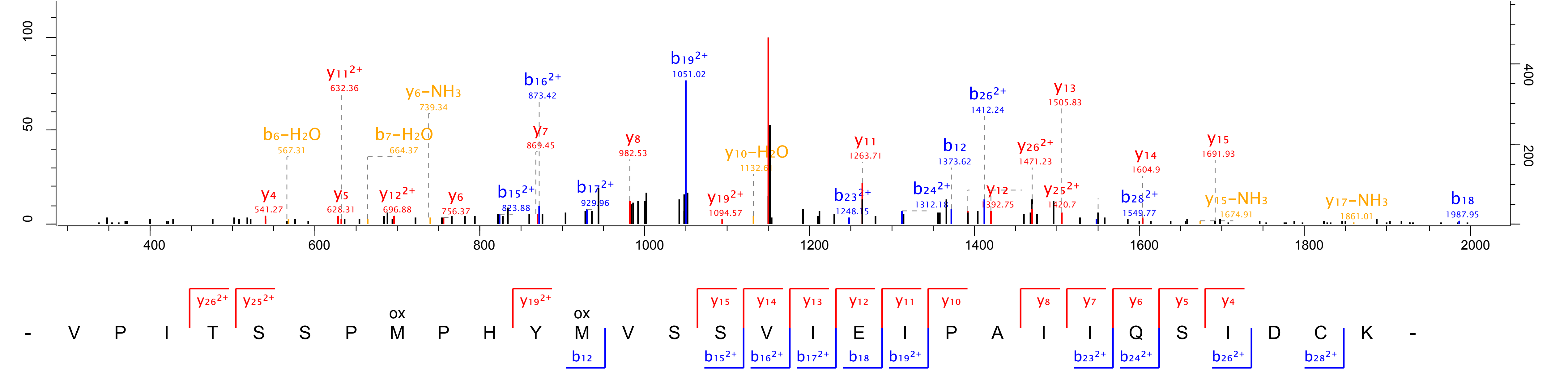

Raw file	Scan	Method	Score	m/z	Gene names
HBT_20130916_BV2_IL102_04	23880	ITMS; CID	62.69	600.69	Fam211a

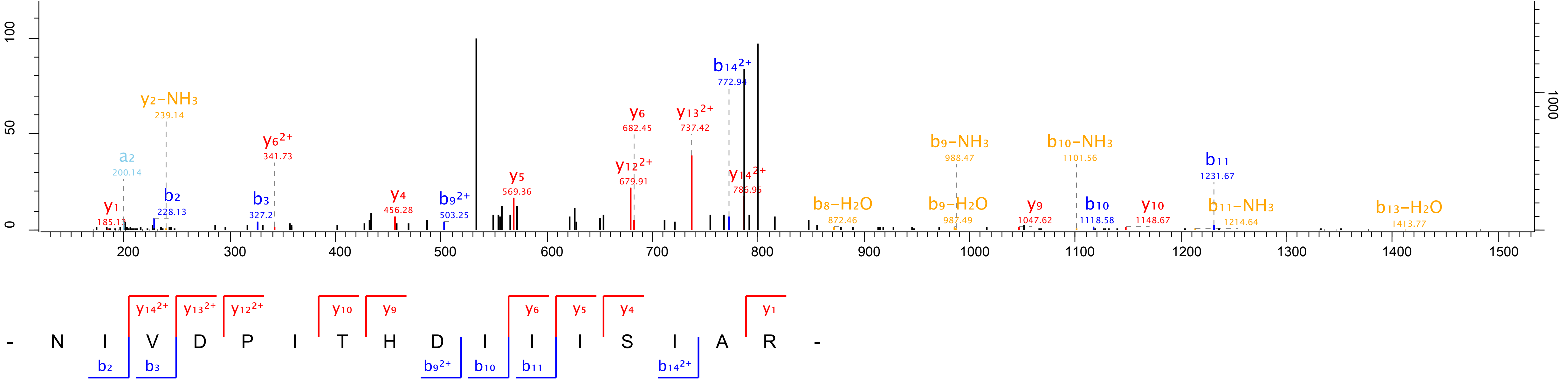

Raw file	Scan	Method	Score	m/z	Gene names
HBT_20130916_BV2_IL102_04	17517	ITMS; CID	100.11	541.66	Tfb1m

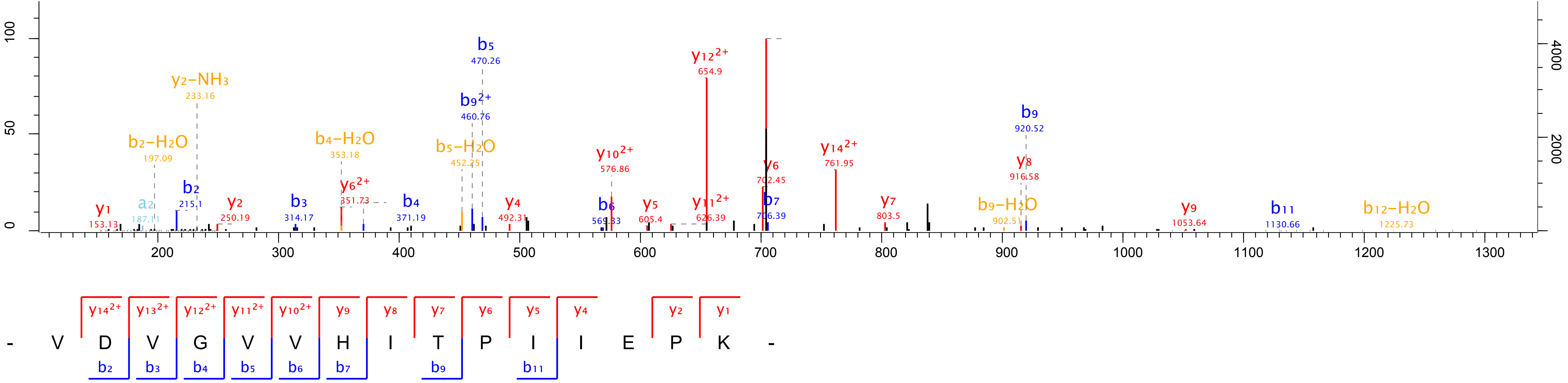

Raw file	Scan	Method	Score	m/z	Gene names
HBT_20130916_BV2_IL102_03	5321	ITMS; CID	90.7	645.28	Adam15

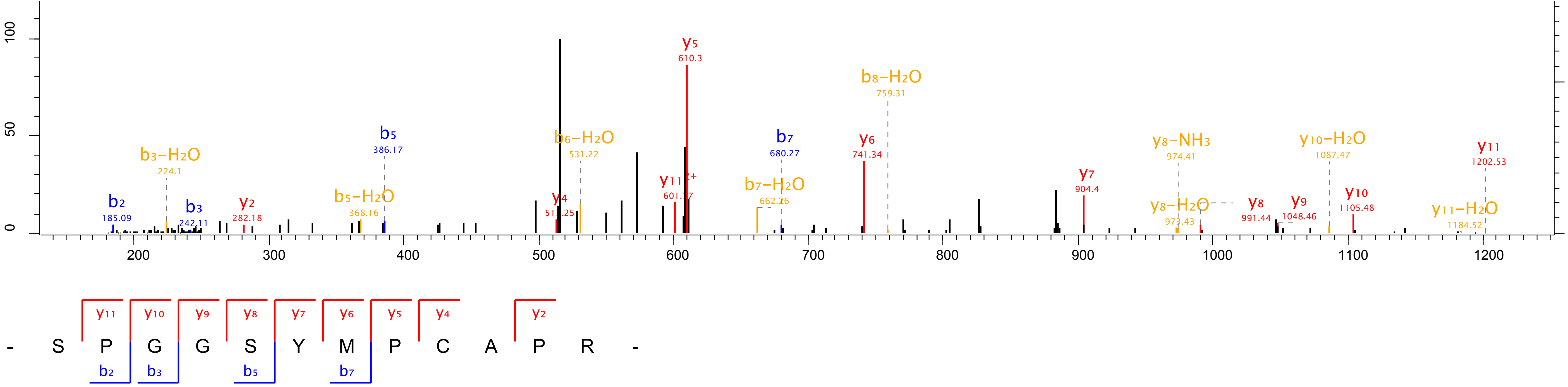

Raw file	Scan	Method	Score	m/z	Gene names
HBT_20130916_BV2_IL102_03	26360	ITMS; CID	119.46	738.37	Zc3h13

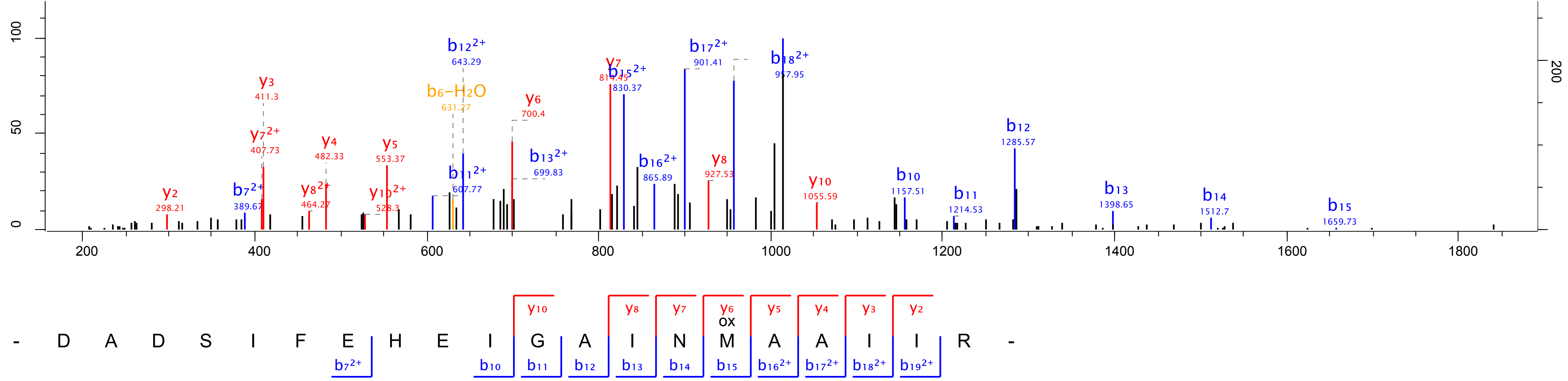

Raw file	Scan	Method	Score	m/z	Gene names
HBT_20130916_BV2_IL102_03	19259	ITMS; CID	133.23	639.36	Klhdc10

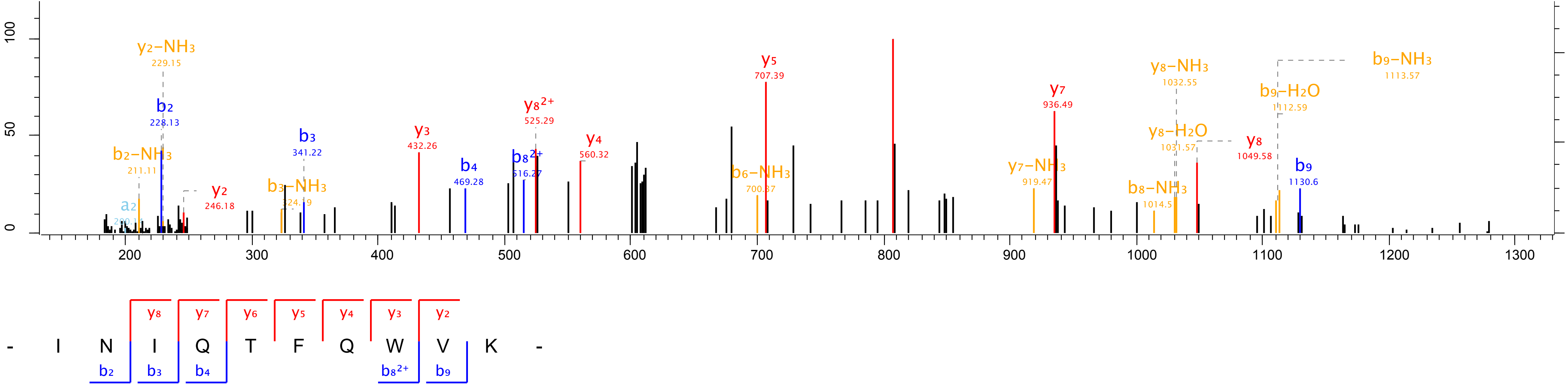

Raw file	Scan	Method	Score	m/z	Gene names
HBT_20130916_BV2_IL102_03	16167	ITMS; CID	134.37	692.86	Fam89b

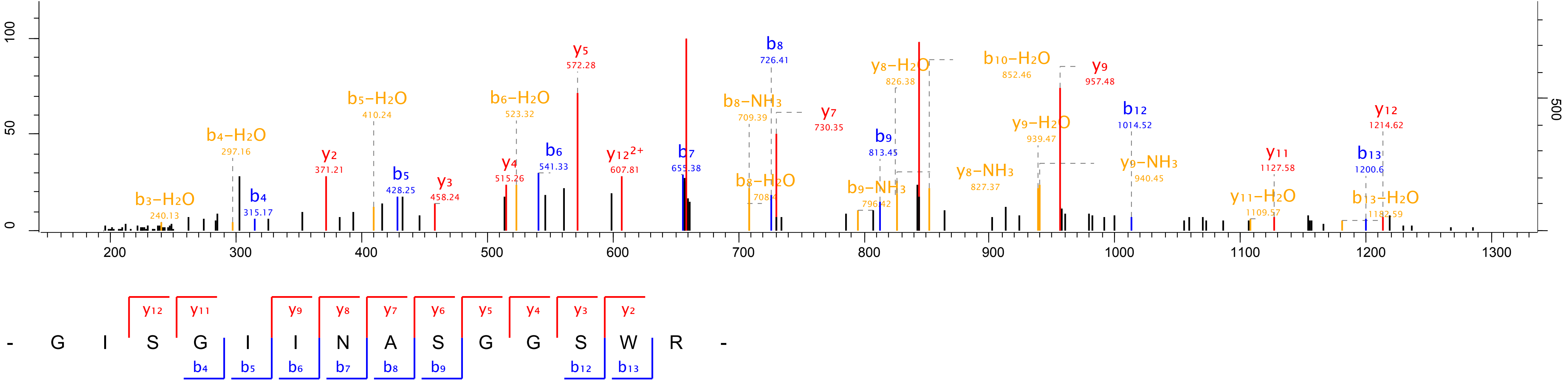

Raw file	Scan	Method	Score	m/z	Gene names
HBT_20130916_BV2_IL102_03	1018	ITMS; CID	70.68	498.88	Snrnp35;1700128F08Rik

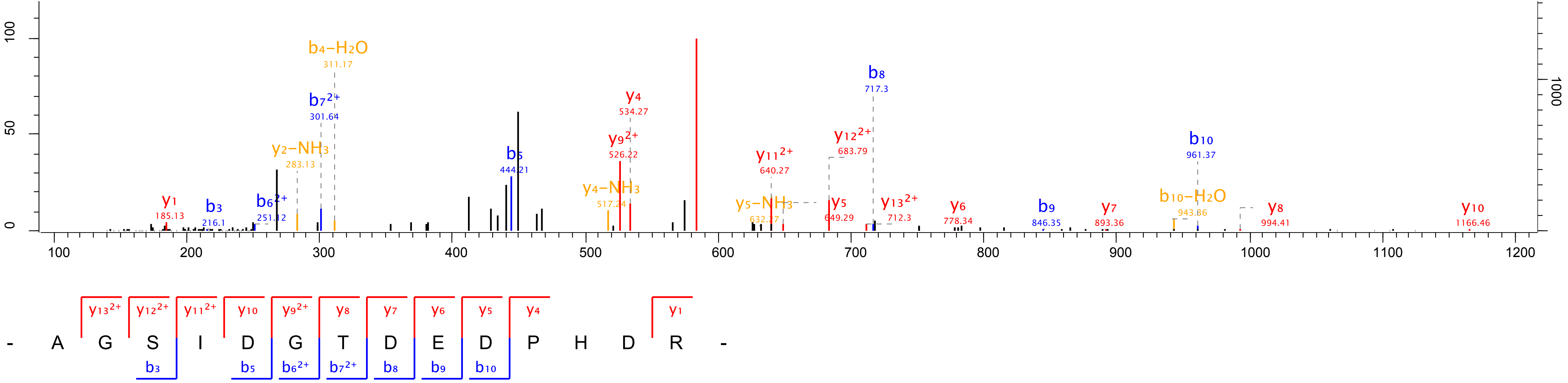

Raw file	Scan	Method	Score	m/z	Gene names
HBT_20130916_BV2_IL102_02	25746	ITMS; CID	102.52	593.84	Zmym1

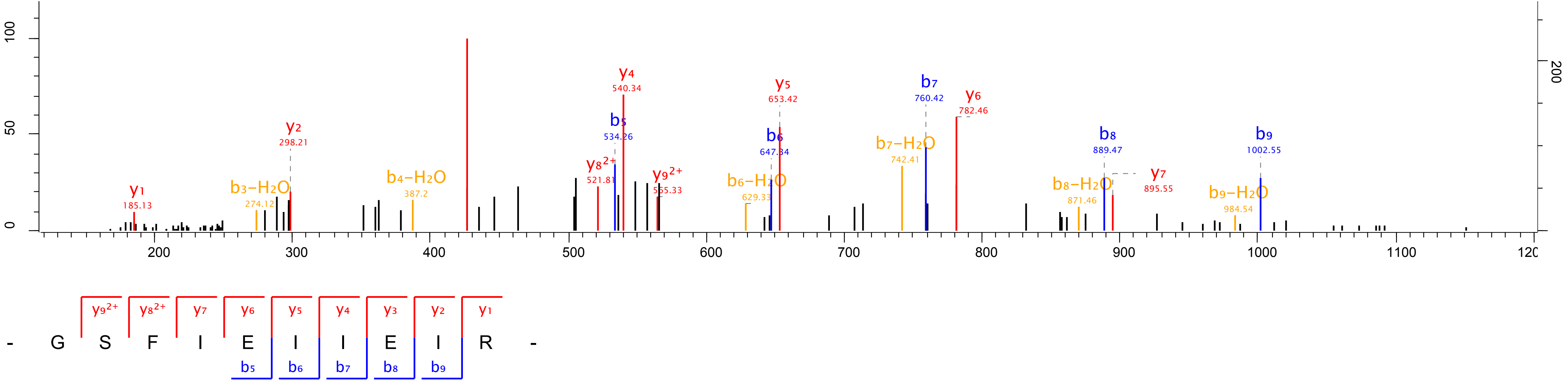

Raw file	Scan	Method	Score	m/z	Gene names
HBT_20130916_BV2_IL102_02	23298	ITMS; CID	150.11	847.45	Vprbp

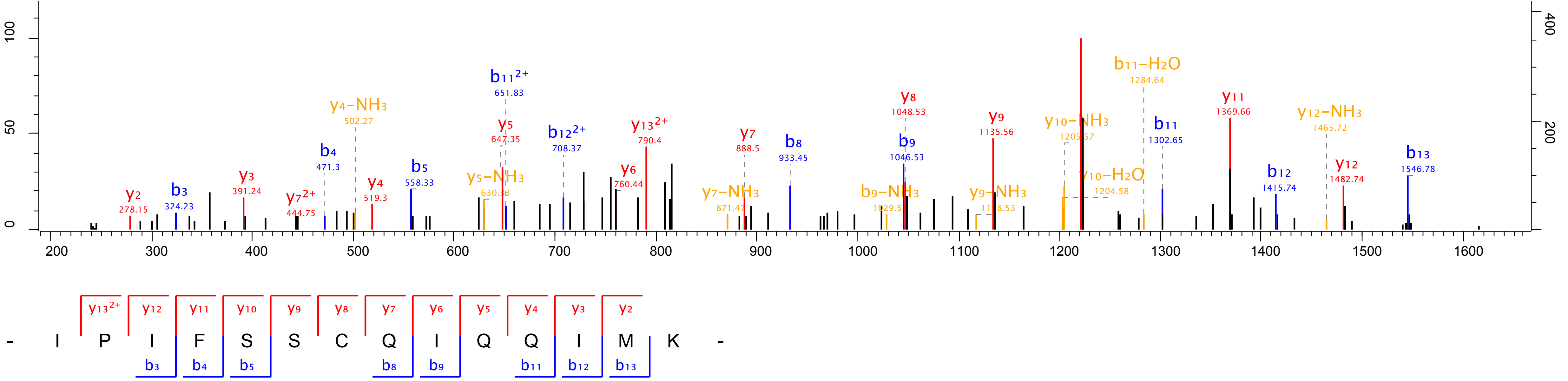

Raw file	Scan	Method	Score	m/z	Gene names
HBT_20130916_BV2_IL102_02	22814	ITMS; CID	98.37	731.39	Tnip1

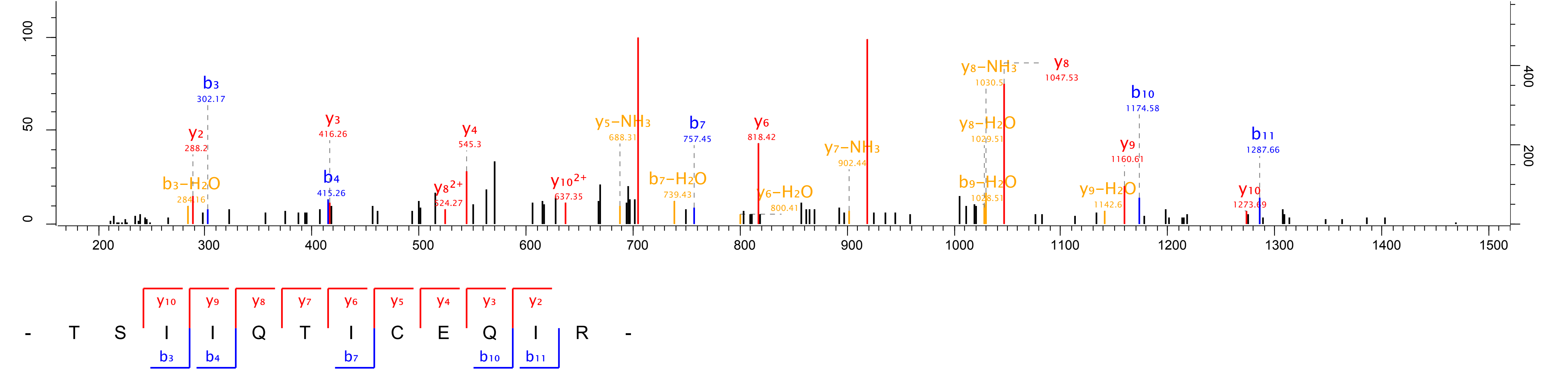

Raw file	Scan	Method	Score	m/z	Gene names
HBT_20130916_BV2_IL102_02	22770	ITMS; CID	134.3	656.39	Exoc6

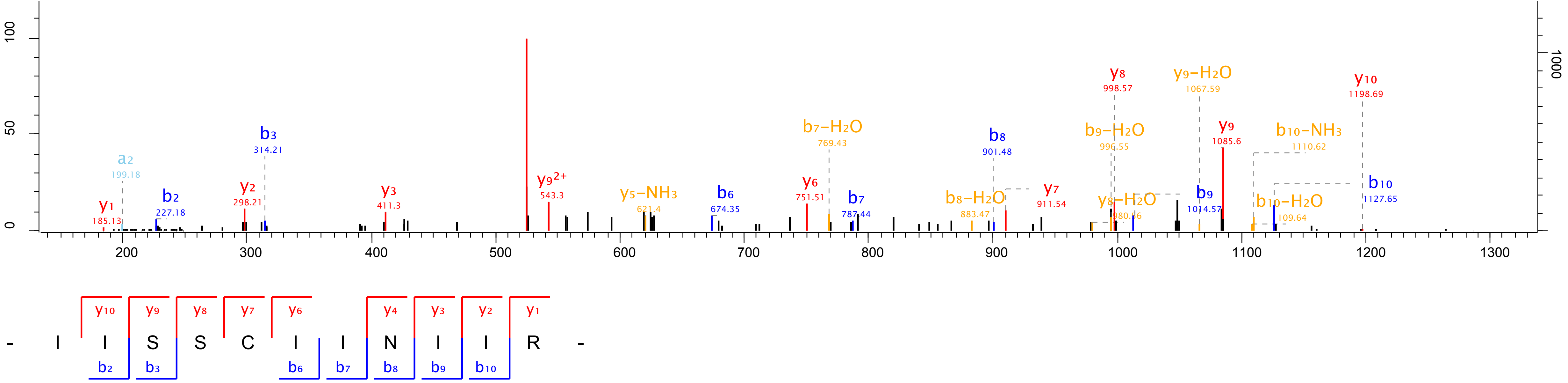

Raw file	Scan	Method	Score	m/z	Gene names
HBT_20130916_BV2_IL102_02	19882	ITMS; CID	191.33	714.9	Ostc

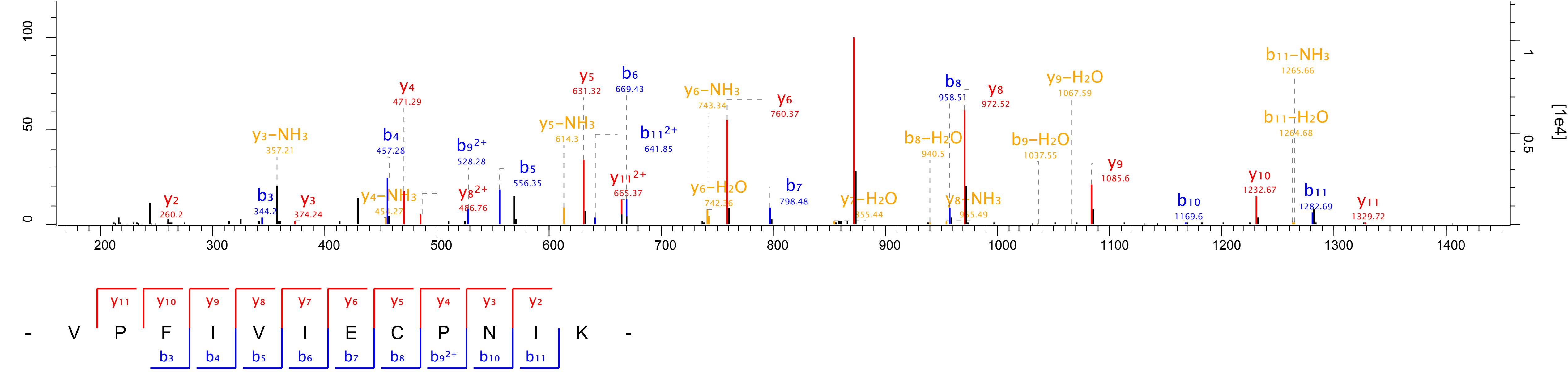

Raw file	Scan	Method	Score	m/z	Gene names
HBT_20130916_BV2_IL102_02	18803	ITMS; CID	91.2	651.39	Tmem186

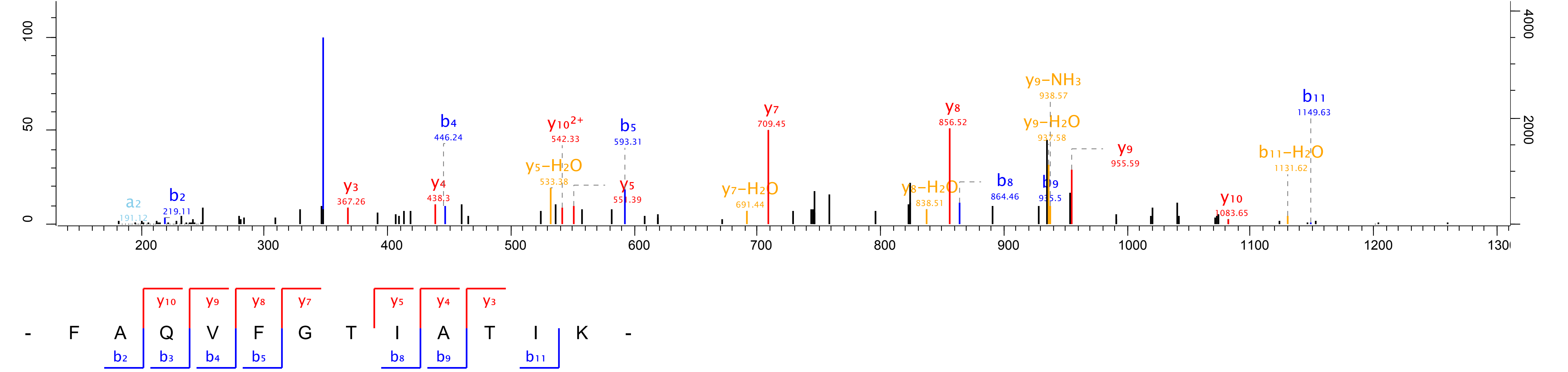

Raw file	Scan	Method	Score	m/z	Gene names
HBT_20130916_BV2_IL102_02	18466	ITMS; CID	98.41	616.86	Grpel2

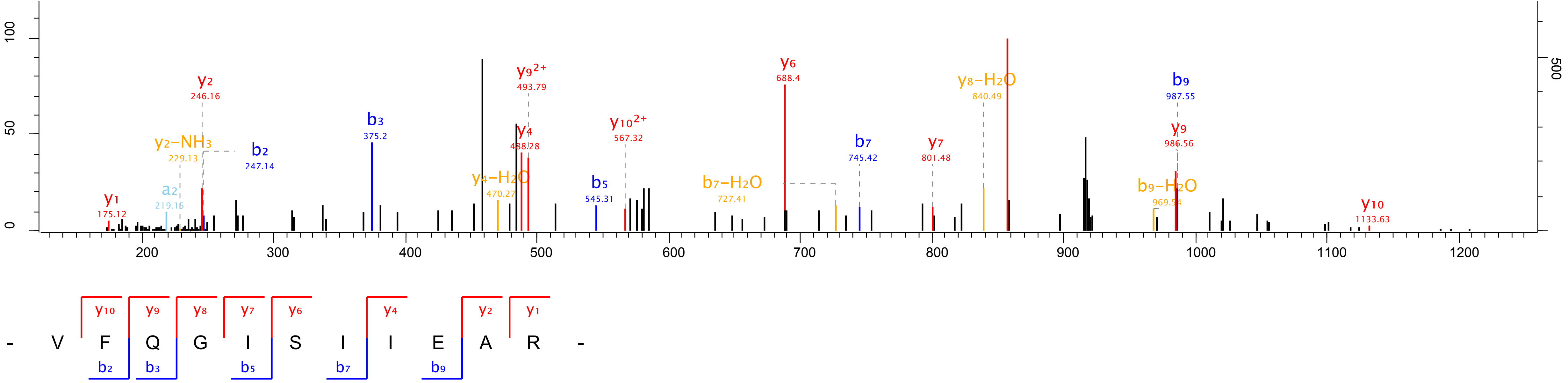

Raw file	Scan	Method	Score	m/z	Gene names
HBT_20130916_BV2_IL102_02	11274	ITMS; CID	121.09	515.79	Klhl40

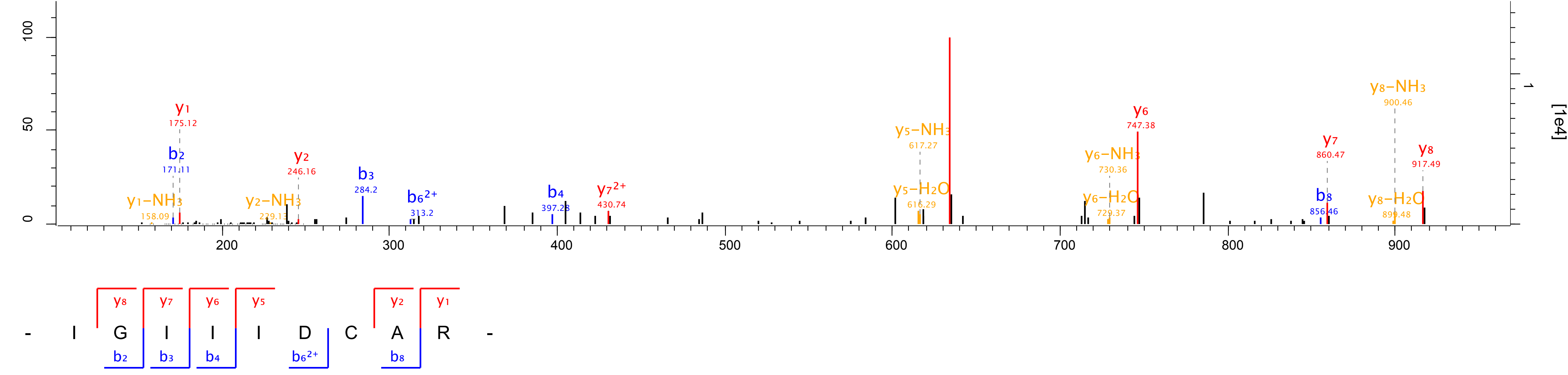

Raw file	Scan	Method	Score	m/z	Gene names
HBT_20130916_BV2_IL102_02	1068	ITMS; CID	123.67	671.28	Fam76b

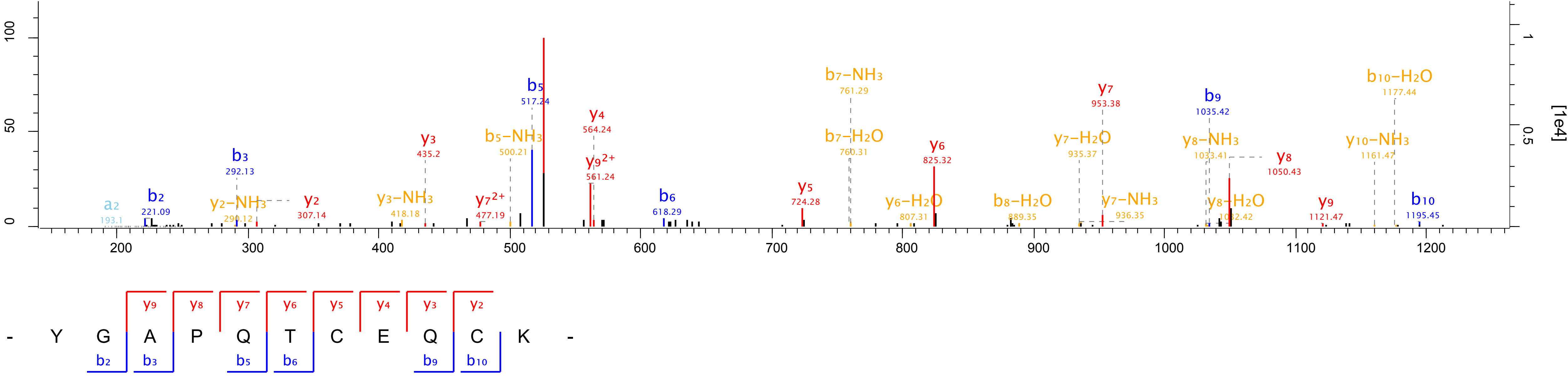

Raw file	Scan	Method	Score	m/z	Gene names
HBT_20130916_BV2_IL102_01	32583	ITMS; CID	114.55	1158.18	Dpp9

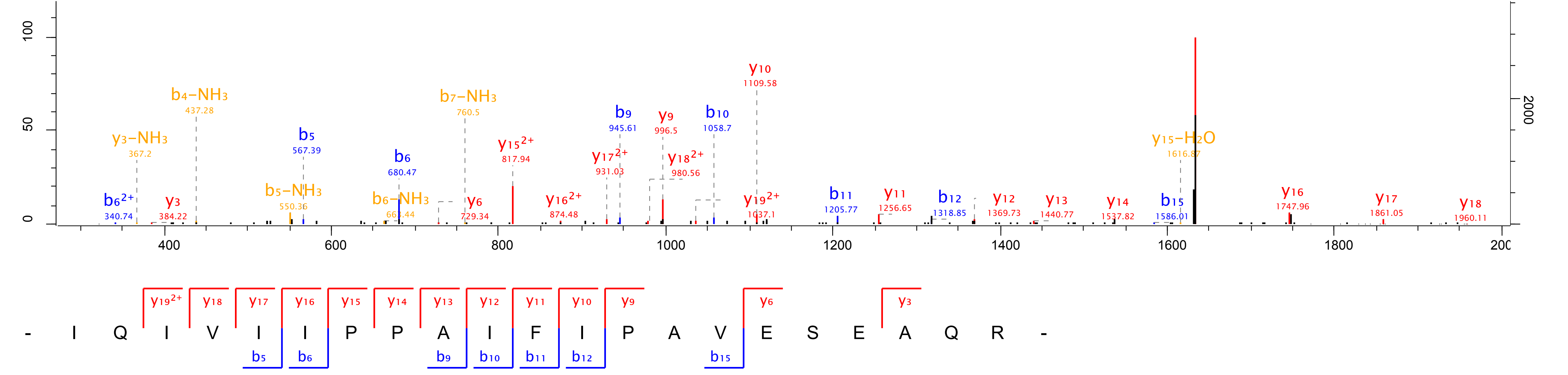

Raw file	Scan	Method	Score	m/z	Gene names
HBT_20130916_BV2_IL102_01	29718	ITMS; CID	170.16	991.55	Pigu

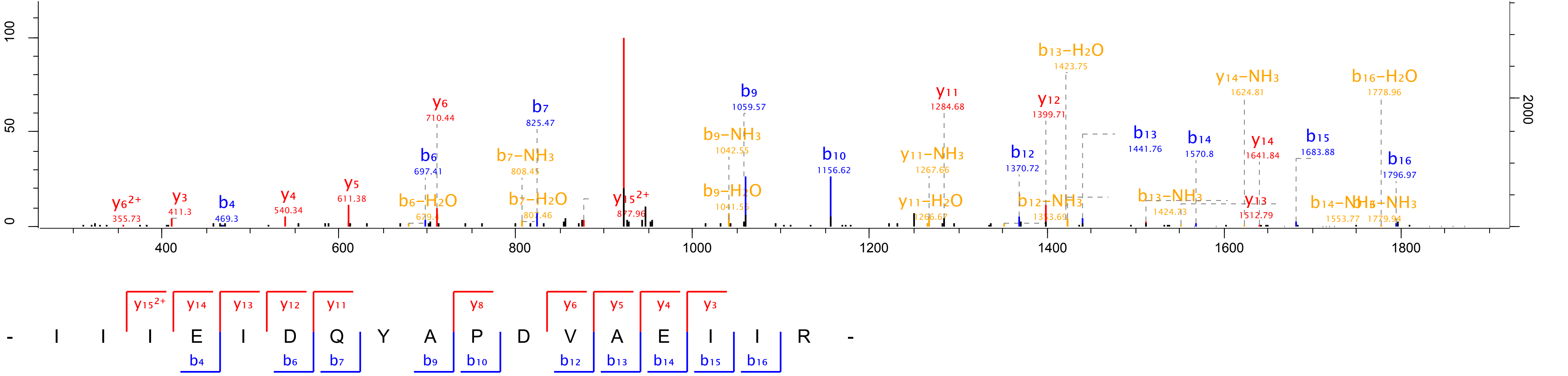

Raw file	Scan	Method	Score	m/z
HBT_20130916_BV2_IL102_01	14472	ITMS; CID	90.79	739.42

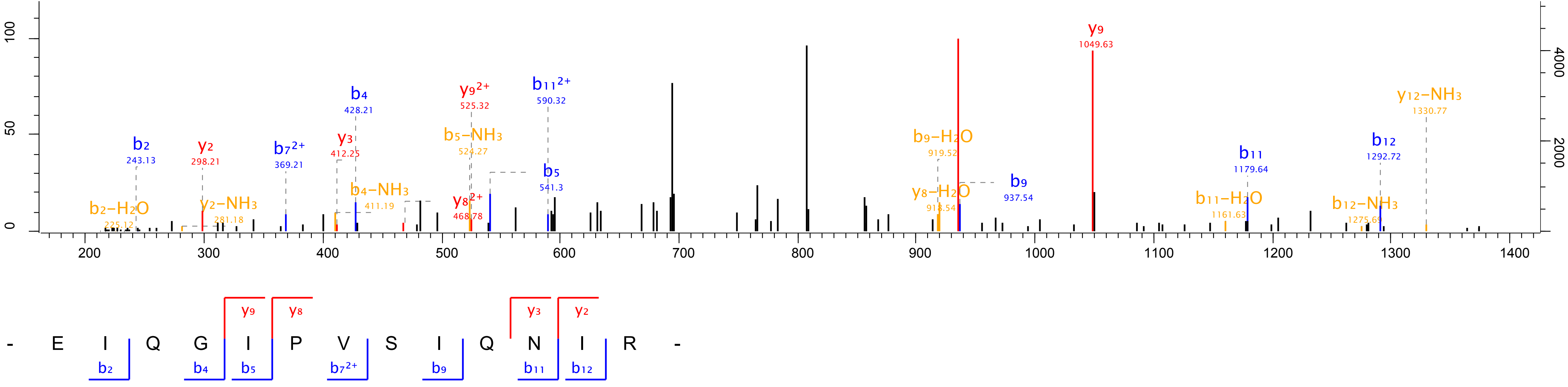

Raw file	Scan	Method	Score	m/z	Gene names
HBT_20130916_BV2_IL101_06	19280	ITMS; CID	133.15	707.43	Ccdc109b

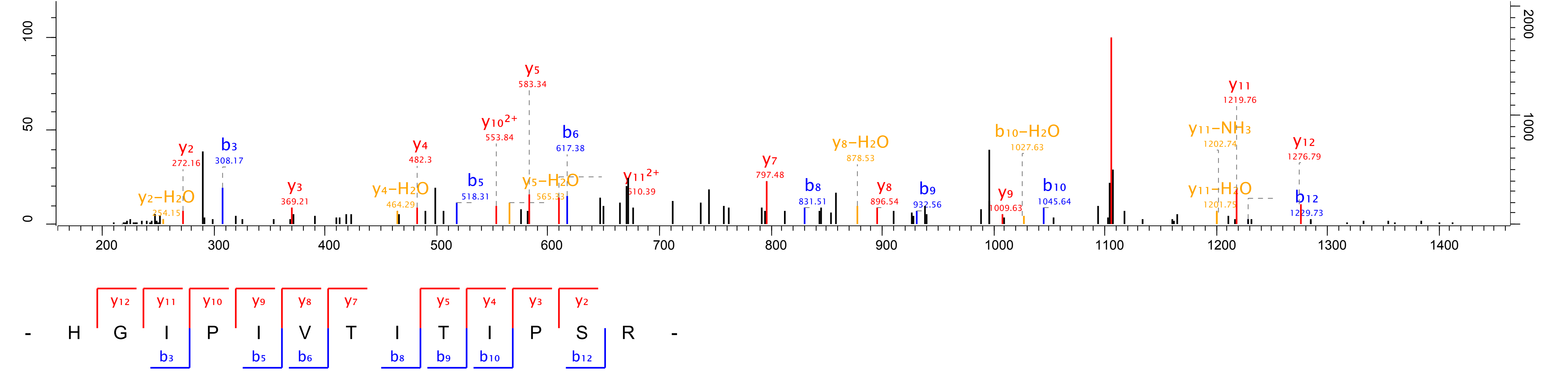

Raw file	Scan	Method	Score	m/z	Gene names
HBT_20130916_BV2_IL101_06	13746	ITMS; CID	91.96	643.38	Mrpl48

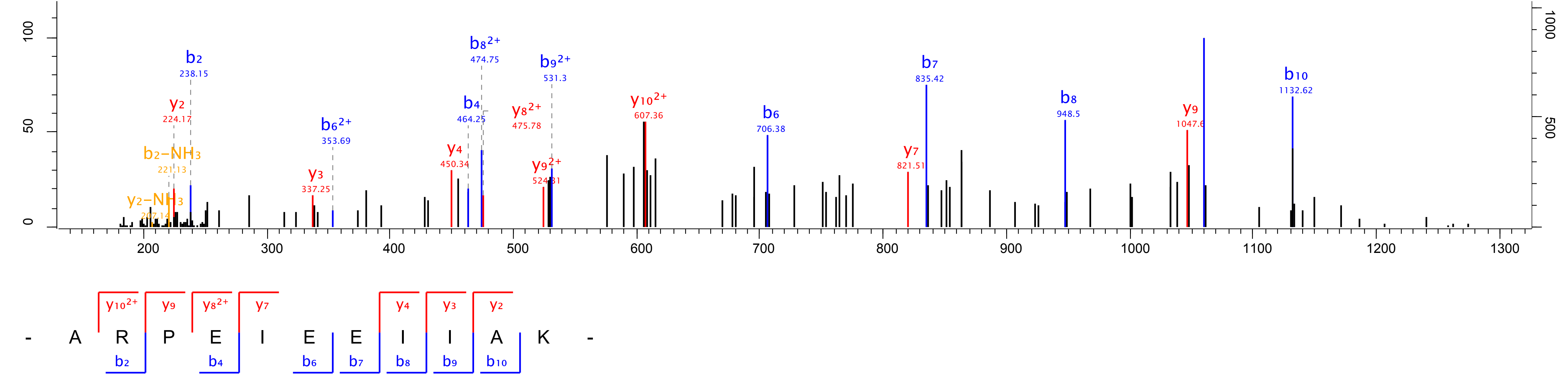

Raw file	Scan	Method	Score	m/z	Gene names
HBT_20130916_BV2_IL101_05	9531	ITMS; CID	135.99	634.37	Mrps6

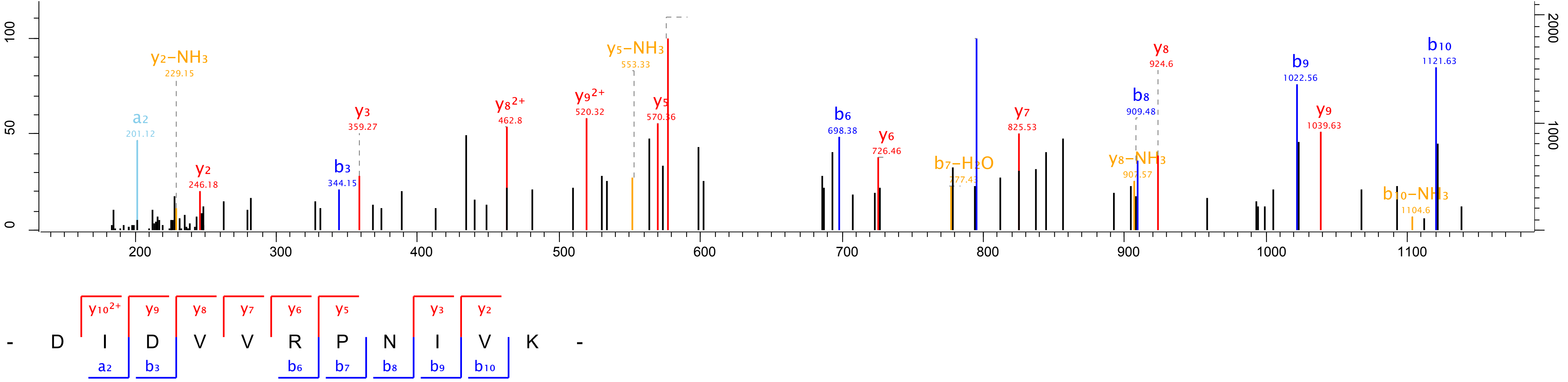

Raw file	Scan	Method	Score	m/z	Gene names
HBT_20130916_BV2_IL101_05	22657	ITMS; CID	87.83	524.32	Ndufaf4

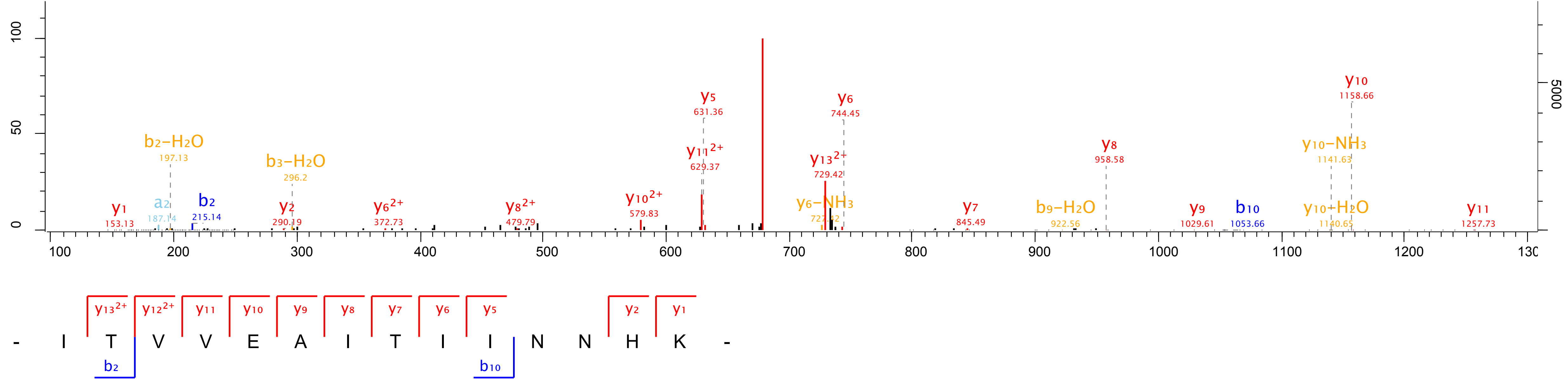

Raw file	Scan	Method	Score	m/z	Gene names
HBT_20130916_BV2_IL101_05	16664	ITMS; CID	96.76	501.62	Nadk2

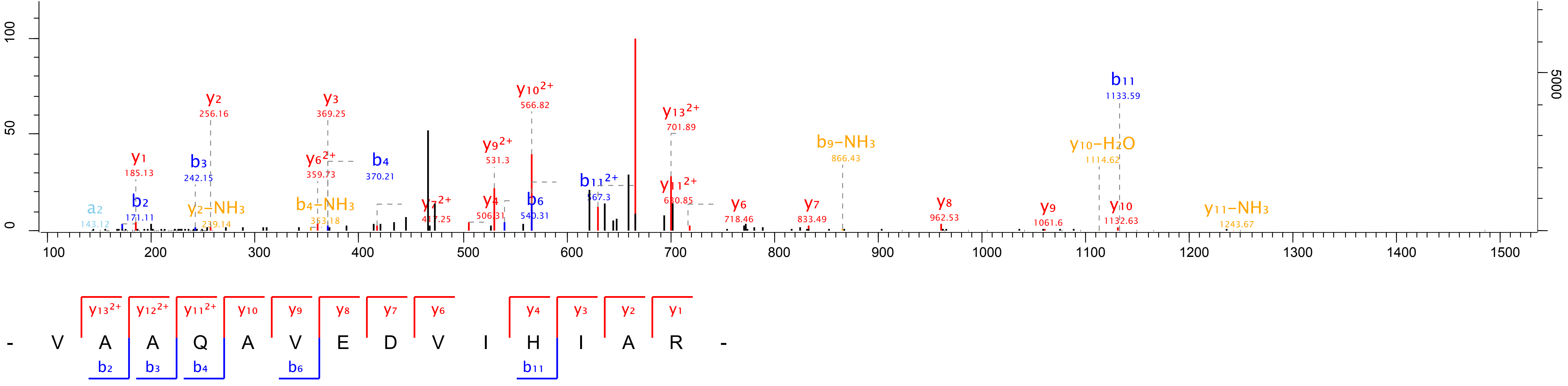

Raw file	Scan	Method	Score	m/z	Gene names
HBT_20130916_BV2_IL101_05	14810	ITMS; CID	94.77	473.61	Mrpl33

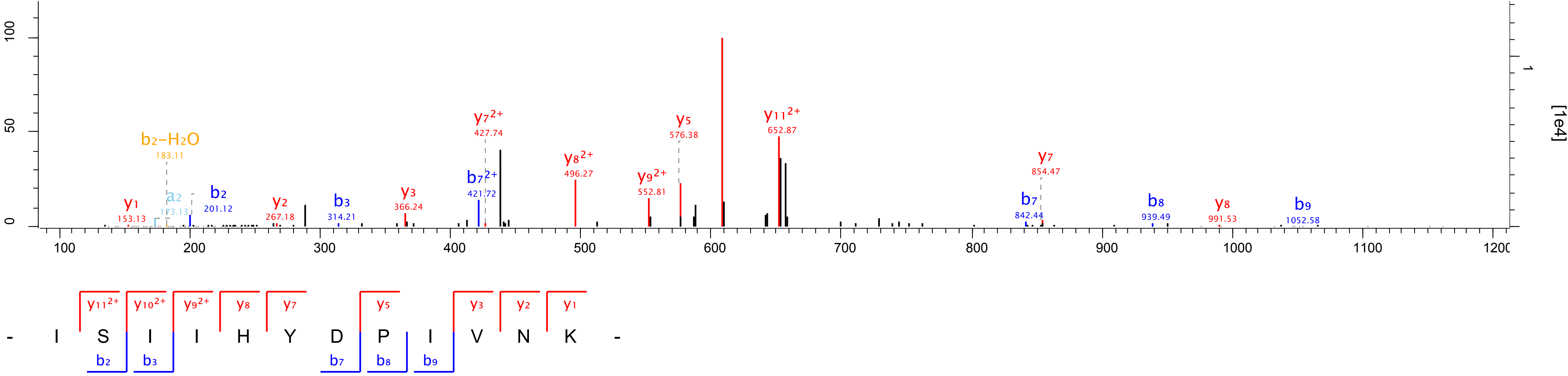

Raw file	Scan	Method	Score	m/z	Gene names
HBT_20130916_BV2_IL101_04	30058	ITMS; CID	91.81	708.38	Pdcd7

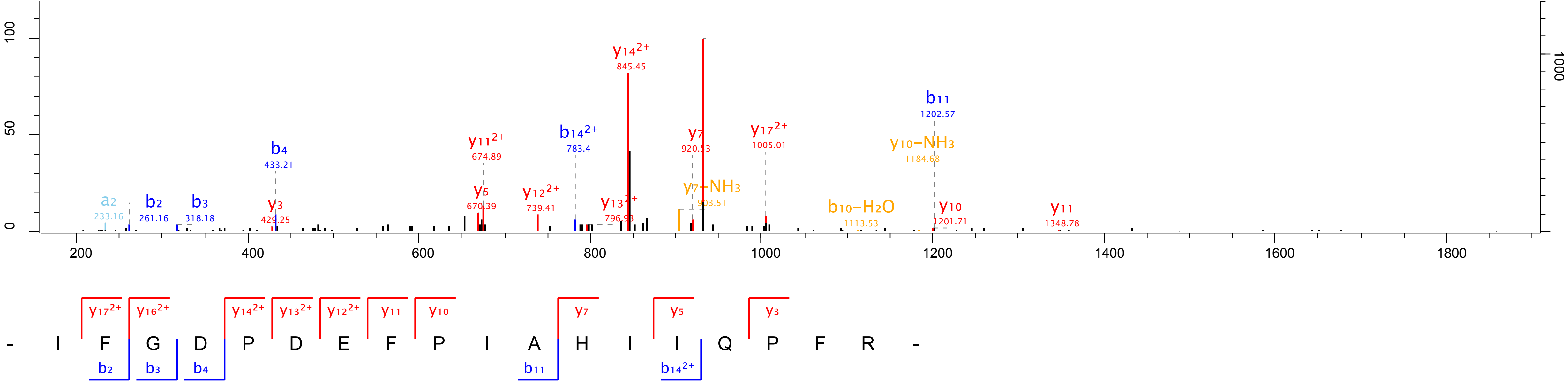

Raw file	Scan	Method	Score	m/z	Gene names
HBT_20130916_BV2_IL101_04	29635	ITMS; CID	84.75	669.01	Nfia;Nfix

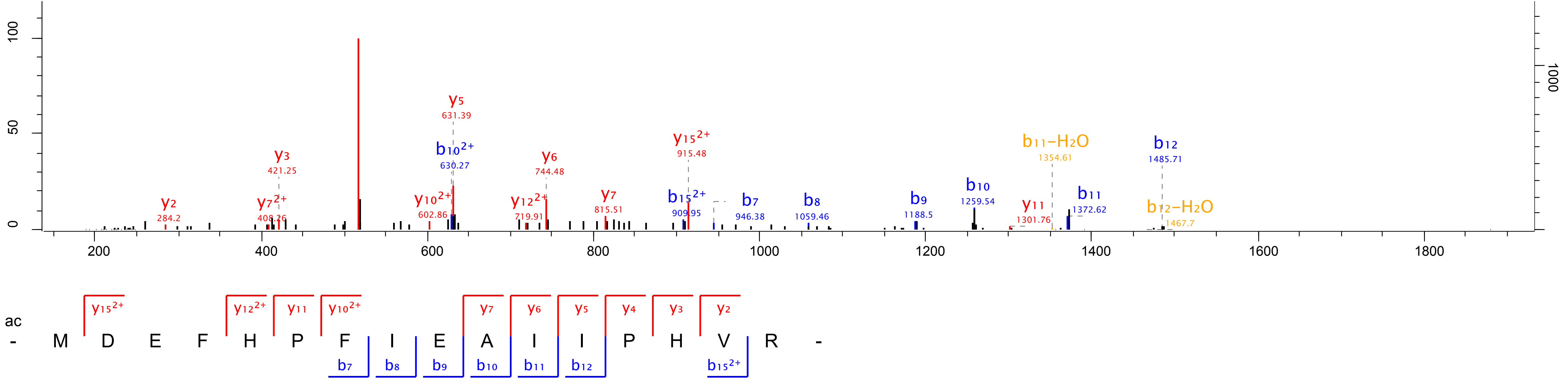

Raw file	Scan	Method	Score	m/z	Gene names
HBT_20130916_BV2_IL101_04	23939	ITMS; CID	156.72	697.38	Mcee

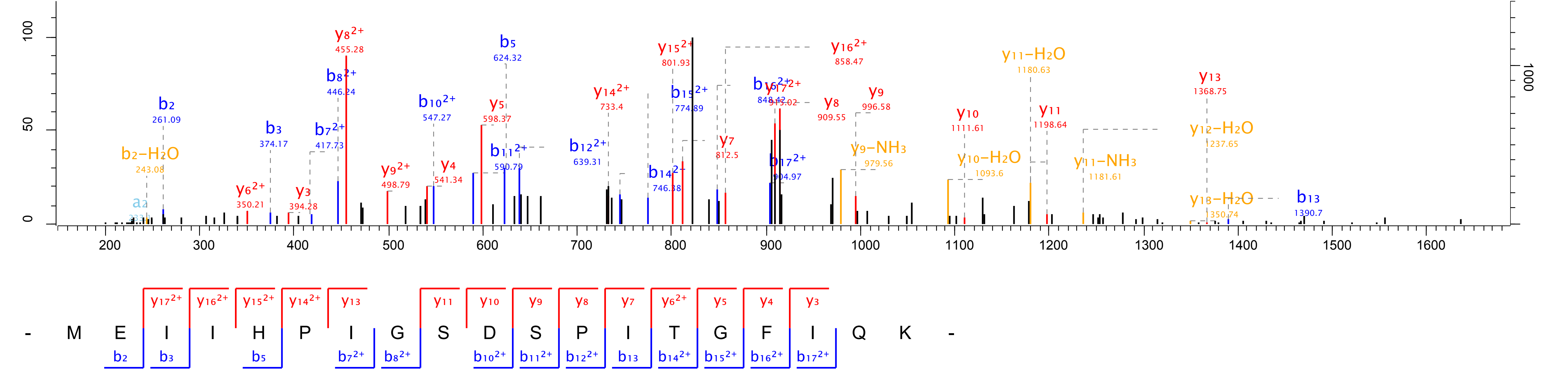

Raw file	Scan	Method	Score	m/z	Gene names
HBT_20130916_BV2_IL101_04	21170	ITMS; CID	166.3	1131.55	Cnih4

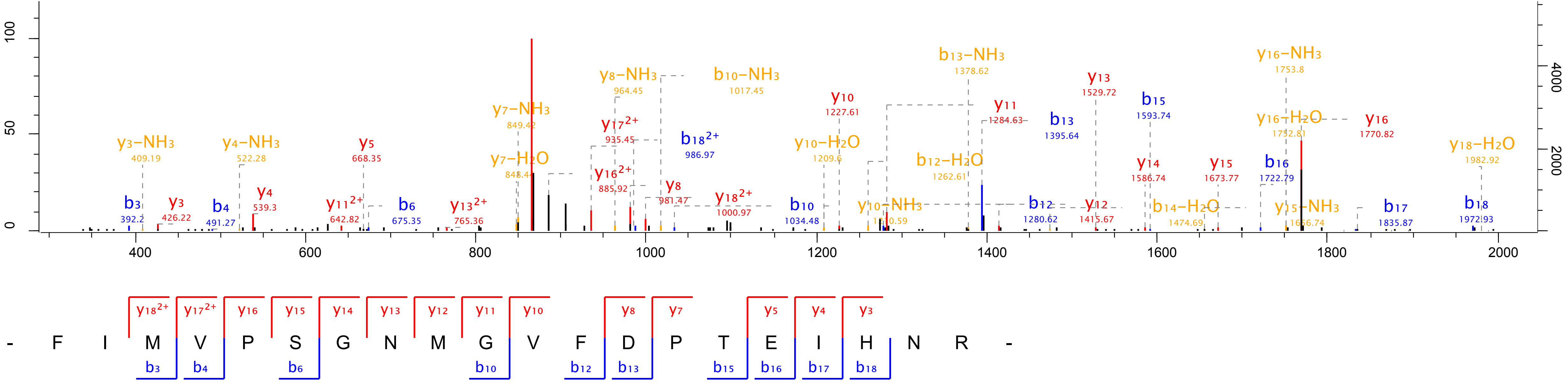

Raw file	Scan	Method	Score	m/z	Gene names
HBT_20130916_BV2_IL101_04	20827	ITMS; CID	62.78	952.16	Slc38a9

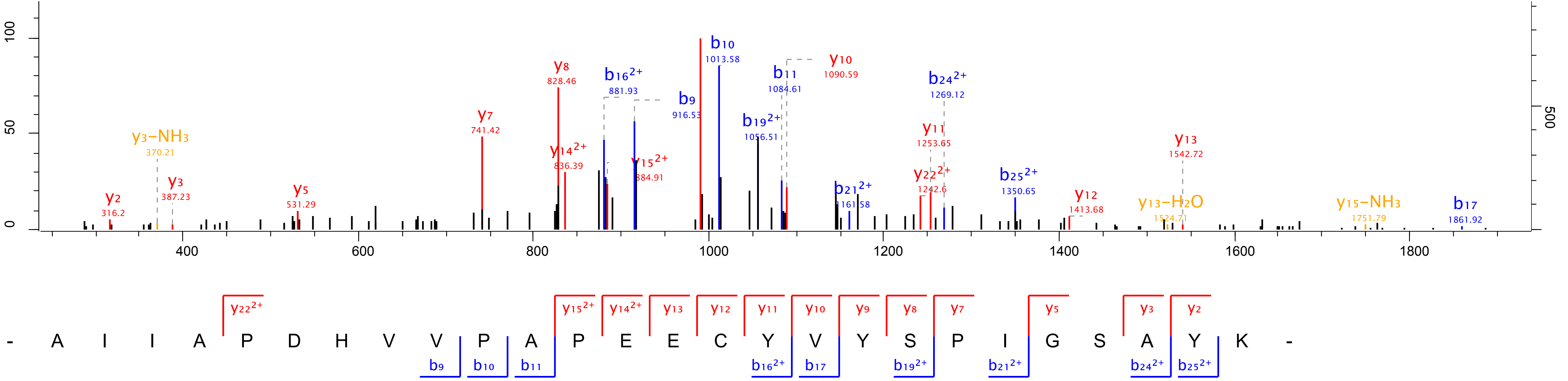

Raw file	Scan	Method	Score	m/z	Gene names
HBT_20130916_BV2_IL101_04	20791	ITMS; CID	118.08	876.46	Tmem256

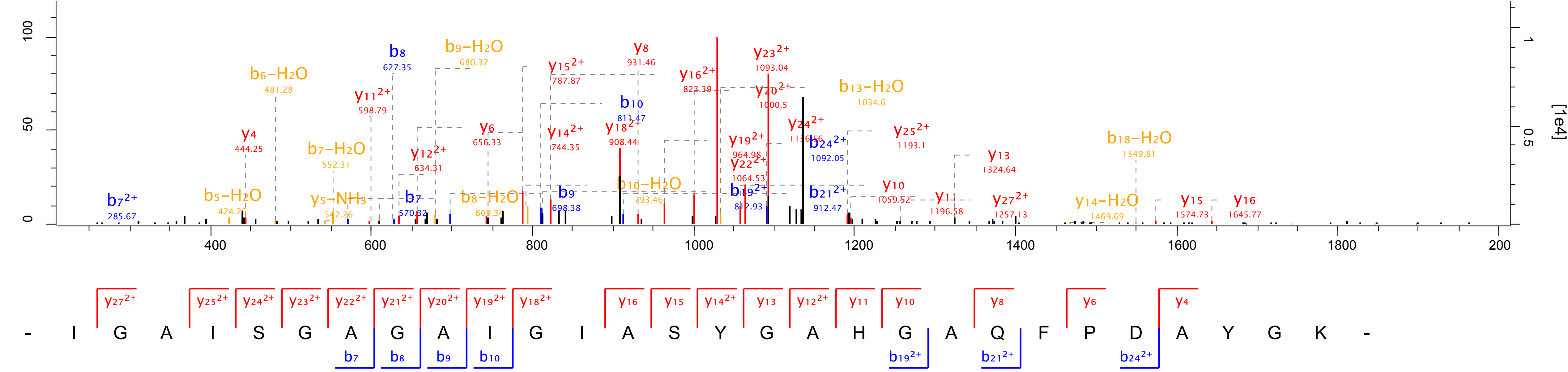

Raw file	Scan	Method	Score	m/z	Gene names
HBT_20130916_BV2_IL101_04	20324	ITMS; CID	102.52	537.94	Runx3

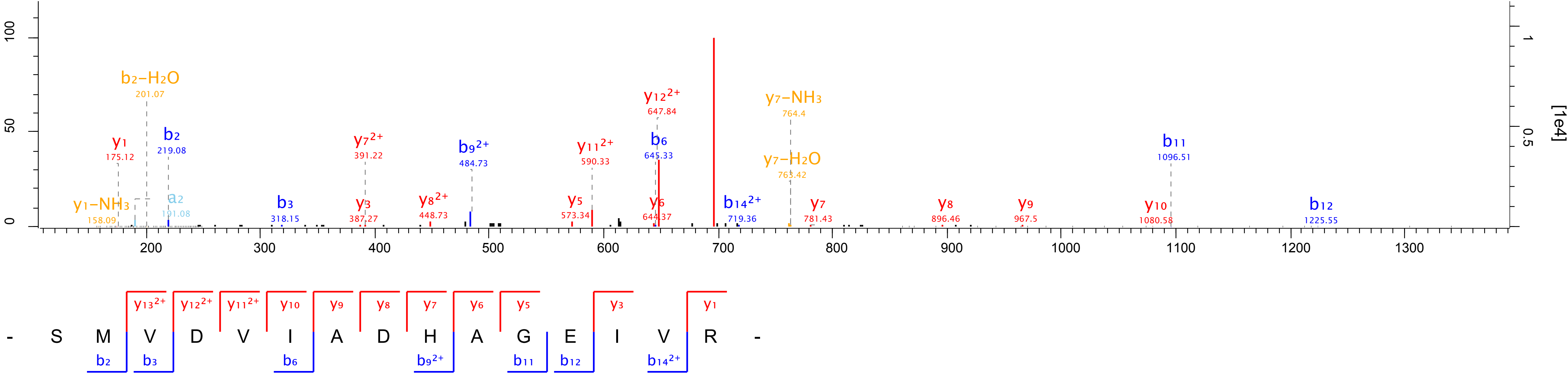

Raw file	Scan	Method	Score	m/z	Gene names
HBT_20130916_BV2_IL101_03	7354	ITMS; CID	134.57	618.83	Ttc4

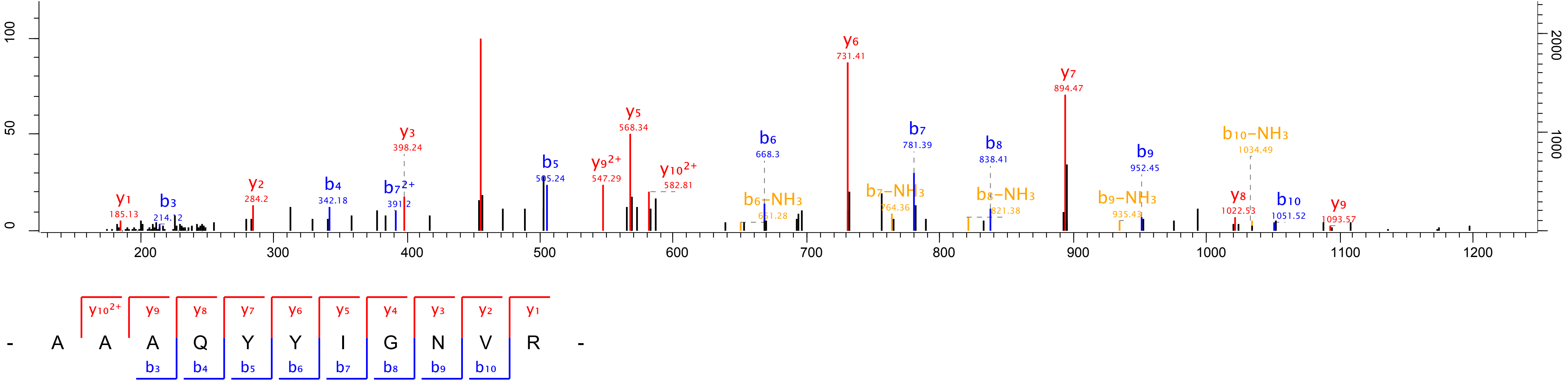

Raw file	Scan	Method	Score	m/z	Gene names
HBT_20130916_BV2_IL101_03	28446	ITMS; CID	64.07	916.49	Fbxl6

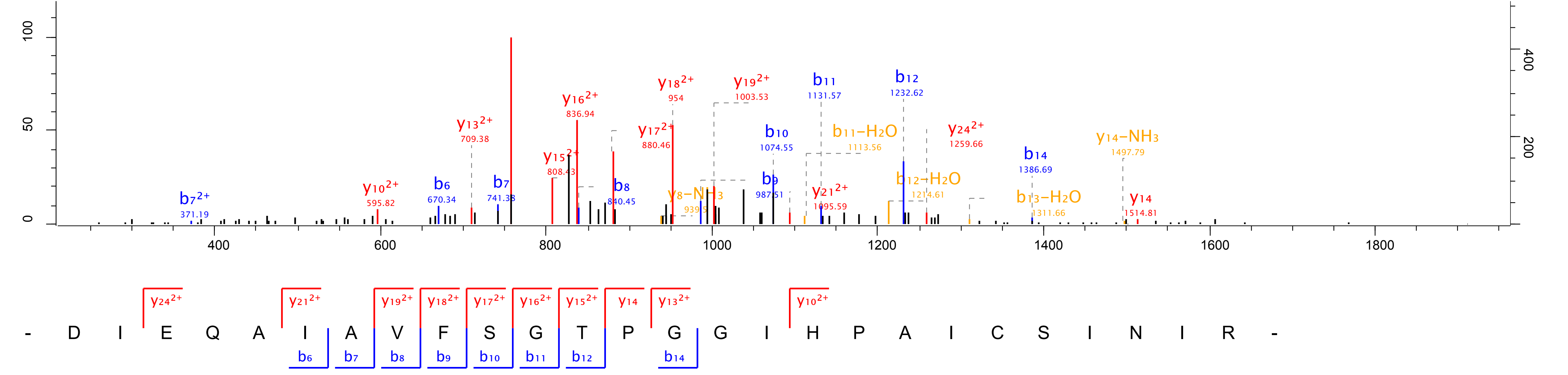

Raw file	Scan	Method	Score	m/z	Gene names
HBT_20130916_BV2_IL101_03	25448	ITMS; CID	132.32	632.86	Slc25a40

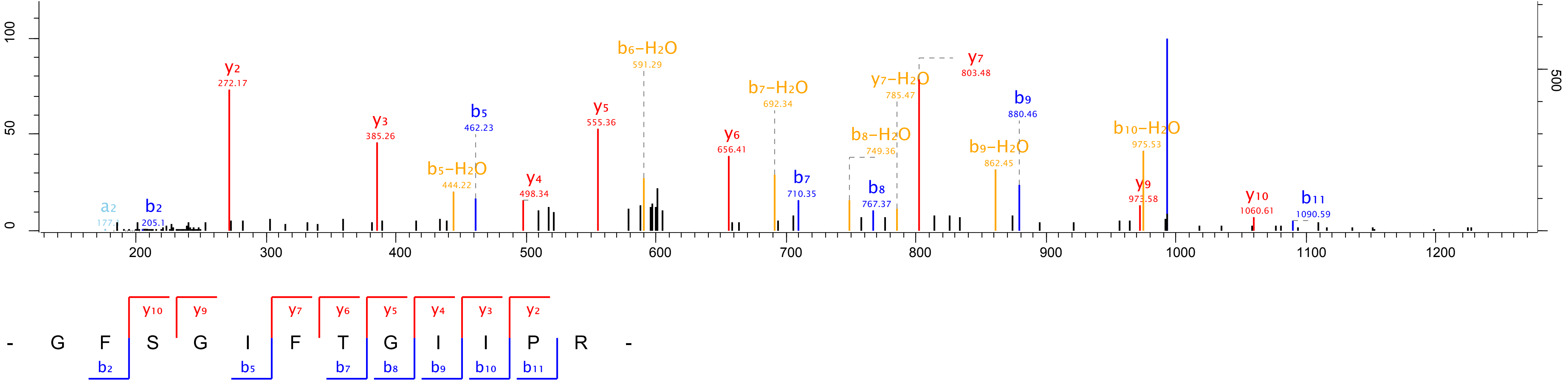

Raw file	Scan	Method	Score	m/z	Gene names
HBT_20130916_BV2_IL101_03	23845	ITMS; CID	122.96	617.36	Nsmce4a

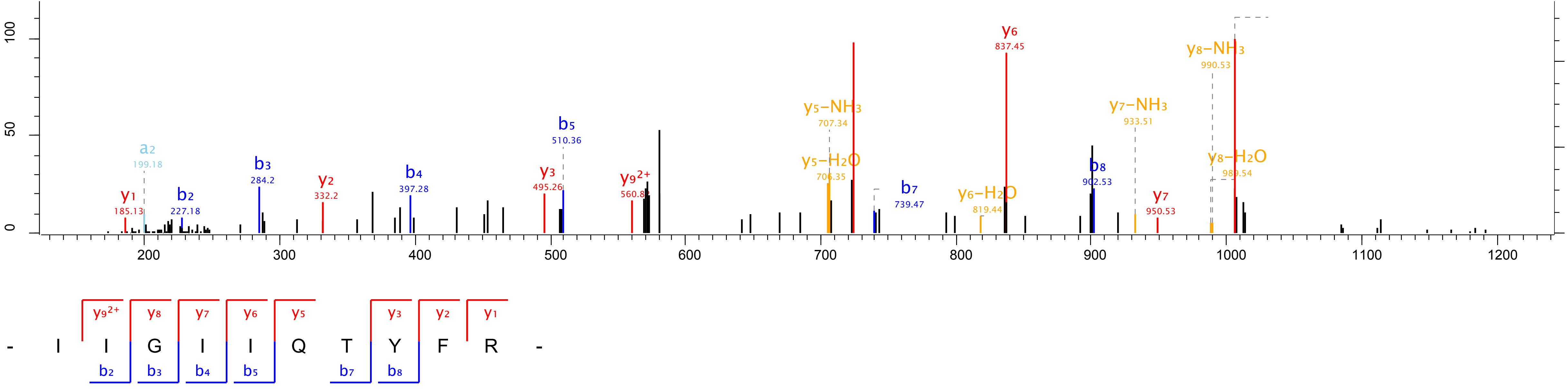

Raw file	Scan	Method	Score	m/z	Gene names
HBT_20130916_BV2_IL101_03	22406	ITMS; CID	81.02	736.39	Tlx3

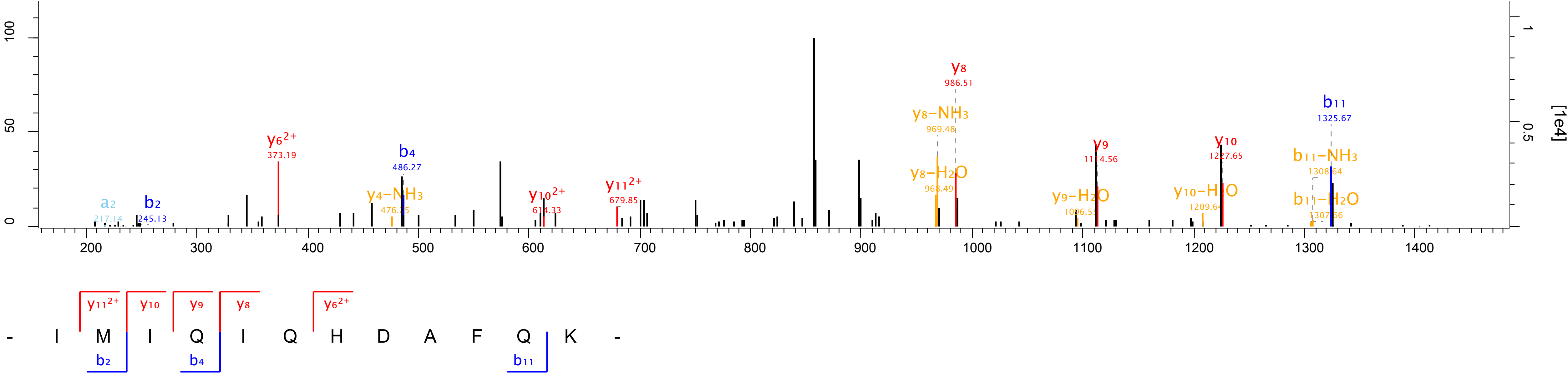

Raw file	Scan	Method	Score	m/z	Gene names
HBT_20130916_BV2_IL101_03	22276	ITMS; CID	102.69	1043.54	Kdelc2

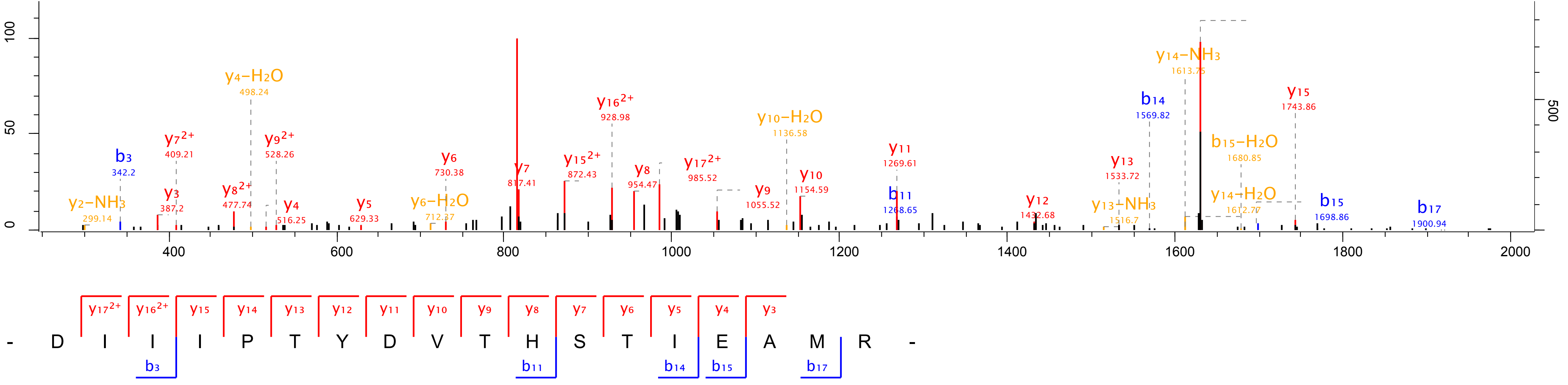

Raw file	Scan	Method	Score	m/z	Gene names
HBT_20130916_BV2_IL101_03	21935	ITMS; CID	166.11	680.85	Zfp800;Znf800

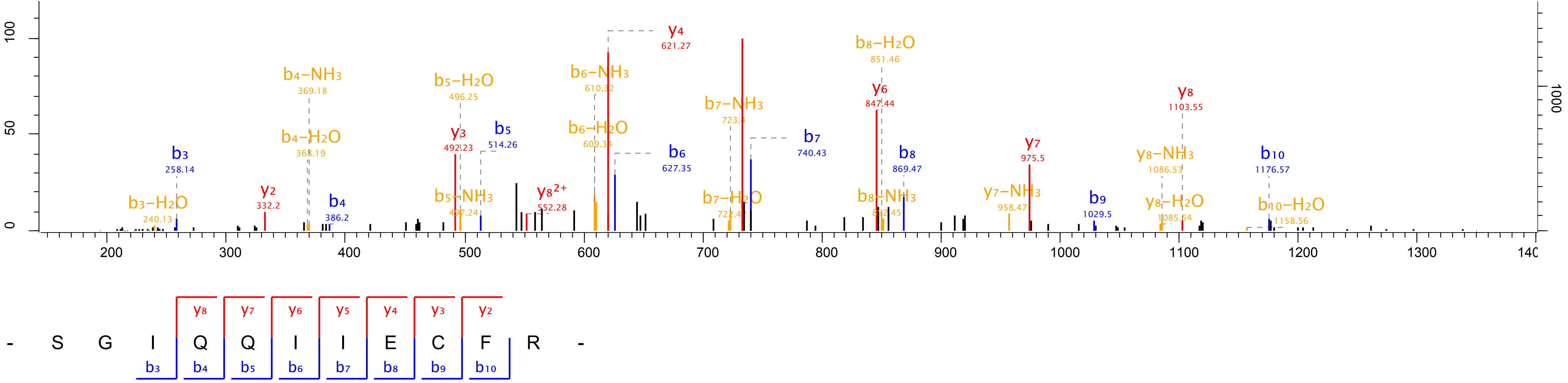

Raw file	Scan	Method	Score	m/z	Gene names
HBT_20130916_BV2_IL101_03	20625	ITMS; CID	237.05	772.91	Aagab

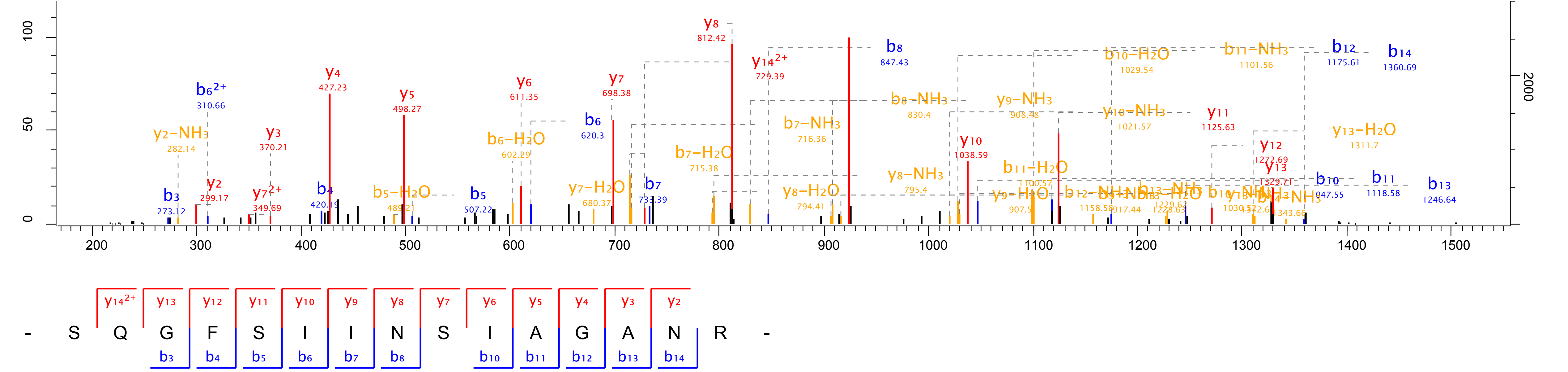

Raw file	Scan	Method	Score	m/z	Gene names
HBT_20130916_BV2_IL101_03	16510	ITMS; CID	91.12	648.85	Phka2

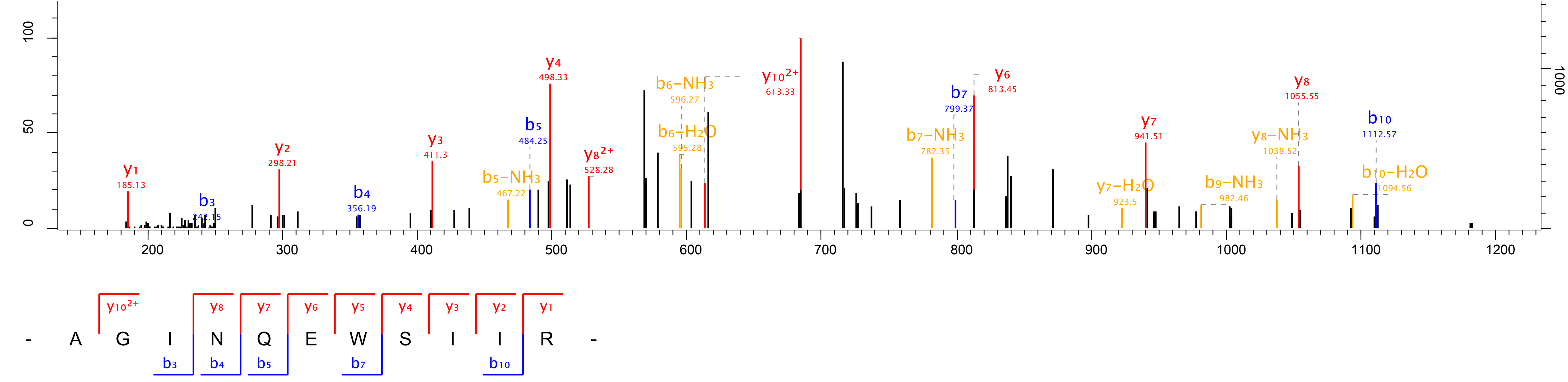

Raw file	Scan	Method	Score	m/z	Gene names
HBT_20130916_BV2_IL101_02	8141	ITMS; CID	133.88	652.82	Igkv1-115

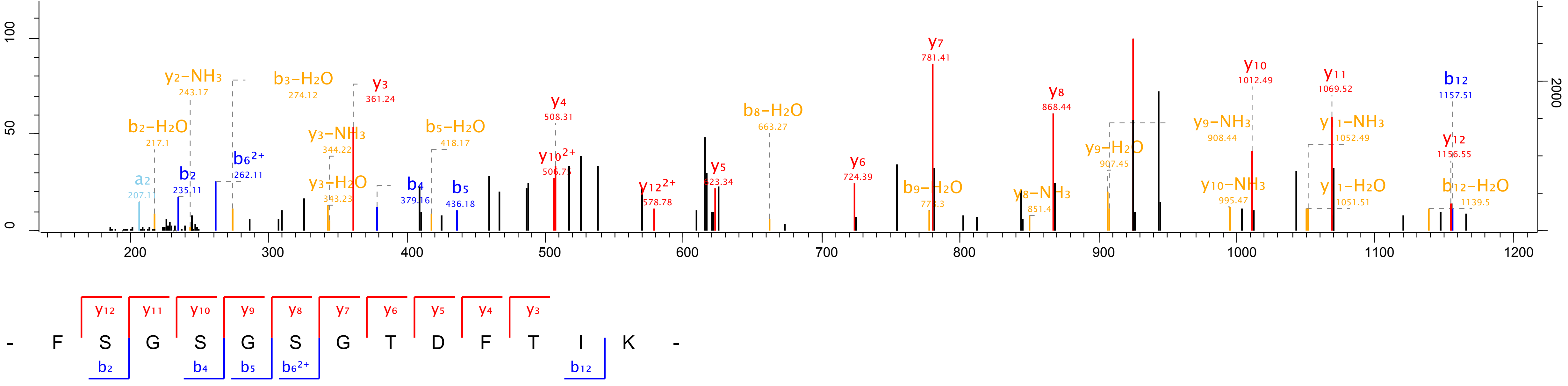

Raw file	Scan	Method	Score	m/z	Gene names
HBT_20130916_BV2_IL101_02	31581	ITMS; CID	105.65	782.44	Dolpp1

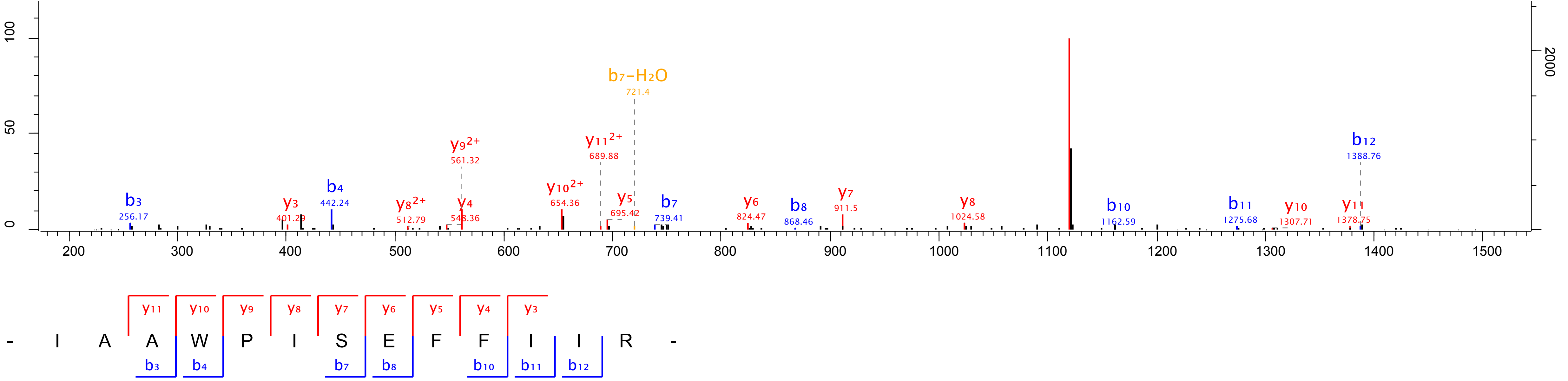

Raw file	Scan	Method	Score	m/z	Gene names
HBT_20130916_BV2_IL101_02	31243	ITMS; CID	86.11	765.95	Itpr1

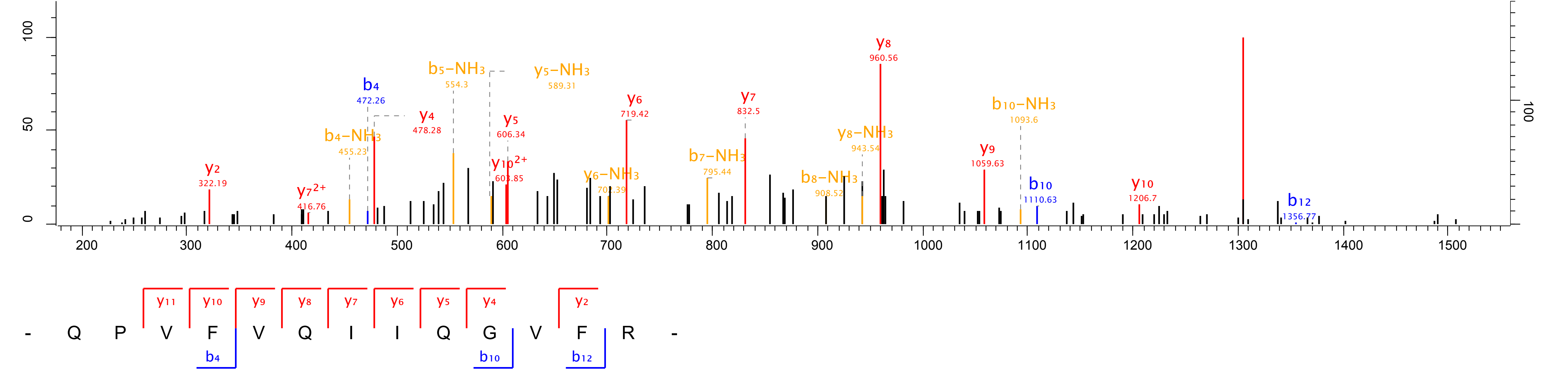

Raw file	Scan	Method	Score	m/z	Gene names
HBT_20130916_BV2_IL101_02	31032	ITMS; CID	86.5	816.01	Armc5

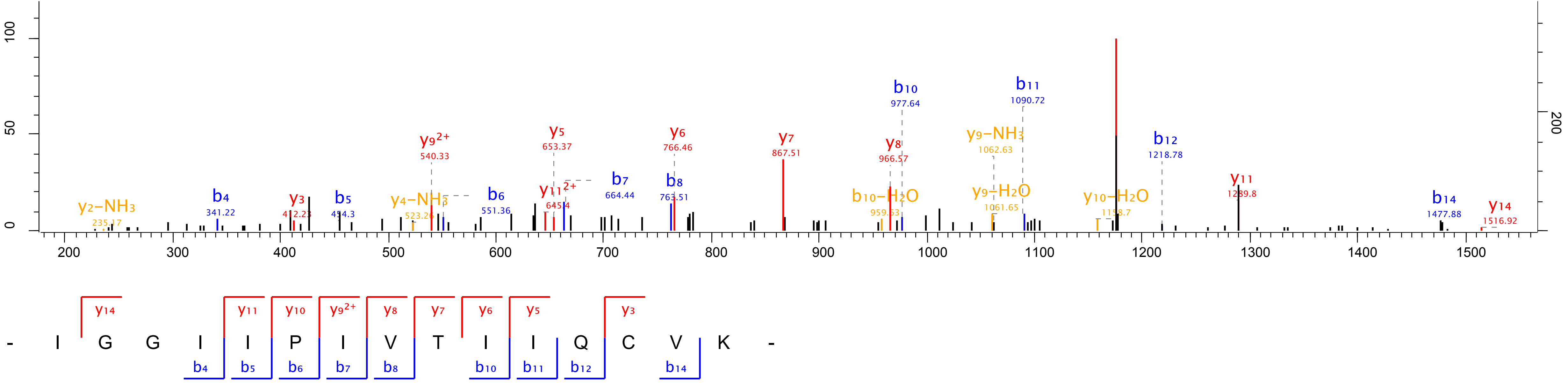

Raw file	Scan	Method	Score	m/z	Gene names
HBT_20130916_BV2_IL101_02	30897	ITMS; CID	95.2	787.95	Cdk5rap3

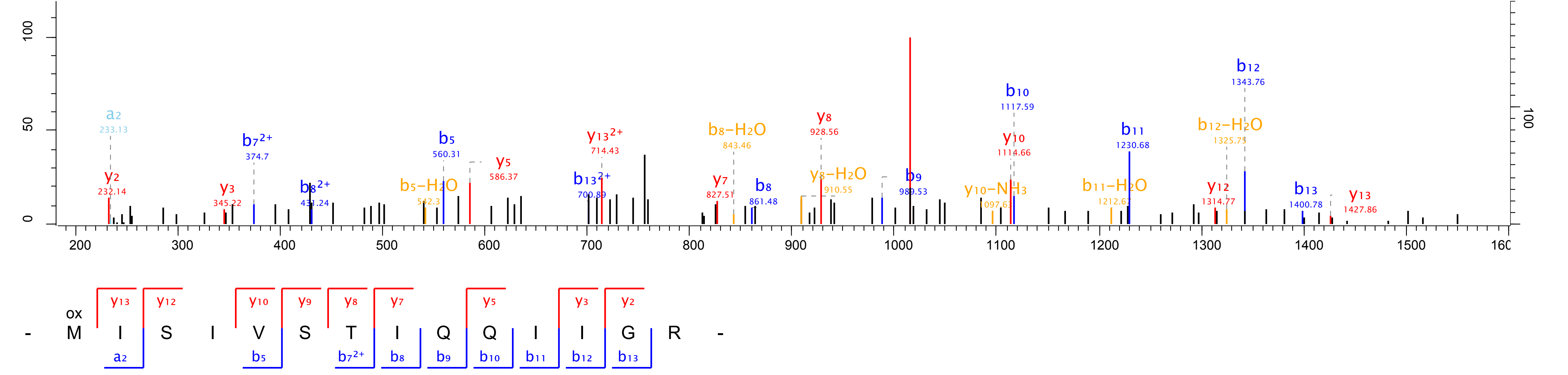

Raw file	Scan	Method	Score	m/z	Gene names
HBT_20130916_BV2_IL101_02	27800	ITMS; CID	81.99	894	Lipt1

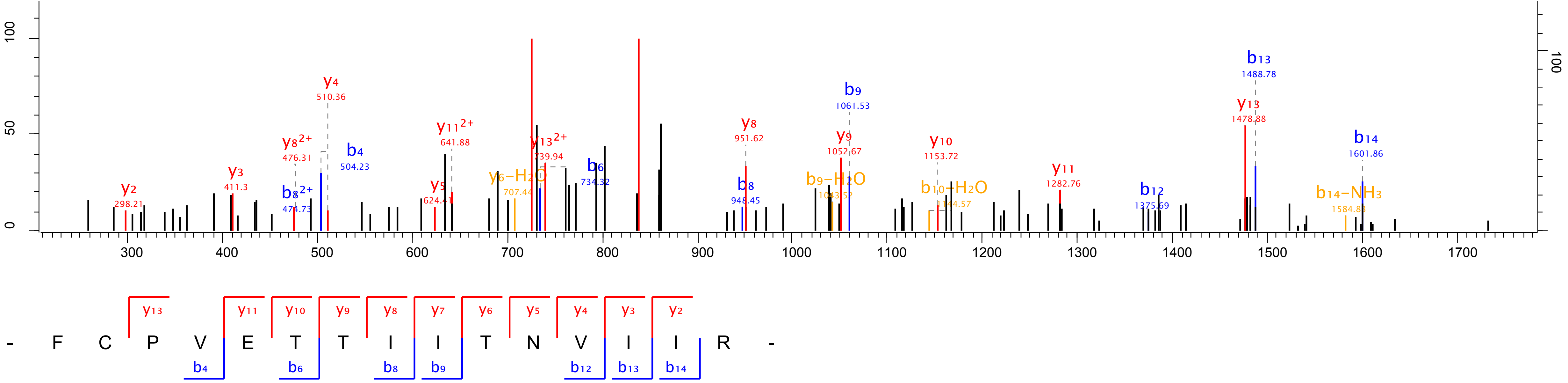

Raw file	Scan	Method	Score	m/z
HBT_20130916_BV2_IL101_02	27785	ITMS; CID	93.35	856.47

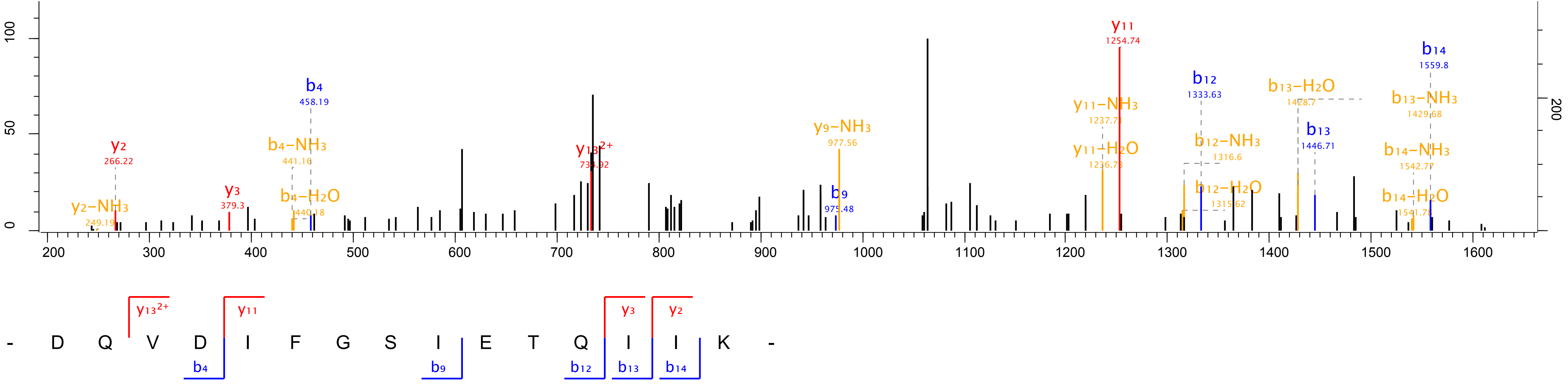

Raw file	Scan	Method	Score	m/z	Gene names
HBT_20130916_BV2_IL101_02	21325	ITMS; CID	81.33	718.94	Ssna1

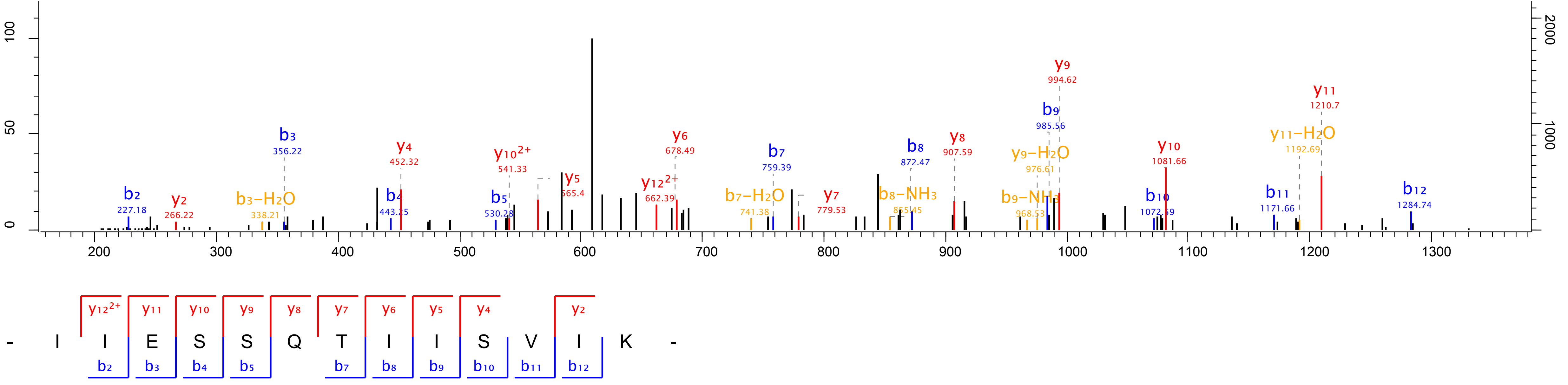

Raw file	Scan	Method	Score	m/z	Gene names
HBT_20130916_BV2_IL101_02	18934	ITMS; CID	121.77	980	Otud7b

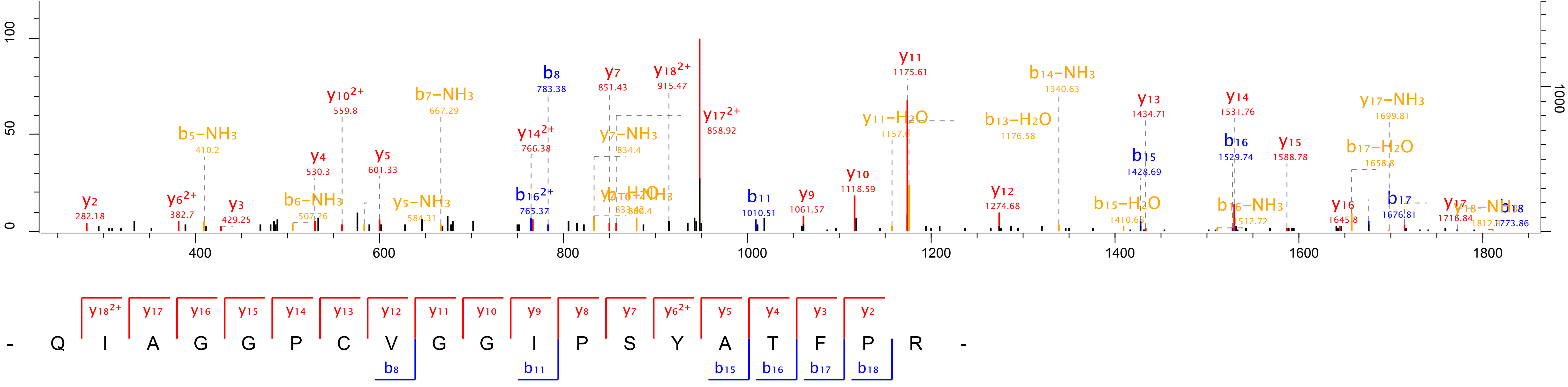

Raw file	Scan	Method	Score	m/z	Gene names
HBT_20130916_BV2_IL101_02	18622	ITMS; CID	119.88	755.39	Serpinb8

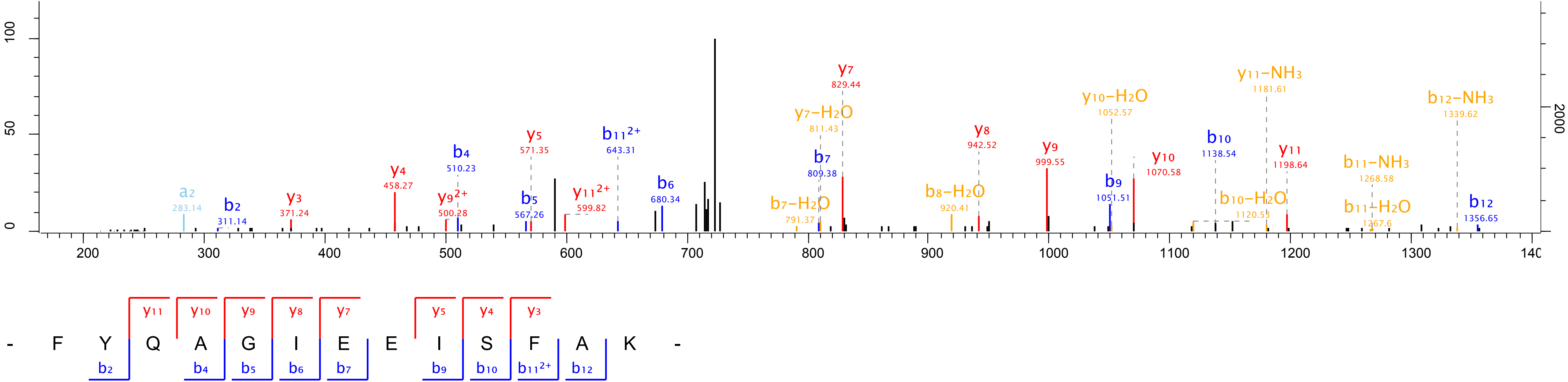

Raw file	Scan	Method	Score	m/z	Gene names
HBT_20130916_BV2_IL101_02	18300	ITMS; CID	79.87	740.94	Pol

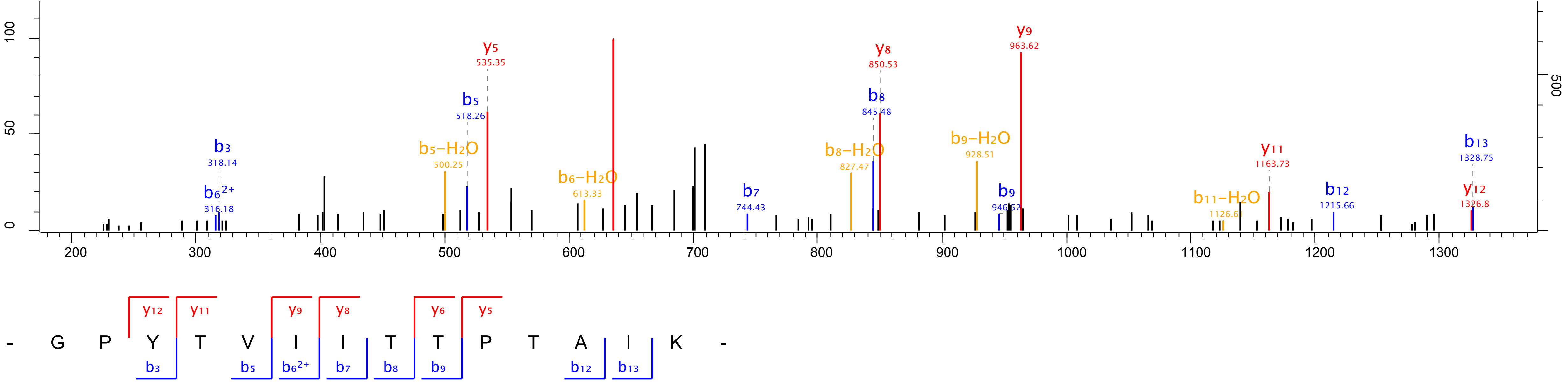

Raw file	Scan	Method	Score	m/z	Gene names
HBT_20130916_BV2_IL101_02	15889	ITMS; CID	150.05	811.46	Cln6

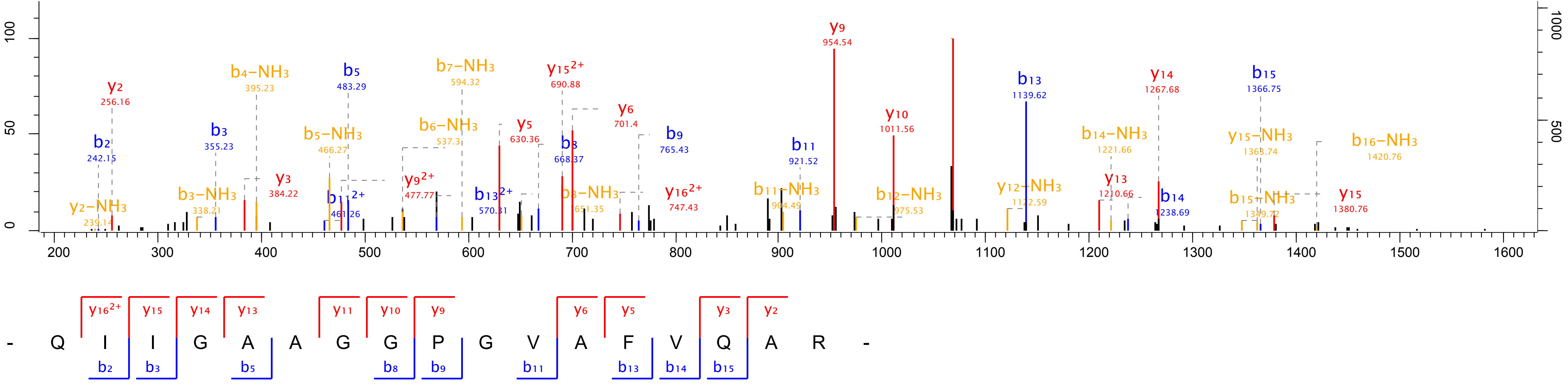

Raw file	Scan	Method	Score	m/z	Gene names
HBT_20130916_BV2_IL101_02	15827	ITMS; CID	130.1	633.83	Syngr1

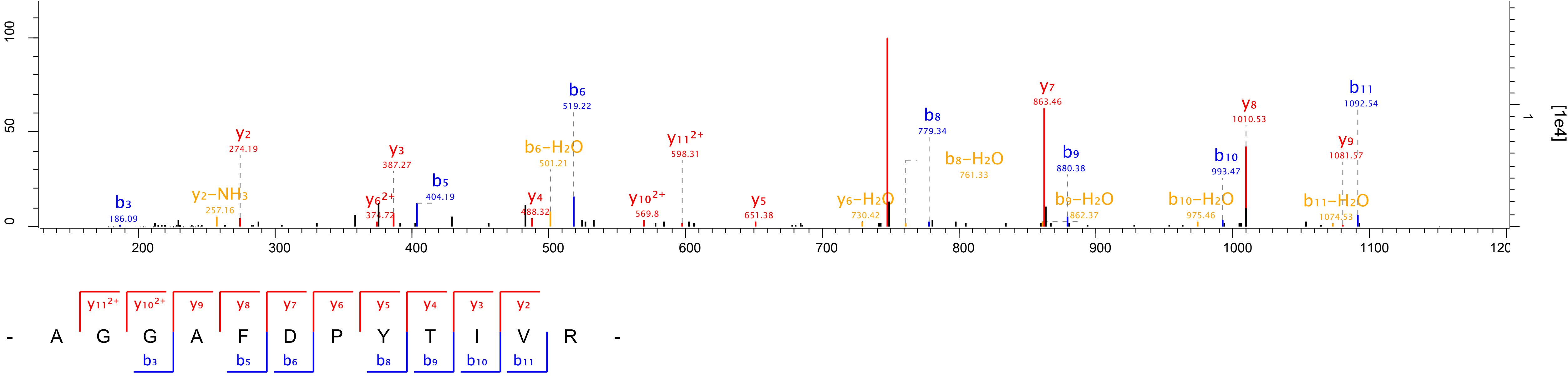

Raw file	Scan	Method	Score	m/z	Gene names
HBT_20130916_BV2_IL101_02	14575	ITMS; CID	101.72	756.4	Ctsf

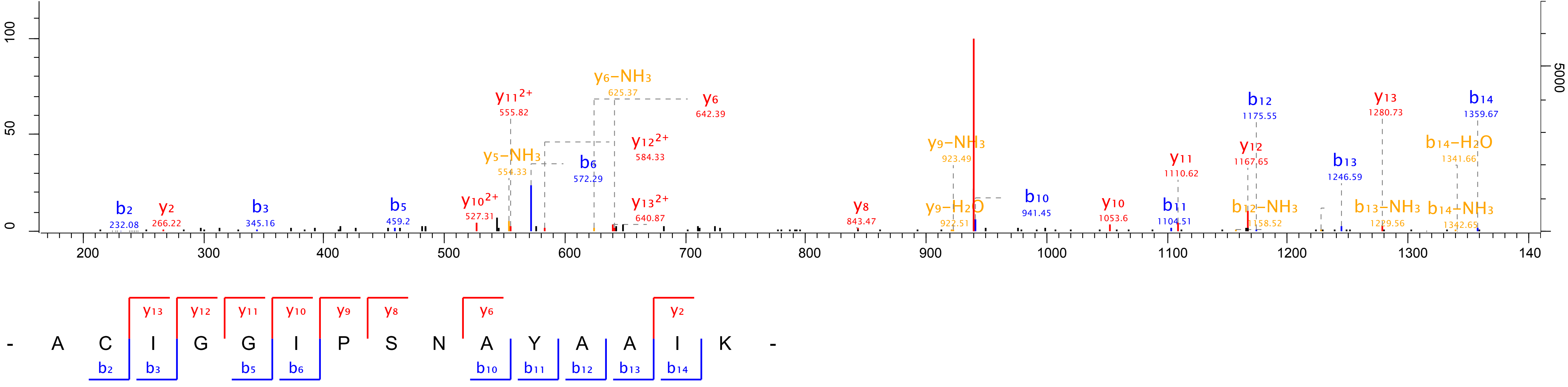

Raw file	Scan	Method	Score	m/z	Gene names
HBT_20130916_BV2_IL101_02	13775	ITMS; CID	115.23	557.32	Fau

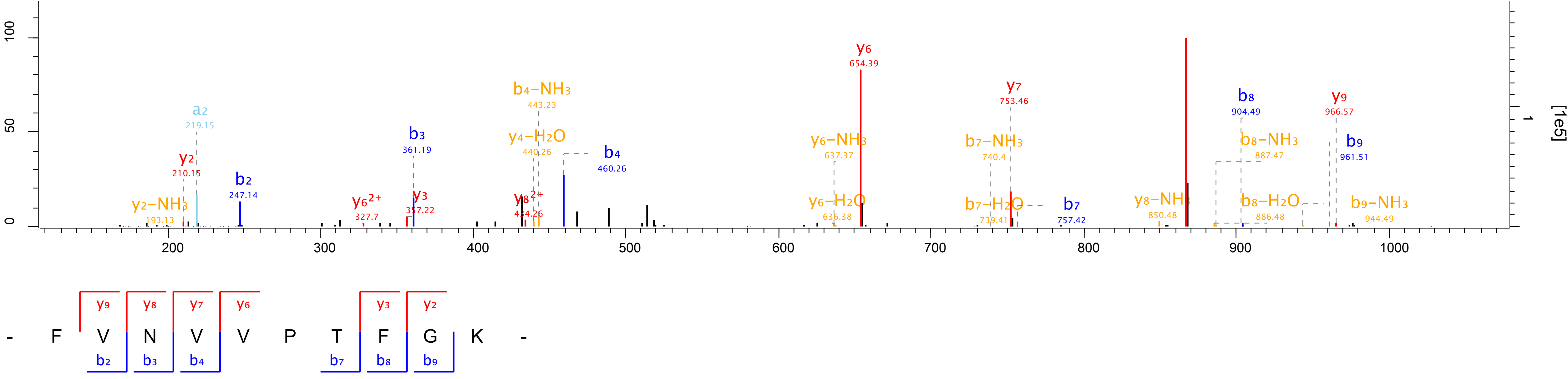

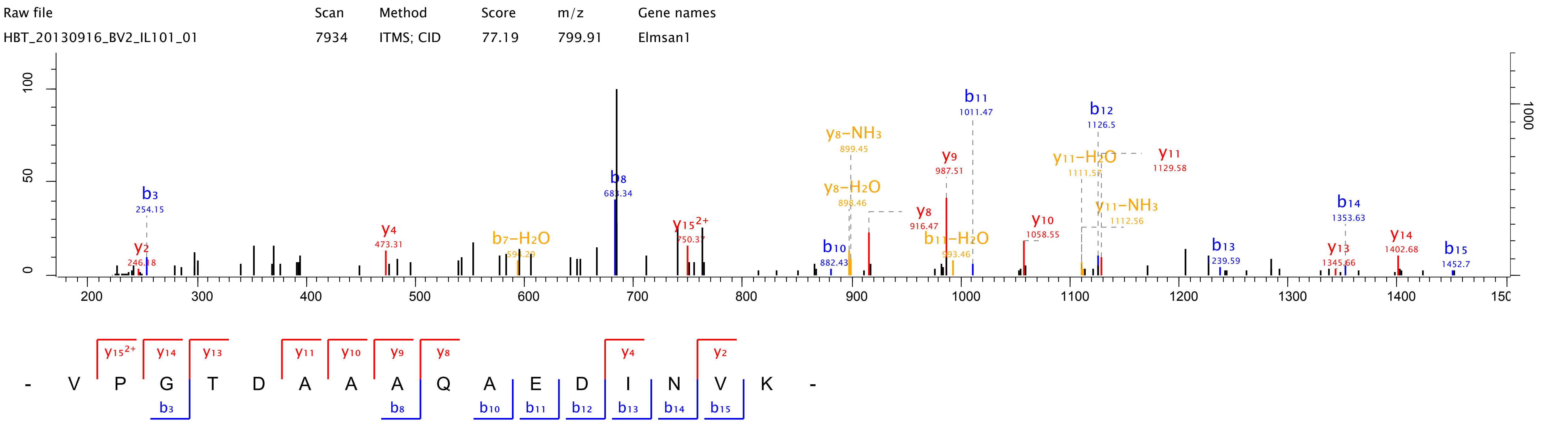
Raw file
Scan
Method
Score
m/z
Gene names
HBT_20130916_BV2_IL101_01
7934
ITMS; CID
77.19
799.91
Elmsan1
100
50
0
1000
b3 254.15
y2 246.18
y4 473.31
b7-H2O 595.29
b8 683.34
y15 2+ 750.37
b10 882.43
y8-NH3 899.45
y8-H2O 898.46
y8 916.47
y9 987.51
b11-H2O 993.46
b11 1011.47
y10 1058.55
y11-H2O 1111.57
y11-NH3 1112.56
b12 1126.5
y11 1129.58
b13 1239.59
y13 1345.66
b14 1353.63
y14 1402.68
b15 1452.7
200
300
400
500
600
700
800
900
1000
1100
1200
1300
1400
150
- V P G T D A A A Q A E D I N V K -
y15 2+
y14
y13
y11
y10
y9
y8
y4
y2
b3
b8
b10
b11
b12
b13
b14
b15

Raw file	Scan	Method	Score	m/z	Gene names
HBT_20130916_BV2_IL101_01	7518	ITMS; CID	136.57	841.36	mt-Nd3;Mtnd3

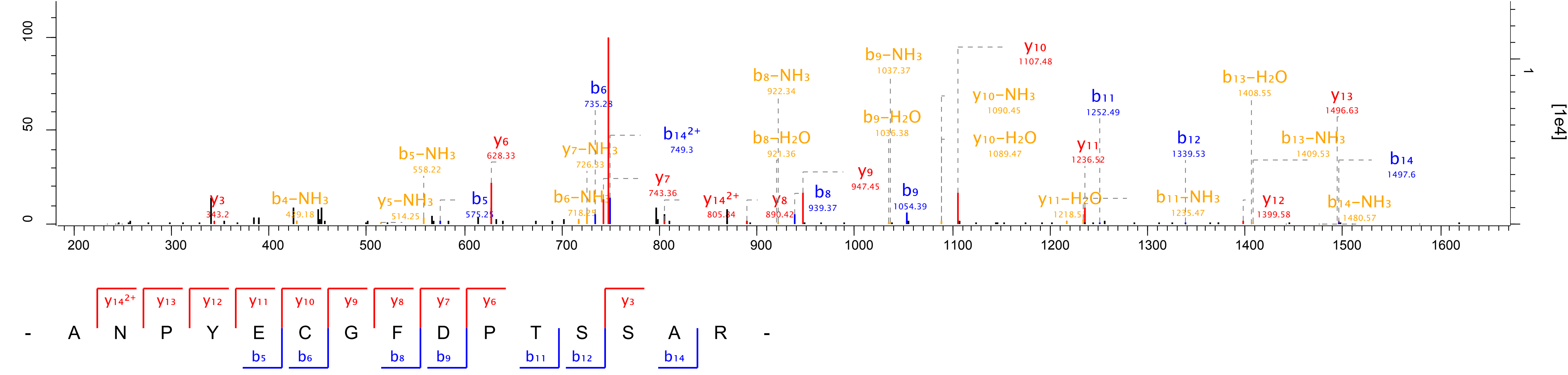

Raw file	Scan	Method	Score	m/z	Gene names
HBT_20130916_BV2_IL101_01	6716	ITMS; CID	170.95	710.33	Vkorc1

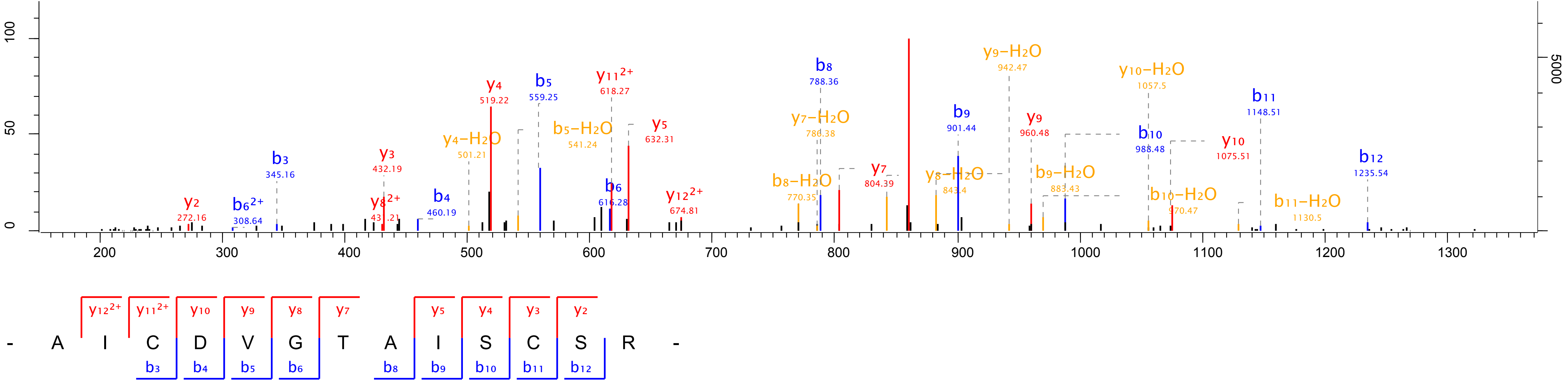

Raw file	Scan	Method	Score	m/z	Gene names
HBT_20130916_BV2_IL101_01	4697	ITMS; CID	88.95	746.42	Guk1

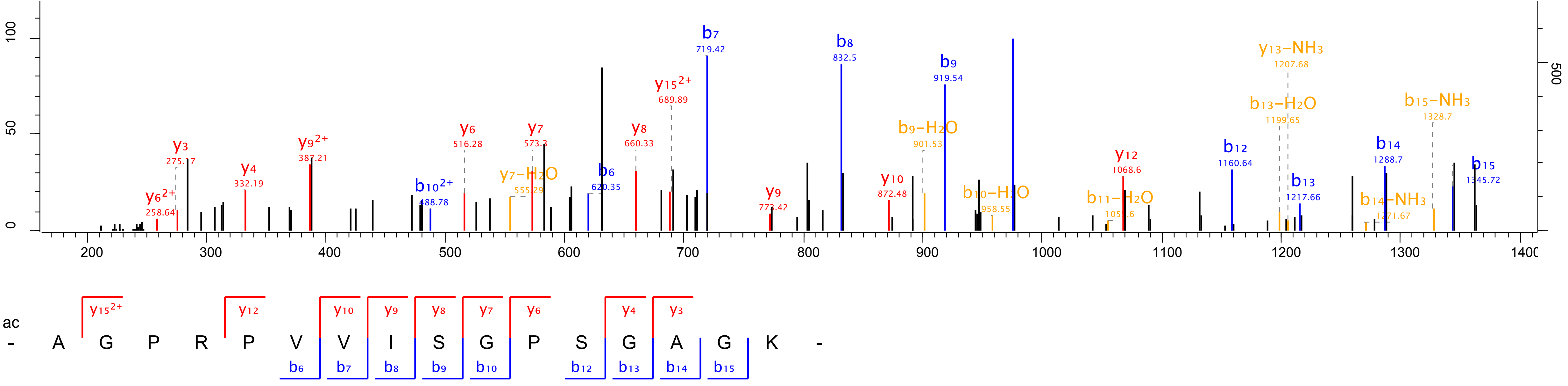

Raw file	Scan	Method	Score	m/z	Gene names
HBT_20130916_BV2_IL101_01	28439	ITMS; CID	99.39	882.53	Tk1

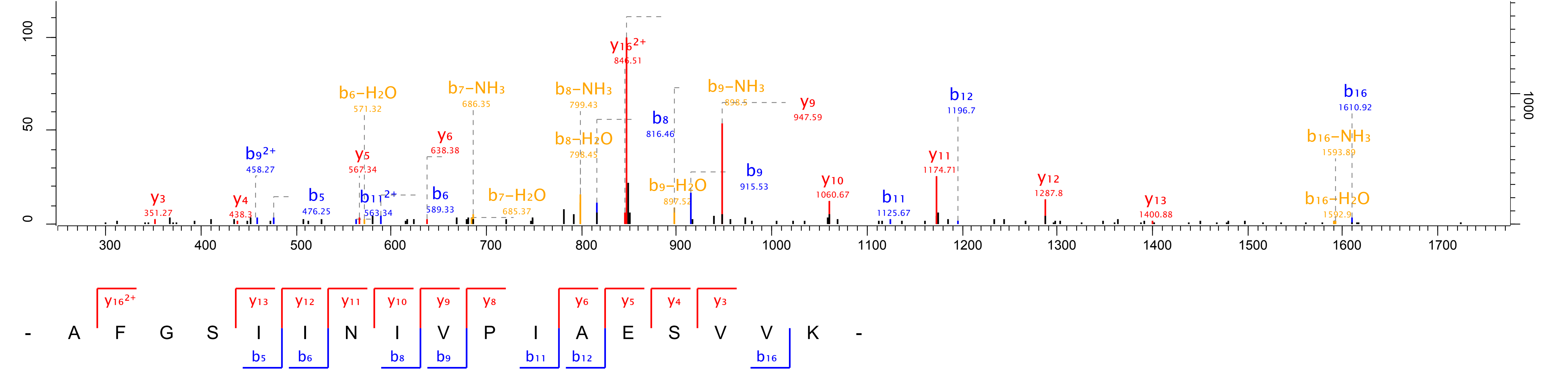

Raw file	Scan	Method	Score	m/z	Gene names
HBT_20130916_BV2_IL101_01	2451	ITMS; CID	106.62	627.82	Tuft1

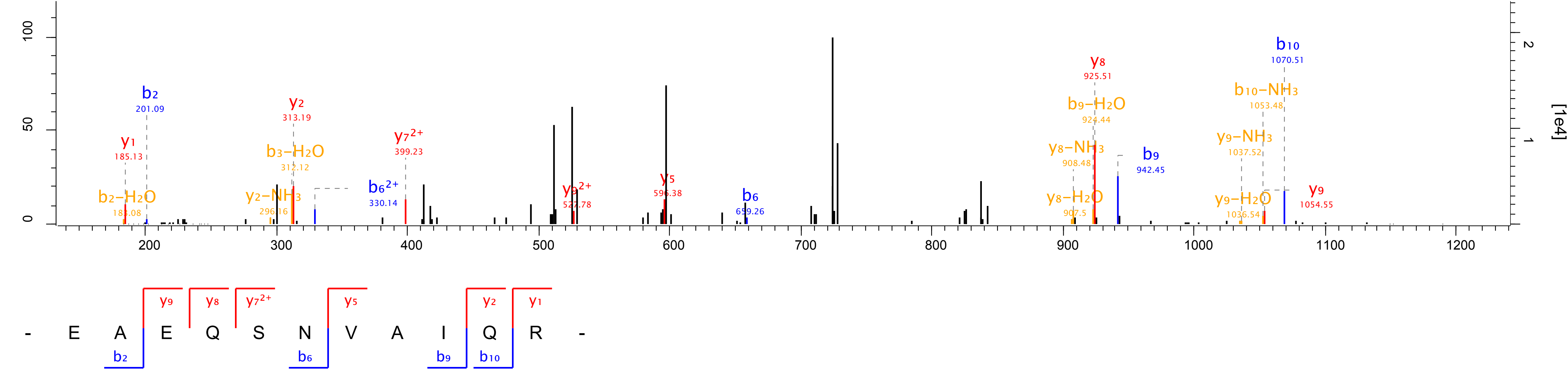

Raw file	Scan	Method	Score	m/z
HBT_20130916_BV2_IL101_01	23842	ITMS; CID	48.9	746.41

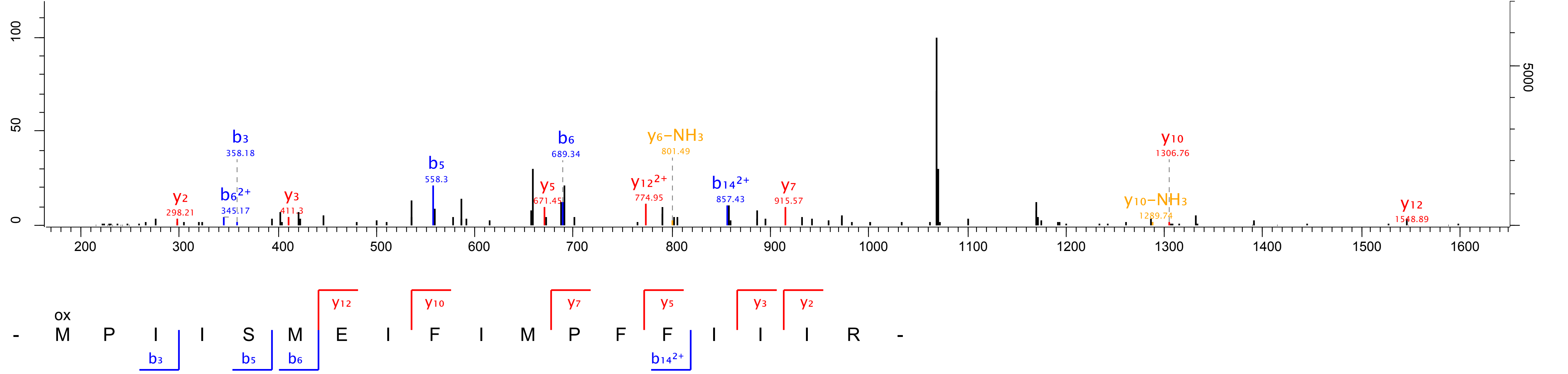

Raw file	Scan	Method	Score	m/z	Gene names
HBT_20130916_BV2_IL101_01	2382	ITMS; CID	132.56	636.29	Vps37c

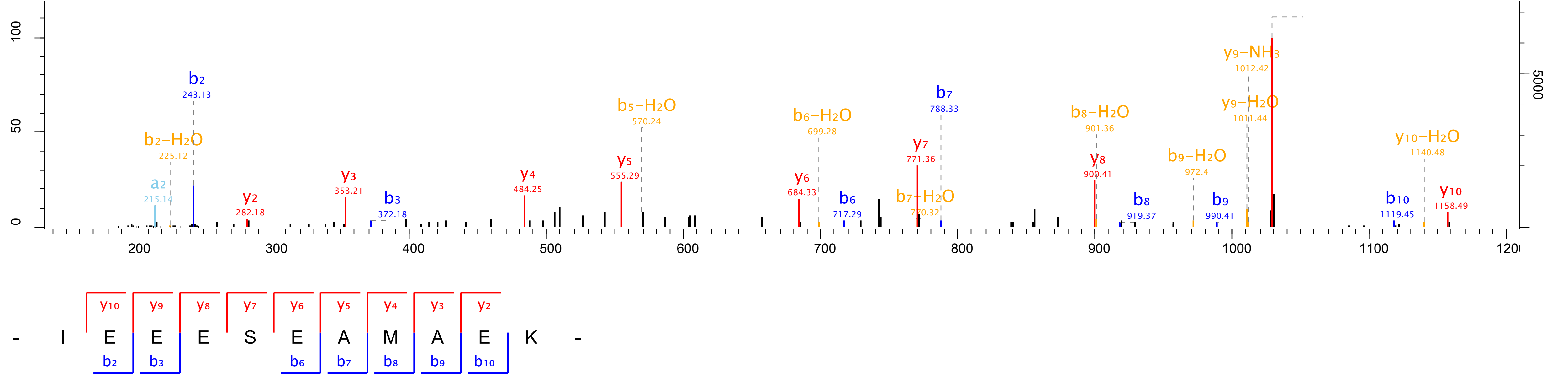

Raw file	Scan	Method	Score	m/z	Gene names
HBT_20130916_BV2_IL101_01	2113	ITMS; CID	116.05	515.26	Nudt11;Nudt10;Nudt4

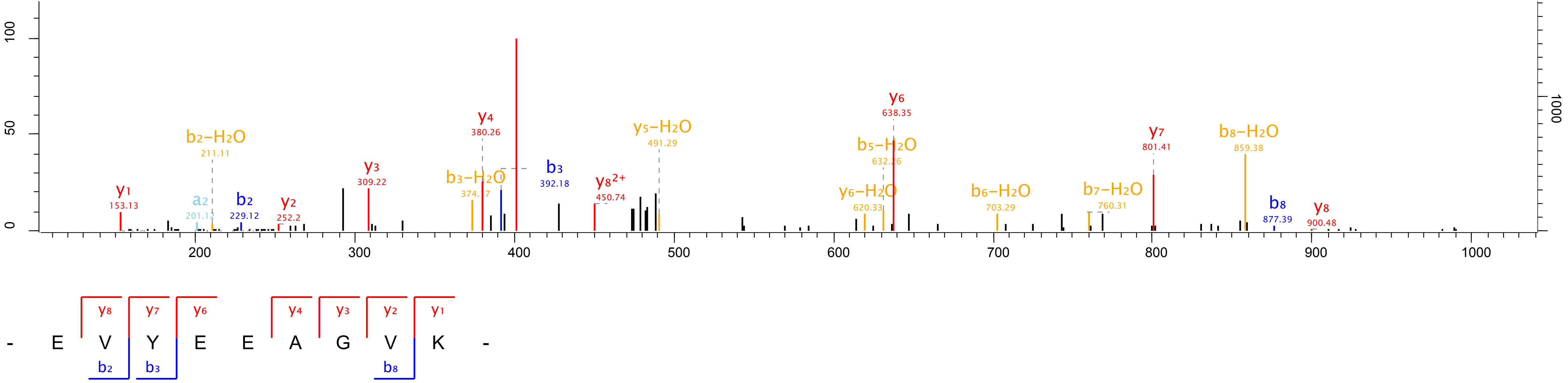

Raw file	Scan	Method	Score	m/z	Gene names
HBT_20130916_BV2_IL101_01	17515	ITMS; CID	182.92	629.84	Gm5435;Gtf2a1

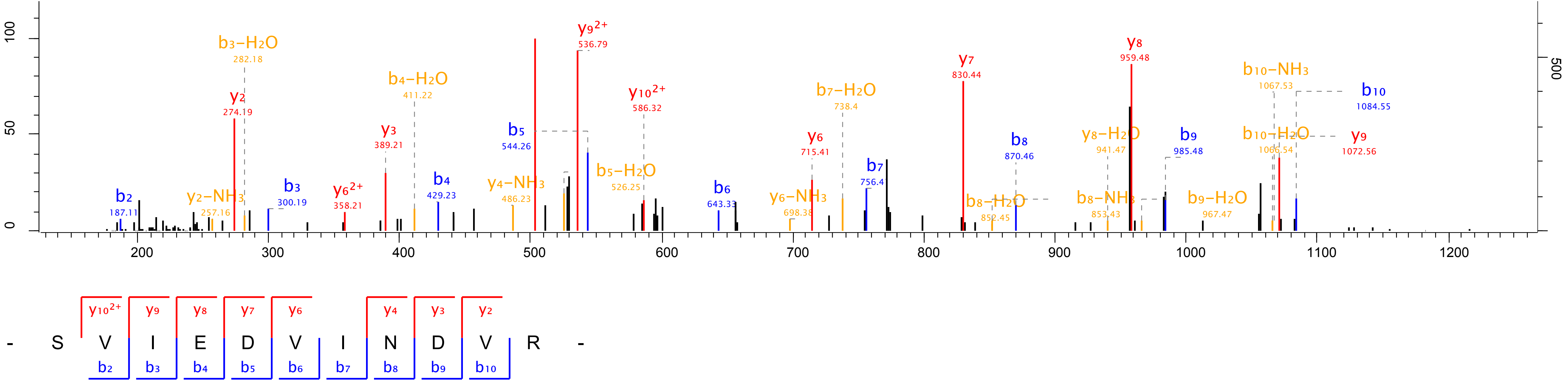

Raw file	Scan	Method	Score	m/z	Gene names
HBT_20130916_BV2_IL101_01	13631	ITMS; CID	64.52	885.92	2810021J22Rik

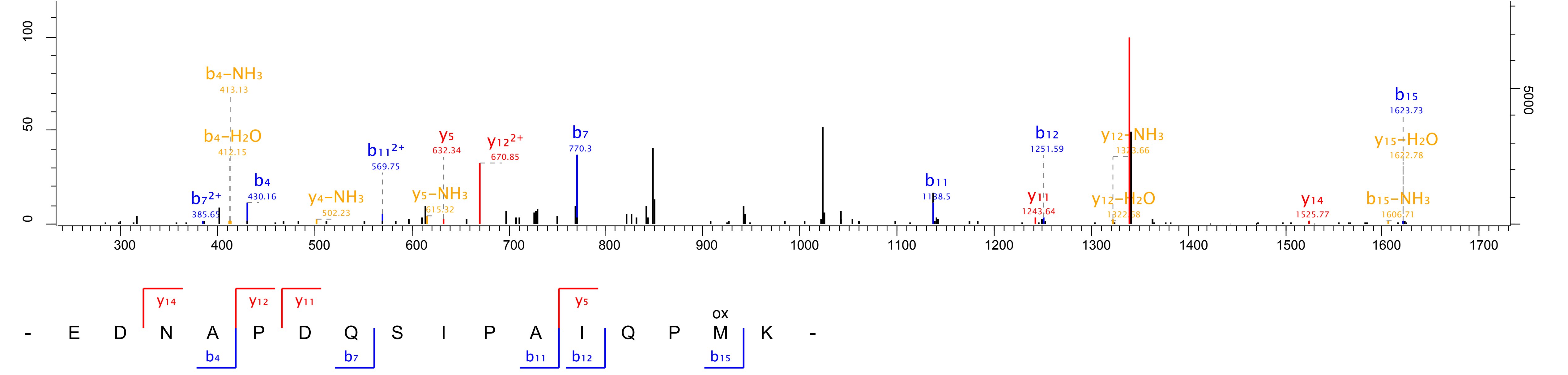

Raw file	Scan	Method	Score	m/z	Gene names
HBT_20130916_BV2_IC3_06	9075	ITMS; CID	131.86	891	Slc35a3

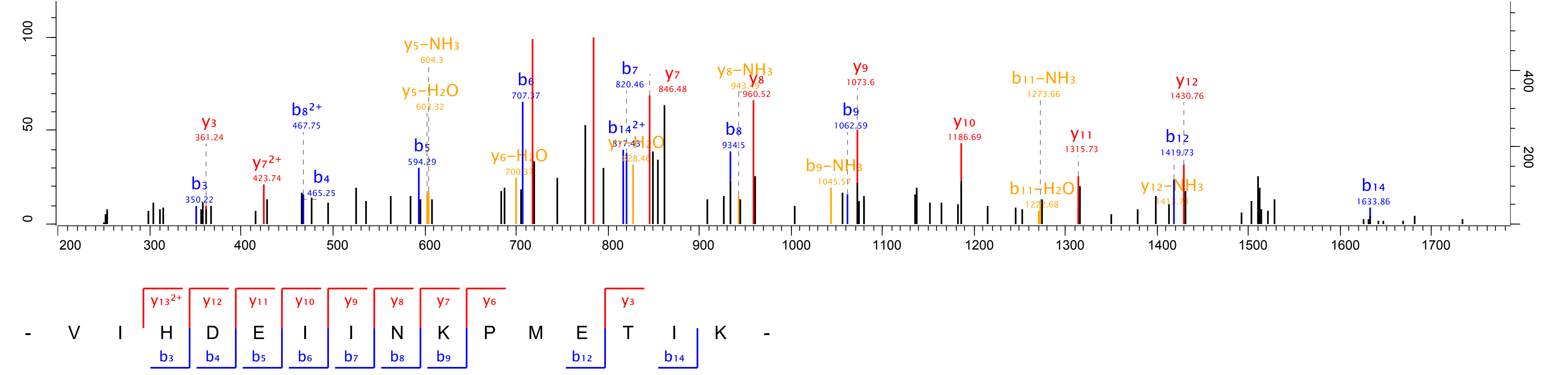

Raw file	Scan	Method	Score	m/z	Gene names
HBT_20130916_BV2_IC3_06	3963	ITMS; CID	106.44	940.47	mt-Nd5;Mtnd5

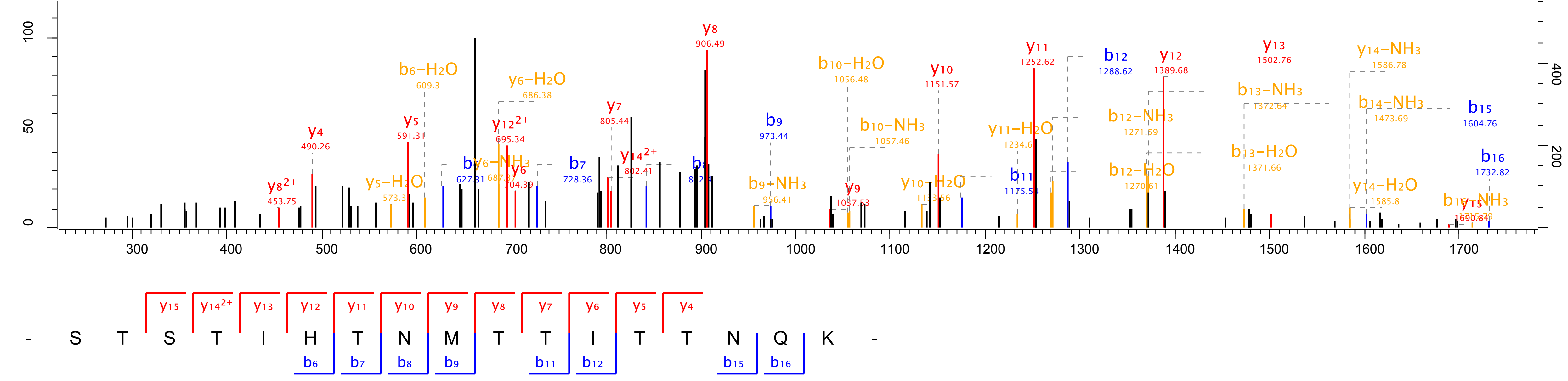

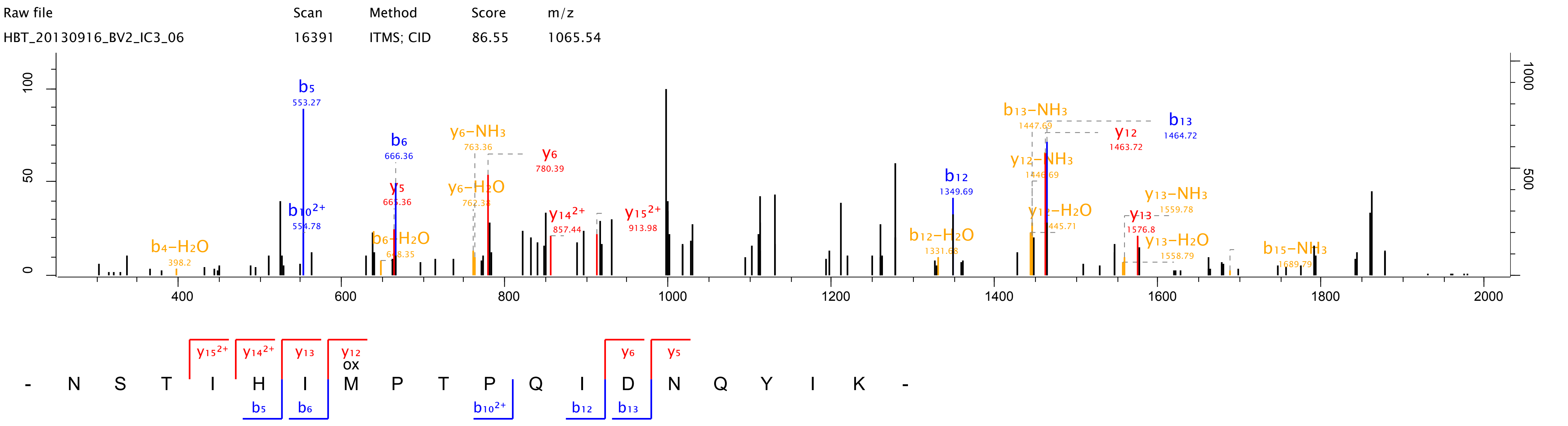

Raw file
HBT_20130916_BV2_IC3_06
Scan
16391
Method
ITMS; CID
Score
86.55
m/z
1065.54
b5
553.27
b6
666.36
y5
665.36
b10 2+
554.78
b4-H2O
398.2
b6-H2O
648.35
y6-NH3
763.36
y6-H2O
762.38
y6
780.39
y14 2+
857.44
y15 2+
913.98
b12
1349.69
b12-H2O
1331.68
b13-NH3
1447.69
b13
1464.72
y12
1463.72
y12-NH3
1446.69
y12-H2O
1445.71
y13
1576.8
y13-NH3
1559.78
y13-H2O
1558.79
b15-NH3
1689.79
0
50
100
500
1000
400
600
800
1000
1200
1400
1600
1800
2000
y15 2+
y14 2+
y13
y12
ox
y6
y5
- N S T I H I M P T P Q I D N Q Y I K -
b5
b6
b10 2+
b12
b13

Raw file	Scan	Method	Score	m/z	Gene names
HBT_20130916_BV2_IC3_06	1489	ITMS; CID	96.43	803.89	Rnaset2

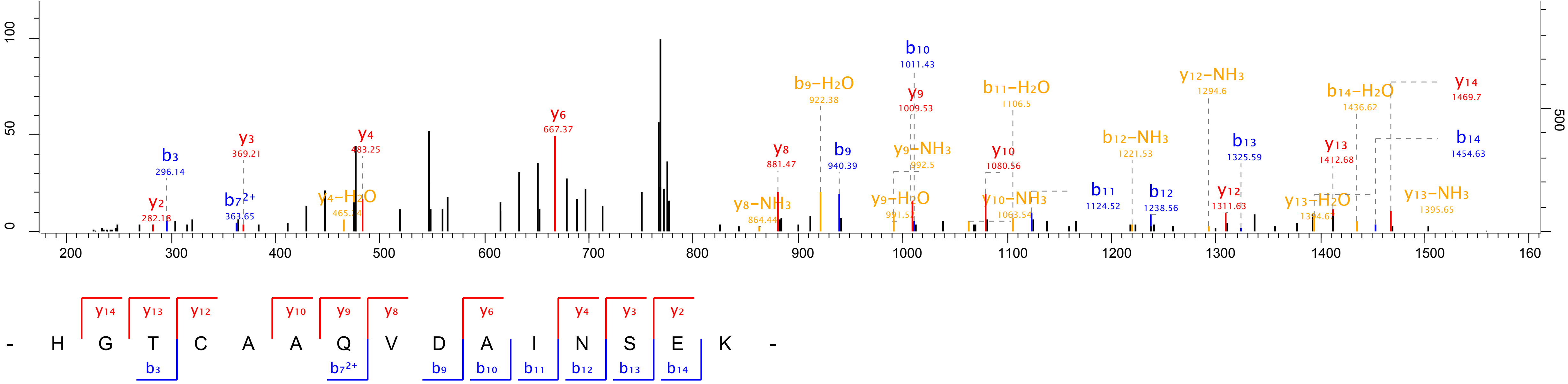

Raw file	Scan	Method	Score	m/z	Gene names
HBT_20130916_BV2_IC3_06	13916	ITMS; CID	86.37	1046.51	Slc43a3

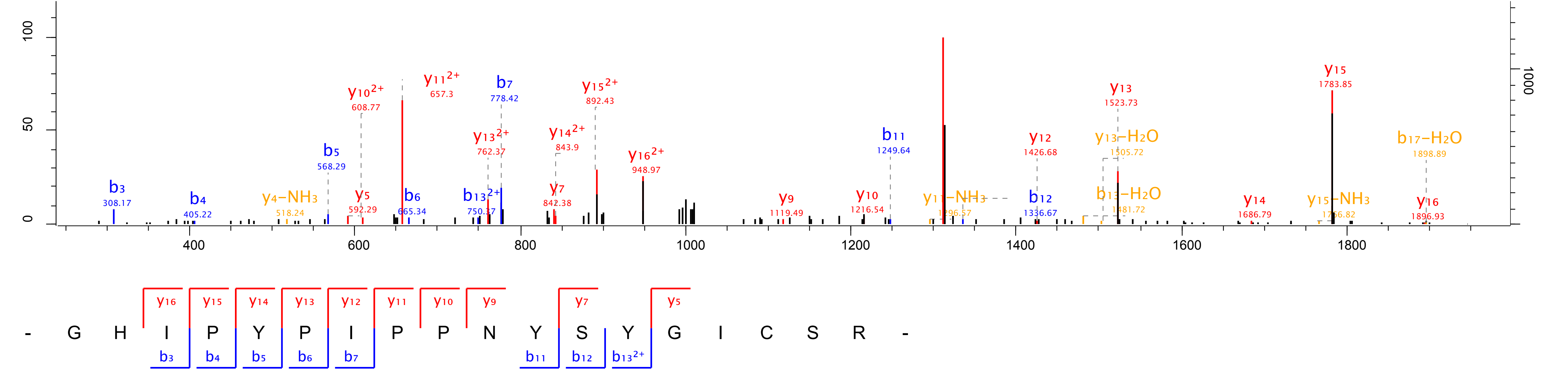

Raw file	Scan	Method	Score	m/z
HBT_20130916_BV2_IC3_05	13360	ITMS; CID	62.24	971.96

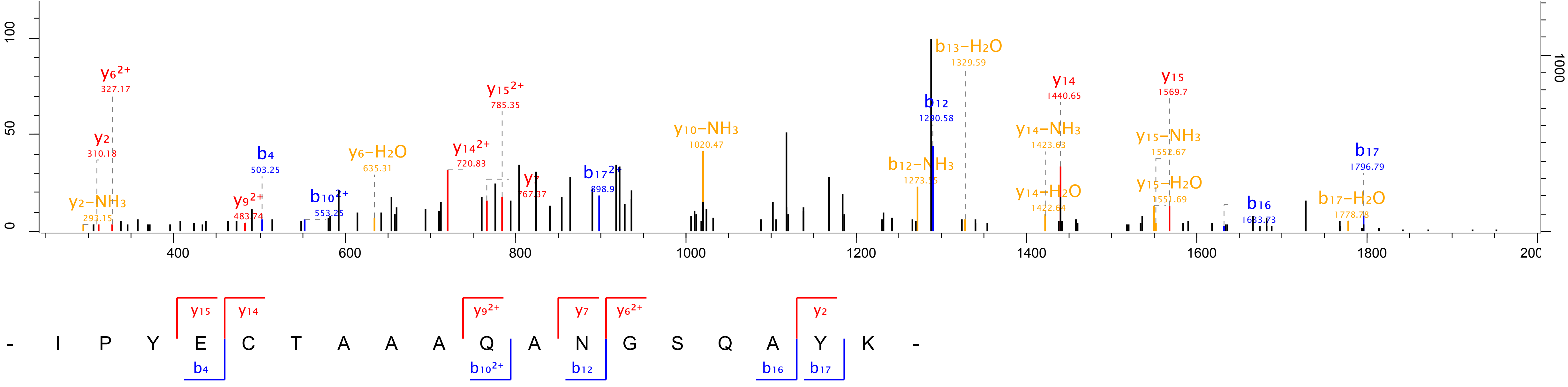

Raw file	Scan	Method	Score	m/z	Gene names
HBT_20130916_BV2_IC3_04	21866	ITMS; CID	41.21	1067.22	Fam20c

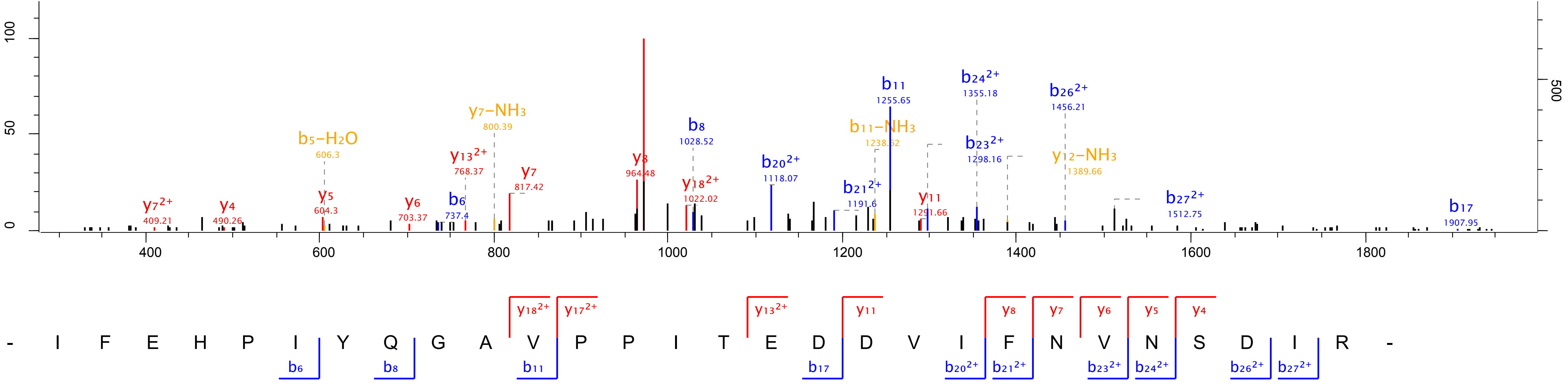

Raw file	Scan	Method	Score	m/z	Gene names
HBT_20130916_BV2_IC3_04	13951	ITMS; CID	78.19	994.97	Erf

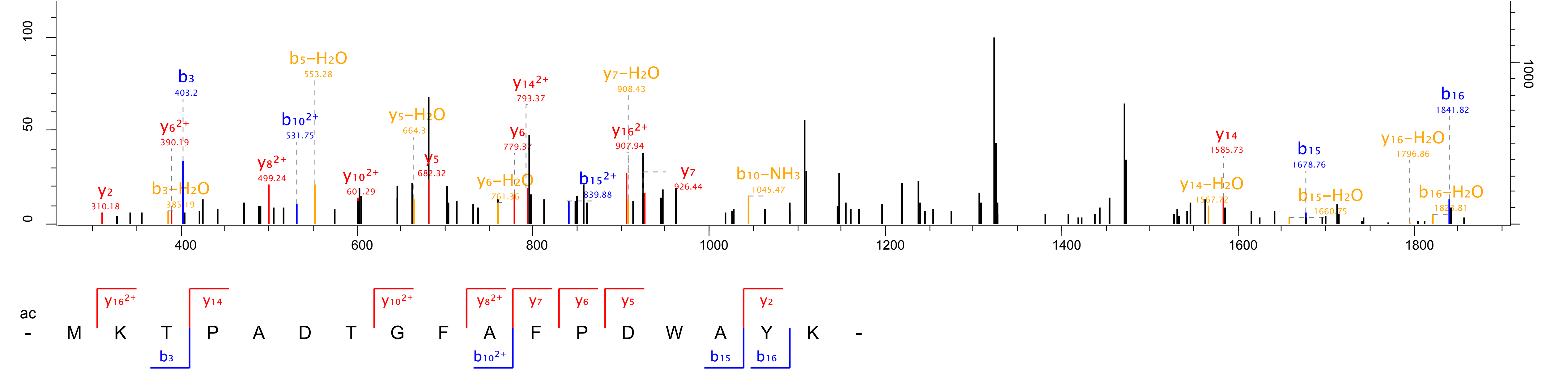

Raw file	Scan	Method	Score	m/z	Gene names
HBT_20130916_BV2_IC3_03	27993	ITMS; CID	88.56	920.5	Ggcx

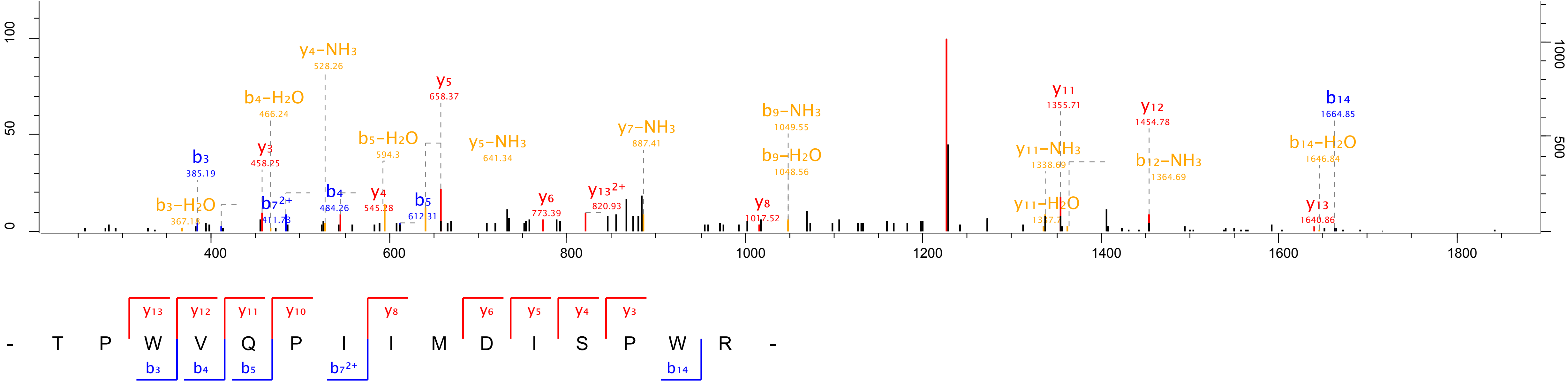

Raw file	Scan	Method	Score	m/z	Gene names
HBT_20130916_BV2_IC3_03	26120	ITMS; CID	64.8	590.31	Kifap3

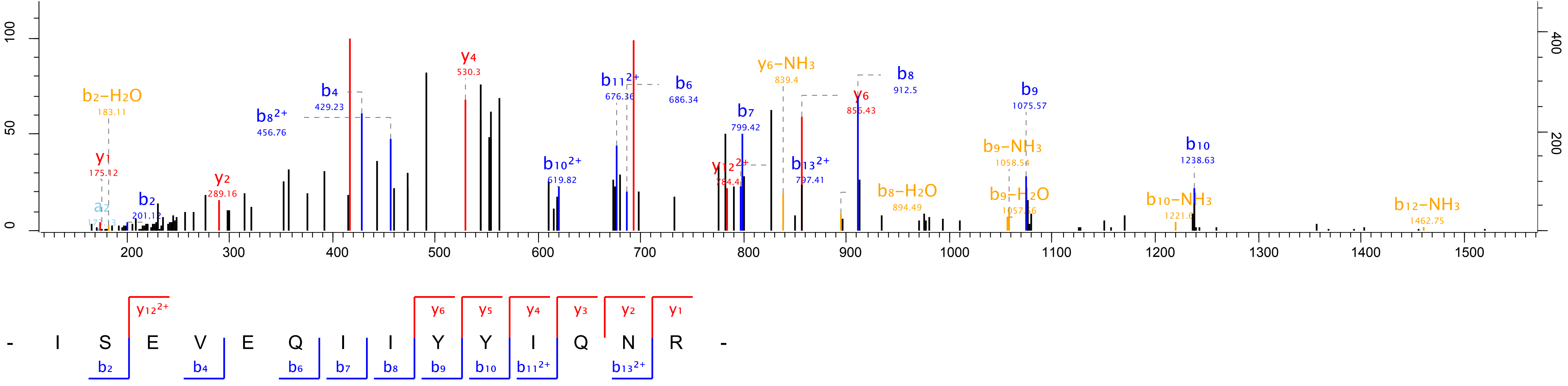

Raw file	Scan	Method	Score	m/z	Gene names
HBT_20130916_BV2_IC3_03	26027	ITMS; CID	175.66	801.93	Nsmce2

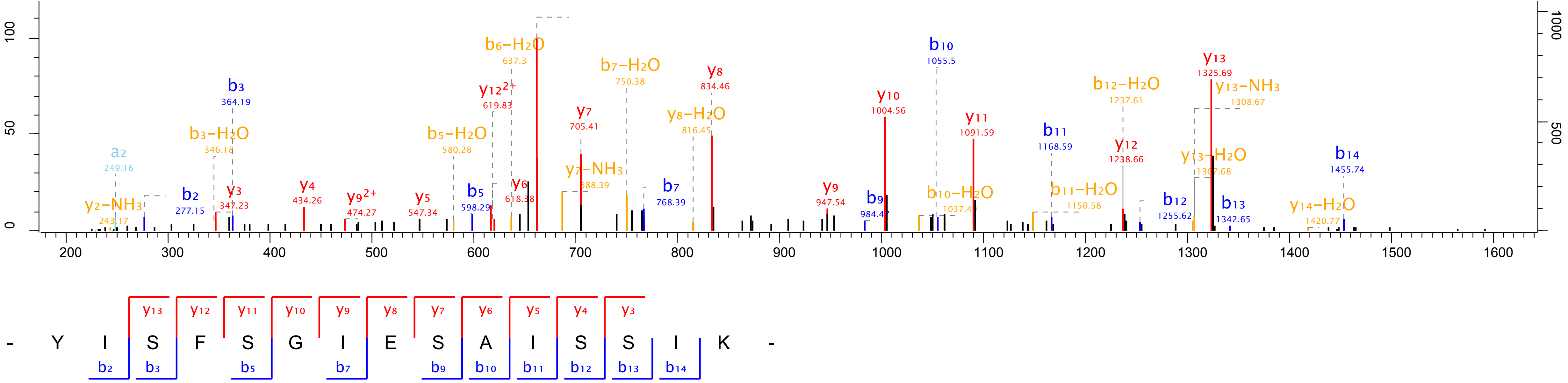

Raw file	Scan	Method	Score	m/z	Gene names
HBT_20130916_BV2_IC3_03	24877	ITMS; CID	163.33	642.39	Nde1;Ndel1

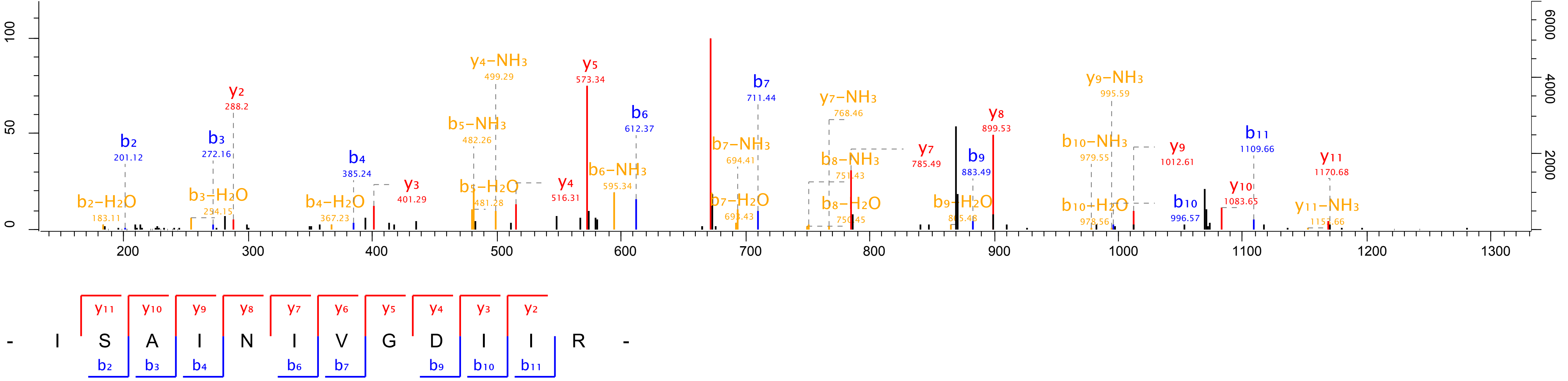

Raw file	Scan	Method	Score	m/z	Gene names
HBT_20130916_BV2_IC3_03	24773	ITMS; CID	109.87	1043.49	Mllt11

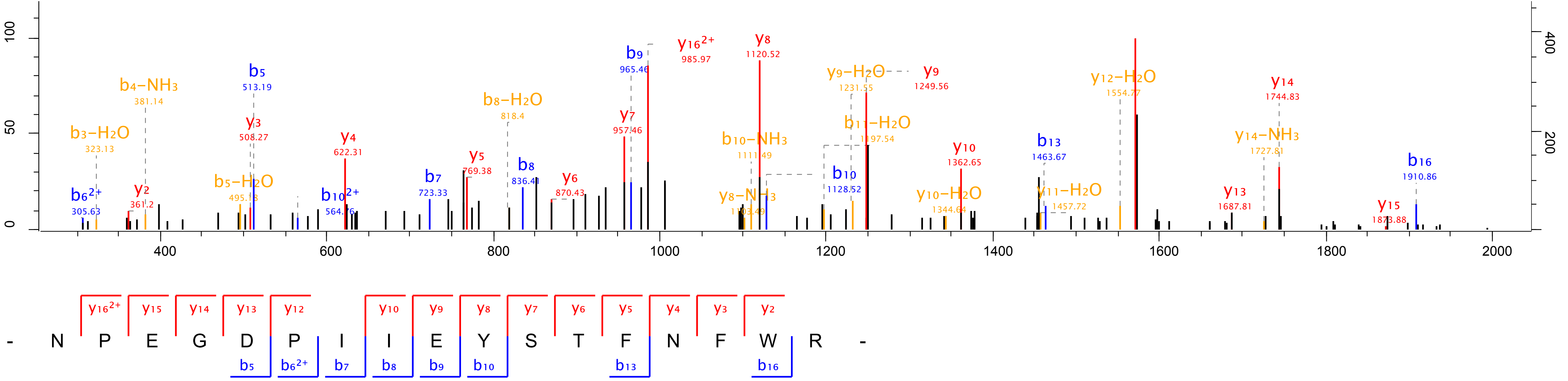

Raw file	Scan	Method	Score	m/z	Gene names
HBT_20130916_BV2_IC3_03	24665	ITMS; CID	181.75	693.41	Tmem176b

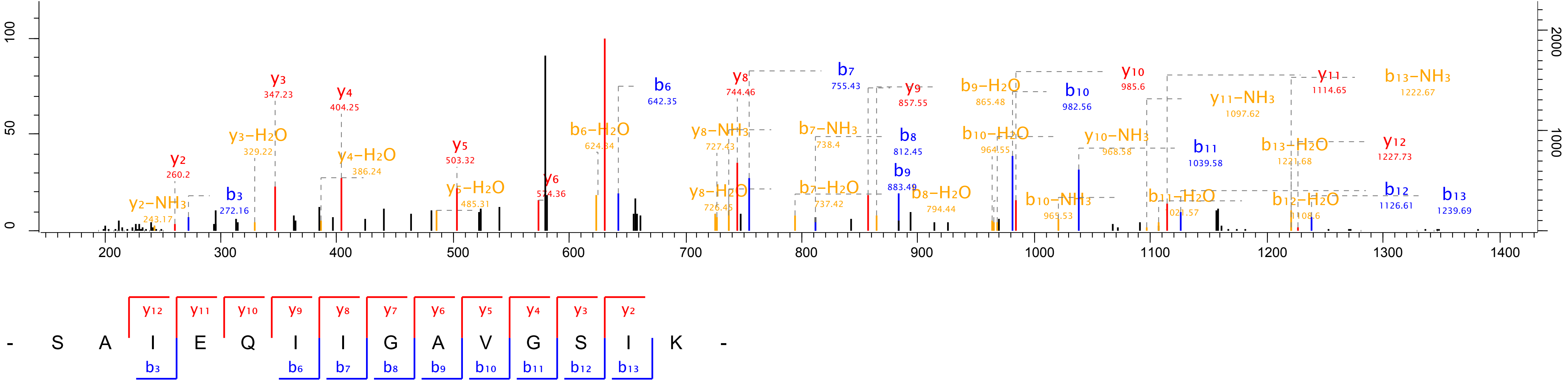

Raw file	Scan	Method	Score	m/z	Gene names
HBT_20130916_BV2_IC3_03	24612	ITMS; CID	97.3	1149.16	Vmp1

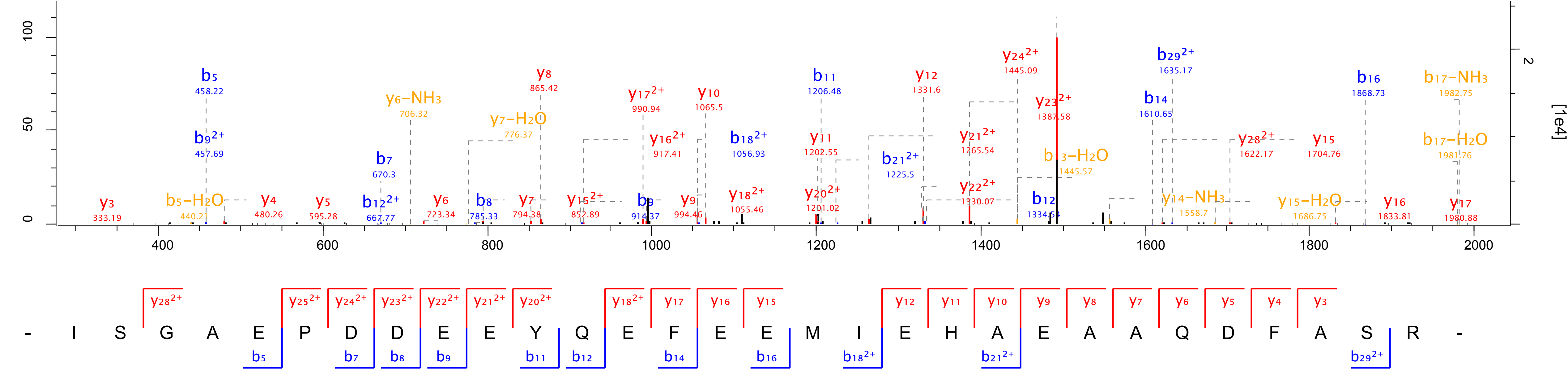

Raw file	Scan	Method	Score	m/z
HBT_20130916_BV2_IC3_03	23496	ITMS; CID	64.1	545.28

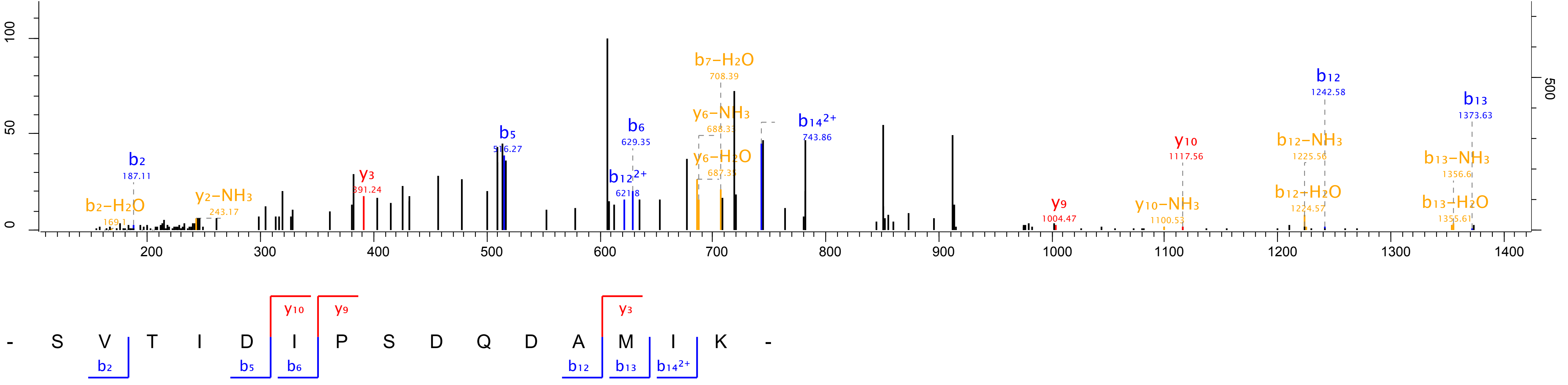

Raw file	Scan	Method	Score	m/z	Gene names
HBT_20130916_BV2_IC3_03	10241	ITMS; CID	99.73	668.87	Vma21

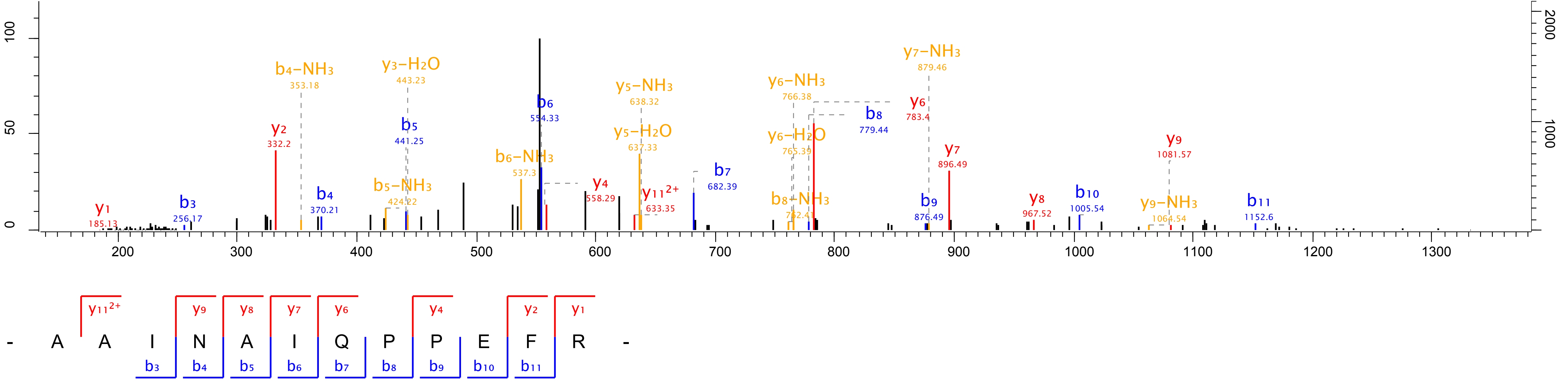

Raw file	Scan	Method	Score	m/z	Gene names
HBT_20130916_BV2_IC3_02	9988	ITMS; CID	60.63	1030.49	Cmtm7

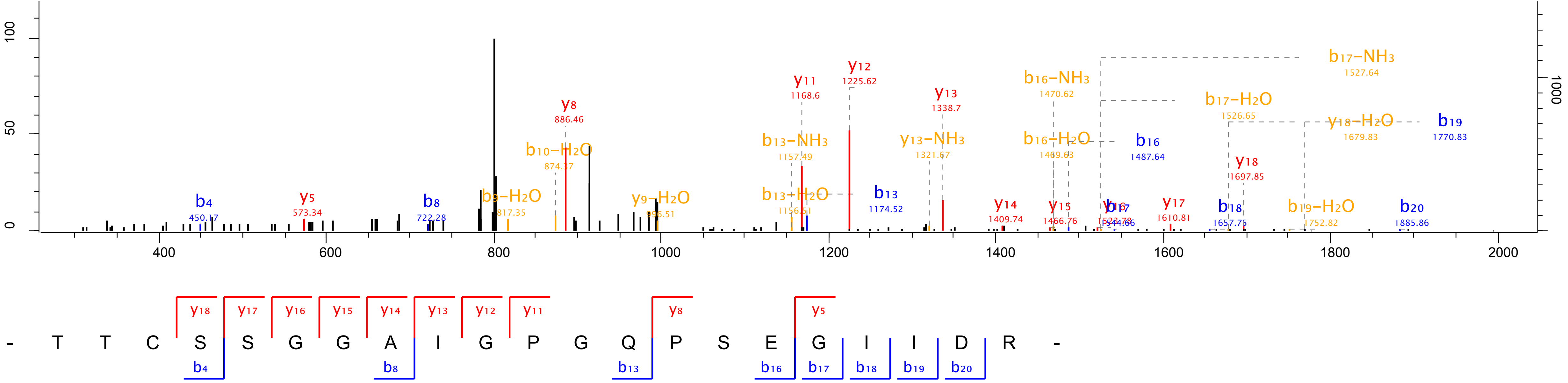

Raw file	Scan	Method	Score	m/z	Gene names
HBT_20130916_BV2_IC3_02	976	ITMS; CID	91.88	971.44	Rbms2

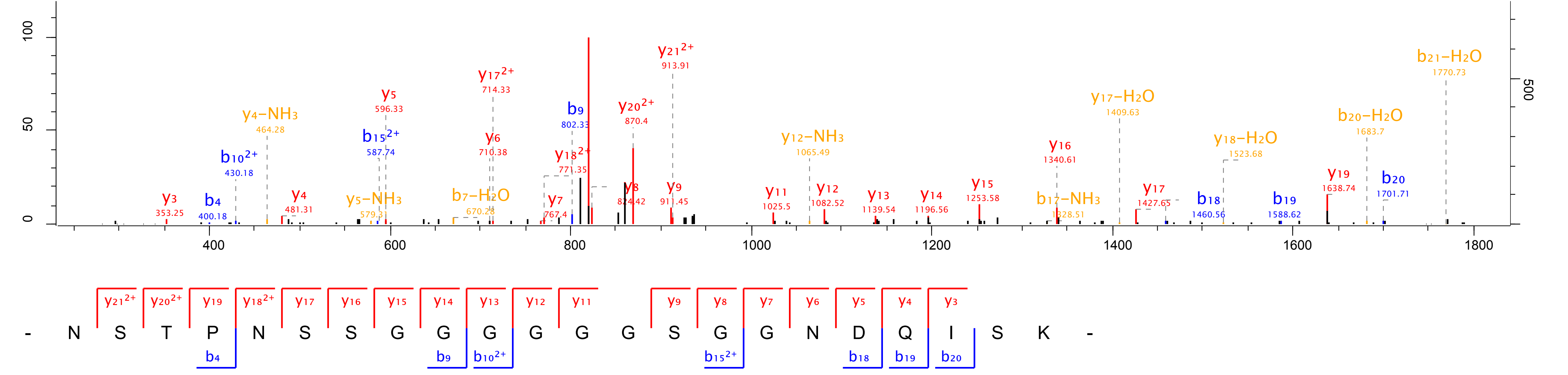

Raw file	Scan	Method	Score	m/z	Gene names
HBT_20130916_BV2_IC3_02	9579	ITMS; CID	95.57	795.89	Chmp6

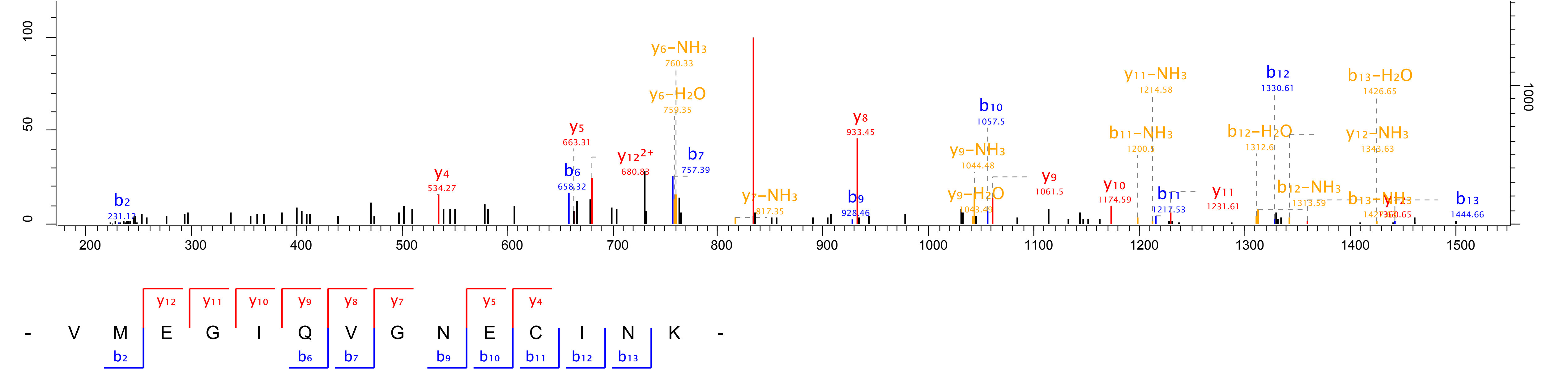

Raw file	Scan	Method	Score	m/z	Gene names
HBT_20130916_BV2_IC3_02	908	ITMS; CID	180.56	779.84	Rbmx2

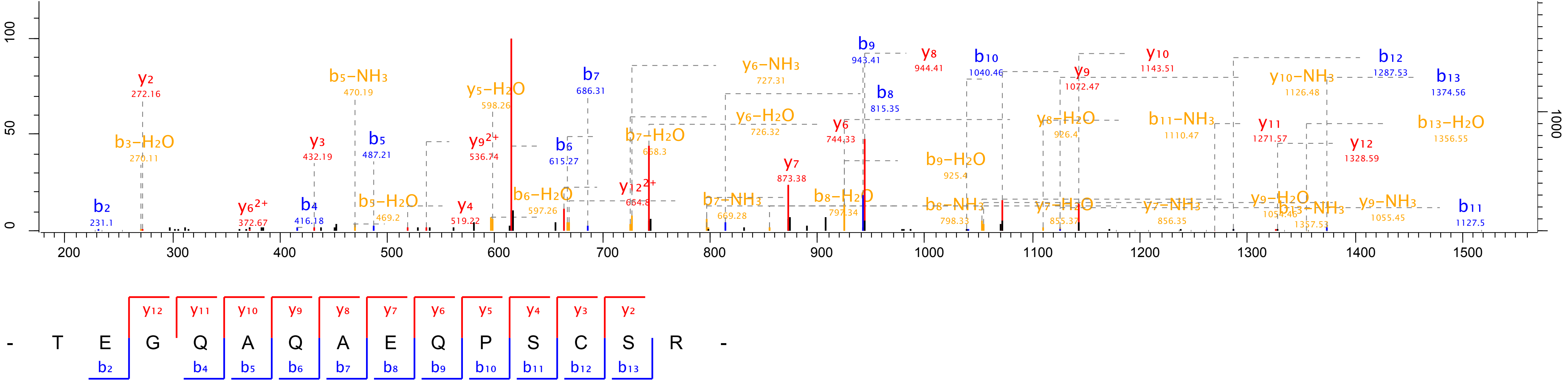

Raw file	Scan	Method	Score	m/z	Gene names
HBT_20130916_BV2_IC3_02	888	ITMS; CID	77.19	767.82	Txlng;Gm8258

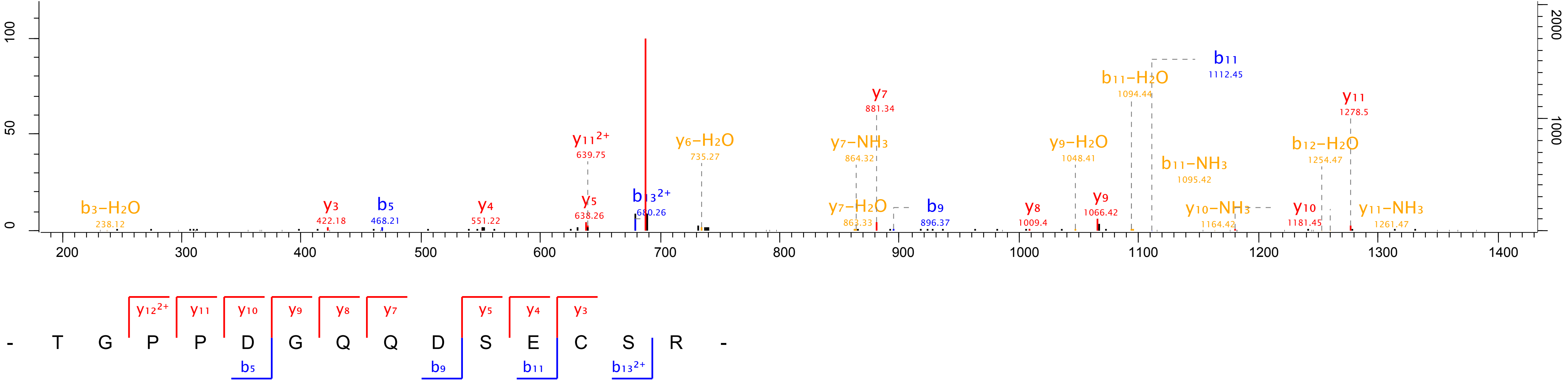

Raw file	Scan	Method	Score	m/z	Gene names
HBT_20130916_BV2_IC3_02	8820	ITMS; CID	218.02	709.34	Mgst1

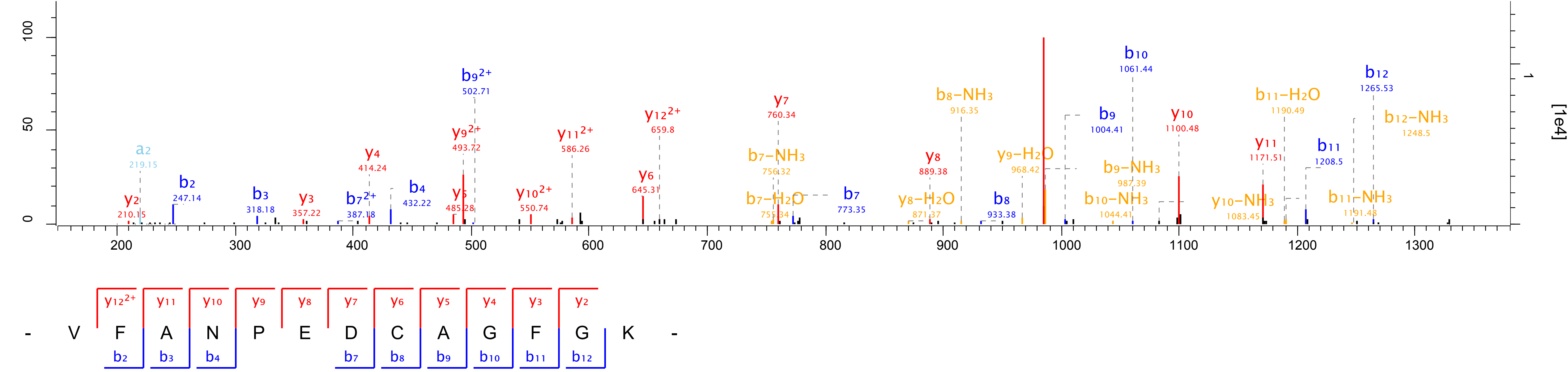

Raw file	Scan	Method	Score	m/z	Gene names
HBT_20130916_BV2_IC3_02	801	ITMS; CID	119.72	1044.95	Rpl24

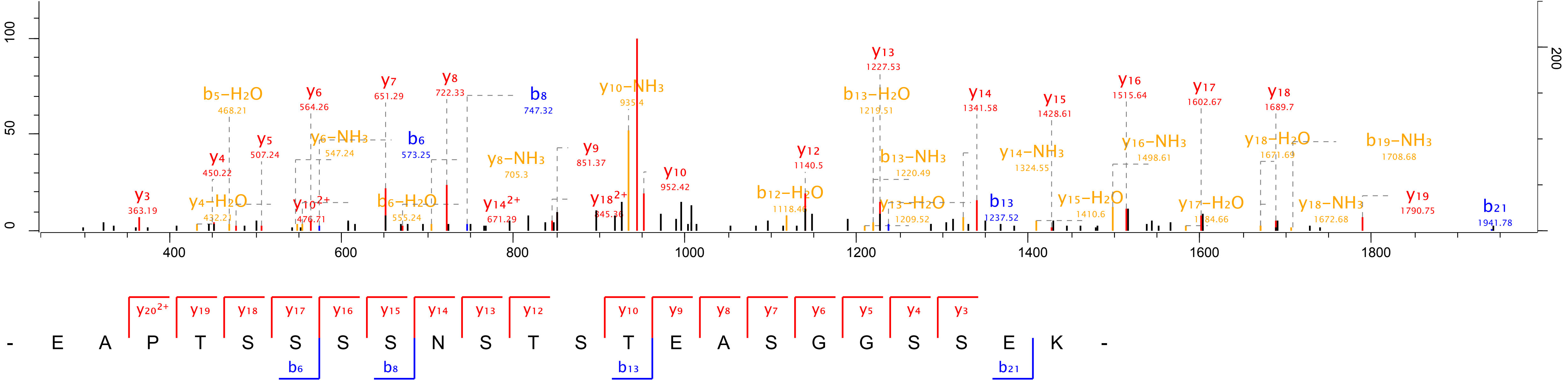

Raw file	Scan	Method	Score	m/z	Gene names
HBT_20130916_BV2_IC3_02	6216	ITMS; CID	80.32	671.29	Slc9a6

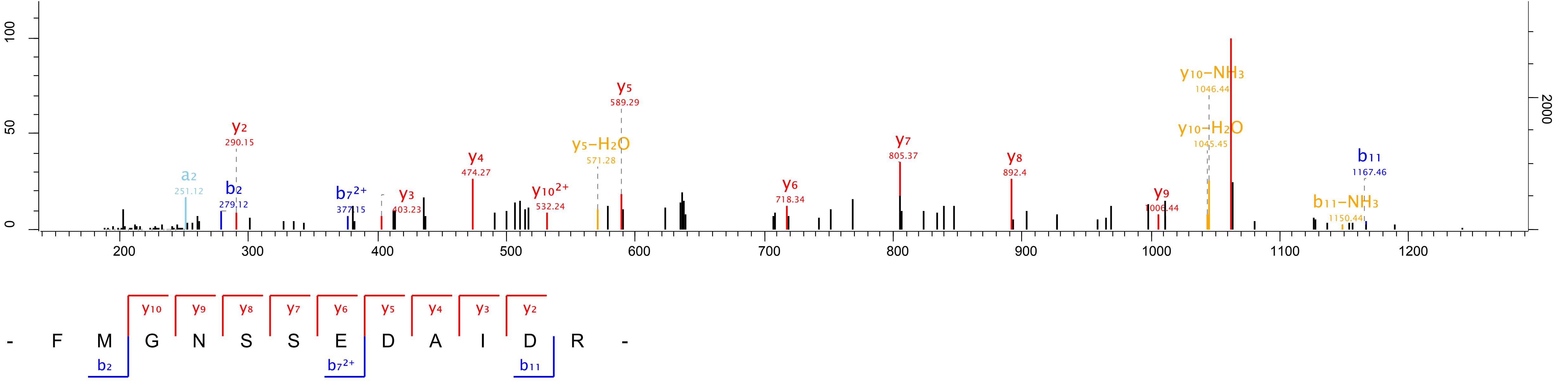

Raw file	Scan	Method	Score	m/z
HBT_20130916_BV2_IC3_02	3813	ITMS; CID	99.57	594.3

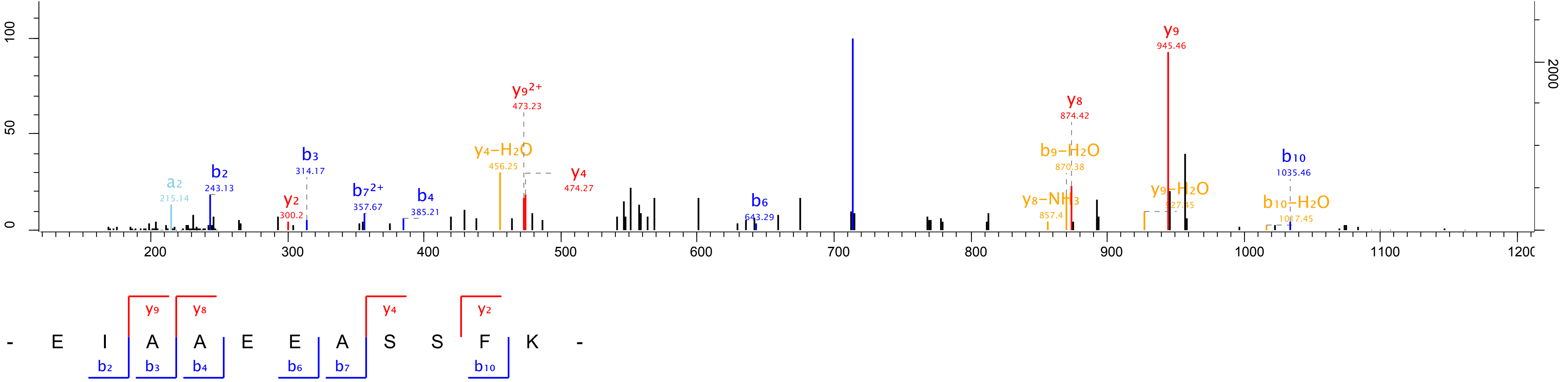

Raw file	Scan	Method	Score	m/z	Gene names
HBT_20130916_BV2_IC3_02	28845	ITMS; CID	105.02	615.66	Fadd

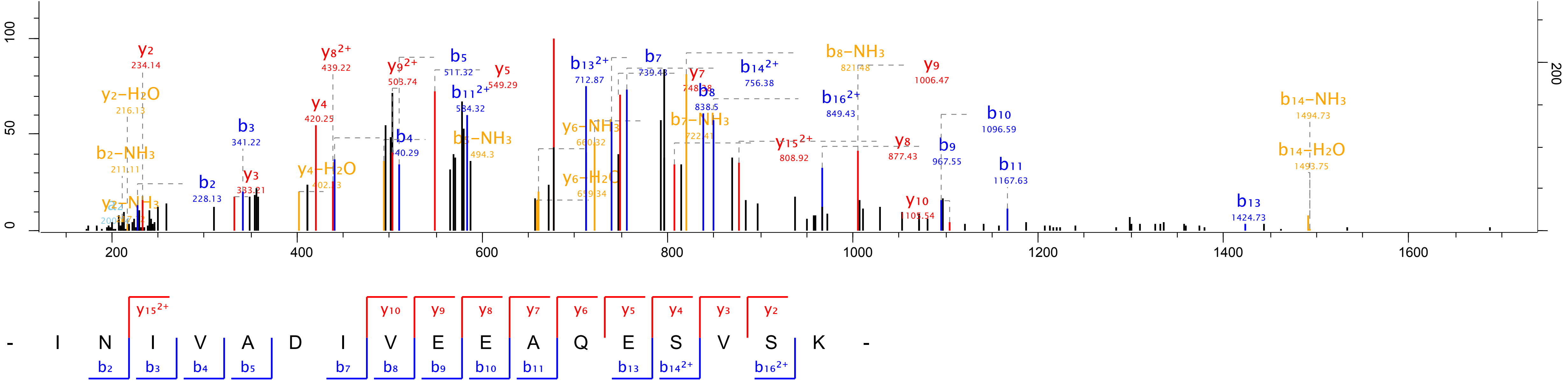

Raw file	Scan	Method	Score	m/z	Gene names
HBT_20130916_BV2_IC3_02	28792	ITMS; CID	81.36	989.03	Mdp1

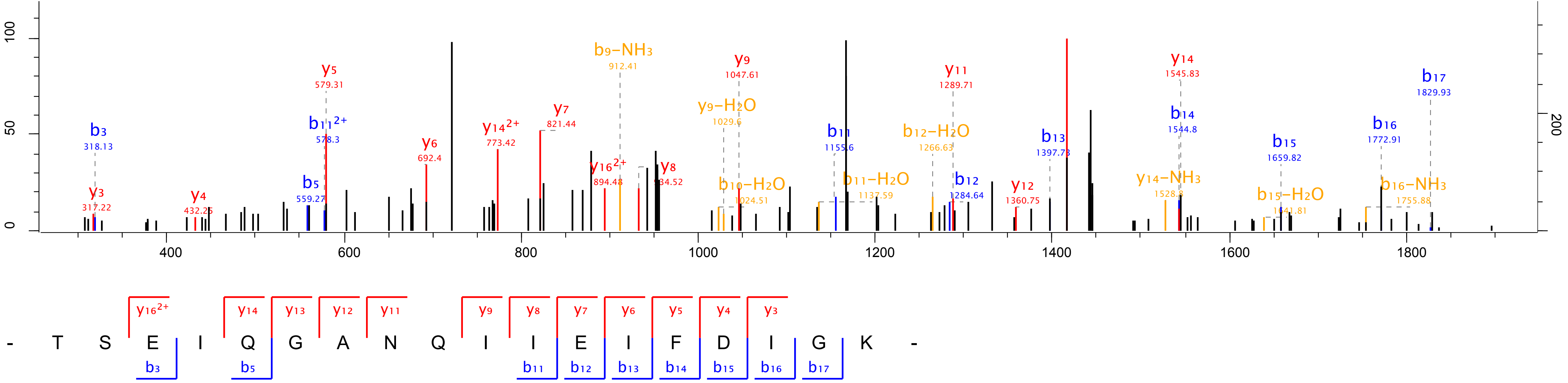

Raw file	Scan	Method	Score	m/z	Gene names
HBT_20130916_BV2_IC3_02	27196	ITMS; CID	84.38	1025.55	Fmn1

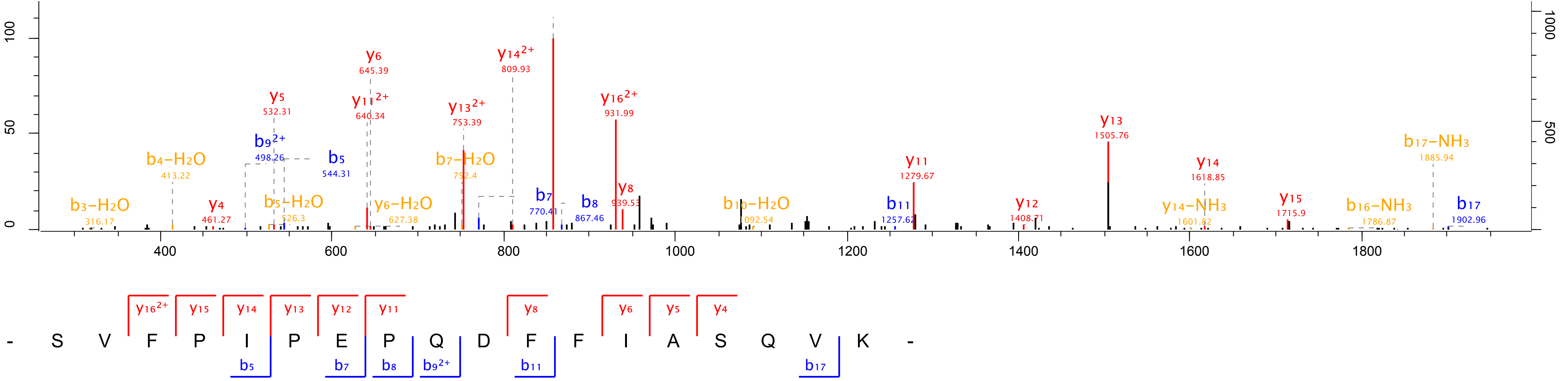

Raw file	Scan	Method	Score	m/z	Gene names
HBT_20130916_BV2_IC3_02	23412	ITMS; CID	100.93	715.9	Uqcr11

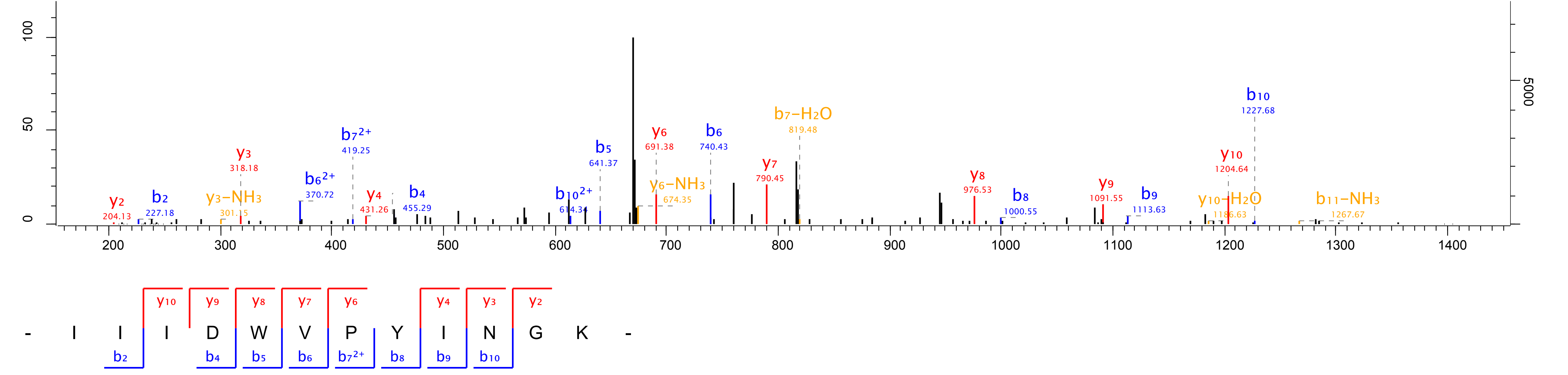

Raw file	Scan	Method	Score	m/z	Gene names
HBT_20130916_BV2_IC3_02	19361	ITMS; CID	158.56	594.81	Fam134b

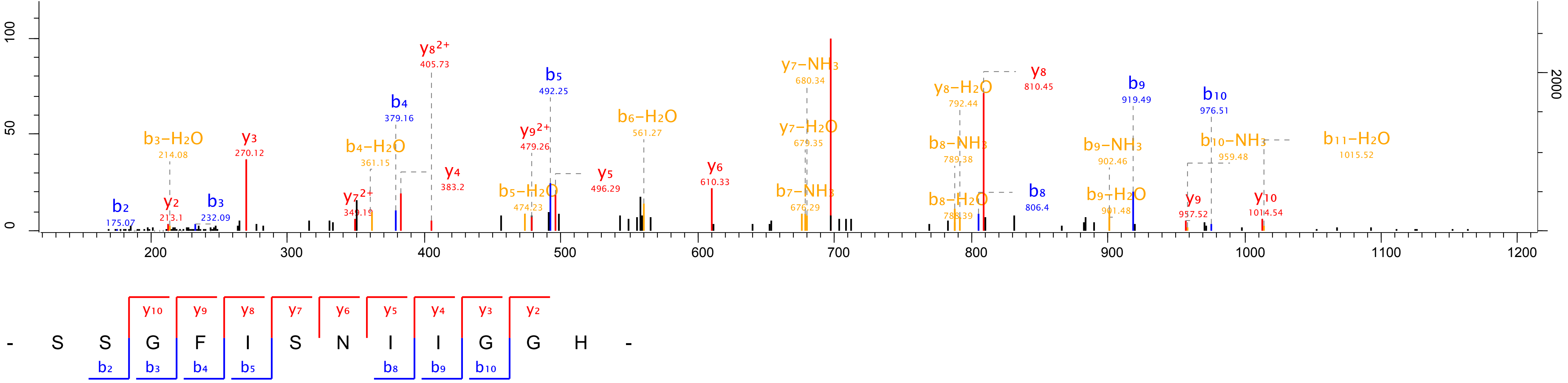

Raw file	Scan	Method	Score	m/z	Gene names
HBT_20130916_BV2_IC3_02	18374	ITMS; CID	120.4	1139.59	Rsrc2

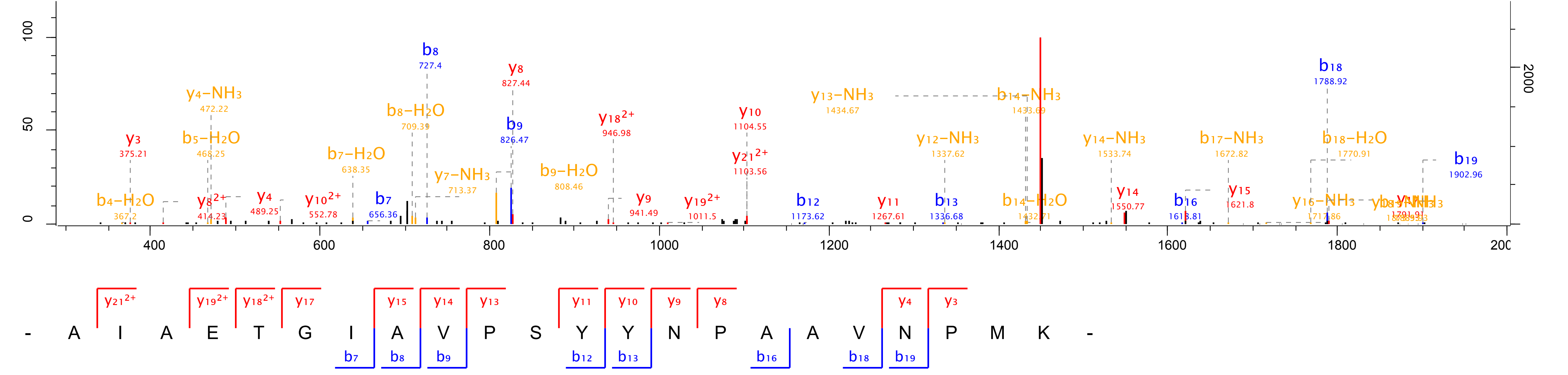

Raw file	Scan	Method	Score	m/z	Gene names
HBT_20130916_BV2_IC3_02	14359	ITMS; CID	122.94	558.29	Ndufs7

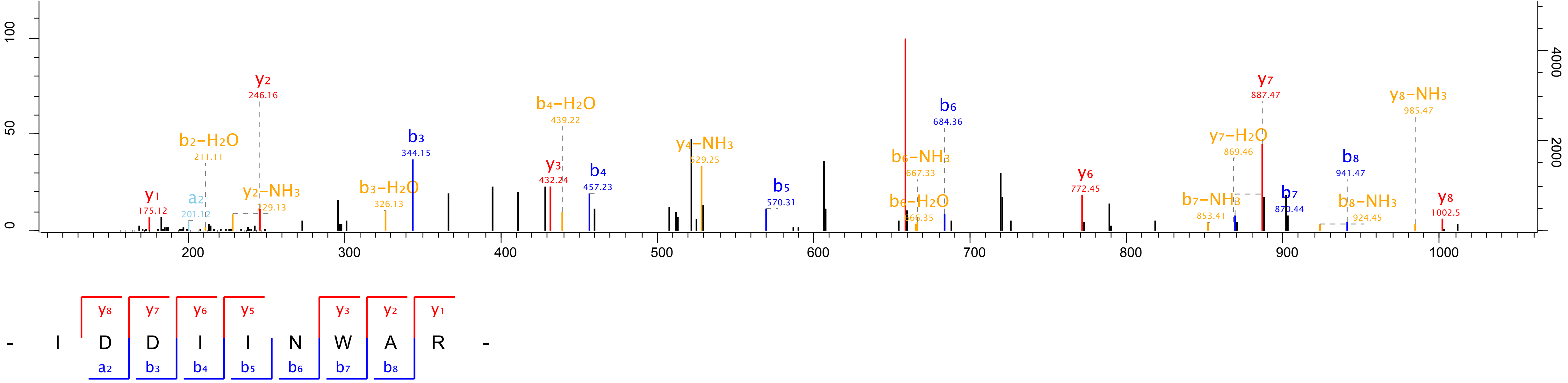

Raw file	Scan	Method	Score	m/z	Gene names
HBT_20130916_BV2_IC3_02	1406	ITMS; CID	121.29	599.78	Dctn5

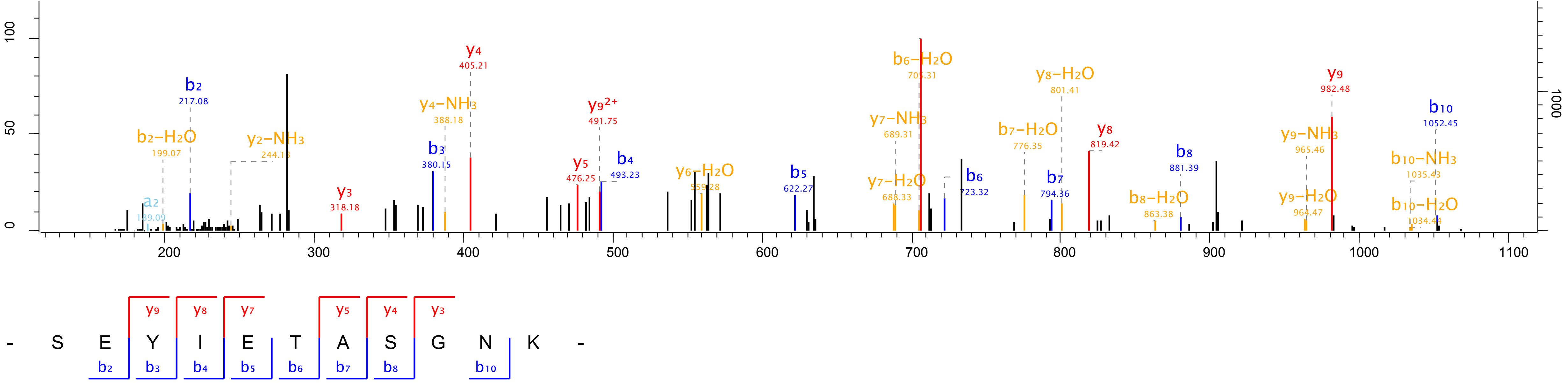

Raw file	Scan	Method	Score	m/z	Gene names
HBT_20130916_BV2_IC3_02	12687	ITMS; CID	119.88	784.37	Timm10

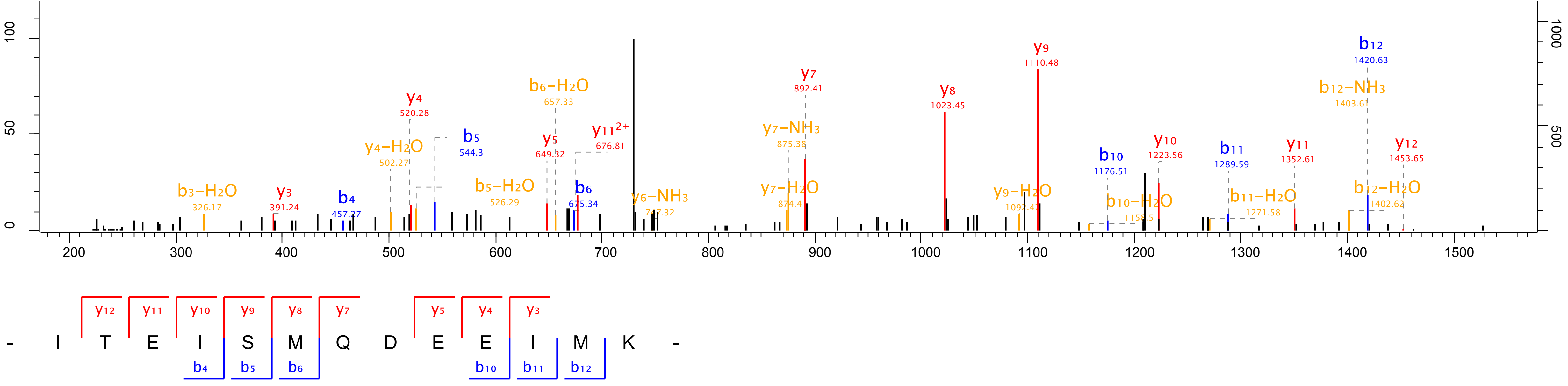

Raw file	Scan	Method	Score	m/z	Gene names
HBT_20130916_BV2_IC3_02	1148	ITMS; CID	152.04	538.77	Med22

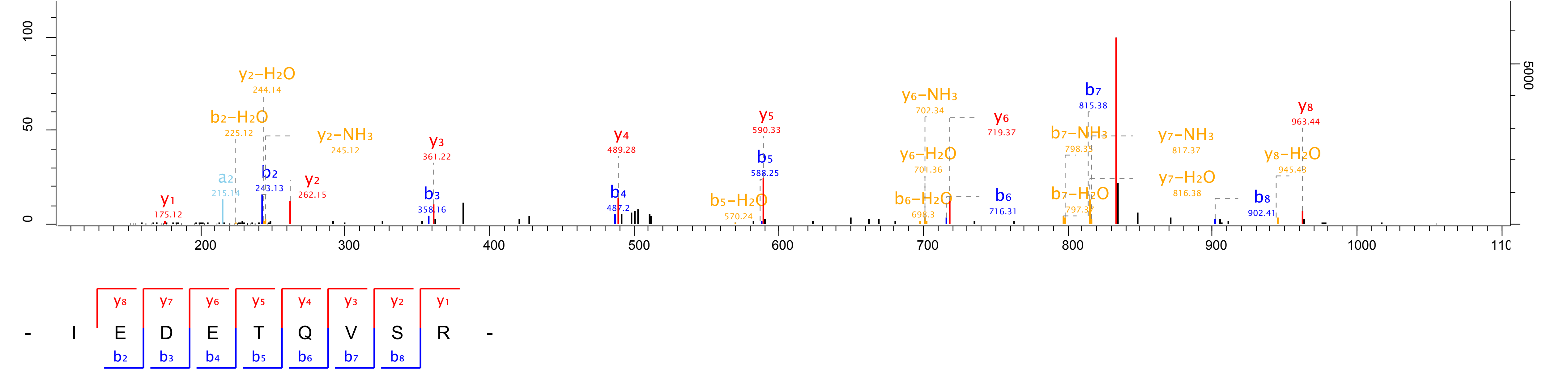

Raw file	Scan	Method	Score	m/z	Gene names
HBT_20130916_BV2_IC3_02	1129	ITMS; CID	167.97	895.36	Bub1

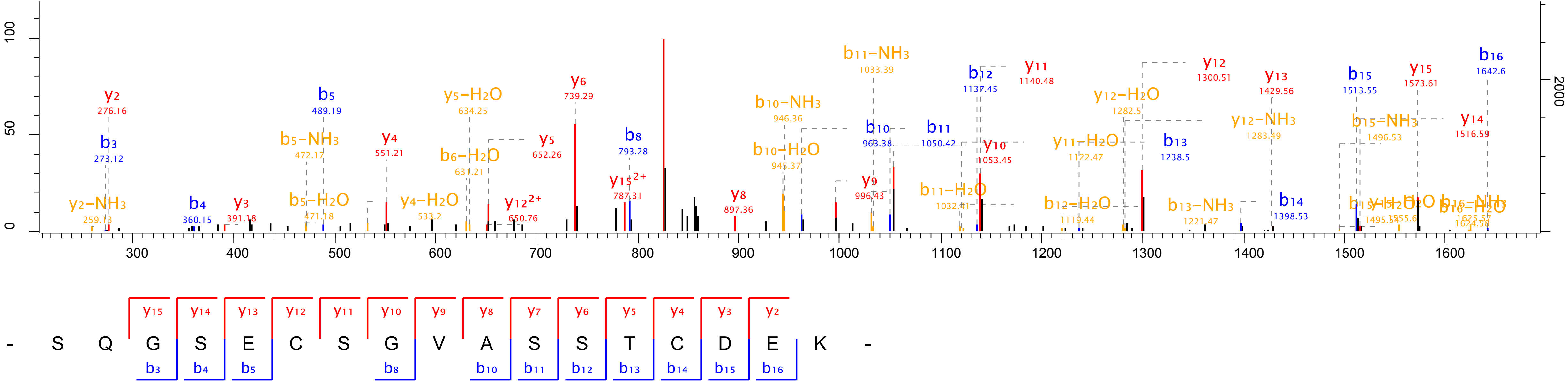

Raw file	Scan	Method	Score	m/z	Gene names
HBT_20130916_BV2_IC3_02	10274	ITMS; CID	74.81	880.48	Parp10

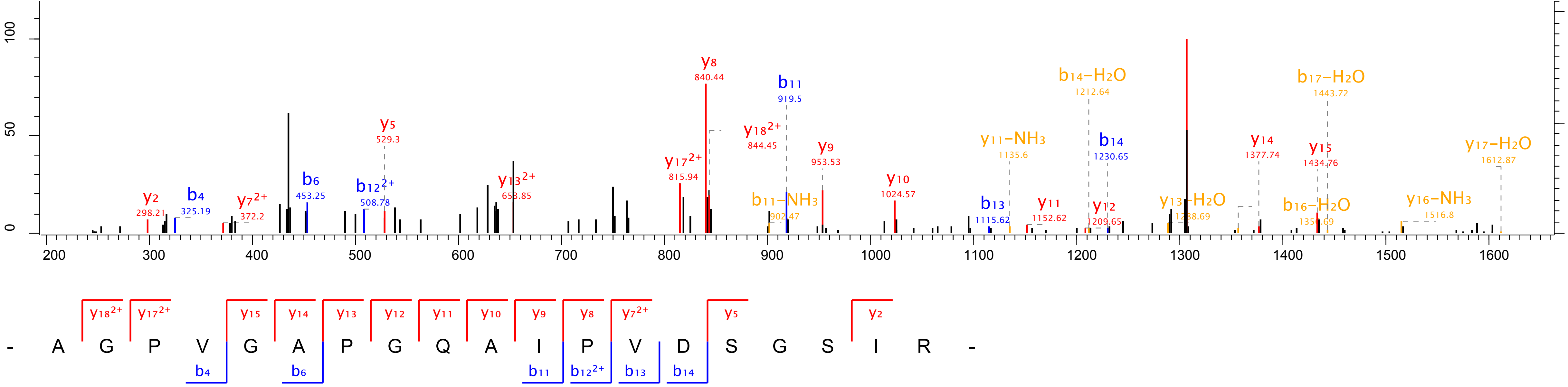

Raw file	Scan	Method	Score	m/z
HBT_20130916_BV2_IC3_01	5021	ITMS; CID	101.9	549.29

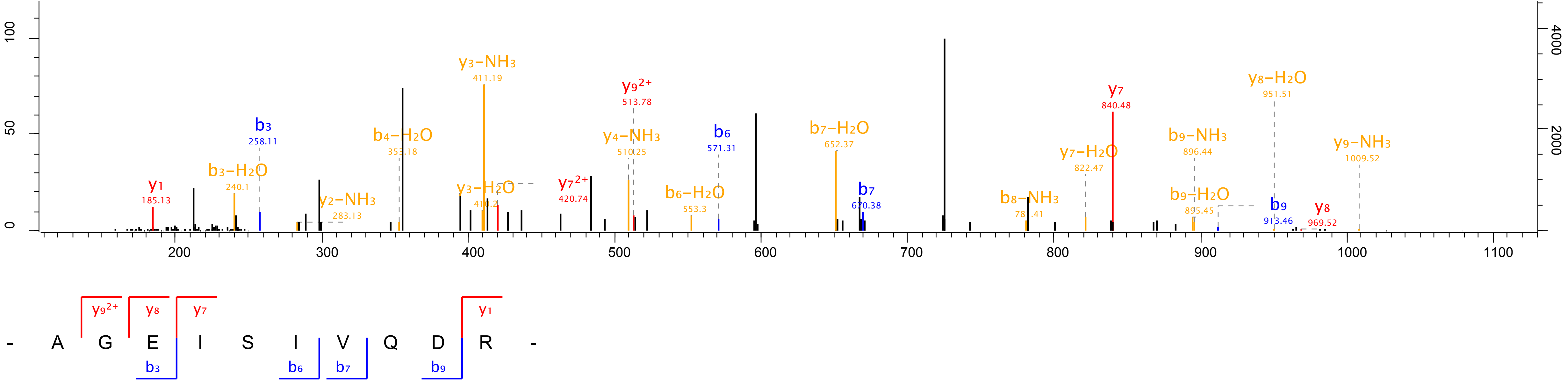

Raw file	Scan	Method	Score	m/z	Gene names
HBT_20130916_BV2_IC3_01	4102	ITMS; CID	101.05	732.35	Fxyd2

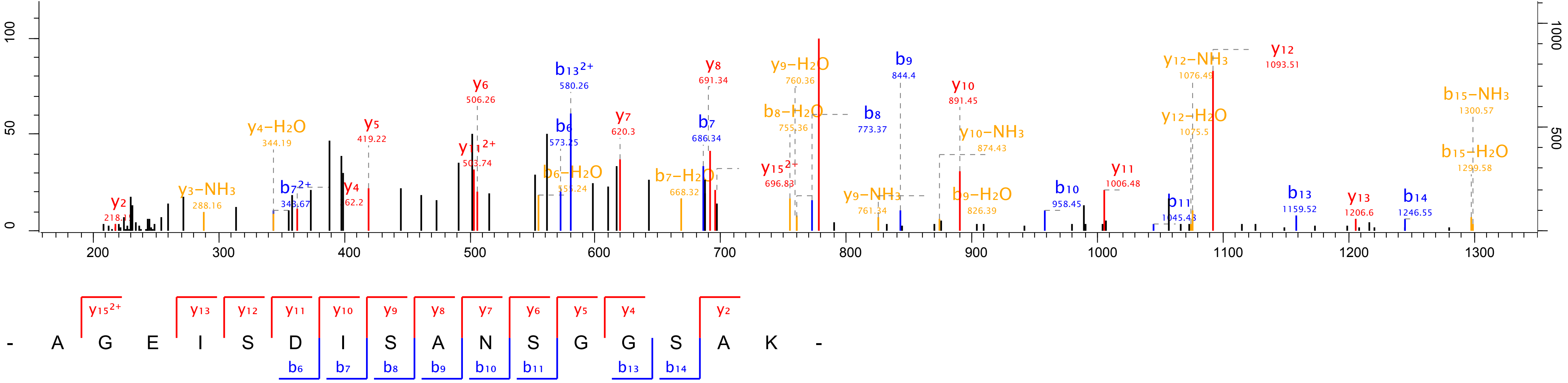

Raw file	Scan	Method	Score	m/z
HBT_20130916_BV2_IC3_01	31793	ITMS; CID	197.29	869.46

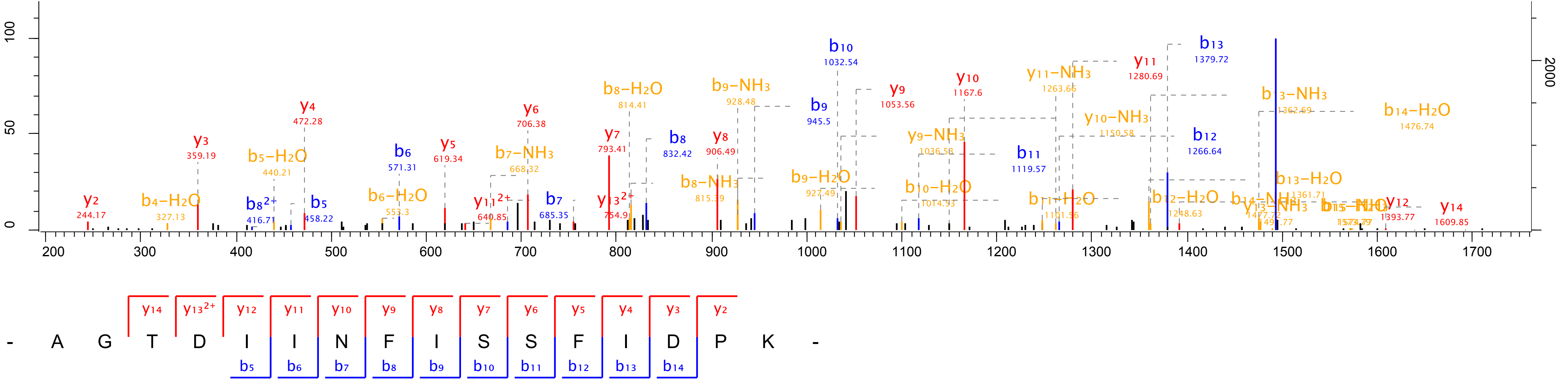

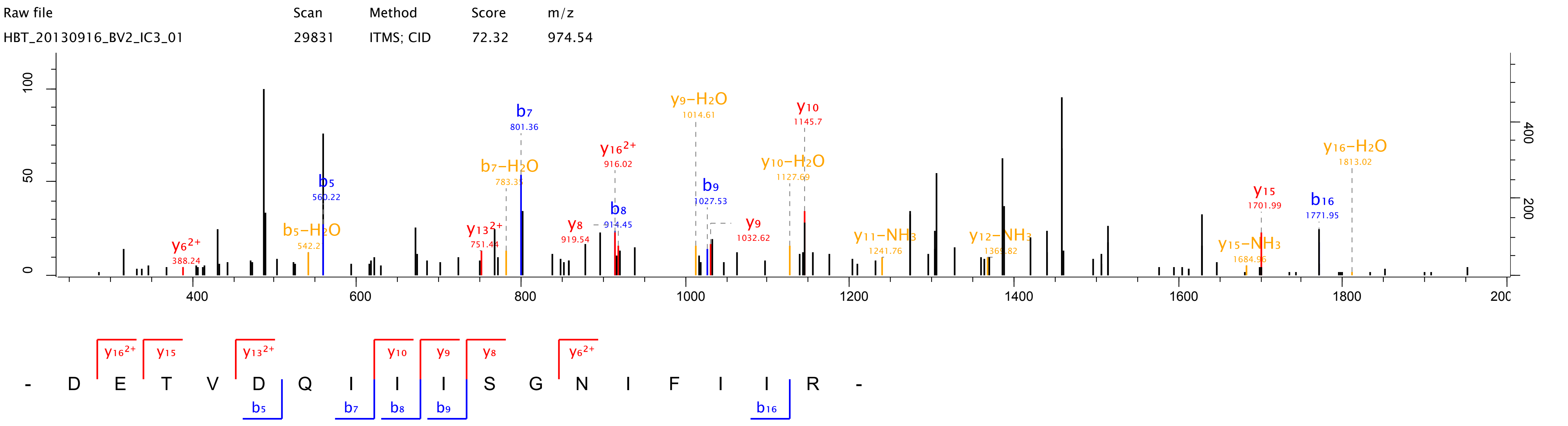

Raw file
HBT_20130916_BV2_IC3_01
Scan
29831
Method
ITMS; CID
Score
72.32
m/z
974.54
y6 2+ 388.24
b5-H2O 542.2
b5 560.22
y13 2+ 751.44
b7-H2O 783.35
b7 801.36
y8 919.54
y16 2+ 916.02
b8 914.45
y9-H2O 1014.61
b9 1027.53
y9 1032.62
y10-H2O 1127.69
y10 1145.7
y11-NH3 1241.76
y12-NH3 1369.82
y15-NH3 1684.96
y15 1701.99
b16 1771.95
y16-H2O 1813.02
- D E T V D Q I I I S G N I F I I R -

Raw file	Scan	Method	Score	m/z	Gene names
HBT_20130916_BV2_IC3_01	29365	ITMS; CID	75.76	912.44	Allc

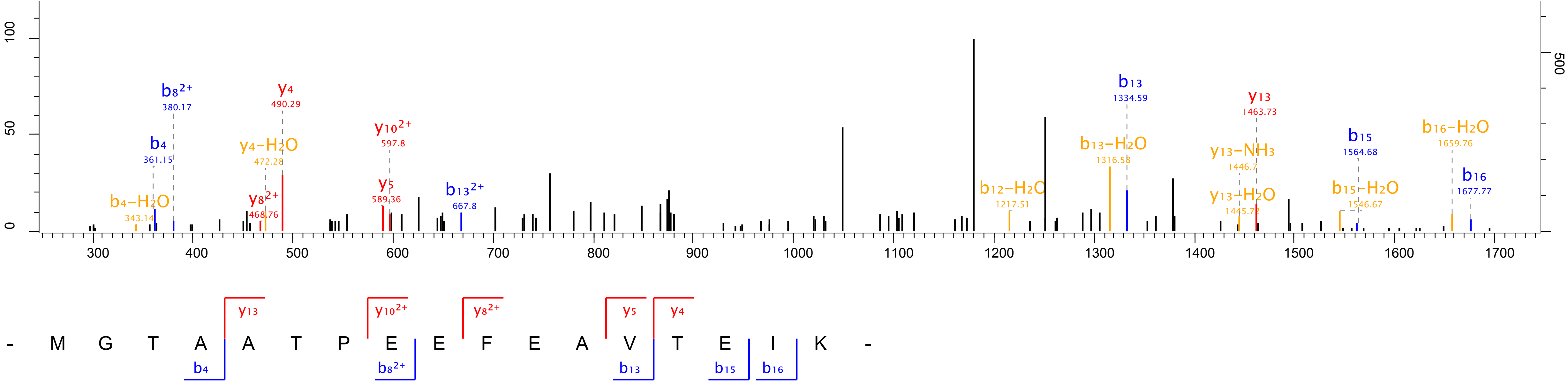

Raw file	Scan	Method	Score	m/z	Gene names
HBT_20130916_BV2_IC3_01	22120	ITMS; CID	108.96	1028.52	Dcun1d1

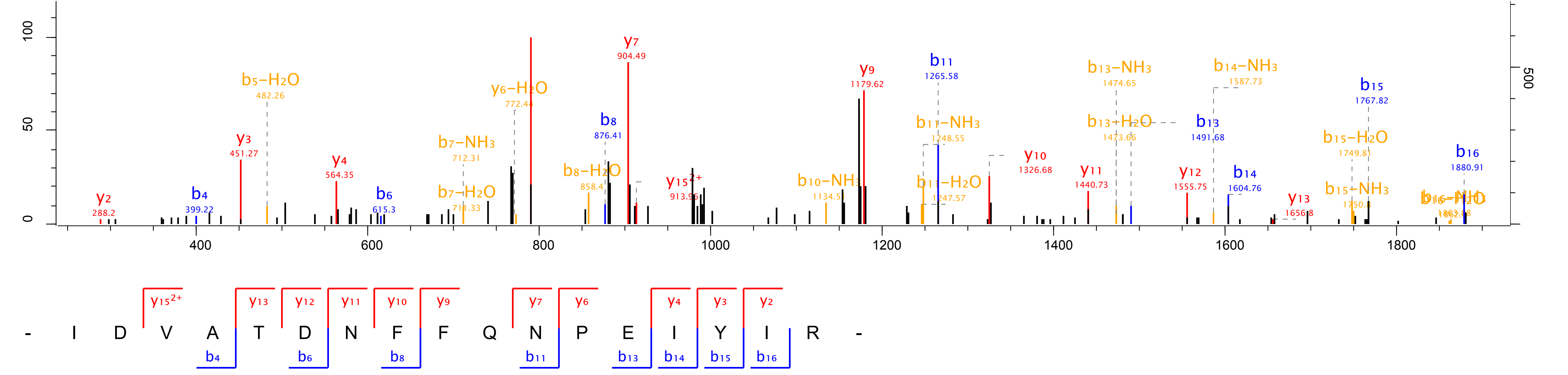

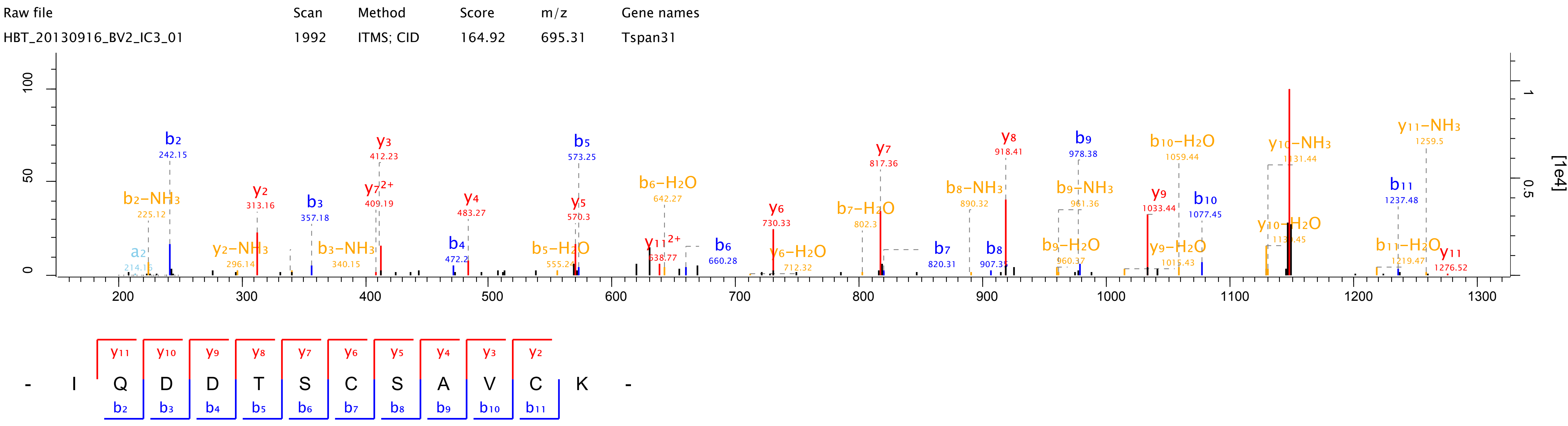

Raw file
HBT_20130916_BV2_IC3_01
Scan
1992
Method
ITMS; CID
Score
164.92
m/z
695.31
Gene names
Tspan31
b2
242.15
b2-NH3
225.12
y2
313.16
b3
357.18
y3
412.23
y7 2+
409.19
y4
483.27
b4
472.2
b5
573.25
y5
570.3
b5-H2O
555.24
b6-H2O
642.27
y11 2+
638.77
b6
660.28
y6
730.33
y6-H2O
712.32
y7
817.36
b7-H2O
802.3
b7
820.31
b8-NH3
890.32
y8
918.41
b8
907.35
b9
978.38
b9-NH3
961.36
b9-H2O
960.37
y9
1033.44
b10-H2O
1059.44
y9-H2O
1015.43
b10
1077.45
y10-NH3
1131.44
y10-H2O
b11
1237.48
y11-NH3
1259.5
b11-H2O
1219.47
y11
1276.52
y2-NH3
296.14
b3-NH3
340.15
a2
100
50
0
200
300
400
500
600
700
800
900
1000
1100
1200
1300
1
0.5
[1e4]
y11 y10 y9 y8 y7 y6 y5 y4 y3 y2
- I Q D D T S C S A V C K -
b2 b3 b4 b5 b6 b7 b8 b9 b10 b11

Raw file	Scan	Method	Score	m/z
HBT_20130916_BV2_IC3_01	19784	ITMS; CID	70.12	928.47

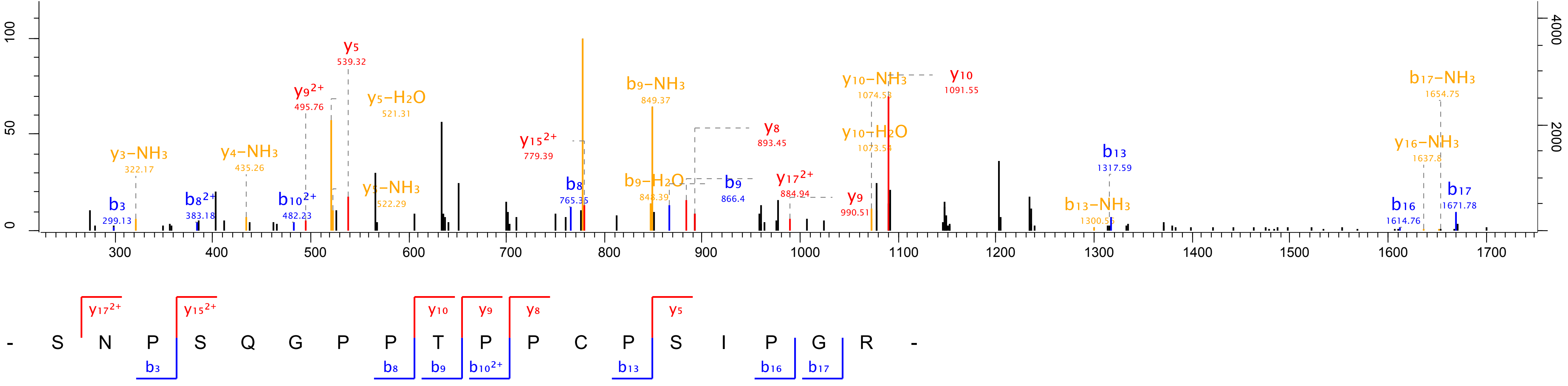

Raw file	Scan	Method	Score	m/z	Gene names
HBT_20130916_BV2_IC3_01	1809	ITMS; CID	135.86	685.31	Tbc1d8b

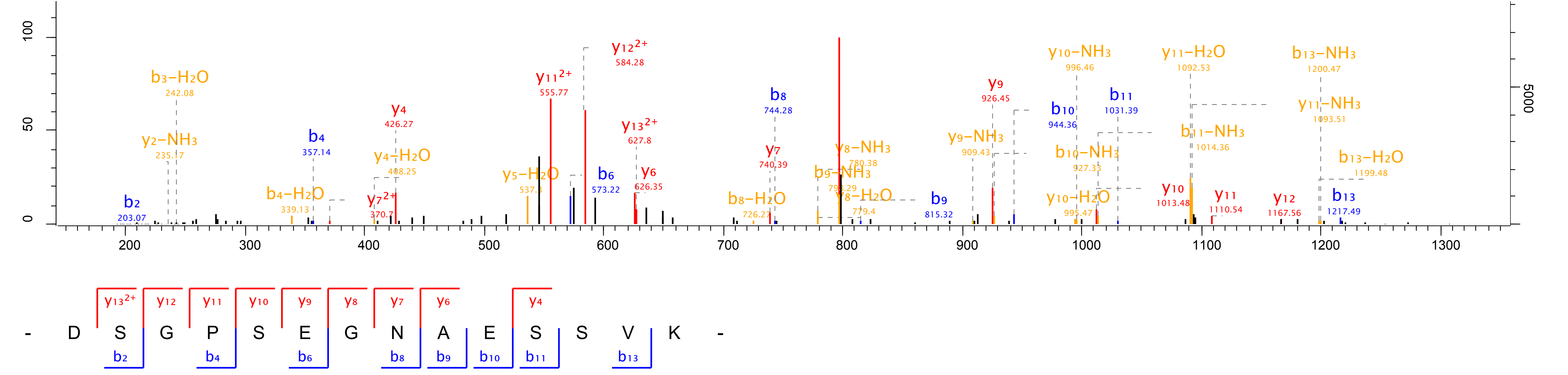

Raw file	Scan	Method	Score	m/z	Gene names
HBT_20130916_BV2_IC3_01	17597	ITMS; CID	82.76	1165.59	Lrmp

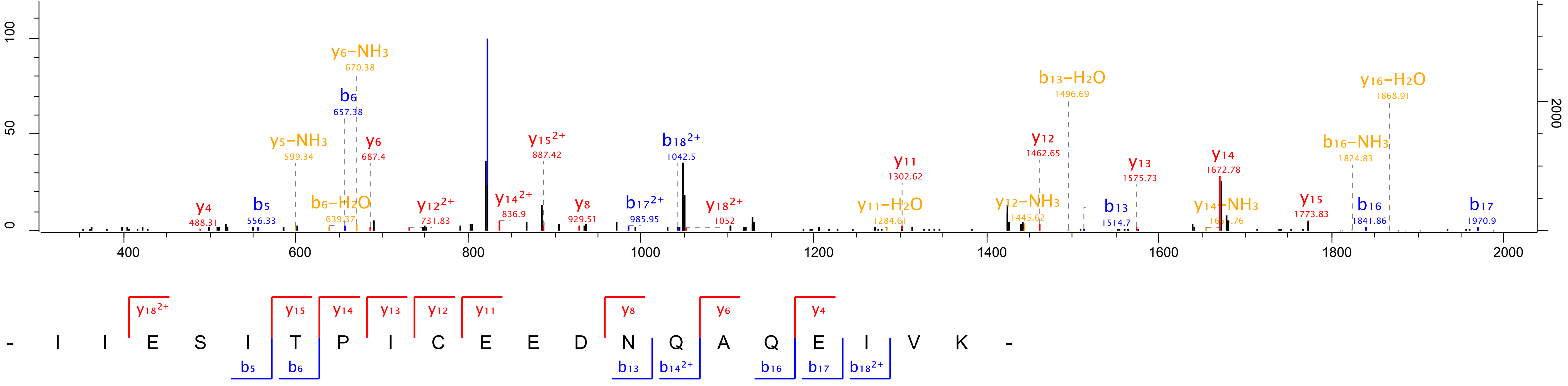

Raw file	Scan	Method	Score	m/z	Gene names
HBT_20130916_BV2_IC3_01	1582	ITMS; CID	200	798.35	Snapc1

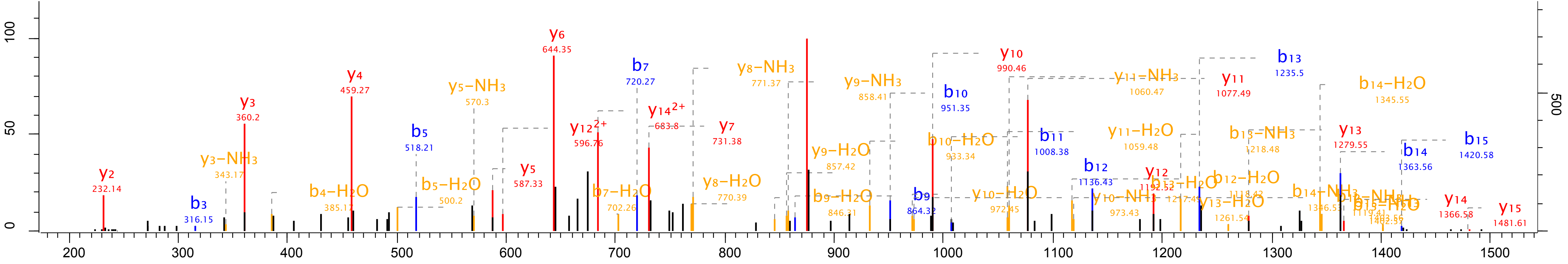

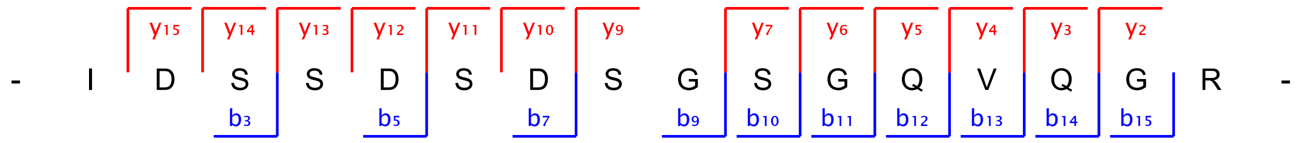

Raw file	Scan	Method	Score	m/z	Gene names
HBT_20130916_BV2_IC3_01	1567	ITMS; CID	153.75	861.87	Zbtb1

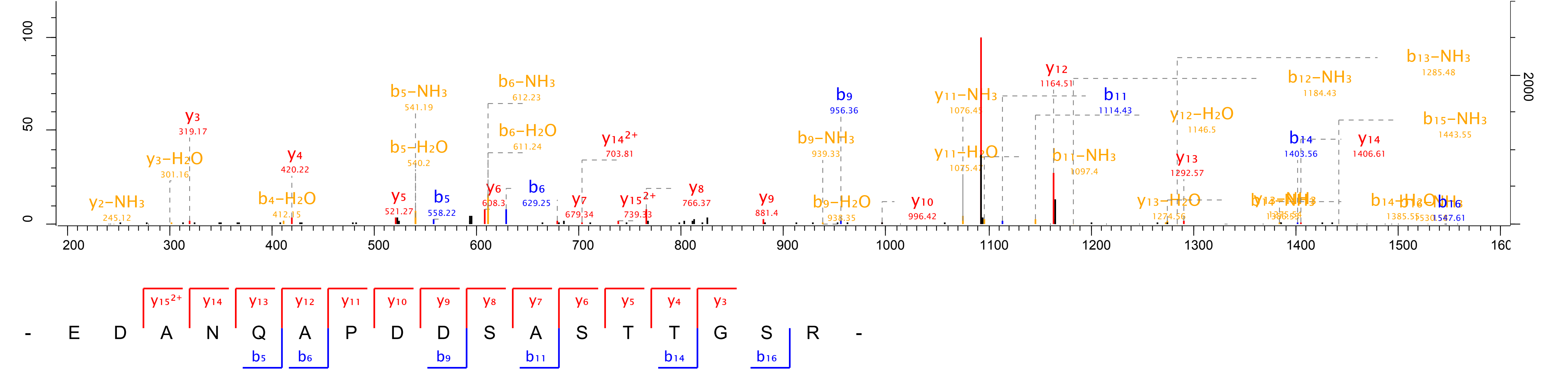

Raw file	Scan	Method	Score	m/z	Gene names
HBT_20130916_BV2_IC3_01	13710	ITMS; CID	141.13	1079.47	Cmtm3

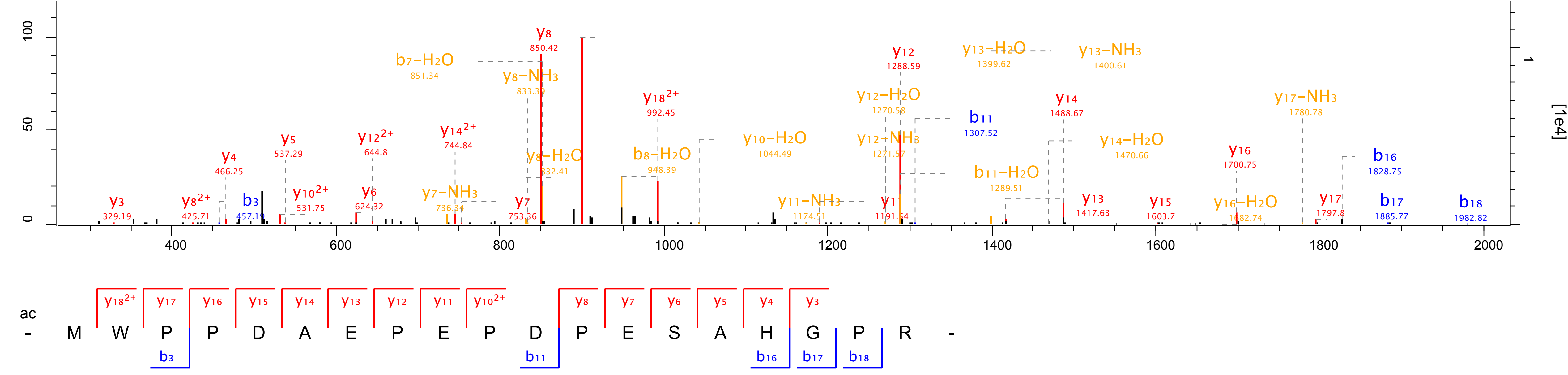

Raw file	Scan	Method	Score	m/z	Gene names
HBT_20130916_BV2_IC3_01	12984	ITMS; CID	145.7	783.36	Tmsb10

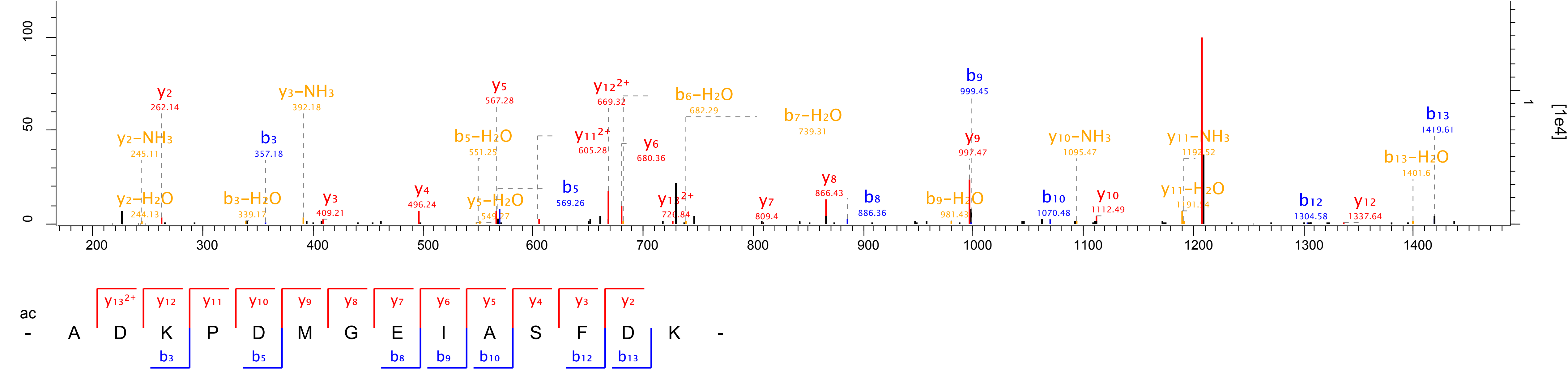

Raw file	Scan	Method	Score	m/z	Gene names
HBT_20130916_BV2_IC3_01	12694	ITMS; CID	147.96	652.35	Tmem109

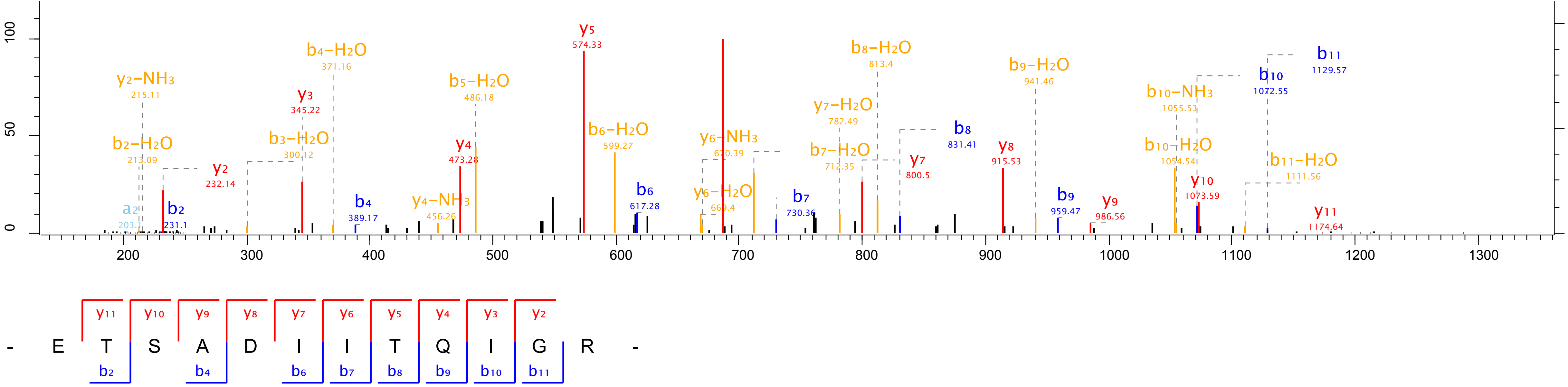

Raw file	Scan	Method	Score	m/z	Gene names
HBT_20130916_BV2_IC3_01	11728	ITMS; CID	66.02	987.47	St7l

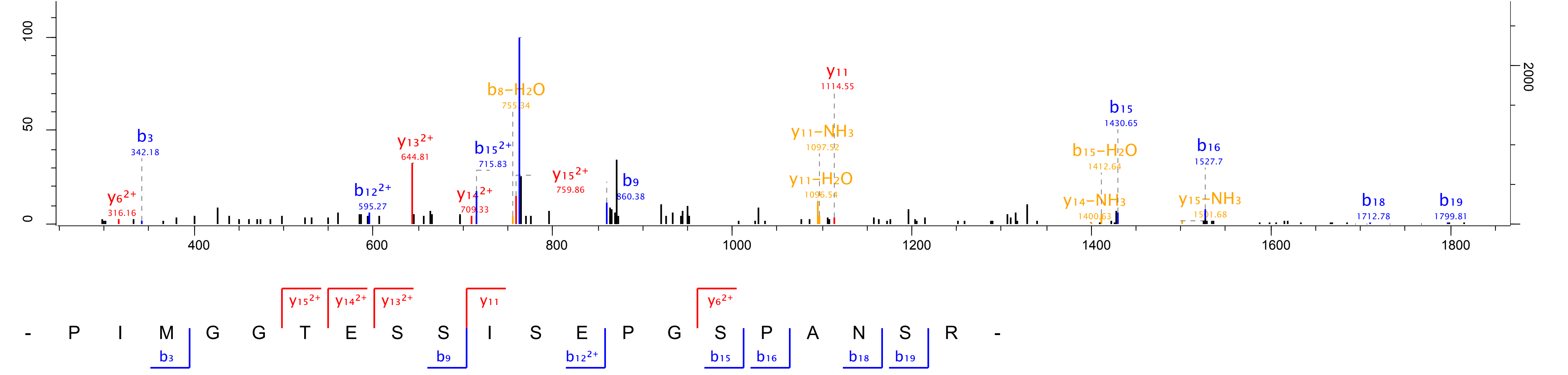

Raw file	Scan	Method	Score	m/z	Gene names
HBT_20130916_BV2_IC3_01	11180	ITMS; CID	174.21	774.85	1810009A15Rik

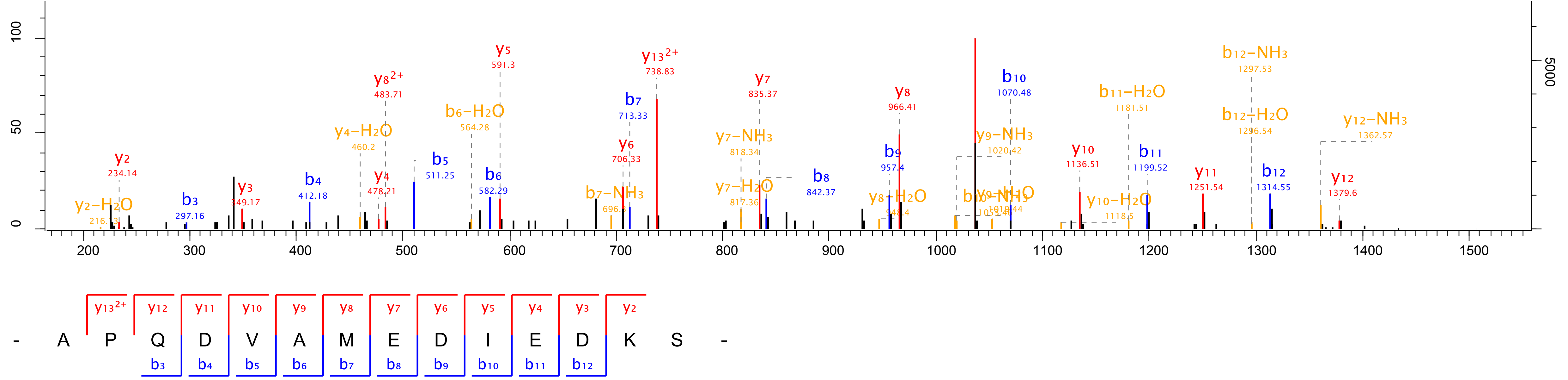

Raw file	Scan	Method	Score	m/z	Gene names
HBT_20130916_BV2_IC3_01	10217	ITMS; CID	85.05	966.48	Timm10b

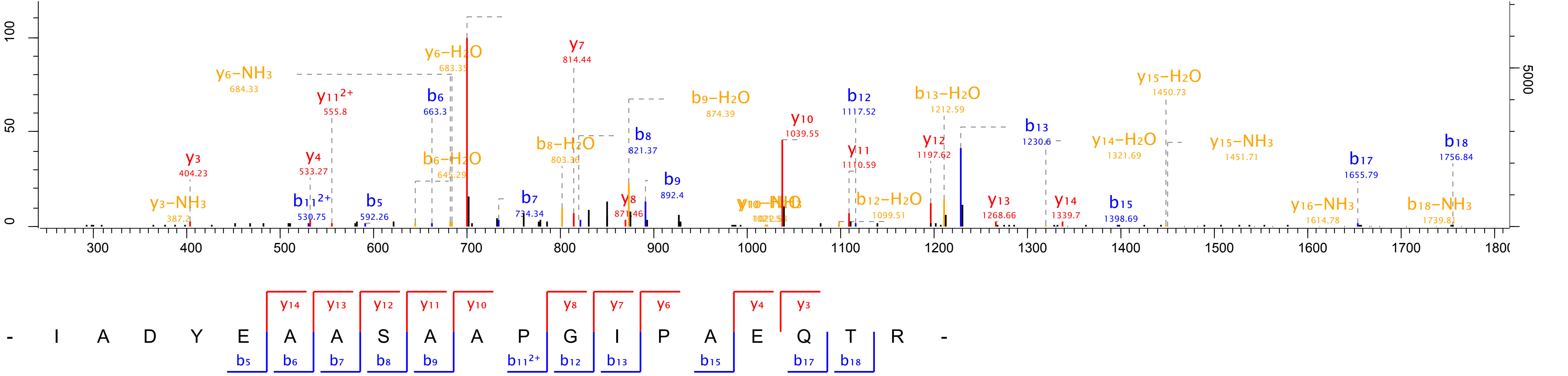

Raw file	Scan	Method	Score	m/z
HBT_20130916_BV2_IC2_06	9739	ITMS; CID	75.65	425.26

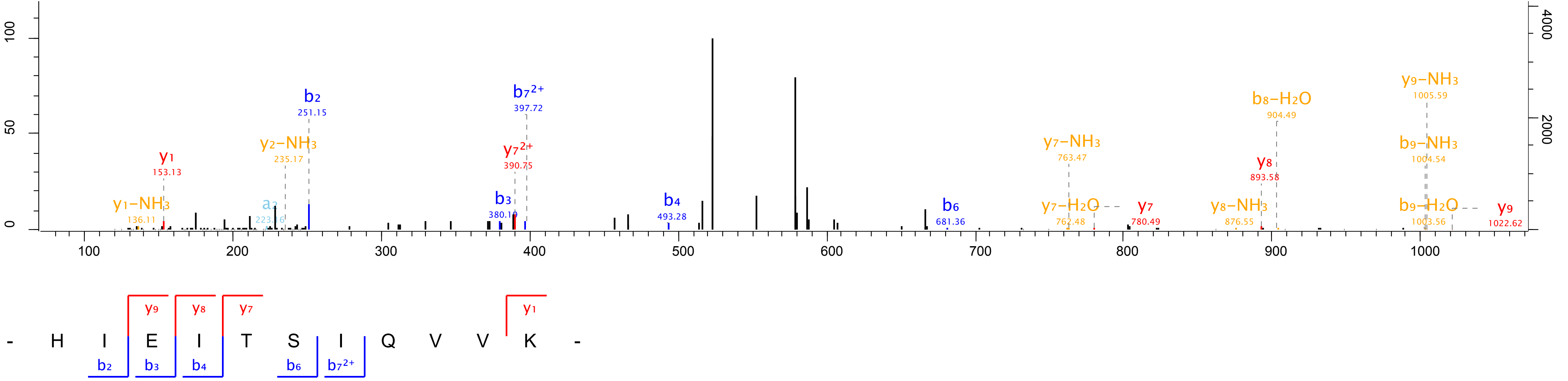

Raw file	Scan	Method	Score	m/z	Gene names
HBT_20130916_BV2_IC2_06	9024	ITMS; CID	67.33	779.39	Gm12253

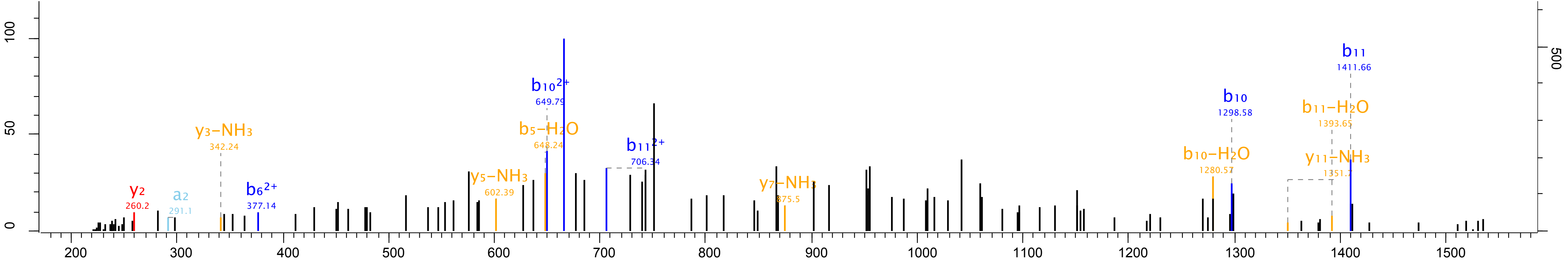

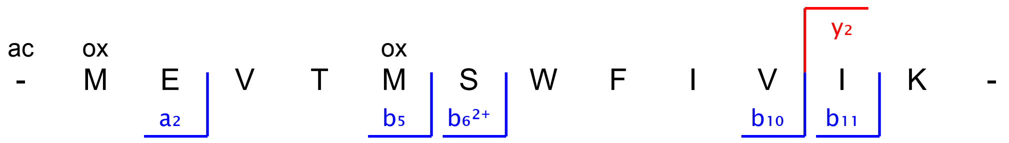

Raw file	Scan	Method	Score	m/z	Gene names
HBT_20130916_BV2_IC2_06	1152	ITMS; CID	123.96	629.35	Lsm14a

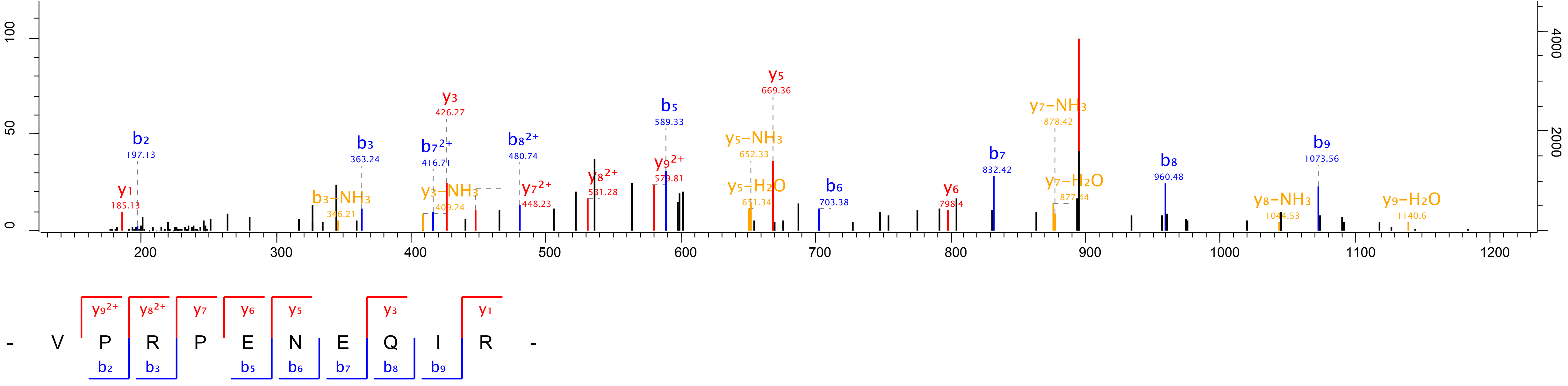

Raw file	Scan	Method	Score	m/z	Gene names
HBT_20130916_BV2_IC2_05	794	ITMS; CID	62.24	556.6	Cbx2

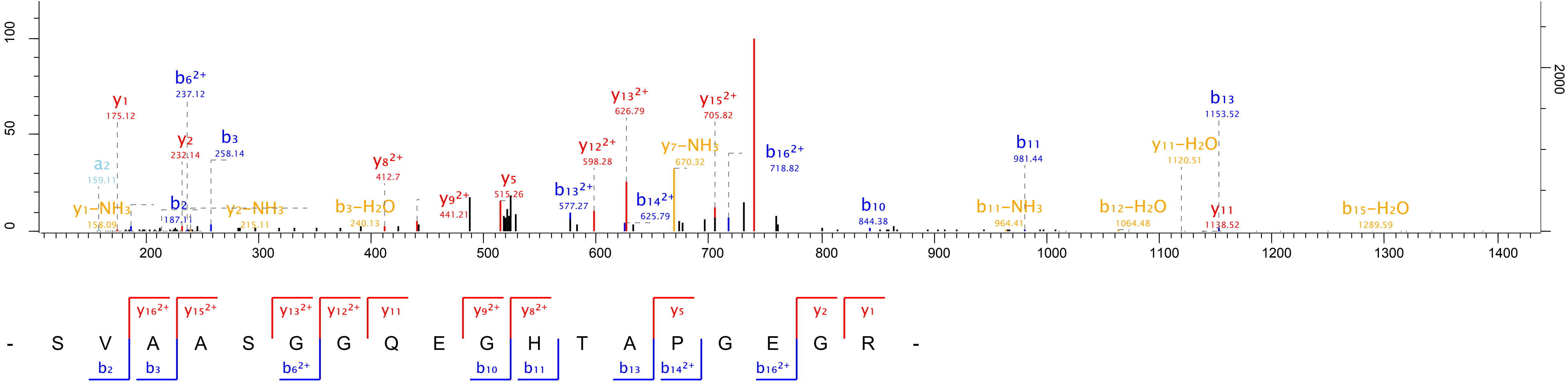

Raw file	Scan	Method	Score	m/z	Gene names
HBT_20130916_BV2_IC2_05	7749	ITMS; CID	120.63	864.44	Chchd1

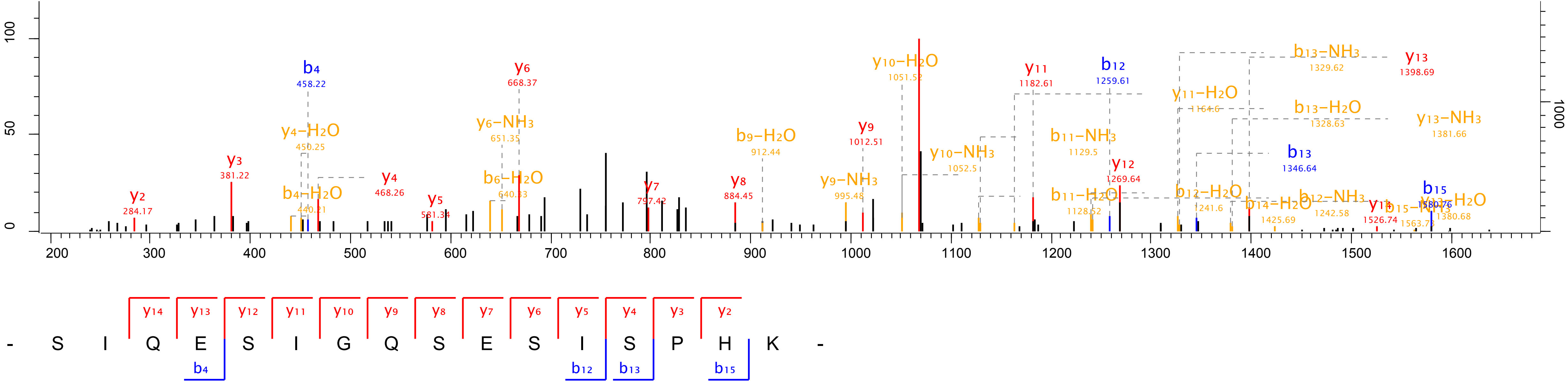

Raw file	Scan	Method	Score	m/z	Gene names
HBT_20130916_BV2_IC2_05	28722	ITMS; CID	81.36	806.42	Selt

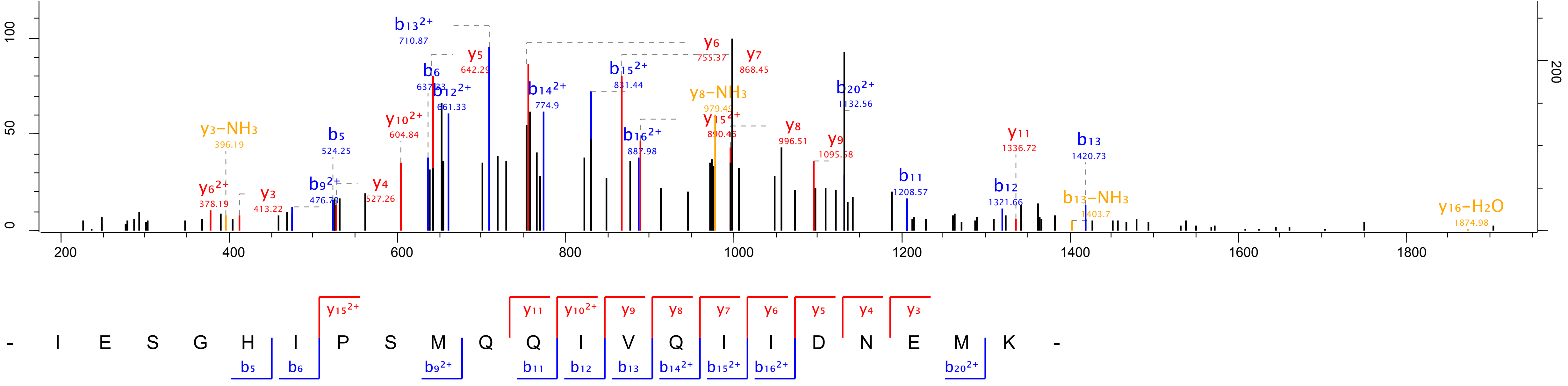

Raw file	Scan	Method	Score	m/z	Gene names
HBT_20130916_BV2_IC2_05	23546	ITMS; CID	71.29	698.04	Ccdc90b

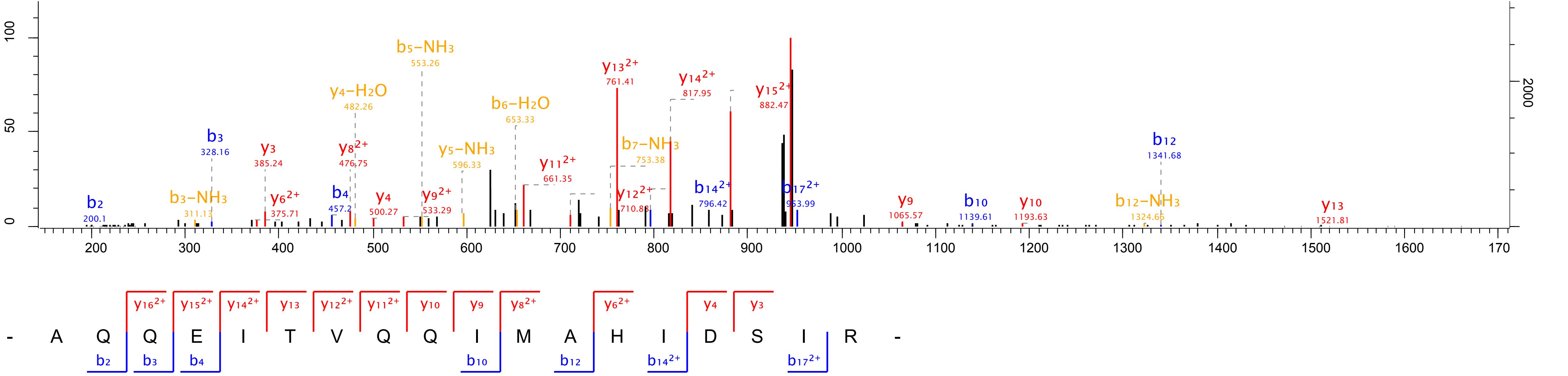

Raw file	Scan	Method	Score	m/z	Gene names
HBT_20130916_BV2_IC2_05	16894	ITMS; CID	65.22	578.99	Mblac1

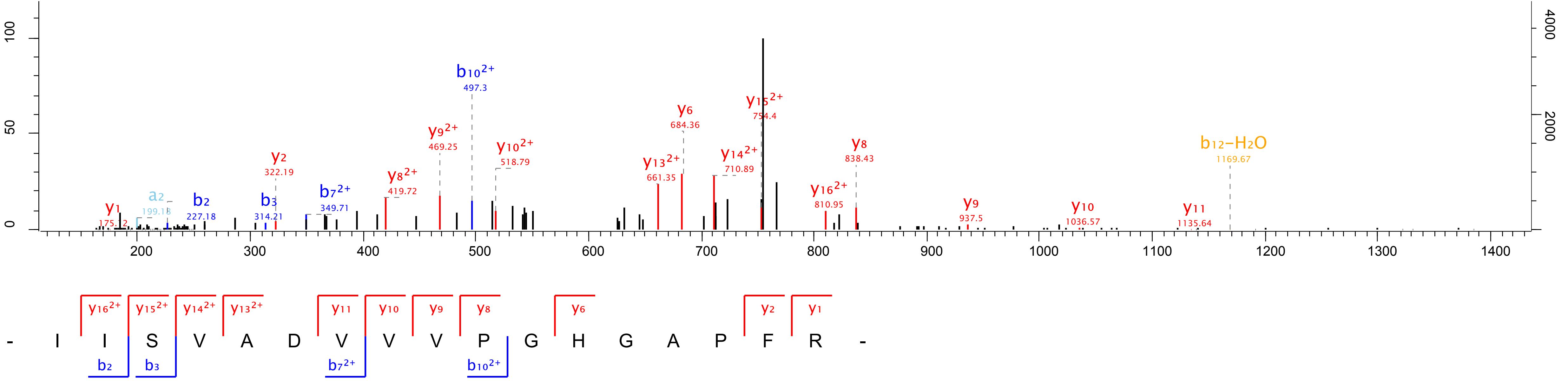

Raw file	Scan	Method	Score	m/z	Gene names
HBT_20130916_BV2_IC2_05	14190	ITMS; CID	189.33	993.49	Llph

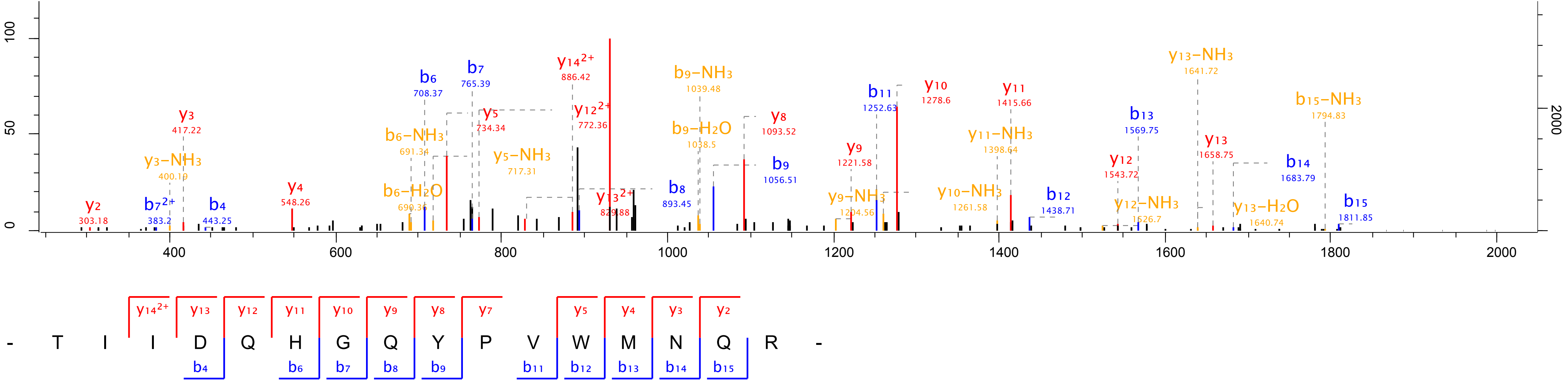

Raw file	Scan	Method	Score	m/z	Gene names
HBT_20130916_BV2_IC2_04	9575	ITMS; CID	98.55	933.47	Pdcd4

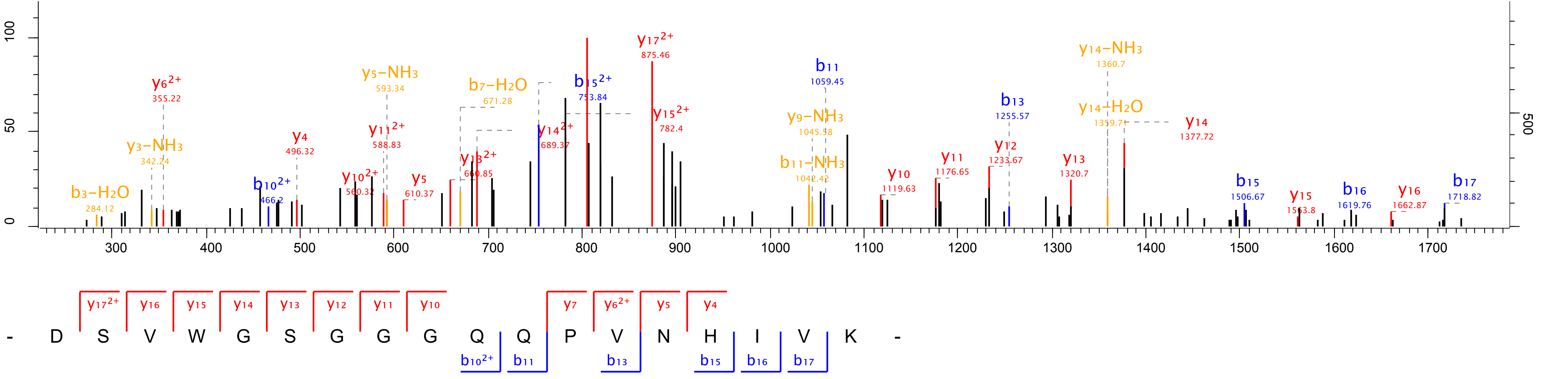

Raw file	Scan	Method	Score	m/z	Gene names
HBT_20130916_BV2_IC2_04	834	ITMS; CID	103.92	702.62	Fbxo28

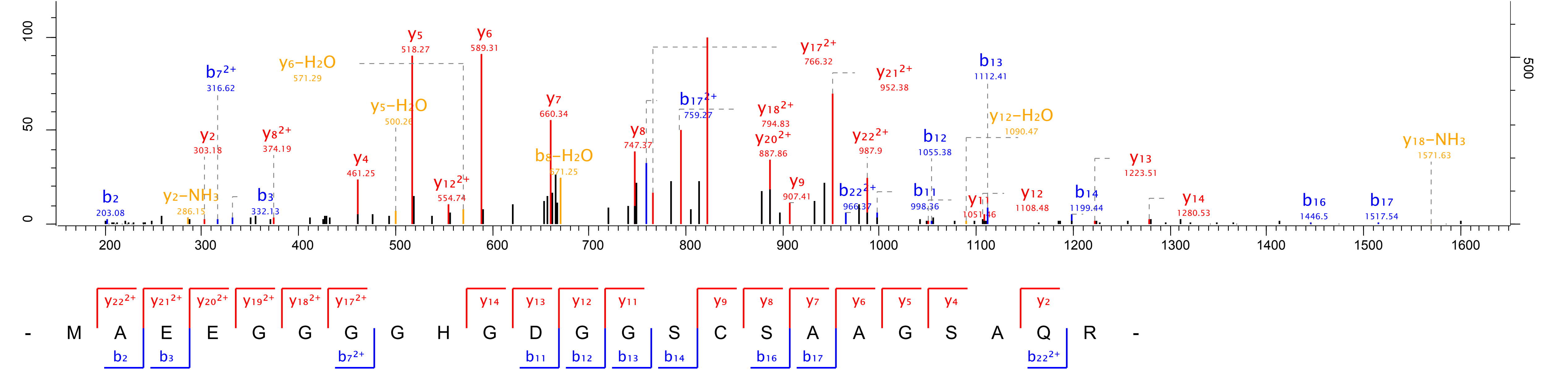

Raw file	Scan	Method	Score	m/z	Gene names
HBT_20130916_BV2_IC2_04	4632	ITMS; CID	174.24	870.93	Tmem181b-ps;Tmem181a

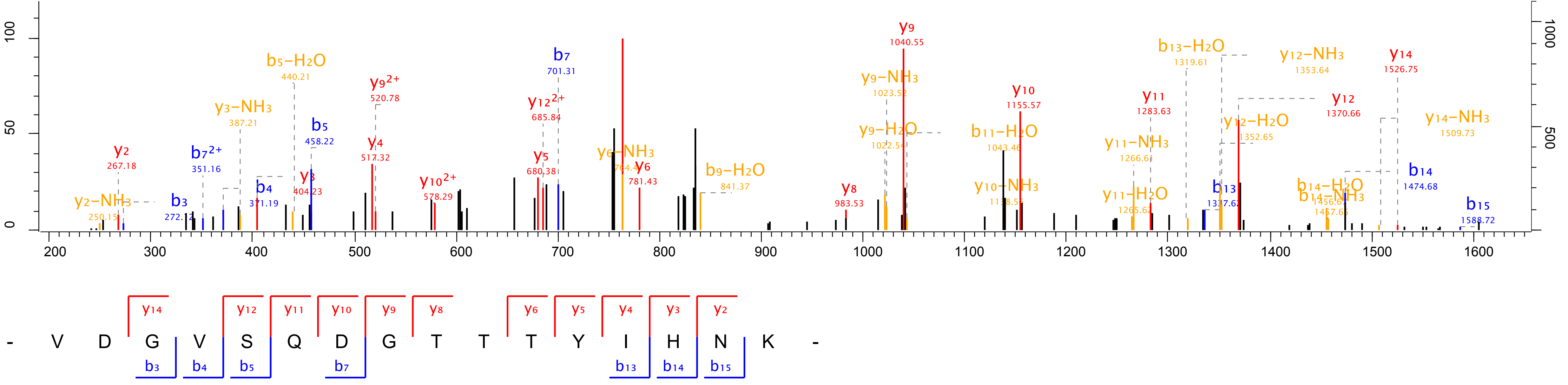

Raw file	Scan	Method	Score	m/z	Gene names
HBT_20130916_BV2_IC2_04	23372	ITMS; CID	111.55	1104.21	Cnih

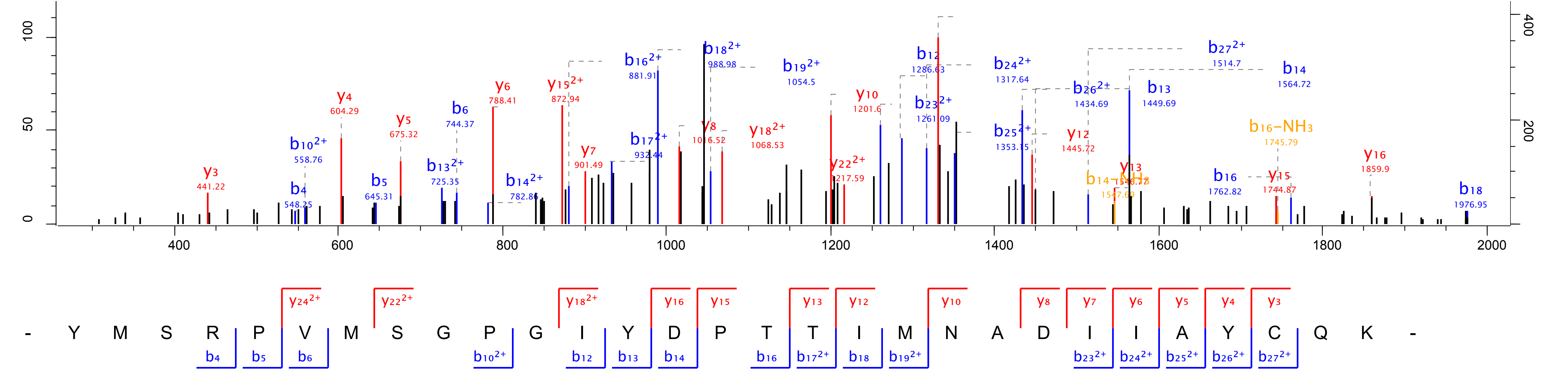

Raw file	Scan	Method	Score	m/z	Gene names
HBT_20130916_BV2_IC2_04	17211	ITMS; CID	143.99	1099.05	Lmbrd1

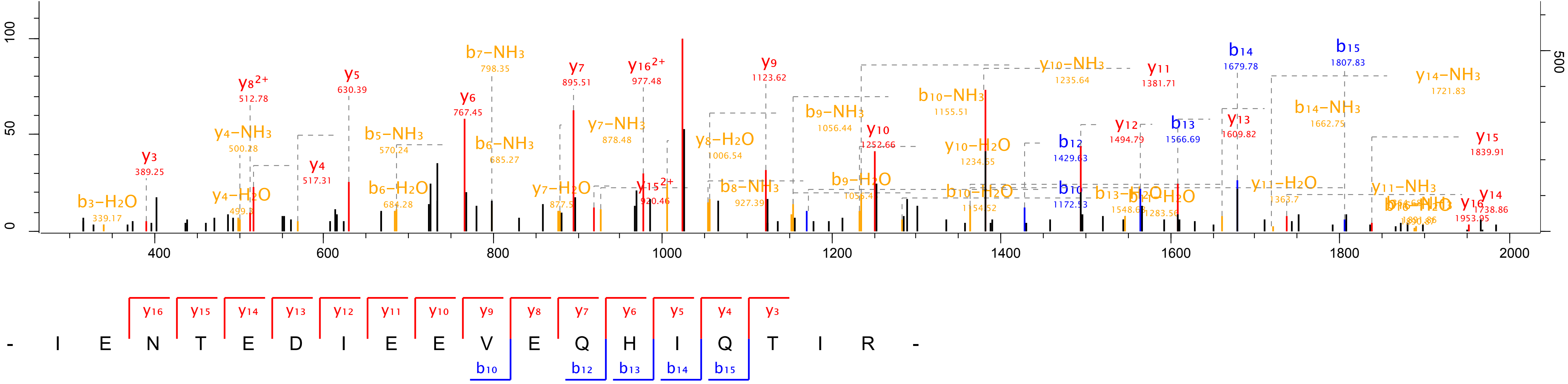

Raw file	Scan	Method	Score	m/z	Gene names
HBT_20130916_BV2_IC2_04	1617	ITMS; CID	147.33	647.3	Pop4

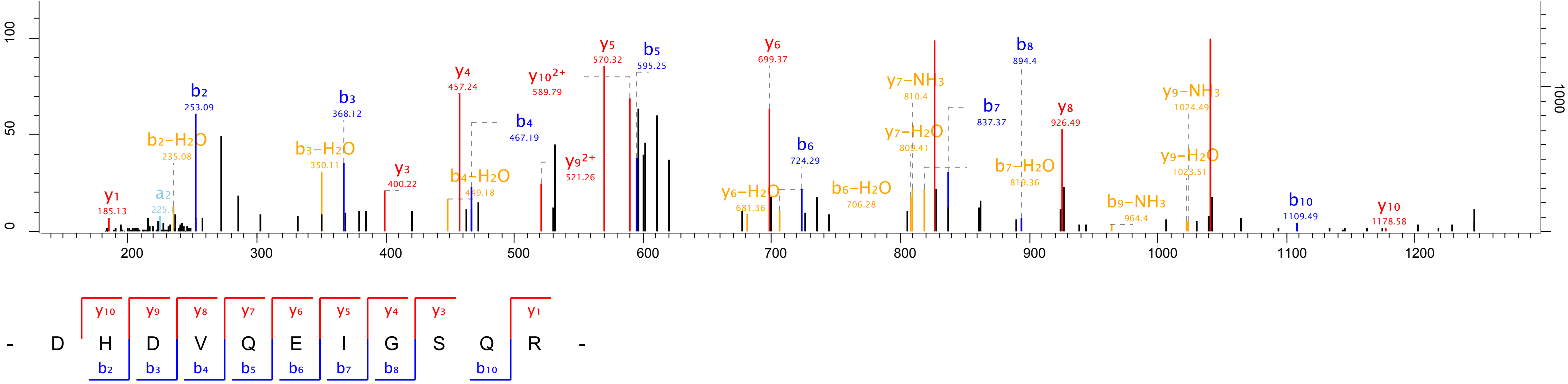

Raw file	Scan	Method	Score	m/z
HBT_20130916_BV2_IC2_04	11550	ITMS; CID	74.99	756.85

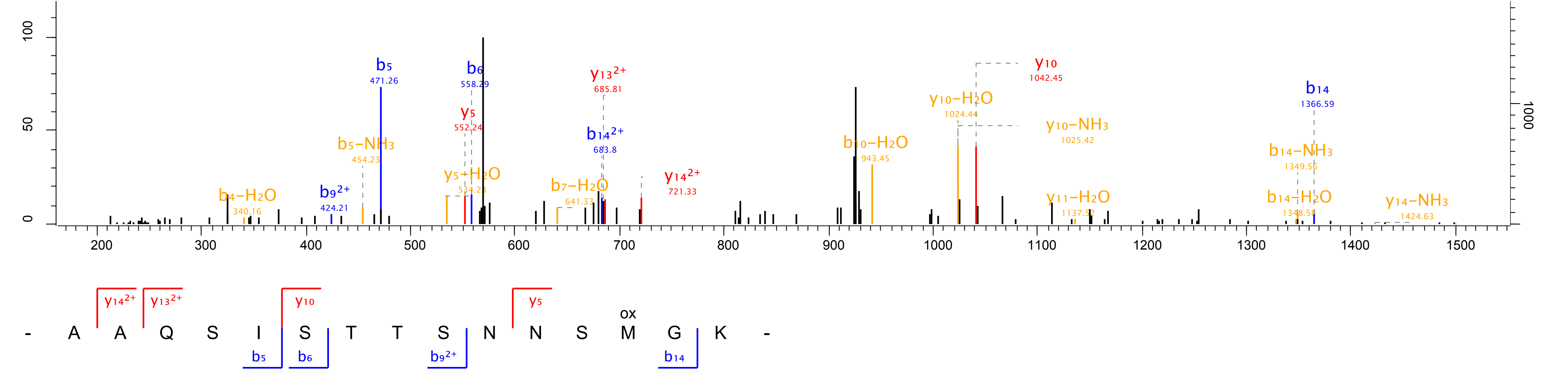

Raw file	Scan	Method	Score	m/z	Gene names
HBT_20130916_BV2_IC2_04	1111	ITMS; CID	131.83	643.82	Smim4

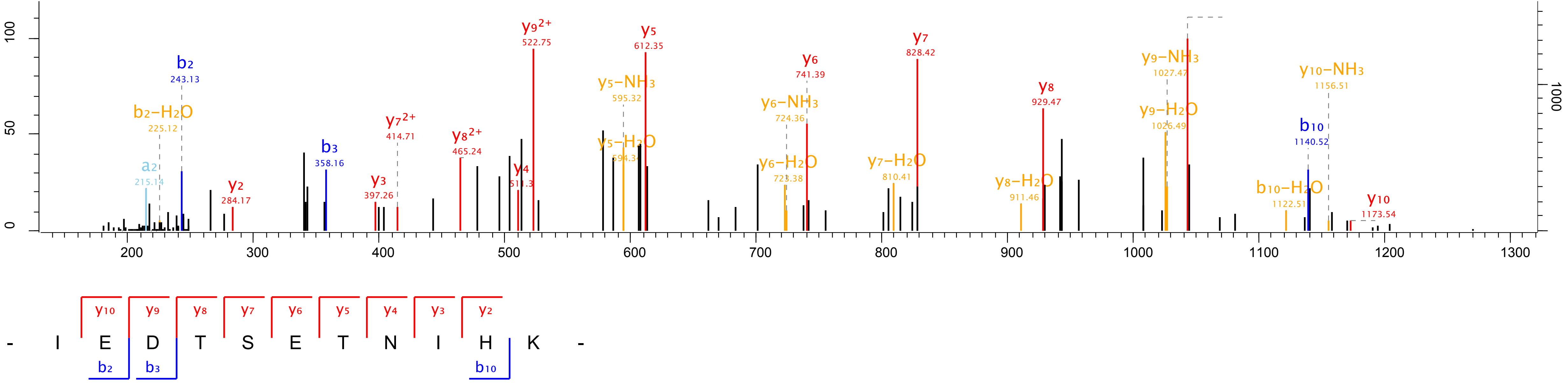

Raw file	Scan	Method	Score	m/z	Gene names
HBT_20130916_BV2_IC2_04	10118	ITMS; CID	81.13	645.68	Gm5283

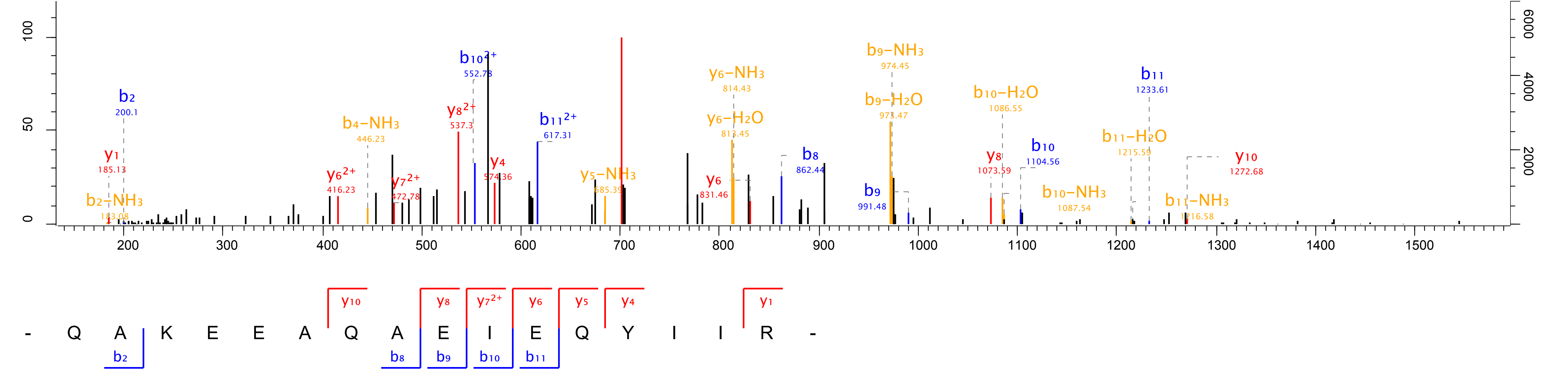

Raw file	Scan	Method	Score	m/z	Gene names
HBT_20130916_BV2_IC2_03	26048	ITMS; CID	105.2	863.48	Nrbf2

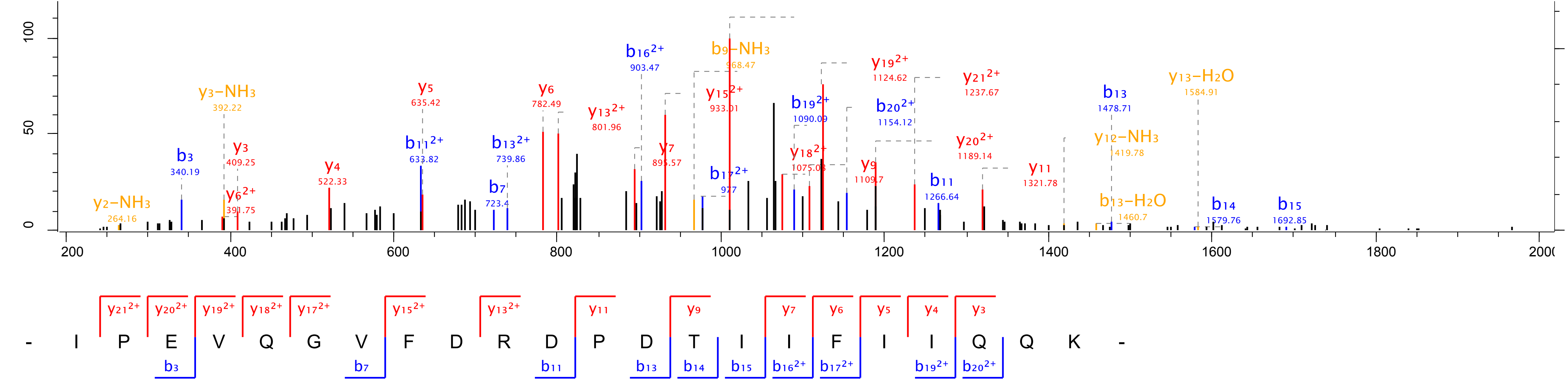

Raw file	Scan	Method	Score	m/z	Gene names
HBT_20130916_BV2_IC2_03	24976	ITMS; CID	83.86	622.35	Hmgxb4

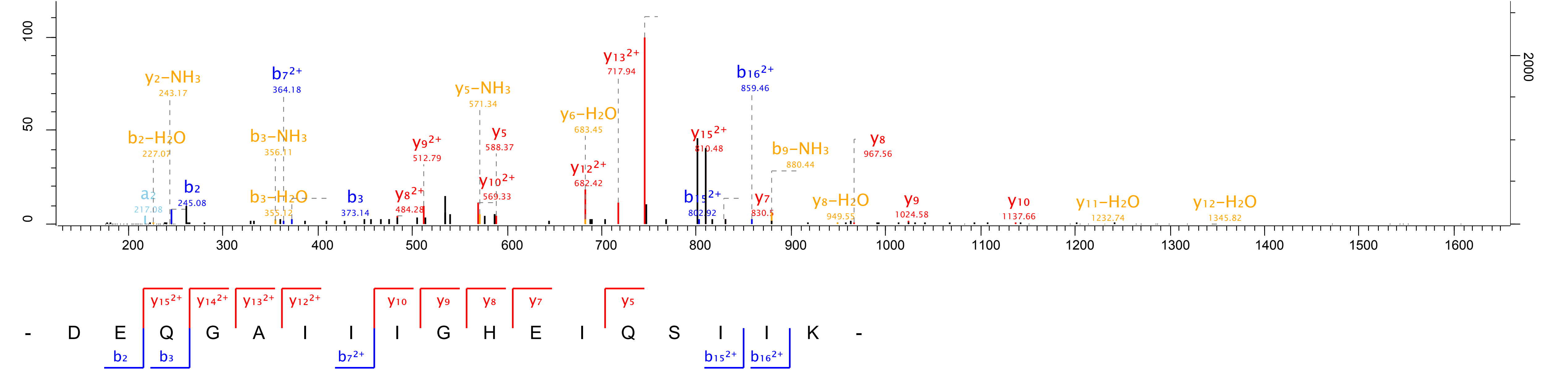

Raw file	Scan	Method	Score	m/z	Gene names
HBT_20130916_BV2_IC2_03	23989	ITMS; CID	47.75	1130.58	Tead4

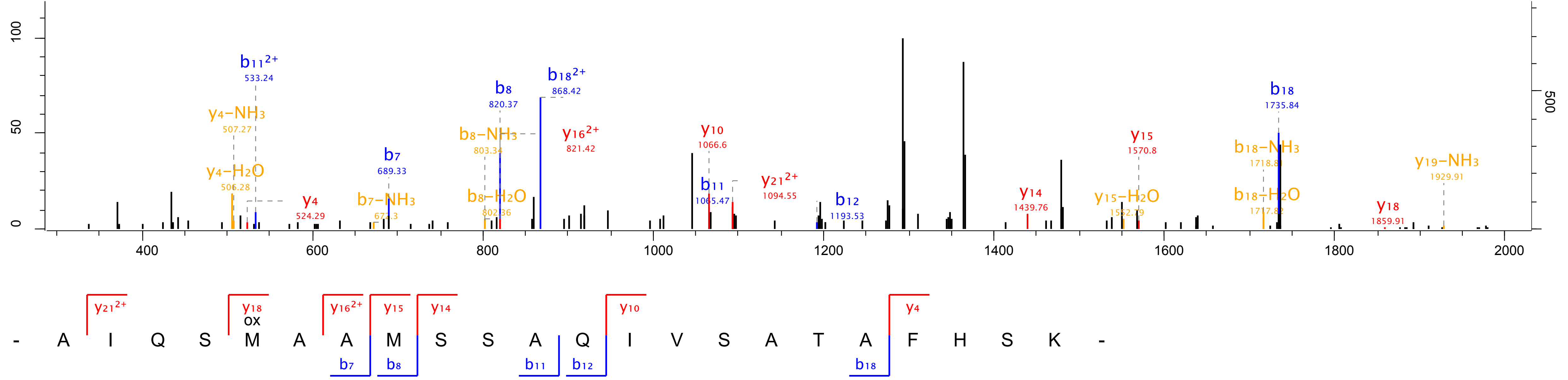

Raw file	Scan	Method	Score	m/z	Gene names
HBT_20130916_BV2_IC2_03	23403	ITMS; CID	53.45	801.74	Itm2c

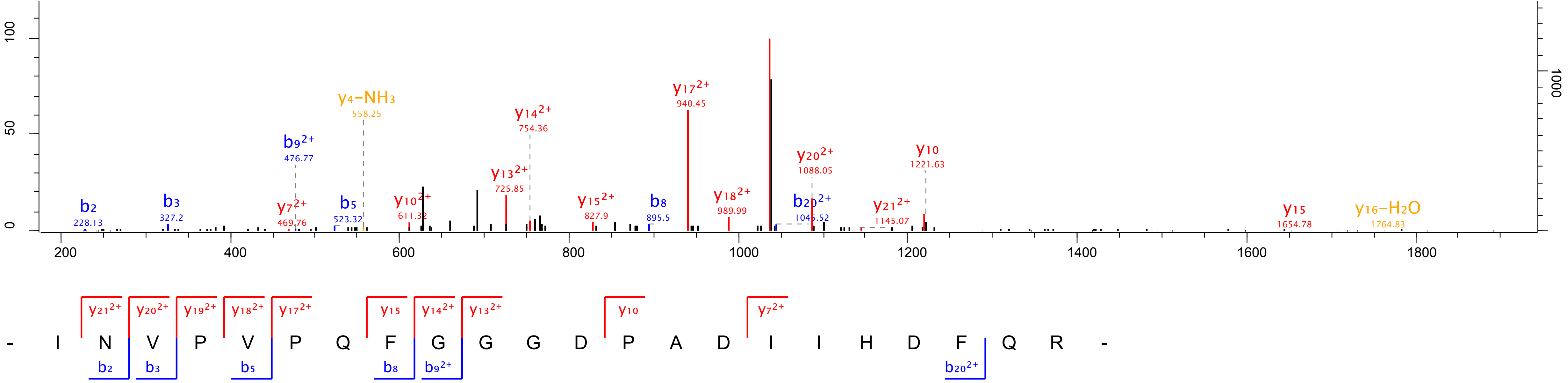

Raw file	Scan	Method	Score	m/z	Gene names
HBT_20130916_BV2_IC2_03	23222	ITMS; CID	84.3	605.3	Diaph2;Diap2

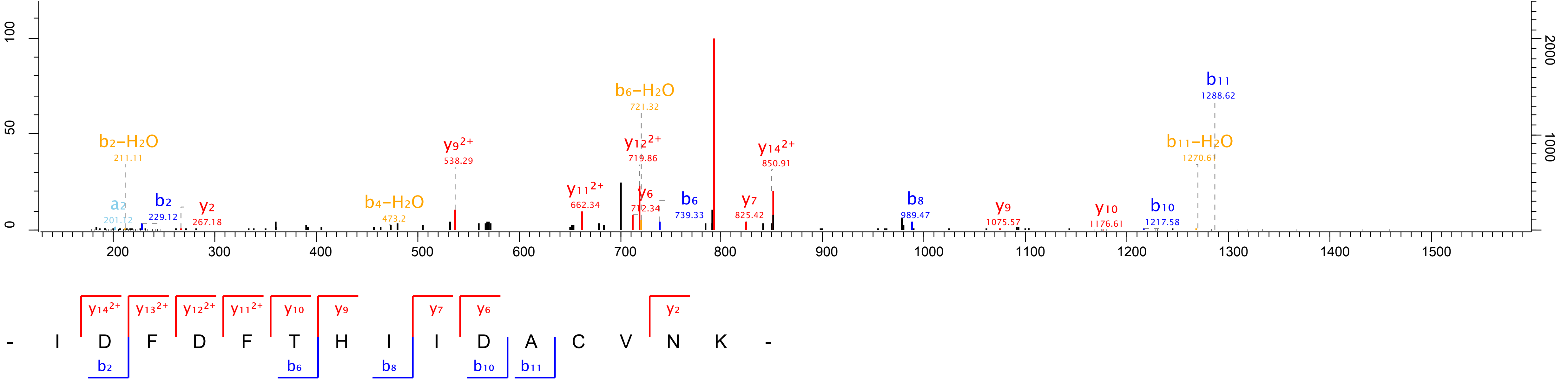

Raw file	Scan	Method	Score	m/z	Gene names
HBT_20130916_BV2_IC2_03	22737	ITMS; CID	66.89	796.37	Ccdc32

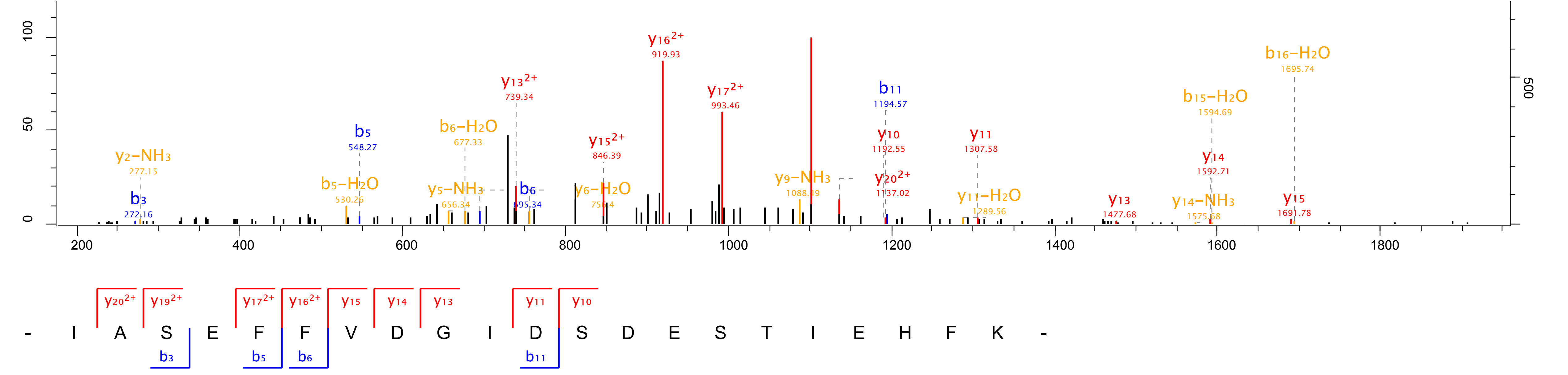

Raw file	Scan	Method	Score	m/z	Gene names
HBT_20130916_BV2_IC2_03	22689	ITMS; CID	38.99	1127.92	Iba57

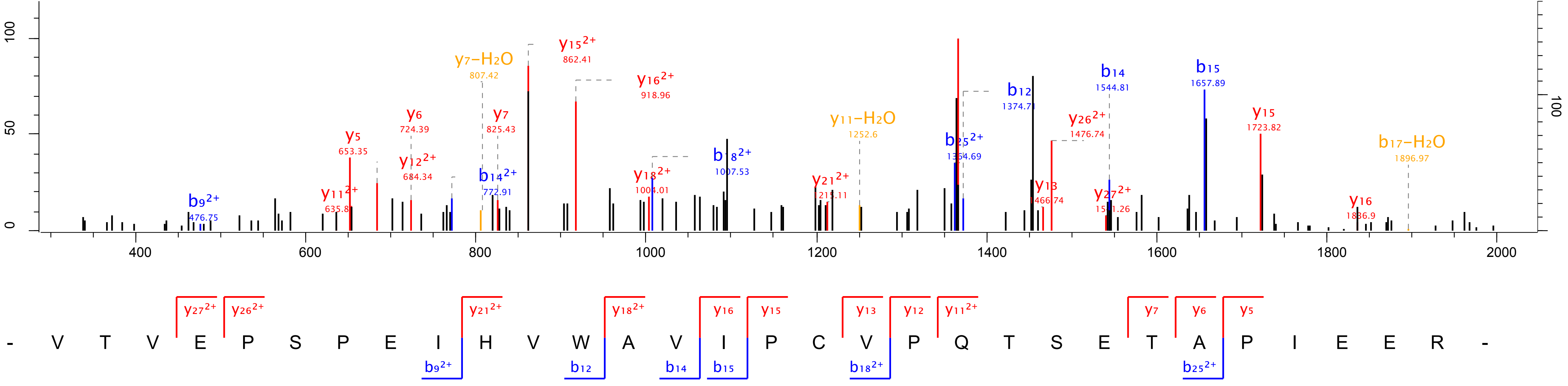

Raw file	Scan	Method	Score	m/z	Gene names
HBT_20130916_BV2_IC2_03	19568	ITMS; CID	45.89	844.79	Ccdc12

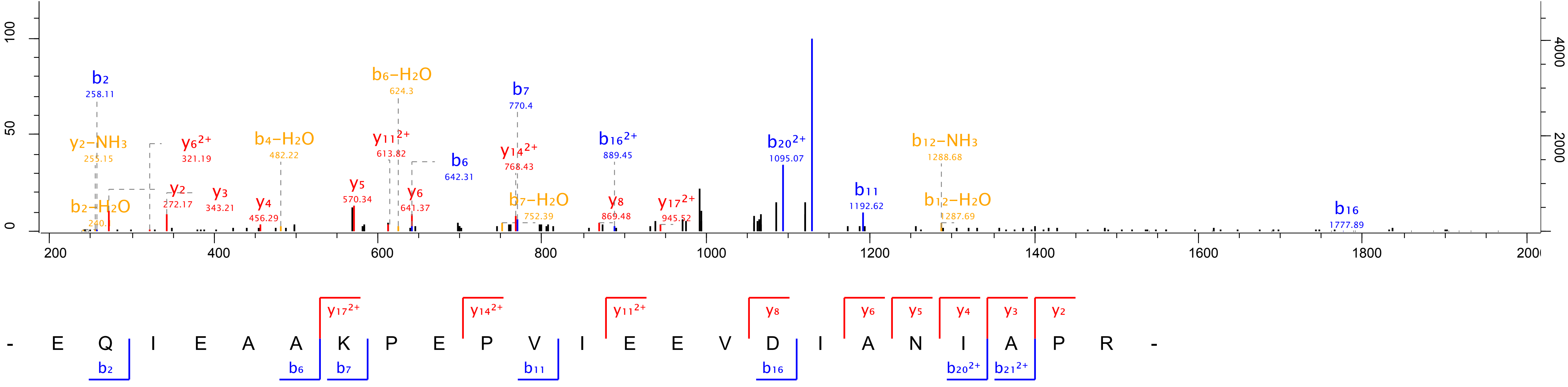

Raw file	Scan	Method	Score	m/z	Gene names
HBT_20130916_BV2_IC2_03	19356	ITMS; CID	70.12	874.46	Adam8

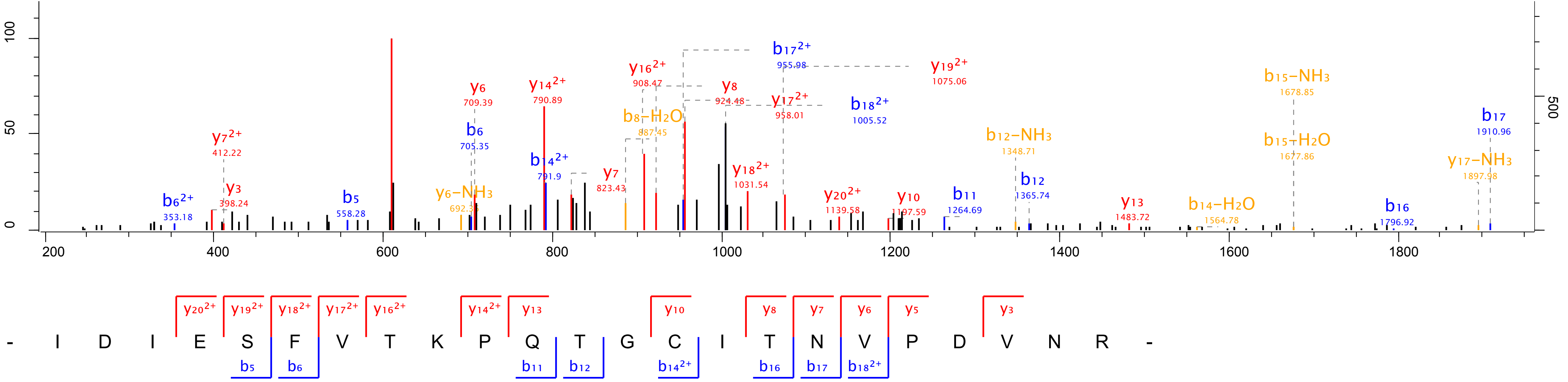

Raw file	Scan	Method	Score	m/z	Gene names
HBT_20130916_BV2_IC2_03	18935	ITMS; CID	109.11	540.28	Exog

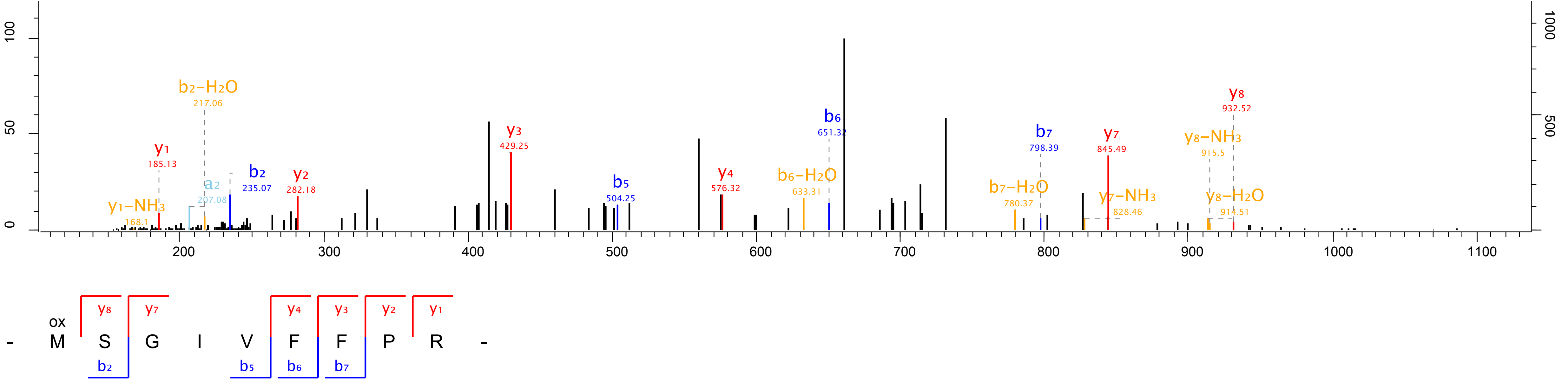

Raw file	Scan	Method	Score	m/z	Gene names
HBT_20130916_BV2_IC2_03	16189	ITMS; CID	71.18	571.96	2700097O09Rik

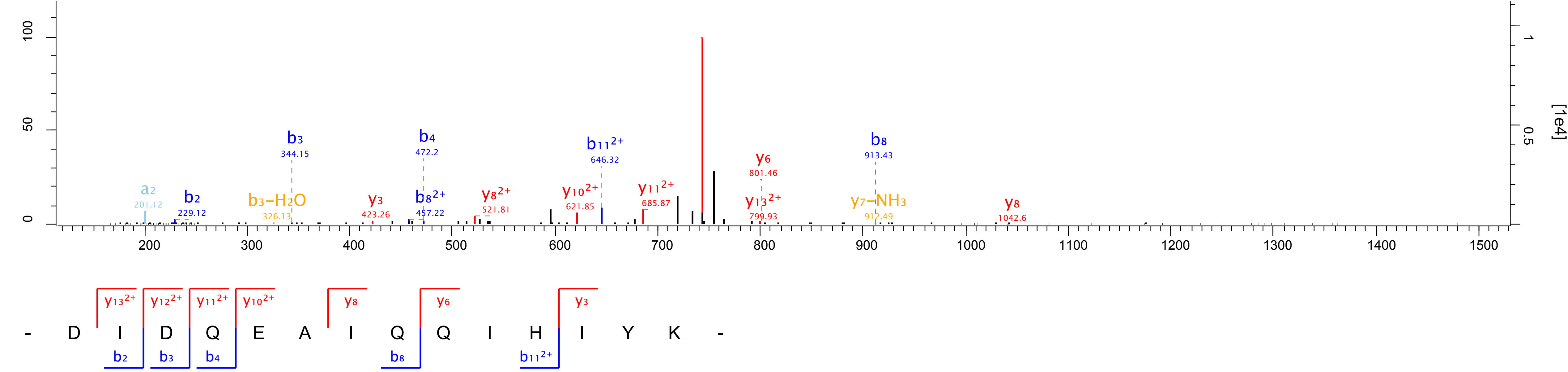

Raw file	Scan	Method	Score	m/z	Gene names
HBT_20130916_BV2_IC2_03	15952	ITMS; CID	52.73	764.73	Tmppe

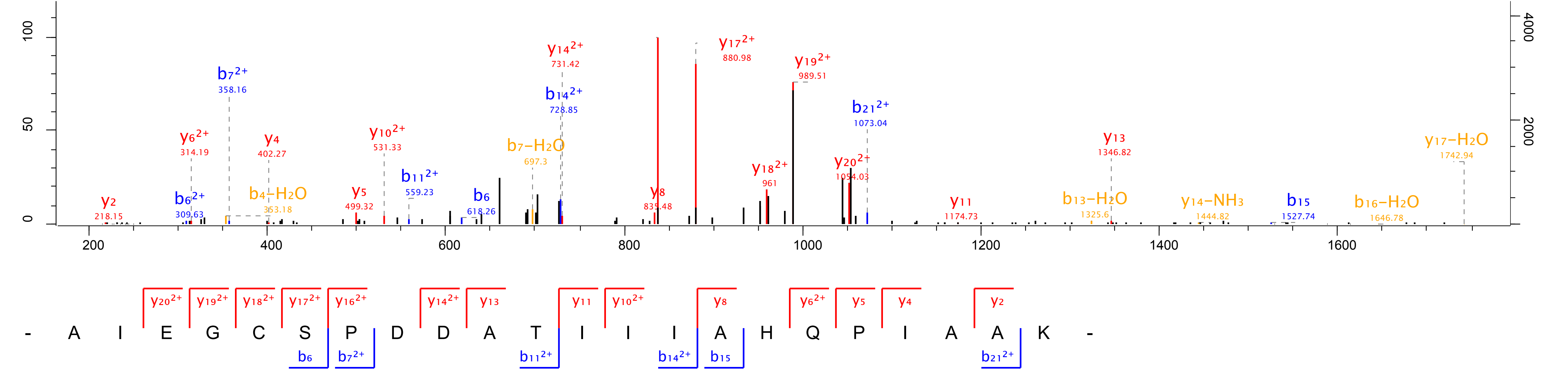

Raw file	Scan	Method	Score	m/z	Gene names
HBT_20130916_BV2_IC2_03	15864	ITMS; CID	110.84	631.99	Nenf

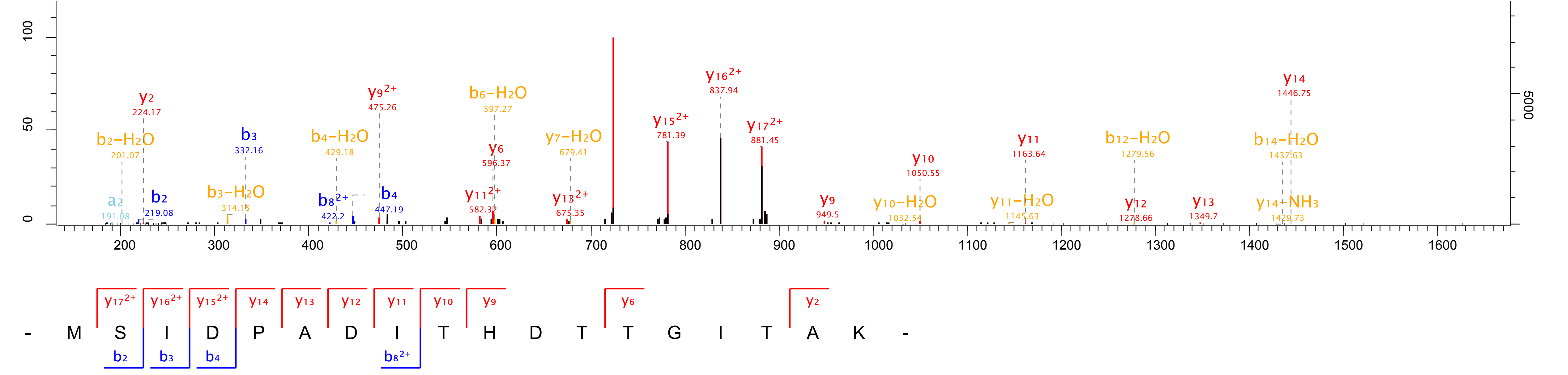

Raw file	Scan	Method	Score	m/z	Gene names
HBT_20130916_BV2_IC2_03	14777	ITMS; CID	158.31	840.39	Rab24

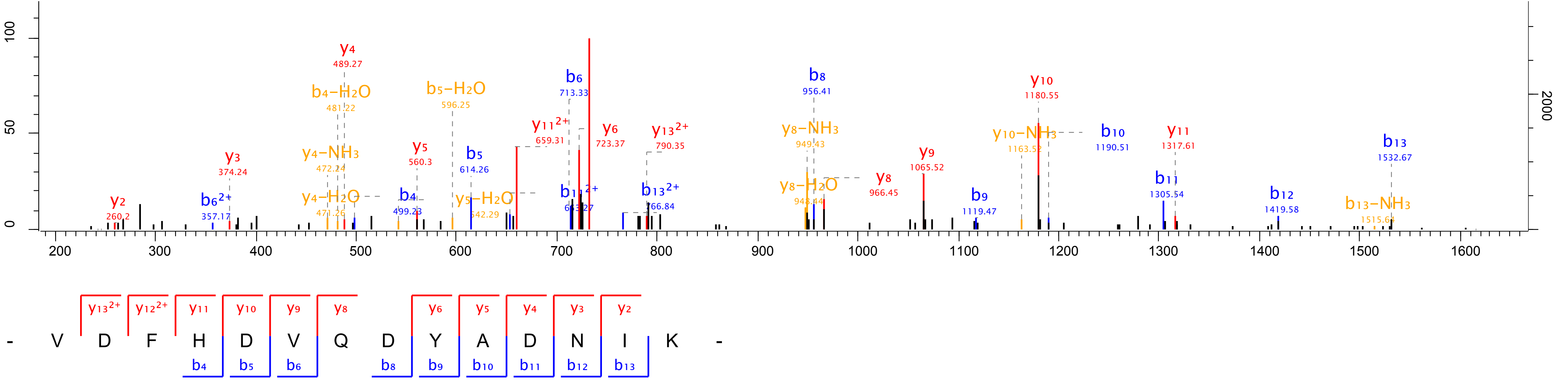

Raw file	Scan	Method	Score	m/z	Gene names
HBT_20130916_BV2_IC2_03	13395	ITMS; CID	76.68	882.45	Impact

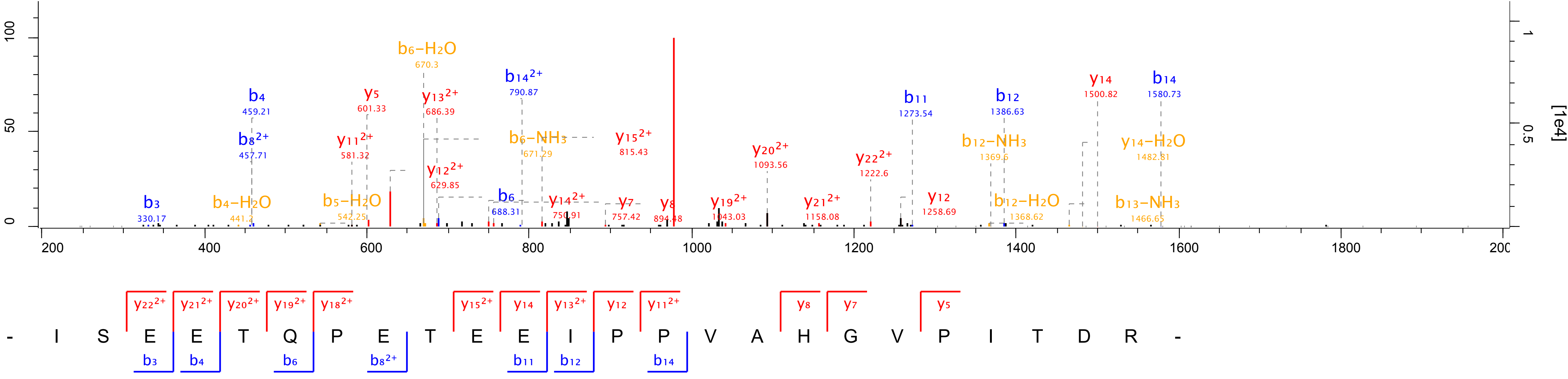

Raw file	Scan	Method	Score	m/z	Gene names
HBT_20130916_BV2_IC2_03	12037	ITMS; CID	59.89	1154.87	Sde2

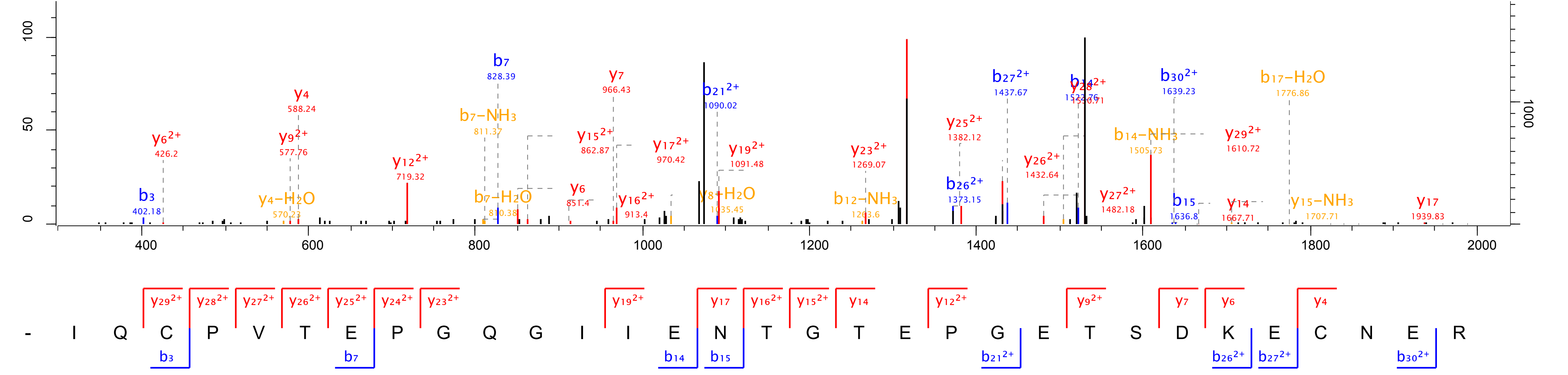

Raw file	Scan	Method	Score	m/z	Gene names
HBT_20130916_BV2_IC2_03	10969	ITMS; CID	88.1	576.26	Tmem9b

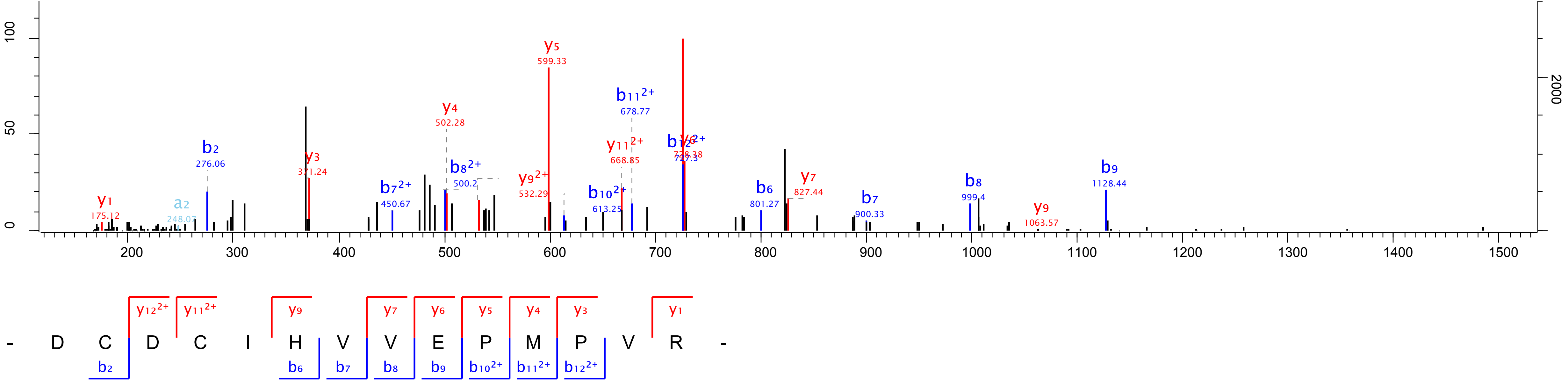

Raw file	Scan	Method	Score	m/z	Gene names
HBT_20130916_BV2_IC2_03	10744	ITMS; CID	85.74	816.74	Phlpp1

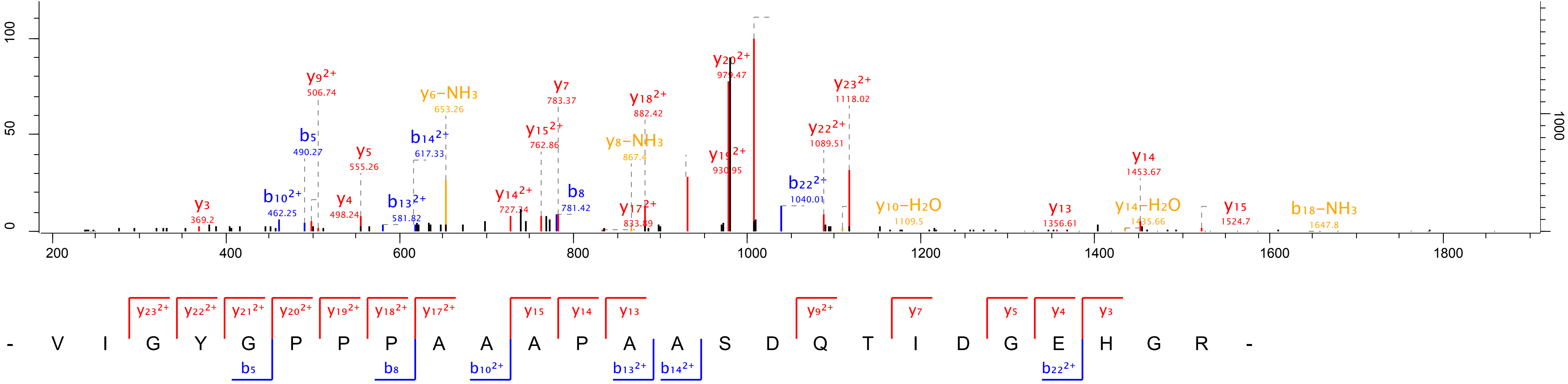

Raw file	Scan	Method	Score	m/z	Gene names
HBT_20130916_BV2_IC2_02	18082	ITMS; CID	115.63	1116.07	Chek2

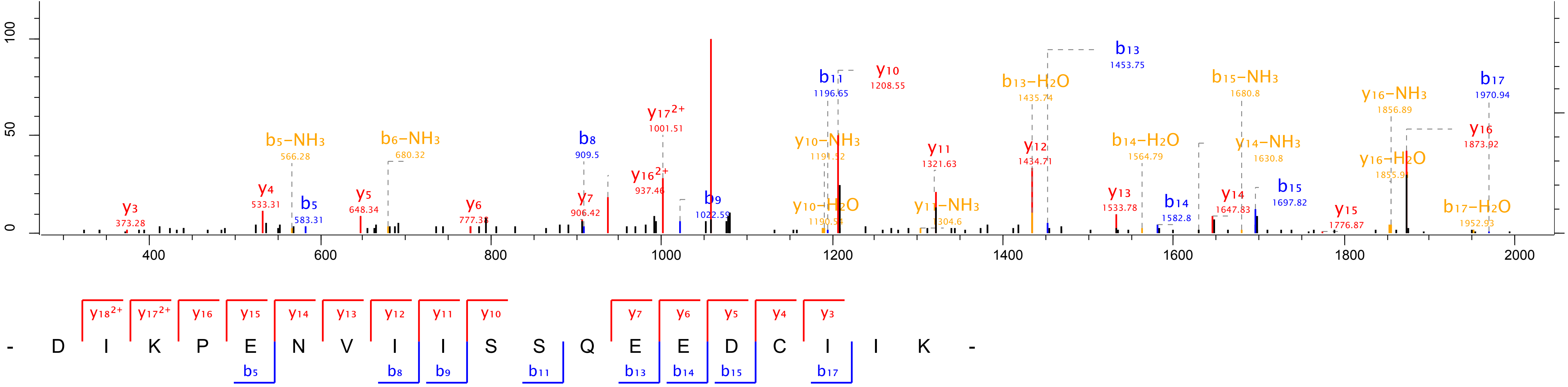

Raw file	Scan	Method	Score	m/z	Gene names
HBT_20130916_BV2_IC2_02	1686	ITMS; CID	116.84	585.82	Mtss1

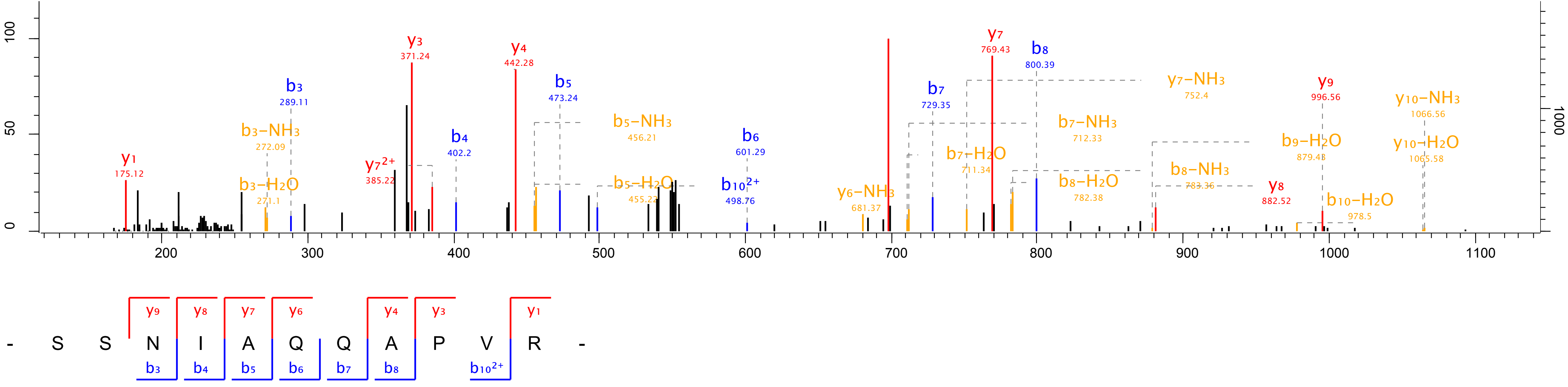

Raw file	Scan	Method	Score	m/z	Gene names
HBT_20130916_BV2_IC2_02	1171	ITMS; CID	150.36	423.25	Ccdc6

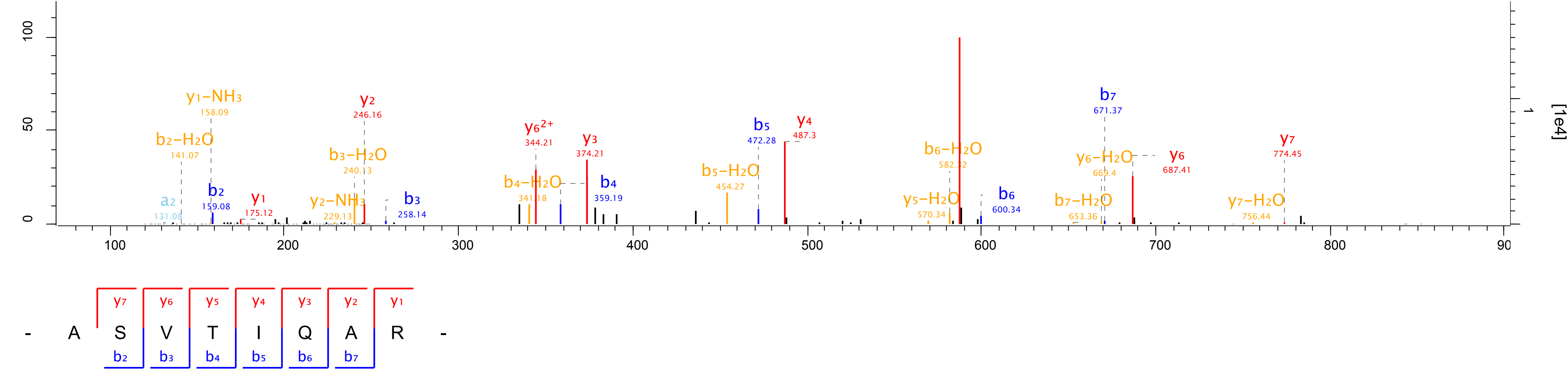

Raw file	Scan	Method	Score	m/z	Gene names
HBT_20130916_BV2_IC2_01	5381	ITMS; CID	85.16	734.34	6430548M08Rik;Kiaa0513

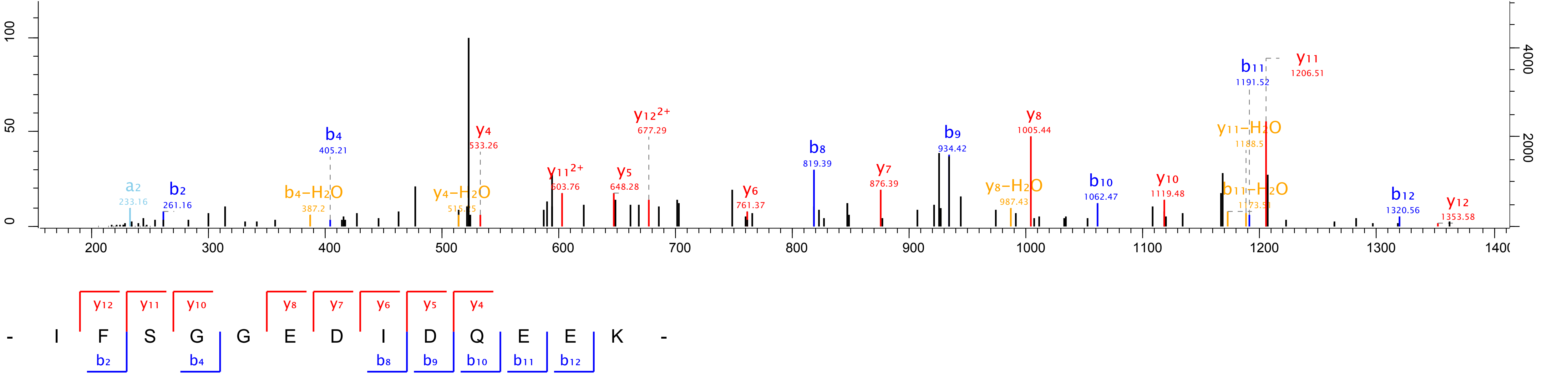

Raw file	Scan	Method	Score	m/z	Gene names
HBT_20130916_BV2_IC2_01	3812	ITMS; CID	85.47	697.84	Uros

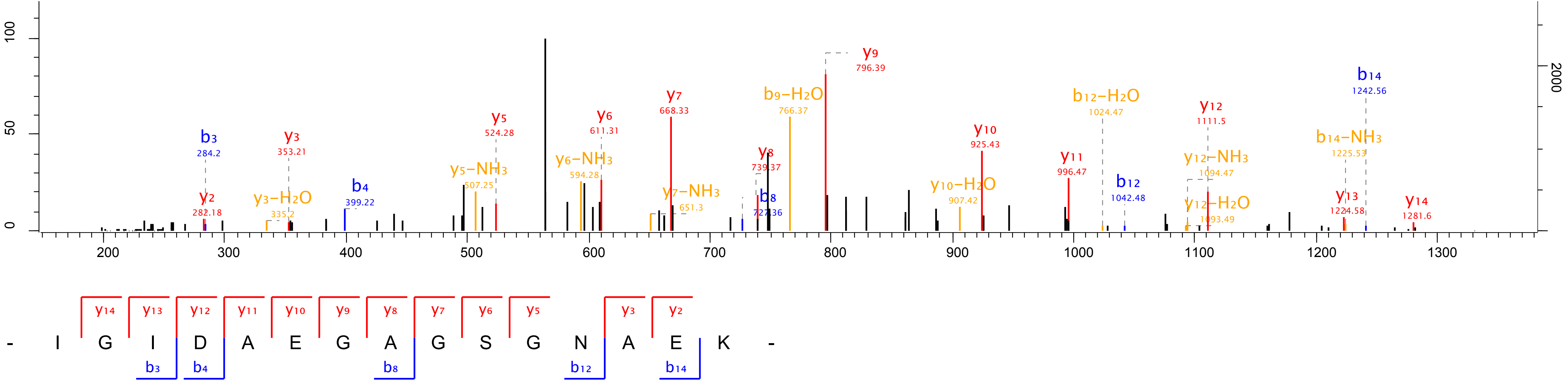

Raw file	Scan	Method	Score	m/z	Gene names
HBT_20130916_BV2_IC2_01	3755	ITMS; CID	111.58	836.93	Myadm

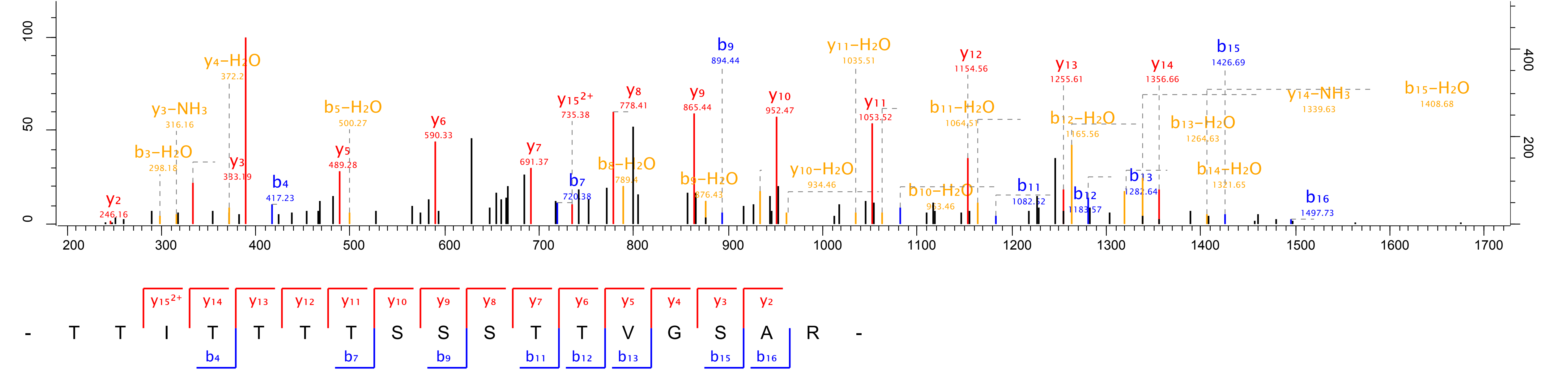

Raw file	Scan	Method	Score	m/z	Gene names
HBT_20130916_BV2_IC2_01	3645	ITMS; CID	140.78	584.8	Lztfl1

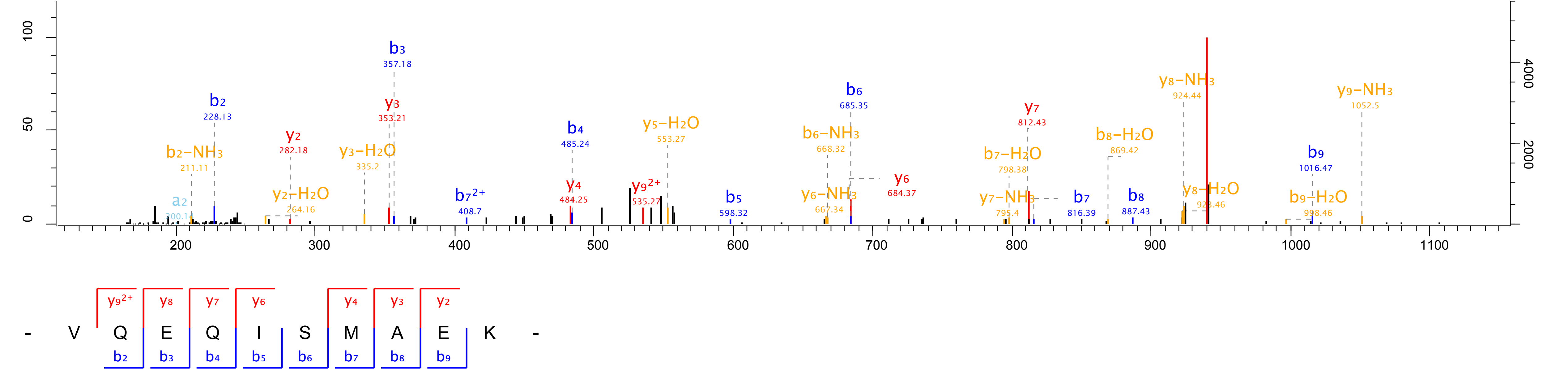

Raw file	Scan	Method	Score	m/z	Gene names
HBT_20130916_BV2_IC2_01	3623	ITMS; CID	93.91	833.36	Akap11

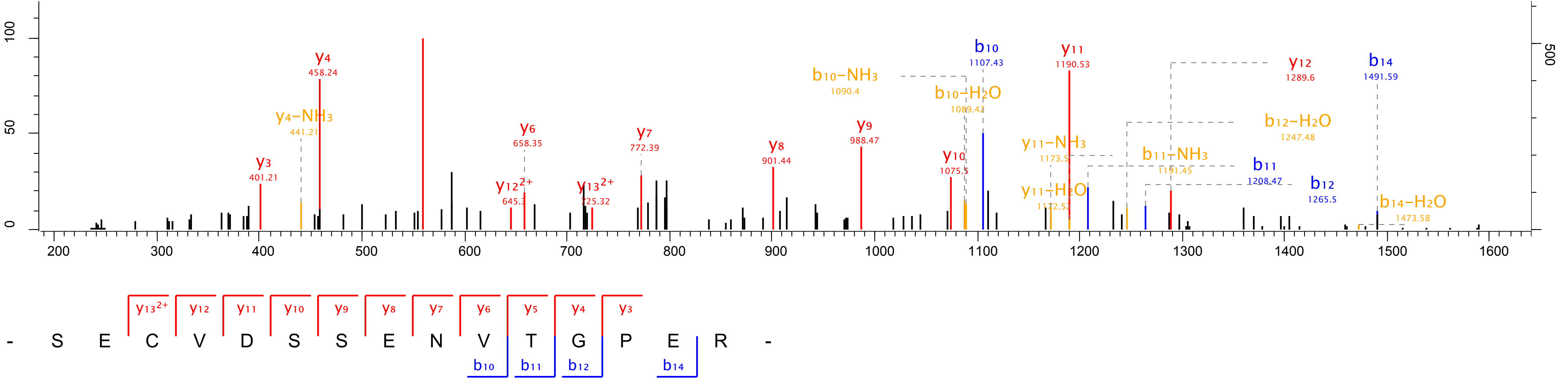

Raw file	Scan	Method	Score	m/z	Gene names
HBT_20130916_BV2_IC1_06	3871	ITMS; CID	73.8	942.96	Nynrin

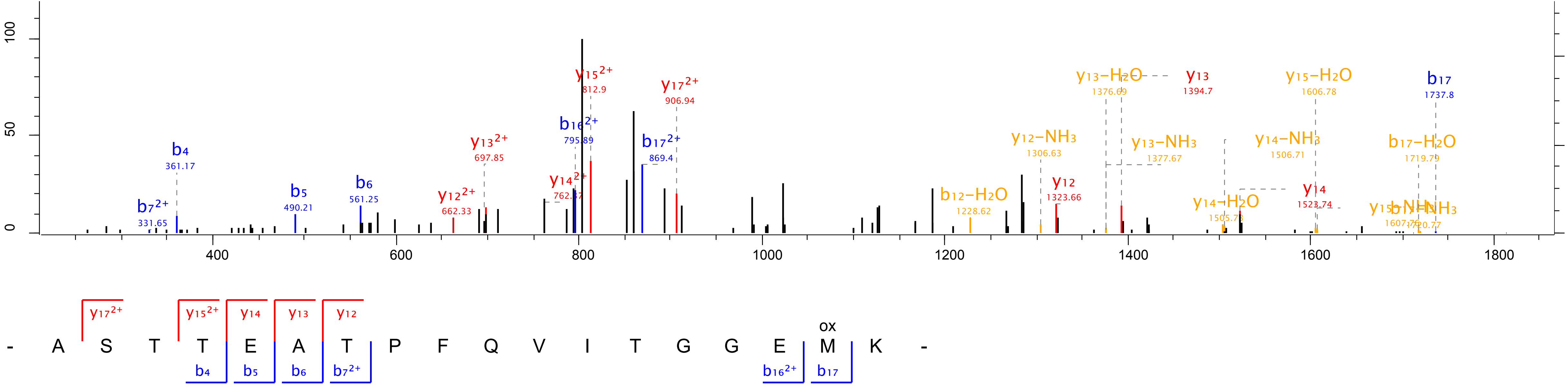

Raw file	Scan	Method	Score	m/z	Gene names
HBT_20130916_BV2_IC1_06	18802	ITMS; CID	55.69	1106.86	Cox6a1

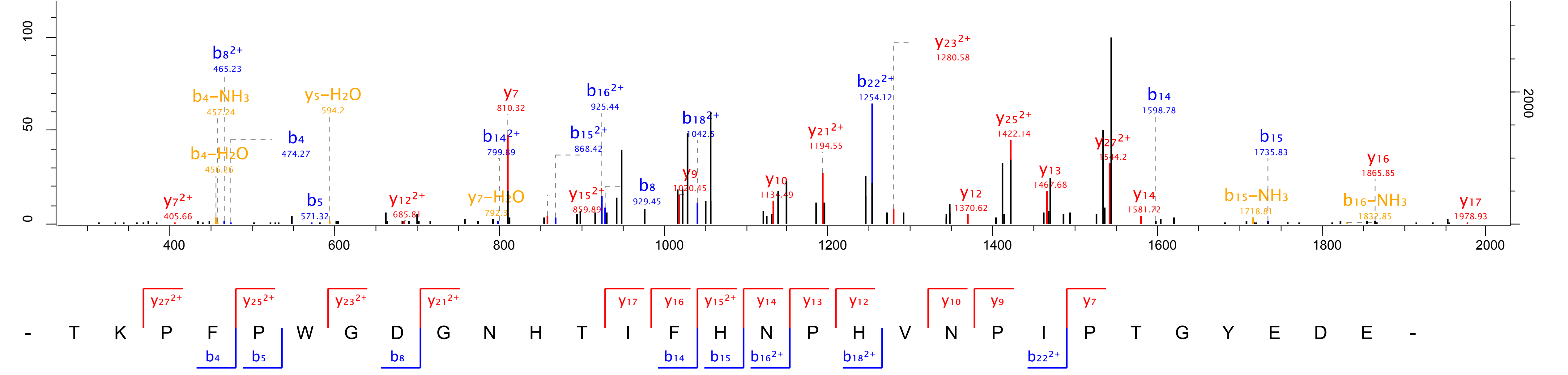

Raw file	Scan	Method	Score	m/z	Gene names
HBT_20130916_BV2_IC1_06	12748	ITMS; CID	57.17	777.71	Bckdhb

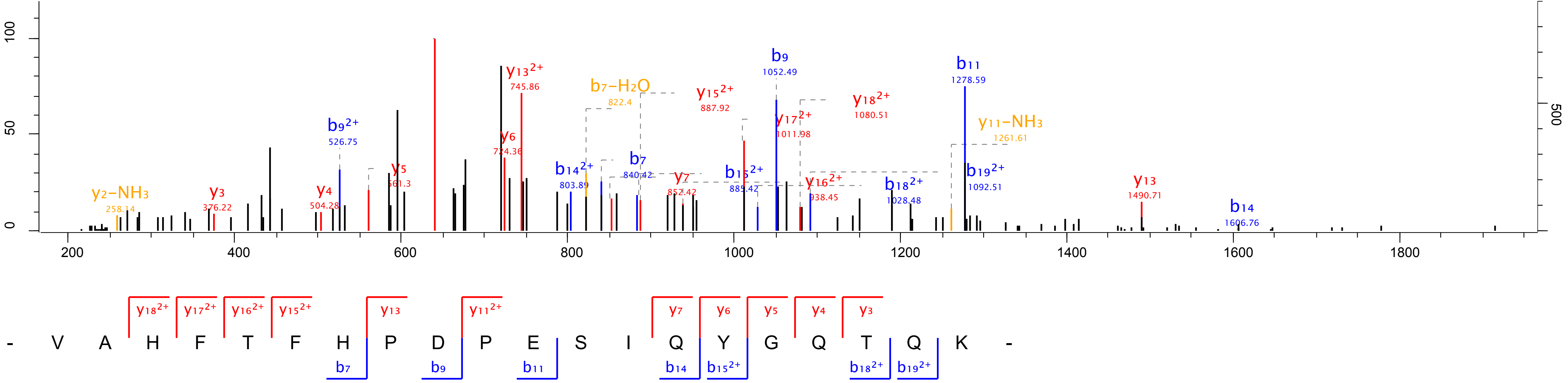

Raw file	Scan	Method	Score	m/z	Gene names
HBT_20130916_BV2_IC1_06	10998	ITMS; CID	62.68	561.31	Tmem251

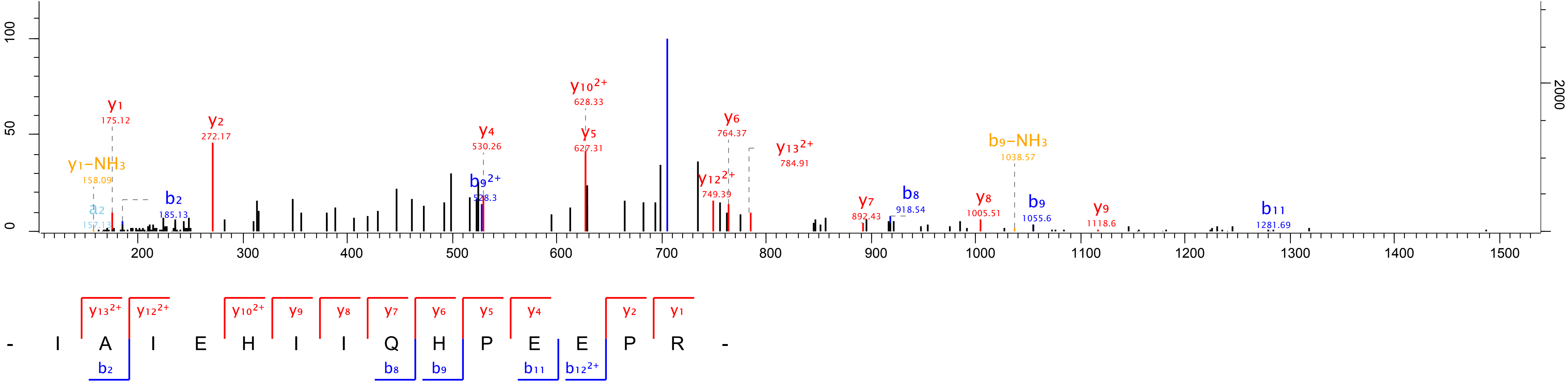

Raw file	Scan	Method	Score	m/z	Gene names
HBT_20130916_BV2_IC1_06	10606	ITMS; CID	98.13	884.42	Gpr84

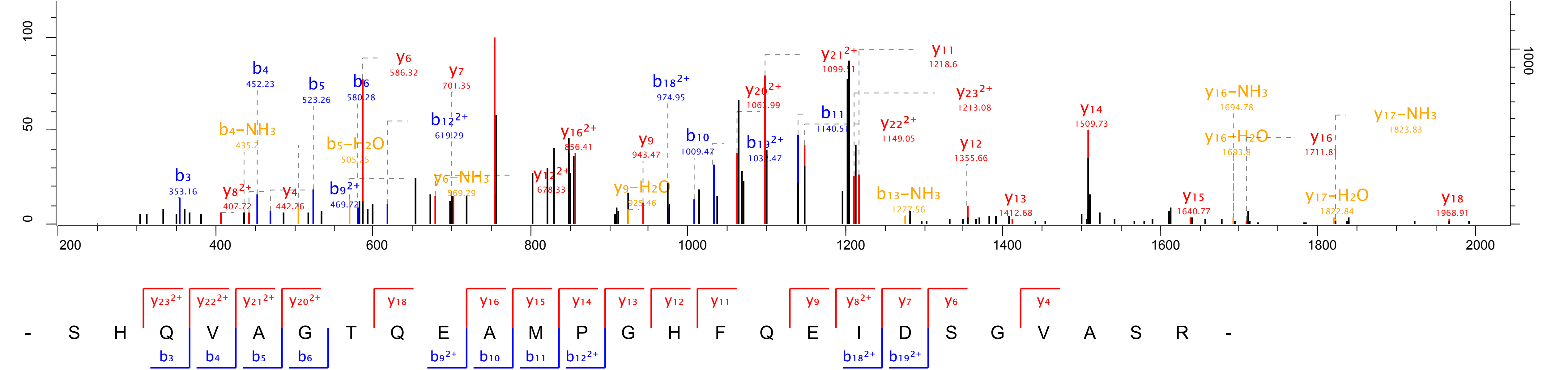

Raw file	Scan	Method	Score	m/z	Gene names
HBT_20130916_BV2_IC1_05	4407	ITMS; CID	120.15	992	Pinx1

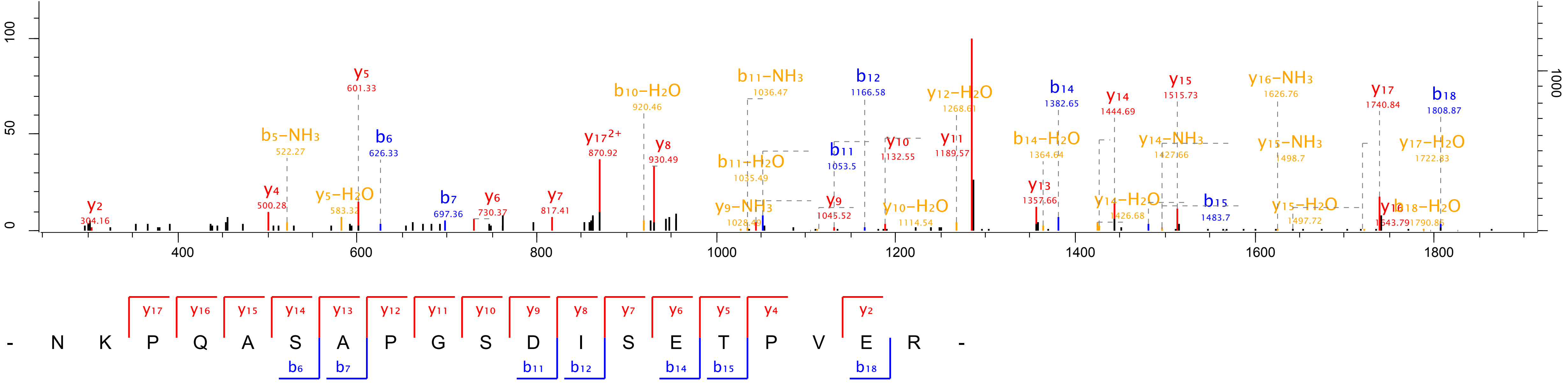

Raw file	Scan	Method	Score	m/z	Gene names
HBT_20130916_BV2_IC1_05	21614	ITMS; CID	66.29	719.06	Ppil3

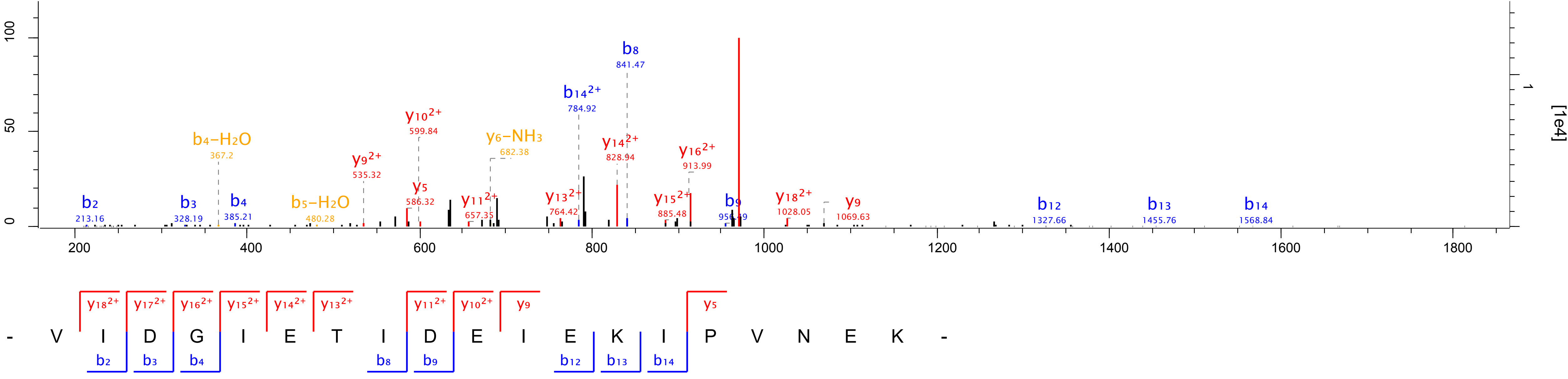

Raw file	Scan	Method	Score	m/z	Gene names
HBT_20130916_BV2_IC1_05	1367	ITMS; CID	150.92	883.93	Tsen15

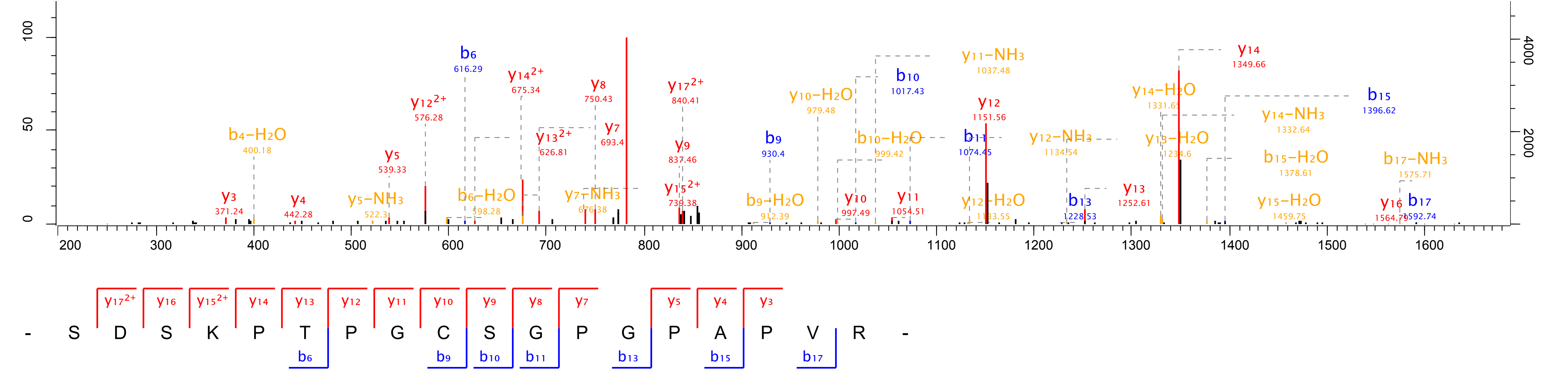

Raw file	Scan	Method	Score	m/z	Gene names
HBT_20130916_BV2_IC1_05	13351	ITMS; CID	83.96	1018.04	Ube2a

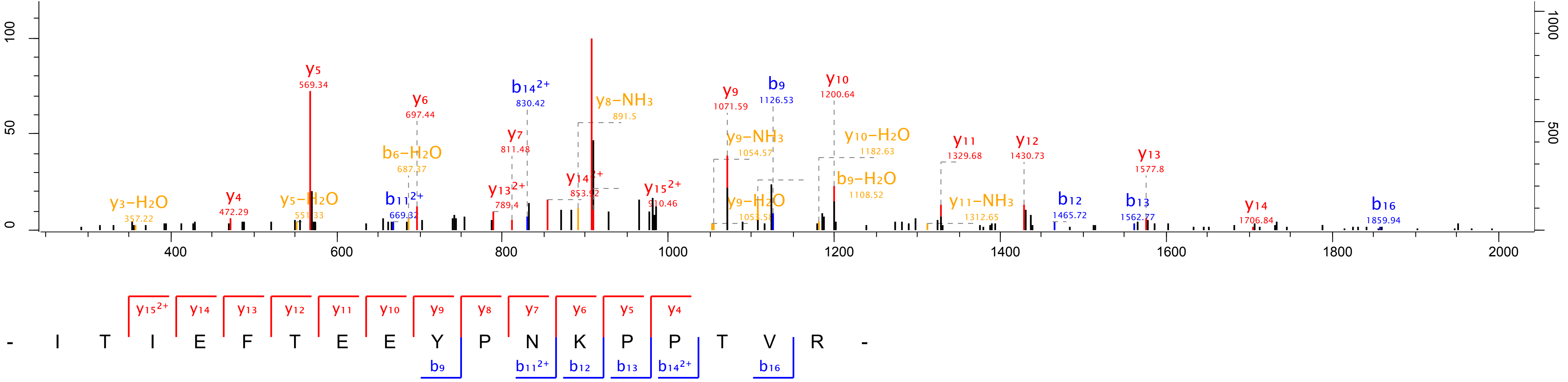

Raw file	Scan	Method	Score	m/z	Gene names
HBT_20130916_BV2_IC1_05	1010	ITMS; CID	99.37	673.95	Akap13

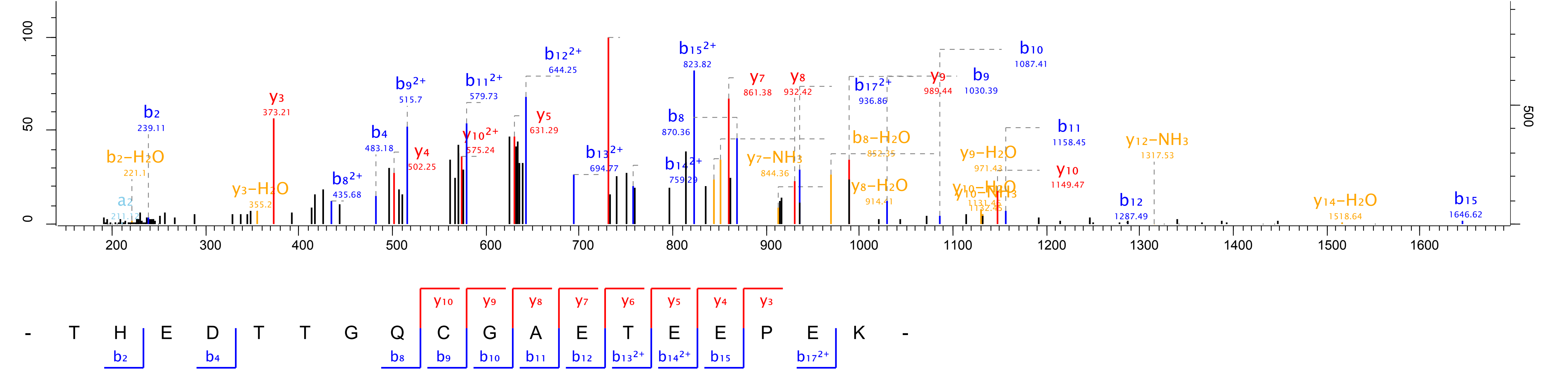

Raw file	Scan	Method	Score	m/z	Gene names
HBT_20130916_BV2_IC1_04	21306	ITMS; CID	49.33	864.47	Znf143

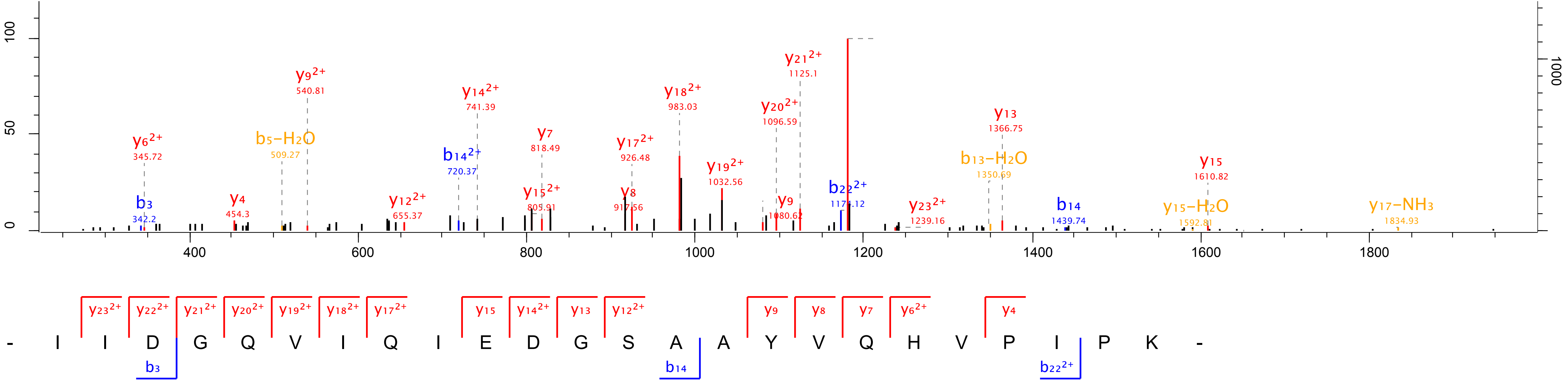

Raw file	Scan	Method	Score	m/z	Gene names
HBT_20130916_BV2_IC1_04	17220	ITMS; CID	90.71	716.37	Pde6d

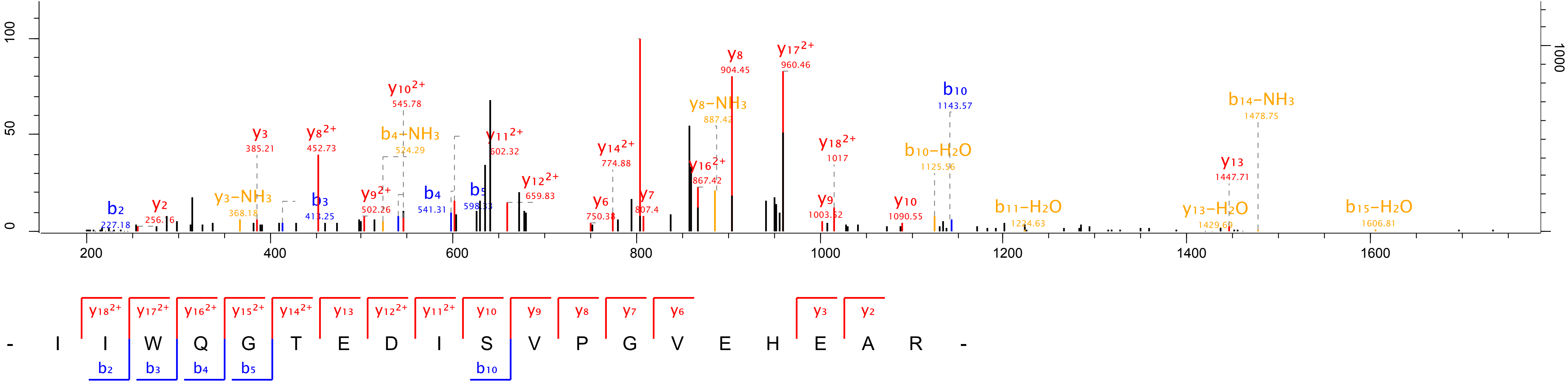

Raw file	Scan	Method	Score	m/z	Gene names
HBT_20130916_BV2_IC1_04	12957	ITMS; CID	64.24	635.65	Rsrc1

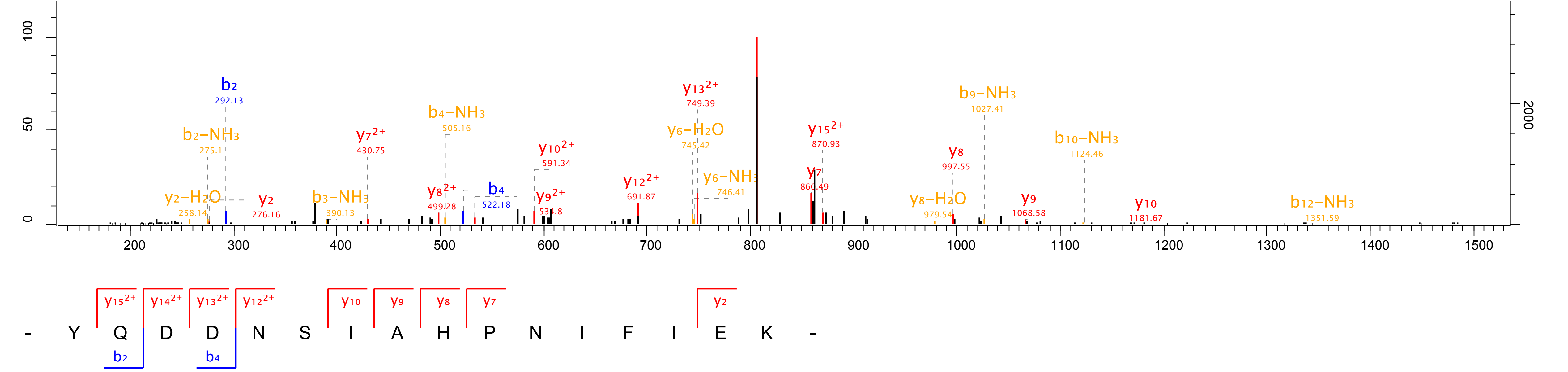

Raw file	Scan	Method	Score	m/z	Gene names
HBT_20130916_BV2_IC1_03	25678	ITMS; CID	99.66	698.39	Scoc

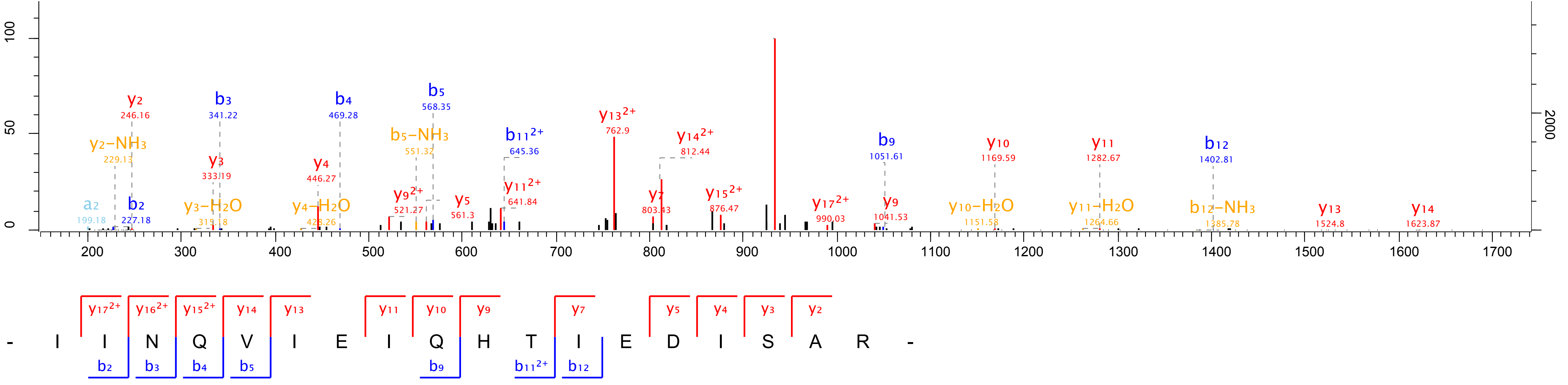

Raw file	Scan	Method	Score	m/z	Gene names
HBT_20130916_BV2_IC1_03	19391	ITMS; CID	81.59	760.68	Ss18

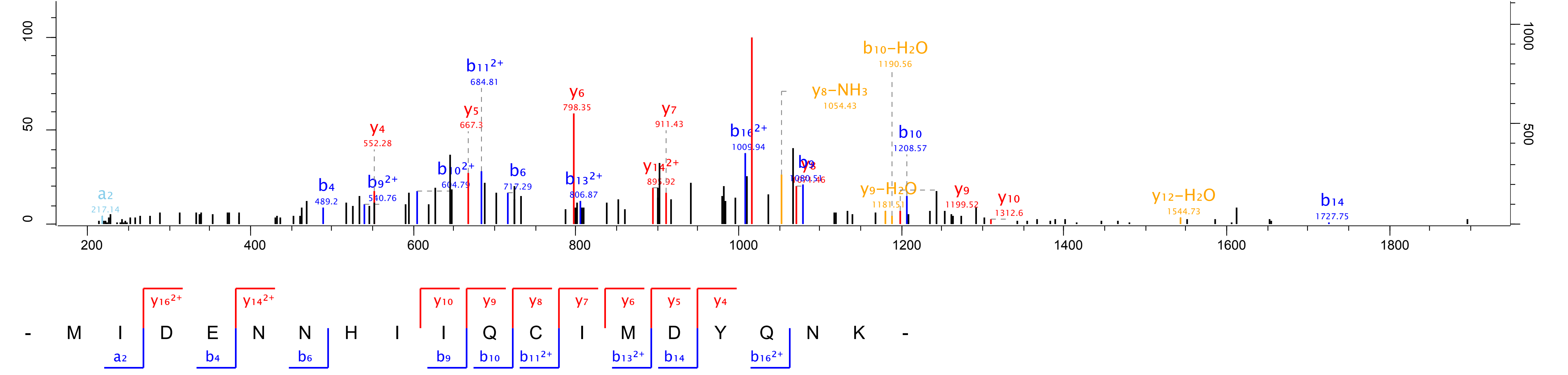

Raw file	Scan	Method	Score	m/z	Gene names
HBT_20130916_BV2_IC1_03	1234	ITMS; CID	163.62	861.88	Slc43a2

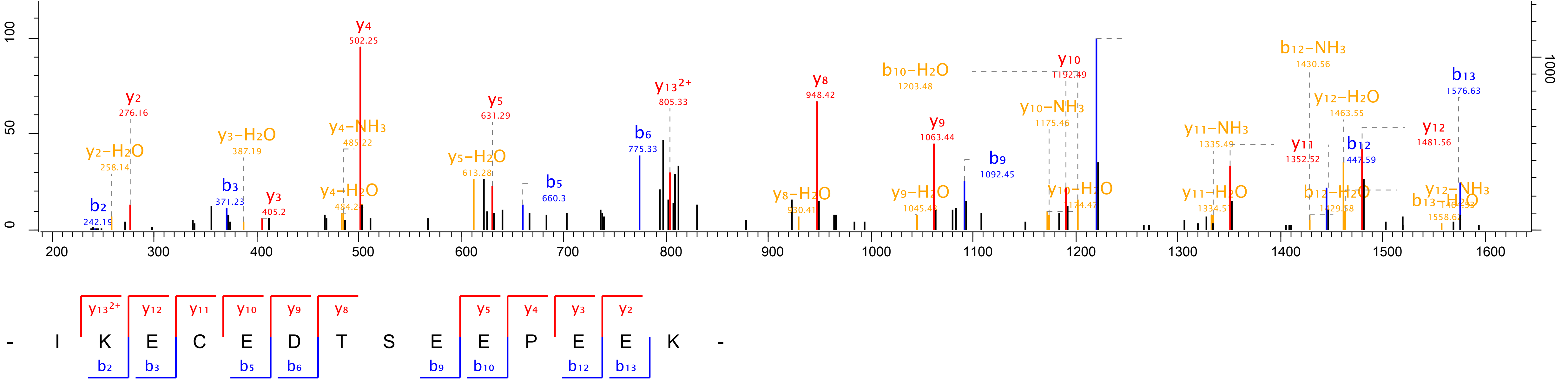

Raw file	Scan	Method	Score	m/z	Gene names
HBT_20130916_BV2_IC1_02	2914	ITMS; CID	83.18	582.31	1110065P20Rik

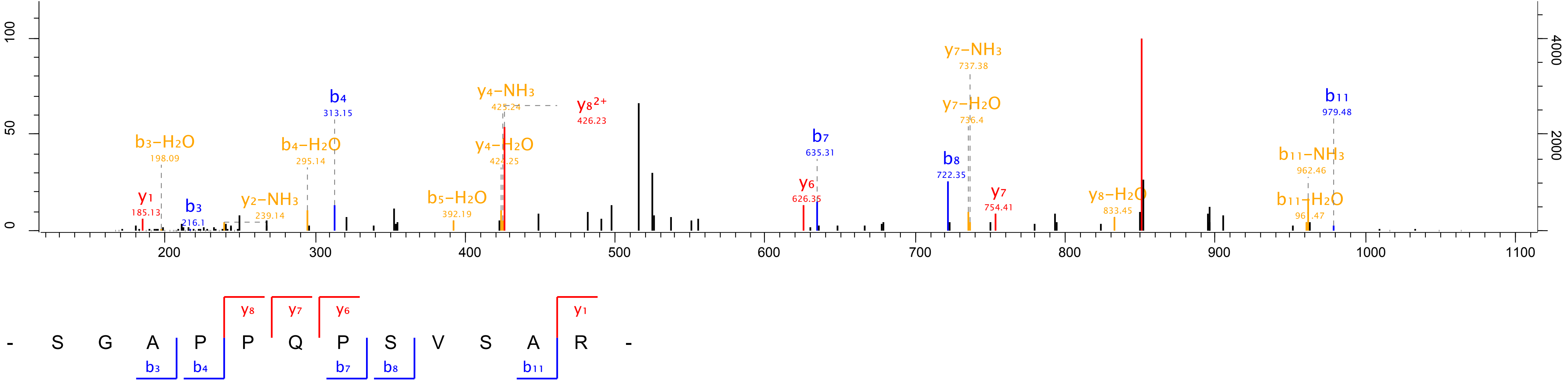

Raw file	Scan	Method	Score	m/z	Gene names
HBT_20130916_BV2_IC1_02	27873	ITMS; CID	74.03	818.12	Aktip

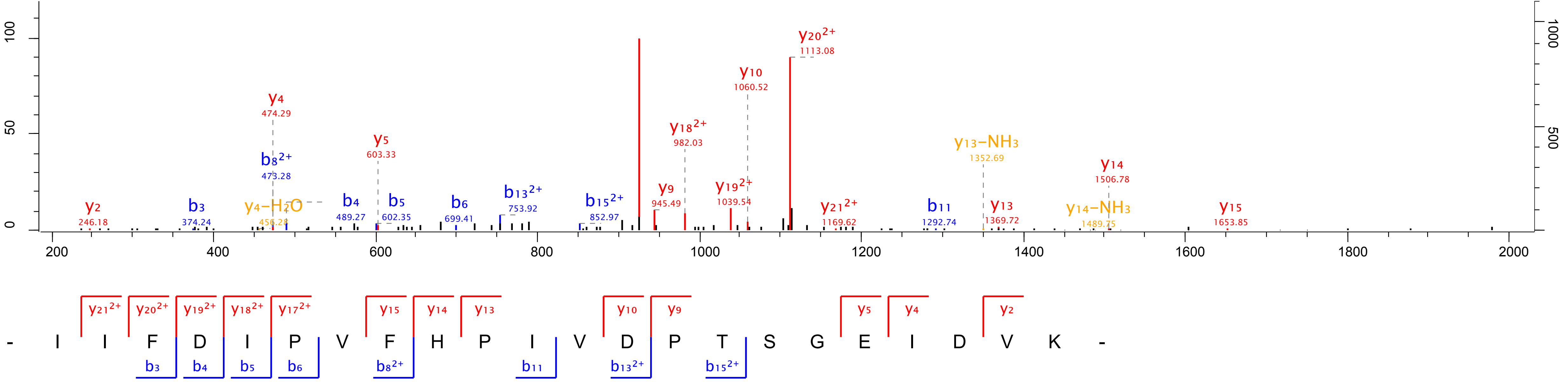

Raw file	Scan	Method	Score	m/z	Gene names
HBT_20130916_BV2_IC1_02	25151	ITMS; CID	76.24	1051.88	Dag1

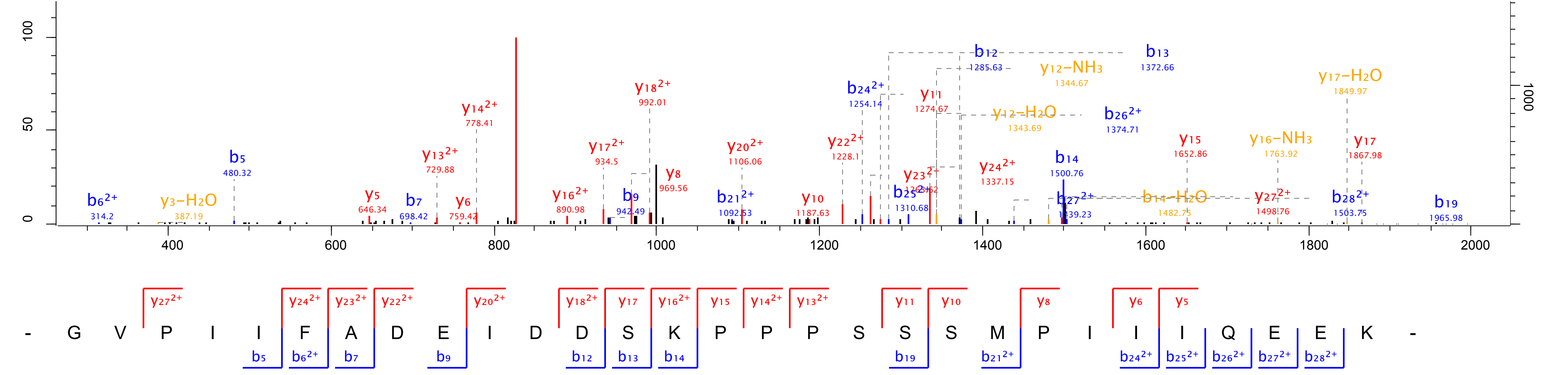

Raw file	Scan	Method	Score	m/z	Gene names
HBT_20130916_BV2_IC1_02	24968	ITMS; CID	82.48	627.66	Pld1

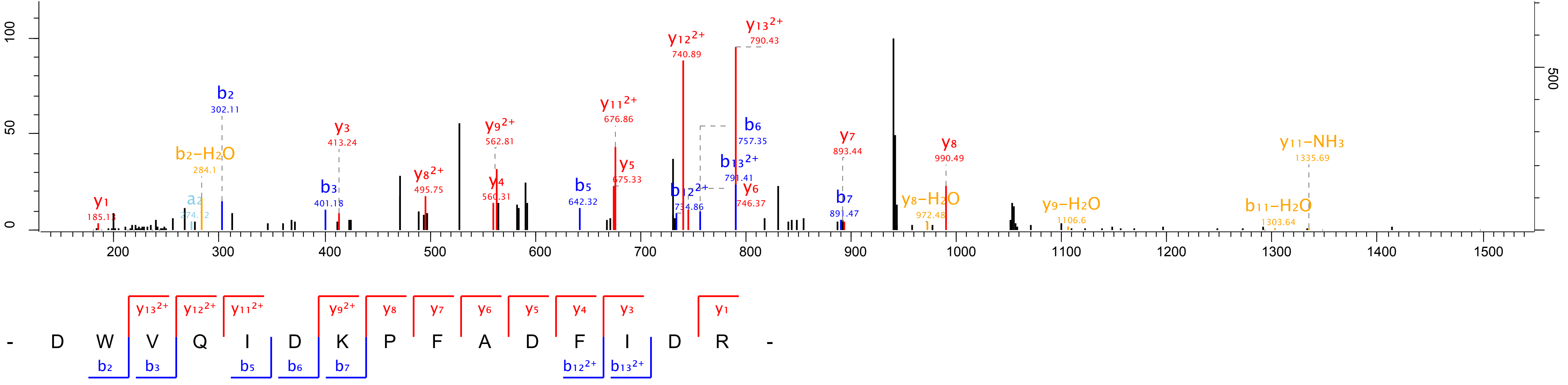

Raw file	Scan	Method	Score	m/z	Gene names
HBT_20130916_BV2_IC1_02	21354	ITMS; CID	100.82	578.35	Trpc4ap

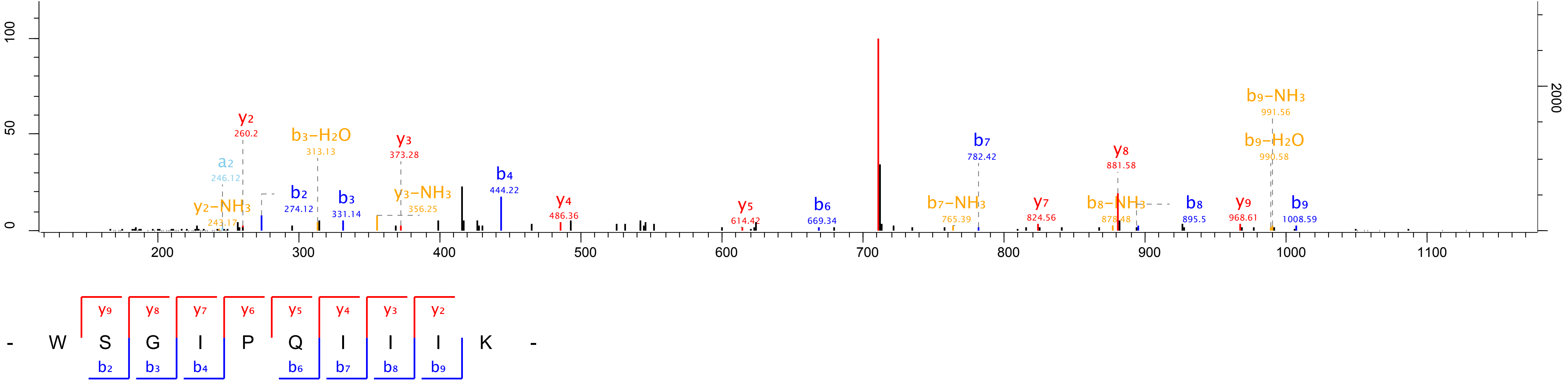

Raw file	Scan	Method	Score	m/z	Gene names
HBT_20130916_BV2_IC1_02	19459	ITMS; CID	146.27	860.91	Appl2

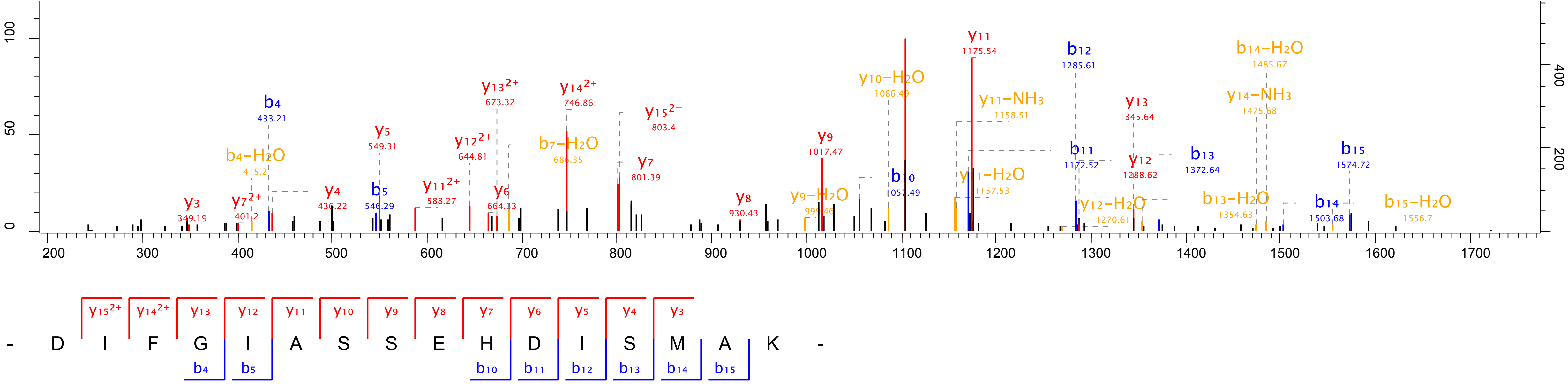

Raw file	Scan	Method	Score	m/z	Gene names
HBT_20130916_BV2_IC1_02	17732	ITMS; CID	115.7	463.79	Rpp25l

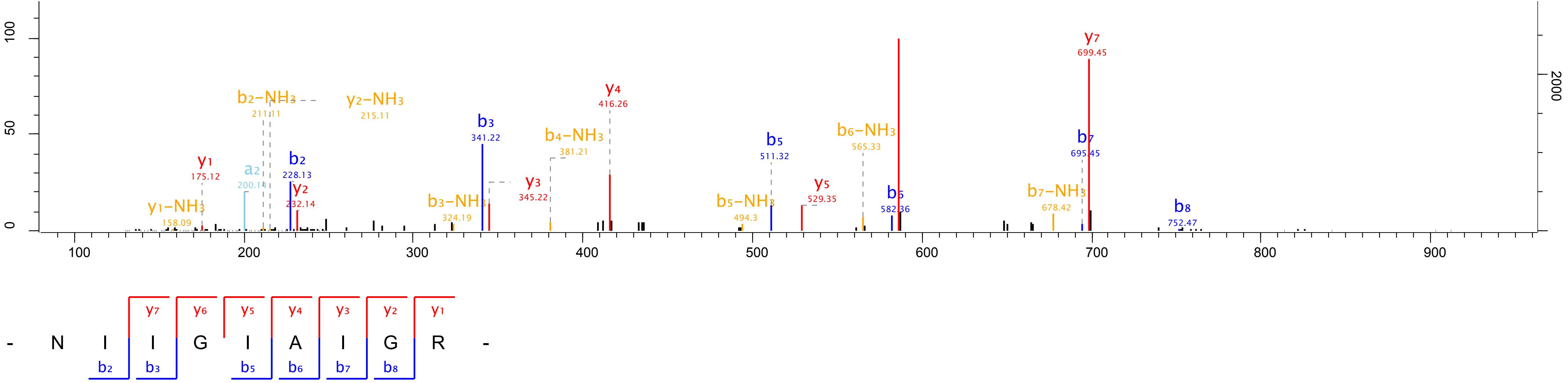

Raw file	Scan	Method	Score	m/z	Gene names
HBT_20130916_BV2_IC1_02	15183	ITMS; CID	122.52	671.86	Fam64a

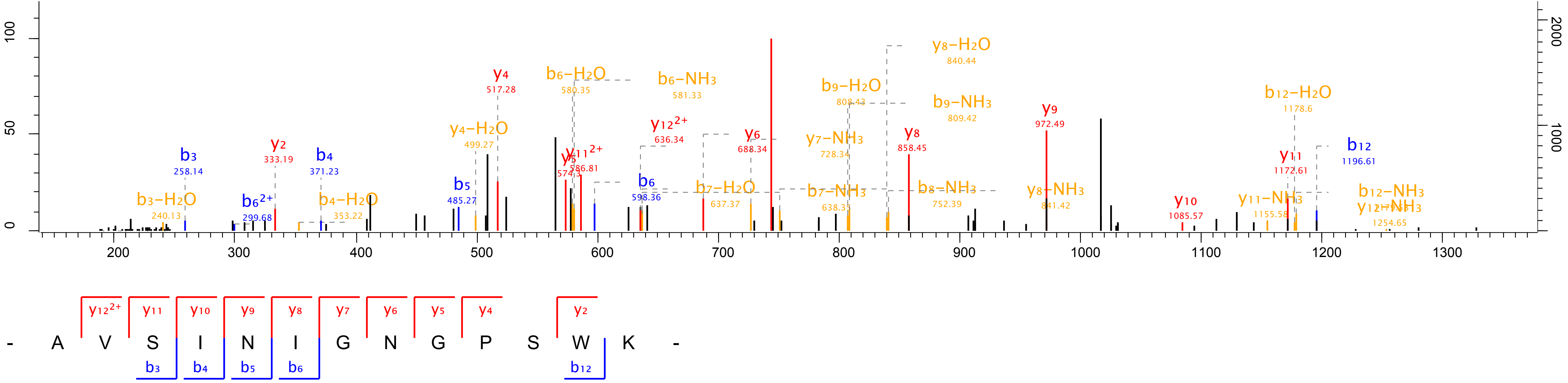

Raw file	Scan	Method	Score	m/z	Gene names
HBT_20130916_BV2_IC1_02	11356	ITMS; CID	117.86	639.85	Rtf1

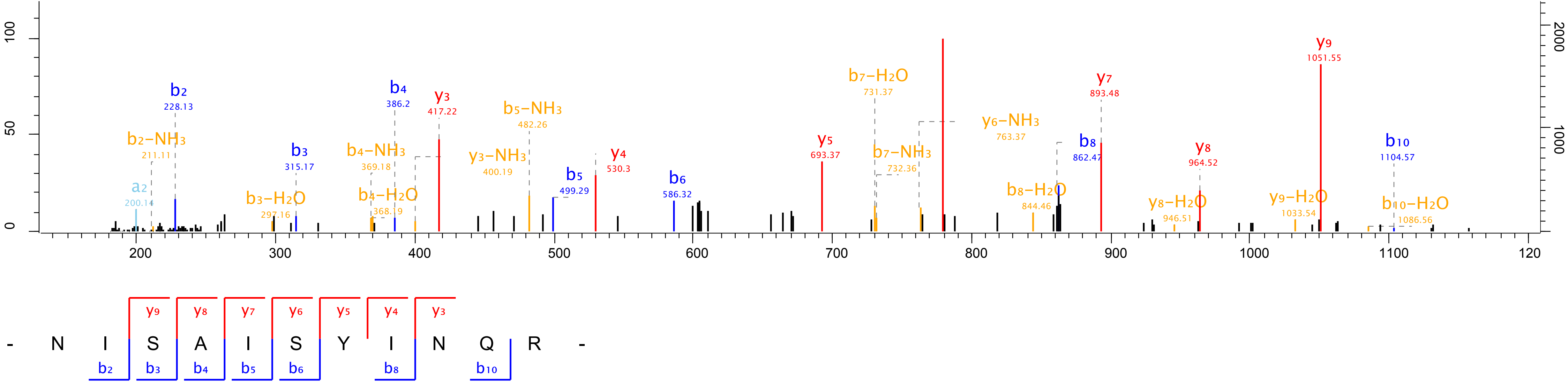

Raw file	Scan	Method	Score	m/z	Gene names
HBT_20130916_BV2_IC1_01	5567	ITMS; CID	92.27	852.39	Galnt6

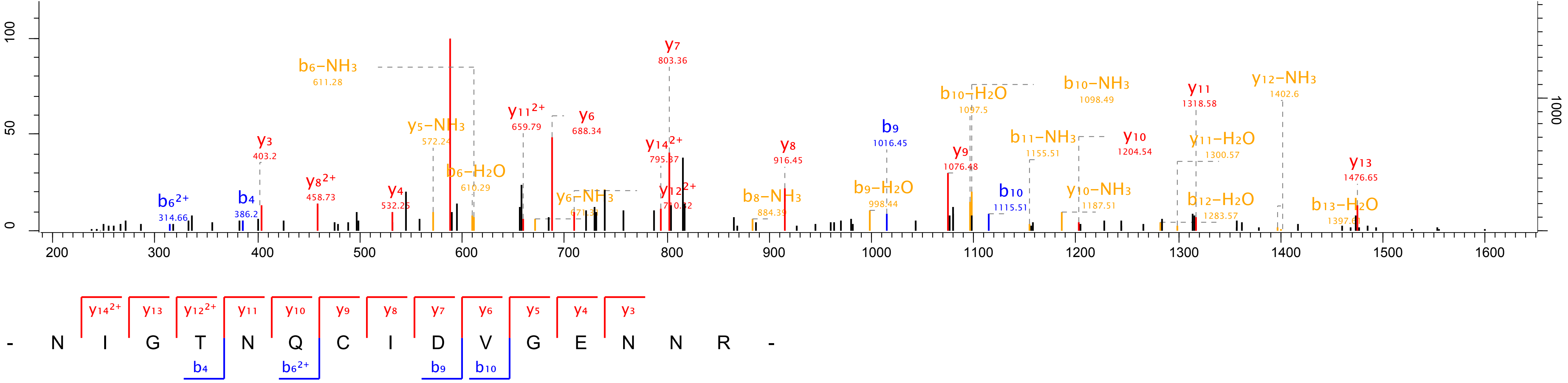

Raw file	Scan	Method	Score	m/z	Gene names
HBT_20130916_BV2_IC1_01	4359	ITMS; CID	142.97	730.81	Gorasp1

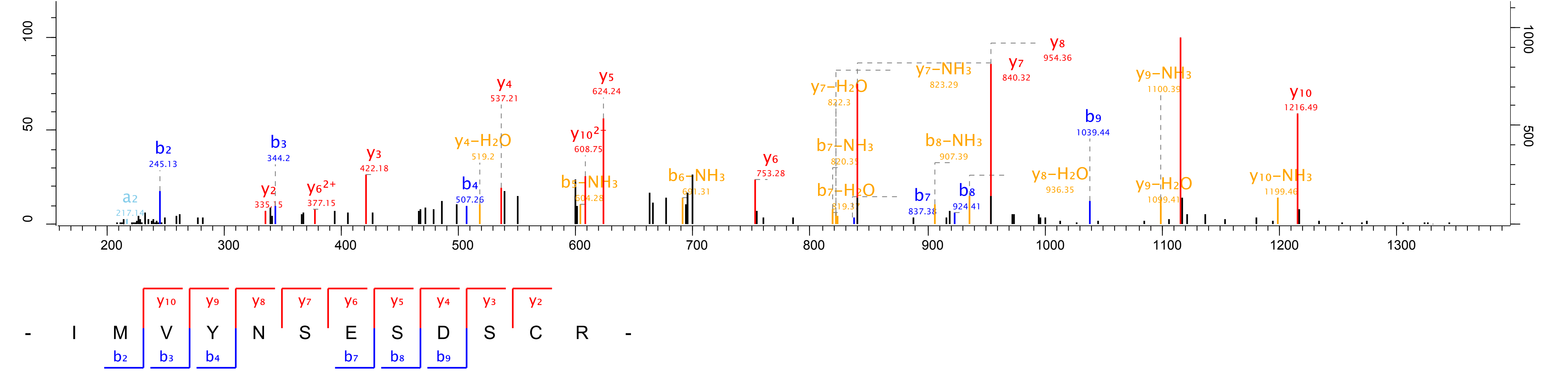

Raw file	Scan	Method	Score	m/z	Gene names
HBT_20130916_BV2_IC1_01	2273	ITMS; CID	98.1	568.31	Rbck1

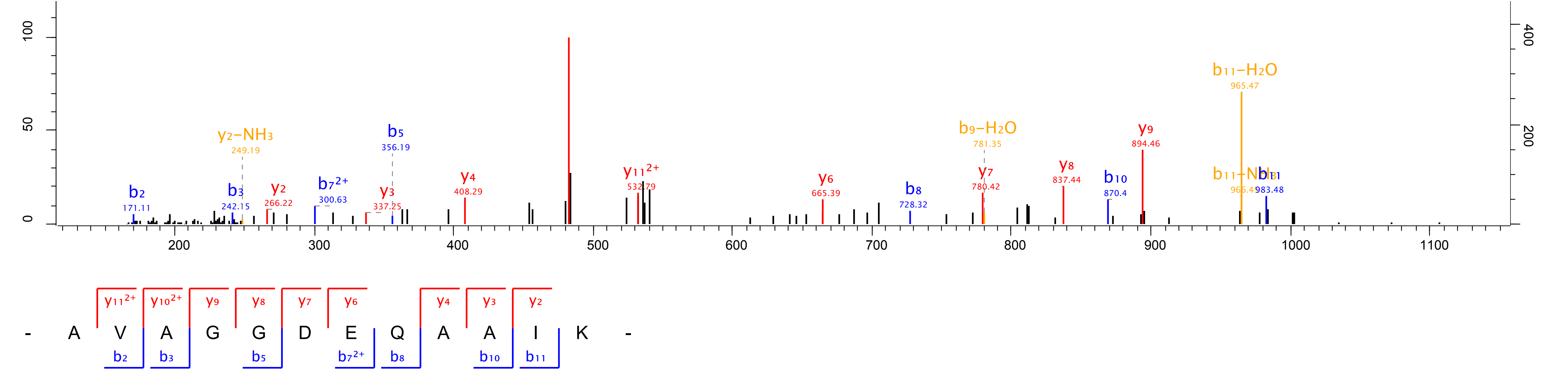

Raw file	Scan	Method	Score	m/z	Gene names
HBT_20130916_BV2_IC1_01	15813	ITMS; CID	100.11	694.89	Lamtor1

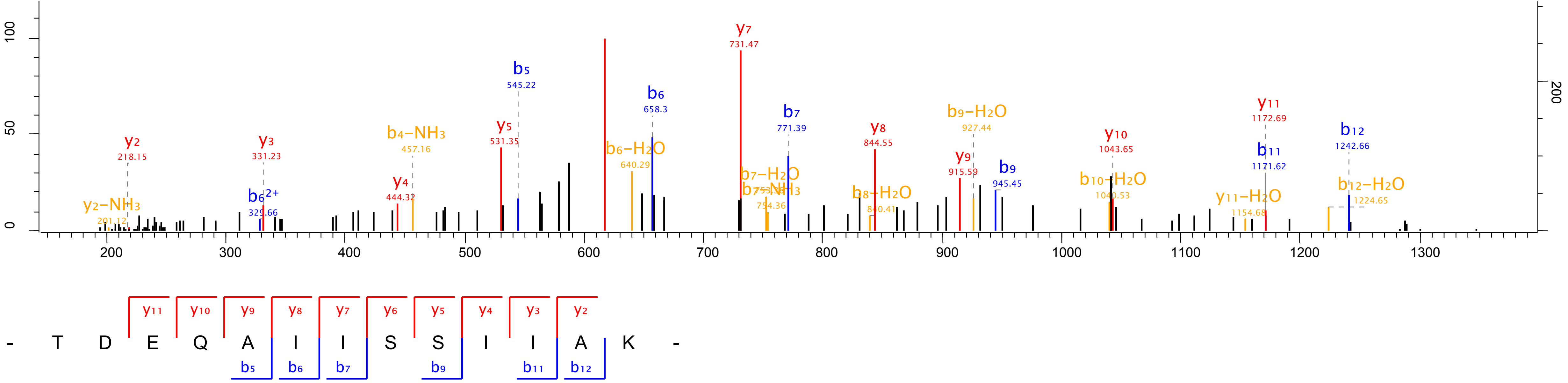

Raw file	Scan	Method	Score	m/z
HBT_20130723_BV2_LPS_3_006	4515	ITMS; CID	75.76	592.66

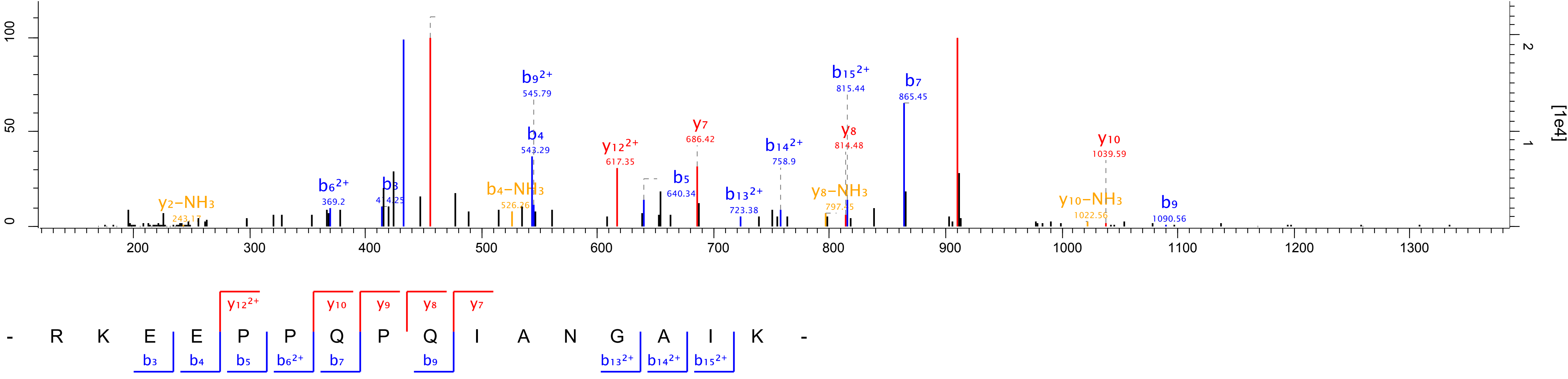

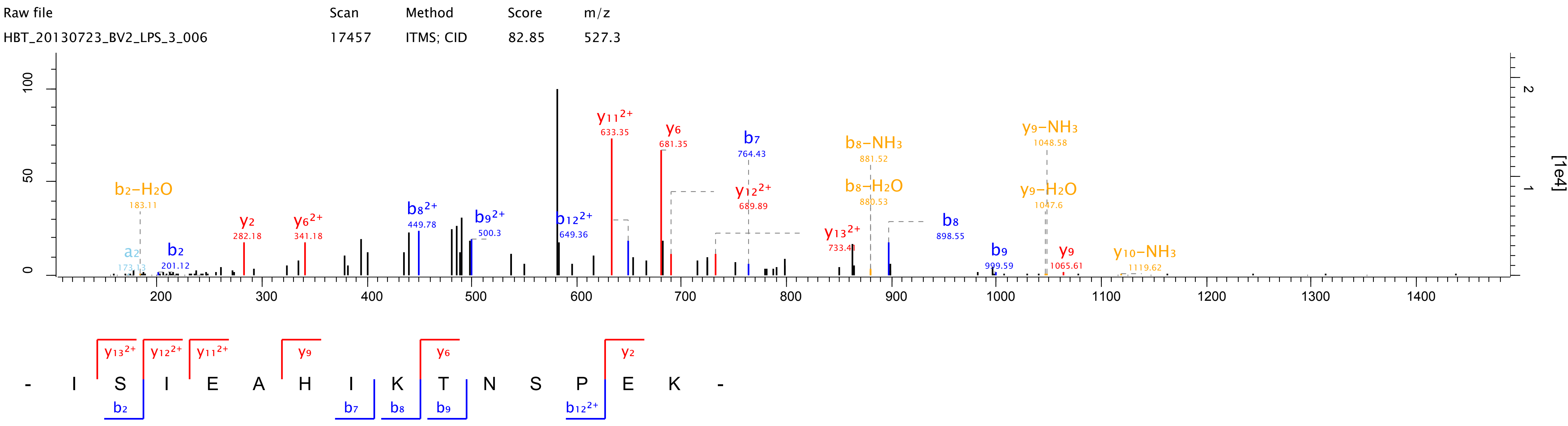

Raw file
HBT_20130723_BV2_LPS_3_006
Scan
17457
Method
ITMS; CID
Score
82.85
m/z
527.3
100
50
0
[1e4]
2
1
200
300
400
500
600
700
800
900
1000
1100
1200
1300
1400
b2-H2O 183.11
a2 173.13
b2 201.12
y2 282.18
y6 2+ 341.18
b8 2+ 449.78
b9 2+ 500.3
b12 2+ 649.36
y11 2+ 633.35
y6 681.35
b7 764.43
y12 2+ 689.89
y13 2+ 733.41
b8-NH3 881.52
b8-H2O 880.53
b8 898.55
b9 999.59
y9-NH3 1048.58
y9-H2O 1047.6
y9 1065.61
y10-NH3 1119.62
- I S I E A H I K T N S P E K -
y13 2+
y12 2+
y11 2+
y9
y6
y2
b2
b7
b8
b9
b12 2+

Raw file	Scan	Method	Score	m/z	Gene names
HBT_20130723_BV2_LPS_3_006	11295	ITMS; CID	47.08	611.06	Tcf20

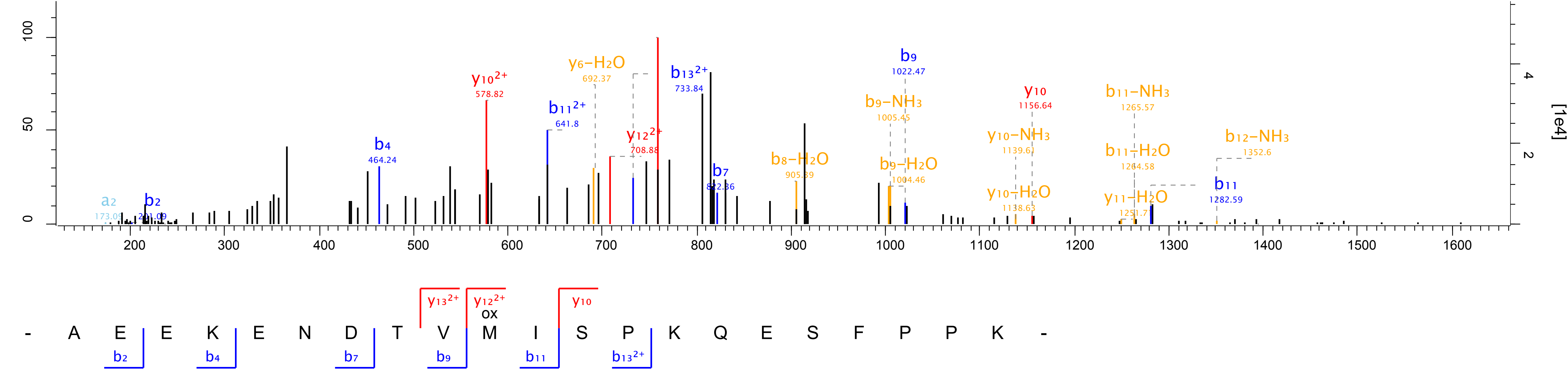

Raw file	Scan	Method	Score	m/z	Gene names
HBT_20130723_BV2_LPS_3_005	8369	ITMS; CID	100.02	402.23	Mpp2

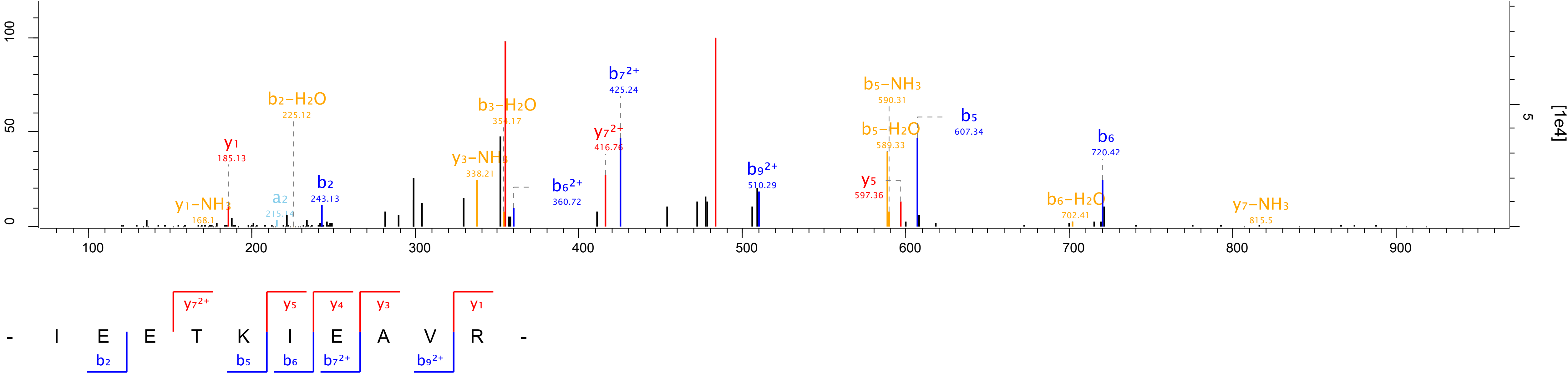

Raw file	Scan	Method	Score	m/z	Gene names
HBT_20130723_BV2_LPS_3_004	29507	ITMS; CID	62.08	724.88	Gen1

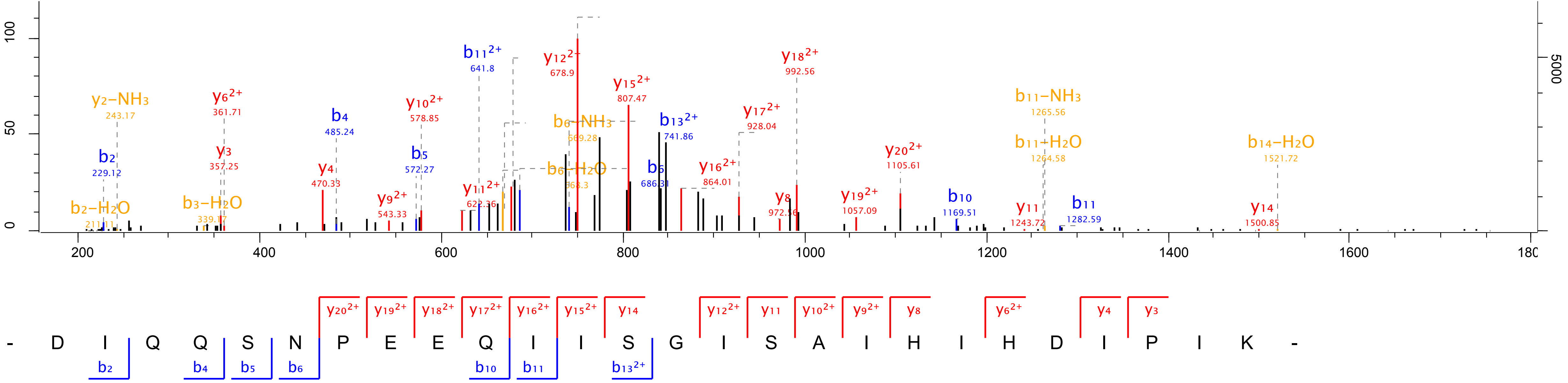

Raw file	Scan	Method	Score	m/z
HBT_20130723_BV2_LPS_3_004	2862	ITMS; CID	85.35	367.54

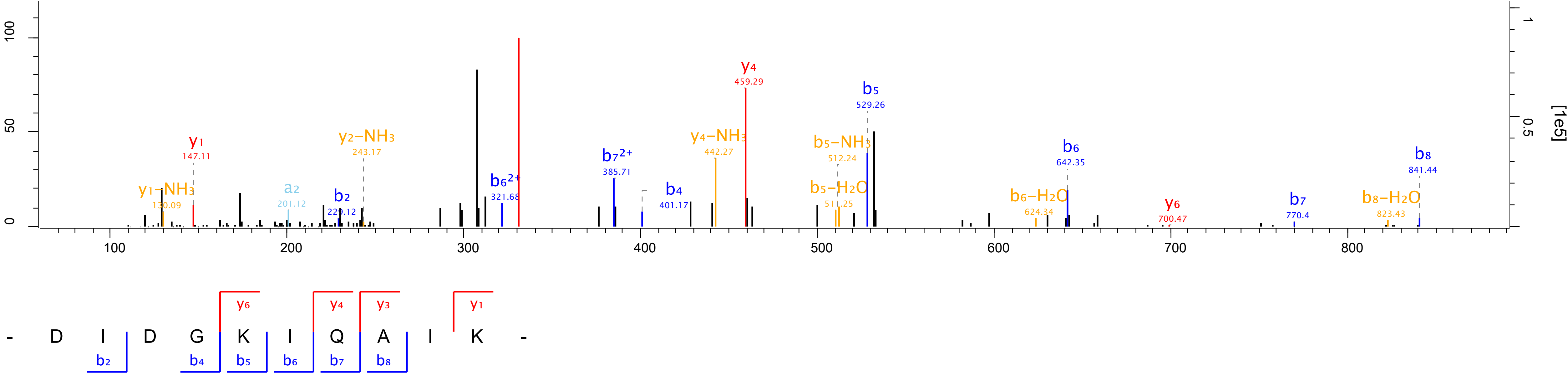

Raw file	Scan	Method	Score	m/z
HBT_20130723_BV2_LPS_3_004	2682	ITMS; CID	51.13	544.94

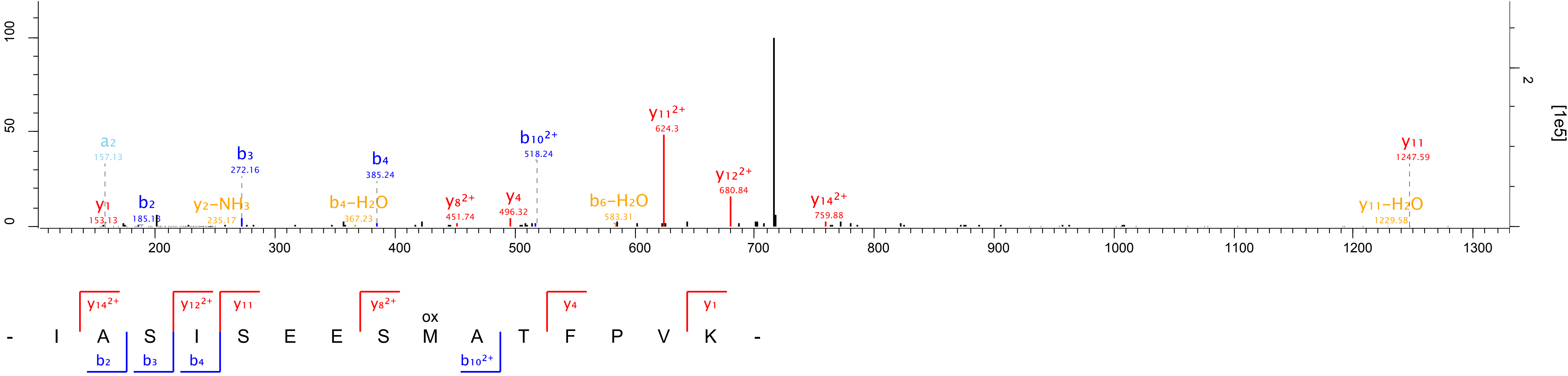

Raw file	Scan	Method	Score	m/z	Gene names
HBT_20130723_BV2_LPS_3_004	20795	ITMS; CID	68.68	565.96	Zranb3

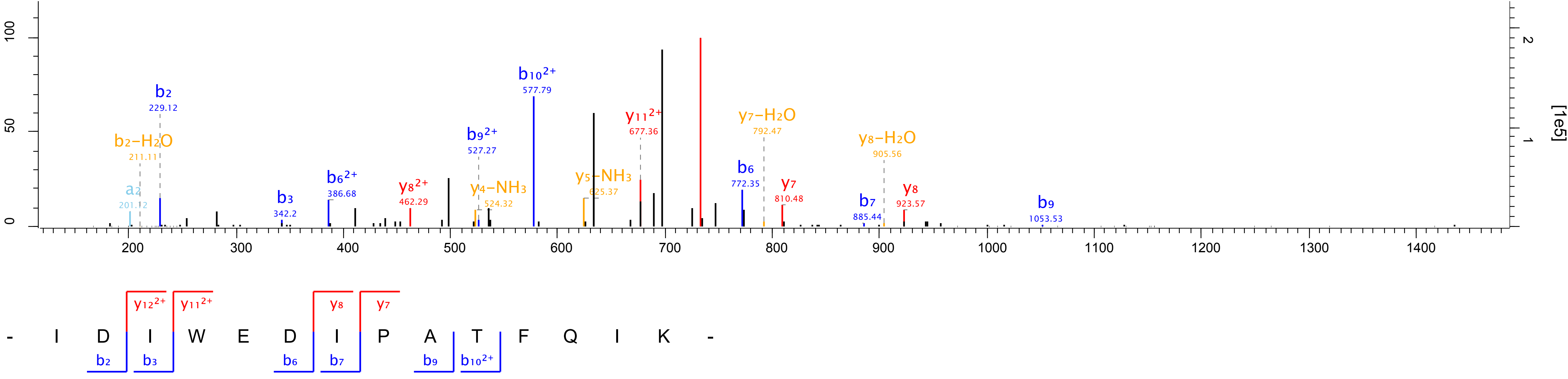

Raw file	Scan	Method	Score	m/z	Gene names
HBT_20130723_BV2_LPS_3_004	16952	ITMS; CID	110.57	852.1	Mmgt1

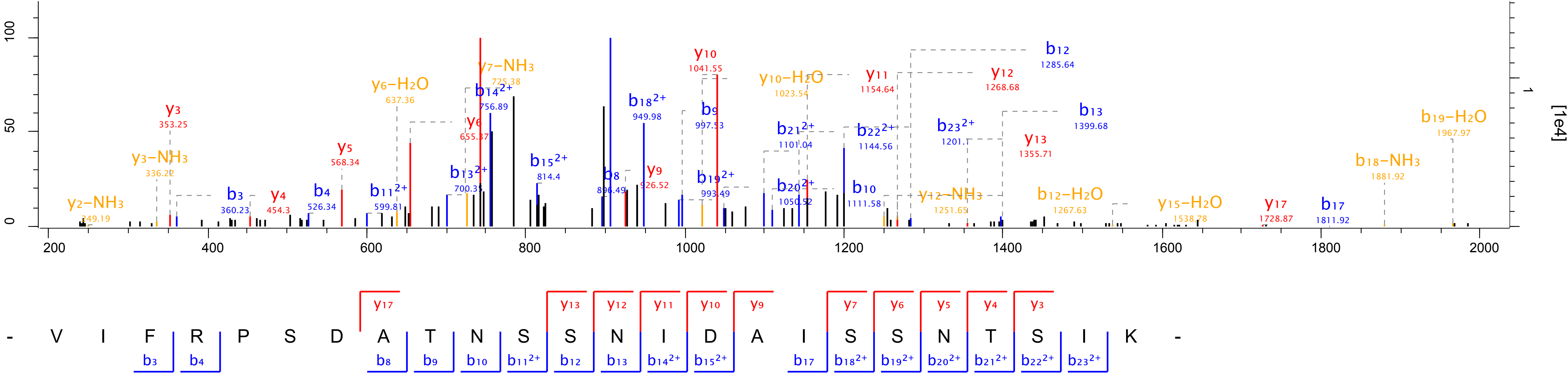

Raw file	Scan	Method	Score	m/z	Gene names
HBT_20130723_BV2_LPS_3_004	11530	ITMS; CID	60.49	688.05	Zfp397

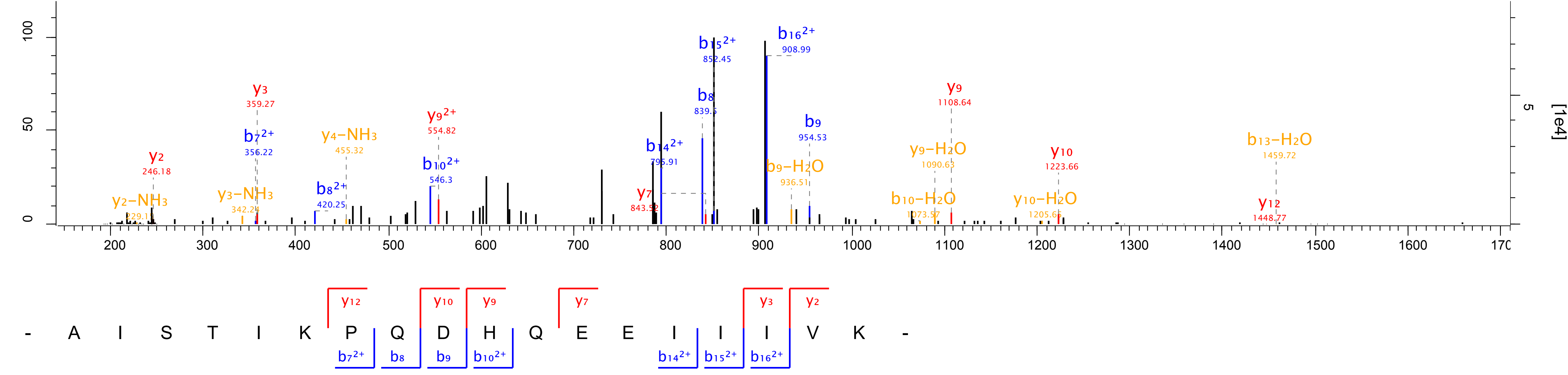

Raw file	Scan	Method	Score	m/z	Gene names
HBT_20130723_BV2_LPS_3_004	1092	ITMS; CID	183.6	793.99	Yaf2

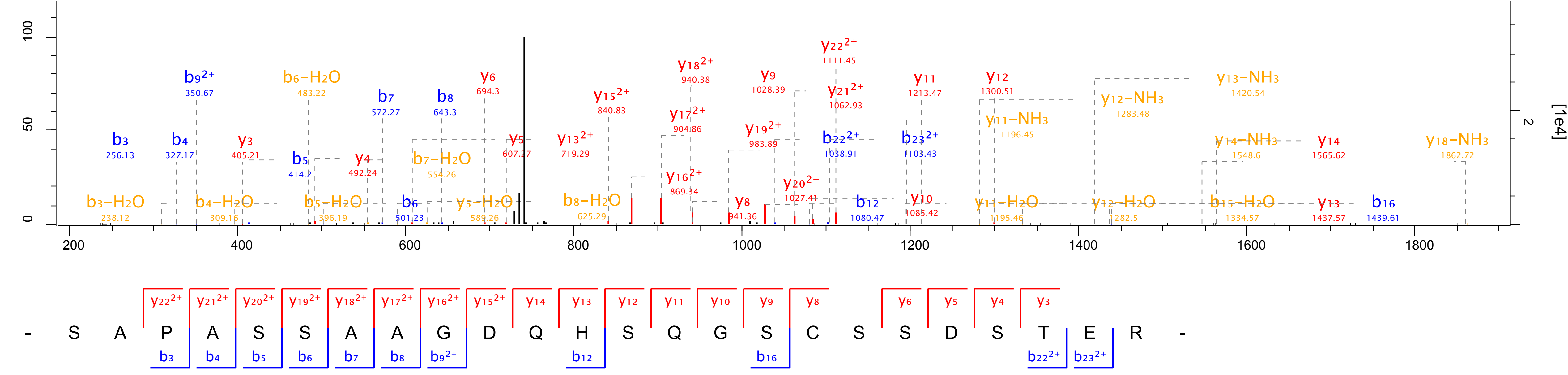

Raw file	Scan	Method	Score	m/z	Gene names
HBT_20130723_BV2_LPS_3_003	34665	ITMS; CID	100	1262.66	Ubac1

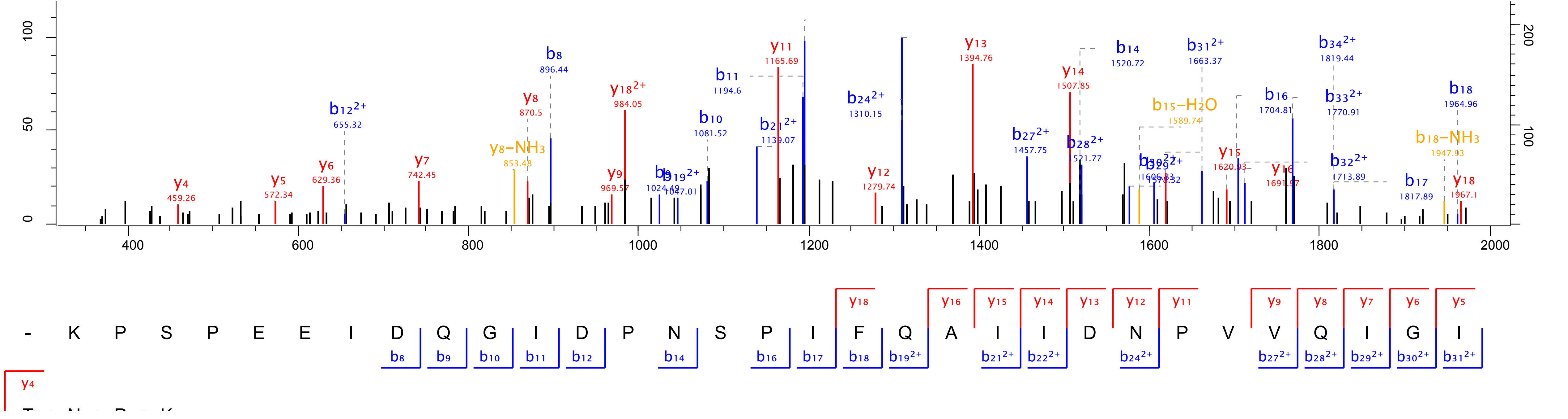

Raw file	Scan	Method	Score	m/z	Gene names
HBT_20130723_BV2_LPS_3_003	32501	ITMS; CID	85.88	945.45	Dus4l

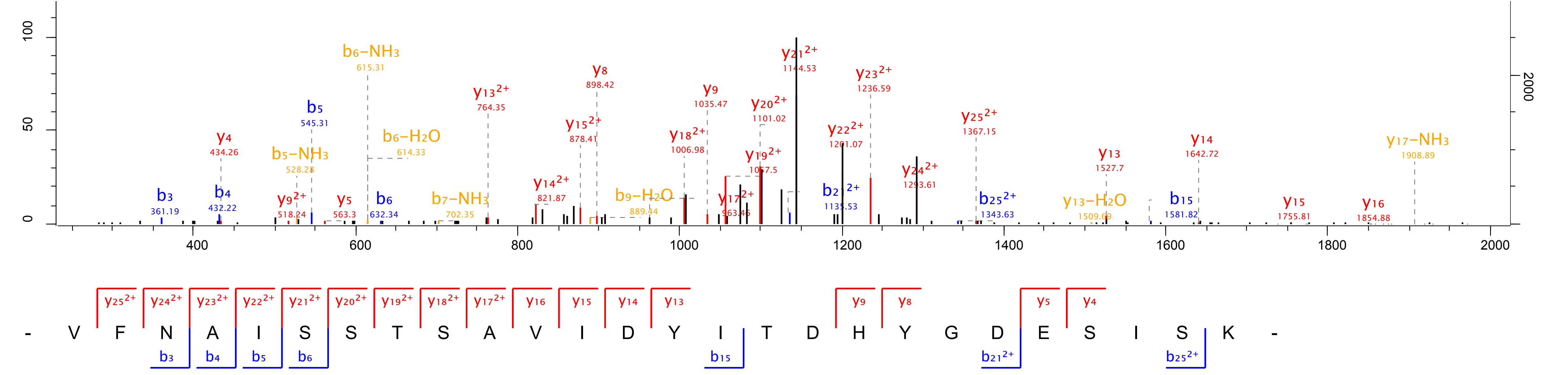

Raw file	Scan	Method	Score	m/z	Gene names
HBT_20130723_BV2_LPS_3_003	32413	ITMS; CID	109.91	997.85	Entpd5

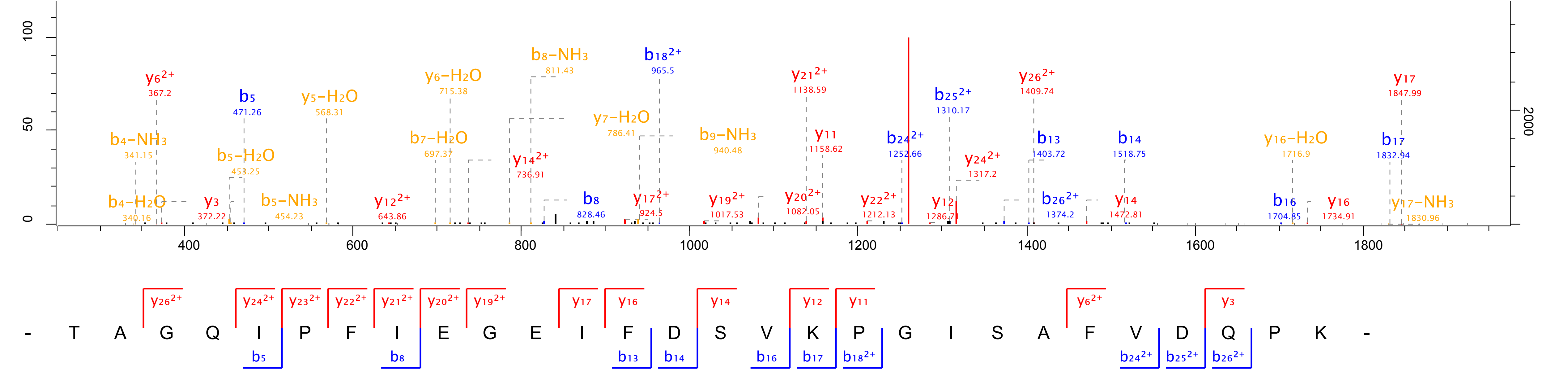

Raw file	Scan	Method	Score	m/z
HBT_20130723_BV2_LPS_3_003	32085	ITMS; CID	128.33	986.53

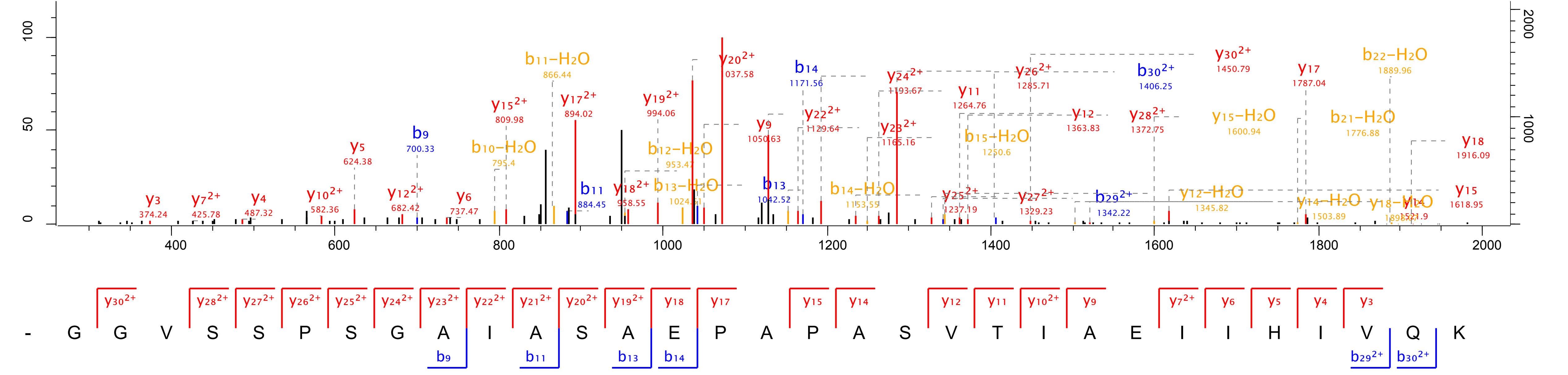

Raw file	Scan	Method	Score	m/z	Gene names
HBT_20130723_BV2_LPS_3_003	26103	ITMS; CID	130.57	1161.9	Mrpl52

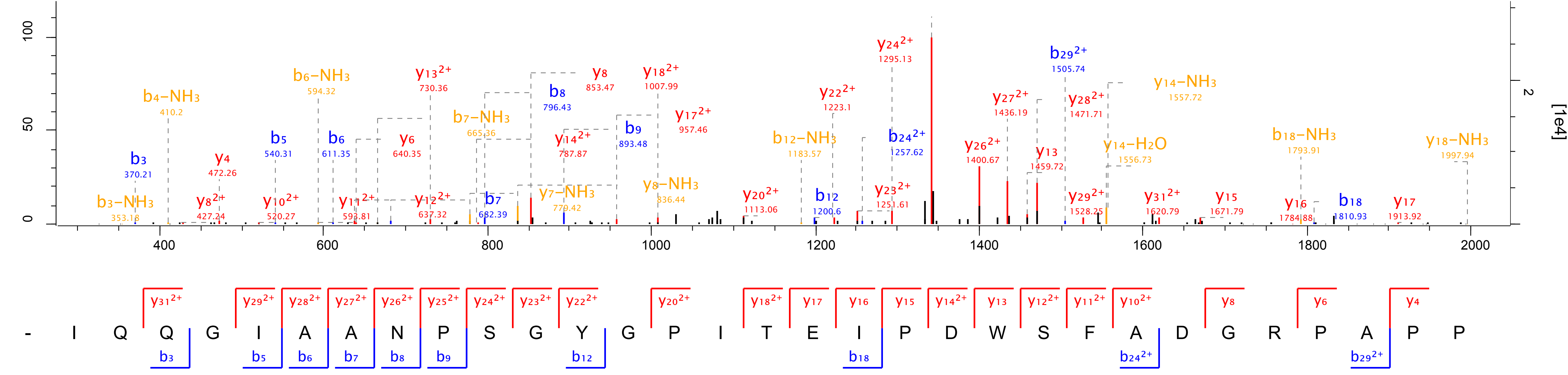

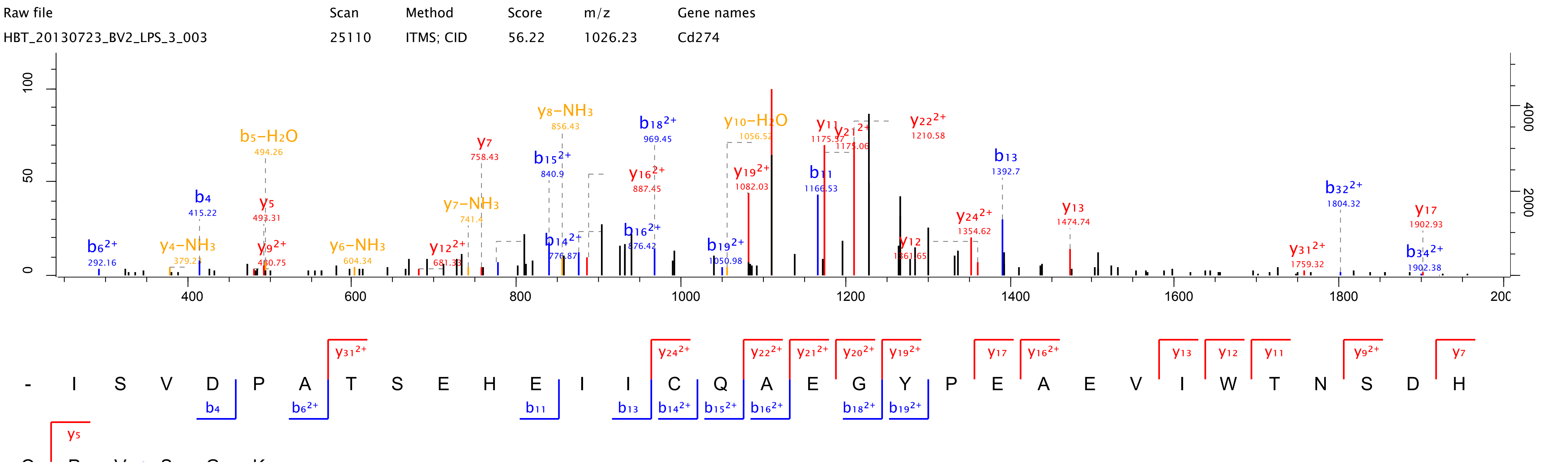

Raw file
HBT_20130723_BV2_LPS_3_003
Scan
25110
Method
ITMS; CID
Score
56.22
m/z
1026.23
Gene names
Cd274
100
50
0
4000
2000
400
600
800
1000
1200
1400
1600
1800
- I S V D P A T S E H E I I C Q A E G Y P E A E V I W T N S D H
Q R V S G K

Raw file	Scan	Method	Score	m/z	Gene names
HBT_20130723_BV2_LPS_3_003	14415	ITMS; CID	86.92	991.84	Ndufv3

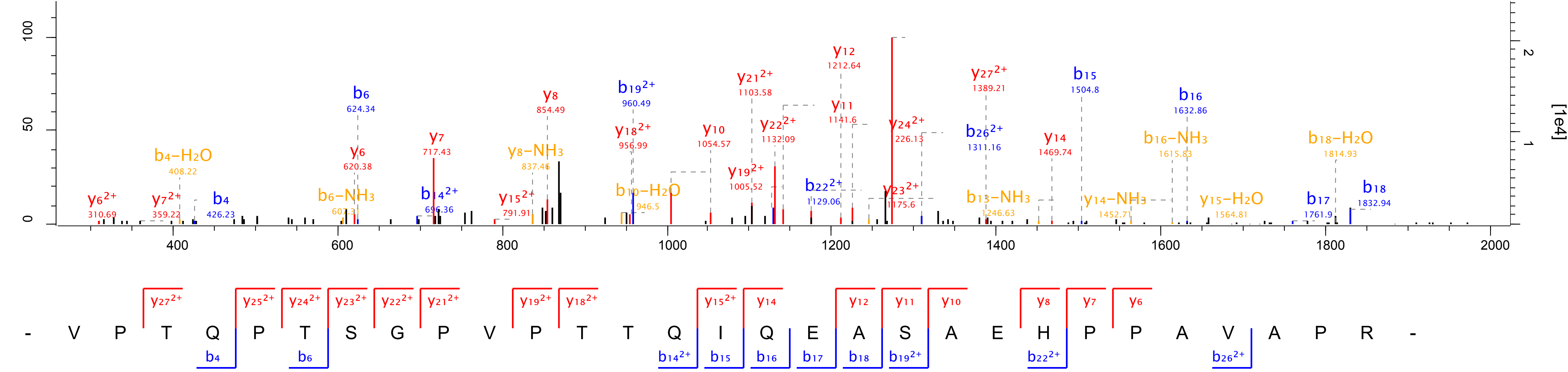

Raw file	Scan	Method	Score	m/z	Gene names
HBT_20130723_BV2_LPS_3_003	1240	ITMS; CID	113.25	591.91	Zdhhc6

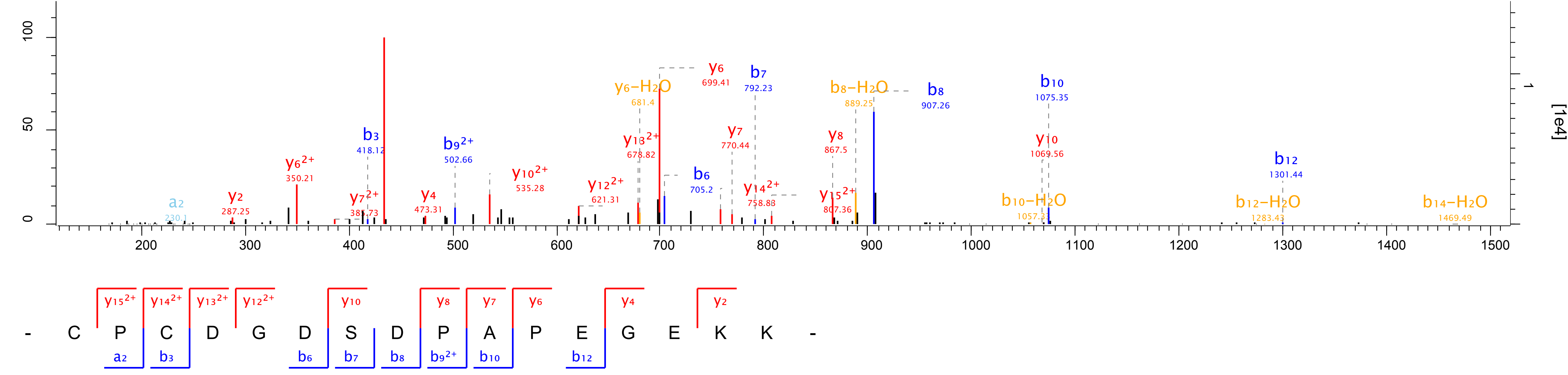

Raw file	Scan	Method	Score	m/z	Gene names
HBT_20130723_BV2_LPS_3_003	10700	ITMS; CID	172.88	1258.87	Hvcn1

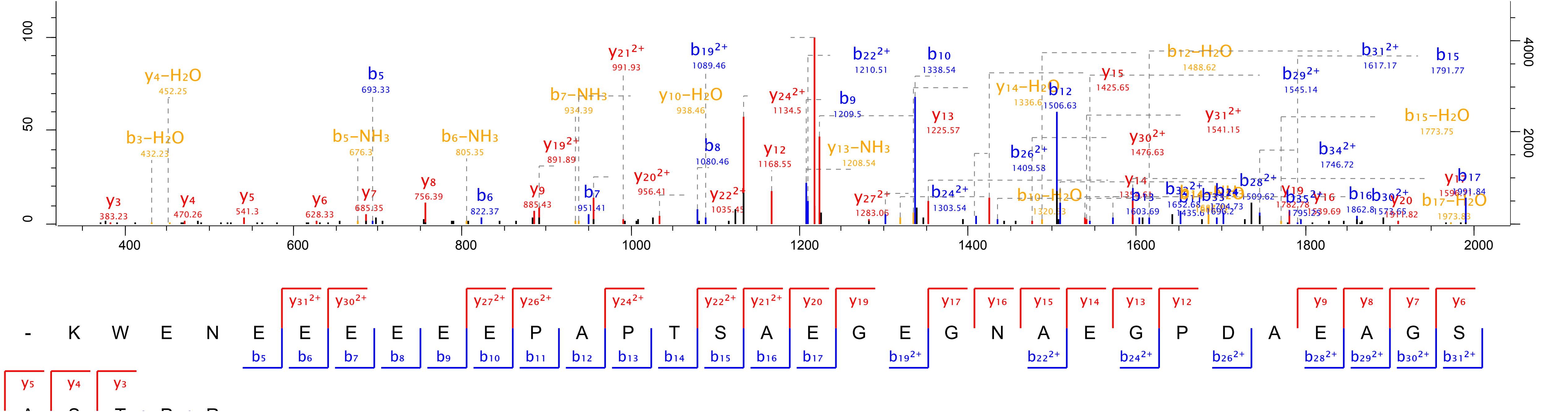

Raw file	Scan	Method	Score	m/z	Gene names
HBT_20130723_BV2_LPS_3_002	34259	ITMS; CID	54	1275.68	Fbxo7

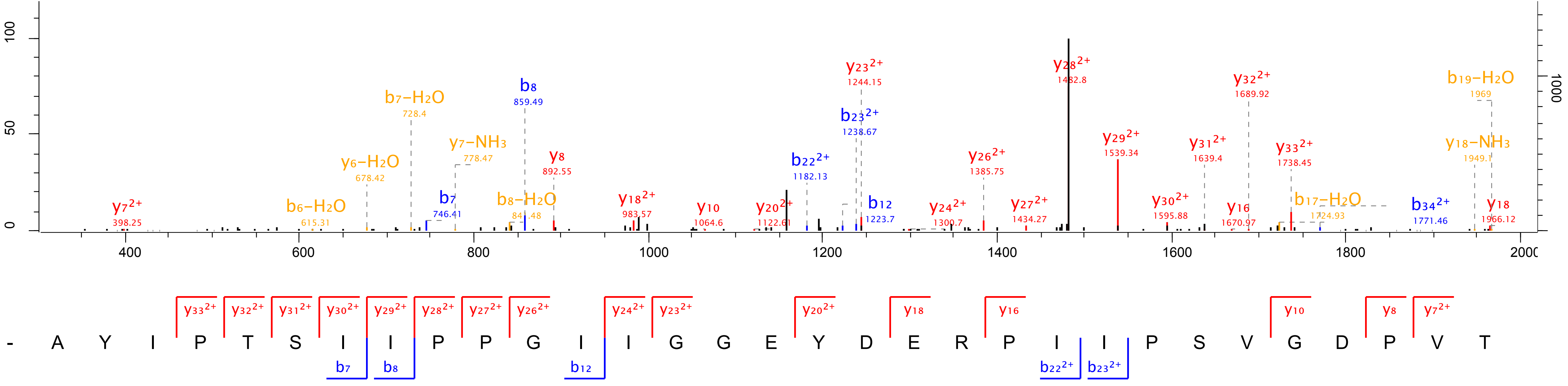

Raw file	Scan	Method	Score	m/z	Gene names
HBT_20130723_BV2_LPS_3_002	32270	ITMS; CID	120.93	1133.89	Bmp2k

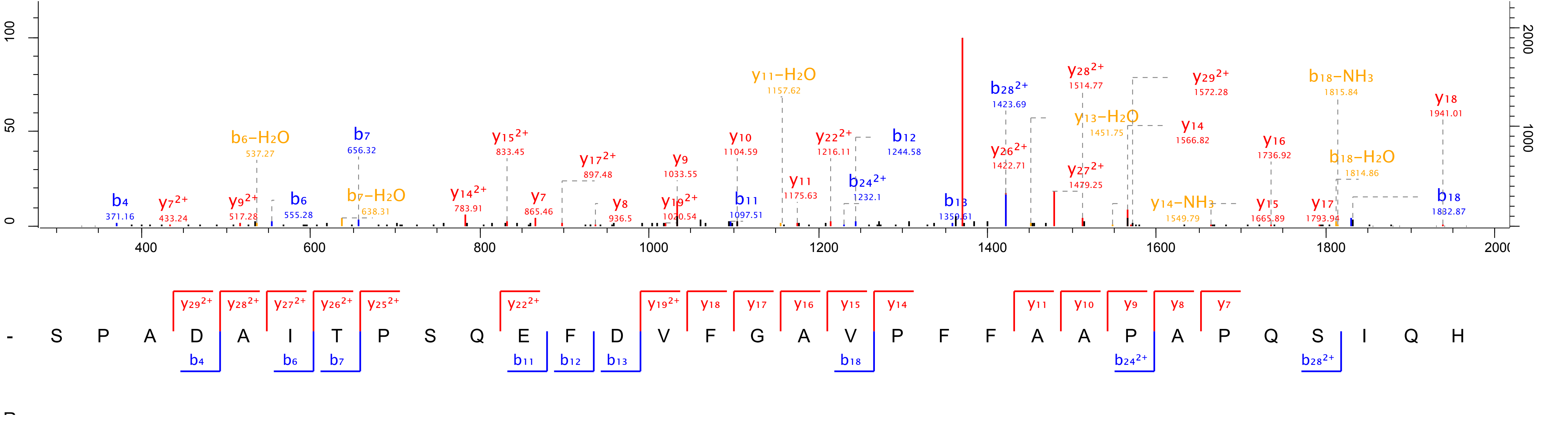

Raw file	Scan	Method	Score	m/z	Gene names
HBT_20130723_BV2_LPS_3_002	25896	ITMS; CID	46.4	978.17	Abcd2

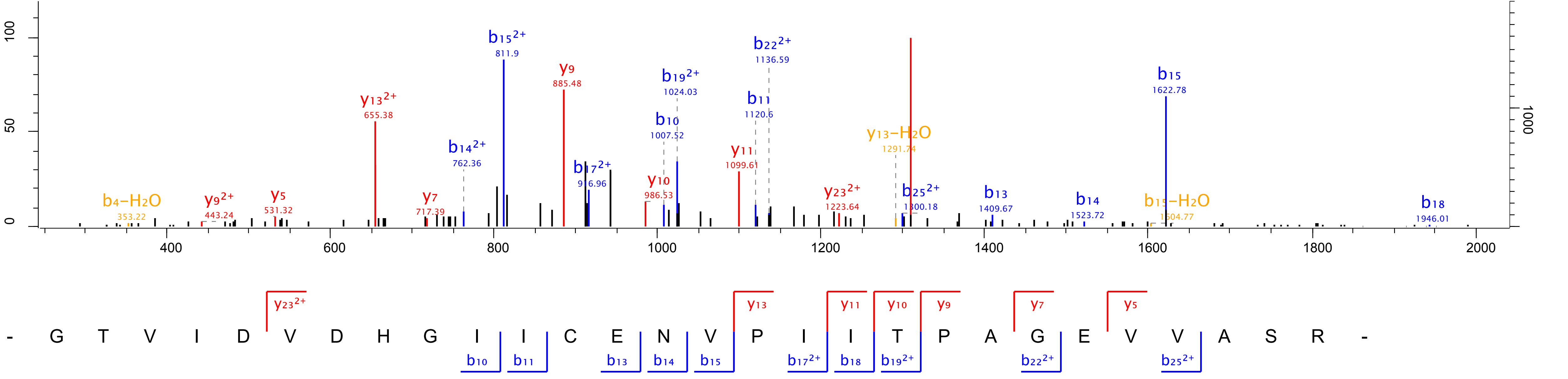

Raw file	Scan	Method	Score	m/z	Gene names
HBT_20130723_BV2_LPS_3_002	25660	ITMS; CID	94.84	1068.84	Mtmr1

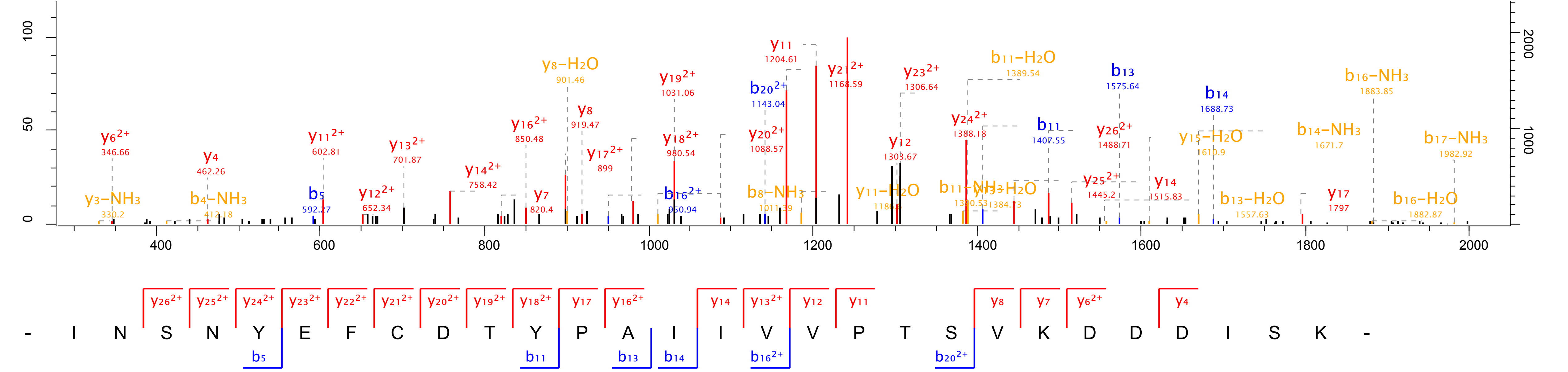

Raw file	Scan	Method	Score	m/z	Gene names
HBT_20130723_BV2_LPS_3_002	25549	ITMS; CID	70.96	891.45	Ercc2

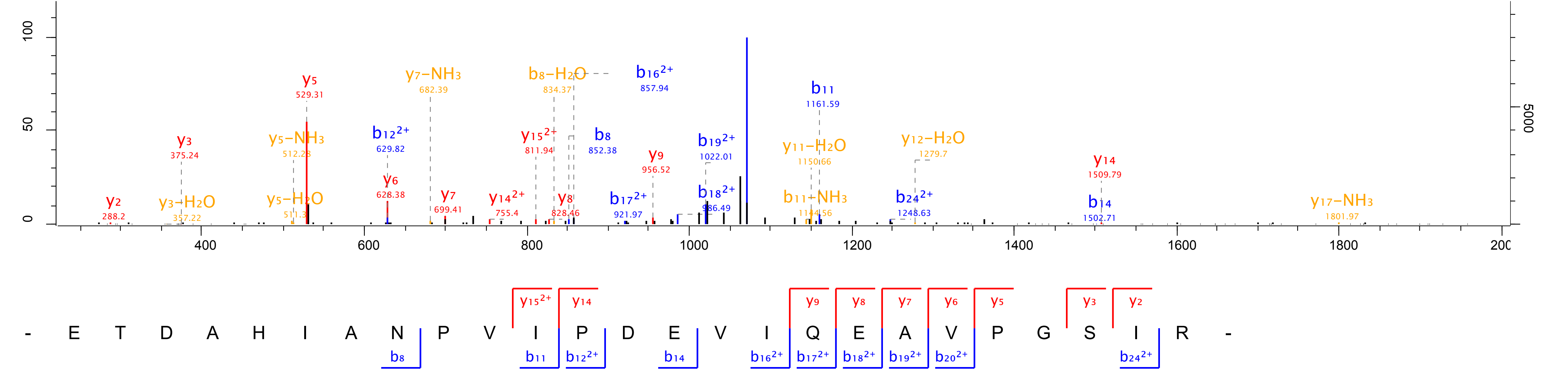

Raw file	Scan	Method	Score	m/z	Gene names
HBT_20130723_BV2_LPS_3_002	24687	ITMS; CID	119.52	1142.85	Derl2

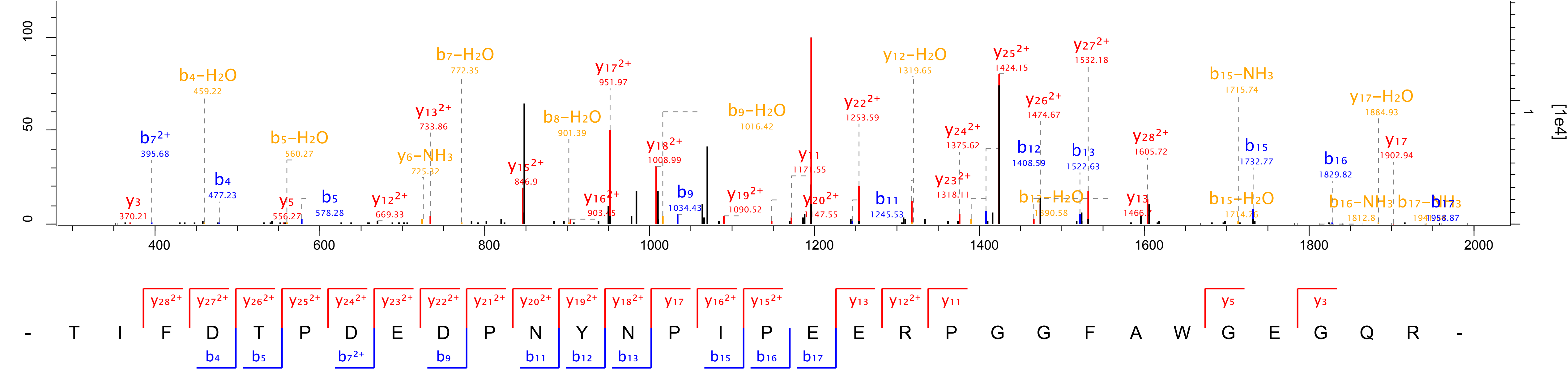

Raw file	Scan	Method	Score	m/z	Gene names
HBT_20130723_BV2_LPS_3_002	21926	ITMS; CID	47.75	874.41	Slc27a1

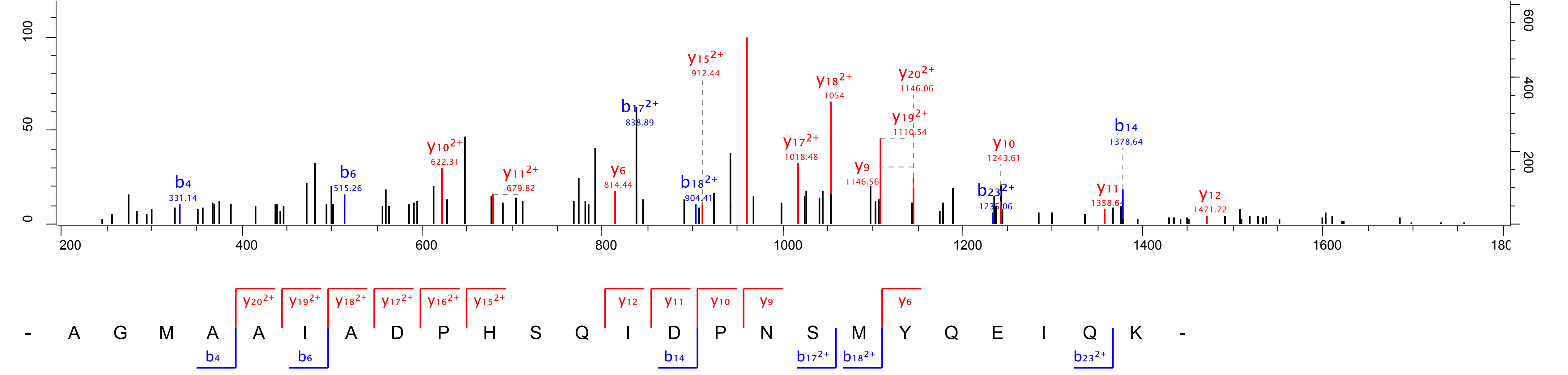

Raw file	Scan	Method	Score	m/z	Gene names
HBT_20130723_BV2_LPS_3_002	19703	ITMS; CID	122.59	1063.48	Scimp

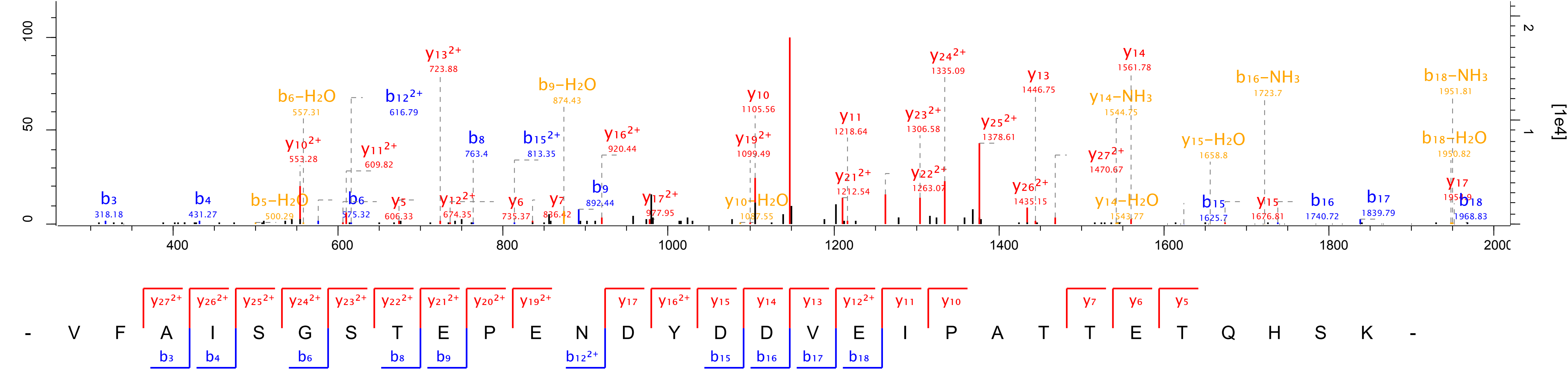

Raw file	Scan	Method	Score	m/z	Gene names
HBT_20130723_BV2_LPS_3_002	19322	ITMS; CID	69.05	893.94	Nxpe3

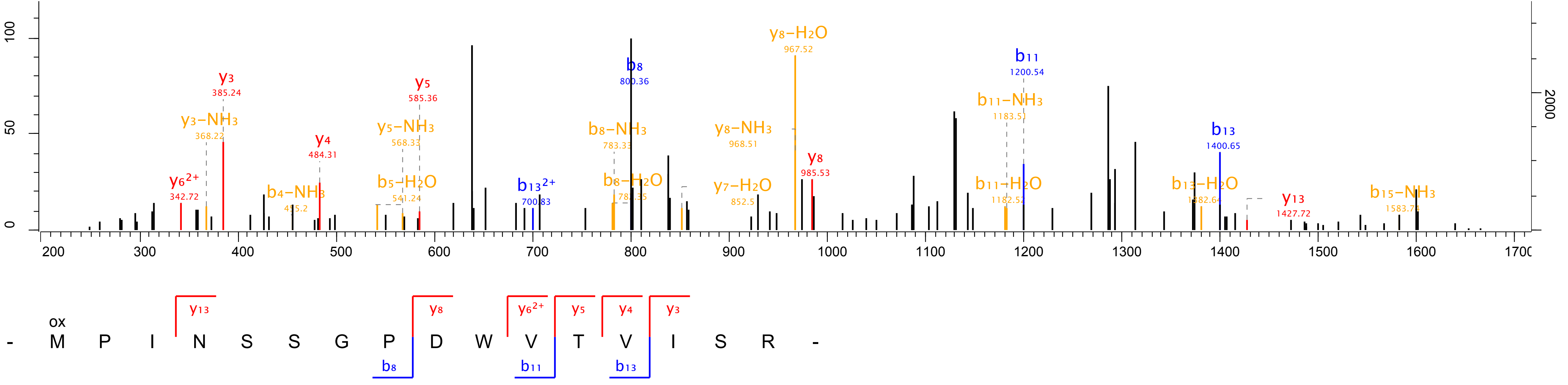

Raw file	Scan	Method	Score	m/z	Gene names
HBT_20130723_BV2_LPS_3_002	17141	ITMS; CID	190.34	846.9	Ube2h

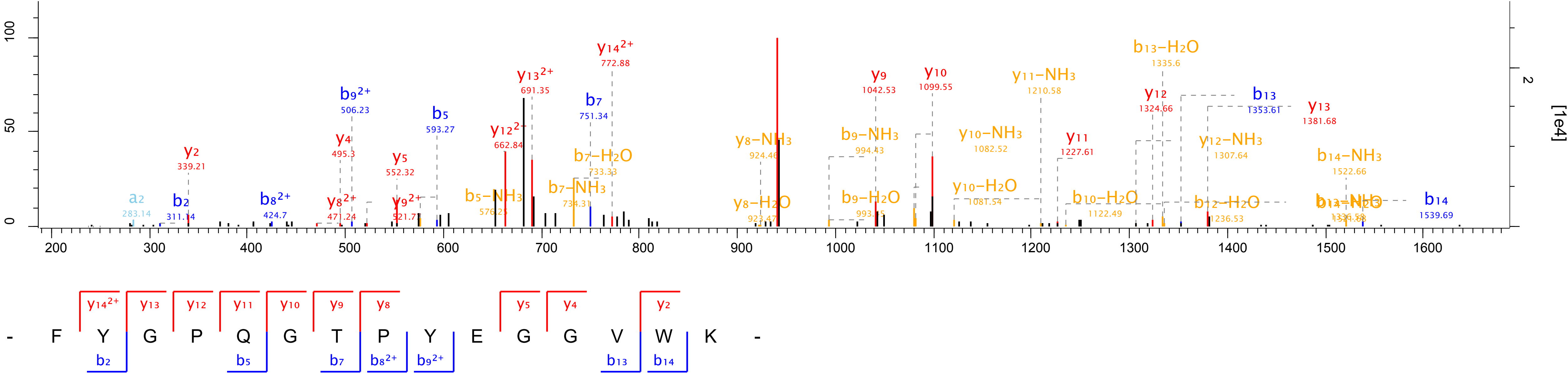

Raw file	Scan	Method	Score	m/z	Gene names
HBT_20130723_BV2_LPS_3_002	17033	ITMS; CID	166.64	884.91	Ly6e

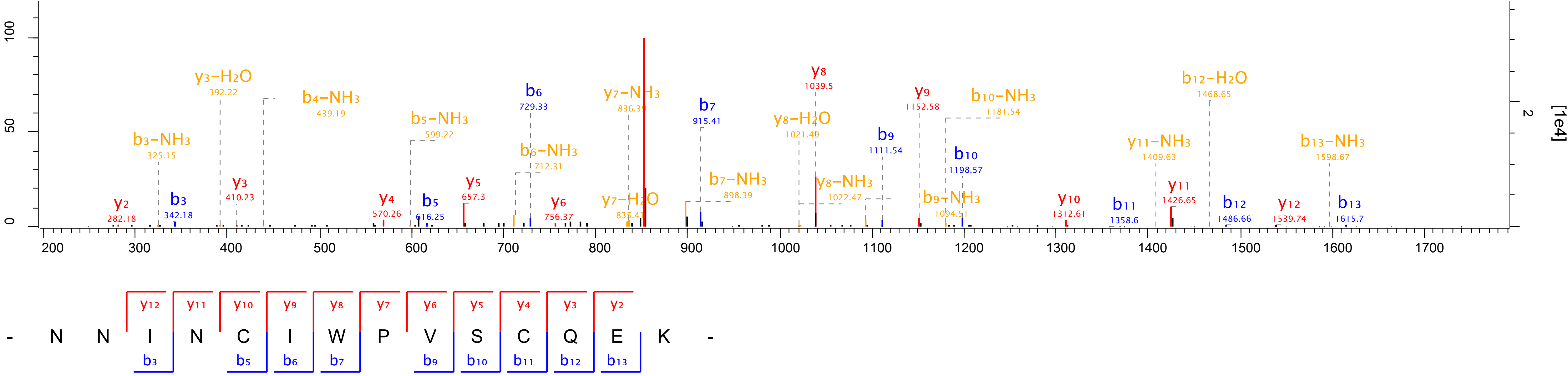

Raw file	Scan	Method	Score	m/z	Gene names
HBT_20130723_BV2_LPS_3_002	15568	ITMS; CID	107.07	920.43	Ccsmst1

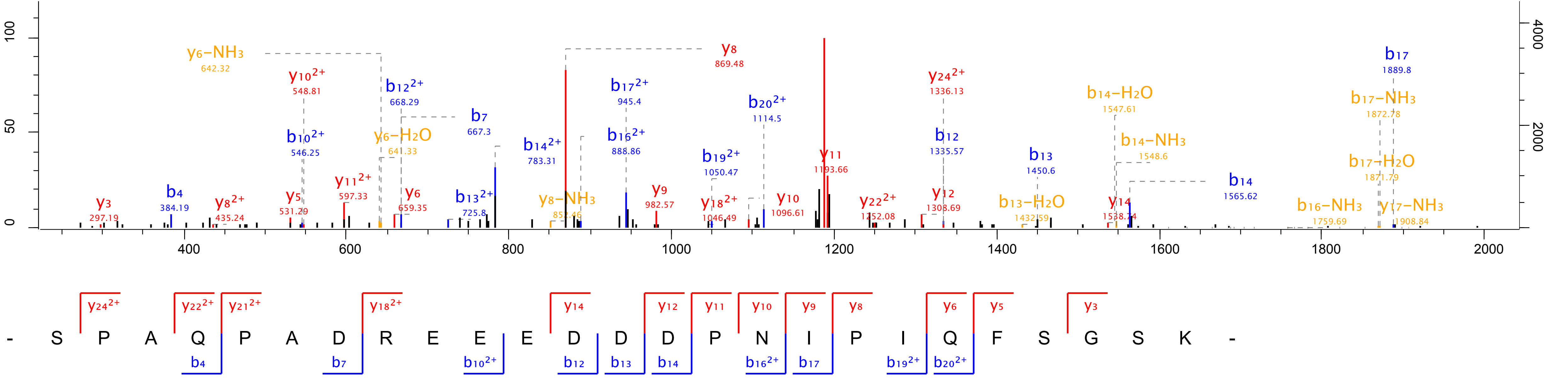

Raw file	Scan	Method	Score	m/z	Gene names
HBT_20130723_BV2_LPS_3_002	1539	ITMS; CID	84.35	638.77	Eif4ebp1

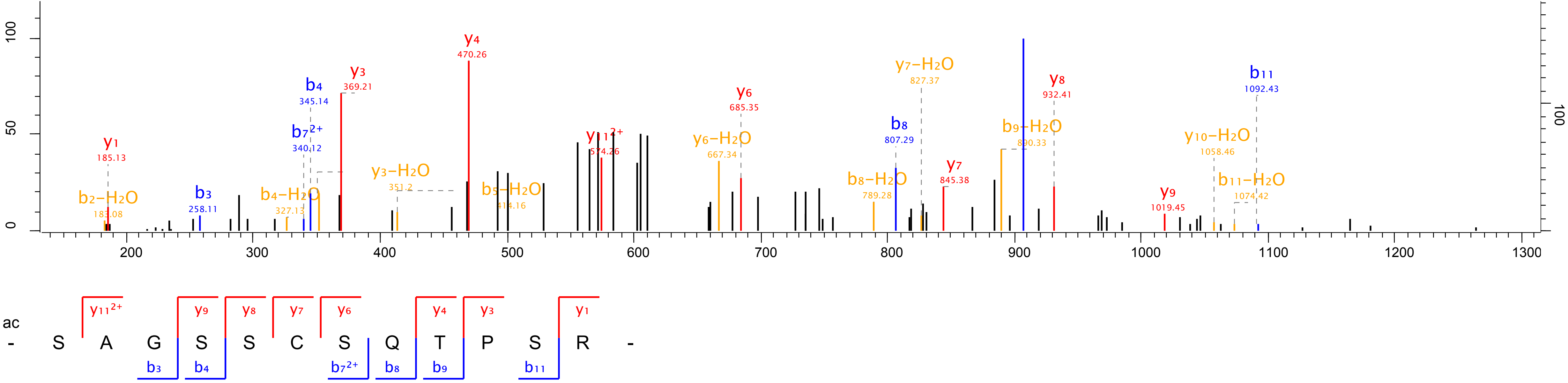

Raw file	Scan	Method	Score	m/z	Gene names
HBT_20130723_BV2_LPS_3_002	15010	ITMS; CID	128.6	526.82	Wnt10b

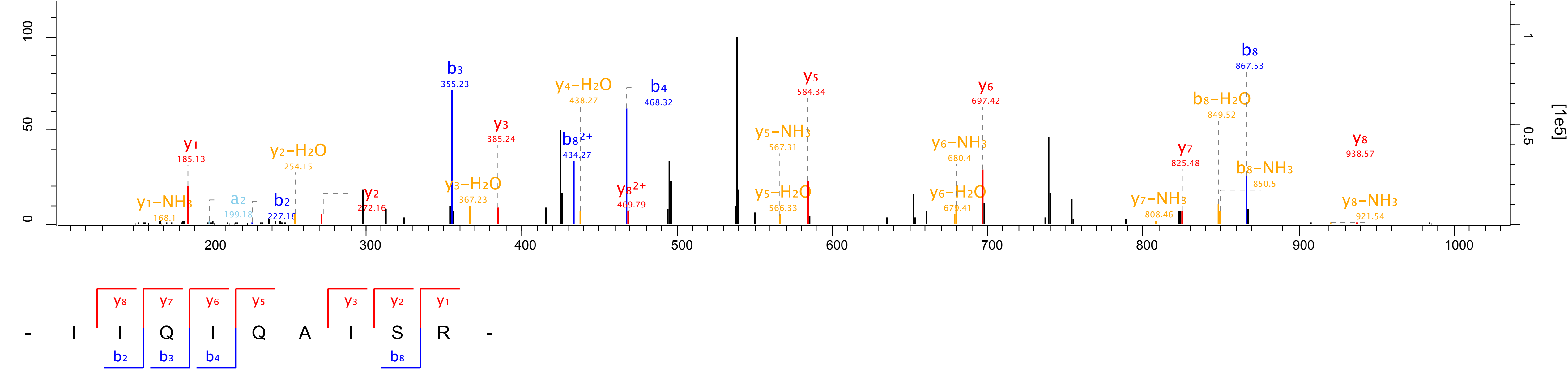

Raw file	Scan	Method	Score	m/z	Gene names
HBT_20130723_BV2_LPS_3_001	30980	ITMS; CID	86.74	883.91	Ssx9

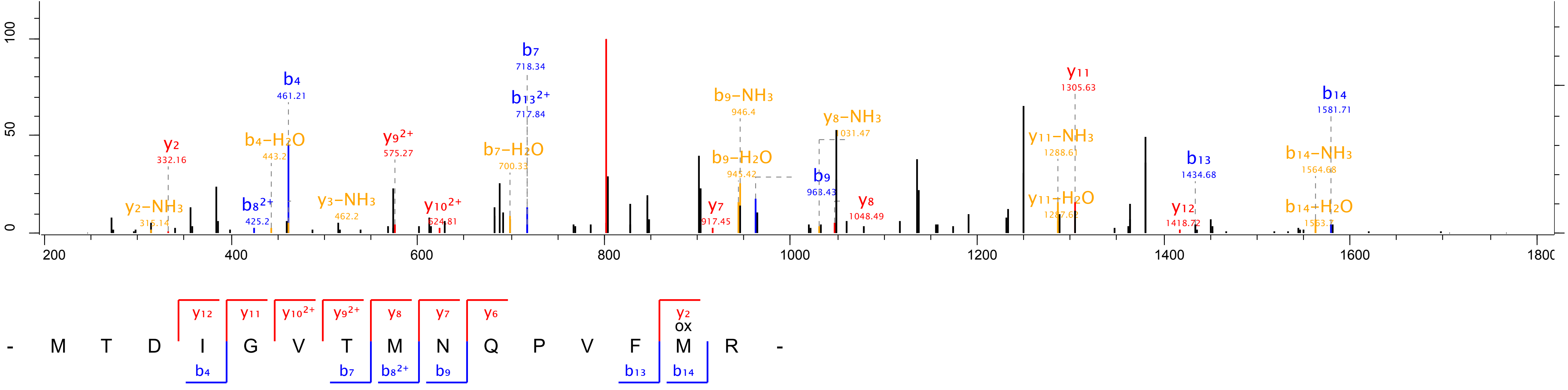

Raw file	Scan	Method	Score	m/z	Gene names
HBT_20130723_BV2_LPS_3_001	2839	ITMS; CID	102.06	523.27	Zc2hc1c

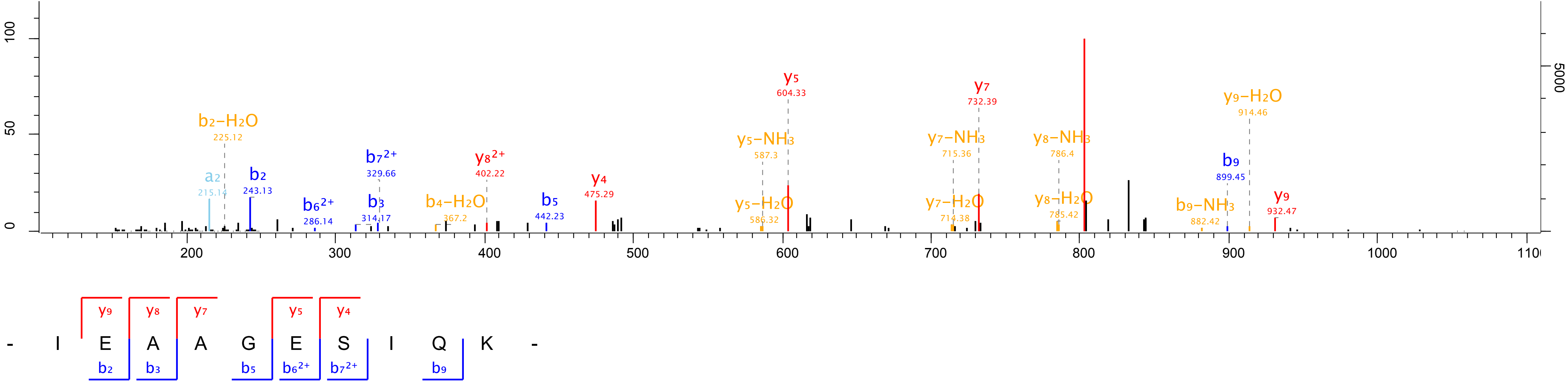

Raw file	Scan	Method	Score	m/z	Gene names
HBT_20130723_BV2_LPS_3_001	26764	ITMS; CID	167.9	995.09	Akt1s1

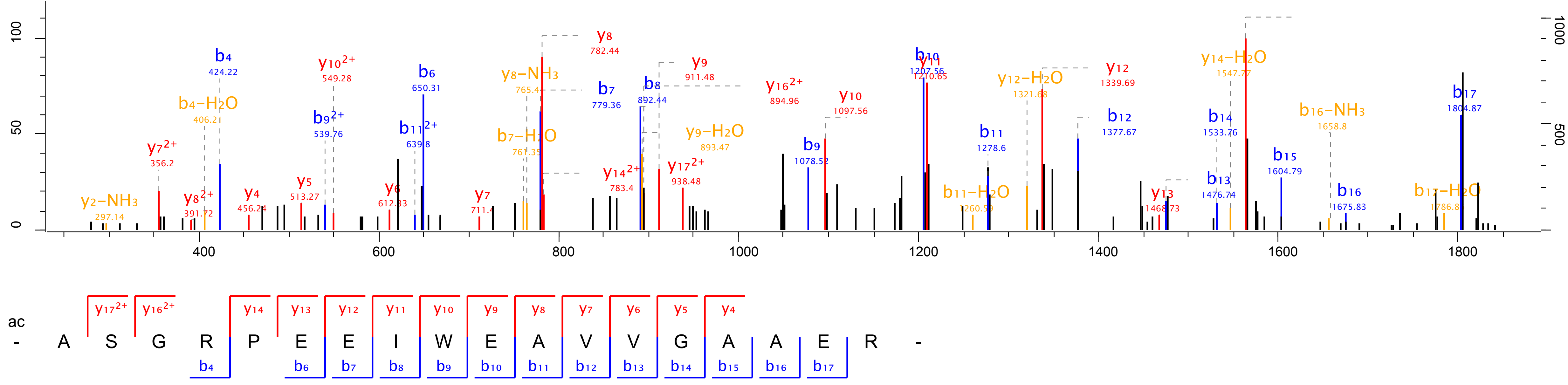

Raw file	Scan	Method	Score	m/z	Gene names
HBT_20130723_BV2_LPS_3_001	13358	ITMS; CID	158.17	902.43	Tmem205

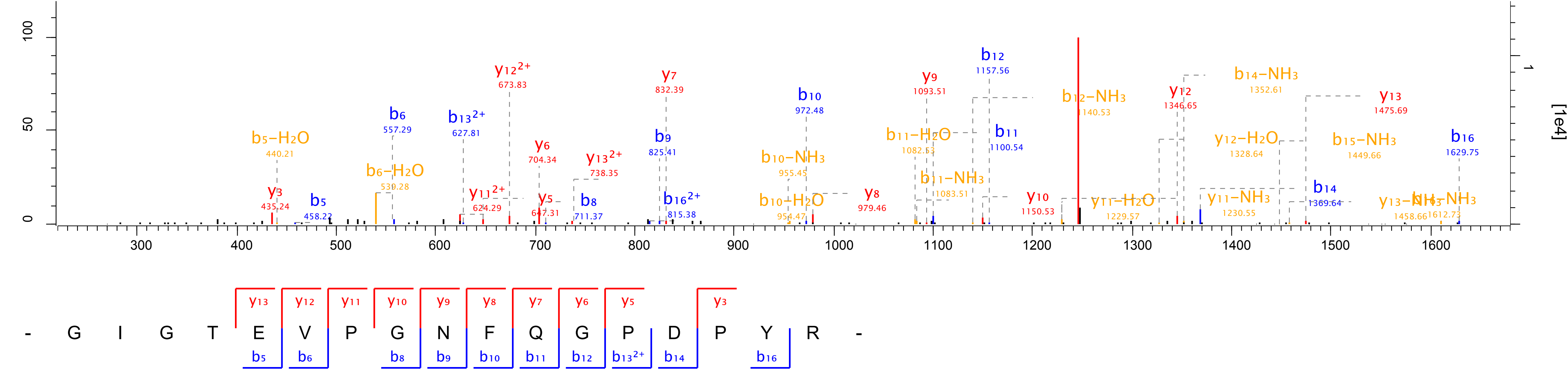

Raw file	Scan	Method	Score	m/z	Gene names
HBT_20130723_BV2_LPS_2_006	9812	ITMS; CID	146.69	654.36	Rpl39

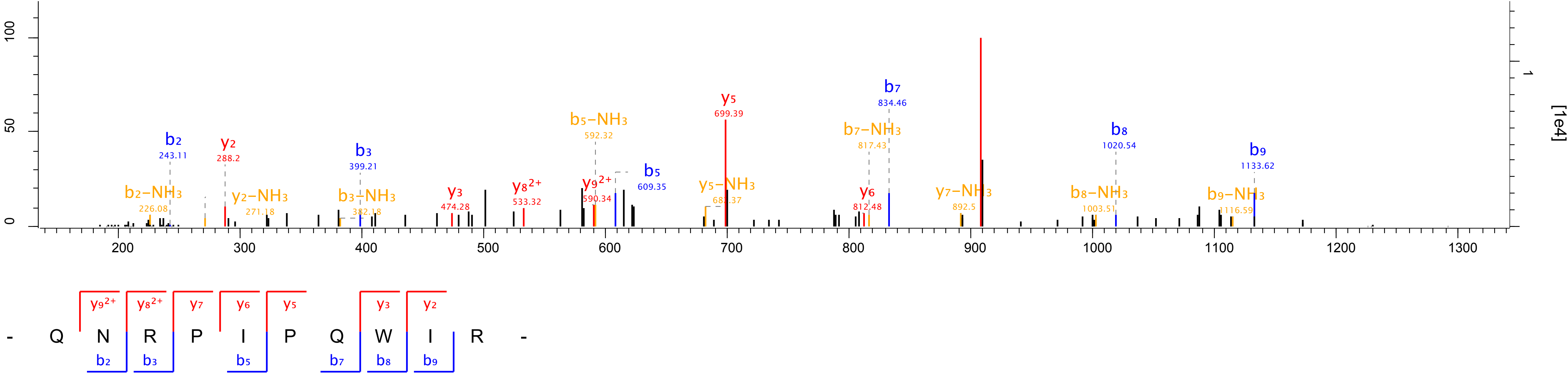

Raw file	Scan	Method	Score	m/z	Gene names
HBT_20130723_BV2_LPS_2_006	7540	ITMS; CID	67.25	471.59	Mrps14

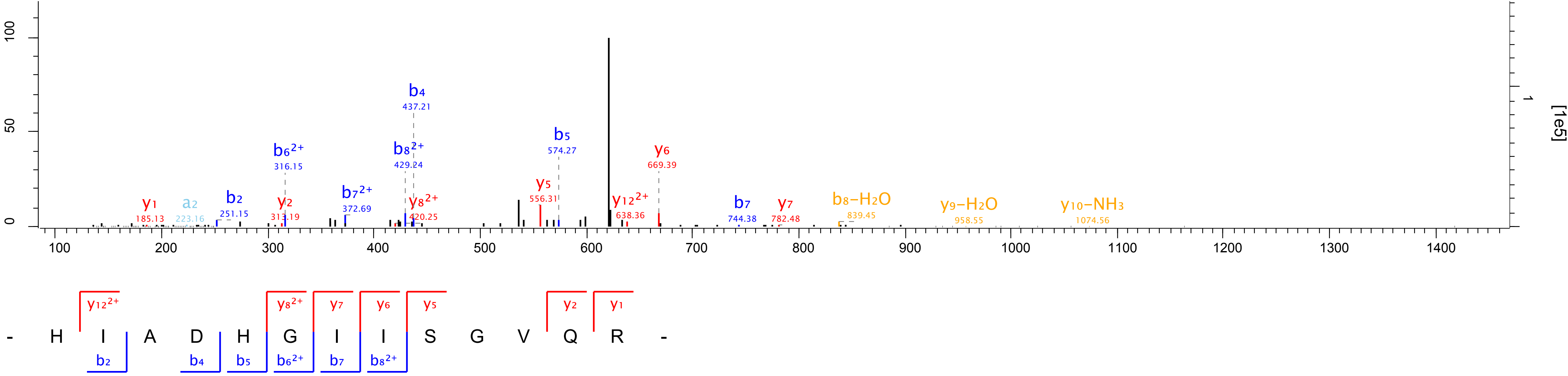

Raw file	Scan	Method	Score	m/z	Gene names
HBT_20130723_BV2_LPS_2_006	6349	ITMS; CID	100.69	484.91	Gabarap

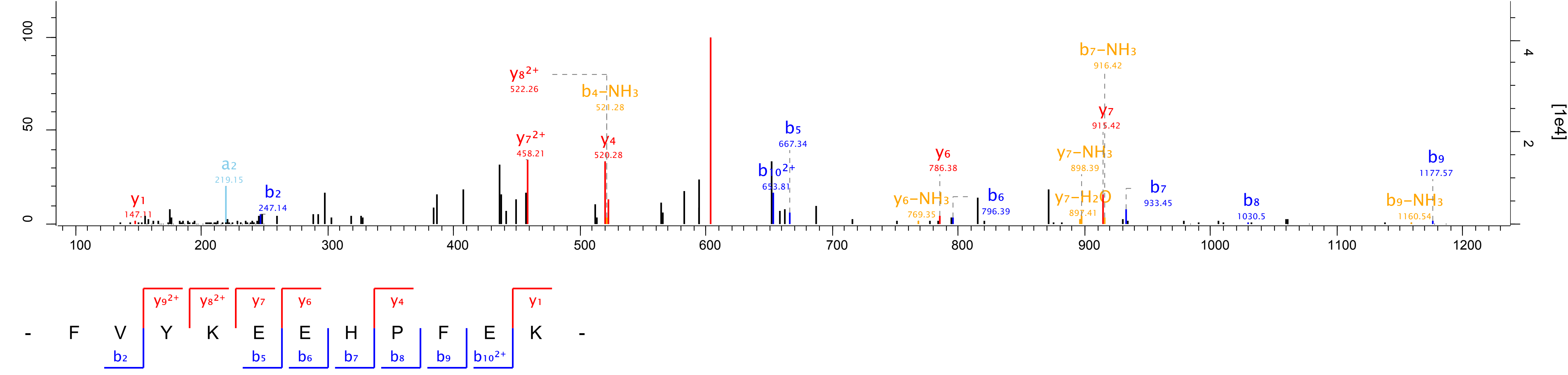

Raw file	Scan	Method	Score	m/z	Gene names
HBT_20130723_BV2_LPS_2_006	4658	ITMS; CID	110.6	692.31	1700037H04Rik

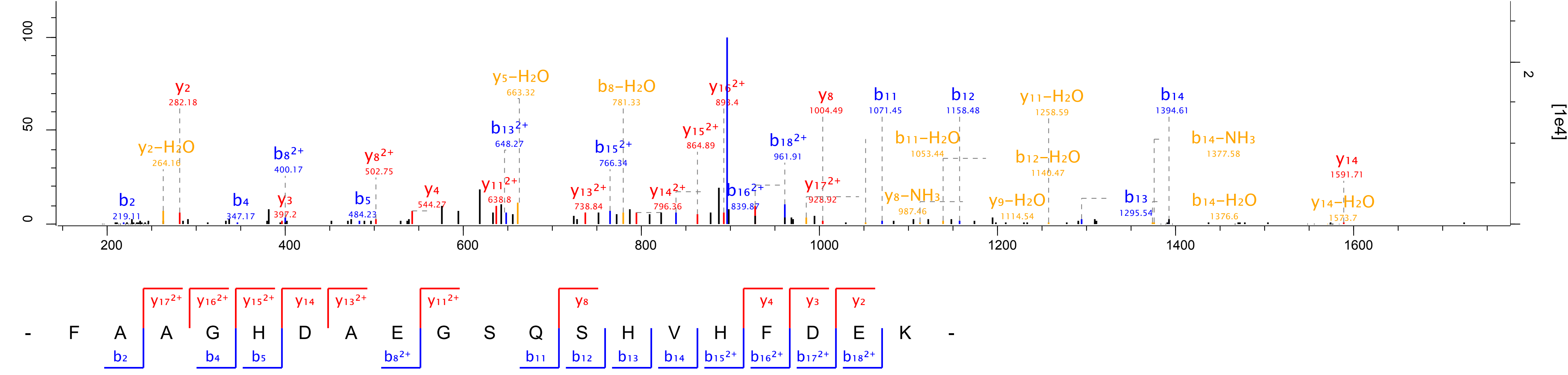

Raw file	Scan	Method	Score	m/z	Gene names
HBT_20130723_BV2_LPS_2_006	4079	ITMS; CID	93.56	448.89	Sumf1

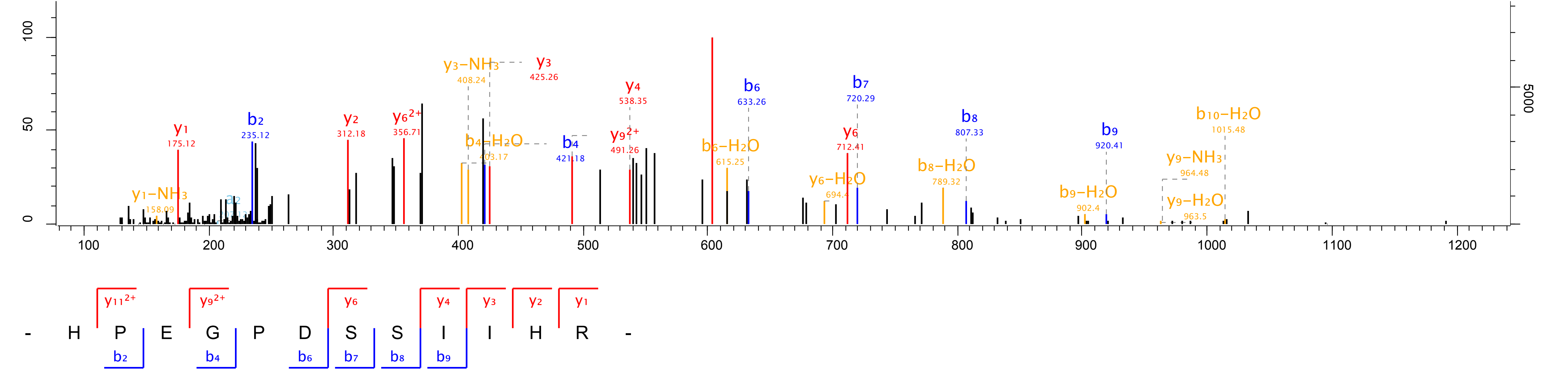

Raw file	Scan	Method	Score	m/z	Gene names
HBT_20130723_BV2_LPS_2_006	3238	ITMS; CID	75.65	426.21	Aifm2

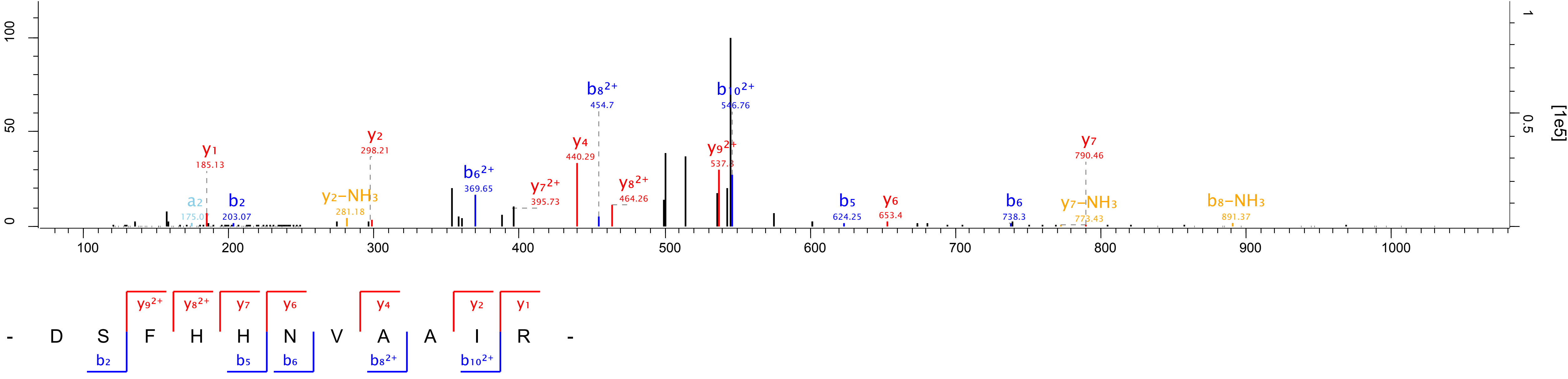

Raw file	Scan	Method	Score	m/z	Gene names
HBT_20130723_BV2_LPS_2_006	17078	ITMS; CID	54.25	538.79	Sco1

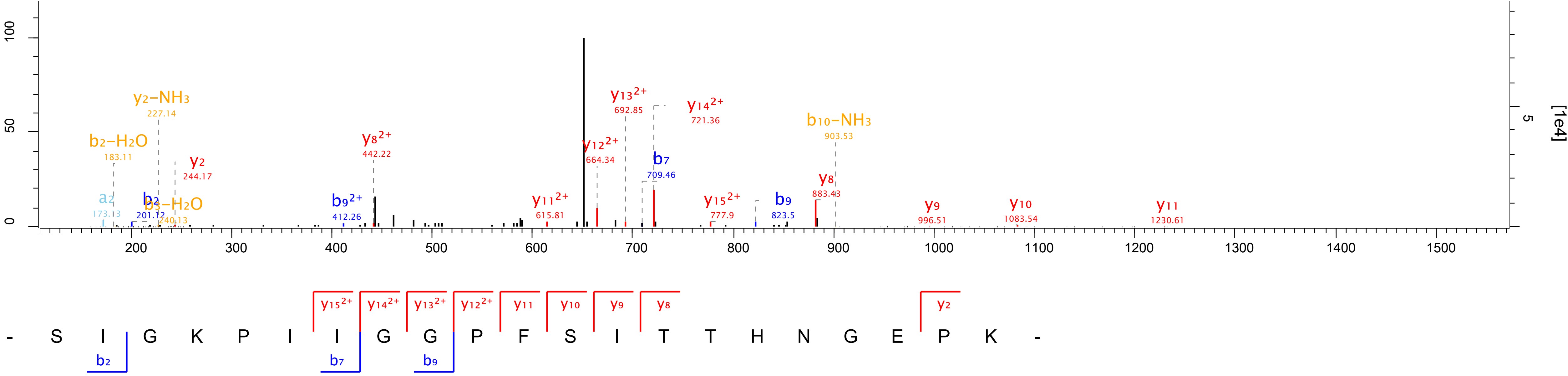

Raw file	Scan	Method	Score	m/z	Gene names
HBT_20130723_BV2_LPS_2_006	1281	ITMS; CID	111.78	576.6	Arfip2

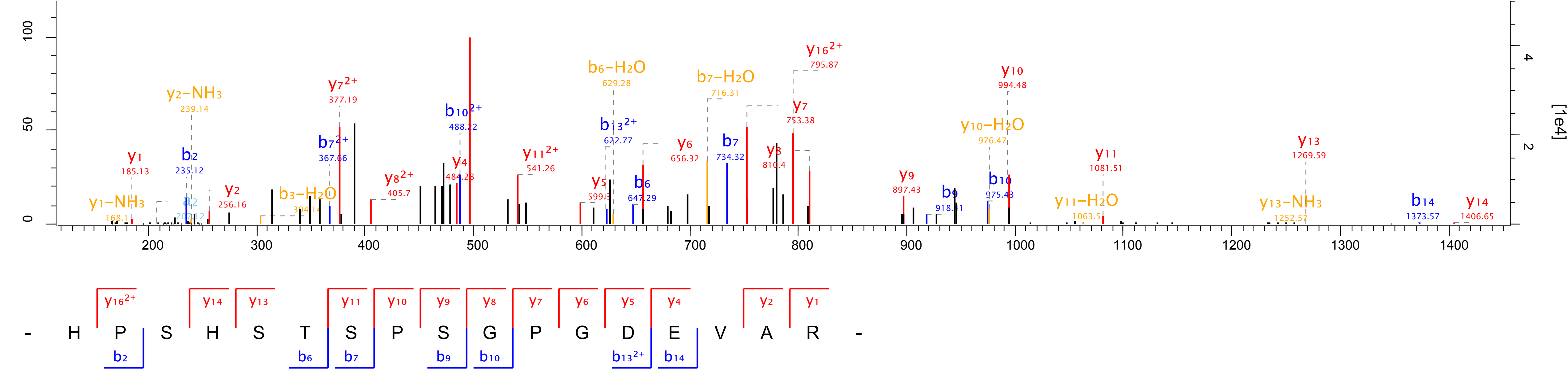

Raw file	Scan	Method	Score	m/z	Gene names
HBT_20130723_BV2_LPS_2_005	9947	ITMS; CID	117.86	529.94	Fam32a

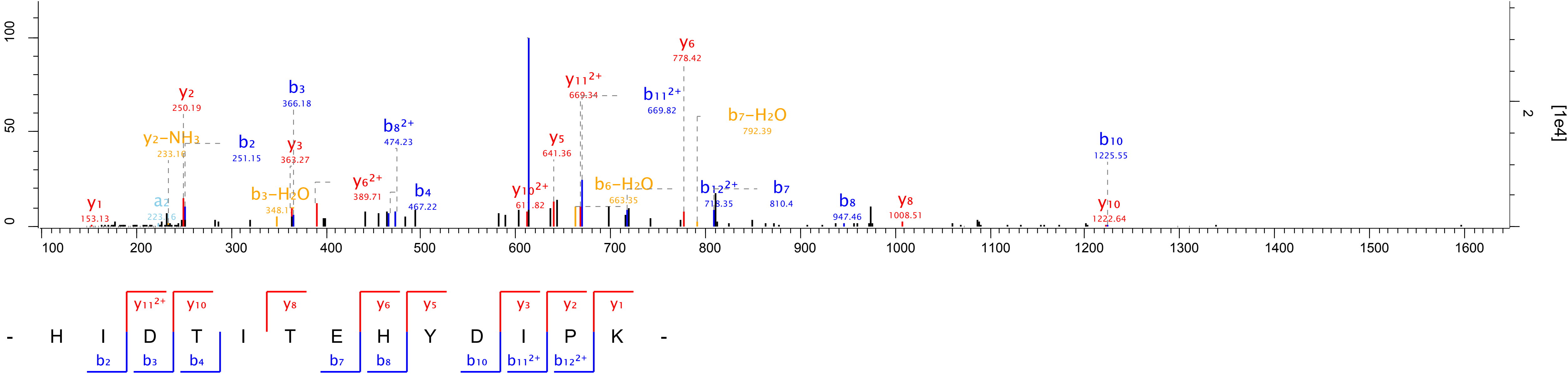

Raw file	Scan	Method	Score	m/z	Gene names
HBT_20130723_BV2_LPS_2_005	25831	ITMS; CID	47.27	881.41	Crtc2

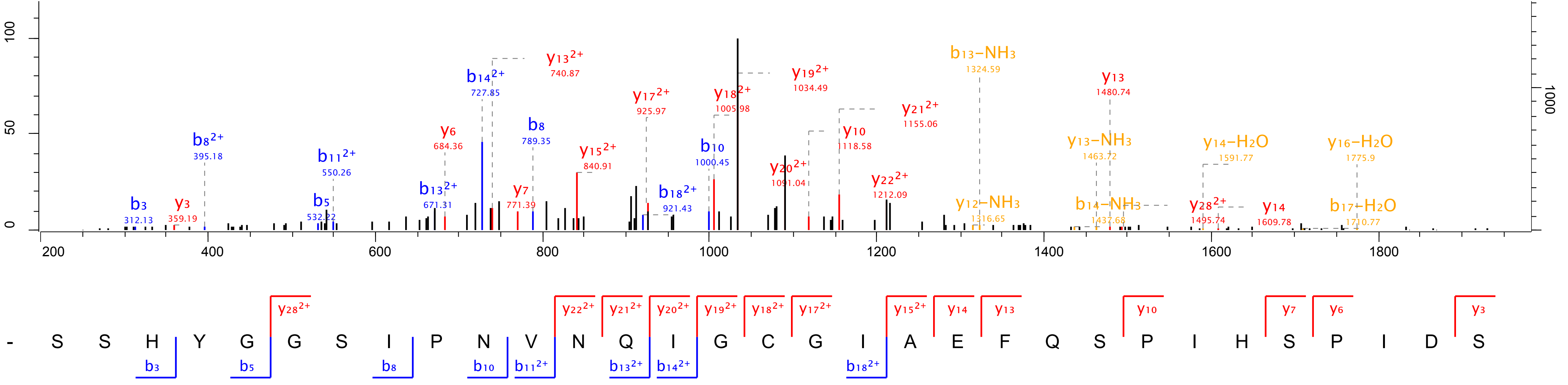

Raw file	Scan	Method	Score	m/z	Gene names
HBT_20130723_BV2_LPS_2_004	8961	ITMS; CID	76.16	464.92	Sema3d

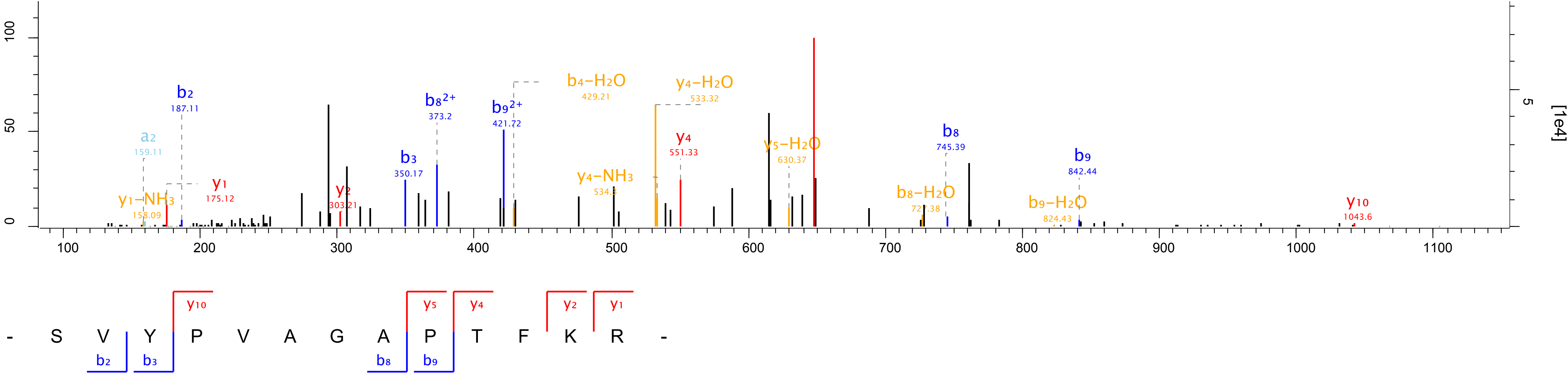

Raw file	Scan	Method	Score	m/z	Gene names
HBT_20130723_BV2_LPS_2_004	11409	ITMS; CID	66.57	675.34	Milr1

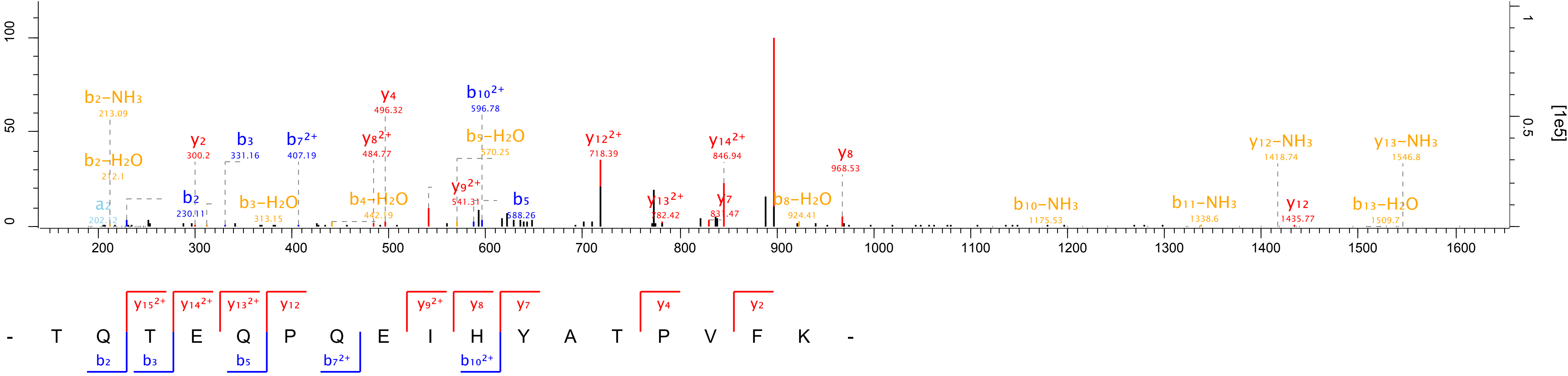

Raw file	Scan	Method	Score	m/z	Gene names
HBT_20130723_BV2_LPS_2_003	7550	ITMS; CID	61.53	970.43	Dgkh

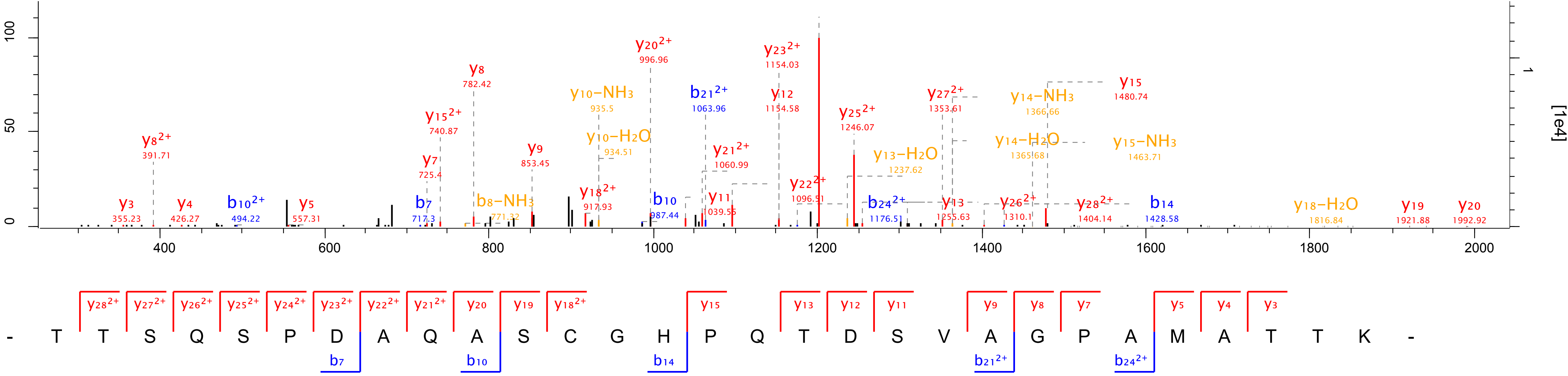

Raw file	Scan	Method	Score	m/z	Gene names
HBT_20130723_BV2_LPS_2_003	26630	ITMS; CID	76.12	809.74	Trappc13

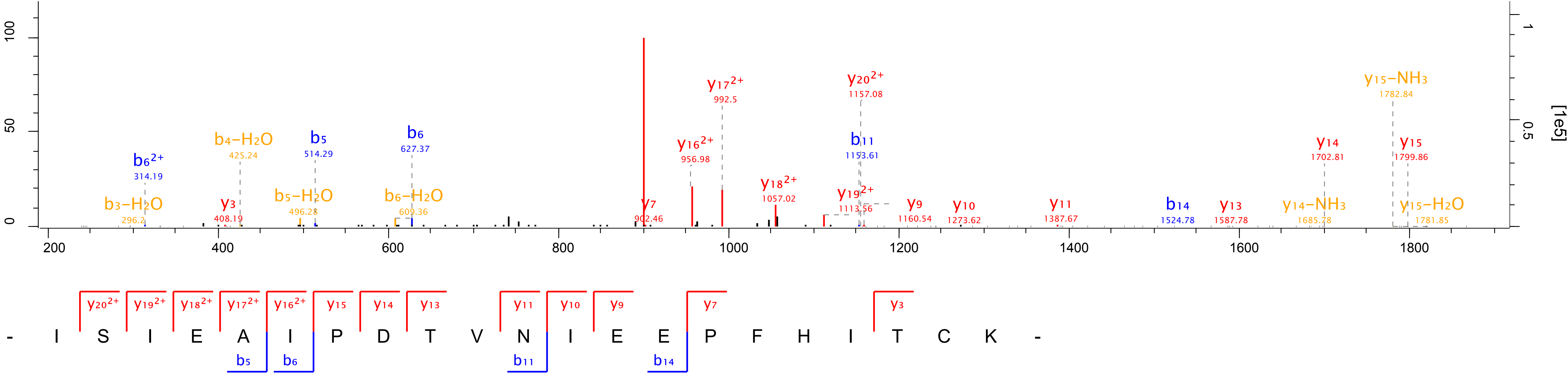

Raw file	Scan	Method	Score	m/z	Gene names
HBT_20130723_BV2_LPS_2_003	15598	ITMS; CID	55.71	811.39	Slc6a9

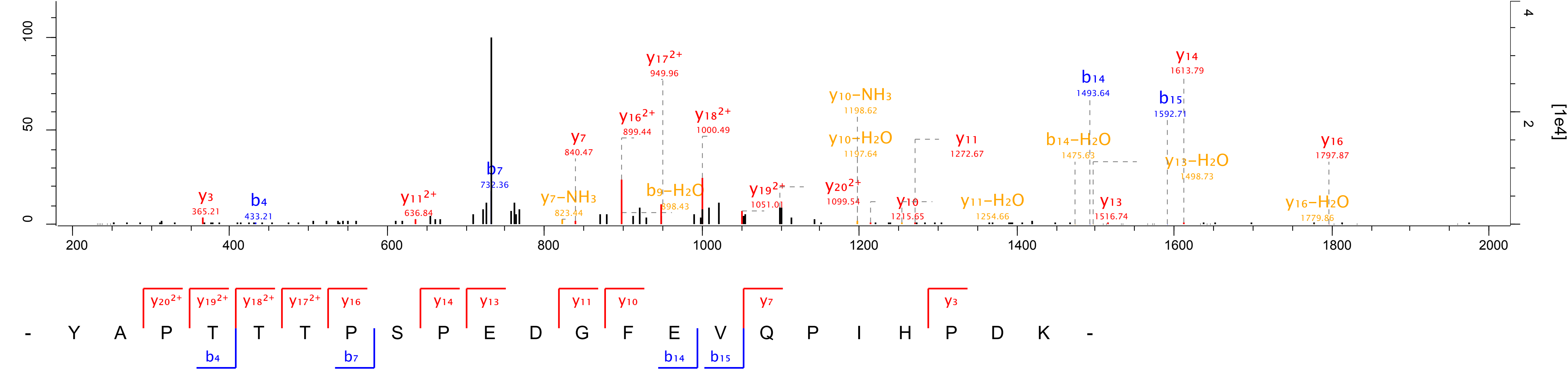

Raw file	Scan	Method	Score	m/z	Gene names
HBT_20130723_BV2_LPS_2_003	15469	ITMS; CID	216.71	945.43	Slc11a1

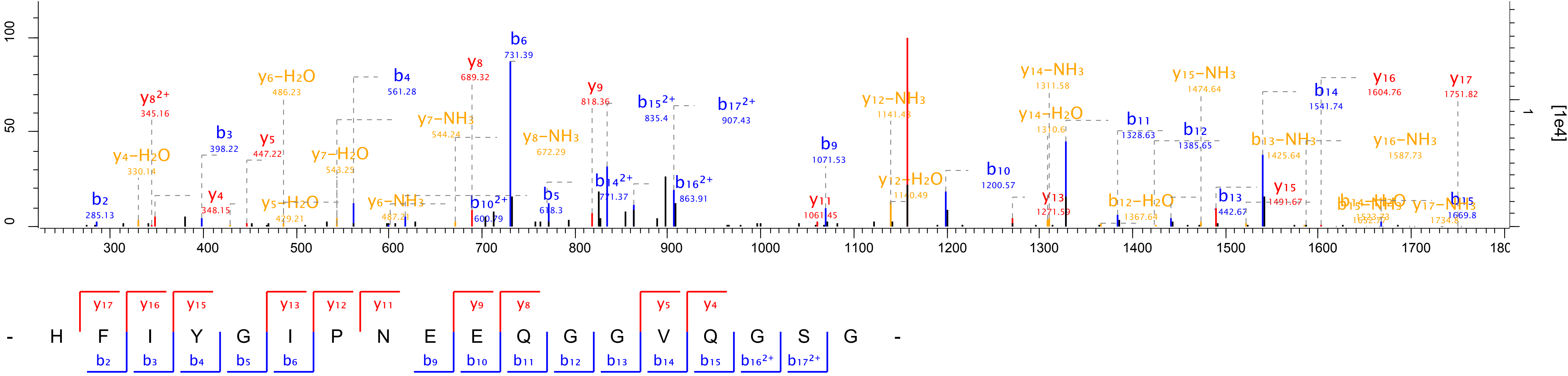

Raw file	Scan	Method	Score	m/z	Gene names
HBT_20130723_BV2_LPS_2_003	1174	ITMS; CID	139.12	684.97	Rabl2

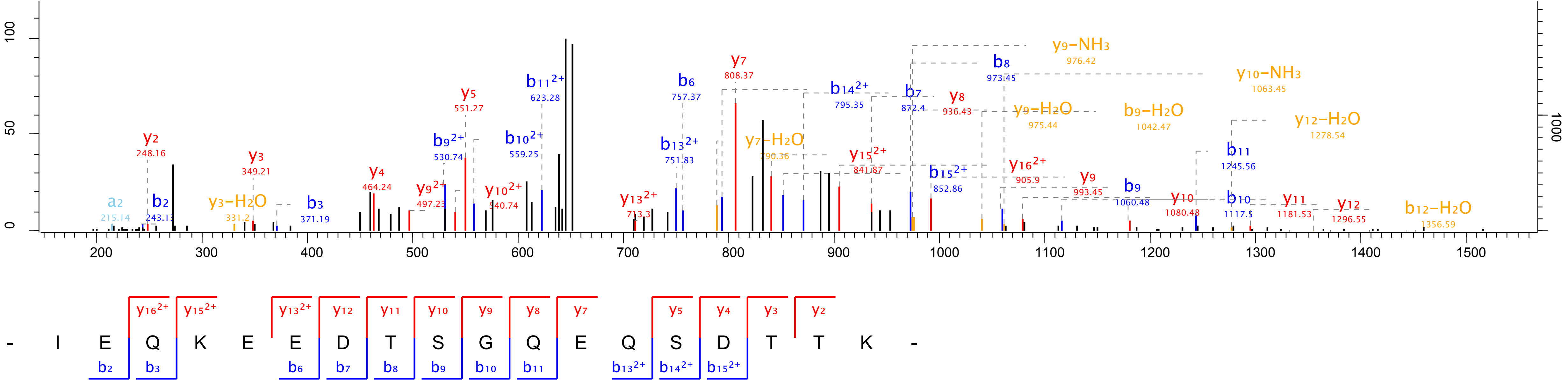

Raw file	Scan	Method	Score	m/z	Gene names
HBT_20130723_BV2_LPS_2_003	11356	ITMS; CID	78.42	767.38	Rybp

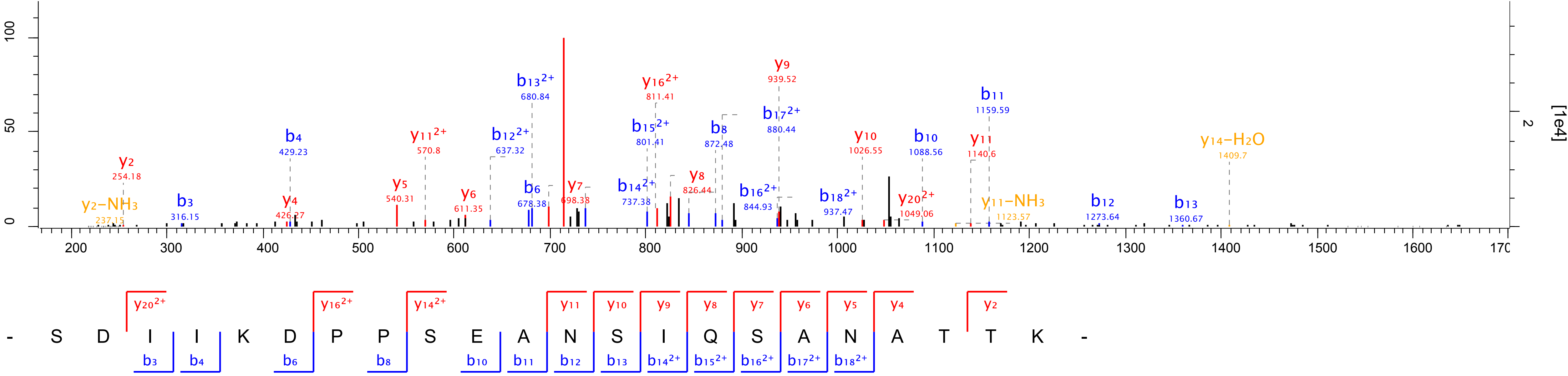

Raw file	Scan	Method	Score	m/z	Gene names
HBT_20130723_BV2_LPS_2_002	20822	ITMS; CID	109.83	970.79	Tmem128

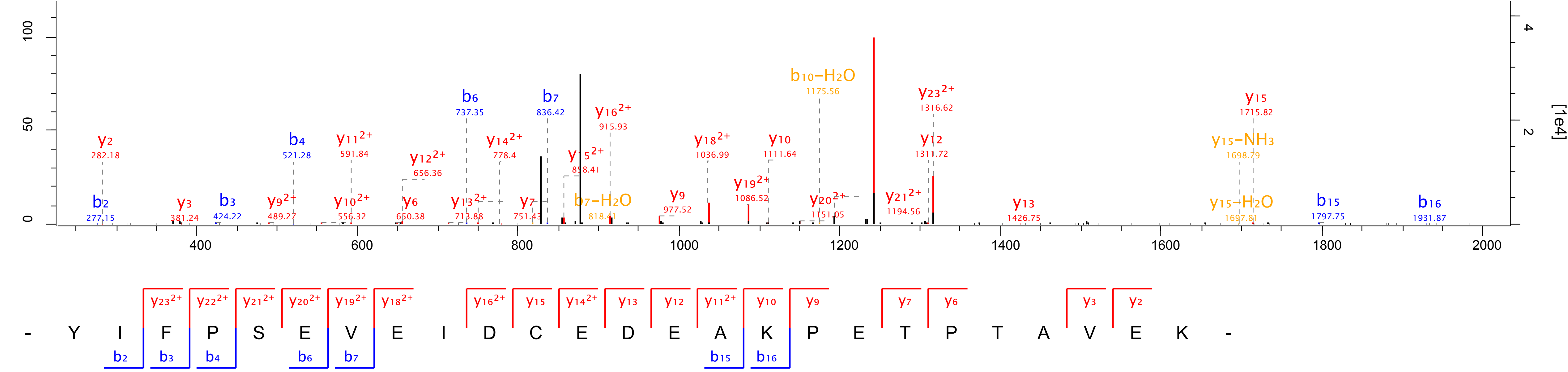

Raw file	Scan	Method	Score	m/z
HBT_20130723_BV2_LPS_2_002	15984	ITMS; CID	116.52	558.3

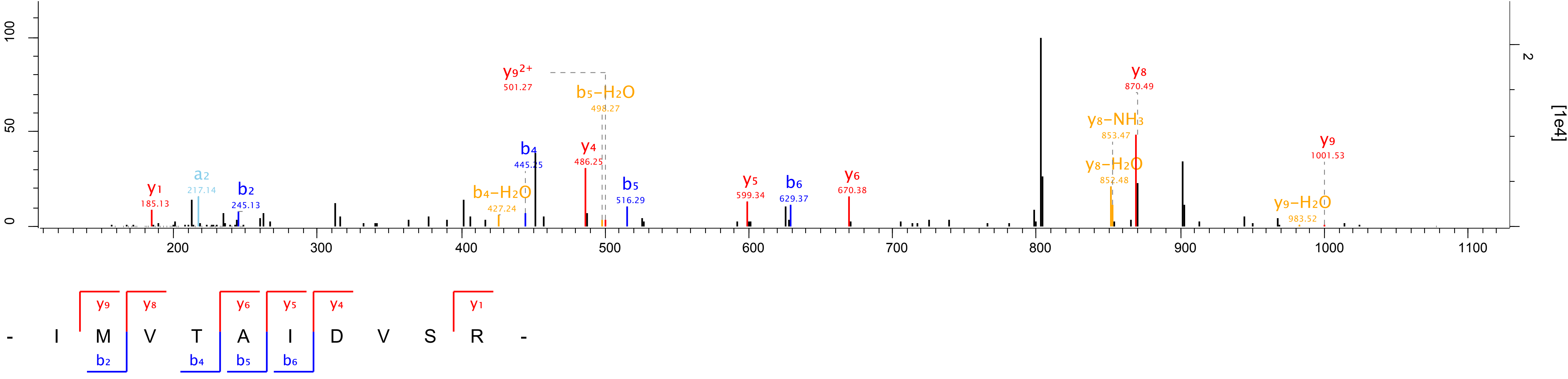

Raw file	Scan	Method	Score	m/z	Gene names
HBT_20130723_BV2_LPS_2_002	10316	ITMS; CID	196.27	646.8	Scamp4

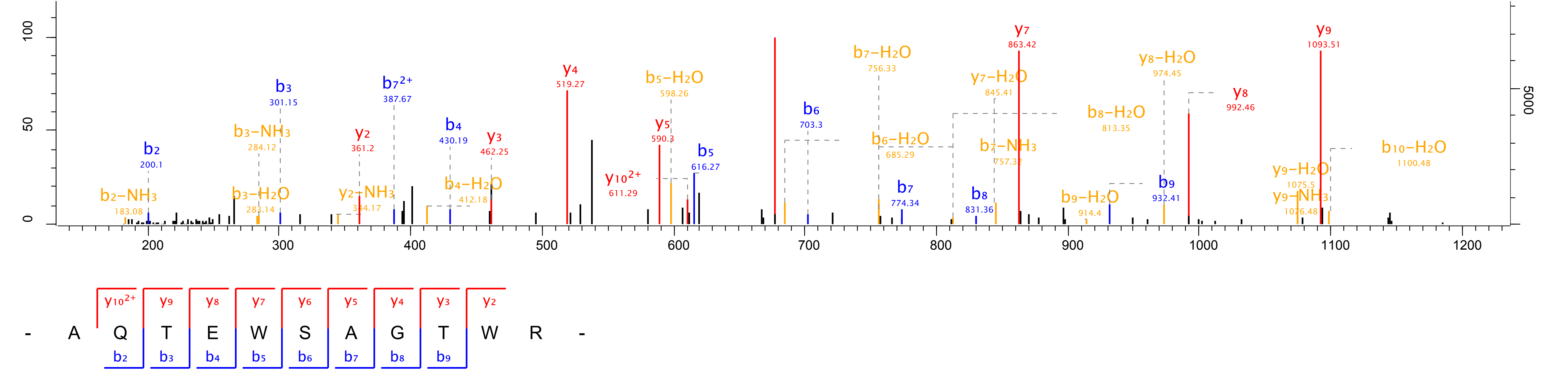

Raw file	Scan	Method	Score	m/z	Gene names
HBT_20130723_BV2_LPS_2_001	8637	ITMS; CID	135.57	933.42	Romo1

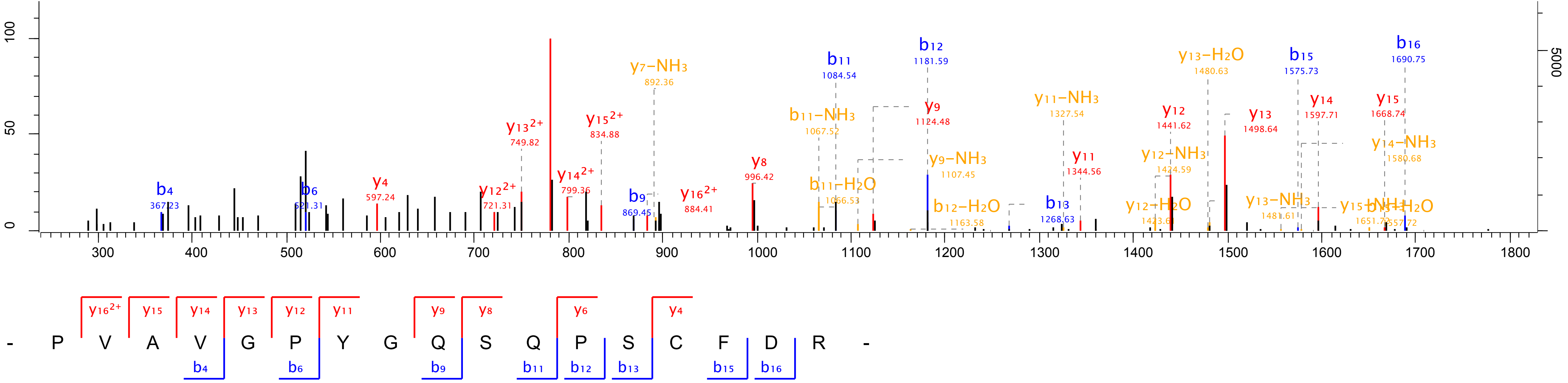

Raw file	Scan	Method	Score	m/z	Gene names
HBT_20130723_BV2_LPS_2_001	5258	ITMS; CID	153.1	597.79	Serpina12

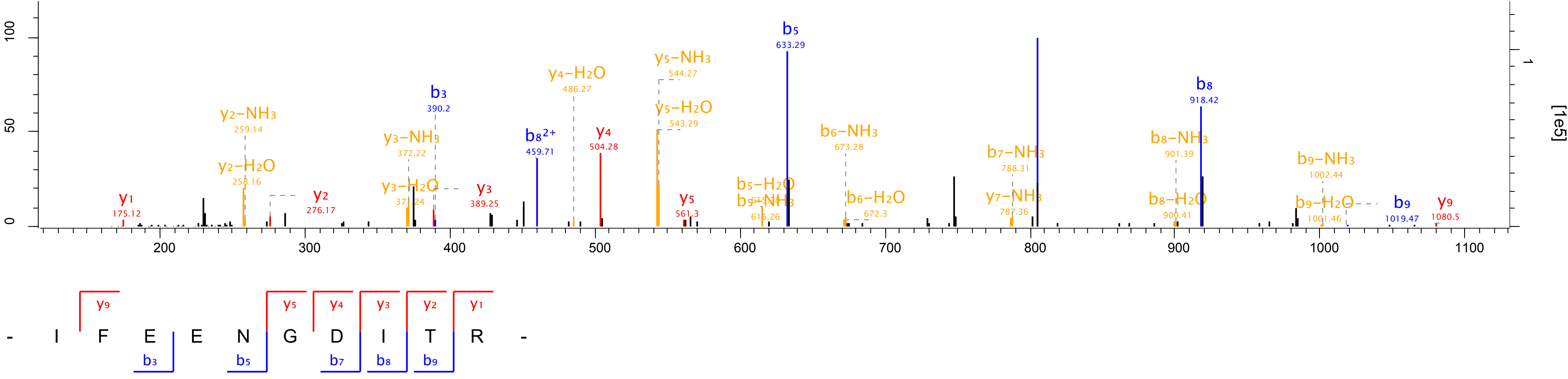

Raw file	Scan	Method	Score	m/z	Gene names
HBT_20130723_BV2_LPS_2_001	2828	ITMS; CID	140.31	641.8	Cdkn2aip

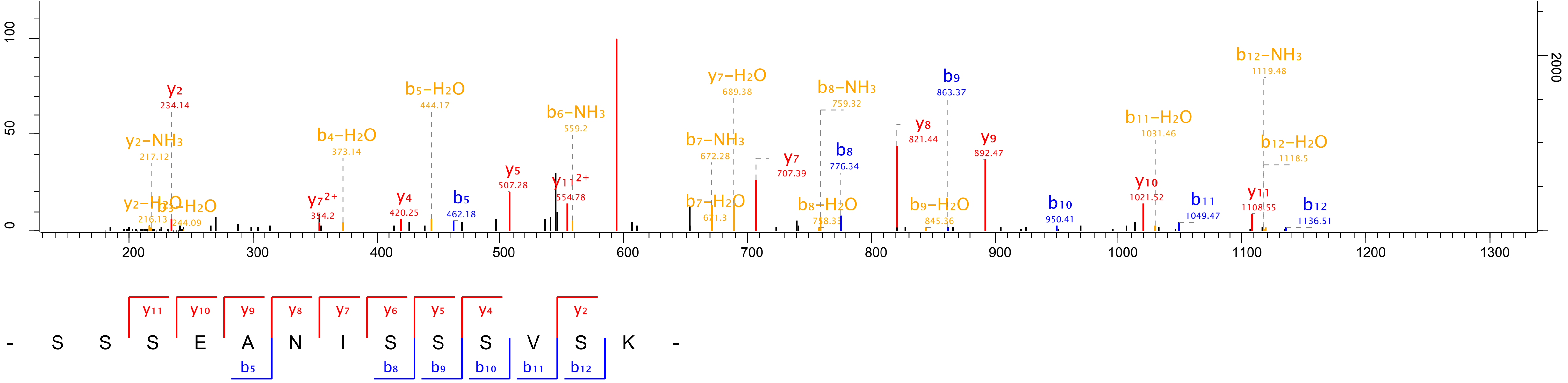

Raw file	Scan	Method	Score	m/z	Gene names
HBT_20130723_BV2_LPS_2_001	1345	ITMS; CID	169.76	480.76	Mecp2

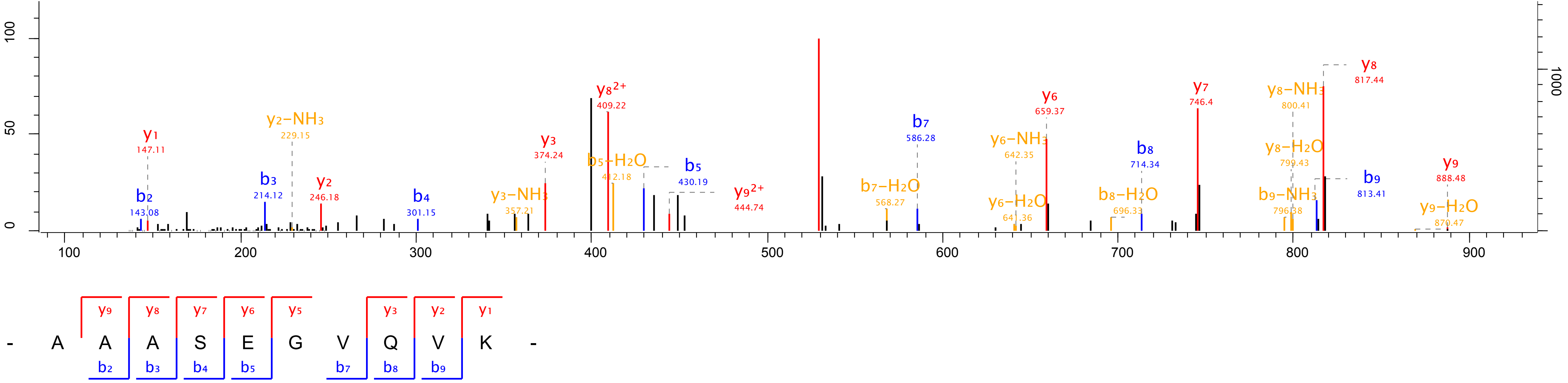

Raw file	Scan	Method	Score	m/z	Gene names
HBT_20130723_BV2_LPS_1_006	24454	ITMS; CID	58.81	773.16	Ubap1

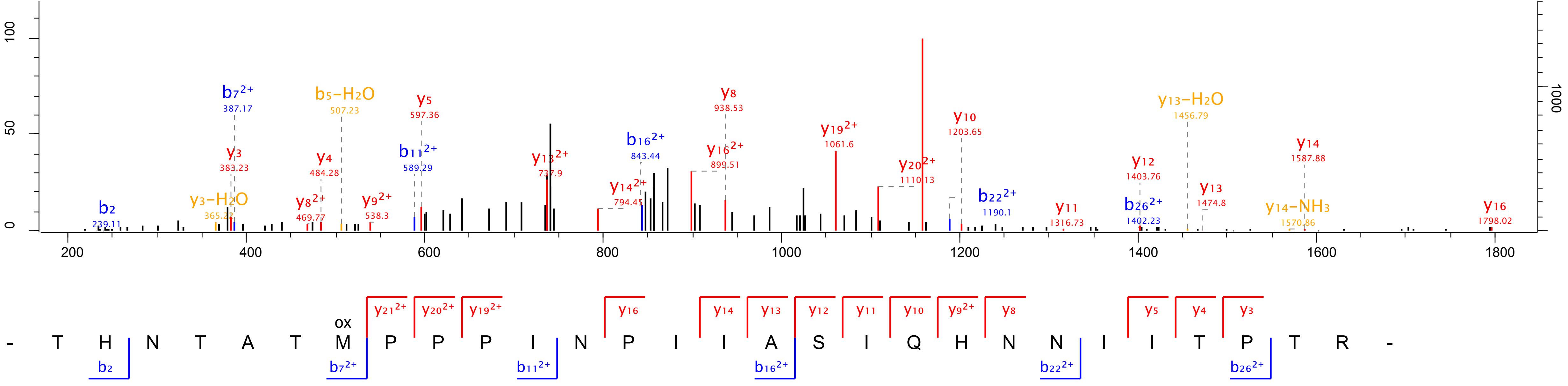

Raw file	Scan	Method	Score	m/z	Gene names
HBT_20130723_BV2_LPS_1_006	2025	ITMS; CID	61.41	627.35	Dlx5;Dlx2

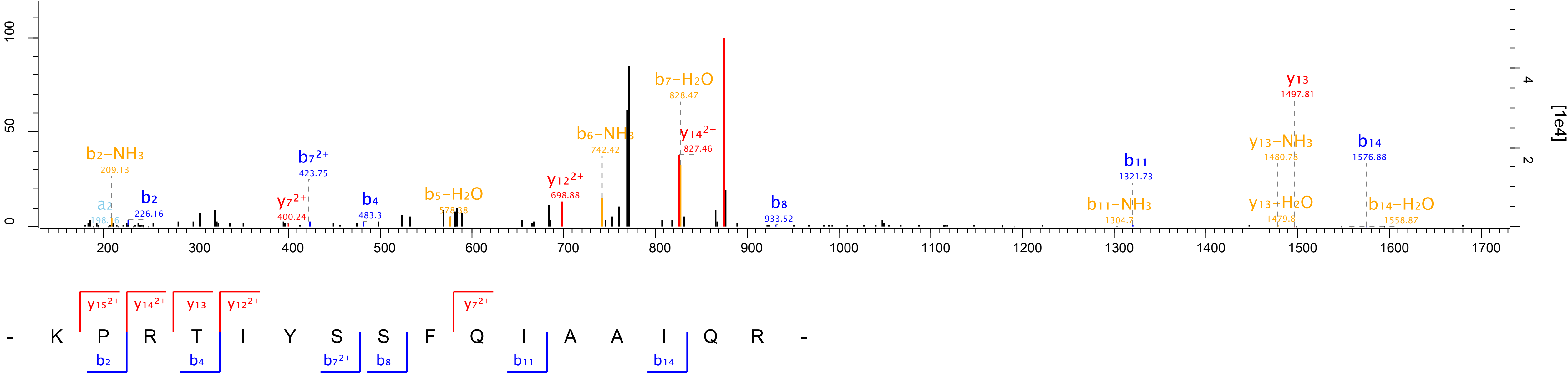

Raw file	Scan	Method	Score	m/z	Gene names
HBT_20130723_BV2_LPS_1_006	13943	ITMS; CID	68.41	504.28	Mrps18a

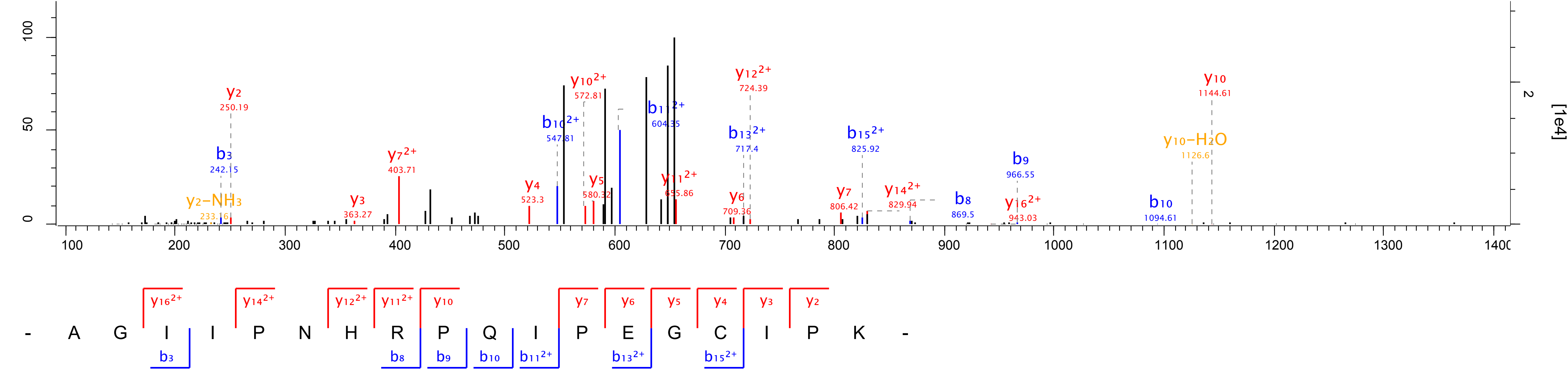

Raw file	Scan	Method	Score	m/z	Gene names
HBT_20130723_BV2_LPS_1_006	10615	ITMS; CID	84.75	560.96	Thy1

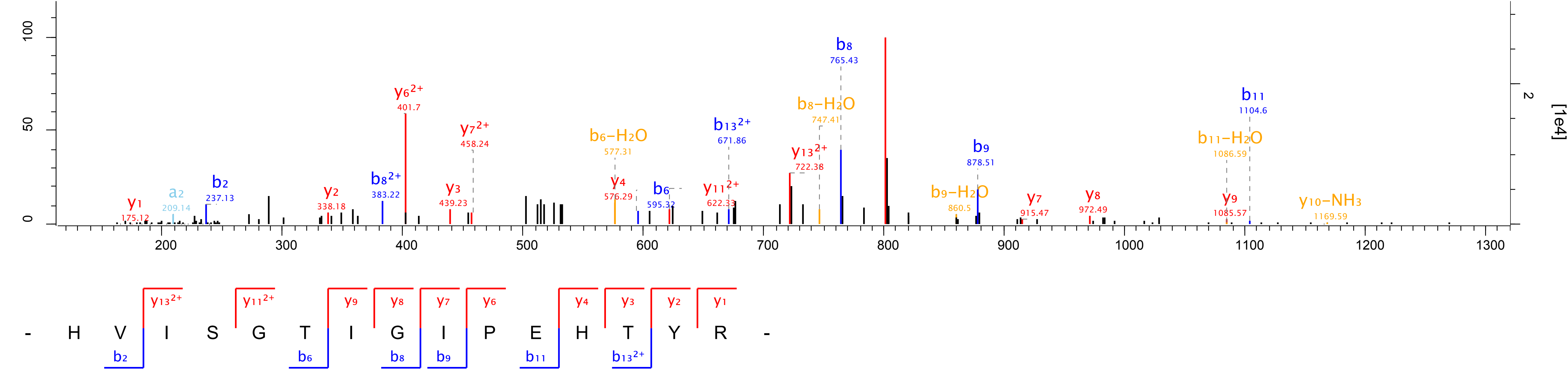

Raw file	Scan	Method	Score	m/z	Gene names
HBT_20130723_BV2_LPS_1_005	27015	ITMS; CID	58.8	879.43	Cd81

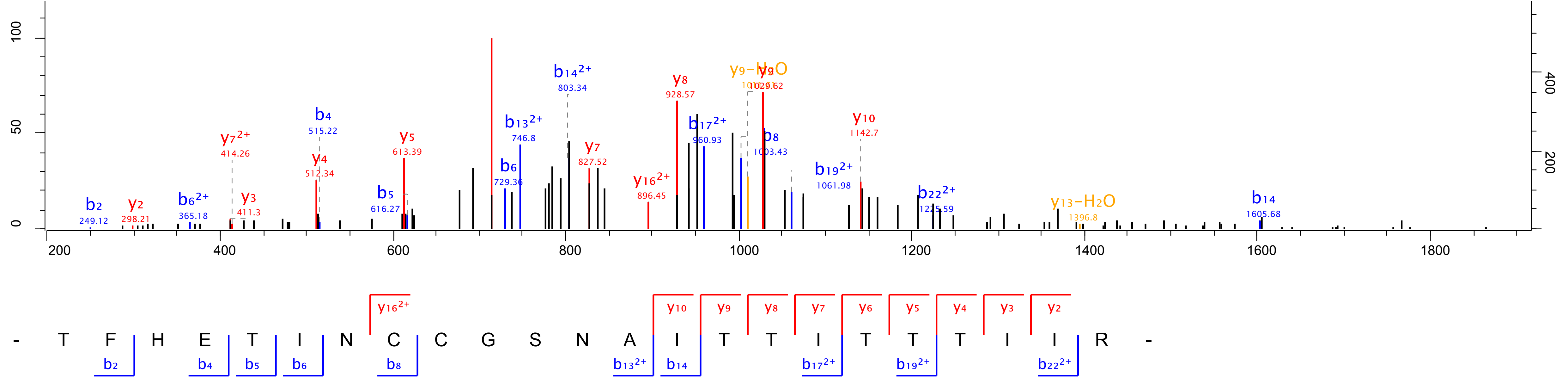

Raw file	Scan	Method	Score	m/z	Gene names
HBT_20130723_BV2_LPS_1_005	24993	ITMS; CID	56.47	982.2	Cdc42se2

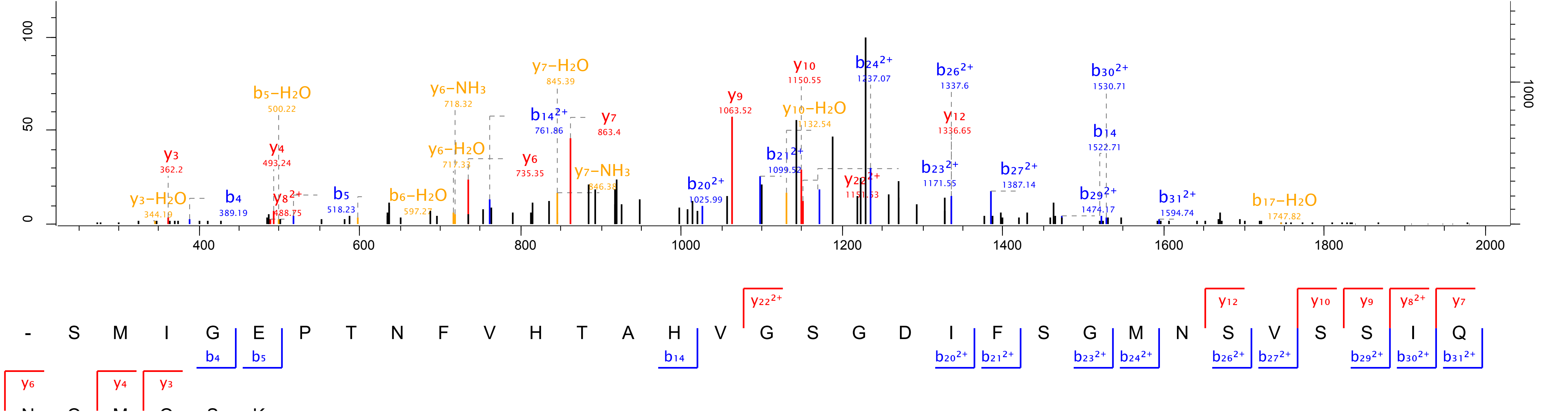

Raw file	Scan	Method	Score	m/z	Gene names
HBT_20130723_BV2_LPS_1_004	27768	ITMS; CID	52.75	865.79	Med28

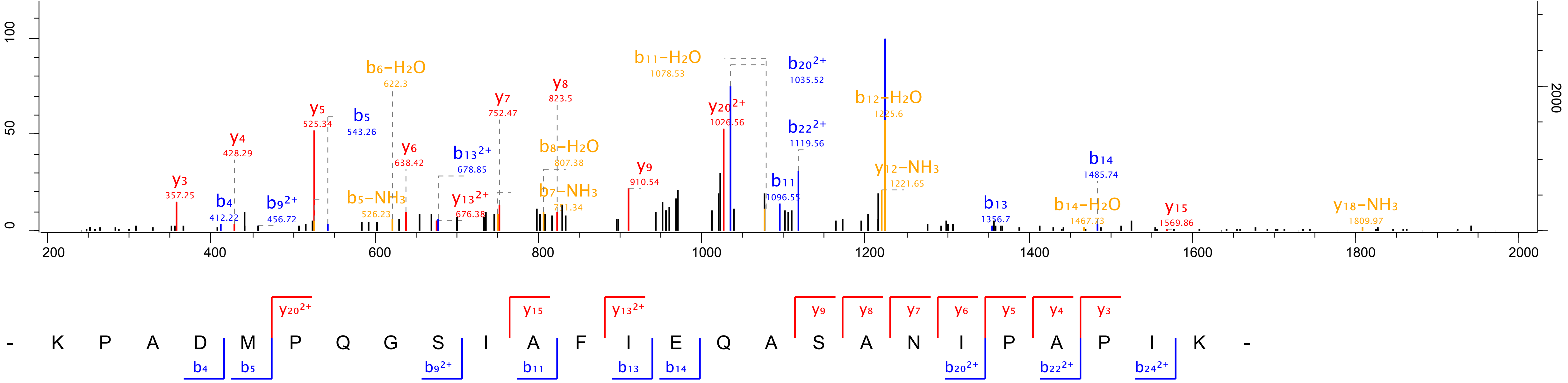

Raw file	Scan	Method	Score	m/z	Gene names
HBT_20130723_BV2_LPS_1_004	19723	ITMS; CID	139.12	955	Atp6v0c

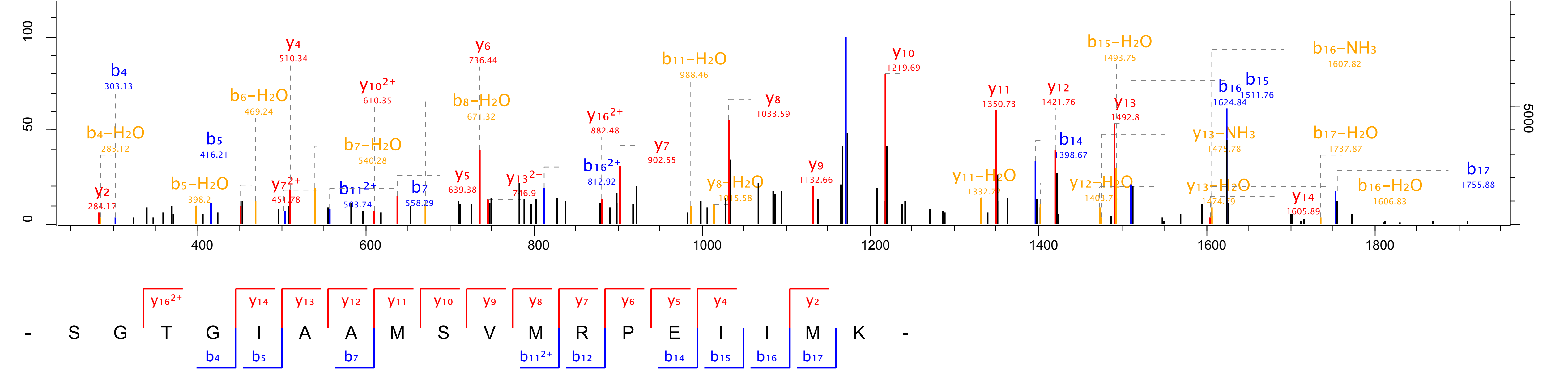

Raw file	Scan	Method	Score	m/z	Gene names
HBT_20130723_BV2_LPS_1_004	1186	ITMS; CID	65.86	593.99	Smox

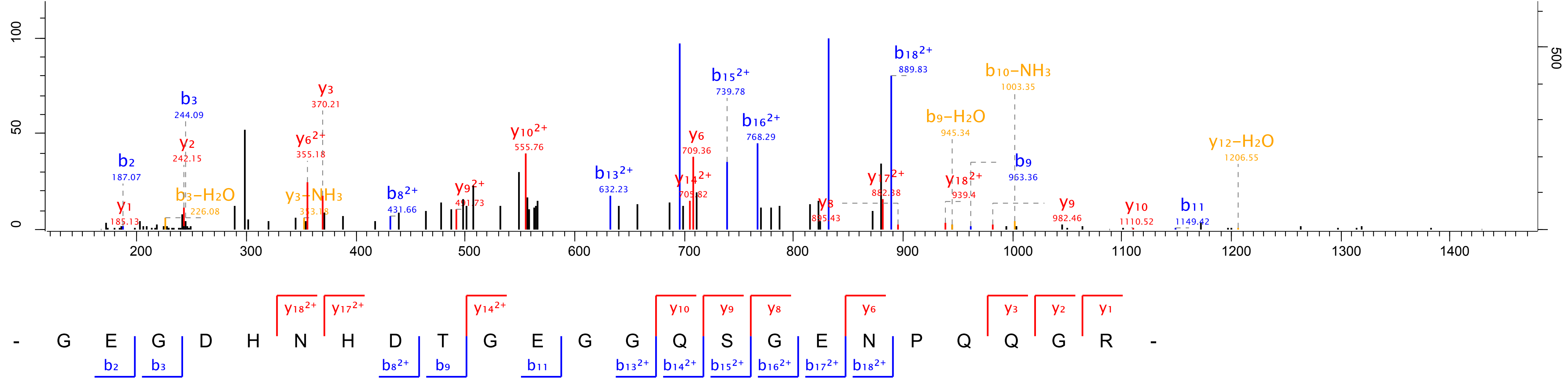

Raw file	Scan	Method	Score	m/z	Gene names
HBT_20130723_BV2_LPS_1_003	987	ITMS; CID	177.1	574.74	Ankrd54

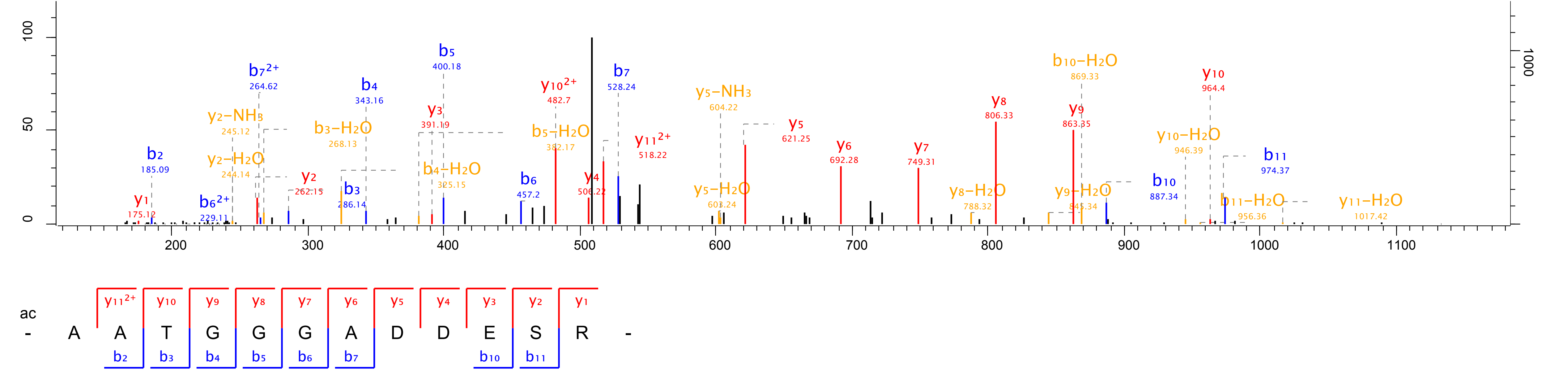

Raw file	Scan	Method	Score	m/z	Gene names
HBT_20130723_BV2_LPS_1_003	33979	ITMS; CID	118.52	863.93	Tomm7

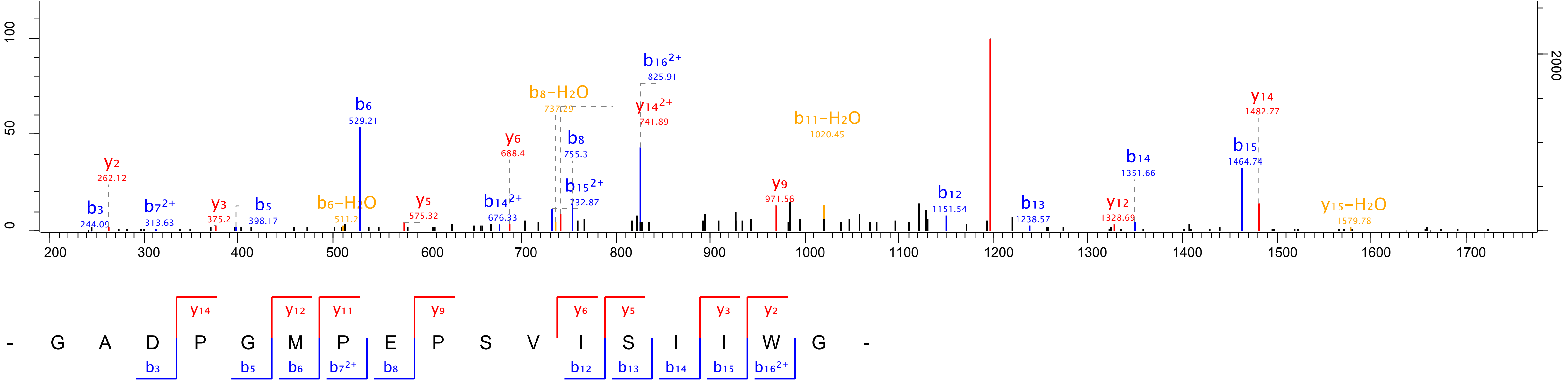

Raw file	Scan	Method	Score	m/z	Gene names
HBT_20130723_BV2_LPS_1_003	1470	ITMS; CID	112.41	480.24	Ticrr

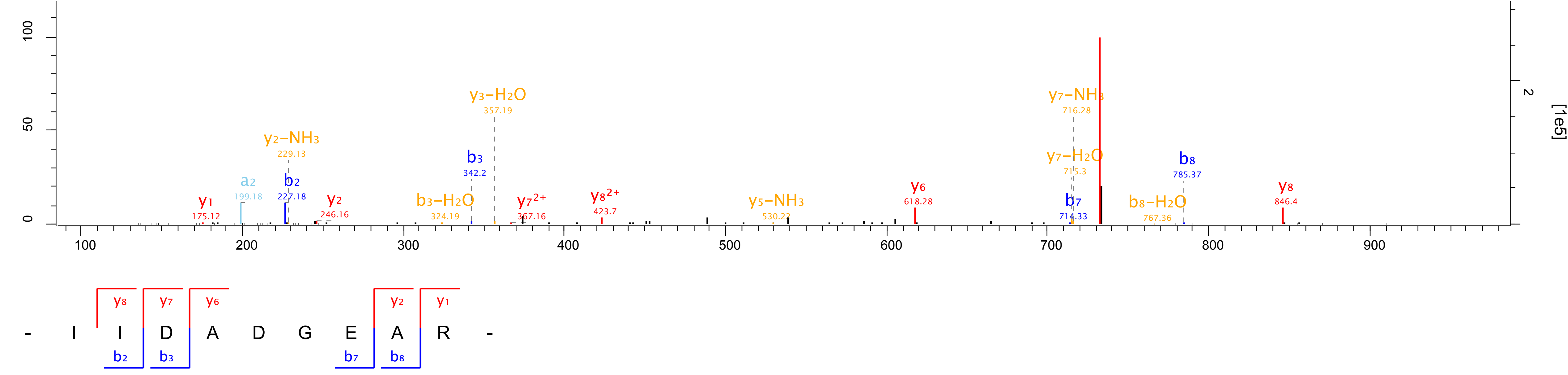

Raw file	Scan	Method	Score	m/z	Gene names
HBT_20130723_BV2_LPS_1_003	1174	ITMS; CID	196.8	774.31	Cend1

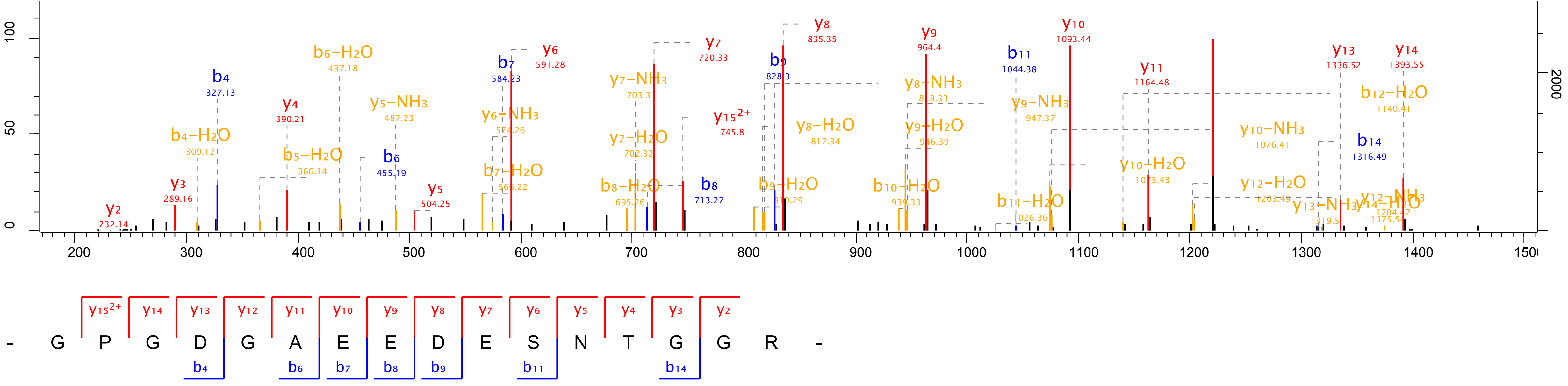

Raw file	Scan	Method	Score	m/z	Gene names
HBT_20130723_BV2_LPS_1_003	1055	ITMS; CID	287.88	832.36	Tgoln1;Tgoln2

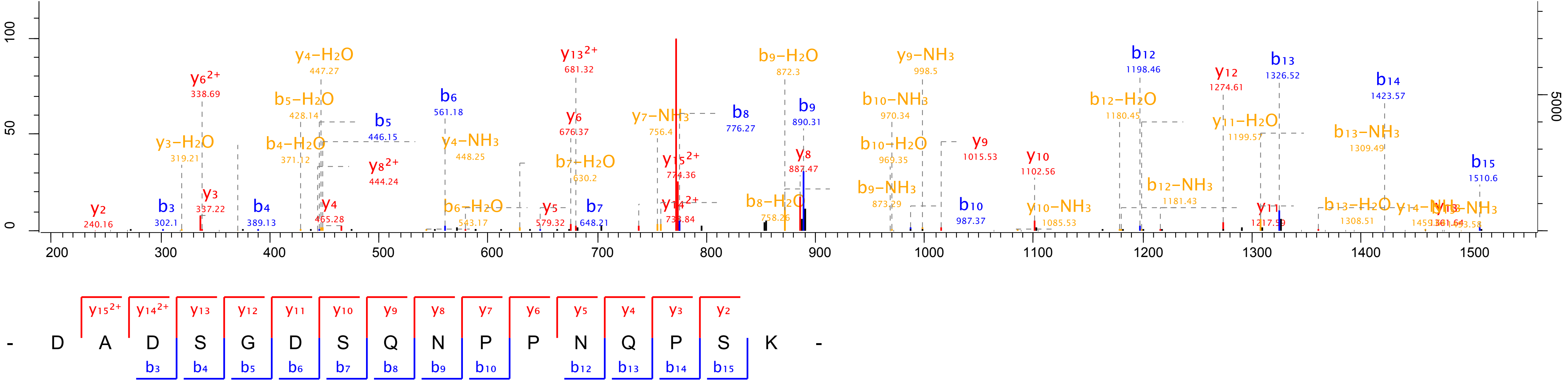

Raw file	Scan	Method	Score	m/z	Gene names
HBT_20130723_BV2_LPS_1_002	7929	ITMS; CID	82.49	689.31	Zfp597

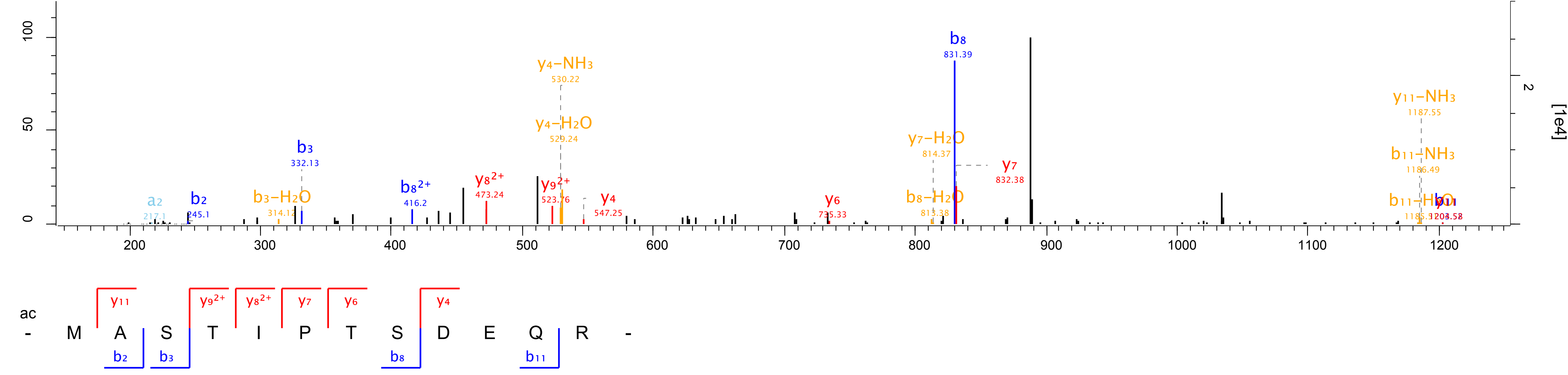

Raw file	Scan	Method	Score	m/z	Gene names
HBT_20130723_BV2_LPS_1_002	7201	ITMS; CID	109.83	619.81	Chmp2b

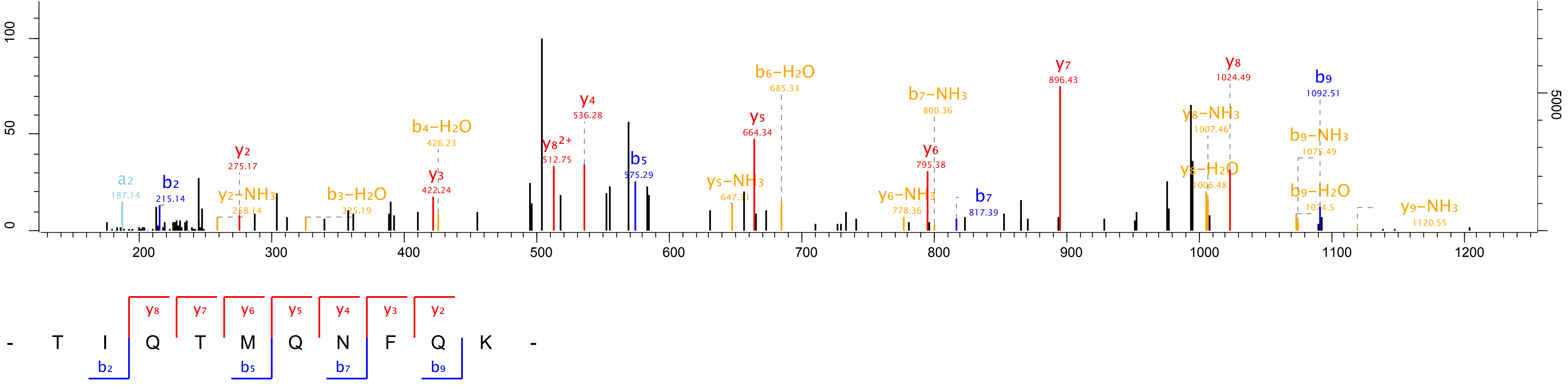

Raw file	Scan	Method	Score	m/z	Gene names
HBT_20130723_BV2_LPS_1_002	32404	ITMS; CID	62.57	1200.54	Tfdp1

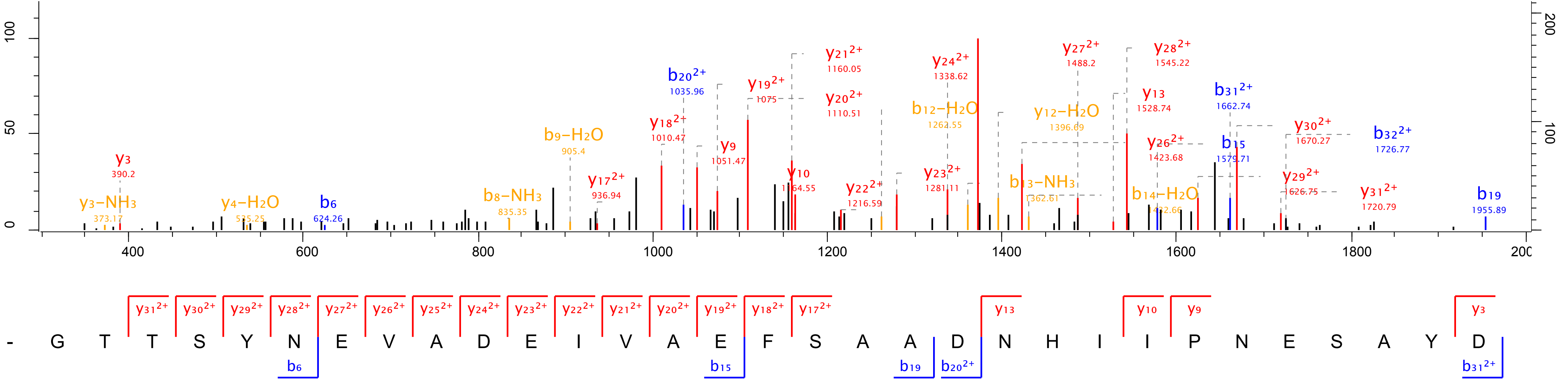

Raw file	Scan	Method	Score	m/z	Gene names
HBT_20130723_BV2_LPS_1_002	32117	ITMS; CID	103.02	993.47	Dusp28

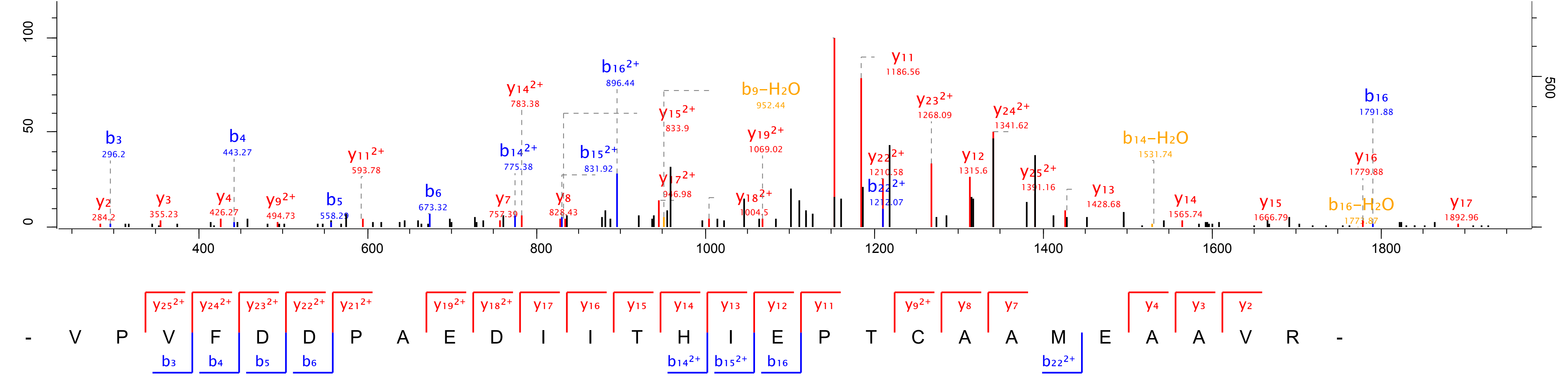

Raw file	Scan	Method	Score	m/z	Gene names
HBT_20130723_BV2_LPS_1_002	31186	ITMS; CID	145.67	957.84	Gpatch1

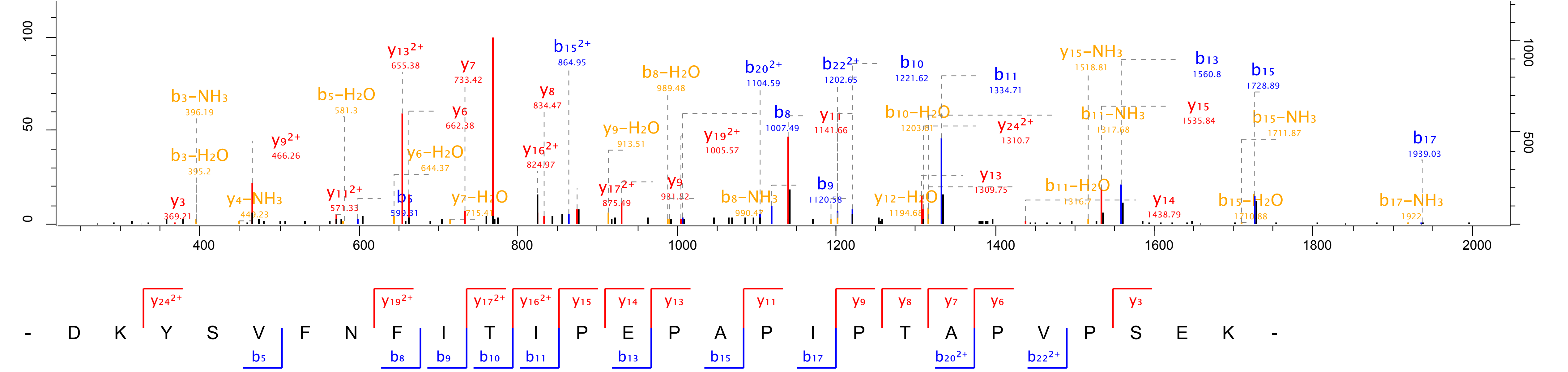

Raw file	Scan	Method	Score	m/z	Gene names
HBT_20130723_BV2_LPS_1_002	30310	ITMS; CID	121.35	1019.15	Rpp14

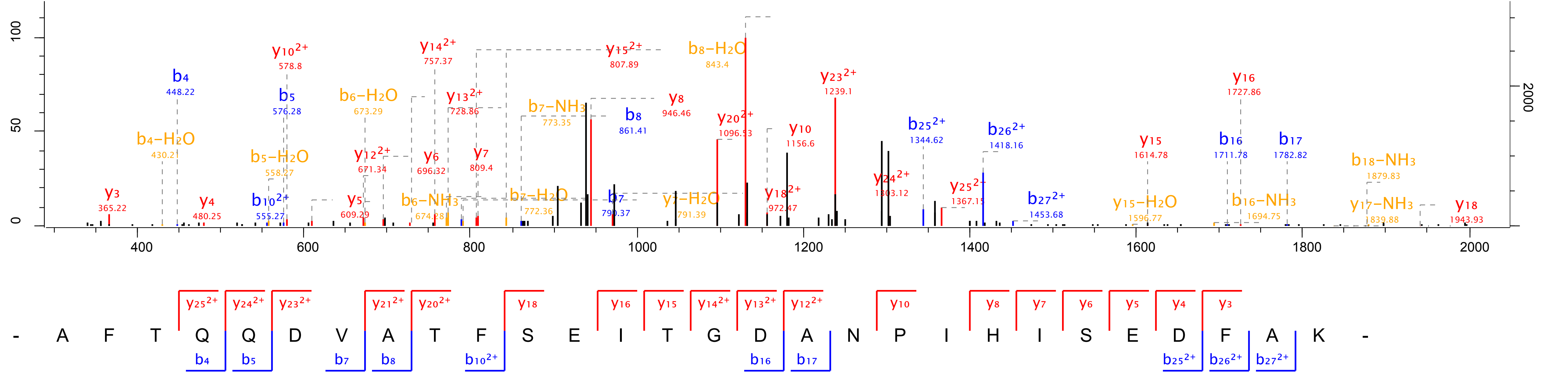

Raw file	Scan	Method	Score	m/z	Gene names
HBT_20130723_BV2_LPS_1_002	29841	ITMS; CID	69.53	1013.88	Eif2d

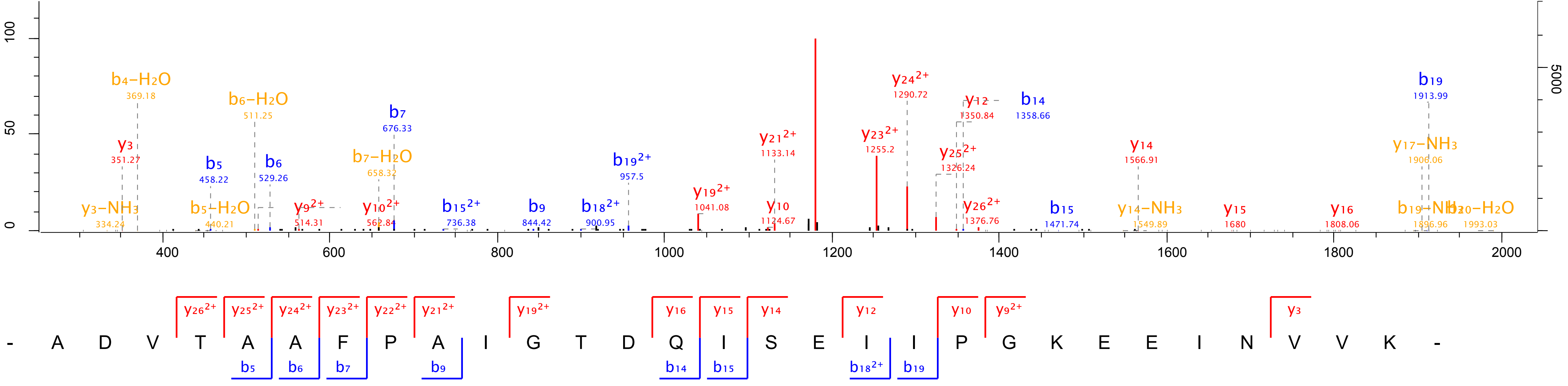

Raw file	Scan	Method	Score	m/z	Gene names
HBT_20130723_BV2_LPS_1_002	25455	ITMS; CID	113.38	740.87	Plp1

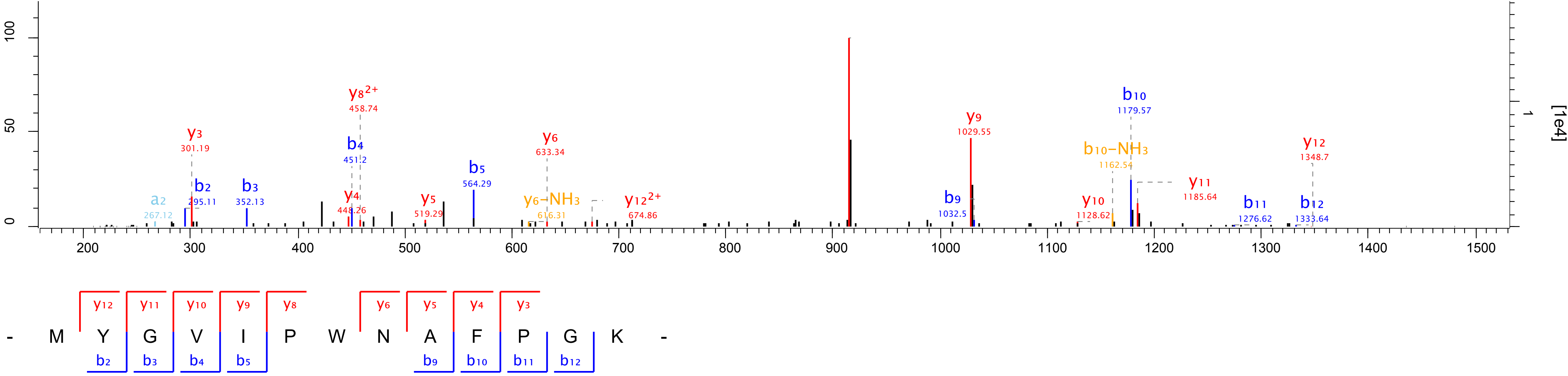

Raw file	Scan	Method	Score	m/z	Gene names
HBT_20130723_BV2_LPS_1_002	24791	ITMS; CID	101.45	943.43	Tmem222

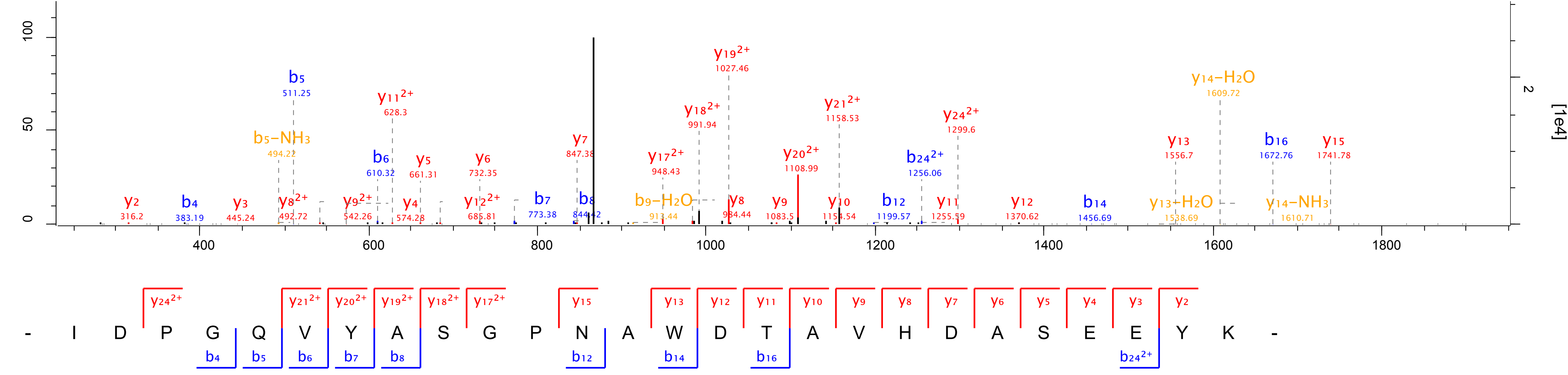

Raw file	Scan	Method	Score	m/z	Gene names
HBT_20130723_BV2_LPS_1_002	24783	ITMS; CID	97.9	607.29	Cdc45

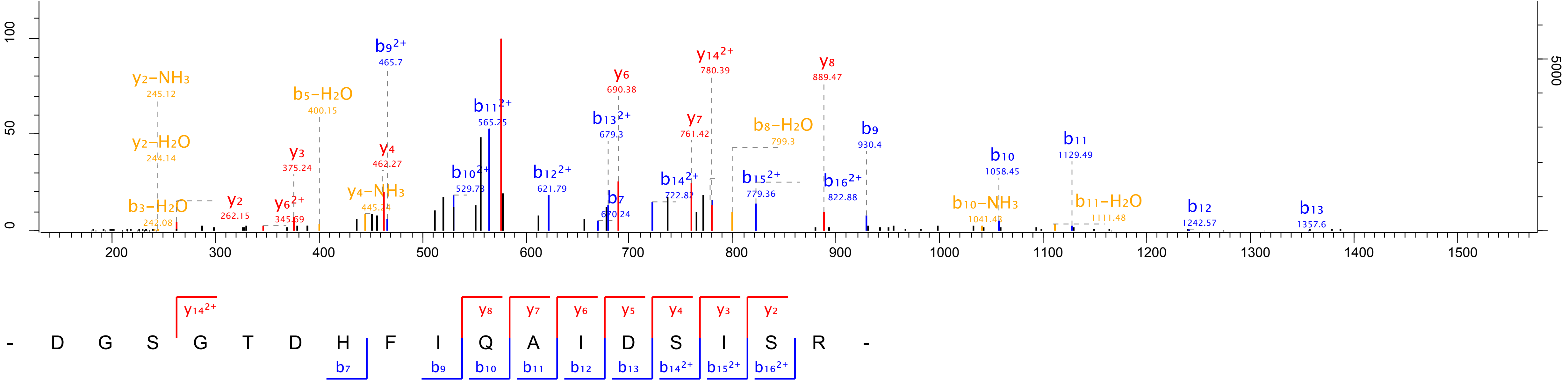

Raw file	Scan	Method	Score	m/z	Gene names
HBT_20130723_BV2_LPS_1_002	24650	ITMS; CID	111.66	897.11	Lair1

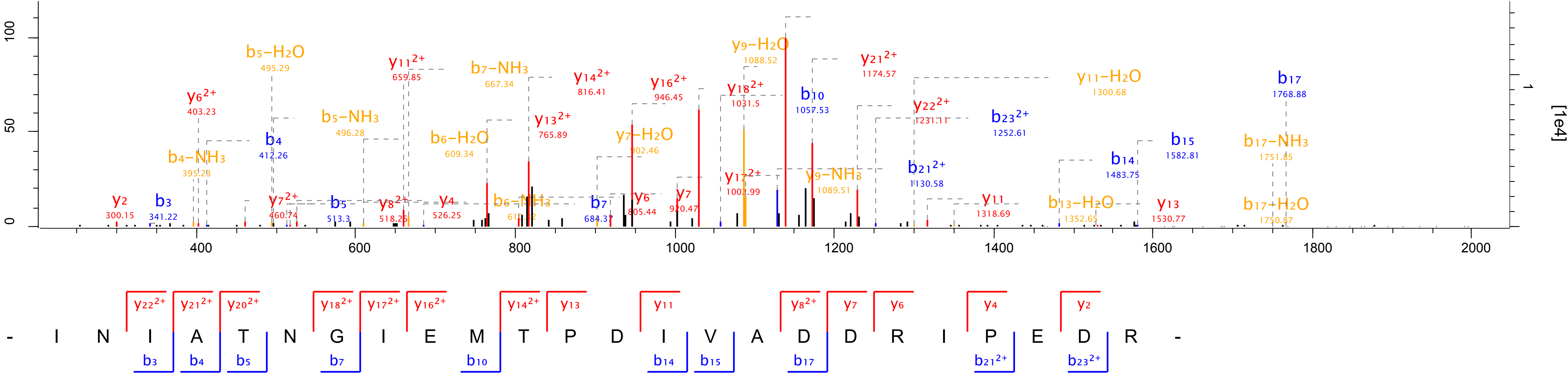

Raw file	Scan	Method	Score	m/z	Gene names
HBT_20130723_BV2_LPS_1_002	23849	ITMS; CID	106.02	965.1	Tab1

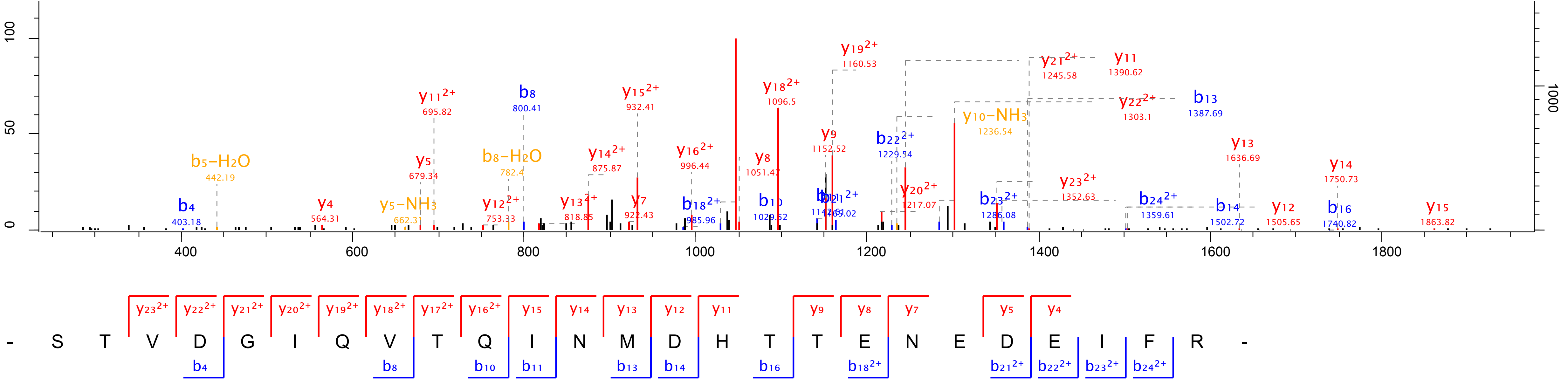

Raw file	Scan	Method	Score	m/z	Gene names
HBT_20130723_BV2_LPS_1_002	23181	ITMS; CID	212.83	703.88	Sirt2

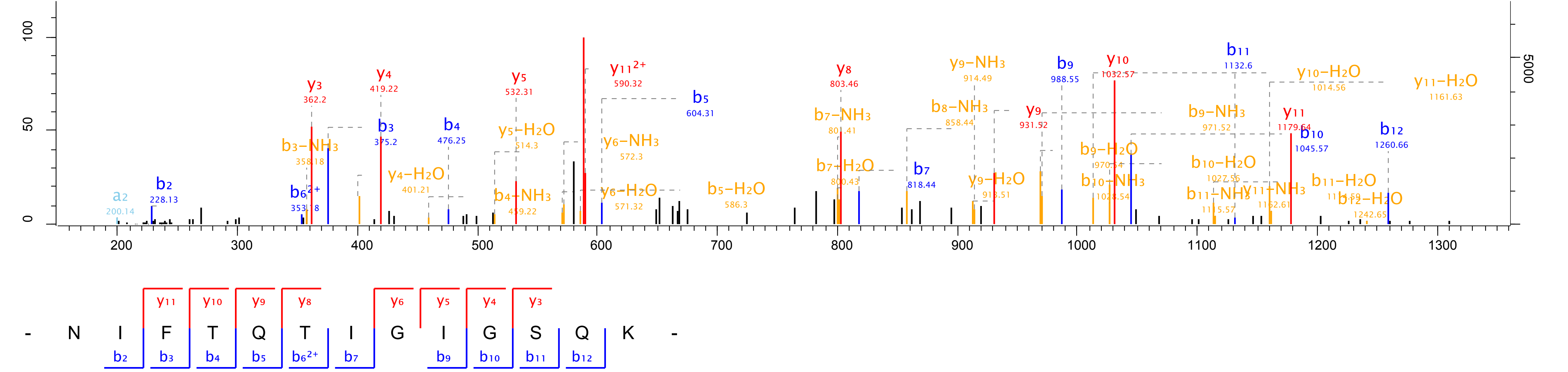

Raw file	Scan	Method	Score	m/z	Gene names
HBT_20130723_BV2_LPS_1_002	22746	ITMS; CID	137.9	1108.84	Cebpg

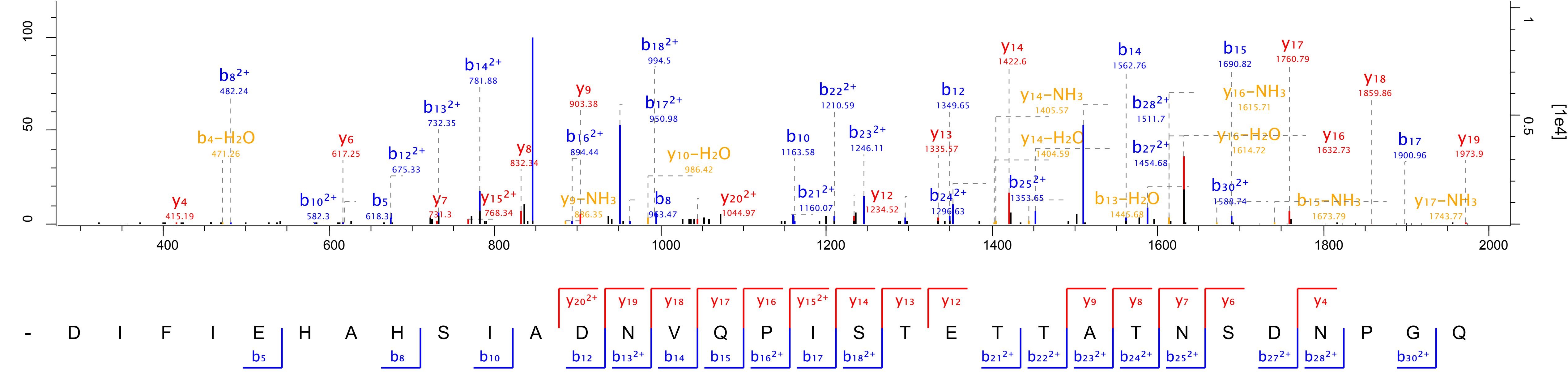

Raw file	Scan	Method	Score	m/z	Gene names
HBT_20130723_BV2_LPS_1_002	20743	ITMS; CID	160.52	736.87	Gpaa1

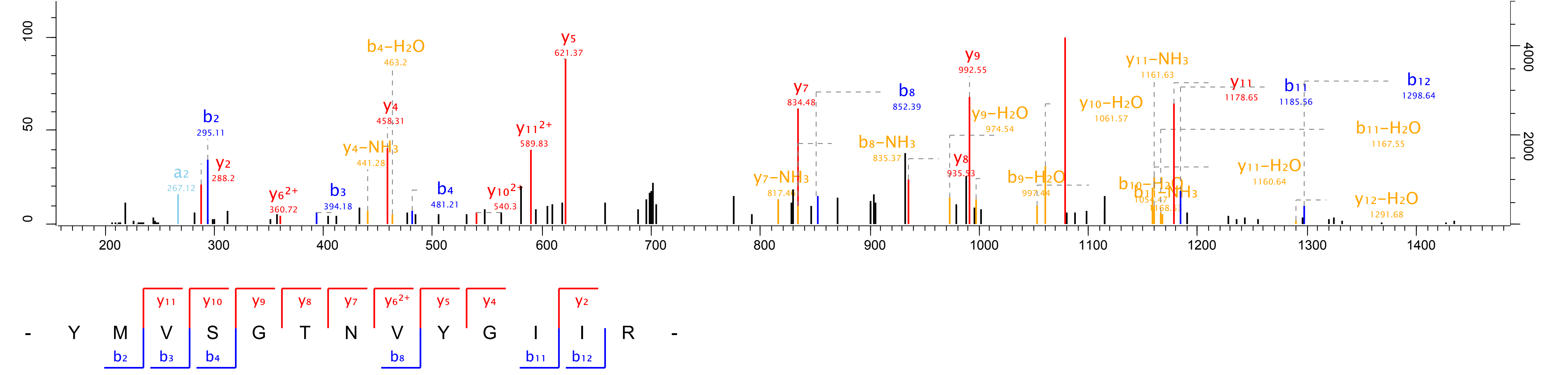

Raw file	Scan	Method	Score	m/z	Gene names
HBT_20130723_BV2_LPS_1_002	19193	ITMS; CID	153.2	944.42	Calu

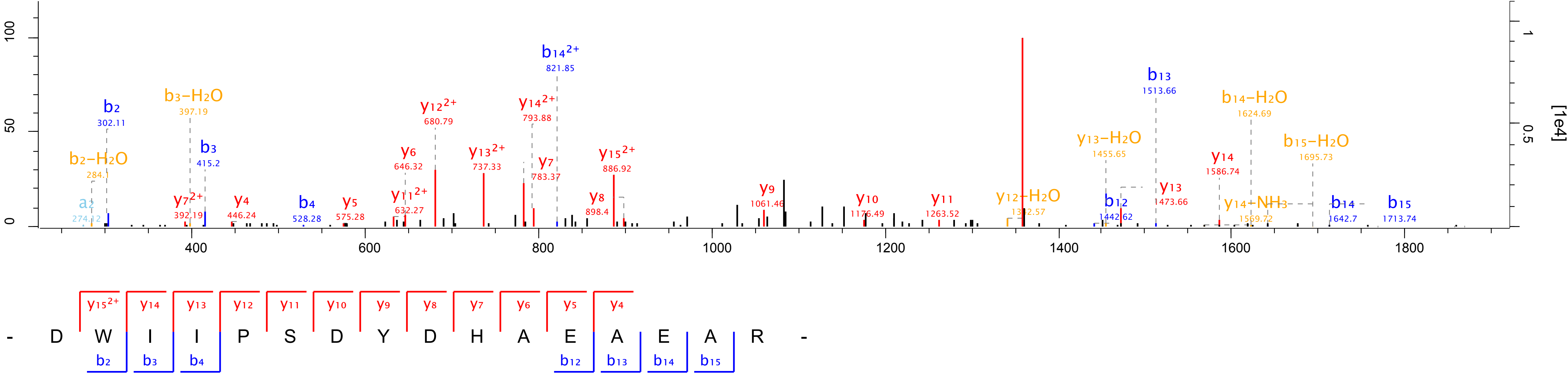

Raw file	Scan	Method	Score	m/z	Gene names
HBT_20130723_BV2_LPS_1_002	1569	ITMS; CID	189.17	723.75	Mt2

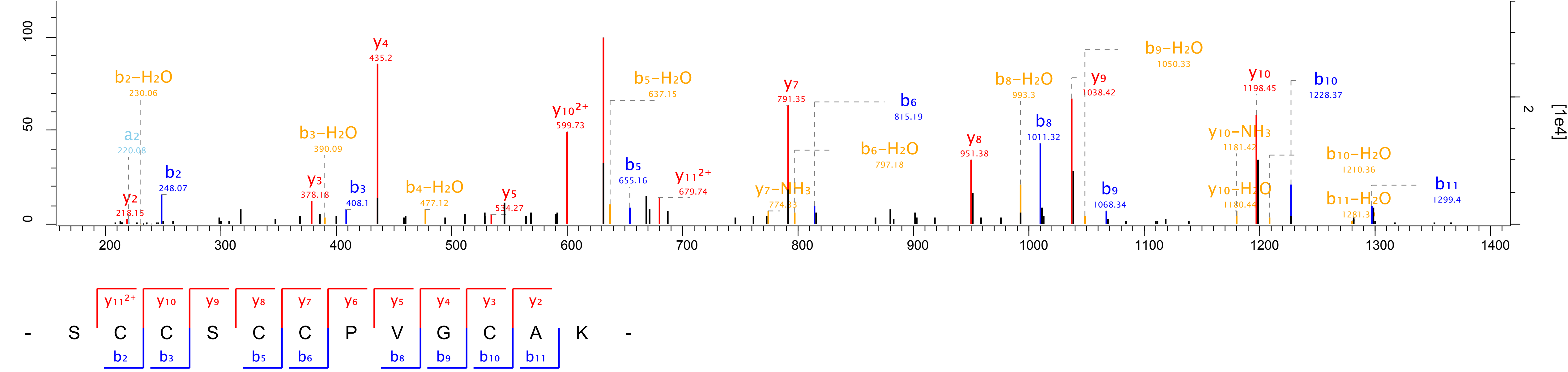

Raw file	Scan	Method	Score	m/z	Gene names
HBT_20130723_BV2_LPS_1_002	1426	ITMS; CID	64.84	585.56	Asxl1

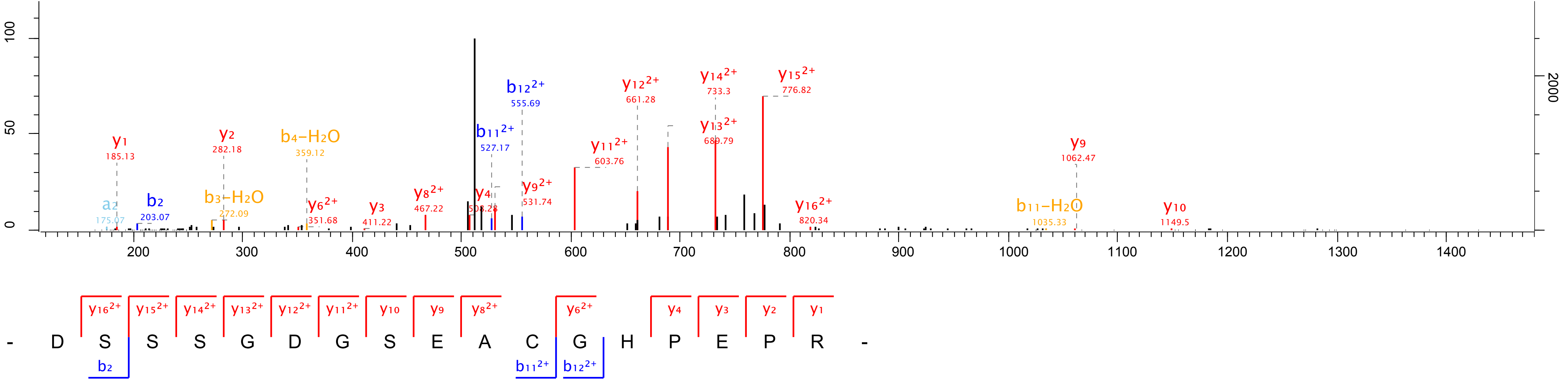

Raw file	Scan	Method	Score	m/z	Gene names
HBT_20130723_BV2_LPS_1_002	1300	ITMS; CID	138.01	533.91	Akap12

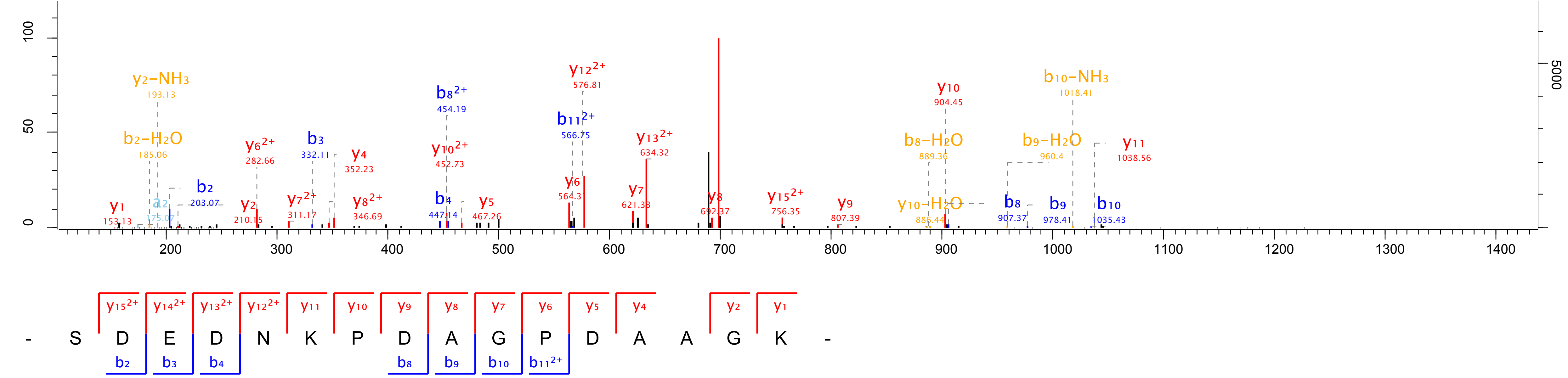

Raw file	Scan	Method	Score	m/z	Gene names
HBT_20130723_BV2_LPS_1_002	12983	ITMS; CID	58.11	895.76	Bcl7b

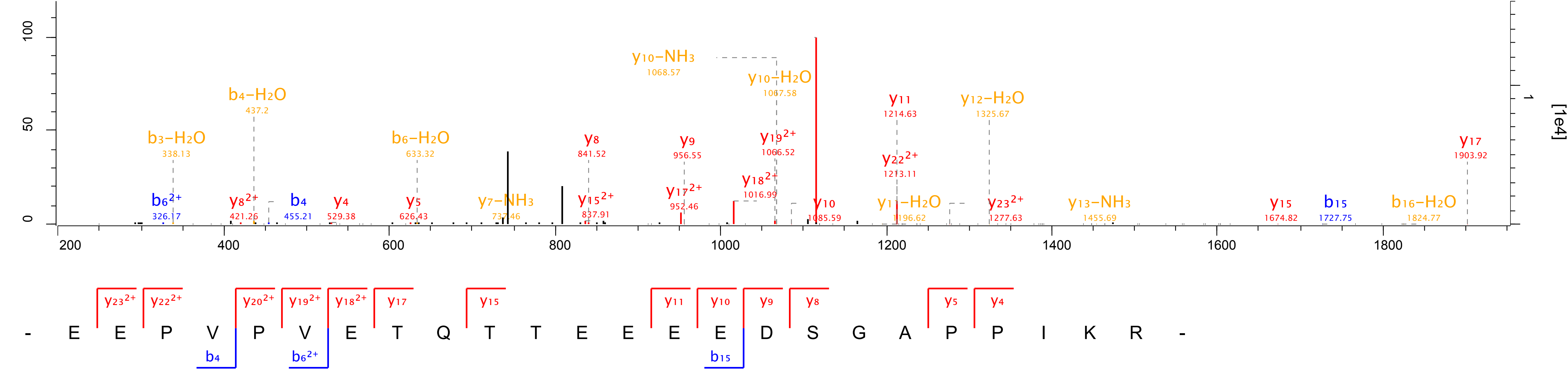

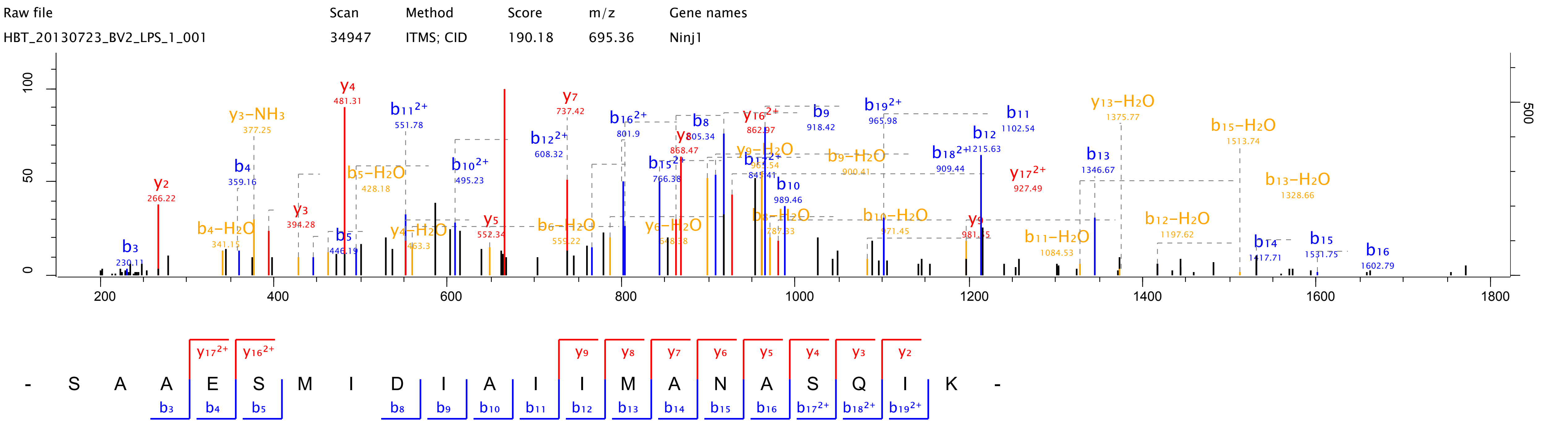
Raw file
HBT_20130723_BV2_LPS_1_001
Scan
34947
Method
ITMS; CID
Score
190.18
m/z
695.36
Gene names
Ninj1
100
50
0
500
200
400
600
800
1000
1200
1400
1600
1800
- S A A E S M I D I A I I M A N A S Q I K -

Raw file	Scan	Method	Score	m/z	Gene names
HBT_20130723_BV2_LPS_1_001	33302	ITMS; CID	62.71	905.12	Bsdc1

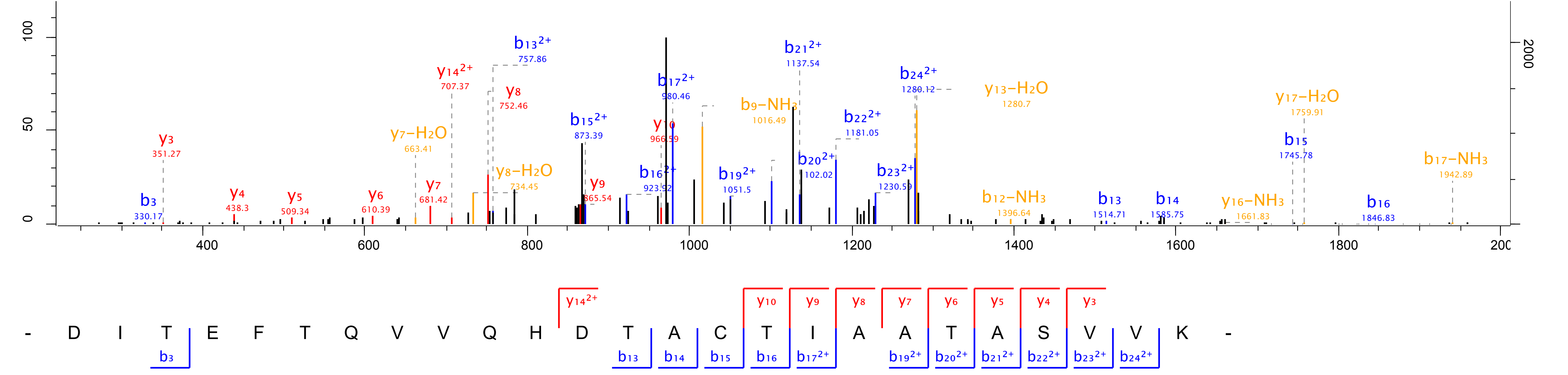

Raw file	Scan	Method	Score	m/z	Gene names
HBT_20130723_BV2_LPS_1_001	25615	ITMS; CID	155.39	803.93	Gtf2e1

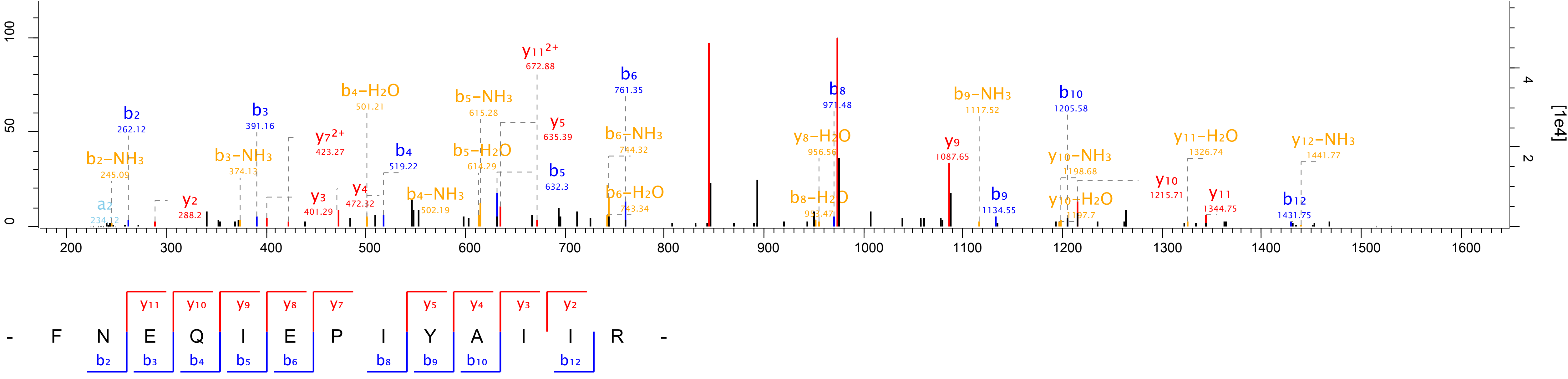

Raw file	Scan	Method	Score	m/z	Gene names
HBT_20130723_BV2_LPS_1_001	24889	ITMS; CID	109.77	1112.04	Mrps11

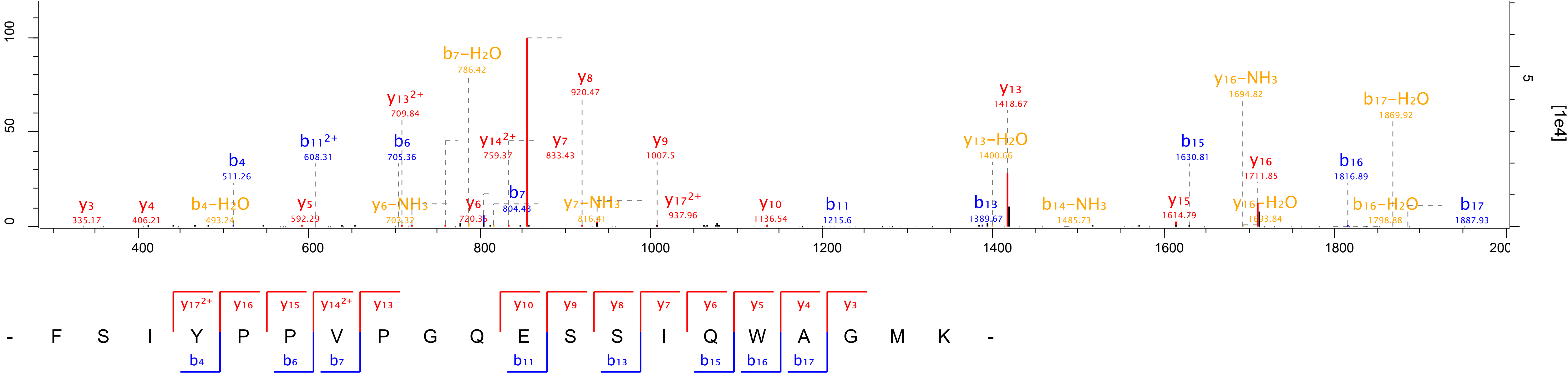

Raw file	Scan	Method	Score	m/z	Gene names
HBT_20130723_BV2_LPS_1_001	23175	ITMS; CID	112.13	629.83	Plcd3

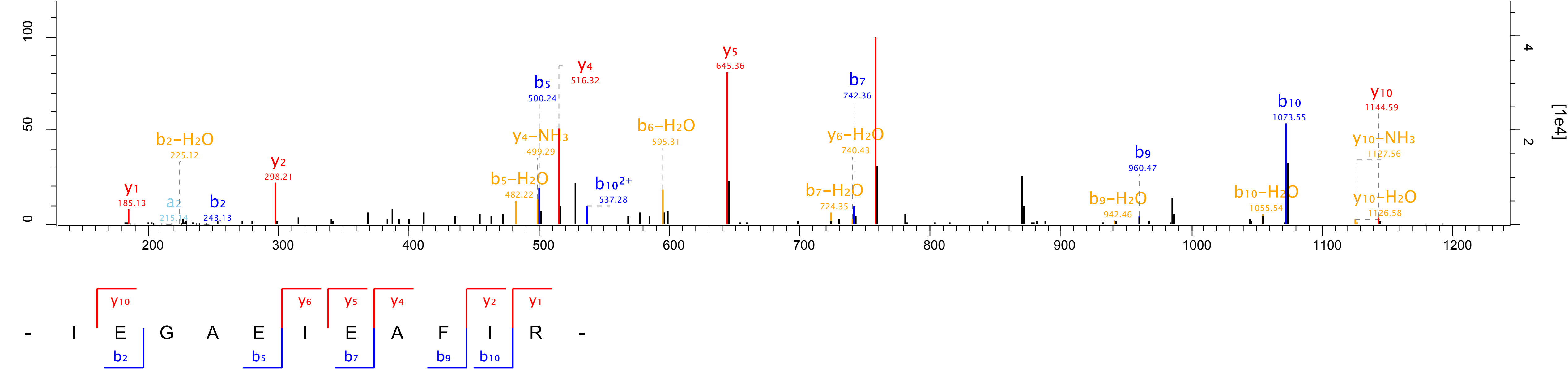

Raw file	Scan	Method	Score	m/z	Gene names
HBT_20130723_BV2_LPS_1_001	20442	ITMS; CID	124.62	767.91	Dr1

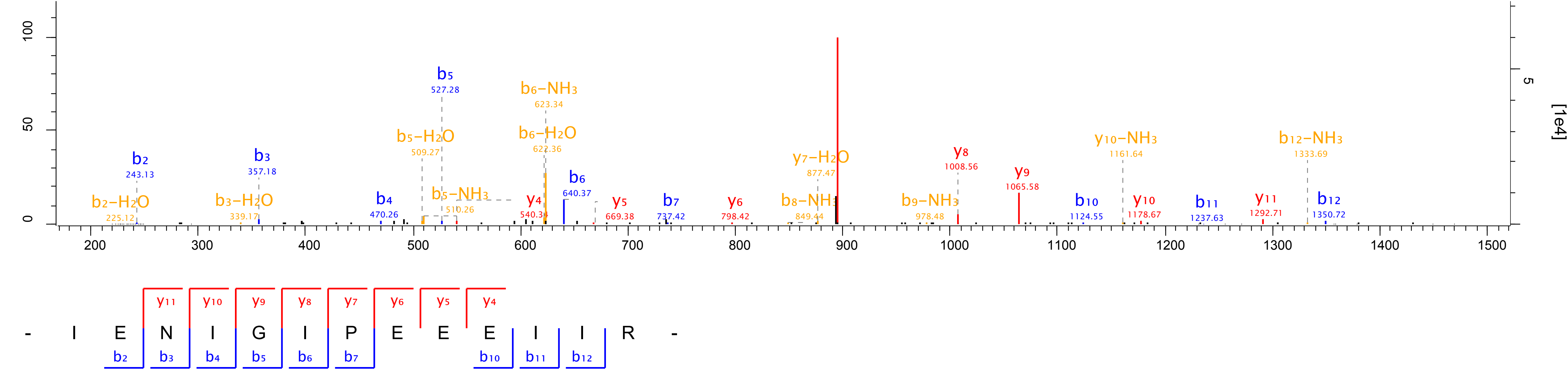

Raw file	Scan	Method	Score	m/z	Gene names
HBT_20130723_BV2_LPS_1_001	1604	ITMS; CID	142.76	601.27	Pik3cg

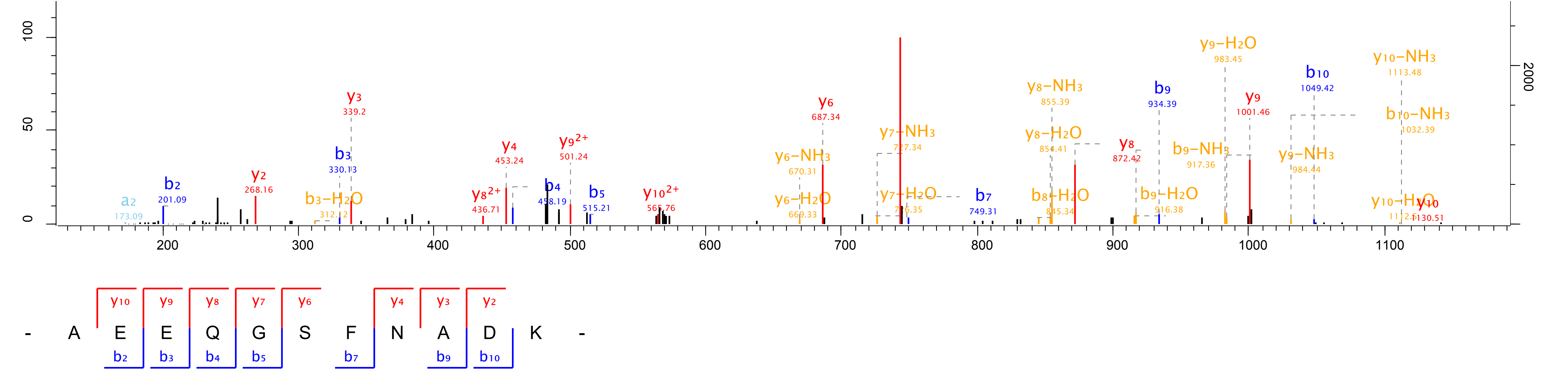

Raw file	Scan	Method	Score	m/z	Gene names
HBT_20130723_BV2_LPS_1_001	14821	ITMS; CID	135.55	946.95	Mrpl30

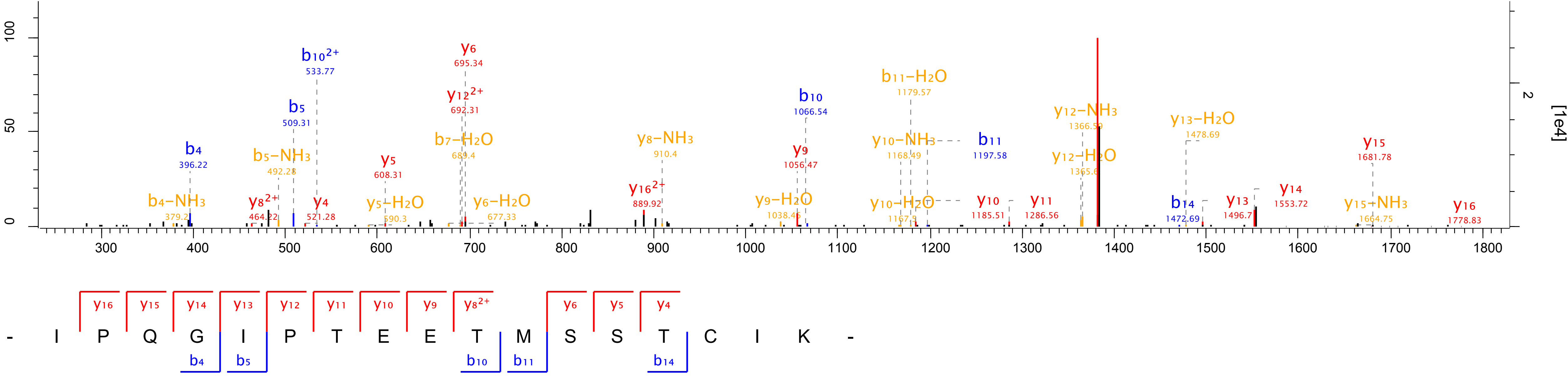

Raw file	Scan	Method	Score	m/z	Gene names
HBT_20130723_BV2_LPS_1_001	1475	ITMS; CID	169.93	511.26	Rabep2

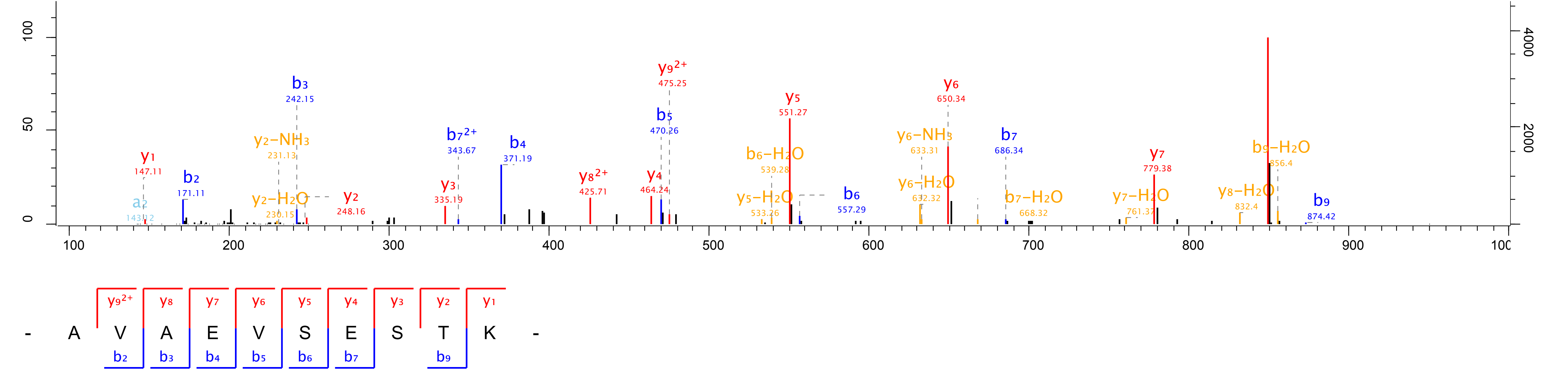

Supplement: Supplemental Data [file 10.1074_M115.053926_mcp.M115.053926-1.pdf]
