# Supplementary material for: Novel Molecular Insights into Classical and Alternative Activation States of Microglia as Revealed by Stable Isotope Labeling by Amino Acids in Cell Culture (SILAC)-based Proteomics
Source: Mol Cell Proteomics. 2015 Sep 30;14(12):3173–84. doi: 10.1074/mcp.M115.053926 (PMC4762627; doi:10.1074/mcp.M115.053926)
Supplement: Supplemental Data [file 10.1074_M115.053926_mcp.M115.053926-2.docx]

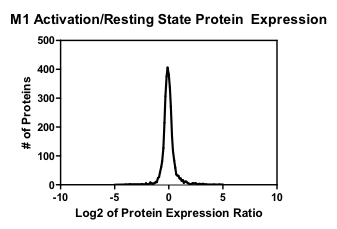

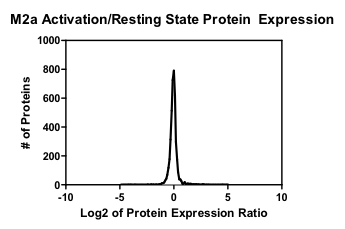


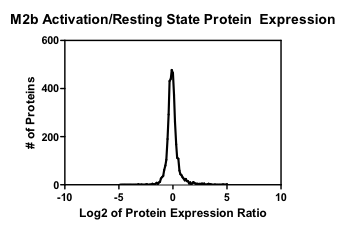

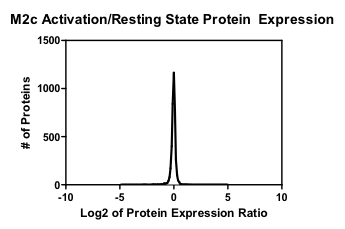


**Supplemental Figure 1: SILAC Protein Expression Ratio Distributions.** Log2 protein expression ratios for all 4 activation states compared to control.


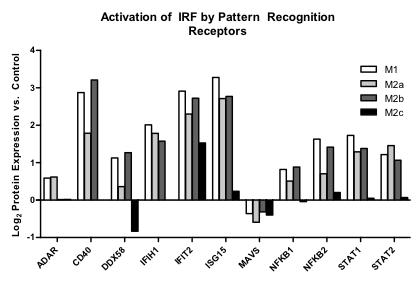


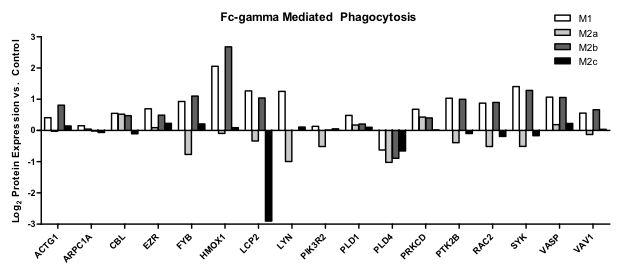


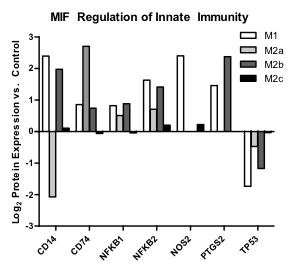


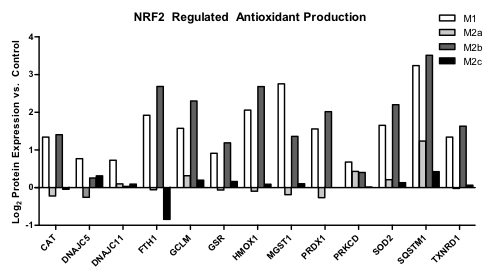


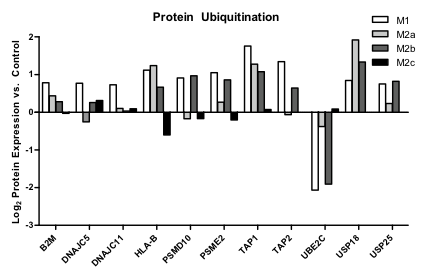


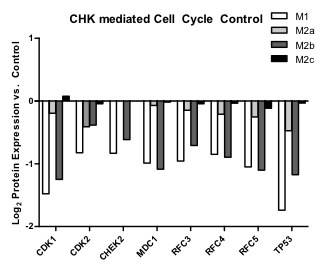


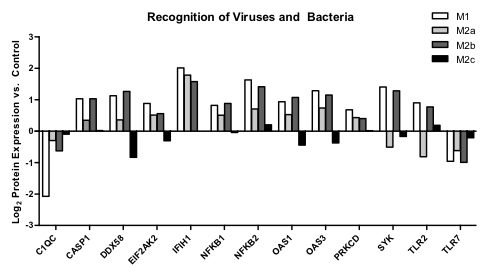


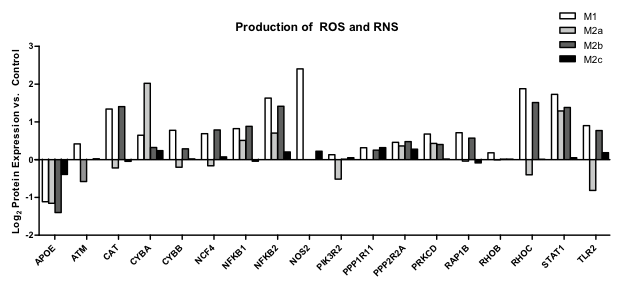


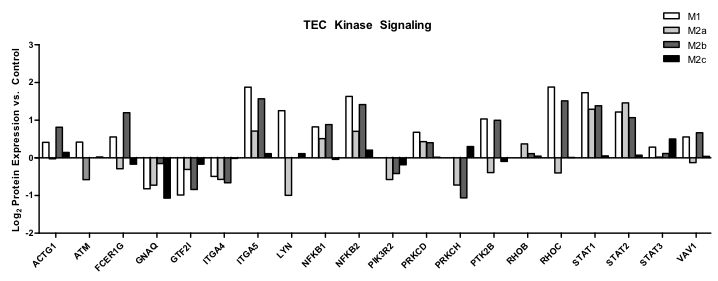


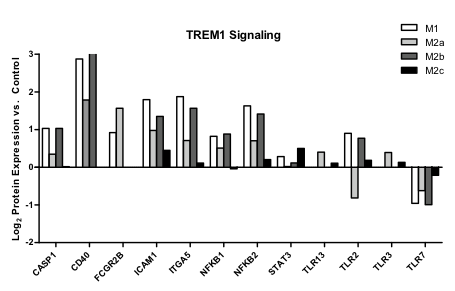


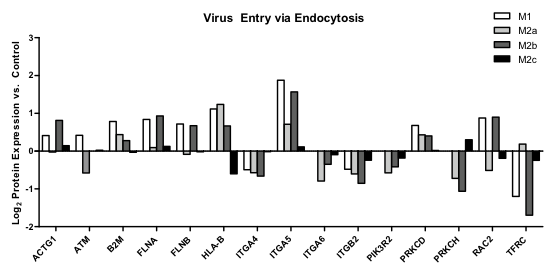


**Supplemental Figure 2: Selected Canonical Pathway Differential Protein Expression.** The bar charts above represent the log2 of treatment vs. control for various selected canonical pathways across all 4 microglial activation states.

**Supplemental Table 1: M1 State Differentially Expressed Proteins**. Protein Expression levels for all significant (p<0.05) proteins for the M1 activation state.

| Uniprot ID | Protein name | M1 |
| --- | --- | --- |
| A2A4Z1 | Ubiquitin-conjugating enzyme E2 C | 0.24 |
| A2AL85 | Aspartyl/asparaginyl beta-hydroxylase | 1.57 |
| A2APB8 | Targeting protein for Xklp2 | 0.30 |
| A2AR26 |  | 2.34 |
| A2AS05 |  | 3.07 |
| A2AWF8 | Receptor-type tyrosine-protein phosphatase eta | 2.64 |
| A6H5X4 | PHD finger protein 11 | 5.77 |
| A9XX86 |  | 0.33 |
| B1AQR8 | Galectin-9 | 5.12 |
| B1ASZ3 | Glycerol kinase | 1.72 |
| B1ATI9 | Growth arrest-specific protein 7 | 2.66 |
| B1AVY7 | Kinesin-like protein KIF16B | 1.64 |
| B1AZ46 | Brain-specific angiogenesis inhibitor 1-associated protein 2 | 1.59 |
| B7FAU9 | Filamin-A | 1.79 |
| B9EKJ3 | Protein MON2 homolog | 0.08 |
| D3YTU2 | 2-oxoglutarate and iron-dependent oxygenase domain-containing protein 1 | 1.71 |
| D3YU00 | StAR-related lipid transfer protein 5 | 2.00 |
| D3YU60 | Microsomal glutathione S-transferase 1 | 6.74 |
| D3YXD1 | Ubiquitin-conjugating enzyme E2 E2 | 3.03 |
| D3Z016 | Proteasome assembly chaperone 4 | 0.31 |
| D3Z131 | THO complex subunit 6 homolog | 2.13 |
| D3Z3S1 | Prolactin regulatory element-binding protein | 1.74 |
| D3Z3X7 | GPN-loop GTPase 1 | 0.56 |
| D3Z585 | Guanine nucleotide exchange factor for Rab-3A | 0.37 |
| D3Z5I1 | Zinc finger CCCH-type antiviral protein 1 | 1.57 |
| D3Z6B9 | Mitochondrial 10-formyltetrahydrofolate dehydrogenase | 8.53 |
| D6RCJ3 | Tripartite motif-containing protein 12A | 1.81 |
| D6RFN5 | Ninjurin-1 | 2.51 |
| E0CY74 |  | 5.16 |
| E9PVX6 |  | 0.23 |
| E9PW03 | Glycosylphosphatidylinositol anchor attachment 1 protein | 2.76 |
| E9PW15 | Polycomb protein Suz12 | 0.53 |
| E9PWG6 |  | 0.50 |
| E9Q1M6 |  | 0.26 |
| E9Q1P8 | Interferon regulatory factor 2-binding protein 2 | 0.54 |
| E9Q223 | Hemoglobin subunit beta-1 | 0.24 |
| E9Q368 |  | 2.06 |
| E9Q415 | Low affinity immunoglobulin gamma Fc region receptor II | 1.89 |
| E9Q4G8 | CD166 antigen | 2.37 |
| E9Q4K7 |  | 10.41 |
| E9Q555 | E3 ubiquitin-protein ligase RNF213 | 3.00 |
| E9Q7H6 | Threonine--tRNA ligase, mitochondrial | 1.77 |
| E9Q8I7 |  | 0.08 |
| E9Q9E4 | Cyclin-dependent kinase 4 | 0.48 |
| E9QA47 | CUGBP Elav-like family member 2 | 0.54 |
| E9QKL6 |  | 3.93 |
| E9QN37 | Macrophage-expressed gene 1 protein | 3.53 |
| E9QPE7 | Myosin-11 | 4.88 |
| F6QP10 | Sn1-specific diacylglycerol lipase beta | 1.66 |
| F6SMY7 | Probable E3 ubiquitin-protein ligase MYCBP2 | 1.93 |
| F6TLV3 | Fibronectin type-III domain-containing protein 3A | 2.65 |
| F6YBY4 | Integrin alpha-M | 1.84 |
| F7CVJ5 |  | 2.80 |
| F7DBB3 |  | 3.24 |
| F8VQC9 | Sodium bicarbonate cotransporter 3 | 2.05 |
| F8WHZ9 | Alpha-adducin | 0.56 |
| F8WJ72 | Cell division control protein 45 homolog | 0.44 |
| G3UX50 | Death domain-associated protein 6 | 2.05 |
| G3UYD0 | General transcription factor II-I | 0.50 |
| G3UYZ1 | Immunoglobulin superfamily member 8 | 2.35 |
| G3X8Y8 | Toll-like receptor | 1.87 |
| G3X963 | ATPase family AAA domain-containing protein 2 | 0.57 |
| G3X9V0 | Proteasome activator complex subunit 2 | 2.07 |
| G5E850 | Cytochrome b5 | 1.67 |
| G8JL74 | Polypyrimidine tract-binding protein 3 | 2.55 |
| H3BJ51 | All-trans-retinol 13,14-reductase | 0.50 |
| H3BK44 | cTAGE family member 5 | 1.77 |
| H9KUZ3 | Sialoadhesin | 2.68 |
| I7HIK9 | Cellular tumor antigen p53 | 0.30 |
| J3QNT2 | Transcription initiation factor IIA subunit 1;Transcription initiation factor IIA alpha chain;Transcription initiation factor IIA beta chain | 1.55 |
| J3QP81 | CLIP-associating protein 1 | 2.10 |
| J3QPY0 | Protein C19orf12 homolog | 5.00 |
| O08739 | AMP deaminase 3 | 7.49 |
| O08804 |  | 2.17 |
| O09172 | Glutamate--cysteine ligase regulatory subunit | 2.98 |
| O35309 | N-myc-interactor | 2.09 |
| O35316 | Sodium- and chloride-dependent taurine transporter | 0.44 |
| O35601 | FYN-binding protein | 1.91 |
| O35604 | Niemann-Pick C1 protein | 1.60 |
| O54782 | Epididymis-specific alpha-mannosidase | 2.78 |
| P01831 | Thy-1 membrane glycoprotein | 0.17 |
| P01887 | Beta-2-microglobulin | 1.72 |
| P01899 | H-2 class I histocompatibility antigen, D-B alpha chain | 2.17 |
| P01901 | H-2 class I histocompatibility antigen, K-B alpha chain | 1.93 |
| P04441 | H-2 class II histocompatibility antigen gamma chain | 1.81 |
| P06801 | NADP-dependent malic enzyme | 1.72 |
| P07356 | Annexin A2 | 2.22 |
| P07742 | Ribonucleoside-diphosphate reductase large subunit | 0.35 |
| P08226 | Apolipoprotein E | 0.46 |
| P09242 | Alkaline phosphatase, tissue-nonspecific isozyme | 0.39 |
| P09528 | Ferritin heavy chain | 3.78 |
| P09671 | Superoxide dismutase [Mn], mitochondrial | 3.14 |
| P10605 | Cathepsin B;Cathepsin B light chain;Cathepsin B heavy chain | 2.12 |
| P10810 | Monocyte differentiation antigen CD14 | 5.27 |
| P10852 | 4F2 cell-surface antigen heavy chain | 1.76 |
| P11157 | Ribonucleoside-diphosphate reductase subunit M2 | 0.33 |
| P11440 | Cyclin-dependent kinase 1 | 0.36 |
| P11688 | Integrin alpha-5;Integrin alpha-5 heavy chain;Integrin alpha-5 light chain | 3.67 |
| P11928 | 2-5-oligoadenylate synthase 1A | 1.91 |
| P13011 | Acyl-CoA desaturase 2 | 0.50 |
| P13597 | Intercellular adhesion molecule 1 | 3.47 |
| P13864 | DNA (cytosine-5)-methyltransferase 1 | 0.52 |
| P14733 | Lamin-B1 | 0.57 |
| P14901 | Heme oxygenase 1 | 4.16 |
| P15864 | Histone H1.2 | 0.48 |
| P19182 | Interferon-related developmental regulator 1 | 3.52 |
| P19426 | Negative elongation factor E | 25.43 |
| P19973 | Lymphocyte-specific protein 1 | 0.33 |
| P20152 | Vimentin | 2.22 |
| P20664 | DNA primase small subunit | 0.53 |
| P21279 | Guanine nucleotide-binding protein G(q) subunit alpha | 0.57 |
| P21460 | Cystatin-C | 0.50 |
| P24270 | Catalase | 2.53 |
| P24860 | G2/mitotic-specific cyclin-B1 | 0.28 |
| P25799 | Nuclear factor NF-kappa-B p105 subunit;Nuclear factor NF-kappa-B p50 subunit | 1.77 |
| P25911 | Tyrosine-protein kinase Lyn | 2.38 |
| P26040 | Ezrin | 1.62 |
| P26041 | Moesin | 2.04 |
| P26323 | Friend leukemia integration 1 transcription factor | 0.50 |
| P26645 | Myristoylated alanine-rich C-kinase substrate | 1.72 |
| P27046 | Alpha-mannosidase 2 | 2.02 |
| P27512 | Tumor necrosis factor receptor superfamily member 5 | 7.33 |
| P28667 | MARCKS-related protein | 3.32 |
| P28867 | Protein kinase C delta type | 1.60 |
| P29452 | Caspase-1;Caspase-1 subunit p20;Caspase-1 subunit p10 | 2.05 |
| P29477 | Nitric oxide synthase, inducible | 5.29 |
| P30204 | Macrophage scavenger receptor types I and II | 2.94 |
| P32921 | Tryptophan--tRNA ligase, cytoplasmic;T1-TrpRS;T2-TrpRS | 1.55 |
| P33174 | Chromosome-associated kinesin KIF4 | 0.36 |
| P33609 | DNA polymerase alpha catalytic subunit | 0.54 |
| P33610 | DNA primase large subunit | 0.44 |
| P35700 | Peroxiredoxin-1 | 2.94 |
| P35761 | Dual specificity protein kinase TTK | 7.10 |
| P35951 | Low-density lipoprotein receptor | 1.76 |
| P36371 | Antigen peptide transporter 2 | 2.54 |
| P37040 | NADPH--cytochrome P450 reductase | 1.66 |
| P40237 | CD82 antigen | 2.30 |
| P41216 | Long-chain-fatty-acid--CoA ligase 1 | 2.07 |
| P41230 | Lysine-specific demethylase 5C | 0.06 |
| P41233 | ATP-binding cassette sub-family A member 1 | 1.65 |
| P43274 | Histone H1.4 | 0.55 |
| P43276 | Histone H1.5 | 0.46 |
| P43277 | Histone H1.3 | 0.49 |
| P47791 | Glutathione reductase, mitochondrial | 1.88 |
| P47856 | Glutamine--fructose-6-phosphate aminotransferase [isomerizing] 1 | 1.63 |
| P48025 | Tyrosine-protein kinase SYK | 2.65 |
| P48036 | Annexin A5 | 1.60 |
| P49282 | Natural resistance-associated macrophage protein 2 | 4.79 |
| P49710 | Hematopoietic lineage cell-specific protein | 2.14 |
| P52293 | Importin subunit alpha-2 | 0.31 |
| P54227 | Stathmin | 0.52 |
| P54729 | NEDD8 ultimate buster 1 | 1.98 |
| P54987 | Immune-responsive gene 1 protein | 18.15 |
| P55937 | Golgin subfamily A member 3 | 2.20 |
| P57080 | Ubiquitin carboxyl-terminal hydrolase 25 | 1.68 |
| P58058 | NAD kinase | 2.28 |
| P58681 | Toll-like receptor 7 | 0.51 |
| P60202 | Myelin proteolipid protein | 0.19 |
| P60904 | DnaJ homolog subfamily C member 5 | 1.70 |
| P61027 | Ras-related protein Rab-10 | 1.56 |
| P61028 | Ras-related protein Rab-8B | 4.27 |
| P62774 | Myotrophin | 1.59 |
| P70460 | Vasodilator-stimulated phosphoprotein | 2.10 |
| P97329 | Kinesin-like protein KIF20A | 0.28 |
| P97333 | Neuropilin-1 | 0.46 |
| P97369 | Neutrophil cytosol factor 4 | 1.61 |
| P97377 | Cyclin-dependent kinase 2 | 0.56 |
| P97429 | Annexin A4 | 1.75 |
| P98078 | Disabled homolog 2 | 0.55 |
| P99029 | Peroxiredoxin-5, mitochondrial | 3.07 |
| Q00612 | Glucose-6-phosphate 1-dehydrogenase X | 1.77 |
| Q01320 | DNA topoisomerase 2-alpha | 0.31 |
| Q02105 | Complement C1q subcomponent subunit C | 0.24 |
| Q03963 | Interferon-induced, double-stranded RNA-activated protein kinase | 1.84 |
| Q04447 | Creatine kinase B-type | 0.36 |
| Q04899 | Cyclin-dependent kinase 18 | 1.76 |
| Q05144 | Ras-related C3 botulinum toxin substrate 2 | 1.83 |
| Q05769 | Prostaglandin G/H synthase 2 | 2.75 |
| Q07076 | Annexin A7 | 1.67 |
| Q08619 | Interferon-activable protein 205-B | 8.44 |
| Q08639 | Transcription factor Dp-1 | 0.41 |
| Q09200 | Beta-1,4 N-acetylgalactosaminyltransferase 1 | 0.52 |
| Q14AI0 | Sister chromatid cohesion protein DCC1 | 0.56 |
| Q2EMV9 | Poly [ADP-ribose] polymerase 14 | 2.38 |
| Q3T9A5 | Glomulin | 0.17 |
| Q3TBA3 | Antigen peptide transporter 1 | 3.38 |
| Q3TBV5 | Interleukin-1 receptor antagonist protein | 11.36 |
| Q3TIR3 | Synembryn-A | 1.88 |
| Q3TJZ6 | Protein FAM98A | 0.43 |
| Q3TRM8 | Hexokinase-3 | 2.30 |
| Q3U0X8 | T-cell surface protein tactile | 1.69 |
| Q3U4X8 | DNA ligase | 0.49 |
| Q3U5Q7 | UMP-CMP kinase 2, mitochondrial | 7.05 |
| Q3U7R1 | Extended synaptotagmin-1 | 2.15 |
| Q3UDE9 | Protein-tyrosine kinase 2-beta | 2.05 |
| Q3UDK1 | TRAF-type zinc finger domain-containing protein 1 | 2.32 |
| Q3UFB2 | Box C/D snoRNA protein 1 | 0.54 |
| Q3UGL1 | Epidermal growth factor receptor kinase substrate 8 | 0.54 |
| Q3UGP9 | Leucine-rich repeat-containing protein 58 | 0.22 |
| Q3UIR3 | E3 ubiquitin-protein ligase DTX3L | 2.27 |
| Q3ULB1 | Testin | 1.86 |
| Q3ULJ0 | Glycerol-3-phosphate dehydrogenase 1-like protein | 1.86 |
| Q3UMQ8 | H/ACA ribonucleoprotein complex non-core subunit NAF1 | 0.53 |
| Q3UND0 | Src kinase-associated phosphoprotein 2 | 1.85 |
| Q3UU41 | SLP adapter and CSK-interacting membrane protein | 2.81 |
| Q4FZC9 | Nesprin-3 | 0.41 |
| Q4KL52 | Zinc finger FYVE domain-containing protein 19 | 4.45 |
| Q4KMM3 | Oxidation resistance protein 1 | 2.07 |
| Q4LDD4 | Arf-GAP with Rho-GAP domain, ANK repeat and PH domain-containing protein 1 | 2.21 |
| Q52KC3 | DNA replication licensing factor MCM5 | 0.56 |
| Q543K9 | Purine nucleoside phosphorylase | 2.04 |
| Q571H0 | Nucleolar pre-ribosomal-associated protein 1 | 2.68 |
| Q5NC05 | Transcription termination factor 2 | 1.61 |
| Q5PSV9 | Mediator of DNA damage checkpoint protein 1 | 0.50 |
| Q5SF07 | Insulin-like growth factor 2 mRNA-binding protein 2 | 2.03 |
| Q5U458 | DnaJ homolog subfamily C member 11 | 1.66 |
| Q60591 | Nuclear factor of activated T-cells, cytoplasmic 2 | 0.55 |
| Q60710 | SAM domain and HD domain-containing protein 1 | 2.01 |
| Q60787 | Lymphocyte cytosolic protein 2 | 2.41 |
| Q60848 | Lymphocyte-specific helicase | 0.39 |
| Q60972 | Histone-binding protein RBBP4 | 0.57 |
| Q61093 | Cytochrome b-245 heavy chain | 1.71 |
| Q61107 | Guanylate-binding protein 4 | 6.13 |
| Q61127 | NGFI-A-binding protein 2 | 0.44 |
| Q61285 | ATP-binding cassette sub-family D member 2 | 0.45 |
| Q61333 | Tumor necrosis factor alpha-induced protein 2 | 3.12 |
| Q61462 | Cytochrome b-245 light chain | 1.56 |
| Q61543 | Golgi apparatus protein 1 | 0.54 |
| Q61549 | EGF-like module-containing mucin-like hormone receptor-like 1 | 1.61 |
| Q61635 |  | 1.59 |
| Q61686 | Chromobox protein homolog 5 | 0.52 |
| Q61735 | Leukocyte surface antigen CD47 | 2.39 |
| Q62018 | RNA polymerase-associated protein CTR9 homolog | 0.56 |
| Q62048 | Astrocytic phosphoprotein PEA-15 | 1.84 |
| Q62159 | Rho-related GTP-binding protein RhoC | 3.67 |
| Q62176 | RNA-binding protein 38 | 0.46 |
| Q62351 | Transferrin receptor protein 1 | 0.43 |
| Q641P0 | Actin-related protein 3B | 0.53 |
| Q64281 | Leukocyte immunoglobulin-like receptor subfamily B member 4 | 1.99 |
| Q64282 | Interferon-induced protein with tetratricopeptide repeats 1 | 6.38 |
| Q64337 | Sequestosome-1 | 9.44 |
| Q64339 | Ubiquitin-like protein ISG15 | 9.70 |
| Q64345 | Interferon-induced protein with tetratricopeptide repeats 3 | 6.66 |
| Q64695 | Endothelial protein C receptor | 2.15 |
| Q69Z37 | Sterile alpha motif domain-containing protein 9-like | 5.09 |
| Q69ZN7 | Myoferlin | 1.56 |
| Q6GTM0 | Interferon-induced protein with tetratricopeptide repeats 2 | 7.53 |
| Q6IRU2 | Tropomyosin alpha-4 chain | 2.24 |
| Q6NV52 | Transforming acidic coiled-coil-containing protein 3 | 0.40 |
| Q6NXL1 |  | 1.74 |
| Q6P9P6 | Kinesin-like protein KIF11 | 0.30 |
| Q6P9Q4 | FH1/FH2 domain-containing protein 1 | 0.50 |
| Q6P9Q6 | FK506-binding protein 15 | 2.07 |
| Q6PB44 | Tyrosine-protein phosphatase non-receptor type 23 | 1.57 |
| Q6PFQ7 | Ras GTPase-activating protein 4 | 4.84 |
| Q6PHN7 | Transmembrane protein 164 | 1.66 |
| Q6Q899 | Probable ATP-dependent RNA helicase DDX58 | 2.18 |
| Q6WVG3 | BTB/POZ domain-containing protein KCTD12 | 2.51 |
| Q6XAS3 |  | 0.53 |
| Q6ZQM8 | UDP-glucuronosyltransferase 1-7C | 2.52 |
| Q6ZWY8 | Thymosin beta-10 | 2.38 |
| Q7TMM9 | Tubulin beta-2A chain | 1.68 |
| Q80SU7 | Interferon-induced very large GTPase 1 | 3.18 |
| Q80X90 | Filamin-B | 1.64 |
| Q80X95 | Ras-related GTP-binding protein A | 1.64 |
| Q8BGQ7 | Alanine--tRNA ligase, cytoplasmic | 1.64 |
| Q8BGR9 | Ubiquitin-like domain-containing CTD phosphatase 1 | 2.34 |
| Q8BHZ0 | Protein FAM49A | 2.77 |
| Q8BL66 | Early endosome antigen 1 | 1.69 |
| Q8BMI4 | Flap endonuclease GEN homolog 1 | 0.52 |
| Q8BPX9 | Solute carrier family 15 member 3 | 6.09 |
| Q8BR63 | Protein FAM177A1 | 2.05 |
| Q8BT07 | Centrosomal protein of 55 kDa | 0.31 |
| Q8BTV2 | Cleavage and polyadenylation specificity factor subunit 7 | 1.64 |
| Q8BU33 | Acetolactate synthase-like protein | 1.75 |
| Q8BV49 | Pyrin and HIN domain-containing protein 1 | 9.01 |
| Q8BVK9 | Sp110 nuclear body protein | 3.46 |
| Q8BWZ3 | N-alpha-acetyltransferase 25, NatB auxiliary subunit | 1.58 |
| Q8BZN6 | Dedicator of cytokinesis protein 10 | 1.87 |
| Q8C1E7 | Transmembrane protein 120A | 1.76 |
| Q8C2T6 | DNA polymerase alpha subunit B | 0.51 |
| Q8C3V4 | Signal transducer and activator of transcription 1 | 3.31 |
| Q8C3Y4 | Kinetochore-associated protein 1 | 0.56 |
| Q8CAS9 | Poly [ADP-ribose] polymerase 9 | 2.30 |
| Q8CBB9 | Radical S-adenosyl methionine domain-containing protein 2 | 9.15 |
| Q8CD15 | Bifunctional lysine-specific demethylase and histidyl-hydroxylase MINA | 1.95 |
| Q8CFB4 | Guanylate-binding protein 5 | 3.13 |
| Q8CFB8 |  | 1.61 |
| Q8CGP2 | Histone H2B type 1-P;Histone H2B type 1-K;Histone H2B type 1-B;Histone H2B type 1-F/J/L;Histone H2B;Histone H2B type 3-A;Histone H2B type 3-B;Histone H2B type 1-A | 0.55 |
| Q8CHP5 | Partner of Y14 and mago | 0.44 |
| Q8JZR0 | Long-chain-fatty-acid--CoA ligase 5 | 1.58 |
| Q8K078 | Solute carrier organic anion transporter family member 4A1 | 1.88 |
| Q8K1T1 | Leucine-rich repeat-containing protein 25 | 4.66 |
| Q8K2Z4 | Condensin complex subunit 1 | 0.57 |
| Q8K337 | Type II inositol 1,4,5-trisphosphate 5-phosphatase | 1.87 |
| Q8K352 | SAM and SH3 domain-containing protein 3 | 0.56 |
| Q8K3G5 | Inactive serine/threonine-protein kinase VRK3 | 0.54 |
| Q8K4I3 | Rho guanine nucleotide exchange factor 6 | 2.11 |
| Q8K595 | Mucolipin-2 | 2.39 |
| Q8R151 | NFX1-type zinc finger-containing protein 1 | 1.90 |
| Q8R1S0 | Ubiquinone biosynthesis monooxygenase COQ6 | 0.57 |
| Q8R2Q8 | Bone marrow stromal antigen 2 | 2.83 |
| Q8R323 | Replication factor C subunit 3 | 0.52 |
| Q8R3D1 | TBC1 domain family member 13 | 1.55 |
| Q8R5F7 | Interferon-induced helicase C domain-containing protein 1 | 4.04 |
| Q8VBT6 | Apolipoprotein B receptor | 1.82 |
| Q8VC04 | Transmembrane protein 106A | 1.62 |
| Q8VDF2 | E3 ubiquitin-protein ligase UHRF1 | 0.34 |
| Q8VDQ8 | NAD-dependent protein deacetylase sirtuin-2 | 1.77 |
| Q8VEH6 | COBW domain-containing protein 1 | 0.43 |
| Q8VHK9 | Probable ATP-dependent RNA helicase DHX36 | 1.65 |
| Q8VHN8 | Protein syndesmos | 0.52 |
| Q8VI93 | 2-5-oligoadenylate synthase 3 | 2.44 |
| Q8VI94 | 2-5-oligoadenylate synthase-like protein 1 | 8.54 |
| Q91VC9 | Growth hormone-inducible transmembrane protein | 1.82 |
| Q91VL8 | Telomeric repeat-binding factor 2-interacting protein 1 | 0.54 |
| Q91VY9 | Zinc finger protein 622 | 1.64 |
| Q91WR3 | Activating signal cointegrator 1 complex subunit 2 | 1.83 |
| Q91XA2 | Golgi membrane protein 1 | 0.56 |
| Q91XB0 | Three prime repair exonuclease 1 | 3.00 |
| Q91XV3 | Brain acid soluble protein 1 | 1.72 |
| Q91YR9 | Prostaglandin reductase 1 | 1.88 |
| Q91Z96 | BMP-2-inducible protein kinase | 0.50 |
| Q91ZX7 | Prolow-density lipoprotein receptor-related protein 1 | 0.51 |
| Q920L1 | Fatty acid desaturase 1 | 0.52 |
| Q922S8 | Kinesin-like protein KIF2C | 0.23 |
| Q923D2 | Flavin reductase (NADPH) | 3.22 |
| Q925E7 | Serine/threonine-protein phosphatase 2A 55 kDa regulatory subunit B delta isoform | 0.03 |
| Q99J62 | Replication factor C subunit 4 | 0.56 |
| Q99J83 | Autophagy protein 5 | 1.79 |
| Q99JF8 | PC4 and SFRS1-interacting protein | 0.54 |
| Q99JI6 | Ras-related protein Rap-1b | 1.64 |
| Q99K51 | Plastin-3 | 2.18 |
| Q99K82 | Spermine oxidase | 1.81 |
| Q99KC8 | von Willebrand factor A domain-containing protein 5A | 1.67 |
| Q99KG3 | RNA-binding protein 10 | 0.41 |
| Q99KQ4 | Nicotinamide phosphoribosyltransferase | 1.69 |
| Q99LE1 | RILP-like protein 2 | 2.75 |
| Q99LI2 | Chloride channel CLIC-like protein 1 | 0.48 |
| Q99LW6 | YY1-associated factor 2 | 0.56 |
| Q99P72 | Reticulon-4 | 1.95 |
| Q9CQ43 |  | 0.39 |
| Q9CQE5 | Regulator of G-protein signaling 10 | 0.47 |
| Q9CQL7 | MORF4 family-associated protein 1 | 0.50 |
| Q9CQU5 | ZW10 interactor | 0.23 |
| Q9CQW9 | Interferon-induced transmembrane protein 3 | 3.27 |
| Q9CQX4 | PCNA-associated factor | 0.19 |
| Q9CQZ0 | ORM1-like protein 2;ORM1-like protein 1 | 2.15 |
| Q9CR02 | Translation machinery-associated protein 16 | 1.57 |
| Q9CR26 | Vacuolar protein sorting-associated protein VTA1 homolog | 2.00 |
| Q9CR80 | Protein FAM32A | 0.57 |
| Q9CRY7 | Glycerophosphodiester phosphodiesterase domain-containing protein 1 | 1.66 |
| Q9CWP6 | Motile sperm domain-containing protein 2 | 1.56 |
| Q9CZ15 | DNA replication complex GINS protein PSF1 | 0.50 |
| Q9CZL2 | Uncharacterized protein C4orf32 homolog | 1.55 |
| Q9D0D4 | Probable dimethyladenosine transferase | 0.52 |
| Q9D0F6 | Replication factor C subunit 5 | 0.48 |
| Q9D0N7 | Chromatin assembly factor 1 subunit B | 0.43 |
| Q9D154 | Leukocyte elastase inhibitor A | 0.45 |
| Q9D1A2 | Cytosolic non-specific dipeptidase | 1.68 |
| Q9D2Y4 | Mixed lineage kinase domain-like protein | 2.23 |
| Q9D3U0 | Putative tRNA pseudouridine synthase Pus10 | 0.40 |
| Q9D620 | Rab11 family-interacting protein 1 | 4.64 |
| Q9D6N1 | Carbonic anhydrase 13 | 2.42 |
| Q9D6Y9 | 1,4-alpha-glucan-branching enzyme | 2.13 |
| Q9D7I8 | Protein FAM83D | 1.78 |
| Q9D7X8 | Gamma-glutamylcyclotransferase | 2.13 |
| Q9D8B3 | Charged multivesicular body protein 4b | 1.63 |
| Q9D8C4 | Interferon-induced 35 kDa protein homolog | 2.19 |
| Q9D964 | Glycine amidinotransferase, mitochondrial | 0.55 |
| Q9D975 | Sulfiredoxin-1 | 8.07 |
| Q9DAA6 | Exosome complex component CSL4 | 1.63 |
| Q9DB40 | Mediator of RNA polymerase II transcription subunit 27 | 0.57 |
| Q9DBG7 | Signal recognition particle receptor subunit alpha | 1.86 |
| Q9DCD0 | 6-phosphogluconate dehydrogenase, decarboxylating | 1.77 |
| Q9DCE9 |  | 4.64 |
| Q9EP73 | Programmed cell death 1 ligand 1 | 5.74 |
| Q9EQK5 | Major vault protein | 2.42 |
| Q9ESY9 | Gamma-interferon-inducible lysosomal thiol reductase | 0.54 |
| Q9JHK5 | Pleckstrin | 1.83 |
| Q9JIA7 | Sphingosine kinase 2 | 2.04 |
| Q9JJ78 | Lymphokine-activated killer T-cell-originated protein kinase | 0.30 |
| Q9JL16 | Interferon-stimulated gene 20 kDa protein | 3.30 |
| Q9JM90 | Signal-transducing adaptor protein 1 | 2.43 |
| Q9JMH6 | Thioredoxin reductase 1, cytoplasmic | 2.53 |
| Q9QXJ2 | Signal transducer and activator of transcription 2 | 2.32 |
| Q9QY24 | Z-DNA-binding protein 1 | 1.92 |
| Q9QY81 | Nuclear pore membrane glycoprotein 210 | 0.52 |
| Q9QYB1 | Chloride intracellular channel protein 4 | 1.95 |
| Q9QZI9 | Serine incorporator 3 | 0.32 |
| Q9R0E2 | Procollagen-lysine,2-oxoglutarate 5-dioxygenase 1 | 0.44 |
| Q9R0P3 | S-formylglutathione hydrolase | 2.93 |
| Q9R1X4 | Protein timeless homolog | 0.50 |
| Q9R233 | Tapasin | 2.83 |
| Q9WTK5 | Nuclear factor NF-kappa-B p100 subunit;Nuclear factor NF-kappa-B p52 subunit | 3.10 |
| Q9WTQ5 | A-kinase anchor protein 12 | 4.75 |
| Q9WTQ8 | Mitochondrial import inner membrane translocase subunit Tim23 | 1.88 |
| Q9WTR6 | Cystine/glutamate transporter | 5.77 |
| Q9WTV6 | Ubl carboxyl-terminal hydrolase 18 | 1.79 |
| Q9WVF7 | DNA polymerase epsilon catalytic subunit A | 0.40 |
| Q9WVG6 | Histone-arginine methyltransferase CARM1 | 0.56 |
| Q9WVK4 | EH domain-containing protein 1 | 5.80 |
| Q9Z0E6 | Interferon-induced guanylate-binding protein 2 | 5.79 |
| Q9Z1M2 |  | 2.90 |
| Q9Z1Q2 | Abhydrolase domain-containing protein 16A | 1.65 |
| Q9Z265 | Serine/threonine-protein kinase Chk2 | 0.56 |
| Q9Z277 | Tyrosine-protein kinase BAZ1B | 0.53 |
| Q9Z2F2 | 2-5-oligoadenylate synthase-like protein 2 | 1.92 |
| Q9Z2X2 | 26S proteasome non-ATPase regulatory subunit 10 | 1.88 |

**Supplemental Table 2: M2a State Differentially Expressed Proteins**. Protein Expression levels for all significant (p<0.05) proteins for the M2a activation state.

| Uniprot ID | Protein name | M2a |
| --- | --- | --- |
| A2AKI5 | Integrin alpha-V;Integrin alpha-V heavy chain;Integrin alpha-V light chain | 0.57 |
| A2ALR9 | Ubiquitin carboxyl-terminal hydrolase 48 | 1.29 |
| A2AR26 |  | 1.68 |
| A2AS05 |  | 1.91 |
| A2AWF8 | Receptor-type tyrosine-protein phosphatase eta | 1.48 |
| A2CF65 | C-type lectin domain family 10 member A | 3.65 |
| A2CG76 | Histone-lysine N-methyltransferase EHMT2 | 0.67 |
| A4QPD3 | Proto-oncogene c-Rel | 1.53 |
| A6H630 | UPF0364 protein C6orf211 homolog | 4.38 |
| A6PWS5 |  | 0.62 |
| A7UAK4 | 6-phosphofructo-2-kinase/fructose-2,6-bisphosphatase 2;6-phosphofructo-2-kinase;Fructose-2,6-bisphosphatase | 0.47 |
| A9XX86 |  | 4.34 |
| B0QZX1 | Cytokine receptor common subunit gamma | 1.35 |
| B1AQR8 | Galectin-9 | 1.68 |
| B1ATI9 | Growth arrest-specific protein 7 | 0.45 |
| B1AW21 | Myotubularin | 1.38 |
| B1AZ46 | Brain-specific angiogenesis inhibitor 1-associated protein 2 | 1.43 |
| B2RUJ8 | Rho GTPase-activating protein 12 | 1.75 |
| B2RY04 | Dedicator of cytokinesis protein 5 | 0.66 |
| D3YUG2 | DCN1-like protein 1 | 3.66 |
| D3YUT6 |  | 0.57 |
| D3YVK0 | Acyl-CoA:lysophosphatidylglycerol acyltransferase 1 | 0.67 |
| D3YVU8 | ETS translocation variant 3 | 1.48 |
| D3YVV1 | Cell cycle control protein 50A | 0.61 |
| D3YVV4 | Plasma alpha-L-fucosidase | 0.62 |
| D3YWQ8 | Protein kinase C eta type | 0.61 |
| D3YX27 | Serine protease HTRA2, mitochondrial | 0.64 |
| D3YX57 | Fanconi anemia group I protein homolog | 1.32 |
| D3YYZ2 |  | 1.30 |
| D3YZ08 | Telomeric repeat-binding factor 2 | 0.60 |
| D3Z016 | Proteasome assembly chaperone 4 | 0.39 |
| D3Z0V2 | Rho guanine nucleotide exchange factor 7 | 0.40 |
| D3Z125 | Tumor protein D52 | 0.59 |
| D3Z282 | RISC-loading complex subunit TARBP2 | 0.45 |
| D3Z2E7 |  | 1.64 |
| D3Z2J4 | AKT-interacting protein | 0.64 |
| D3Z2Q2 | Syntaxin-binding protein 5 | 0.55 |
| D3Z2V4 | Scavenger receptor class B member 1 | 0.65 |
| D3Z3A6 | Protein PML | 1.38 |
| D3Z5I1 | Zinc finger CCCH-type antiviral protein 1 | 1.52 |
| D3Z6I4 | Quinone oxidoreductase-like protein 1 | 1.33 |
| D3Z6Q9 | Bridging integrator 2 | 1.39 |
| D6RCJ3 | Tripartite motif-containing protein 12A | 1.53 |
| E0CY57 |  | 0.03 |
| E0CY74 |  | 2.54 |
| E0CYH4 | WD repeat-containing protein 26 | 1.46 |
| E9PVX6 |  | 0.57 |
| E9PXE6 | Formin-like protein 2 | 2.01 |
| E9PYX5 | Probable cation-transporting ATPase 13A2 | 0.58 |
| E9Q246 | DENN domain-containing protein 1B | 1.51 |
| E9Q3N1 | High affinity cationic amino acid transporter 1 | 1.59 |
| E9Q415 | Low affinity immunoglobulin gamma Fc region receptor II | 2.96 |
| E9Q555 | E3 ubiquitin-protein ligase RNF213 | 1.96 |
| E9Q5I7 | Endoglin | 1.30 |
| E9Q616 |  | 1.33 |
| E9Q634 | Unconventional myosin-Ie | 1.39 |
| E9Q6B2 | Coiled-coil domain-containing protein 85C | 0.06 |
| E9Q6F0 | Protein FAM3C | 0.39 |
| E9Q7S1 | Zinc finger protein 106 | 0.66 |
| E9Q852 | Afadin | 1.45 |
| E9Q9A9 | 2-5-oligoadenylate synthase 2 | 1.46 |
| E9QK04 | Neogenin | 0.51 |
| E9QKL6 |  | 3.17 |
| E9QKR1 | Protein enabled homolog | 1.58 |
| E9QN39 |  | 0.47 |
| E9QQ48 | Disks large-associated protein 5 | 0.08 |
| F6S7U1 | Protein RIC1 homolog | 1.35 |
| F6YX19 | Phosphatidylinositol-glycan biosynthesis class X protein | 2.67 |
| F7CVJ5 |  | 0.67 |
| F8VPN4 |  | 0.61 |
| F8WJB9 | Ena/VASP-like protein | 1.33 |
| G3UX50 | Death domain-associated protein 6 | 1.91 |
| G3UYL4 | G-protein-signaling modulator 3 | 0.31 |
| G3X8Y8 | Toll-like receptor | 0.57 |
| H3BJ53 |  | 0.64 |
| I1E4X0 | Disks large-associated protein 4 | 1.97 |
| I6L974 | TBC1 domain family member 17 | 0.67 |
| M0QWR5 | C-type lectin domain family 7 member A | 1.49 |
| O08738 | Caspase-6;Caspase-6 subunit p18;Caspase-6 subunit p11 | 2.38 |
| O08784 | Treacle protein | 1.33 |
| O08804 |  | 2.06 |
| O08908 | Phosphatidylinositol 3-kinase regulatory subunit beta | 0.67 |
| O35309 | N-myc-interactor | 1.85 |
| O35601 | FYN-binding protein | 0.59 |
| O35955 | Proteasome subunit beta type-10 | 1.86 |
| O54782 | Epididymis-specific alpha-mannosidase | 2.11 |
| O54918 | Bcl-2-like protein 11 | 2.48 |
| O70475 | UDP-glucose 6-dehydrogenase | 0.58 |
| O70494 | Transcription factor Sp3 | 1.66 |
| O70566 | Protein diaphanous homolog 2 | 1.31 |
| O89001 | Carboxypeptidase D | 0.53 |
| O89050 | Muskelin | 1.50 |
| P01027 | Complement C3 | 4.17 |
| P01887 | Beta-2-microglobulin | 1.35 |
| P01899 | H-2 class I histocompatibility antigen, D-B alpha chain | 1.55 |
| P01900 | H-2 class I histocompatibility antigen, D-D alpha chain | 2.36 |
| P01901 | H-2 class I histocompatibility antigen, K-B alpha chain;H-2 class I histocompatibility antigen, K-K alpha chain | 1.55 |
| P04117 | Fatty acid-binding protein, adipocyte | 1.31 |
| P04184 | Thymidine kinase, cytosolic | 0.33 |
| P04441 | H-2 class II histocompatibility antigen gamma chain | 6.52 |
| P05063 | Fructose-bisphosphate aldolase C | 0.62 |
| P05132 | cAMP-dependent protein kinase catalytic subunit alpha | 0.67 |
| P06797 | Cathepsin L1;Cathepsin L1 heavy chain;Cathepsin L1 light chain | 1.30 |
| P08003 | Protein disulfide-isomerase A4 | 1.34 |
| P08226 | Apolipoprotein E | 0.45 |
| P08905 | Lysozyme C-2 | 0.19 |
| P09581 | Macrophage colony-stimulating factor 1 receptor | 0.43 |
| P10400 | Retrovirus-related Pol polyprotein;Reverse transcriptase;Endonuclease | 0.55 |
| P10810 | Monocyte differentiation antigen CD14 | 0.24 |
| P10923 | Osteopontin | 0.54 |
| P11157 | Ribonucleoside-diphosphate reductase subunit M2 | 0.64 |
| P11688 | Integrin alpha-5;Integrin alpha-5 heavy chain;Integrin alpha-5 light chain | 1.63 |
| P11928 | 2-5-oligoadenylate synthase 1A | 1.44 |
| P13020 | Gelsolin | 0.67 |
| P13597 | Intercellular adhesion molecule 1 | 1.97 |
| P18242 | Cathepsin D | 0.58 |
| P19182 | Interferon-related developmental regulator 1 | 2.20 |
| P21279 | Guanine nucleotide-binding protein G(q) subunit alpha | 0.61 |
| P21956 | Lactadherin | 0.39 |
| P22437 | Prostaglandin G/H synthase 1 | 1.64 |
| P22682 | E3 ubiquitin-protein ligase CBL | 1.44 |
| P25799 | Nuclear factor NF-kappa-B p105 subunit;Nuclear factor NF-kappa-B p50 subunit | 1.42 |
| P25911 | Tyrosine-protein kinase Lyn | 0.50 |
| P26645 | Myristoylated alanine-rich C-kinase substrate | 1.36 |
| P27512 | Tumor necrosis factor receptor superfamily member 5 | 3.45 |
| P27808 | Alpha-1,3-mannosyl-glycoprotein 2-beta-N-acetylglucosaminyltransferase | 1.38 |
| P28033 | CCAAT/enhancer-binding protein beta | 1.28 |
| P28650 | Adenylosuccinate synthetase isozyme 1 | 0.52 |
| P28667 | MARCKS-related protein | 1.39 |
| P28867 | Protein kinase C delta type;Protein kinase C delta type regulatory subunit;Protein kinase C delta type catalytic subunit | 1.35 |
| P29416 | Beta-hexosaminidase subunit alpha | 0.67 |
| P35761 | Dual specificity protein kinase TTK | 0.66 |
| P35951 | Low-density lipoprotein receptor | 1.42 |
| P46718 | Programmed cell death protein 2 | 1.30 |
| P50096 | Inosine-5-monophosphate dehydrogenase 1;Inosine-5-monophosphate dehydrogenase | 1.48 |
| P50427 | Steryl-sulfatase | 0.57 |
| P51175 | Protoporphyrinogen oxidase | 0.58 |
| P52431 | DNA polymerase delta catalytic subunit | 1.68 |
| P52503 | NADH dehydrogenase [ubiquinone] iron-sulfur protein 6, mitochondrial | 0.51 |
| P54116 | Erythrocyte band 7 integral membrane protein | 0.60 |
| P54729 | NEDD8 ultimate buster 1 | 1.62 |
| P54987 | Immune-responsive gene 1 protein | 6.28 |
| P55097 | Cathepsin K | 0.55 |
| P56380 | Bis(5-nucleosyl)-tetraphosphatase [asymmetrical] | 1.37 |
| P57787 | Monocarboxylate transporter 4 | 0.67 |
| P58044 | Isopentenyl-diphosphate Delta-isomerase 1 | 1.55 |
| P58058 | NAD kinase | 1.32 |
| P58059 | 28S ribosomal protein S21, mitochondrial | 1.93 |
| P58681 | Toll-like receptor 7 | 0.65 |
| P59325 | Eukaryotic translation initiation factor 5 | 1.47 |
| P59326 | YTH domain family protein 1 | 1.40 |
| P59764 | Dedicator of cytokinesis protein 4 | 1.38 |
| P60330 | Separin | 0.45 |
| P60840 | Alpha-endosulfine | 6.80 |
| P61028 | Ras-related protein Rab-8B | 2.13 |
| P61222 | ATP-binding cassette sub-family E member 1 | 1.49 |
| P61600 | N-alpha-acetyltransferase 20 | 0.65 |
| P62746 | Rho-related GTP-binding protein RhoB | 1.29 |
| P70387 | Hereditary hemochromatosis protein homolog | 1.99 |
| P83741 | Serine/threonine-protein kinase WNK1 | 1.75 |
| P97329 | Kinesin-like protein KIF20A | 0.56 |
| P97333 | Neuropilin-1 | 0.52 |
| P97429 | Annexin A4 | 1.60 |
| P97465 | Docking protein 1 | 0.48 |
| P97477 | Aurora kinase A | 0.66 |
| P97865 | Peroxisomal targeting signal 2 receptor | 1.35 |
| P98078 | Disabled homolog 2 | 2.06 |
| Q00519 | Xanthine dehydrogenase/oxidase;Xanthine dehydrogenase;Xanthine oxidase | 1.77 |
| Q03963 | Interferon-induced, double-stranded RNA-activated protein kinase | 1.42 |
| Q04447 | Creatine kinase B-type | 0.48 |
| Q05186 | Reticulocalbin-1 | 1.44 |
| Q07139 | Protein ECT2 | 0.57 |
| Q2EMV9 | Poly [ADP-ribose] polymerase 14 | 2.51 |
| Q3TAS6 | ER membrane protein complex subunit 10 | 1.45 |
| Q3TB82 | Pleckstrin homology domain-containing family F member 1 | 0.19 |
| Q3TBA3 | Antigen peptide transporter 1 | 2.42 |
| Q3TBU7 | Arf-GAP domain and FG repeat-containing protein 2 | 0.63 |
| Q3TBV5 | Interleukin-1 receptor antagonist protein | 1.38 |
| Q3TFD2 | Lysophosphatidylcholine acyltransferase 1 | 1.48 |
| Q3TGW2 | Endonuclease/exonuclease/phosphatase family domain-containing protein 1 | 0.67 |
| Q3TH73 | Protein tweety homolog 2 | 0.63 |
| Q3TJ22 |  | 1.56 |
| Q3TJM4 | Centromere protein T | 0.59 |
| Q3TLZ6 | Glutamyl-tRNA(Gln) amidotransferase subunit B, mitochondrial | 3.95 |
| Q3TQ29 | Pumilio homolog 2 | 1.57 |
| Q3TVP5 | Protein FAM105A | 0.63 |
| Q3TZ86 | C5a anaphylatoxin chemotactic receptor | 0.43 |
| Q3U2C5 | E3 ubiquitin-protein ligase RNF149 | 0.46 |
| Q3U5Q7 | UMP-CMP kinase 2, mitochondrial | 5.62 |
| Q3U7K7 | E3 ubiquitin-protein ligase TRIM21 | 1.46 |
| Q3UDK1 | TRAF-type zinc finger domain-containing protein 1 | 2.81 |
| Q3UIR3 | E3 ubiquitin-protein ligase DTX3L | 2.10 |
| Q3UM18 | Large subunit GTPase 1 homolog | 1.33 |
| Q3UMB5 | Smith-Magenis syndrome chromosomal region candidate gene 8 protein homolog | 1.35 |
| Q3UMC0 | Spermatogenesis-associated protein 5 | 1.36 |
| Q3UNI1 | NAD-dependent protein deacetylase sirtuin-1;SirtT1 75 kDa fragment | 1.42 |
| Q3UQ44 | Ras GTPase-activating-like protein IQGAP2 | 0.63 |
| Q3UUQ7 | GPI inositol-deacylase | 0.51 |
| Q3V1V3 | ESF1 homolog | 1.28 |
| Q3V209 | Transmembrane and ubiquitin-like domain-containing protein 2 | 1.30 |
| Q3V300 | Kinesin-like protein KIF22 | 0.58 |
| Q542I8 | Integrin beta;Integrin beta-2 | 0.66 |
| Q543K9 | Purine nucleoside phosphorylase | 1.50 |
| Q58A65 | C-Jun-amino-terminal kinase-interacting protein 4 | 1.28 |
| Q5NBU8 | XIAP-associated factor 1 | 1.62 |
| Q5NCB5 |  | 2.20 |
| Q5NCQ5 | Diphthamide biosynthesis protein 1 | 1.62 |
| Q5PRF0 | HEAT repeat-containing protein 5A | 1.34 |
| Q5SU47 | Cytoplasmic polyadenylation element-binding protein 4 | 1.55 |
| Q5SYD0 | Unconventional myosin-Id | 0.41 |
| Q60591 | Nuclear factor of activated T-cells, cytoplasmic 2 | 0.64 |
| Q60710 | SAM domain and HD domain-containing protein 1 | 1.58 |
| Q60738 | Zinc transporter 1 | 1.67 |
| Q60739 | BAG family molecular chaperone regulator 1 | 0.60 |
| Q60766 | Immunity-related GTPase family M protein 1 | 3.33 |
| Q60770 | Syntaxin-binding protein 3 | 1.36 |
| Q61176 | Arginase-1 | 4.73 |
| Q61249 | Immunoglobulin-binding protein 1 | 1.56 |
| Q61462 | Cytochrome b-245 light chain | 4.06 |
| Q61549 | EGF-like module-containing mucin-like hormone receptor-like 1 | 1.61 |
| Q61635 |  | 2.51 |
| Q61735 | Leukocyte surface antigen CD47 | 1.95 |
| Q61739 | Integrin alpha-6;Integrin alpha-6 heavy chain;Integrin alpha-6 light chain | 0.58 |
| Q61830 | Macrophage mannose receptor 1 | 2.81 |
| Q62293 |  | 2.18 |
| Q62388 | Serine-protein kinase ATM | 0.67 |
| Q62433 | Protein NDRG1 | 0.50 |
| Q63836 | Selenium-binding protein 2;Selenium-binding protein 1 | 0.54 |
| Q64253 | Lymphocyte antigen 6E | 1.29 |
| Q64261 | Cyclin-dependent kinase 6 | 1.28 |
| Q64282 | Interferon-induced protein with tetratricopeptide repeats 1 | 4.29 |
| Q64337 | Sequestosome-1 | 2.35 |
| Q64339 | Ubiquitin-like protein ISG15 | 6.55 |
| Q64343 | ATP-binding cassette sub-family G member 1 | 1.30 |
| Q64345 | Interferon-induced protein with tetratricopeptide repeats 3 | 5.97 |
| Q69Z37 | Sterile alpha motif domain-containing protein 9-like | 2.02 |
| Q6GTM0 | Interferon-induced protein with tetratricopeptide repeats 2 | 4.93 |
| Q6KAR6 | Exocyst complex component 3 | 0.49 |
| Q6NV52 | Transforming acidic coiled-coil-containing protein 3 | 0.65 |
| Q6NVG1 | Lysophospholipid acyltransferase LPCAT4 | 0.52 |
| Q6NXL1 |  | 1.33 |
| Q6P1F6 | Serine/threonine-protein phosphatase 2A 55 kDa regulatory subunit B alpha isoform | 1.29 |
| Q6P549 | Phosphatidylinositol 3,4,5-trisphosphate 5-phosphatase 2 | 0.66 |
| Q6P8H8 | Probable dolichyl pyrophosphate Glc1Man9GlcNAc2 alpha-1,3-glucosyltransferase | 0.28 |
| Q6P8X1 | Sorting nexin-6 | 1.30 |
| Q6P9P6 | Kinesin-like protein KIF11 | 0.67 |
| Q6P9Q4 | FH1/FH2 domain-containing protein 1 | 0.59 |
| Q6PAV2 | Probable E3 ubiquitin-protein ligase HERC4 | 1.29 |
| Q6PD03 | Serine/threonine-protein phosphatase 2A 56 kDa regulatory subunit alpha isoform | 0.64 |
| Q6Q899 | Probable ATP-dependent RNA helicase DDX58 | 1.28 |
| Q6R5N8 | Toll-like receptor 13 | 1.32 |
| Q6RT24 | Centromere-associated protein E | 0.42 |
| Q6WKZ8 | E3 ubiquitin-protein ligase UBR2 | 1.34 |
| Q6ZQK5 | Arf-GAP with coiled-coil, ANK repeat and PH domain-containing protein 2 | 0.65 |
| Q76LS9 | Protein FAM63A | 0.23 |
| Q792F9 | Integrin alpha-4 | 0.67 |
| Q7M6Y3 | Phosphatidylinositol-binding clathrin assembly protein | 1.53 |
| Q7TME2 | Sperm-associated antigen 5 | 1.35 |
| Q7TMG8 | Protein NipSnap homolog 2 | 0.67 |
| Q7TMI3 | E3 ubiquitin-protein ligase UHRF2 | 0.62 |
| Q7TMY4 | THO complex subunit 7 homolog | 0.66 |
| Q7TN29 | Stromal membrane-associated protein 2 | 1.99 |
| Q7TND5 | Ribosome production factor 1 | 1.30 |
| Q7TSF4 | Leucine-rich repeat-containing protein FAM211A | 0.50 |
| Q7TSH2 | Phosphorylase b kinase regulatory subunit beta | 2.70 |
| Q80SU7 | Interferon-induced very large GTPase 1 | 2.38 |
| Q80UK0 | SEC14 domain and spectrin repeat-containing protein 1 | 1.75 |
| Q80UP3 | Diacylglycerol kinase zeta | 0.61 |
| Q80UU1 | Ankyrin repeat and zinc finger domain-containing protein 1 | 4.12 |
| Q80UZ2 | Protein SDA1 homolog | 1.35 |
| Q80VP0 | Tectonin beta-propeller repeat-containing protein 1 | 0.46 |
| Q80WW9 | DDRGK domain-containing protein 1 | 1.68 |
| Q80X41 | Serine/threonine-protein kinase VRK1 | 1.36 |
| Q80Y19 | Rho GTPase-activating protein 11A | 0.63 |
| Q80YW0 | Cytohesin-4 | 0.57 |
| Q811J3 | Iron-responsive element-binding protein 2 | 0.64 |
| Q8BG07 | Phospholipase D4 | 0.49 |
| Q8BG73 | SH3 domain-binding glutamic acid-rich-like protein 2 | 0.60 |
| Q8BGR9 | Ubiquitin-like domain-containing CTD phosphatase 1 | 1.32 |
| Q8BGS0 | Protein MAK16 homolog | 1.94 |
| Q8BGU5 | Cyclin-Y | 0.62 |
| Q8BH86 | UPF0317 protein C14orf159 homolog, mitochondrial | 0.64 |
| Q8BHS6 | Armadillo repeat-containing X-linked protein 3 | 1.32 |
| Q8BHX3 | Borealin | 1.42 |
| Q8BJ56 | Patatin-like phospholipase domain-containing protein 2 | 1.29 |
| Q8BK35 |  | 1.47 |
| Q8BLD4 |  | 0.42 |
| Q8BLH7 | HIRA-interacting protein 3 | 1.30 |
| Q8BNU0 | Armadillo repeat-containing protein 6 | 1.29 |
| Q8BU33 | Acetolactate synthase-like protein | 0.63 |
| Q8BUB4 | WD repeat and FYVE domain-containing protein 2 | 2.37 |
| Q8BV49 | Pyrin and HIN domain-containing protein 1 | 4.84 |
| Q8BVG4 | Dipeptidyl peptidase 9 | 0.23 |
| Q8BVK9 | Sp110 nuclear body protein | 2.82 |
| Q8BVN4 | mTERF domain-containing protein 2 | 0.58 |
| Q8BWZ3 | N-alpha-acetyltransferase 25, NatB auxiliary subunit | 1.30 |
| Q8BX09 | Retinoblastoma-binding protein 5 | 0.67 |
| Q8BYW1 | Rho GTPase-activating protein 25 | 0.61 |
| Q8BZ20 | Poly [ADP-ribose] polymerase 12 | 1.64 |
| Q8BZW8 | NHL repeat-containing protein 2 | 1.31 |
| Q8C0E2 | Vacuolar protein sorting-associated protein 26B | 0.65 |
| Q8C0Y0 | Serine/threonine-protein phosphatase 4 regulatory subunit 4 | 1.37 |
| Q8C2E4 | Pentatricopeptide repeat-containing protein 1, mitochondrial | 1.32 |
| Q8C3V4 | Signal transducer and activator of transcription 1 | 2.44 |
| Q8C6H0 | Galectin-8 | 2.45 |
| Q8C7K6 | Prenylcysteine oxidase-like | 1.40 |
| Q8C9T1 |  | 1.55 |
| Q8CAS9 | Poly [ADP-ribose] polymerase 9 | 2.35 |
| Q8CB77 | Transcription elongation factor B polypeptide 3 | 1.55 |
| Q8CBB9 | Radical S-adenosyl methionine domain-containing protein 2 | 7.08 |
| Q8CE80 | Calpastatin | 0.60 |
| Q8CEE7 | Retinol dehydrogenase 13 | 294.79 |
| Q8CFB4 | Guanylate-binding protein 5 | 2.97 |
| Q8CFB8 |  | 1.37 |
| Q8CG29 | Unconventional myosin-If | 0.66 |
| Q8CG50 | Ras-related protein Rab-43 | 1.39 |
| Q8CGC4 | Protein LSM14 homolog B | 0.61 |
| Q8CGC7 | Bifunctional glutamate/proline--tRNA ligase;Glutamate--tRNA ligase;Proline--tRNA ligase | 1.31 |
| Q8CHI8 | E1A-binding protein p400 | 0.48 |
| Q8CIH9 | Amidophosphoribosyltransferase | 2.06 |
| Q8CIW5 | Twinkle protein, mitochondrial | 1.87 |
| Q8CJ53 | Cdc42-interacting protein 4 | 1.32 |
| Q8K072 | Receptor expression-enhancing protein 4 | 0.60 |
| Q8K0S9 | snRNA-activating protein complex subunit 1 | 23.02 |
| Q8K0V4 | CCR4-NOT transcription complex subunit 3 | 1.39 |
| Q8K268 | ATP-binding cassette sub-family F member 3 | 1.33 |
| Q8K4Q8 | Collectin-12 | 0.61 |
| Q8QZV7 | Protein asunder homolog | 0.27 |
| Q8R080 | G2 and S phase-expressed protein 1 | 0.53 |
| Q8R151 | NFX1-type zinc finger-containing protein 1 | 1.31 |
| Q8R164 | Valacyclovir hydrolase | 0.56 |
| Q8R1N0 | Zinc finger protein 830 | 1.29 |
| Q8R2E9 | ERO1-like protein beta | 0.59 |
| Q8R2Q8 | Bone marrow stromal antigen 2 | 1.94 |
| Q8R3F9 | Speckle targeted PIP5K1A-regulated poly(A) polymerase | 1.31 |
| Q8R4G6 | Alpha-1,6-mannosylglycoprotein 6-beta-N-acetylglucosaminyltransferase A | 0.65 |
| Q8R5F7 | Interferon-induced helicase C domain-containing protein 1 | 3.45 |
| Q8R5M8 | Cell adhesion molecule 1 | 0.65 |
| Q8VBT6 | Apolipoprotein B receptor | 0.62 |
| Q8VCF0 | Mitochondrial antiviral-signaling protein | 0.66 |
| Q8VCR4 | tRNA methyltransferase 112 homolog | 1.64 |
| Q8VCW4 | Protein unc-93 homolog B1 | 0.61 |
| Q8VDQ9 | Protein KRI1 homolog | 1.29 |
| Q8VDS8 | Syntaxin-18 | 3.54 |
| Q8VDU3 |  | 4.91 |
| Q8VE08 | F-box only protein 33 | 1.33 |
| Q8VE97 | Serine/arginine-rich splicing factor 4 | 1.36 |
| Q8VEG6 | CCR4-NOT transcription complex subunit 6-like | 1.65 |
| Q8VI84 | Nucleolar complex protein 3 homolog | 1.30 |
| Q8VI93 | 2-5-oligoadenylate synthase 3 | 1.67 |
| Q8VI94 | 2-5-oligoadenylate synthase-like protein 1 | 2.69 |
| Q8VIM0 | Hepatitis A virus cellular receptor 2 homolog | 0.53 |
| Q91VE0 | Long-chain fatty acid transport protein 4 | 0.54 |
| Q91VY9 | Zinc finger protein 622 | 1.97 |
| Q91WG4 | Elongator complex protein 2 | 1.54 |
| Q91XB0 | Three prime repair exonuclease 1 | 2.75 |
| Q91XV3 | Brain acid soluble protein 1 | 0.67 |
| Q91YJ3 | Thymocyte nuclear protein 1 | 1.31 |
| Q91YX0 | Protein THEMIS2 | 1.47 |
| Q91Z40 |  | 2.99 |
| Q91ZX7 | Prolow-density lipoprotein receptor-related protein 1 | 0.46 |
| Q921H8 | 3-ketoacyl-CoA thiolase A, peroxisomal | 0.65 |
| Q922H1 | Protein arginine N-methyltransferase 3 | 1.33 |
| Q922J3 | CAP-Gly domain-containing linker protein 1 | 0.62 |
| Q922S8 | Kinesin-like protein KIF2C | 0.62 |
| Q923B1 | Lariat debranching enzyme | 0.59 |
| Q925E7 | Serine/threonine-protein phosphatase 2A 55 kDa regulatory subunit B delta isoform | 1.36 |
| Q99J87 | Probable ATP-dependent RNA helicase DHX58 | 2.32 |
| Q99J99 | 3-mercaptopyruvate sulfurtransferase;Sulfurtransferase | 0.64 |
| Q99KC8 | von Willebrand factor A domain-containing protein 5A | 2.00 |
| Q99L04 | Dehydrogenase/reductase SDR family member 1 | 0.55 |
| Q99L48 | 60S ribosomal export protein NMD3 | 1.53 |
| Q99LC8 | Translation initiation factor eIF-2B subunit alpha | 1.48 |
| Q99LE1 | RILP-like protein 2 | 0.62 |
| Q99MB1 | Toll-like receptor 3 | 1.31 |
| Q99MU3 | Double-stranded RNA-specific adenosine deaminase | 1.53 |
| Q99N69 | Leupaxin | 2.64 |
| Q99P65 | Equilibrative nucleoside transporter 3 | 0.63 |
| Q99P91 | Transmembrane glycoprotein NMB | 0.57 |
| Q9CPR8 | Melanoma-associated antigen G1 | 0.49 |
| Q9CPU4 | Microsomal glutathione S-transferase 3 | 0.60 |
| Q9CPW3 | 39S ribosomal protein L54, mitochondrial | 0.67 |
| Q9CPW7 | Zinc finger matrin-type protein 2 | 0.05 |
| Q9CQ28 | Diphthine--ammonia ligase | 1.48 |
| Q9CQ86 | Migration and invasion enhancer 1 | 1.64 |
| Q9CQA5 | Mediator of RNA polymerase II transcription subunit 4 | 0.61 |
| Q9CQE5 | Regulator of G-protein signaling 10 | 0.53 |
| Q9CQT0 | Probable tRNA(His) guanylyltransferase | 1.32 |
| Q9CQX4 | PCNA-associated factor | 0.64 |
| Q9CR02 | Translation machinery-associated protein 16 | 1.31 |
| Q9CR29 | Coiled-coil domain-containing protein 43 | 1.61 |
| Q9CR95 | Adaptin ear-binding coat-associated protein 1 | 1.95 |
| Q9CRY7 | Glycerophosphodiester phosphodiesterase domain-containing protein 1 | 0.52 |
| Q9CWK8 | Sorting nexin-2 | 1.31 |
| Q9CWZ3 | RNA-binding protein 8A | 1.31 |
| Q9CXY9 | GPI-anchor transamidase | 1.73 |
| Q9CYZ6 | Uncharacterized protein C19orf60 homolog | 2.76 |
| Q9CZN8 | Glutamyl-tRNA(Gln) amidotransferase subunit A, mitochondrial | 2.62 |
| Q9CZS1 | Aldehyde dehydrogenase X, mitochondrial | 1.60 |
| Q9CZX0 | Elongator complex protein 3 | 1.43 |
| Q9D061 | Acyl-CoA-binding domain-containing protein 6 | 1.35 |
| Q9D0C1 | E3 ubiquitin-protein ligase RNF115 | 1.41 |
| Q9D154 | Leukocyte elastase inhibitor A | 1.31 |
| Q9D168 | Integrator complex subunit 12 | 0.55 |
| Q9D1I5 | Methylmalonyl-CoA epimerase, mitochondrial | 0.61 |
| Q9D4E6 |  | 1.45 |
| Q9D6N1 | Carbonic anhydrase 13 | 1.51 |
| Q9D7S7 | 60S ribosomal protein L22-like 1 | 1.53 |
| Q9D7Z3 | Nucleolar protein 7 | 1.29 |
| Q9D8C4 | Interferon-induced 35 kDa protein homolog | 1.70 |
| Q9D8U0 |  | 1.37 |
| Q9D964 | Glycine amidinotransferase, mitochondrial | 1.47 |
| Q9D975 | Sulfiredoxin-1 | 2.32 |
| Q9DAK9 | 14 kDa phosphohistidine phosphatase | 0.67 |
| Q9DBA6 | Peroxisomal leader peptide-processing protease;Peroxisomal leader peptide-processing protease, 10 kDa form;Peroxisomal leader peptide-processing protease, 49 kDa form | 9.05 |
| Q9DBA9 | General transcription factor IIH subunit 1 | 1.37 |
| Q9DBC3 | Cap-specific mRNA (nucleoside-2-O-)-methyltransferase 1 | 1.31 |
| Q9DBW0 | Cytochrome P450 4V2 | 1.67 |
| Q9DC50 | Peroxisomal carnitine O-octanoyltransferase | 3.45 |
| Q9DC70 | NADH dehydrogenase [ubiquinone] iron-sulfur protein 7, mitochondrial | 1.48 |
| Q9DCE9 |  | 2.98 |
| Q9EP82 | tRNA (guanine-N(7)-)-methyltransferase subunit WDR4 | 1.39 |
| Q9EPK2 | Protein XRP2 | 0.66 |
| Q9EQ06 | Estradiol 17-beta-dehydrogenase 11 | 0.66 |
| Q9EQJ0 | Two pore calcium channel protein 1 | 0.51 |
| Q9ER38 | Torsin-3A | 1.91 |
| Q9ER81 | 15 kDa interferon-responsive protein | 1.56 |
| Q9ERH4 | Nucleolar and spindle-associated protein 1 | 3.13 |
| Q9ERR7 | 15 kDa selenoprotein | 0.60 |
| Q9ESV0 | ATP-dependent RNA helicase DDX24 | 1.35 |
| Q9ET26 | RING finger protein 114 | 1.45 |
| Q9JHF5 |  | 0.66 |
| Q9JHK5 | Pleckstrin | 1.48 |
| Q9JI99 | Sphingosine-1-phosphate phosphatase 1 | 0.58 |
| Q9JIP4 | Pannexin-1 | 0.58 |
| Q9JL16 | Interferon-stimulated gene 20 kDa protein | 8.28 |
| Q9JL26 | Formin-like protein 1 | 0.61 |
| Q9JLQ2 | ARF GTPase-activating protein GIT2 | 0.34 |
| Q9JLZ6 | Hypermethylated in cancer 2 protein | 0.61 |
| Q9QUH0 | Glutaredoxin-1 | 1.46 |
| Q9QWF0 | Chromatin assembly factor 1 subunit A | 0.66 |
| Q9QXJ2 | Signal transducer and activator of transcription 2 | 2.75 |
| Q9QXW0 | F-box/LRR-repeat protein 6 | 1.96 |
| Q9QY93 | dCTP pyrophosphatase 1 | 0.58 |
| Q9QYB1 | Chloride intracellular channel protein 4 | 1.48 |
| Q9QYC7 | Vitamin K-dependent gamma-carboxylase | 0.64 |
| Q9QYH6 | Melanoma-associated antigen D1 | 0.64 |
| Q9QZ85 | Interferon-inducible GTPase 1 | 6.68 |
| Q9QZU9 | Ubiquitin/ISG15-conjugating enzyme E2 L6 | 1.39 |
| Q9R059 | Four and a half LIM domains protein 3 | 1.31 |
| Q9R0H0 | Peroxisomal acyl-coenzyme A oxidase 1 | 0.62 |
| Q9R0Q4 | Mortality factor 4-like protein 2 | 1.46 |
| Q9R233 | Tapasin | 1.81 |
| Q9WTK5 | Nuclear factor NF-kappa-B p100 subunit;Nuclear factor NF-kappa-B p52 subunit | 1.63 |
| Q9WTR1 | Transient receptor potential cation channel subfamily V member 2 | 1.29 |
| Q9WTV6 | Ubl carboxyl-terminal hydrolase 18 | 3.78 |
| Q9WUB0 | RanBP-type and C3HC4-type zinc finger-containing protein 1 | 0.56 |
| Q9WUZ9 | Ectonucleoside triphosphate diphosphohydrolase 5 | 0.58 |
| Q9WVF7 | DNA polymerase epsilon catalytic subunit A | 1.63 |
| Q9Z0E6 | Interferon-induced guanylate-binding protein 2 | 2.82 |
| Q9Z0H1 | WD repeat-containing protein 46 | 1.32 |
| Q9Z0M5 | Lysosomal acid lipase/cholesteryl ester hydrolase | 0.55 |
| Q9Z0Z3 | S-phase kinase-associated protein 2 | 0.63 |
| Q9Z1N2 | Origin recognition complex subunit 1 | 1.30 |
| Q9Z1S0 | Mitotic checkpoint serine/threonine-protein kinase BUB1 beta | 0.52 |
| Q9Z2E1 | Methyl-CpG-binding domain protein 2 | 0.63 |
| Q9Z2F2 | 2-5-oligoadenylate synthase-like protein 2 | 3.11 |

**Supplemental Table 3: M2b State Differentially Expressed Proteins**. Protein Expression levels for all significant (p<0.05) proteins for the M2b activation state.

| Uniprot ID | Protein names | M2b |
| --- | --- | --- |
| A2A4Z1 | Ubiquitin-conjugating enzyme E2 C | 0.27 |
| A2ALA4 | Mediator of RNA polymerase II transcription subunit 22 | 3.49 |
| A2APB8 | Targeting protein for Xklp2 | 0.55 |
| A2AR02 | Peptidyl-prolyl cis-trans isomerase G | 2.53 |
| A2AR26 |  | 1.75 |
| A2AS05 |  | 2.61 |
| A2AS45 | Plakophilin-4 | 0.47 |
| A2AWF8 | Receptor-type tyrosine-protein phosphatase eta | 2.72 |
| A2CF65 | C-type lectin domain family 10 member A | 0.23 |
| A2CG76 | Histone-lysine N-methyltransferase EHMT2 | 0.54 |
| A3KGB4 | TBC1 domain family member 8B | 1.95 |
| A6H5X4 | PHD finger protein 11 | 3.79 |
| A9C471 | Baculoviral IAP repeat-containing protein 1b | 1.95 |
| B0QZT2 | Dehydrogenase/reductase SDR family member 13 | 0.49 |
| B1AQR8 | Galectin-9 | 3.06 |
| B1ASZ3 | Glycerol kinase | 1.65 |
| B1ATI9 | Growth arrest-specific protein 7 | 2.14 |
| B1AU75 |  | 0.45 |
| B1AVY7 | Kinesin-like protein KIF16B | 1.54 |
| B1AW21 | Myotubularin | 1.53 |
| B7FAU9 | Filamin-A | 1.90 |
| B7ZCU2 | Abl interactor 1 | 2.16 |
| D3YU00 | StAR-related lipid transfer protein 5 | 1.87 |
| D3YU60 | Microsomal glutathione S-transferase 1 | 2.57 |
| D3YUG2 | DCN1-like protein 1 | 4.04 |
| D3YWJ5 | Tumor necrosis factor alpha-induced protein 8 | 1.85 |
| D3YWQ8 | Protein kinase C eta type | 0.48 |
| D3YWS2 | Echinoderm microtubule-associated protein-like 2 | 1.69 |
| D3YZ08 | Telomeric repeat-binding factor 2 | 0.53 |
| D3Z0M4 | Peroxisomal membrane protein 11B | 9.67 |
| D3Z2K2 | 28S ribosomal protein S14, mitochondrial | 2.35 |
| D3Z2V4 | Scavenger receptor class B member 1 | 0.49 |
| D3Z585 | Guanine nucleotide exchange factor for Rab-3A | 0.51 |
| D6RFN5 | Ninjurin-1 | 3.04 |
| E0CXE7 | CST complex subunit CTC1 | 0.49 |
| E0CY74 |  | 4.97 |
| E0CZ78 | Serine/threonine-protein phosphatase;Serine/threonine-protein phosphatase 2B catalytic subunit beta isoform | 0.17 |
| E9PU93 | Set1/Ash2 histone methyltransferase complex subunit ASH2 | 0.50 |
| E9PUE7 | Active breakpoint cluster region-related protein | 1.58 |
| E9PV93 | T-lymphocyte surface antigen Ly-9 | 0.28 |
| E9PVX6 |  | 0.29 |
| E9Q3T3 |  | 0.33 |
| E9Q3Y4 | Lipopolysaccharide-responsive and beige-like anchor protein | 1.62 |
| E9Q4G8 | CD166 antigen | 2.01 |
| E9Q4K7 |  | 1.94 |
| E9Q555 | E3 ubiquitin-protein ligase RNF213 | 2.50 |
| E9Q5G3 |  | 0.37 |
| E9Q6B2 | Coiled-coil domain-containing protein 85C | 0.42 |
| E9Q6H7 |  | 0.52 |
| E9Q6J5 | Biorientation of chromosomes in cell division protein 1 | 0.49 |
| E9Q6K1 | TFIIH basal transcription factor complex helicase XPD subunit | 0.52 |
| E9Q6R7 |  | 0.50 |
| E9Q774 |  | 0.55 |
| E9Q8I7 |  | 1.55 |
| E9QKL6 |  | 3.32 |
| E9QM90 | LisH domain and HEAT repeat-containing protein KIAA1468 | 25.75 |
| E9QN37 | Macrophage-expressed gene 1 protein | 2.30 |
| E9QN99 |  | 5.08 |
| F6WKY8 | Phospholipase D1 | 1.56 |
| F6YTC4 | Zinc transporter ZIP10 | 0.48 |
| F7CVJ5 |  | 3.39 |
| F7DBB3 |  | 2.90 |
| F8VPN4 |  | 1.66 |
| G3UWX1 | Replication factor C subunit 1 | 0.54 |
| G3UYD0 | General transcription factor II-I | 0.56 |
| G3X8Y8 | Toll-like receptor | 1.71 |
| G3X9V0 | Proteasome activator complex subunit 2 | 1.81 |
| G5E850 | Cytochrome b5 | 2.32 |
| G5E8J8 | Protein Wiz | 1.65 |
| G5E8R3 | Pyruvate carboxylase | 1.58 |
| H3BJ51 | All-trans-retinol 13,14-reductase | 0.55 |
| I7HIK9 | Cellular tumor antigen p53 | 0.44 |
| J3QMN4 | Thioredoxin reductase 2, mitochondrial | 2.13 |
| J3QPY0 | Protein C19orf12 homolog | 1.72 |
| O08739 | AMP deaminase 3 | 2.46 |
| O08804 |  | 1.59 |
| O08901 | Mitotic checkpoint serine/threonine-protein kinase BUB1 | 0.52 |
| O08997 | Copper transport protein ATOX1 | 1.70 |
| O09172 | Glutamate--cysteine ligase regulatory subunit | 4.93 |
| O35309 | N-myc-interactor | 1.67 |
| O35316 | Sodium- and chloride-dependent taurine transporter | 0.46 |
| O35379 | Multidrug resistance-associated protein 1 | 1.89 |
| O35601 | FYN-binding protein | 2.15 |
| O35621 | Phosphomannomutase 1 | 0.11 |
| O35704 | Serine palmitoyltransferase 1 | 2.70 |
| O35887 | Calumenin | 2.04 |
| O35955 | Proteasome subunit beta type-10 | 0.54 |
| O54782 | Epididymis-specific alpha-mannosidase | 3.48 |
| O55022 | Membrane-associated progesterone receptor component 1 | 1.74 |
| O55091 | Protein IMPACT | 2.64 |
| O70126 | Aurora kinase B | 0.51 |
| O70422 | General transcription factor IIH subunit 4 | 3.82 |
| O70566 | Protein diaphanous homolog 2 | 1.55 |
| O88907 | E3 SUMO-protein ligase PIAS1 | 0.53 |
| O89001 | Carboxypeptidase D | 1.93 |
| O89110 | Caspase-8;Caspase-8 subunit p18;Caspase-8 subunit p10 | 1.56 |
| O89112 | LanC-like protein 1 | 0.19 |
| P01899 | H-2 class I histocompatibility antigen, D-B alpha chain | 1.59 |
| P01901 | H-2 class I histocompatibility antigen, K-B alpha chain | 1.59 |
| P04441 | H-2 class II histocompatibility antigen gamma chain | 1.67 |
| P06801 | NADP-dependent malic enzyme | 2.09 |
| P07356 | Annexin A2 | 2.69 |
| P07607 | Thymidylate synthase | 0.51 |
| P07742 | Ribonucleoside-diphosphate reductase large subunit | 0.32 |
| P08103 | Tyrosine-protein kinase HCK | 2.35 |
| P08226 | Apolipoprotein E | 0.38 |
| P09242 | Alkaline phosphatase, tissue-nonspecific isozyme | 1.92 |
| P09528 | Ferritin heavy chain | 6.43 |
| P09671 | Superoxide dismutase [Mn], mitochondrial | 4.59 |
| P10107 | Annexin A1 | 1.55 |
| P10605 | Cathepsin B;Cathepsin B light chain;Cathepsin B heavy chain | 2.40 |
| P10649 | Glutathione S-transferase Mu 1 | 1.61 |
| P10810 | Monocyte differentiation antigen CD14 | 3.93 |
| P11370 | Retrovirus-related Env polyprotein from Fv-4 locus | 0.53 |
| P11440 | Cyclin-dependent kinase 1 | 0.42 |
| P11688 | Integrin alpha-5;Integrin alpha-5 heavy chain;Integrin alpha-5 light chain | 2.96 |
| P11928 | 2-5-oligoadenylate synthase 1A | 2.10 |
| P13597 | Intercellular adhesion molecule 1 | 2.55 |
| P13864 | DNA (cytosine-5)-methyltransferase 1 | 0.54 |
| P14106 | Complement C1q subcomponent subunit B | 0.50 |
| P14901 | Heme oxygenase 1 | 6.41 |
| P15864 | Histone H1.2 | 0.46 |
| P17710 | Hexokinase-1 | 1.54 |
| P18242 | Cathepsin D | 1.57 |
| P19182 | Interferon-related developmental regulator 1 | 2.27 |
| P19536 | Cytochrome c oxidase subunit 5B, mitochondrial | 0.55 |
| P19973 | Lymphocyte-specific protein 1 | 0.47 |
| P20029 | 78 kDa glucose-regulated protein | 1.56 |
| P20060 | Beta-hexosaminidase subunit beta | 0.50 |
| P20152 | Vimentin | 2.12 |
| P20491 | High affinity immunoglobulin epsilon receptor subunit gamma | 2.29 |
| P21460 | Cystatin-C | 0.41 |
| P24270 | Catalase | 2.65 |
| P24860 | G2/mitotic-specific cyclin-B1 | 0.53 |
| P25799 | Nuclear factor NF-kappa-B p105 subunit;Nuclear factor NF-kappa-B p50 subunit | 1.84 |
| P26041 | Moesin | 1.79 |
| P26231 | Catenin alpha-1 | 2.62 |
| P27512 | Tumor necrosis factor receptor superfamily member 5 | 9.26 |
| P27870 | Proto-oncogene vav | 1.59 |
| P28033 | CCAAT/enhancer-binding protein beta | 1.65 |
| P28667 | MARCKS-related protein | 3.46 |
| P29452 | Caspase-1;Caspase-1 subunit p20;Caspase-1 subunit p10 | 2.05 |
| P30204 | Macrophage scavenger receptor types I and II | 1.89 |
| P30412 | Peptidyl-prolyl cis-trans isomerase C | 1.66 |
| P30658 | Chromobox protein homolog 2 | 0.51 |
| P31324 | cAMP-dependent protein kinase type II-beta regulatory subunit | 1.66 |
| P33174 | Chromosome-associated kinesin KIF4 | 0.36 |
| P33610 | DNA primase large subunit | 0.39 |
| P35700 | Peroxiredoxin-1 | 4.04 |
| P35761 | Dual specificity protein kinase TTK | 0.53 |
| P35762 | CD81 antigen | 0.56 |
| P35831 | Tyrosine-protein phosphatase non-receptor type 12 | 1.77 |
| P35951 | Low-density lipoprotein receptor | 1.86 |
| P36371 | Antigen peptide transporter 2 | 1.56 |
| P37040 | NADPH--cytochrome P450 reductase | 1.76 |
| P41216 | Long-chain-fatty-acid--CoA ligase 1 | 1.90 |
| P42230 | Signal transducer and activator of transcription 5A | 0.49 |
| P43024 | Cytochrome c oxidase subunit 6A1, mitochondrial;Cytochrome c oxidase subunit 6A, mitochondrial | 0.42 |
| P43276 | Histone H1.5 | 0.54 |
| P43277 | Histone H1.3 | 0.39 |
| P45377 | Aldose reductase-related protein 2 | 1.54 |
| P47791 | Glutathione reductase, mitochondrial | 2.28 |
| P48025 | Tyrosine-protein kinase SYK | 2.44 |
| P48036 | Annexin A5 | 1.72 |
| P48722 | Heat shock 70 kDa protein 4L | 1.79 |
| P48760 | Folylpolyglutamate synthase, mitochondrial | 0.55 |
| P48771 | Cytochrome c oxidase subunit 7A2, mitochondrial | 0.51 |
| P49282 | Natural resistance-associated macrophage protein 2 | 4.53 |
| P49710 | Hematopoietic lineage cell-specific protein | 2.31 |
| P50096 | Inosine-5-monophosphate dehydrogenase 1;Inosine-5-monophosphate dehydrogenase | 1.83 |
| P50396 | Rab GDP dissociation inhibitor alpha | 1.75 |
| P50543 | Protein S100-A11 | 2.22 |
| P51163 | Uroporphyrinogen-III synthase | 1.62 |
| P51943 | Cyclin-A2 | 0.52 |
| P52293 | Importin subunit alpha-2 | 0.48 |
| P52483 | Ubiquitin-conjugating enzyme E2 E3 | 0.41 |
| P54227 | Stathmin | 0.48 |
| P54729 | NEDD8 ultimate buster 1 | 1.88 |
| P54987 | Immune-responsive gene 1 protein | 40.67 |
| P56391 | Cytochrome c oxidase subunit 6B1 | 0.47 |
| P56873 | Sjoegren syndrome/scleroderma autoantigen 1 homolog | 1.86 |
| P57080 | Ubiquitin carboxyl-terminal hydrolase 25 | 1.77 |
| P58681 | Toll-like receptor 7 | 0.50 |
| P59108 | Copine-2 | 1.89 |
| P59328 | WD repeat and HMG-box DNA-binding protein 1 | 0.55 |
| P60330 | Separin | 0.35 |
| P61025 | Cyclin-dependent kinases regulatory subunit 1 | 0.40 |
| P61027 | Ras-related protein Rab-10 | 1.79 |
| P62342 | Selenoprotein T | 1.61 |
| P63260 | Actin, cytoplasmic 2;Actin, cytoplasmic 2, N-terminally processed | 1.75 |
| P70261 | Paladin | 11.31 |
| P70460 | Vasodilator-stimulated phosphoprotein | 2.08 |
| P81117 | Nucleobindin-2;Nesfatin-1 | 1.74 |
| P83741 | Serine/threonine-protein kinase WNK1 | 6.14 |
| P97313 | DNA-dependent protein kinase catalytic subunit | 14.91 |
| P97333 | Neuropilin-1 | 0.47 |
| P97369 | Neutrophil cytosol factor 4 | 1.73 |
| P97429 | Annexin A4 | 2.00 |
| P97471 | Mothers against decapentaplegic homolog 4 | 3.24 |
| P97499 | Telomerase protein component 1 | 1.92 |
| P97783 | Protein AF1q | 2.31 |
| P98078 | Disabled homolog 2 | 0.49 |
| P99029 | Peroxiredoxin-5, mitochondrial | 3.13 |
| Q000W5 |  | 4.60 |
| Q00612 | Glucose-6-phosphate 1-dehydrogenase X | 2.04 |
| Q01320 | DNA topoisomerase 2-alpha | 0.55 |
| Q04899 | Cyclin-dependent kinase 18 | 1.59 |
| Q05144 | Ras-related C3 botulinum toxin substrate 2 | 1.86 |
| Q05769 | Prostaglandin G/H synthase 2 | 5.19 |
| Q07076 | Annexin A7 | 1.74 |
| Q07113 | Cation-independent mannose-6-phosphate receptor | 2.04 |
| Q08619 | Interferon-activable protein 205-B | 5.71 |
| Q09200 | Beta-1,4 N-acetylgalactosaminyltransferase 1 | 0.51 |
| Q2EMV9 | Poly [ADP-ribose] polymerase 14 | 1.89 |
| Q3TBA3 | Antigen peptide transporter 1 | 2.11 |
| Q3TBV5 | Interleukin-1 receptor antagonist protein | 9.79 |
| Q3TIR3 | Synembryn-A | 1.69 |
| Q3TRM8 | Hexokinase-3 | 2.54 |
| Q3TZ86 | C5a anaphylatoxin chemotactic receptor | 1.61 |
| Q3U0X8 | T-cell surface protein tactile | 0.13 |
| Q3U2C5 | E3 ubiquitin-protein ligase RNF149 | 2.78 |
| Q3U319 | E3 ubiquitin-protein ligase BRE1B | 1.55 |
| Q3U4X8 | DNA ligase | 0.55 |
| Q3U5Q7 | UMP-CMP kinase 2, mitochondrial | 9.03 |
| Q3U7K7 | E3 ubiquitin-protein ligase TRIM21 | 1.91 |
| Q3U7R1 | Extended synaptotagmin-1 | 2.40 |
| Q3UDE9 | Protein-tyrosine kinase 2-beta | 2.00 |
| Q3UFB2 | Box C/D snoRNA protein 1 | 0.55 |
| Q3UGP9 | Leucine-rich repeat-containing protein 58 | 0.26 |
| Q3UHJ0 | AP2-associated protein kinase 1 | 1.78 |
| Q3UHX9 | Uncharacterized protein C9orf114 homolog | 1.95 |
| Q3UIR3 | E3 ubiquitin-protein ligase DTX3L | 2.04 |
| Q3ULB1 | Testin | 1.93 |
| Q3UND0 | Src kinase-associated phosphoprotein 2 | 2.33 |
| Q3UVG3 | Protein FAM91A1 | 0.51 |
| Q4FZC9 | Nesprin-3 | 0.44 |
| Q4LDD4 | Arf-GAP with Rho-GAP domain, ANK repeat and PH domain-containing protein 1 | 2.26 |
| Q542I8 | Integrin beta;Integrin beta-2 | 0.55 |
| Q5DU37 | Zinc finger FYVE domain-containing protein 26 | 1.62 |
| Q5DW34 | Histone-lysine N-methyltransferase EHMT1 | 0.50 |
| Q5NBU8 | XIAP-associated factor 1 | 2.24 |
| Q5NCB5 |  | 2.21 |
| Q5PSV9 | Mediator of DNA damage checkpoint protein 1 | 0.47 |
| Q5SFM8 | RNA-binding protein 27 | 0.11 |
| Q5SU47 | Cytoplasmic polyadenylation element-binding protein 4 | 1.97 |
| Q5XG73 | Acyl-CoA-binding domain-containing protein 5 | 1.70 |
| Q60648 | Ganglioside GM2 activator | 0.51 |
| Q60710 | SAM domain and HD domain-containing protein 1 | 1.73 |
| Q60787 | Lymphocyte cytosolic protein 2 | 2.06 |
| Q60837 | Interleukin-12 receptor subunit beta-1 | 9.61 |
| Q60848 | Lymphocyte-specific helicase | 0.46 |
| Q61033 | Lamina-associated polypeptide 2, isoforms alpha/zeta | 0.51 |
| Q61107 | Guanylate-binding protein 4 | 3.69 |
| Q61127 | NGFI-A-binding protein 2 | 0.45 |
| Q61187 | Tumor susceptibility gene 101 protein | 2.64 |
| Q61285 | ATP-binding cassette sub-family D member 2 | 0.50 |
| Q61333 | Tumor necrosis factor alpha-induced protein 2 | 2.34 |
| Q61549 | EGF-like module-containing mucin-like hormone receptor-like 1 | 2.21 |
| Q61686 | Chromobox protein homolog 5 | 0.50 |
| Q61735 | Leukocyte surface antigen CD47 | 1.61 |
| Q62048 | Astrocytic phosphoprotein PEA-15 | 1.55 |
| Q62159 | Rho-related GTP-binding protein RhoC | 2.85 |
| Q62176 | RNA-binding protein 38 | 0.51 |
| Q62351 | Transferrin receptor protein 1 | 0.31 |
| Q62407 | Striated muscle-specific serine/threonine-protein kinase | 0.52 |
| Q62425 | NADH dehydrogenase [ubiquinone] 1 alpha subcomplex subunit 4 | 0.35 |
| Q62433 | Protein NDRG1 | 0.44 |
| Q64281 | Leukocyte immunoglobulin-like receptor subfamily B member 4 | 1.61 |
| Q64282 | Interferon-induced protein with tetratricopeptide repeats 1 | 6.55 |
| Q64337 | Sequestosome-1 | 11.40 |
| Q64339 | Ubiquitin-like protein ISG15 | 6.83 |
| Q64345 | Interferon-induced protein with tetratricopeptide repeats 3 | 7.61 |
| Q64695 | Endothelial protein C receptor | 5.81 |
| Q64701 | Retinoblastoma-like protein 1 | 0.46 |
| Q68ED3 | PAP-associated domain-containing protein 5 | 3.68 |
| Q69ZN7 | Myoferlin | 1.89 |
| Q6DFV1 | Condensin-2 complex subunit G2 | 0.47 |
| Q6GTM0 | Interferon-induced protein with tetratricopeptide repeats 2 | 6.60 |
| Q6IRU2 | Tropomyosin alpha-4 chain | 2.46 |
| Q6NXL1 |  | 2.10 |
| Q6P9P6 | Kinesin-like protein KIF11 | 0.39 |
| Q6PAR0 | Kelch domain-containing protein 10 | 1.72 |
| Q6PB44 | Tyrosine-protein phosphatase non-receptor type 23 | 1.59 |
| Q6PFQ7 | Ras GTPase-activating protein 4 | 3.77 |
| Q6Q899 | Probable ATP-dependent RNA helicase DDX58 | 2.41 |
| Q6RT24 | Centromere-associated protein E | 6.83 |
| Q6WKZ8 | E3 ubiquitin-protein ligase UBR2 | 1.91 |
| Q6WVG3 | BTB/POZ domain-containing protein KCTD12 | 2.46 |
| Q6Y685 | Transforming acidic coiled-coil-containing protein 1 | 0.38 |
| Q6ZQF0 | DNA topoisomerase 2-binding protein 1 | 0.45 |
| Q6ZQK0 | Condensin-2 complex subunit D3 | 0.47 |
| Q6ZQM8 | UDP-glucuronosyltransferase 1-7C | 1.65 |
| Q6ZWQ9 |  | 1.74 |
| Q7JCZ1 | Cytochrome c oxidase subunit 2 | 0.54 |
| Q7TMM9 | Tubulin beta-2A chain | 1.78 |
| Q80SU7 | Interferon-induced very large GTPase 1 | 3.01 |
| Q80SY4 | E3 ubiquitin-protein ligase MIB1 | 3.47 |
| Q80X90 | Filamin-B | 1.59 |
| Q80Y19 | Rho GTPase-activating protein 11A | 0.35 |
| Q8BG07 | Phospholipase D4 | 0.54 |
| Q8BGC4 | Zinc-binding alcohol dehydrogenase domain-containing protein 2 | 1.72 |
| Q8BGQ7 | Alanine--tRNA ligase, cytoplasmic | 1.67 |
| Q8BHZ0 | Protein FAM49A | 1.78 |
| Q8BIG4 | F-box only protein 28 | 0.53 |
| Q8BJ48 | N-acetylglucosamine-1-phosphodiester alpha-N-acetylglucosaminidase | 0.54 |
| Q8BJS4 | SUN domain-containing protein 2 | 0.42 |
| Q8BJY1 | 26S proteasome non-ATPase regulatory subunit 5 | 1.59 |
| Q8BMD8 | Calcium-binding mitochondrial carrier protein SCaMC-1 | 1.75 |
| Q8BMP6 | Golgi resident protein GCP60 | 1.87 |
| Q8BN21 | Serine/threonine-protein kinase VRK2 | 0.46 |
| Q8BP92 | Reticulocalbin-2 | 3.04 |
| Q8BPX9 | Solute carrier family 15 member 3 | 6.30 |
| Q8BT07 | Centrosomal protein of 55 kDa | 0.34 |
| Q8BTV2 | Cleavage and polyadenylation specificity factor subunit 7 | 2.51 |
| Q8BTX9 | Inactive hydroxysteroid dehydrogenase-like protein 1 | 2.63 |
| Q8BTZ7 | Mannose-1-phosphate guanyltransferase beta | 1.78 |
| Q8BV49 | Pyrin and HIN domain-containing protein 1 | 3.61 |
| Q8BVA5 | UPF0554 protein C2orf43 homolog | 1.91 |
| Q8BVK9 | Sp110 nuclear body protein | 1.75 |
| Q8BXA1 | Golgi integral membrane protein 4 | 1.58 |
| Q8BYW1 | Rho GTPase-activating protein 25 | 0.47 |
| Q8C0D5 | Elongation factor Tu GTP-binding domain-containing protein 1 | 1.56 |
| Q8C0L6 | Peroxisomal N(1)-acetyl-spermine/spermidine oxidase | 0.45 |
| Q8C0Y0 | Serine/threonine-protein phosphatase 4 regulatory subunit 4 | 1.66 |
| Q8C1E7 | Transmembrane protein 120A | 1.76 |
| Q8C2K1 | Differentially expressed in FDCP 6 | 1.92 |
| Q8C3V4 | Signal transducer and activator of transcription 1 | 2.60 |
| Q8C5W3 | Tubulin-specific chaperone cofactor E-like protein | 1.68 |
| Q8C9X3 | DNA-binding protein Ikaros | 0.36 |
| Q8CAS9 | Poly [ADP-ribose] polymerase 9 | 1.88 |
| Q8CB96 | Ras association domain-containing protein 4 | 1.98 |
| Q8CBB9 | Radical S-adenosyl methionine domain-containing protein 2 | 5.51 |
| Q8CC88 | von Willebrand factor A domain-containing protein 8 | 2.35 |
| Q8CD15 | Bifunctional lysine-specific demethylase and histidyl-hydroxylase MINA | 1.85 |
| Q8CE80 | Calpastatin | 1.79 |
| Q8CI59 | Metalloreductase STEAP3 | 0.36 |
| Q8CIE4 |  | 2.28 |
| Q8CIM5 | G-protein coupled receptor 84 | 4.13 |
| Q8K078 | Solute carrier organic anion transporter family member 4A1 | 1.95 |
| Q8K0C4 | Lanosterol 14-alpha demethylase | 0.55 |
| Q8K0S9 | snRNA-activating protein complex subunit 1 | 24.23 |
| Q8K1C9 | Leucine-rich repeat-containing protein 41 | 0.56 |
| Q8K1T1 | Leucine-rich repeat-containing protein 25 | 4.17 |
| Q8K284 | General transcription factor 3C polypeptide 1 | 9.75 |
| Q8K298 | Actin-binding protein anillin | 0.47 |
| Q8K2T8 | RNA polymerase II-associated factor 1 homolog | 4.01 |
| Q8K337 | Type II inositol 1,4,5-trisphosphate 5-phosphatase | 2.53 |
| Q8K352 | SAM and SH3 domain-containing protein 3 | 0.52 |
| Q8K3H0 | DCC-interacting protein 13-alpha | 4.26 |
| Q8K4I3 | Rho guanine nucleotide exchange factor 6 | 1.53 |
| Q8K595 | Mucolipin-2 | 1.88 |
| Q8R105 | Vacuolar protein sorting-associated protein 37C | 2.22 |
| Q8R180 | ERO1-like protein alpha | 1.64 |
| Q8R1S0 | Ubiquinone biosynthesis monooxygenase COQ6 | 0.51 |
| Q8R2Q8 | Bone marrow stromal antigen 2 | 2.29 |
| Q8R3I3 | Conserved oligomeric Golgi complex subunit 6 | 2.00 |
| Q8R5F7 | Interferon-induced helicase C domain-containing protein 1 | 2.98 |
| Q8VCL2 | Protein SCO2 homolog, mitochondrial | 2.44 |
| Q8VDF2 | E3 ubiquitin-protein ligase UHRF1 | 0.48 |
| Q8VDK1 | Nitrilase homolog 1 | 1.63 |
| Q8VDQ8 | NAD-dependent protein deacetylase sirtuin-2 | 1.69 |
| Q8VDS8 | Syntaxin-18 | 2.99 |
| Q8VE62 | Polyadenylate-binding protein-interacting protein 1 | 3.74 |
| Q8VHN8 | Protein syndesmos | 0.49 |
| Q8VI93 | 2-5-oligoadenylate synthase 3 | 2.22 |
| Q8VI94 | 2-5-oligoadenylate synthase-like protein 1 | 7.55 |
| Q91VC9 | Growth hormone-inducible transmembrane protein | 2.05 |
| Q91VL8 | Telomeric repeat-binding factor 2-interacting protein 1 | 0.44 |
| Q91VL9 | Zinc finger and BTB domain-containing protein 1 | 0.55 |
| Q91VY9 | Zinc finger protein 622 | 1.90 |
| Q91XB0 | Three prime repair exonuclease 1 | 3.46 |
| Q91XV3 | Brain acid soluble protein 1 | 1.91 |
| Q91YR9 | Prostaglandin reductase 1 | 2.90 |
| Q91YS8 | Calcium/calmodulin-dependent protein kinase type 1 | 0.49 |
| Q91Z40 |  | 1.71 |
| Q91Z49 | UAP56-interacting factor | 1.91 |
| Q91ZU6 | Dystonin | 2.64 |
| Q91ZX7 | Prolow-density lipoprotein receptor-related protein 1 | 0.41 |
| Q920Q8 | Influenza virus NS1A-binding protein homolog | 0.40 |
| Q922S8 | Kinesin-like protein KIF2C | 0.29 |
| Q923D2 | Flavin reductase (NADPH) | 3.69 |
| Q93092 | Transaldolase | 1.64 |
| Q99J62 | Replication factor C subunit 4 | 0.54 |
| Q99K51 | Plastin-3 | 2.17 |
| Q99KQ4 | Nicotinamide phosphoribosyltransferase | 1.54 |
| Q99LC8 | Translation initiation factor eIF-2B subunit alpha | 3.84 |
| Q99LE1 | RILP-like protein 2 | 2.35 |
| Q99LH2 | Phosphatidylserine synthase 1 | 7.36 |
| Q99LW6 | YY1-associated factor 2 | 0.53 |
| Q99P65 | Equilibrative nucleoside transporter 3 | 0.45 |
| Q99P72 | Reticulon-4 | 1.64 |
| Q9CQ01 | Ribonuclease T2 | 2.34 |
| Q9CQ43 |  | 0.45 |
| Q9CQ75 | NADH dehydrogenase [ubiquinone] 1 alpha subcomplex subunit 2 | 0.54 |
| Q9CQE5 | Regulator of G-protein signaling 10 | 0.42 |
| Q9CQF6 | L-aminoadipate-semialdehyde dehydrogenase-phosphopantetheinyl transferase | 0.33 |
| Q9CQX4 | PCNA-associated factor | 0.27 |
| Q9CR29 | Coiled-coil domain-containing protein 43 | 3.88 |
| Q9CZN8 | Glutamyl-tRNA(Gln) amidotransferase subunit A, mitochondrial | 2.65 |
| Q9D0F6 | Replication factor C subunit 5 | 0.47 |
| Q9D0F9 | Phosphoglucomutase-1 | 1.60 |
| Q9D0J4 | ADP-ribosylation factor-like protein 2 | 4.25 |
| Q9D154 | Leukocyte elastase inhibitor A | 0.33 |
| Q9D1A2 | Cytosolic non-specific dipeptidase | 1.64 |
| Q9D2Y4 | Mixed lineage kinase domain-like protein | 2.11 |
| Q9D3W4 | GPN-loop GTPase 3 | 0.56 |
| Q9D4R6 |  | 0.20 |
| Q9D6N1 | Carbonic anhydrase 13 | 7.49 |
| Q9D6Y9 | 1,4-alpha-glucan-branching enzyme | 2.86 |
| Q9D7I8 | Protein FAM83D | 1.66 |
| Q9D7S9 | Charged multivesicular body protein 5 | 1.63 |
| Q9D7X8 | Gamma-glutamylcyclotransferase | 2.01 |
| Q9D8C4 | Interferon-induced 35 kDa protein homolog | 1.71 |
| Q9D8S3 | ADP-ribosylation factor GTPase-activating protein 3 | 1.76 |
| Q9D964 | Glycine amidinotransferase, mitochondrial | 0.52 |
| Q9D975 | Sulfiredoxin-1 | 10.74 |
| Q9DB40 | Mediator of RNA polymerase II transcription subunit 27 | 0.47 |
| Q9DBB8 | Trans-1,2-dihydrobenzene-1,2-diol dehydrogenase | 2.08 |
| Q9DBG7 | Signal recognition particle receptor subunit alpha | 1.94 |
| Q9DBH0 | NEDD4-like E3 ubiquitin-protein ligase WWP2 | 2.50 |
| Q9DCD0 | 6-phosphogluconate dehydrogenase, decarboxylating | 1.97 |
| Q9DCE9 |  | 2.34 |
| Q9DCN2 | NADH-cytochrome b5 reductase 3 | 1.58 |
| Q9EP73 | Programmed cell death 1 ligand 1 | 4.12 |
| Q9EP82 | tRNA (guanine-N(7)-)-methyltransferase subunit WDR4 | 3.76 |
| Q9EQ32 | Phosphoinositide 3-kinase adapter protein 1 | 1.97 |
| Q9EQK5 | Major vault protein | 2.20 |
| Q9ER38 | Torsin-3A | 1.54 |
| Q9ERD8 | Gamma-parvin | 2.01 |
| Q9ESY9 | Gamma-interferon-inducible lysosomal thiol reductase | 0.50 |
| Q9JHK5 | Pleckstrin | 1.78 |
| Q9JI44 | DNA methyltransferase 1-associated protein 1 | 4.94 |
| Q9JI99 | Sphingosine-1-phosphate phosphatase 1 | 0.52 |
| Q9JIA7 | Sphingosine kinase 2 | 2.34 |
| Q9JII6 | Alcohol dehydrogenase [NADP(+)] | 1.68 |
| Q9JIP4 | Pannexin-1 | 1.78 |
| Q9JM90 | Signal-transducing adaptor protein 1 | 2.93 |
| Q9JMH6 | Thioredoxin reductase 1, cytoplasmic | 3.10 |
| Q9QUH0 | Glutaredoxin-1 | 1.65 |
| Q9QWR8 | Alpha-N-acetylgalactosaminidase | 2.25 |
| Q9QXJ2 | Signal transducer and activator of transcription 2 | 2.09 |
| Q9QXW0 | F-box/LRR-repeat protein 6 | 0.50 |
| Q9QYB1 | Chloride intracellular channel protein 4 | 1.62 |
| Q9QYH6 | Melanoma-associated antigen D1 | 0.49 |
| Q9QZK7 | Docking protein 3 | 10.92 |
| Q9QZU9 | Ubiquitin/ISG15-conjugating enzyme E2 L6 | 1.59 |
| Q9R059 | Four and a half LIM domains protein 3 | 1.53 |
| Q9R0E2 | Procollagen-lysine,2-oxoglutarate 5-dioxygenase 1 | 0.53 |
| Q9R0P3 | S-formylglutathione hydrolase | 4.23 |
| Q9R0P9 | Ubiquitin carboxyl-terminal hydrolase isozyme L1 | 3.84 |
| Q9R112 | Sulfide:quinone oxidoreductase, mitochondrial | 1.83 |
| Q9R1Q7 | Proteolipid protein 2 | 0.44 |
| Q9R1X4 | Protein timeless homolog | 0.46 |
| Q9R233 | Tapasin | 1.68 |
| Q9WTK5 | Nuclear factor NF-kappa-B p100 subunit;Nuclear factor NF-kappa-B p52 subunit | 2.66 |
| Q9WTR6 | Cystine/glutamate transporter | 5.53 |
| Q9WTV6 | Ubl carboxyl-terminal hydrolase 18 | 2.52 |
| Q9WU62 | Inner centromere protein | 0.40 |
| Q9WUK4 | Replication factor C subunit 2 | 0.48 |
| Q9WUM3 | Coronin-1B | 1.56 |
| Q9WUN2 | Serine/threonine-protein kinase TBK1 | 41.33 |
| Q9WVK4 | EH domain-containing protein 1 | 7.34 |
| Q9Z0E6 | Interferon-induced guanylate-binding protein 2 | 4.60 |
| Q9Z1B5 | Mitotic spindle assembly checkpoint protein MAD2A | 0.52 |
| Q9Z1S0 | Mitotic checkpoint serine/threonine-protein kinase BUB1 beta | 0.44 |
| Q9Z2F2 | 2-5-oligoadenylate synthase-like protein 2 | 2.26 |
| Q9Z2X2 | 26S proteasome non-ATPase regulatory subunit 10 | 1.95 |

**Supplemental Table 4: M2c State Differentially Expressed Proteins**. Protein Expression levels for all significant (p<0.05) proteins for the M2c activation state.

| Uniprot ID | Protein names | IL10 |
| --- | --- | --- |
| A2AHC4 | Calmodulin-regulated spectrin-associated protein 1 | 1.18 |
| A2AIV8 |  | 1.18 |
| A2AJG0 | Ribonuclease P protein subunit p38 | 1.18 |
| A2AKI5 | Integrin alpha-V;Integrin alpha-V heavy chain;Integrin alpha-V light chain | 0.66 |
| A2AQM9 | Nuclear receptor coactivator 6 | 1.18 |
| A2CF65 | C-type lectin domain family 10 member A | 0.71 |
| A3KMP2 | Tetratricopeptide repeat protein 38 | 1.25 |
| A6PWS5 |  | 0.70 |
| A7UAK4 | 6-phosphofructo-2-kinase/fructose-2,6-bisphosphatase 2 | 0.30 |
| A9C471 | Baculoviral IAP repeat-containing protein 1b | 1.25 |
| B1ARD6 |  | 0.48 |
| B1ARW4 | NADH dehydrogenase [ubiquinone] iron-sulfur protein 5 | 1.21 |
| B1ASZ3 | Glycerol kinase | 1.23 |
| B1ATZ0 | Hepatocyte growth factor-regulated tyrosine kinase substrate | 0.53 |
| B1AUB6 | Nuclear factor 1 | 0.76 |
| B2RXW8 |  | 0.12 |
| B8ZXI1 | Queuine tRNA-ribosyltransferase subunit QTRTD1 | 1.32 |
| D3YUG2 | DCN1-like protein 1 | 2.44 |
| D3YWQ8 | Protein kinase C eta type | 1.23 |
| D3YWS2 | Echinoderm microtubule-associated protein-like 2 | 0.29 |
| D3YX57 | Fanconi anemia group I protein homolog | 1.20 |
| D3Z016 | Proteasome assembly chaperone 4 | 0.30 |
| D3Z0V2 | Rho guanine nucleotide exchange factor 7 | 0.57 |
| D3Z2Q2 | Syntaxin-binding protein 5 | 0.25 |
| D3Z2V8 | Transport and Golgi organization 2 homolog | 0.52 |
| D3Z3X7 | GPN-loop GTPase 1 | 0.77 |
| D3Z4L7 | Biogenesis of lysosome-related organelles complex 1 subunit 1 | 0.36 |
| D3Z4W3 | Proline-rich AKT1 substrate 1 | 1.42 |
| D3Z585 | Guanine nucleotide exchange factor for Rab-3A | 1.36 |
| D3Z6I4 | Quinone oxidoreductase-like protein 1 | 1.35 |
| D3Z774 | Histone-lysine N-methyltransferase EZH2 | 1.19 |
| D6RG49 | Protein FAM76B | 1.29 |
| D6RGR2 | Rab GTPase-binding effector protein 2 | 1.26 |
| E0CX20 | Protein BUD31 homolog | 1.17 |
| E0CY74 |  | 1.24 |
| E9PWK1 | Epoxide hydrolase 1 | 0.74 |
| E9PXG6 |  | 1.41 |
| E9PYJ8 | Histone acetyltransferase p300 | 0.67 |
| E9PYX5 | Probable cation-transporting ATPase 13A2 | 0.77 |
| E9Q3T3 |  | 1.36 |
| E9Q4G8 | CD166 antigen | 0.43 |
| E9Q557 | Desmoplakin | 0.17 |
| E9Q616 |  | 0.76 |
| E9Q6L4 | TBC1 domain family member 1 | 1.26 |
| E9Q8I9 | Protein furry homolog | 1.22 |
| E9Q8V6 |  | 1.20 |
| E9QAF9 | Protein TANC1 | 0.78 |
| E9QKL6 |  | 1.39 |
| E9QKR1 | Protein enabled homolog | 1.30 |
| E9QN37 | Macrophage-expressed gene 1 protein | 0.28 |
| E9QN39 |  | 0.63 |
| E9QND8 | Atlastin-2 | 1.67 |
| F6TWM7 | FXYD domain-containing ion transport regulator 5 | 0.77 |
| F6YTC4 | Zinc transporter ZIP10 | 0.72 |
| F7BGR7 | RNA-binding protein 4 | 1.37 |
| F8VPN4 |  | 0.71 |
| F8VPP8 |  | 0.77 |
| F8WGT2 | Transmembrane protein 209 | 1.55 |
| F8WHZ9 | Alpha-adducin | 1.33 |
| G3UYL4 | G-protein-signaling modulator 3 | 0.36 |
| G3UYU5 | Ribonuclease P protein subunit p40 | 1.38 |
| G3UZ30 | Protein phosphatase 1 regulatory subunit 11 | 1.25 |
| G3UZY2 | Thioredoxin;Thioredoxin, mitochondrial | 0.70 |
| G3X9K3 | Brefeldin A-inhibited guanine nucleotide-exchange protein 1 | 1.24 |
| G3XA22 | rRNA-processing protein UTP23 homolog | 1.25 |
| G5E897 |  | 0.24 |
| G5E8R2 |  | 0.47 |
| H3BJG4 |  | 1.20 |
| I1E4X0 | Disks large-associated protein 4 | 2.25 |
| J3QMN4 | Thioredoxin reductase 2, mitochondrial | 0.72 |
| J3QPY0 | Protein C19orf12 homolog | 1.75 |
| O08600 | Endonuclease G, mitochondrial | 1.28 |
| O08739 | AMP deaminase 3 | 0.74 |
| O35114 | Lysosome membrane protein 2 | 0.70 |
| O35316 | Sodium- and chloride-dependent taurine transporter | 1.52 |
| O35326 | Serine/arginine-rich splicing factor 5 | 1.26 |
| O35704 | Serine palmitoyltransferase 1 | 0.08 |
| O55106 | Striatin | 0.77 |
| O55229 | Choline/ethanolamine kinase | 0.59 |
| O70422 | General transcription factor IIH subunit 4 | 0.64 |
| O70456 | 14-3-3 protein sigma | 0.09 |
| O70475 | UDP-glucose 6-dehydrogenase | 0.78 |
| O88325 |  | 0.72 |
| O88520 | Leucine-rich repeat protein SHOC-2 | 1.33 |
| O88712 | C-terminal-binding protein 1 | 1.70 |
| O88848 | ADP-ribosylation factor-like protein 6 | 1.26 |
| O88907 | E3 SUMO-protein ligase PIAS1 | 0.40 |
| O88967 | ATP-dependent zinc metalloprotease YME1L1 | 0.63 |
| O88983 | Syntaxin-8 | 1.18 |
| O89086 | Putative RNA-binding protein 3 | 1.26 |
| P01027 | Complement C3 | 0.34 |
| P01631 | Ig kappa chain V-II region 26-10 | 0.10 |
| P01901 | H-2 class I histocompatibility antigen, K-B alpha chain | 0.66 |
| P03888 | NADH-ubiquinone oxidoreductase chain 1 | 1.24 |
| P04184 | Thymidine kinase, cytosolic | 1.22 |
| P06797 | Cathepsin L1;Cathepsin L1 heavy chain;Cathepsin L1 light chain | 1.19 |
| P07356 | Annexin A2 | 0.72 |
| P08226 | Apolipoprotein E | 0.76 |
| P09528 | Ferritin heavy chain | 0.56 |
| P09581 | Macrophage colony-stimulating factor 1 receptor | 0.76 |
| P10605 | Cathepsin B;Cathepsin B light chain;Cathepsin B heavy chain | 1.34 |
| P10925 | Zinc finger Y-chromosomal protein 1 | 0.54 |
| P11928 | 2-5-oligoadenylate synthase 1A | 0.74 |
| P13020 | Gelsolin | 0.76 |
| P13597 | Intercellular adhesion molecule 1 | 1.36 |
| P18181 | CD48 antigen | 0.63 |
| P19182 | Interferon-related developmental regulator 1 | 1.44 |
| P20060 | Beta-hexosaminidase subunit beta | 0.74 |
| P20664 | DNA primase small subunit | 1.19 |
| P21279 | Guanine nucleotide-binding protein G(q) subunit alpha | 0.48 |
| P21460 | Cystatin-C | 0.77 |
| P21956 | Lactadherin | 0.68 |
| P23249 | Putative helicase MOV-10 | 0.75 |
| P26231 | Catenin alpha-1 | 1.40 |
| P28033 | CCAAT/enhancer-binding protein beta | 1.42 |
| P29416 | Beta-hexosaminidase subunit alpha | 0.72 |
| P30730 | Lutropin-choriogonadotropic hormone receptor | 0.73 |
| P35290 | Ras-related protein Rab-24 | 0.68 |
| P41731 | CD63 antigen | 0.74 |
| P42227 | Signal transducer and activator of transcription 3 | 1.41 |
| P42230 | Signal transducer and activator of transcription 5A | 0.70 |
| P45591 | Cofilin-2 | 0.76 |
| P46718 | Programmed cell death protein 2 | 1.32 |
| P47713 | Cytosolic phospholipase A2;Phospholipase A2;Lysophospholipase | 1.20 |
| P48410 | ATP-binding cassette sub-family D member 1 | 0.15 |
| P48725 | Pericentrin | 0.53 |
| P50096 | Inosine-5-monophosphate dehydrogenase 1;Inosine-5-monophosphate dehydrogenase | 1.23 |
| P50396 | Rab GDP dissociation inhibitor alpha | 0.70 |
| P51175 | Protoporphyrinogen oxidase | 0.72 |
| P51943 | Cyclin-A2 | 1.29 |
| P53996 | Cellular nucleic acid-binding protein | 1.21 |
| P54731 | FAS-associated factor 1 | 0.42 |
| P54987 | Immune-responsive gene 1 protein | 2.62 |
| P55097 | Cathepsin K | 0.74 |
| P56873 | Sjoegren syndrome/scleroderma autoantigen 1 homolog | 1.22 |
| P58044 | Isopentenyl-diphosphate Delta-isomerase 1 | 1.32 |
| P58468 | Protein FAM207A | 1.18 |
| P58501 | PAX3- and PAX7-binding protein 1 | 1.26 |
| P59325 | Eukaryotic translation initiation factor 5 | 1.26 |
| P59326 | YTH domain family protein 1 | 1.34 |
| P60003 | Transcription elongation factor 1 homolog | 1.21 |
| P60670 | Nuclear protein localization protein 4 homolog | 1.18 |
| P60840 | Alpha-endosulfine | 16.95 |
| P60904 | DnaJ homolog subfamily C member 5 | 1.24 |
| P61025 | Cyclin-dependent kinases regulatory subunit 1 | 1.28 |
| P61211 | ADP-ribosylation factor-like protein 1 | 1.25 |
| P61222 | ATP-binding cassette sub-family E member 1 | 1.24 |
| P70188 | Kinesin-associated protein 3 | 0.39 |
| P83741 | Serine/threonine-protein kinase WNK1 | 5.89 |
| P97304 | DNA-directed RNA polymerases I and III subunit RPAC2 | 1.68 |
| P97350 | Plakophilin-1 | 0.74 |
| P97449 | Aminopeptidase N | 0.68 |
| P97742 | Carnitine O-palmitoyltransferase 1, liver isoform | 1.18 |
| P97789 | 5-3 exoribonuclease 1 | 0.72 |
| Q00519 | Xanthine dehydrogenase/oxidase;Xanthine dehydrogenase;Xanthine oxidase | 0.74 |
| Q00899 | Transcriptional repressor protein YY1 | 1.24 |
| Q02257 | Junction plakoglobin | 0.07 |
| Q04447 | Creatine kinase B-type | 0.76 |
| Q07797 | Galectin-3-binding protein | 0.69 |
| Q0KL02 | Triple functional domain protein | 1.18 |
| Q0VBD2 | Protein MCM10 homolog | 1.24 |
| Q3T9A5 | Glomulin | 1.23 |
| Q3TAA7 | Serine/threonine-protein kinase 11-interacting protein | 0.77 |
| Q3TBU7 | Arf-GAP domain and FG repeat-containing protein 2 | 0.35 |
| Q3TBV5 | Interleukin-1 receptor antagonist protein | 1.28 |
| Q3TCX3 | UPF0469 protein KIAA0907 | 0.77 |
| Q3TH73 | Protein tweety homolog 2 | 0.74 |
| Q3THJ3 | Probable RNA-binding protein EIF1AD | 1.39 |
| Q3TIR3 | Synembryn-A | 0.59 |
| Q3TIU4 | 2,5-phosphodiesterase 12 | 1.22 |
| Q3TMX7 | Sulfhydryl oxidase 2 | 1.20 |
| Q3TQ29 | Pumilio homolog 2 | 1.46 |
| Q3TQI7 | Uncharacterized protein C9orf78 homolog | 1.29 |
| Q3TXU5 | Deoxyhypusine synthase | 1.25 |
| Q3U3T8 | WD repeat-containing protein 62 | 1.30 |
| Q3U5Q7 | UMP-CMP kinase 2, mitochondrial | 1.58 |
| Q3UIR3 | E3 ubiquitin-protein ligase DTX3L | 1.30 |
| Q3ULD5 | Methylcrotonoyl-CoA carboxylase beta chain, mitochondrial | 0.63 |
| Q3UMW8 | Ceroid-lipofuscinosis neuronal protein 5 homolog | 3.60 |
| Q3UND0 | Src kinase-associated phosphoprotein 2 | 1.29 |
| Q3URS9 | Coiled-coil domain-containing protein 51 | 0.75 |
| Q3UYC0 | Protein phosphatase 1H | 0.72 |
| Q5NCQ5 | Diphthamide biosynthesis protein 1 | 1.40 |
| Q5RL57 | A-kinase anchor protein 8-like | 1.77 |
| Q5SVQ0 | Histone acetyltransferase KAT7 | 1.26 |
| Q5SXA5 | TOM1-like protein 2 | 0.68 |
| Q5U4F6 | WD repeat-containing protein 34 | 1.22 |
| Q5U680 | S-adenosylmethionine mitochondrial carrier protein | 0.74 |
| Q60738 | Zinc transporter 1 | 1.41 |
| Q60787 | Lymphocyte cytosolic protein 2 | 0.13 |
| Q60809 | CCR4-NOT transcription complex subunit 7 | 1.39 |
| Q60838 | Segment polarity protein dishevelled homolog DVL-2 | 1.45 |
| Q60876 | Eukaryotic translation initiation factor 4E-binding protein 1 | 1.40 |
| Q60996 | Serine/threonine-protein phosphatase 2A 56 kDa regulatory subunit gamma isoform | 1.19 |
| Q61239 | Protein farnesyltransferase/geranylgeranyltransferase type-1 subunit alpha | 0.60 |
| Q61333 | Tumor necrosis factor alpha-induced protein 2 | 1.21 |
| Q61462 | Cytochrome b-245 light chain | 1.18 |
| Q61542 | StAR-related lipid transfer protein 3 | 0.64 |
| Q61584 | Fragile X mental retardation syndrome-related protein 1 | 1.20 |
| Q61823 | Programmed cell death protein 4 | 0.77 |
| Q62241 | U1 small nuclear ribonucleoprotein C | 1.22 |
| Q62417 | Sorbin and SH3 domain-containing protein 1 | 1.19 |
| Q62432 | Mothers against decapentaplegic homolog 2 | 1.22 |
| Q62433 | Protein NDRG1 | 0.58 |
| Q62523 | Zyxin | 1.34 |
| Q63836 | Selenium-binding protein 2;Selenium-binding protein 1 | 0.62 |
| Q64281 | Leukocyte immunoglobulin-like receptor subfamily B member 4 | 0.76 |
| Q64282 | Interferon-induced protein with tetratricopeptide repeats 1 | 2.11 |
| Q64337 | Sequestosome-1 | 1.34 |
| Q64339 | Ubiquitin-like protein ISG15 | 1.18 |
| Q64345 | Interferon-induced protein with tetratricopeptide repeats 3 | 2.36 |
| Q64364 | Cyclin-dependent kinase inhibitor 2A, isoform 3 | 0.59 |
| Q64735 | Complement component receptor 1-like protein | 2.08 |
| Q69Z38 | Pseudopodium-enriched atypical kinase 1 | 1.18 |
| Q69ZA1 | Cyclin-dependent kinase 13 | 0.74 |
| Q6DID3 | Protein SCAF8 | 1.19 |
| Q6GQT1 | Alpha-2-macroglobulin-P | 0.52 |
| Q6GTM0 | Interferon-induced protein with tetratricopeptide repeats 2 | 2.88 |
| Q6IRU2 | Tropomyosin alpha-4 chain | 1.18 |
| Q6KAR6 | Exocyst complex component 3 | 0.26 |
| Q6P1F6 | Serine/threonine-protein phosphatase 2A 55 kDa regulatory subunit B alpha isoform | 1.21 |
| Q6PAR0 | Kelch domain-containing protein 10 | 1.20 |
| Q6PAV2 | Probable E3 ubiquitin-protein ligase HERC4 | 1.19 |
| Q6PDM2 | Serine/arginine-rich splicing factor 1 | 1.18 |
| Q6Q899 | Probable ATP-dependent RNA helicase DDX58 | 0.56 |
| Q6RT24 | Centromere-associated protein E | 0.69 |
| Q6XLQ8 |  | 0.67 |
| Q6ZPJ3 | Ubiquitin-conjugating enzyme E2 O | 1.21 |
| Q6ZQ03 | Formin-binding protein 4 | 1.32 |
| Q6ZQ18 | Protein EFR3 homolog B | 0.71 |
| Q6ZQB6 | Inositol hexakisphosphate and diphosphoinositol-pentakisphosphate kinase 2 | 1.58 |
| Q6ZQK5 | Arf-GAP with coiled-coil, ANK repeat and PH domain-containing protein 2 | 0.77 |
| Q6ZQL4 | WD repeat-containing protein 43 | 1.18 |
| Q6ZWY8 | Thymosin beta-10 | 0.69 |
| Q76LS9 | Protein FAM63A | 1.37 |
| Q78XR0 | Trafficking protein particle complex subunit 6A | 0.76 |
| Q791N7 | DNA-directed RNA polymerase I subunit RPA12 | 1.19 |
| Q7TMG8 | Protein NipSnap homolog 2 | 0.54 |
| Q7TMI3 | E3 ubiquitin-protein ligase UHRF2 | 1.28 |
| Q7TMV3 | FAST kinase domain-containing protein 5 | 1.26 |
| Q7TN29 | Stromal membrane-associated protein 2 | 1.83 |
| Q7TNC4 | Putative RNA-binding protein Luc7-like 2 | 1.18 |
| Q7TND5 | Ribosome production factor 1 | 1.23 |
| Q7TPE5 | Probable RNA polymerase II nuclear localization protein SLC7A6OS | 1.28 |
| Q80SU7 | Interferon-induced very large GTPase 1 | 1.22 |
| Q80TR8 | Protein VPRBP | 1.32 |
| Q80UK8 | Integrator complex subunit 2 | 1.34 |
| Q80UP3 | Diacylglycerol kinase zeta | 0.14 |
| Q80VP0 | Tectonin beta-propeller repeat-containing protein 1 | 0.65 |
| Q80ZS3 | 28S ribosomal protein S26, mitochondrial | 0.71 |
| Q811J3 | Iron-responsive element-binding protein 2 | 0.74 |
| Q8BFU3 | RING finger protein 214 | 1.26 |
| Q8BFV2 | PCI domain-containing protein 2 | 1.25 |
| Q8BFY6 | Peflin | 1.19 |
| Q8BG07 | Phospholipase D4 | 0.64 |
| Q8BGB5 | LIM domain-containing protein 2 | 1.20 |
| Q8BGD6 | Putative sodium-coupled neutral amino acid transporter 9 | 0.74 |
| Q8BGS0 | Protein MAK16 homolog | 1.44 |
| Q8BGU5 | Cyclin-Y | 0.74 |
| Q8BH40 | Syntaxin-7 | 1.45 |
| Q8BHB4 | WD repeat-containing protein 3 | 1.18 |
| Q8BHX3 | Borealin | 1.28 |
| Q8BHZ0 | Protein FAM49A | 0.70 |
| Q8BK35 |  | 1.40 |
| Q8BLD4 |  | 0.54 |
| Q8BLH7 | HIRA-interacting protein 3 | 0.74 |
| Q8BMQ2 | General transcription factor 3C polypeptide 4 | 1.25 |
| Q8BN21 | Serine/threonine-protein kinase VRK2 | 0.74 |
| Q8BTT6 | Digestive organ expansion factor homolog | 1.19 |
| Q8BTV2 | Cleavage and polyadenylation specificity factor subunit 7 | 1.53 |
| Q8BUM3 | Tyrosine-protein phosphatase non-receptor type 7 | 0.73 |
| Q8BVG4 | Dipeptidyl peptidase 9 | 0.27 |
| Q8BVI5 | Syntaxin-16 | 0.62 |
| Q8BVK9 | Sp110 nuclear body protein | 1.53 |
| Q8BVQ5 | Protein phosphatase methylesterase 1 | 0.75 |
| Q8BWU5 | Probable tRNA threonylcarbamoyladenosine biosynthesis protein Osgep | 1.30 |
| Q8BX09 | Retinoblastoma-binding protein 5 | 0.76 |
| Q8BX10 | Serine/threonine-protein phosphatase PGAM5, mitochondrial | 1.28 |
| Q8BX57 | PX domain-containing protein kinase-like protein | 0.53 |
| Q8BX94 | Oxysterol-binding protein-related protein 2 | 1.22 |
| Q8BXQ2 | GPI transamidase component PIG-T | 0.55 |
| Q8BYC6 | Serine/threonine-protein kinase TAO3 | 1.37 |
| Q8C0P5 | Coronin-2A;Coronin | 0.15 |
| Q8C3Q9 | Caspase-9;Caspase-9 subunit p35;Caspase-9 subunit p10 | 0.71 |
| Q8C460 | ERI1 exoribonuclease 3 | 0.57 |
| Q8C547 | HEAT repeat-containing protein 5B | 4.10 |
| Q8C6E0 | Coiled-coil domain-containing protein 104 | 1.22 |
| Q8C9T1 |  | 1.44 |
| Q8CBB9 | Radical S-adenosyl methionine domain-containing protein 2 | 2.22 |
| Q8CC12 | Codanin-1 | 1.19 |
| Q8CDM8 | Protein FAM160B1 | 0.41 |
| Q8CE46 | Pseudouridylate synthase 7 homolog-like protein | 1.22 |
| Q8CFK1 | Glycylpeptide N-tetradecanoyltransferase;Glycylpeptide N-tetradecanoyltransferase 2 | 0.43 |
| Q8CHC4 | Synaptojanin-1 | 0.17 |
| Q8CHI8 | E1A-binding protein p400 | 0.28 |
| Q8CI95 | Oxysterol-binding protein-related protein 11;Oxysterol-binding protein | 0.53 |
| Q8CIH9 | Amidophosphoribosyltransferase | 1.18 |
| Q8CIW5 | Twinkle protein, mitochondrial | 1.24 |
| Q8CJF7 | Protein ELYS | 0.76 |
| Q8K0E8 | Fibrinogen beta chain;Fibrinopeptide B;Fibrinogen beta chain | 0.08 |
| Q8K0V4 | CCR4-NOT transcription complex subunit 3 | 0.62 |
| Q8K284 | General transcription factor 3C polypeptide 1 | 1.35 |
| Q8K2L8 | Trafficking protein particle complex subunit 12 | 0.76 |
| Q8K3J1 | NADH dehydrogenase [ubiquinone] iron-sulfur protein 8, mitochondrial | 1.19 |
| Q8K3X4 | Interferon regulatory factor 2-binding protein-like | 1.31 |
| Q8QZY9 | Splicing factor 3B subunit 4 | 0.70 |
| Q8R0F8 | Acylpyruvase FAHD1, mitochondrial | 0.73 |
| Q8R0J7 | Vacuolar protein sorting-associated protein 37B | 0.74 |
| Q8R2N0 | Thyroid transcription factor 1-associated protein 26 | 1.18 |
| Q8R2U2 |  | 1.18 |
| Q8R3K3 | Pentatricopeptide repeat-containing protein 2, mitochondrial | 1.28 |
| Q8R3Y8 | Interferon regulatory factor 2-binding protein 1 | 1.21 |
| Q8R570 | Synaptosomal-associated protein 47 | 1.19 |
| Q8VBT6 | Apolipoprotein B receptor | 1.26 |
| Q8VBZ0 | Dehydrogenase/reductase SDR family member on chromosome X homolog | 0.69 |
| Q8VC03 | Echinoderm microtubule-associated protein-like 3 | 1.68 |
| Q8VC04 | Transmembrane protein 106A | 0.57 |
| Q8VCF0 | Mitochondrial antiviral-signaling protein | 0.76 |
| Q8VCL2 | Protein SCO2 homolog, mitochondrial | 1.21 |
| Q8VCM4 | Lipoyltransferase 1, mitochondrial | 0.61 |
| Q8VCM7 | Fibrinogen gamma chain | 0.04 |
| Q8VCR4 | tRNA methyltransferase 112 homolog | 1.85 |
| Q8VCW4 | Protein unc-93 homolog B1 | 0.75 |
| Q8VE38 | Oxidoreductase NAD-binding domain-containing protein 1 | 0.75 |
| Q8VE62 | Polyadenylate-binding protein-interacting protein 1 | 0.57 |
| Q8VEH8 | Endoplasmic reticulum lectin 1 | 0.72 |
| Q8VI84 | Nucleolar complex protein 3 homolog | 1.26 |
| Q8VI93 | 2-5-oligoadenylate synthase 3 | 0.77 |
| Q8VIM0 | Hepatitis A virus cellular receptor 2 homolog | 0.54 |
| Q91VE0 | Long-chain fatty acid transport protein 4 | 0.75 |
| Q91VY9 | Zinc finger protein 622 | 1.34 |
| Q91WC0 | Histone-lysine N-methyltransferase setd3 | 1.23 |
| Q91WG4 | Elongator complex protein 2 | 1.40 |
| Q91X78 | Erlin-1 | 0.76 |
| Q91YJ3 | Thymocyte nuclear protein 1 | 1.19 |
| Q91YN5 | UDP-N-acetylhexosamine pyrophosphorylase | 1.25 |
| Q91YR5 | Methyltransferase-like protein 13 | 1.26 |
| Q91YX0 | Protein THEMIS2 | 1.28 |
| Q91ZR2 | Sorting nexin-18 | 1.27 |
| Q921J4 | Ubiquitin-conjugating enzyme E2 S | 0.78 |
| Q922D4 | Serine/threonine-protein phosphatase 6 regulatory subunit 3 | 1.18 |
| Q922J3 | CAP-Gly domain-containing linker protein 1 | 0.49 |
| Q922Q2 | Serine/threonine-protein kinase RIO1 | 0.71 |
| Q922Q9 | Chitinase domain-containing protein 1 | 0.75 |
| Q924Z6 | Exportin-6 | 0.47 |
| Q99J99 | 3-mercaptopyruvate sulfurtransferase;Sulfurtransferase | 0.76 |
| Q99JH1 | Ribonuclease P protein subunit p25-like protein | 1.19 |
| Q99JR8 | SWI/SNF-related matrix-associated actin-dependent regulator of chromatin subfamily D member 2 | 1.19 |
| Q99K28 | ADP-ribosylation factor GTPase-activating protein 2 | 0.73 |
| Q99L04 | Dehydrogenase/reductase SDR family member 1 | 0.69 |
| Q99L48 | 60S ribosomal export protein NMD3 | 1.33 |
| Q99LH2 | Phosphatidylserine synthase 1 | 1.20 |
| Q99LM2 | CDK5 regulatory subunit-associated protein 3 | 0.67 |
| Q99LS3 | Phosphoserine phosphatase | 1.37 |
| Q99M07 | Cytochrome c oxidase assembly factor 5 | 1.18 |
| Q99M08 | Uncharacterized protein C4orf3 homolog | 0.53 |
| Q99MR3 | Solute carrier family 12 member 9 | 0.76 |
| Q99MX0 | Transketolase-like protein 1 | 1.21 |
| Q99N92 | 39S ribosomal protein L27, mitochondrial | 0.32 |
| Q99P65 | Equilibrative nucleoside transporter 3 | 0.77 |
| Q9CPV7 | Palmitoyltransferase ZDHHC6 | 1.27 |
| Q9CPW3 | 39S ribosomal protein L54, mitochondrial | 0.73 |
| Q9CPW7 | Zinc finger matrin-type protein 2 | 0.06 |
| Q9CPX4 | Ferritin | 0.51 |
| Q9CPX7 | 28S ribosomal protein S16, mitochondrial | 1.18 |
| Q9CQ75 | NADH dehydrogenase [ubiquinone] 1 alpha subcomplex subunit 2 | 1.20 |
| Q9CQ86 | Migration and invasion enhancer 1 | 0.26 |
| Q9CQ91 | NADH dehydrogenase [ubiquinone] 1 alpha subcomplex subunit 3 | 0.72 |
| Q9CQE5 | Regulator of G-protein signaling 10 | 0.77 |
| Q9CQF6 | L-aminoadipate-semialdehyde dehydrogenase-phosphopantetheinyl transferase | 1.18 |
| Q9CQL5 | 39S ribosomal protein L18, mitochondrial | 1.33 |
| Q9CQT5 | Proteasome maturation protein | 1.23 |
| Q9CQU5 | ZW10 interactor | 0.78 |
| Q9CQV5 | 28S ribosomal protein S24, mitochondrial | 0.61 |
| Q9CQZ0 | ORM1-like protein 2;ORM1-like protein 1 | 0.65 |
| Q9CR02 | Translation machinery-associated protein 16 | 1.19 |
| Q9CR26 | Vacuolar protein sorting-associated protein VTA1 homolog | 0.69 |
| Q9CR29 | Coiled-coil domain-containing protein 43 | 0.39 |
| Q9CR61 | NADH dehydrogenase [ubiquinone] 1 beta subcomplex subunit 7 | 0.73 |
| Q9CRY7 | Glycerophosphodiester phosphodiesterase domain-containing protein 1 | 0.66 |
| Q9CWF2 | Tubulin beta-2B chain | 0.70 |
| Q9CWN7 | CCR4-NOT transcription complex subunit 11 | 0.15 |
| Q9CWP6 | Motile sperm domain-containing protein 2 | 0.70 |
| Q9CWR1 | WD repeat-containing protein 73 | 0.56 |
| Q9CWU9 | Nucleoporin Nup37 | 1.24 |
| Q9CX00 | IST1 homolog | 0.68 |
| Q9CXI0 | 2-methoxy-6-polyprenyl-1,4-benzoquinol methylase, mitochondrial | 0.77 |
| Q9CY73 | 39S ribosomal protein L44, mitochondrial | 0.74 |
| Q9CY97 | RNA polymerase II subunit A C-terminal domain phosphatase SSU72 | 0.53 |
| Q9CYH2 | Redox-regulatory protein FAM213A | 0.74 |
| Q9CYN9 | Renin receptor | 1.17 |
| Q9CZ91 | Serum response factor-binding protein 1 | 1.27 |
| Q9CZN8 | Glutamyl-tRNA(Gln) amidotransferase subunit A, mitochondrial | 1.95 |
| Q9CZS1 | Aldehyde dehydrogenase X, mitochondrial | 1.28 |
| Q9CZU4 | GTPase Era, mitochondrial | 0.68 |
| Q9CZX0 | Elongator complex protein 3 | 1.20 |
| Q9D154 | Leukocyte elastase inhibitor A | 1.37 |
| Q9D168 | Integrator complex subunit 12 | 1.60 |
| Q9D1Q4 | Dolichol-phosphate mannosyltransferase subunit 3 | 1.44 |
| Q9D289 | Trafficking protein particle complex subunit 6B | 1.34 |
| Q9D338 | 39S ribosomal protein L19, mitochondrial | 0.73 |
| Q9D394 | Protein RUFY3 | 0.73 |
| Q9D483 | DNA-directed RNA polymerase III subunit RPC3 | 2.87 |
| Q9D6J6 | NADH dehydrogenase [ubiquinone] flavoprotein 2, mitochondrial | 1.20 |
| Q9D6Y7 | Mitochondrial peptide methionine sulfoxide reductase | 0.76 |
| Q9D6Z0 | Alpha-ketoglutarate-dependent dioxygenase alkB homolog 7 | 1.25 |
| Q9D7J4 | Cytochrome c oxidase protein 20 homolog | 0.77 |
| Q9D7S9 | Charged multivesicular body protein 5 | 1.21 |
| Q9D7X8 | Gamma-glutamylcyclotransferase | 1.30 |
| Q9D7Z3 | Nucleolar protein 7 | 1.28 |
| Q9D8S4 | Oligoribonuclease, mitochondrial | 1.24 |
| Q9D8T7 | SRA stem-loop-interacting RNA-binding protein, mitochondrial | 1.20 |
| Q9D902 | General transcription factor IIE subunit 2 | 0.68 |
| Q9D975 | Sulfiredoxin-1 | 2.02 |
| Q9DAK9 | 14 kDa phosphohistidine phosphatase | 0.73 |
| Q9DB40 | Mediator of RNA polymerase II transcription subunit 27 | 0.70 |
| Q9DB42 | Zinc finger protein 593 | 1.19 |
| Q9DB90 | Protein SMG9 | 0.43 |
| Q9DBA9 | General transcription factor IIH subunit 1 | 1.52 |
| Q9DBH0 | NEDD4-like E3 ubiquitin-protein ligase WWP2 | 1.36 |
| Q9DC70 | NADH dehydrogenase [ubiquinone] iron-sulfur protein 7, mitochondrial | 1.54 |
| Q9DCA2 | 28S ribosomal protein S11, mitochondrial | 1.18 |
| Q9DCI9 | 39S ribosomal protein L32, mitochondrial | 1.99 |
| Q9DCT1 | 1,5-anhydro-D-fructose reductase | 2.42 |
| Q9EP82 | tRNA (guanine-N(7)-)-methyltransferase subunit WDR4 | 1.22 |
| Q9EPB5 | Serine hydrolase-like protein | 0.74 |
| Q9EQC5 | N-terminal kinase-like protein | 0.57 |
| Q9EQJ0 | Two pore calcium channel protein 1 | 0.47 |
| Q9ER81 | 15 kDa interferon-responsive protein | 1.38 |
| Q9ERD8 | Gamma-parvin | 0.76 |
| Q9ERN0 | Secretory carrier-associated membrane protein 2 | 0.75 |
| Q9JHP7 | KDEL motif-containing protein 1 | 1.26 |
| Q9JI39 | ATP-binding cassette sub-family B member 10, mitochondrial | 0.44 |
| Q9JI99 | Sphingosine-1-phosphate phosphatase 1 | 0.63 |
| Q9JID9 | SH2B adapter protein 2 | 0.66 |
| Q9JIX0 | Enhancer of yellow 2 transcription factor homolog | 0.74 |
| Q9QWF0 | Chromatin assembly factor 1 subunit A | 0.78 |
| Q9QXD8 | LIM domain-containing protein 1 | 1.20 |
| Q9QYI3 | DnaJ homolog subfamily C member 7 | 1.19 |
| Q9QYL7 | Activator of basal transcription 1 | 1.22 |
| Q9QZ08 | N-acetyl-D-glucosamine kinase | 0.55 |
| Q9QZK7 | Docking protein 3 | 0.76 |
| Q9QZU9 | Ubiquitin/ISG15-conjugating enzyme E2 L6 | 0.77 |
| Q9R013 | Cathepsin F | 1.20 |
| Q9R0Q4 | Mortality factor 4-like protein 2 | 1.20 |
| Q9R0Q6 | Actin-related protein 2/3 complex subunit 1A | 0.67 |
| Q9R1Q7 | Proteolipid protein 2 | 0.54 |
| Q9WTY1 | Programmed cell death protein 7 | 1.25 |
| Q9WUB0 | RanBP-type and C3HC4-type zinc finger-containing protein 1 | 0.28 |
| Q9WUP4 | Polyprenol reductase | 0.74 |
| Q9WUR9 | GTP:AMP phosphotransferase AK4, mitochondrial | 0.73 |
| Q9WVL0 | Maleylacetoacetate isomerase | 0.76 |
| Q9Z0H1 | WD repeat-containing protein 46 | 1.19 |
| Q9Z0J0 | Epididymal secretory protein E1 | 0.77 |
| Q9Z0M5 | Lysosomal acid lipase/cholesteryl ester hydrolase | 0.61 |
| Q9Z130 | Heterogeneous nuclear ribonucleoprotein D-like | 1.51 |
| Q9Z1E4 | Glycogen [starch] synthase, muscle | 0.75 |
| Q9Z1P6 | NADH dehydrogenase [ubiquinone] 1 alpha subcomplex subunit 7 | 1.35 |
| Q9Z1S0 | Mitotic checkpoint serine/threonine-protein kinase BUB1 beta | 1.20 |
| Q9Z2E2 | Methyl-CpG-binding domain protein 1 | 0.67 |
| Q9Z2F2 | 2-5-oligoadenylate synthase-like protein 2 | 0.69 |
|  |  |  |
